# Gain and loss of polyadenylation signals during evolution of green algae

**DOI:** 10.1186/1471-2148-7-65

**Published:** 2007-04-18

**Authors:** Sabina Wodniok, Andreas Simon, Gernot Glöckner, Burkhard Becker

**Affiliations:** 1 Botanisches Institut, Universität zu Köln, Gyrhofstr. 15, 50931 Köln, Germany; 2 Genome Analysis, FLI, Beutenbergstr. 11, 07745 Jena, Germany

## Abstract

**Background:**

The Viridiplantae (green algae and land plants) consist of two monophyletic lineages: the Chlorophyta and the Streptophyta. Most green algae belong to the Chlorophyta, while the Streptophyta include all land plants and a small group of freshwater algae known as Charophyceae. Eukaryotes attach a poly-A tail to the 3' ends of most nuclear-encoded mRNAs. In embryophytes, animals and fungi, the signal for polyadenylation contains an A-rich sequence (often AAUAAA or related sequence) 13 to 30 nucleotides upstream from the cleavage site, which is commonly referred to as the near upstream element (NUE). However, it has been reported that the pentanucleotide UGUAA is used as polyadenylation signal for some genes in volvocalean algae.

**Results:**

We set out to investigate polyadenylation signal differences between streptophytes and chlorophytes that may have emerged shortly after the evolutionary split between Streptophyta and Chlorophyta. We therefore analyzed expressed genes (ESTs) from three streptophyte algae, *Mesostigma viride*, *Klebsormidium subtile *and *Coleochaete scutata*, and from two early-branching chlorophytes, *Pyramimonas parkeae *and *Scherffelia dubia*. In addition, to extend the database, our analyses included ESTs from six other chlorophytes (*Acetabularia acetabulum*, *Chlamydomonas reinhardtii*, *Helicosporidium *sp. ex Simulium jonesii, *Prototheca wickerhamii, Scenedesmus obliquus *and *Ulva linza*) and one streptophyte (*Closterium peracerosum*). Our results indicate that polyadenylation signals in green algae vary widely. The UGUAA motif is confined to late-branching Chlorophyta. Most streptophyte algae do not have an A-rich sequence motif like that in embryophytes, animals and fungi. We observed polyadenylation signals similar to those of *Arabidopsis *and other land plants only in *Mesostigma*.

**Conclusion:**

Polyadenylation signals in green algae show considerable variation. A new NUE (UGUAA) was invented in derived chlorophytes and replaced not only the A-rich NUE but the complete poly(A) signal in all chlorophytes investigated except *Scherffelia *(only NUE replaced) and *Pyramimonas *(UGUAA completely missing). The UGUAA element is completely absent from streptophytes. However, the structure of the poly(A) signal was often modified in streptophyte algae. In most species investigated, an A-rich NUE is missing; instead, these species seem to rely mainly on U-rich elements.

## Background

In eukaryotes, a polyadenylate tail [poly(A)] is attached to the cleaved 3' end of the nuclear-encoded precursor mRNA of most genes [1]. Polyadenylation is important for the regulation of mRNA stability and also affects translational capacity [2]. The general mechanism of polyadenylation is well understood in yeast and animals [3]. It requires two major components: poly(A) signals (cis-elements) on the pre-mRNA, and a protein complex (trans-acting factors) that carries out the cleavage of the pre-mRNA and the addition of the poly(A). The protein complex is conserved among organisms, but the poly(A) signals show considerable variation among species.

Five classes of cis-acting DNA elements have been identified [4-6] that facilitate polyadenylation: a far upstream element (FUE), a near upstream element (NUE), the cleavage site (CS) and a downstream element (DSE). The CS is surrounded by a cleavage element (CE) in *Arabidopsis *[CE, 4]. The FUE is generally U-rich. There are one or more NUEs, often AAUAAA or a related sequence 13 to 30 nt upstream of the CS (in the following, nucleotide positions are always given relative to the CS), each peculiar to its own cleavage site (CS). Only in animals is there an additional DSE [5,7-9] and the NUE AAUAAA is the major polyadenylation element. In contrast, in embryophytes (land plants), the strict AAUAAA element becomes a minor component and is replaced by variable A-rich sequences. Recently, Loke et al. [4] analyzed mRNA polyadenylation in *Arabidopsis *using the genome sequence and all available cDNAs. They confirmed the absence of highly-conserved consensus signal patterns and showed that in *Arabidopsis *the poly(A) signal consists of a U-rich FUE (-25 to -160), an A-rich NUE (about – 20) and CE. The CE consists of U-rich sequences on both sides of the CS. Furthermore, they presented evidence for the formation of secondary RNA structures in the 3'-UTR. Because known mutations in these regions affect polyadenylation, Loke et al. [4] suggested that the secondary structures might play an important role in the process.

The Viridiplantae (literally meaning green plants), which include all green algae and embryophyte plants, represent a monophyletic group of organisms, which display a surprising diversity in respect of morphology, cell architecture, life histories and reproduction, and in their biochemistry. The Viridiplantae consist of two monophyletic lineages: the Chlorophyta and the Streptophyta [10]. The Chlorophyta comprise the vast majority of green algae including most scaly green flagellates (e.g. *Pyramimonas*, *Tetraselmis*), the Ulvophyceae (e.g. *Ulva*, *Acetabularia*), Chlorophyceae (e.g. *Chlamydomonas*, *Volvox*) and Trebouxiophyceae (e.g. *Chlorella*) [11-13]. The Streptophyta include all embryophyte plants and a diverse paraphyletic assemblage of freshwater green algae, the Mesostigmatales, Chlorokybales, Klebsormidiales, Zygnematales, Coleochaetales and Charales (stoneworts) [13,14]. The Charales are widely believed to be the sister group of the embryophytes, suggesting that the evolution of true land plants started with an already complex organism [15]. However, the position of the Charales is still controversial [16]. Remarkably, only a single scaly green flagellate, *Mesostigma viride *Lauterborn, has been found to belong to the Streptophyta [17-20].

For a few chlorophyte mRNAs (mainly tubulins) an alternative polyadenylation signal (UGUAA) has been suggested [21-24], and it has been proposed that chlorophytes generally use the UGUAA motif instead of AAUAAA as NUE [25]. So far, UGUAA has only been found in chlorophyte algae, which are thought to have branched deeply from the last common ancestor of Viridiplantae [20]. To test whether UGUAA might be a chlorophyte-specific poly(A) signal we have analyzed the ESTs of chlorophyte and streptophyte algae available from public databases. To include more and especially early-branching species, we have sequenced ESTs from the flagellate *Mesostigma viride *[18], the filamentous algae *Klebsormidium subtile *and *Coleochaete scutata*, the flagellate *Pyramimonas parkeae *and the thecate flagellate *Scherffelia dubia *[26]. The first three are streptophytes, whereas the latter two represent chlorophytes. Our results indicate that derived chlorophyte algae use mainly UGUAA, whereas most streptophyte algae prefer U-rich sequences. Interestingly, only in *Mesostigma *have we found a polyadenylation signal similar to that in *Arabidopsis *and other embryophytes.

## Results

### Data sets used

We sequenced ESTs from cDNA libraries for *Mesostigma viride *[18], *Klebsormidium subtile*, *Coleochaete scutata*, *Pyramimonas parkeae *and *Scherffelia dubia*. Details on the preparation and assembly of contigs are given in Materials and Methods. To extend our database on polyadenylation in green algae, all ESTs from chlorophyte and streptophyte algae were downloaded from GenBank and TIGR (June 2006). To perform our analysis we relied on information about the orientation of EST sequence data. Thus, only data in which the polyA tail indicated the orientation could be used. For this reason, we could include only the following organismal data sets: *Chlamydomonas*, *Helicosporidium*, *Acetabularia*, *Prototheca*, *Ulva*, *Scenedesmus *(all chlorophytes) and *Closterium (streptophyte)*. For each data set, the origin, total number of RNAs and number of non-redundant poly(A)-containing mRNAs, and the base compositions of the 200 nt upstream from the CS, are given in Table 1.

**Table 1:** Sources and general characteristics of the organismal datasets used

**Organism**	**Source**	**No of ESTs or mRNAs**	**No sequences analyzed**^1)^	**nt composition**^2)^
**Chlorophyta**				
*Acetabularia*	Genbank	1002	28	A 36.3%
				C 9.5%
				G 19.6%
				U 37.6%
*Chlamydomonas*	TIGR	31608	10508	A 21.2%
				G 30,9%
				C 24,8%
				U 23,1%
*Helicosporidium*	Genbank	1229	359	A 21.1%
				C 26,6%
				G 29,0%
				U 23,3%
*Prototheca*	Genbank	5906	292	A 20.3%
				C 31.2%
				G 28.5%
				U 20.0%
*Pyramimonas*	this study	5034	1260	A 24.2%
				C 21.5%
				G 23.3%
				U 27.0%
*Scenedesmus*	Genbank	6016	265	A 20.1%
				C 23.8%
				G 31.2%
				U 24.9%
*Scherffelia dubia*	[26], and this study	1032	110	A 25.7%
				C 26.0%
				G 26.8%
				U 21.5%
*Ulva linza*	Genbank	1888	54	A 22.3%
				C 22.3%
				G 28.6%
				U 26.8%
**Streptophyta**				
*Closterium*	Genbank	1201	136	A 3.6%
				C 27.6%
				G 33.3%
				U 35.5%
*Coleochaete*	this study	5094	142	A 27.2%
				C 18,9%
				G 22,6%
				U 31,3%
*Klebsormidium*	this study	4651	473	A 27.8%
				C 20.5%
				G 25.6%
				U 26.1%
*Mesostigma*	[18]	10395	1327	A 26.9%
				C 20.6%
				G 23.1%
				U 29.4%

^1) ^No of non-redundant sequences with a poly(A)-tail analyzed, ^2) ^within 200 nt upstream from the CS.

### Positional nucleotide frequencies upstream of the CS

**Figure 1:**
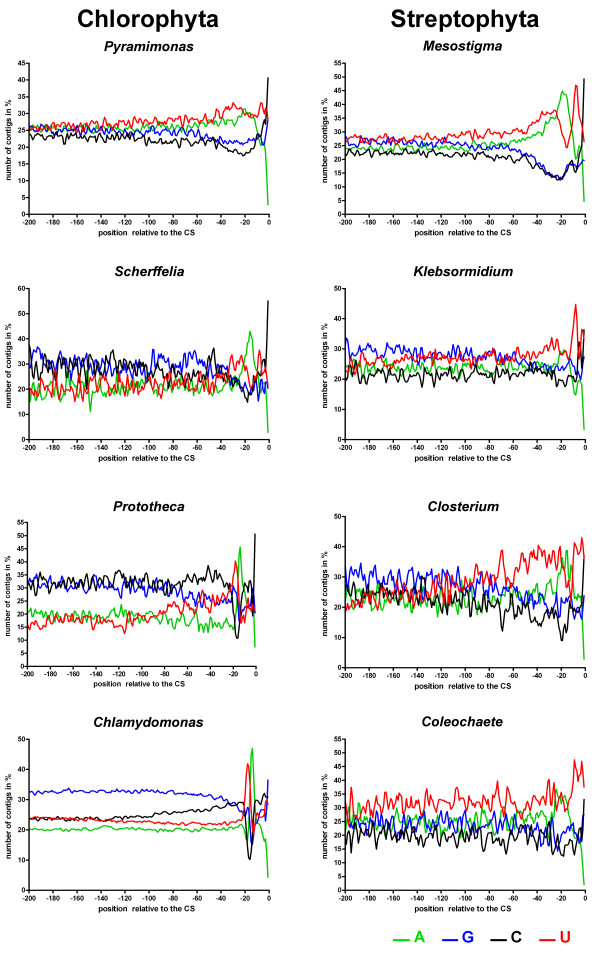
**Single-nucleotide profiles of the 3'UTR in various green algae**. Single-nucleotide frequencies within the 200 nt upstream from the CS are shown. For clarity, smoothed curves using the weighted average of 5 neighbours method are shown. The original point graphs are depicted in supplementary Fig. 1.

For all data sets, we established the 1 nt pattern of the 200 nt upstream from the CS and the frequencies of penta- and hexanucleotide words within the first 50 nt upstream from the CS. The 1 nt patterns of selected genera are shown in Fig. 1.

For all organisms, we observed a clear change in nucleotide frequencies around 20 nt upstream from the CS (Fig. 1) indicative of a putative poly(A) signal. In all chlorophytes except *Pyramimonas*, there is a sharp peak of U followed by sharp peak of A. In contrast, in *Pyramimonas *and all streptophytes, we observed a broad peak of U followed by peaks of A and then U again. These changes are easier to see in large organismal data sets than in small ones, as variations in nucleotide frequencies at individual positions relative to the CS are higher in the latter. For this reason the curves presented in Fig 1 were smoothed using the weighted average of 5 neighbors method. The original point graphs are available as Fig. S1 [see Additional file 1].

### Poly(A) signals in Chlorophyta

A short Python program (available from the authors) was written to scan the 50 nt upstream from the CS for the occurrence of penta- and hexanucleotide words in the various organismal data sets. The base composition differs considerably among the various chlorophytes (Table 1). For this reason, the frequency of the UGUAA-motif expected by chance within 50 nt upstream from the CS varies between 2 and 12%. To test whether the observed over-representation of penta- and hexanucleotide words was statistically significant, we calculated the log odds ratio (lnω) and its 95% confidence interval for each word [27, see Methods for details]). Table S1 [see Additional file 2] lists all penta- and hexanucleotide words for each organism that are at least 2.7 times (lnω > 1) more frequent than expected by chance and for which the over-representation is statistically significant. Many penta- and hexanucleotide words fulfilling this criteria show considerable overlap and can be arranged into a few clusters centered on the most significantly over-represented word [see Additional file 2]. Some of these clusters occur in frequencies too low (although statistically significant) to be considered as putative polyadenylation signals and might represent regulatory sites for a subset of mRNAs from an organism. Further analysis is required to investigate this possibility.

**Figure 2:**
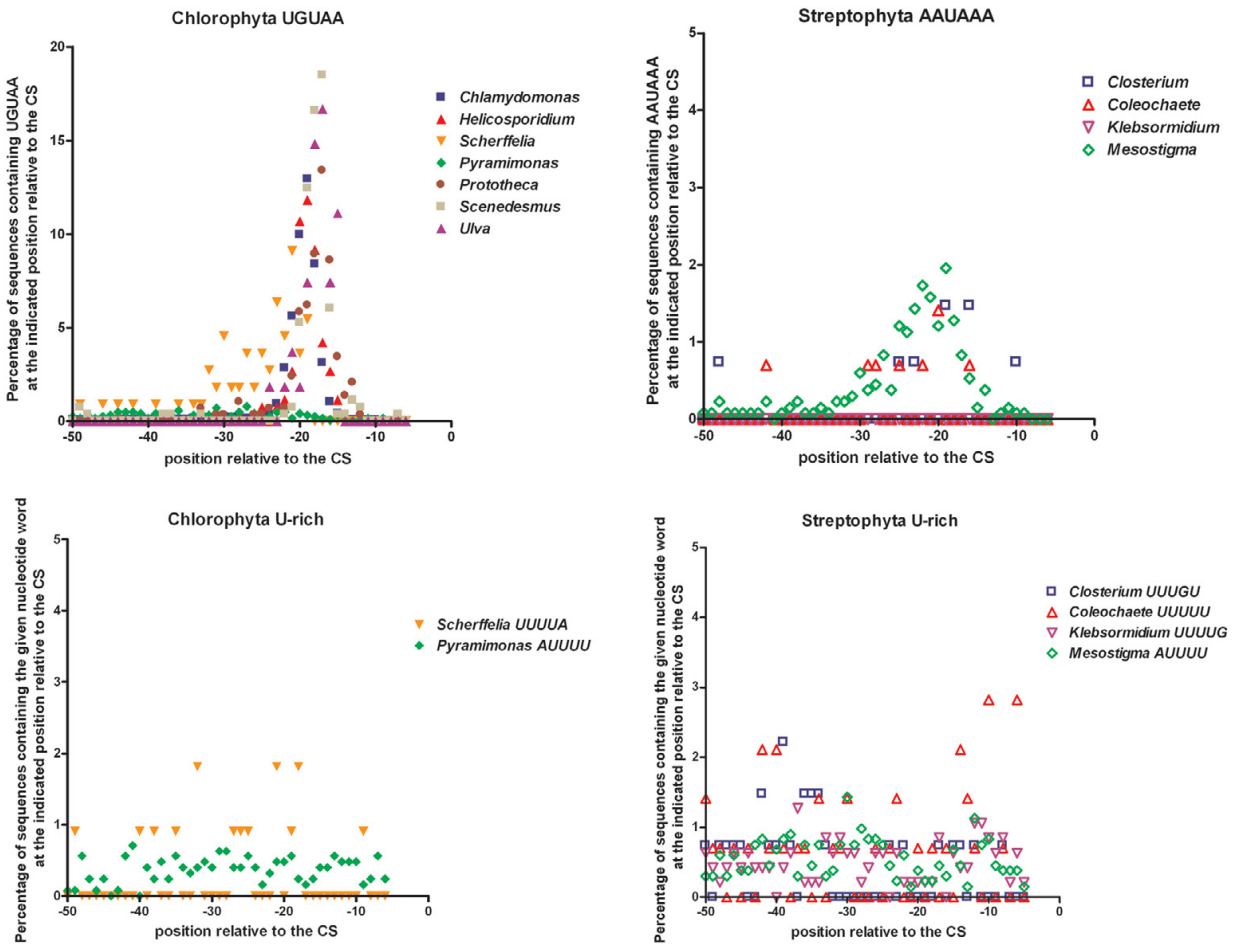
**Distribution of (putative) polyadenylation signals within 50 nt upstream from the CS in different chlorophyte and streptophyte algae**. Distribution of the (putative) polyadenylation signals UGUAA, AAUAAA and U-rich within 50 nt upstream from the CS in different chlorophyte and streptophyte algae.

Table 2 lists the two most significantly over-represented penta- and hexanucleotide words for each organism. It is evident from this table that UGUAA is the top pentanucleotide pattern in all chlorophytes except *Pyramimonas*. On average, about 60% of the sequences contain an UGUAA motif within 50 nucleotides upstream from the CS. The second to sixth most frequent pentanucleotide words overlap with UGUAA [see Additional file 2], highlighting the importance of this motif. NUE are generally found around -15 to -20 nt upstream from the CS. To test whether the UGUAA motif shows a similar distribution, we plotted the percentage of sequences within an organismal data set containing an UGUAA motif at a certain position upstream of the CS against the position relative to the CS. Fig 2 shows the results. In all chlorophytes except *Pyramimonas*, the UGUAA motif peaks sharply around position -17.

**Table 2:** Frequencies of penta- and hexanucleotide words within 50 nt upstream from the CS

**Organism**	**nt at position -1**	**Pentanucleotide words**	**log odds ratio**	**Hexanucleotide words**	**log odds ratio**
**Chlorophyta**					
*Acetabularia*	U 57.1%	UGUAA 71.4%	2.52	AUGUAA 42.9%	1.34
	C 14.3%	UUUGU 50.0%	1.51	UGUAAU 32.9%	0.79
	G 28.6%				
*Chlamydomonas*	G 42.4%	UGUAA 49.8%	3.27	UGUAAC 20.0%	3.33
	C 31.5%	GUAAC 23.1%	2.03	CUGUAA 15.2%	3.00
	U 26.1%				
*Helicosporidium*	C 51.5%	UGUAA 47.3%	3.29	UGUAAG 16.4%	3.06
	T 24.6%	UUGUA 20.6%	1.95	UGUAAC 14.5%	3.00
	G 23.9%				
*Prototheca*	C 61.6%	UGUAA 56.8%	4.12	UGUAAC 32.5%	4.27
	G 19.2%	GUAAC 40.0%	2.81	CUGUAA 23.6%	3.82
	U 19.2%				
*Pyramimonas*	C 45.1%	UUUUG 17.0%	1.22	AAAAAA 6.0%	1.94
	G 28.4%	AUUUU 17.5%	1.21	UUUUUG 8.1%	1.75
	U 26.5%				
*Scenedesmus*	C 38.6%	GUAAC 68.7%	4.12	UGUAAC 52.1%	4.89
	G 34.1%	UGUAA 66.4%	3.97	GUAACA 36.6%	4.44
	U 27.3%				
*Scherffelia*	C 62.7%	UGUAA 61.8%	3.76	UGUAAA 27.3%	3.65
	U 19,1%	UUGUA 19.1%	2.13	UUGUAA 16.3%	3.15
	G 18.2%				
*Ulva*	C 61.1%	UGUAA 66.7%	3.76	UGUAAC 24.1%	3.65
	U 22.2%	GUAAC 31.9%	2.42	UUGUAA 24.1%	3.15
	G 16.7%				
**Streptophyta**					
*Closterium*	C 41.9%	UGUAA 22.8%	3.41	AUUGUA 12.5%	2.96
	U 33.8%	AAUGU 17.6%	3.36	UAUAAU 8.8%	2.66
	G 24.3%				
*Coleochaete*	C 38.0%	UUUUG 31.7%	1.43	UUUUUU 21.1%	1.80
	U 34.5%	UGUUU 19.6%	1.33	UGUUUU 17.6%	1.91
	G 27.5%				
*Klebsormidium*	C 41.3%	CCCCC 10.1%	1.90	CCCCCC 5.9%	2.93
	U 30.1%	CCCUU 13.3%	1.07	CCCCCU 5.5%	2.30
	G 28.6%				

*Mesostigma*	C 54.8%	AAUAA 29.1%	1.68	AAUAAA 19.1%	1.95
	U 26.2%	AUAAA 28.0%	1.53	AAUUAA 15.6%	1.62
	G 19.0%				

In chlorophytes, the AAUAAA poly(A) signal was observed among the top 50 hexanucleotide words within 50 nt upstream from the CS only in *Scherffelia *[see Additional file 2]. We found AAUAAA in 10% of the sequences from *Scherffelia *investigated (1.2% expected by chance). This observed over-representation of the AAUAAA motif is statistically significant [see Additional file 2]. However, in *Scherffelia*, the AAUAAA motif shows a broad distribution and is not restricted to positions -13 to -20 [see Additional file 3]. Similarly, we observed a statistically significant increase of U-rich sequences between -1 and -50 upstream from the CS only for *Pyramimonas *(Table 1) and *Scherffelia *(UUUUA: found in 13.5%, expected 2.4%). Again, the U-rich sequences showed a broad distribution within the 50 nt upstream from the CS (Fig. 2).

### Poly(A) signals in streptophyte algae

In all streptophyte algae analyzed, the chlorophyte-specific UGUAA-motif was present more often within 50 nt upstream from the CS than expected by chance [see Additional file 2]. The over-representation of UGUAA is statistically significant, but the UGUAA-motif showed a more or less random distribution between -1 and -50 nt upstream from the CS [see Additional file 1] for all four streptophyte algae.

We observed significant numbers of the AAUAAA motif only for *Mesostigma*, *Closterium *and *Coleochaete*, whereas it was not over-represented between -1 and -50 upstream from the CS in *Klebsormidium*. However, AAUAAA was enriched between -13 and -25 nt upstream from the CS only in *Mesostigma *(Fig. 2). Some single base variations (UAUAAA and AAUUAA) show the same distribution as the motif AAUAAA within 50 nt upstream from the CS, indicating that AAUAAA-like sequences might function as a NUE of the poly(A) signal in *Mesostigma *in the same way as in land plants. In all streptophyte algae, we observed significant increases in U-rich sequences around -10 and -30 to -40 nt upstream from the CS (Fig. 2).

### Number of NUE per transcript

On average, about 8.2 % of the contigs from chlorophytes (except *Pyramimonas*) and *Mesostigma *contained more than a single copy of the putative poly (A) signals UGUAA or AAUAAA, respectively, within 50 nt upstream from the CS (Table 3). Inspection of the mRNAs comprising these contigs revealed that about 15% of those with two putative poly(A) signals (0.5 % of all contigs) were assembled from two mRNA species differing only in the positions of their poly(A) tails (Table 3). In all cases, one polyadenylation site seemed to be preferred over the other.

**Table 3:** Number of expressed genes containing multiple putative poly(A) signals

Organism	No of expressed genes containing at least one putative poly(A) signal	No of expressed genes containing two putative poly(A) signals	No of expressed genes containing two putative poly(A) signals for which different CS were found	Distance between poly(A) signal und CS for expressed genes with mRNA isoforms
**Chlorophyta**				
*Acetabularia*	20	6	1	26/27
*Chlamydomonas*	5232	160	54	15–23
*Heliosporidium*	124	2	2	18/17 19/23
*Prototheca*	166	4	2	21/18 17/18
*Scenedesmus*	182	5	0	-
*Scherffelia*	68	3	0	-
*Ulva*	36	1	0	-
**Streptophyta**				
*Mesostigma*	386	40	5	9–45

## Discussion

Analysis of the 3'-UTRs of a large set of genes from different chlorophytes and streptophytes revealed major differences in putative polyadenylation signals between chlorophyte and streptophyte algae. Both AAUAAA and UGUAA motifs have previously been described as possible NUE motifs in green algae [25][28]. However, it was not clear how these motifs were distributed phylogenetically. We detected a clear putative AAUAAA-like NUE only in *Mesostigma*. The other streptophyte algae investigated seem to have lost the AAUAAA-like NUEs, although a weak enrichment of A around position -17 can still be detected in 1 nt positional frequency plots (Fig. 1). We found the UGUAA motif in all chlorophytes except *Pyramimonas*. The UGUAA motif in chlorophytes was not randomly distributed within the 50 nt upstream from the CS investigated, but showed a clear peak at -10 to -30 upstream from the CS. Although there is currently no experimental proof that UGUAA is a poly(A) signal, its narrow distribution and its presence in all chlorophytes investigated except *Pyramimonas *is a strong indication that this signal is indeed functional and plays a role in mRNA processing. It could be argued that some data sets are still too small to identify poly(A) signals. Indeed, if we had used only a single data set (e.g. the 28 *Acetabularia *sequences) we could not have drawn this conclusion. However, the over-representation and narrow distribution of UGUAA within 50 nt upstream of the CS in 7 different chlorophytes (including chlorophycean, ulvophycean, trebouxiophycean algae) that vary greatly in GC content (Table 1) make us confident that UGUAA is probably a true polyadenylation signal.

For some species we lowered the criterion for recognizing a poly(A) tail to a stretch of 5 terminal adenines. This was required because many researchers trim their poly(A) tails. For example, in the original *Chlamydomonas *Gene Index as downloaded from TIGR, only 7 out of 31608 sequences contained a stretch of 10 or more terminal adenines. Lowering the criterion to a stretch of 5 terminal adenines and including sequences that start with oligo (T) stretches (possibly representing the reverse complement of mRNAs) increased the number to 10508 sequences. Manual inspection revealed that many of these sequences contained the putative UGUAA poly(A) signal, and indeed we detected the UGUAA motif between -10 and -30 from the CS in about 50% of the sequences. However, as a stretch of 5 adenines can also be found in protein-coding sequences, we cannot exclude the possibility that our data set includes some internal sequences from mRNAs rather than the poly(A)-preceding sequence. Therefore, some of the numbers presented for the organismal data sets downloaded from public databases, which include all late-branching chlorophytes, might actually be too low, making the number of mRNAs possessing the UGUAA motif even higher.

It has been shown that A-rich NUE are necessary for polyadenylation in embryophytes and animals [3,7]. In yeast, U-rich upstream elements are even more important than the NUEs for facilitating polyadenylation [29]. These FUEs are also found in animals and embryophytes [5,6]. The absence of U-rich sequences in the 250 nt upstream from the CS in *Chlamydomonas *and other chlorophytes may indicate that the putative UGUAA-sequence-dependent polyadenylation involves a different mechanism than from A-rich NUE-dependent polyadenylation, although homologues to the known protein machinery for polyadenylation in other eukaryotes can be detected in the *Chlamydomonas *genome. Alternatively, other sequence motifs may be required in addition to the UGUAA signals for proper polyadenylation, although we failed to detect other nucleotide words that were significantly enriched in all chlorophytes.

No clear NUE (AAUAAA-like or UGUAA) is present in *Closterium*, *Klebsormidium *or *Coleochaete*. The lack of a UGUAA motif appears to be a general feature of streptophytes as this sequence motif was also not detected in *Mesostigma *(this study), *Arabidopsis *or rice [4,5]. Given the presence of AAUAAA-like NUE in *Mesostigma *(this study) and land plants [4,5,8], its complete absence from the other streptophyte algae is surprising. Instead, the sequence motifs most often found are U-rich. We propose that these sequence motifs represent FUEs facilitating polyadenylation, as in yeast [5,29]. However, the number of mRNAs with a poly(A) tail investigated is still rather low for these species and there is still no direct proof for the proposed function of U-rich sequences in *Closterium*, *Klebsormidium *and *Coleochaete*. In addition, it is known that auto-correlated sequences such as oligo(U) show increased variance, i.e. there is a higher probability of observing higher or lower word counts than expected [30]. Therefore, the significance of the occurrence of such auto-correlated patterns in our analyses is not yet clear, as we cannot exclude the possibility that a NUE could be detected in a larger data set for each organism. In this context, it would be most interesting to know the structures of polyadenylation signals in the Charales. Currently, the Charales are considered to be a sister group to the embryophyte lineage [15]. EST-projects for two *Chara *strains are underway in Japan and the United States; however, no data have been released yet into the public domain. At present, only 28 mRNAs from *Chara *species can be found in Genbank, and a clear poly(A)-tail is recognizable only for the nuclear-encoded GAPDHB from *Chara vulgaris *[31]. This sequence does not contain either UGUAA or AAUAAA within 50 nt upstream from the CS. However, a U-rich region is present about 60 nt upstream from the CS. Whether this is typical for the Charales as for *Klebsormidium *and *Coleochaete *remains to be seen.

In plants and algae, the usage of multiple polyadenylation signals within a single gene, leading to different mRNA species, has been reported [7,32]. As we observed this phenomenon only in 0.5% of the contigs (expressed genes) investigated, it may not be prominent within green algae. Another type of mRNA variation observed in mammals is the use of different CS downstream from a single polyadenylation signal [33]. In mammals, this seems to occur in a considerable percentage (22–44%) of cases [33]. Polyadenylation heterogeneity is tissue-specific in mammals [34] and has been related to mRNA stability and/or translation efficiency [34]. We encountered only one similar situation during our study. For the rbcs gene from *Scherffelia *we observed two mRNA species. In one of these (35% of sequenced ESTs), polyadenylation started 14 nt downstream from the UGUAA motif; in the other (65% of sequenced ESTs), it started 26 nt downstream from the same UGUAA motif. We do not yet know the functional significance of this finding or whether it is a common phenomenon in green algae.

**Figure 3:**
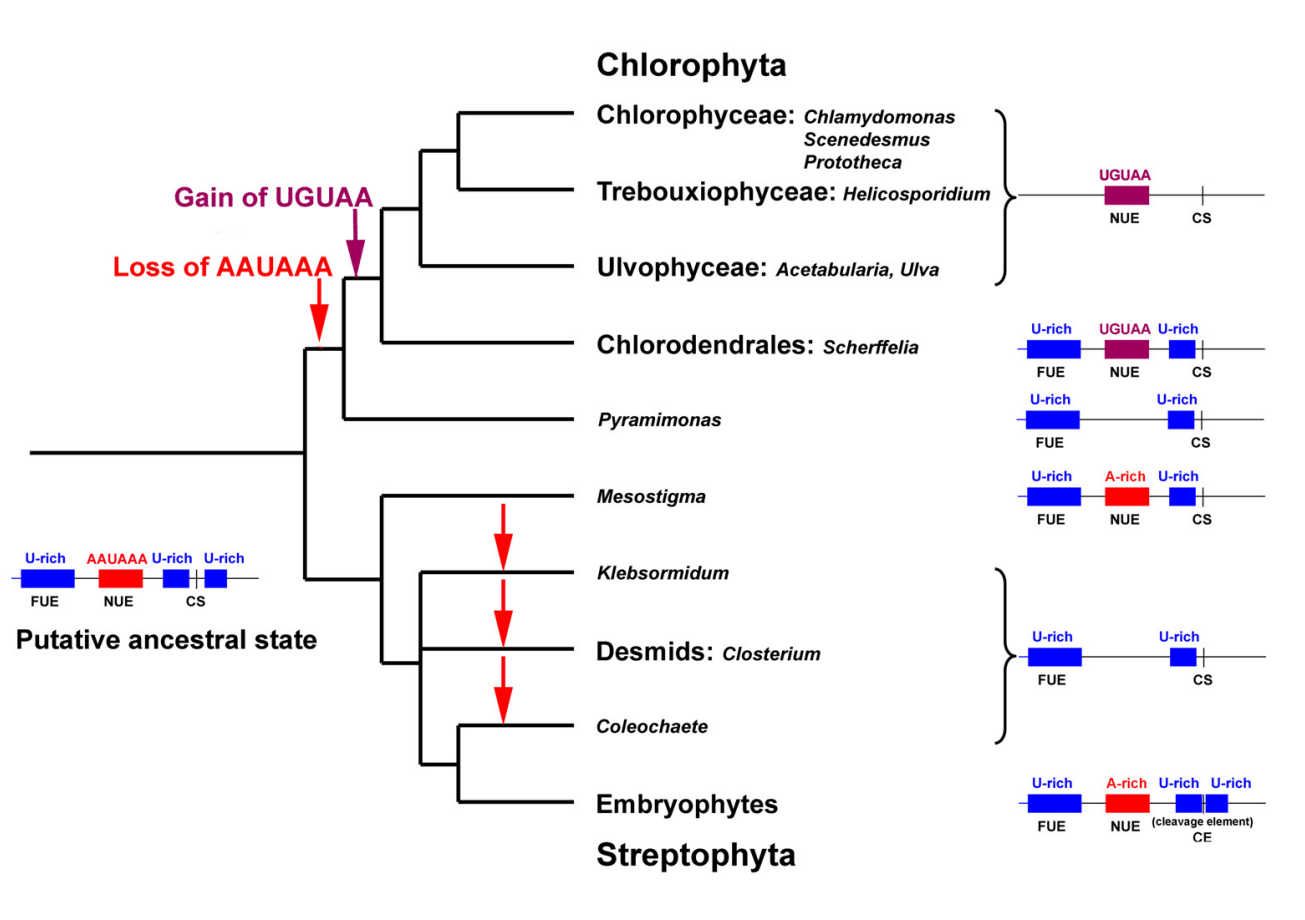
**Drawing showing the phylogenetic relationships for the organisms investigated**. The structure of the putative organismal and the ancestral poly(A) signal is indicated. Loss of the AAUAAA-like signal is indicated with a red arrow and the gain of the UGUAA signal is indicated with a purple arrow.

Fig. 3 summarizes our findings concerning the occurrence of poly(A) signals in the Viridiplantae in an evolutionary context. Two scenarios for the evolution of poly(A) signals in Viridiplantae seem possible. In the first (presented in Fig. 3), the present-day embryophyte signals were already present in the last common ancestor and were lost differentially during the evolution of the different green algal lineages. Only the direct phylogenetic lineage to *Mesostigma *conserved this motif set. Concurrently, the UGUAA signal evolved as a replacement in the Chlorophyte lineage. In the second scenario, polyadenylation in the last common ancestor depended only on U-rich sequences. Then two different additional signals evolved, constituting either an addition to the existing signals (embryophytes) or replacing them (Chlorophyta). We favor the first scenario for the following reason. Polyadenylation is a typical eukaryotic feature and therefore evolutionarily old. Animals, plants and fungi use AAUAAA or A-rich sequences as NUE, and U-rich sequences as additional elements [see introduction for details, see also [6]]). Therefore, either plants, animals and fungi inherited the general structure of the polyadenylation signals from their last common ancestor, or the similar structures of their poly(A) signals indicate convergent evolution. The latter hypothesis seems to us less likely.

If the first scenario outlined above is correct, the proposed replacement of AAUAAA with UGUAA in chlorophytes occurred stepwise and early during chlorophyte evolution. The A-rich NUE was lost in *Pyramimonas*, UGUAA and U-rich elements are present in *Scherffelia*, and UGUAA is present and U-rich elements are absent in all the other late branching chlorophytes investigated. Once the UGA motif was established in late branching chlorophytes, it seems to have been under strong evolutionary pressure, as it has changed so little in the various chlorophyte lineages (compare the observed variation in streptophytes). The gain of the UGUAA-motif as poly(A) signal in *Scherffelia *correlates with major evolutionary transitions such as the conversion of an extracellular matrix consisting of scales into a cell wall, changes in mitosis and cytokinesis and other major cellular changes [12,13,35]. Our finding that *Scherffelia *(a member of the Chlorodendrales), like the other late-branching chlorophyte classes Ulvophyceae, Trebouxiophyceae and Chlorophyceae (UTC clade), possesses a UGUAA-motif in the 3'UTR supports the sister relationship between the Chlorodendrales and the UTC clade as revealed my molecular phylogenetic analyses [13]. The shared derived change in polyadenylation mechanism might represent the first synapomorphic character uniting the Chlorodendrales and the UTC clade.

## Conclusion

Our results show that putative poly(A) signals may vary considerably among organisms. In chlorophytes (except *Pyramimonas*) the A-rich NUE was completely replaced by the UGUAA-motif, which represents a synampomorphic character of the Chlorodendrales and the UTC-clade. Also, the structure of the poly(A) signal was modified in many streptophytes. There is no A-rich NUE and the organisms seem to rely exclusively on U-rich elements.

## Methods

### Data selection

We retrieved all available EST sequences for *Acetabularia acetabulum*, *Closterium peracerosum*, *Helicosporidium *sp. ex Simulium jonesii, *Prototheca wickerhamii*, *Scenedesmus obliquus*, and *Ulva linza *from Genbank. The *Chlamydomonas reinhardtii *Gene index was downloaded from the TIGR database. Additional ESTs were sequenced for *Scherffelia dubia *using the cDNA library described in Becker et al. [26]. The sequences were deposited in EMBL/Genbank under the accession nos. AJ919283 – AJ919992. We prepared cDNA-libraries from *Pyramimonas parkeae *(M1663, courtesy of Prof. Dr. M. Melkonian, Botanical Institute, University of Cologne), *Klebsormidium subtile *(CCAC 0119, Culture Collection of Algae at the University of Cologne) and *Coleochaete scutata *(M0493, courtesy of Prof. Dr. M. Melkonian, Botanical Institute, University of Cologne) as follows. *Klebsormidium subtile *and *Coleochaete scutata *were cultured in modified WARIS solution as described by Simon et al. [18]. *Pyramimonas parkeae *was cultured in modified ASP medium [36]. mRNA was isolated from interphase cultures using the mRNA Isolation Kit (Roche Applied Science). mRNAs (50 ng) were converted into cDNA using the CapFishing™Full-length cDNA Premix Kit (Seegene) and then amplified by PCR with either of the following primer combinations: 5'-RACE (Seegene) and (5'-TTTTTTTTTTTTTTTTN3') (*Coleochaete scutata*), or 3'-RACE (Seegene) and C1 (5'-NNNNNNNNNNNNNNNATG-3') (*Klebsormidium subtile *and *Pyramimonas parkeae*). cDNA was size-fractioned by agarose gel electrophoresis and the smear between 500 bp-5000 bp was isolated and cloned into pGEM-T Easy vector (Promega). All libraries were transformed into ElectroMAX DH10B *E. coli *cells (Invitrogen) by electroporation.

Isolated plasmids were sequenced by the cycle sequencing method using an ABI3700 96 capillary sequencer. A minimal contig set was assembled using the phrap assembler and all contigs were manually curated.

### Sequence analysis

ESTs and cDNAs from public databases were checked for redundancy (within each organism specific data set) using the GCG software or assembled into contigs using the CAP-assembler of the Bioedit program. All non-redundant sequences from the different organisms were checked for the presence of a poly(A)-tail. Initially, sequences with at least 15 adenines in the 3'-end of the insert were assumed to include poly(A)-tails and were truncated to the base preceding the 5'-most adenine and trimmed to the 200 nt upstream the CS using the Bioedit editor. As we noticed that ESTs with fewer than 15 A at the 3'end contained a clear UGUAA about 20 nt upstream from the putative poly(A) in some species, we lowered the required number of As in the poly(A) tail to 5 to increase the number of sequences in the data sets. 1-nucleotide patterns were calculated for the 200 upstream from the CS using the Bioedit program.

The frequencies of all possible penta- and hexanucleotide patterns within the first 50 nt upstream from the CS were determined using a small Python script. The script is available from the authors upon request. To test whether the observed frequencies in penta- and hexanucleotide words were significantly different from chance we used the log-odds ratio (lnω) as described by Sokal and Rohlf [27]. Briefly, the expected oligonucleotide frequency F_e_(b) of oligonucleotide (b) was calculated using the collection of all 200 nt upstream regions for each organism. The expected oligonucleotide frequencies were then used to calculate the number of expected occurrences in a given organismal data set using the formula:

O_e_(b) = F_e_(b) × S × (L-w+1)

where

O_e_(b) = number of expected occurrences of oligonucleotide (b) in the organismal data set

S = number of sequences

L = sequence length

w = oligonucleotide length

The log-odds ratio lnω was calculated as follows

lnω = ln (q_1_/p_1_)/(q_2_/p_2_)

where

q_1 _= number of observed sequences containing oligonucleotide (b) as sequence motif

p_1 _= number of observed sequences **not **containing oligonucleotide (b) as sequence motif

q_2 _= number of sequences expected to contain oligonucleotide (b) as sequence motif

p_2 _= number of sequences expected **not **to contain oligonucleotide (b) as sequence motif

According to Sokol and Rohlf, the log-odds ratios are approximately normally distributed [27]. The standard deviation of the log-odds ratio was calculated using equation 17.19 given in Sokal and Rohlf [27] and used to calculate the upper and lower limits of a 95% confidence interval. If the lower limit is greater than zero the observed differences are statistically significant.

The sequences (200 nt upstream of the CS, in fasta format) of the non-redundant *Pyramimonas*, *Klebsormidium *and *Coleochaete *data sets are presented as data set S1 [see Additional file 4]. The full sequences will be made available with the first general publication on the ESTs from these organisms.

## Authors' contributions

AS prepared the Mesostigma cDNA library and participated in the sequence analysis. SW prepared the *Pyramimonas*, *Klebsormidium *and *Coleochaete *cDNA libraries, and participated in sequence analysis and helped to draft the manuscript. GG performed EST sequencing and helped to draft the manuscript. BB conceived of the study and participated in its design, performed data analysis, and helped to draft the manuscript. All authors read and approved the final manuscript.

## Supplementary Material

Additional file 1 **Figure S1: Single-nucleotide profiles of the 3'UTR in various green algae**. Single-nucleotide frequencies within the 200 nt upstream from the CS are shown for the indicated organisms as point graphs. Click here for file

Additional file 2 Table S1: The top 50 penta- and hexanucleotide words within 50 nt upstream from the CS in various chlorophyte and streptophyte algae. Click here for file

Additional file 3 **Figure S2: Distribution of various hexanucleotide words within 50 nt upstream from the CS in different chlorophyte and streptophyte algae**. Chlorophyte sequence motifs are depicted on the left, streptophyte sequence motifs on the right. Click here for file

Additional file 4 Data set S1: Sequences (200 nt upstream of the CS, in fasta format) of the non-redundant *Pyramimonas*, *Klebsormidium *and *Coleochaete *data sets. Click here for file
