# Supplementary figures and images for: Gain and loss of polyadenylation signals during evolution of green algae
Source: BMC Evol Biol. 2007 Apr 18;7:65. doi: 10.1186/1471-2148-7-65 (PMC1868727; doi:10.1186/1471-2148-7-65)

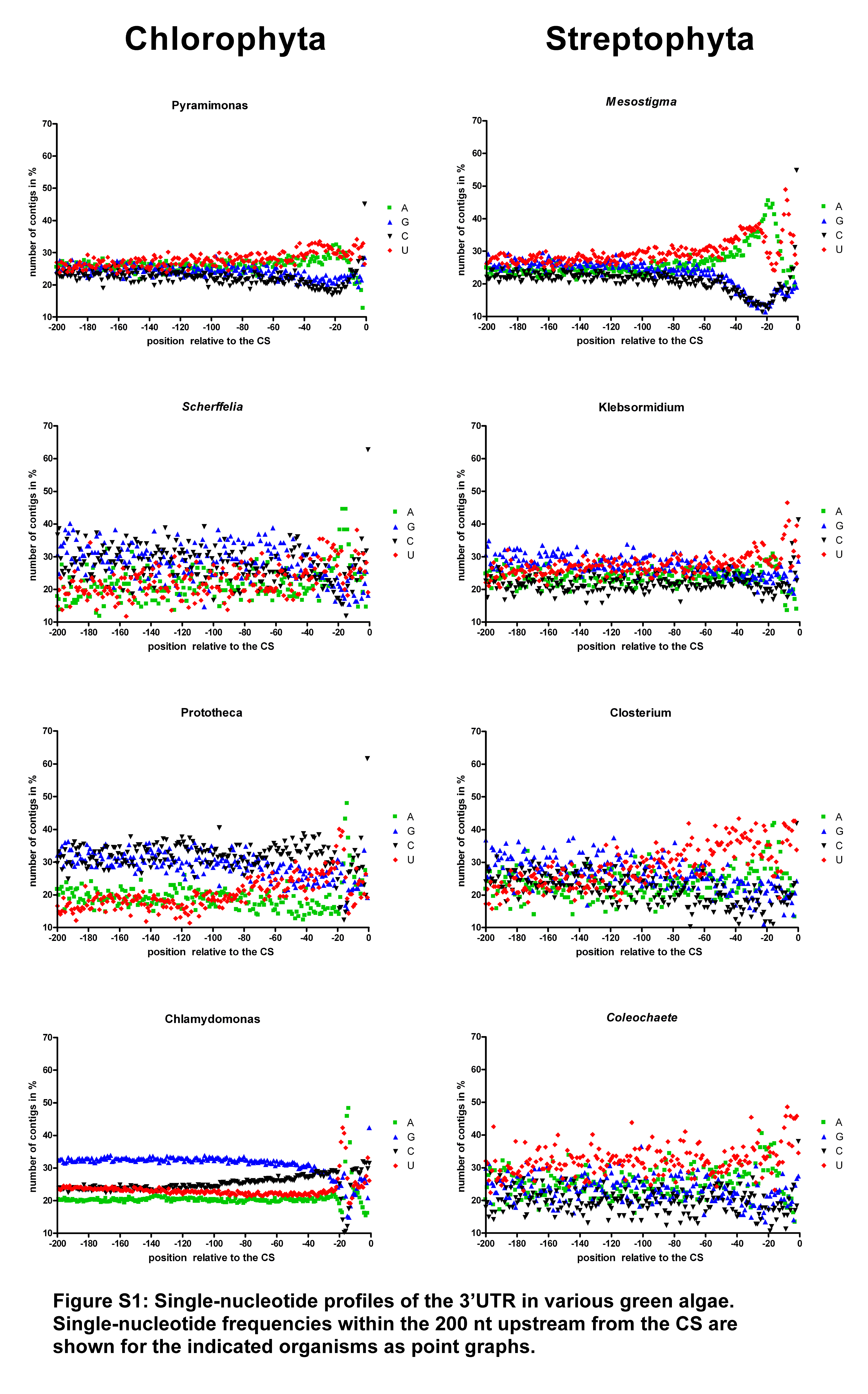

Supplement: Additional file 1 — Figure S1: Single-nucleotide profiles of the 3'UTR in various green algae. Single-nucleotide frequencies within the 200 nt upstream from the CS are shown for the indicated organisms as point graphs. [file 1471-2148-7-65-S1.jpeg]
