# Supplementary material for: Gain and loss of polyadenylation signals during evolution of green algae
Source: BMC Evol Biol. 2007 Apr 18;7:65. doi: 10.1186/1471-2148-7-65 (PMC1868727; doi:10.1186/1471-2148-7-65)
Supplement: Additional file 2 — Table S1: The top 50 penta- and hexanucleotide words within 50 nt upstream from the CS in various chlorophyte and streptophyte algae. [file 1471-2148-7-65-S2.doc]

### Table S1: List of all penta- and hexanucleotide words for each organism investigated that are at least 2.7 times (lnω > 1) more frequent than expected by chance and for which the over-representation is statistically significant. The penta- and hexanucleotide words have been arranged into overlapping sequence clusters. The putative poly(A) signalse UGUAA and AAUAAA are marked by bold letters.

*Acetabularia acetabulum*

N = 28

Base composition: A 36.3% C 9.5% G 19.6% U 37.6%

Pentanucleotide words

sequence observed expected log odds lower limit of 95% CI

AUGUA 12 5 1,31 0,07

**UGUAA** 20 5 2,52 1,23

GUAAA 12 5 1,35 0,10

UUUGU 14 5 1,51 0,30

Hexanucleotide words

AUGUAA 12 5 1,34 0,09

*Chlamydomonas reinhardtii*

N = 10508

Base composition: A 21.2% G 30,9% C 24,8% U 23,1%

Pentanucleotide words

sequence observed expected log odds lower limit of 95% CI

GUAAG 1260 496 1,01 0,90

GUAAU 1282 383 1,30 1,18

UAACC 986 319 1,20 1,07

UAACA 953 273 1,32 1,18

GUAAC 2432 398 2,03 1,92

GUAAA 1384 340 1,51 1,39

**UGUAA** 5232 383 3,27 3,16

CUGUA 1905 449 1,60 1,50

AUGUA 1583 383 1,54 1,43

CAUGU 1425 449 1,26 1,15

UUGUA 1509 432 1,36 1,25

GUGUA 1667 559 1,21 1,11

AACCC 1194 331 1,37 1,25

ACCCC 1156 388 1,17 1,05

GCCCC 1668 565 1,20 1,10

CCCCC 1548 453 1,34 1,23

CCCCA 1010 388 1,02 0,90

CCCCU 1340 437 1,21 1,10

CCCUU 1126 421 1,06 0,94

CCCUG 1365 544 1,01 0,90

UGCAA 1118 398 1,11 0,99

Hexanucleotide words

CUGUAA 1596 93 3,00 2,79

AUGUAA 1260 80 2,88 2,65

UUGUAA 1194 90 2,70 2,48

GUGUAA 1282 116 2,52 2,33

UGUAAA 1138 80 2,77 2,54

UGUAAU 1049 90 2,56 2,34

UGUAAG 1054 116 2,30 2,11

UGUAAC 2105 93 3,33 3,12

GUAACC 716 97 2,07 1,85

GUAACA 748 83 2,27 2,04

GUAACG 624 120 1,70 1,50

AACCCC 526 80 1,92 1,69

GCCCCC 713 137 1,71 1,52

CCCCCC 689 110 1,89 1,69

CCCCCG 548 137 1,43 1,24

CCCCCU 547 106 1,68 1,47

CCUGUA 581 109 1,72 1,51

GCUGUA 681 136 1,67 1,48

CAUGUA 674 93 2,04 1,82

UGUGUA 595 131 1,56 1,37

CGUGUA 532 136 1,41 1,22

*Helicosporidium spec.*

N = 262

Base composition: A 21.1% C 26,6% G 29,0% U 23,3%

Pentanucleotide words

sequence observed expected log odds lower limit of 95% CI

UGUGU 38 13 1,19 0,53

AUGUG 40 12 1,36 0,68

GUGUG 43 16 1,11 0,51

UGUGA 48 12 1,58 0,91

GUGUA 48 12 1,58 0,91

CUGUA 44 11 1,56 0,87

GUAAC 40 10 1,55 0,82

GUAAG 44 11 1,57 0,88

**UGUAA** 124 8 3,29 2,57

UUGUA 54 9 1,95 1,23

AUGUA 40 8 1,69 0,92

CAUGU 46 11 1,61 0,92

CAUGC 31 12 1,01 0,33

GCAUG 41 13 1,25 0,60

UGCAU 42 11 1,51 0,81

AUGCA 33 10 1,33 0,59

UGUUU 39 10 1,45 0,74

UUUUG 32 10 1,22 0,50

UUUGU 35 10 1,33 0,61

UUUGC 35 12 1,19 0,50

UUGCU 36 12 1,22 0,54

AUUGC 31 11 1,15 0,43

CGUGC 45 17 1,11 0,52

CGUGU 43 15 1,20 0,58

CUCUG 36 13 1,08 0,43

UCUGU 32 12 1,09 0,39

CUGUU 34 12 1,15 0,47

UGUUG 33 13 1,03 0,36

UUGUG 35 13 1,10 0,43

CUUGU 36 12 1,22 0,54

UGCUU 36 12 1,22 0,54

CUGCU 40 13 1,20 0,56

GUGCU 43 15 1,20 0,58

UGUGC 45 15 1,25 0,64

UGCUC 38 13 1,14 0,50

UGCUG 59 15 1,59 0,99

GCUGU 49 15 1,36 0,75

GCUGC 43 17 1,06 0,46

UGCGC 44 17 1,09 0,49

Hexanucleotide words

GUAACG 18 3 1,94 0,66

UGUAAU 23 2 2,56 1,08

GUAAGA 21 2 2,34 0,94

UGUAAG 43 2 3,06 1,74

UGUAAC 38 2 3,00 1,63

UUGUAA 32 2 2,93 1,47

UGUAAA 23 2 2,66 1,12

AUGUAA 28 2 2,88 1,34

CAUGUA 18 2 2,17 0,75

GUGUAA 35 2 2,82 1,49

CGUGUA 17 3 1,78 0,55

CUGUAA 30 2 2,73 1,35

CCUGUA 14 3 1,66 0,36

UUUGUA 16 2 2,07 0,63

UGUUUG 17 3 1,82 0,56

UGCGUG 18 4 1,52 0,44

GCGUGU 17 4 1,46 0,37

GCGUGC 18 5 1,39 0,36

CGUGCU 16 4 1,48 0,35

UCGUGC 16 4 1,48 0,35

CGUGUG 16 4 1,40 0,30

UGCUUG 17 3 1,68 0,50

UGCUUU 16 3 1,84 0,53

UGCUCU 18 3 1,83 0,61

UUGCUG 20 3 1,86 0,69

UGCUGU 26 3 2,14 0,99

GCUGUG 16 4 1,40 0,30

GCUGUU 19 3 1,80 0,63

UGCUGC 21 4 1,78 0,67

GUGCUG 17 4 1,46 0,37

UGUGCU 15 3 1,55 0,35

CUGCUG 17 4 1,55 0,42

GCUGCU 17 4 1,55 0,42

GAUGCA 15 3 1,75 0,45

UGCAUG 19 3 1,90 0,68

GCAUGU 18 3 1,84 0,61

CAUGUG 18 3 1,84 0,61

GCAUGC 16 3 1,58 0,41

CUGCGC 16 4 1,35 0,27

GUGCGC 16 5 1,26 0,22

UGCGCG 17 5 1,33 0,29

GCGCUG 16 5 1,26 0,22

GUGUGA 18 3 1,76 0,57

CUGUGC 17 4 1,55 0,42

CUCUGU 19 3 1,89 0,67

GCUCUG 17 4 1,55 0,42

UGUUGU 17 3 1,82 0,56

CCUUGC 14 3 1,43 0,24

UGCCUG 15 4 1,42 0,28

UUUCAU 16 2 2,16 0,66

*Prototheca wickerhamii*

N = 291

Base composition: A 20.3% C 31.2% G 28.5% U 20.0%

Pentanucleotide words

sequence observed expected log odds lower limit of 95% CI

AUGUA 53 6 2,35 1,49

GAUGU 38 9 1,50 0,77

CAUGU 42 9 1,62 0,89

UUGUA 43 6 2,10 1,23

GUGUA 56 9 2,07 1,33

GCUGU 70 13 1,89 1,28

CUGUA 92 9 2,63 1,93

**UGUAA** 166 6 4,12 3,29

GUAAC 105 10 2,81 2,12

UAACA 53 7 2,25 1,43

UGUGC 53 13 1,57 0,94

CUGUG 43 13 1,29 0,65

CUGUC 39 15 1,08 0,45

CCUGU 60 23 1,12 0,61

UCUGU 39 9 1,55 0,81

GCAUG 62 13 1,72 1,10

UGCAU 58 9 2,01 1,30

UGCAC 48 15 1,30 0,70

GUGCA 48 13 1,40 0,77

CUGCA 51 15 1,38 0,77

ACUGC 38 15 1,04 0,41

UCUGC 53 15 1,44 0,84

CUCUG 49 15 1,35 0,74

CUGCU 65 15 1,70 1,10

UGCUG 68 13 1,86 1,24

CAUGC 50 15 1,35 0,75

CCAUG 43 16 1,10 0,50

CCUGC 65 23 1,23 0,72

CCCUG 72 23 1,36 0,85

UCCCU 48 16 1,23 0,64

CCCUU 42 16 1,07 0,47

CCUUG 40 15 1,11 0,48

CUUGU 40 9 1,57 0,84

CUUGC 38 9 1,52 0,78

GCUUG 43 13 1,30 0,66

UGCCU 39 15 1,08 0,45

Hexanucleotide words

UAACAC 22 2 2,42 1,00

GUAACA 49 2 3,39 1,96

GUAACC 26 3 2,24 1,03

UGUAAC 95 2 4,27 2,84

CUGUAA 69 2 3,82 2,39

AUGUAA 35 1 3,41 1,66

GUGUAA 39 2 3,27 1,73

UUGUAA 27 1 3,22 1,37

UGUAAA 21 1 2,85 1,07

UGUAAG 36 2 3,18 1,63

GAUGUA 28 2 2,90 1,34

GCUGUA 39 3 2,79 1,55

UGCUGU 34 3 2,68 1,41

UCUGUA 22 2 2,56 1,04

CCUGUA 24 3 2,15 0,94

CCCUGU 30 5 1,97 0,97

UCCCUG 24 5 1,73 0,71

GCCUGU 22 5 1,63 0,61

CAUGCU 27 3 2,28 1,08

GCAUGC 21 4 1,67 0,61

UGCACU 22 3 2,15 0,88

GUGCAC 21 4 1,67 0,61

UGUGCA 29 3 2,45 1,20

UGUCUG 21 3 2,15 0,85

CUUCUG 21 3 2,05 0,81

GCUCUG 26 4 1,90 0,86

CUCUGU 27 3 2,33 1,10

UCUGCC 21 5 1,58 0,56

UGCCUG 24 5 1,73 0,71

UCCCUC 22 5 1,55 0,56

CUCCCU 27 5 1,77 0,80

CUCCCC 23 8 1,15 0,31

CCUCCC 22 8 1,10 0,26

CCCUCC 27 8 1,32 0,50

CCCCUC 27 8 1,32 0,50

CCCCUG 32 7 1,60 0,77

CCCUGC 30 7 1,53 0,69

CUGCCC 25 7 1,33 0,48

CCUGCA 24 5 1,73 0,71

CUGCAU 26 3 2,24 1,03

UGCAUG 31 3 2,53 1,28

CCUGCU 29 5 1,93 0,93

CUGCUC 25 5 1,77 0,76

CUGCUG 31 4 2,10 1,07

UGCUGC 21 4 1,67 0,61

GCUCCC 23 5 1,68 0,66

CACCCC 22 8 1,08 0,25

CCACCC 21 8 1,03 0,19

*Pyramimonas parkeae*

N =1260

Base composition: A 24.2% C 21.5% G 23.3% U 27.0%

Pentanucleotide words

sequence observed expected log odds lower limit of 95% CI

UUUUG 214 72 1,22 0,94

AUUUU 220 75 1,21 0,94

CAUUU 154 59 1,04 0,73

UGUAA 170 58 1,18 0,87

AUGUA 147 58 1,01 0,70

CAUGU 142 51 1,10 0,77

AACAA 122 43 1,12 0,76

CAAAA 126 43 1,15 0,80

AAAAA 138 48 1,13 0,79

AAAAU 152 54 1,13 0,80

Hexanucleotide words

AAAAAA 75 11 1,94 1,31

AAAAAU 66 13 1,69 1,09

AAUUUU 69 18 1,41 0,88

AUUUUU 77 20 1,41 0,91

UUUUUU 107 22 1,65 1,19

UUUUUG 102 19 1,75 1,26

UUUUGG 73 16 1,54 1,00

UUUGGG 68 14 1,62 1,04

UUUUGU 73 19 1,39 0,88

UUUGUU 63 19 1,24 0,72

UUGUAA 68 15 1,54 0,98

UUUUUC 71 17 1,44 0,92

UUUUCC 63 14 1,55 0,96

GUUUUU 82 19 1,52 1,01

UGUUUU 72 19 1,38 0,87

AUGUUU 69 17 1,44 0,91

*Scenedesmus obliquus*

N = 265

Base composition: A 20.1% C 23.8% G 31.2% U 24.9%

Pentanucleotide words

sequence observed expected log odds lower limit of 95% CI

AACUG 33 9 1,39 0,63

UAACU 47 7 2,03 1,23

ACAGU 39 9 1,58 0,84

AACAG 45 7 1,97 1,17

UAACA 100 6 3,25 2,41

GUAAC 182 9 4,12 3,41

**UGUAA** 176 10 3,97 3,28

GUGUA 93 15 2,22 1,63

UGUGU 61 18 1,39 0,84

AUGUA 33 10 1,34 0,60

CAUGU 34 11 1,20 0,50

CUGUA 34 11 1,20 0,50

ACUGU 33 11 1,16 0,46

AGCUG 44 14 1,26 0,64

CAGCU 45 11 1,57 0,88

CAGCA 44 9 1,77 1,02

GCAGC 66 14 1,82 1,21

AGCAG 42 11 1,43 0,75

UGCAG 45 14 1,29 0,66

CUGCA 33 11 1,20 0,49

GCAGU 39 14 1,12 0,48

CAGUG 42 14 1,21 0,58

UGCUU 35 14 1,01 0,36

UUGCU 35 14 1,01 0,36

GUGCU 43 18 1,01 0,42

GCUGC 65 17 1,57 1,00

UGCUG 78 18 1,77 1,22

CUGCU 53 13 1,55 0,92

UUGUU 42 15 1,17 0,55

UGUUG 56 18 1,28 0,72

UUGUA 52 12 1,66 1,00

CUUGU 50 14 1,43 0,81

CUGGU 46 18 1,09 0,51

UGGUA 39 15 1,07 0,45

GGUAA 48 12 1,55 0,89

UGGUG 55 23 1,02 0,49

Hexanucleotide words

AUGUAA 22 2 2,54 1,04

CUGUAA 29 2 2,68 1,30

UGUAAA 20 2 2,44 0,93

UUGUAA 42 2 3,06 1,73

UGUGUA 41 4 2,59 1,49

GUGUAA 86 3 3,77 2,58

UGUAAC 140 2 4,89 3,54

GUAACA 97 2 4,44 2,95

UAACAG 38 2 3,20 1,69

AACAGU 19 2 2,43 0,89

UAACAC 23 1 2,91 1,17

UAACAU 22 1 2,82 1,11

GUAACU 44 2 3,16 1,80

UAACUG 21 2 2,32 0,93

GGUAAC 38 3 2,76 1,53

GUAACC 22 2 2,42 1,00

GUAACG 21 3 2,09 0,83

UGGUAA 33 3 2,55 1,34

AGCAGC 22 3 2,19 0,91

CAGCAG 24 3 2,29 1,01

CUGCAG 20 3 1,87 0,69

UGCAGC 28 3 2,24 1,08

GCAGCA 34 3 2,68 1,42

GCAGCU 25 3 2,11 0,95

CAGCUG 31 3 2,35 1,20

AGCUGG 19 4 1,54 0,48

GCUGCA 21 3 1,92 0,75

CUUGUA 24 3 2,25 0,99

GCUUGU 26 4 1,89 0,86

UGCUUG 19 4 1,55 0,49

CUGCUU 19 3 1,83 0,64

GCUGCU 30 4 2,10 1,05

UGCUGU 21 4 1,66 0,60

UGUUGG 19 6 1,28 0,32

UGUUGU 19 4 1,51 0,46

UUGUUG 19 4 1,51 0,46

CUGUUG 18 4 1,49 0,42

GUGCUG 27 5 1,71 0,76

CUGCUG 31 4 2,14 1,09

UGCUGC 34 4 2,24 1,20

UGCUGG 25 5 1,62 0,67

GGCUGC 25 5 1,67 0,70

GCUGGC 20 5 1,42 0,44

GCUGGU 19 5 1,32 0,35

AGUGUA 20 3 2,00 0,75

CGUGUA 18 3 1,71 0,55

CUGGUG 27 5 1,71 0,76

UGGUGU 28 6 1,70 0,78

UGGUGC 19 5 1,32 0,35

GUGUGU 25 6 1,58 0,64

*Scherffelia dubia*

N = 110

Base composition: A 25.7%, C 26.0%, G 26.8%, U 21.5%

Pentanucleotide words

sequence observed expected log odds lower limit of 95% CI

GUAAU 15 4 1.43 ^ 0.29

GUAAC 19 5 1.48 0.45

GUAAA 31 5 2.11 1.12

**UGUAA** 68 4 3.76 2.69

UGUAU 13 4 1.27 0.11

UUGUA 21 3 2.13 0.89

CUGUA 26 4 2.10 1.01

GUGUA 24 4 2.00 0.90

AUGUA 23 4 1.95 0.85

AAUGU 12 4 1.18 0.01

UAAUG 12 4 1.18 0.01

UUUUA 15 3 1.73 0.46

AUUUU 14 3 1.65 0.37

CUUUU 14 3 1.65 0.37

UUUUG 13 3 1.56 0.28

UUUGU 14 3 1.65 0.37

AUUGU 13 3 1.56 0.28

GCUGC 16 5 1.27 0.23

GCUGU 16 4 1.51 0.38

UGUGU 16 4 1.51 0.38

AGUGU 14 4 1.35 0.21

AUUAU 12 3 1.47 0.18

AAUAA 16 5 1.27 0.23

UAAAA 17 5 1.35 0.31

UAAAU 13 4 1.27 0.11

AUAAA 14 5 1.12 0.06

Hexanucleotide words

UGUAAG 8 1 2.03754 0.03652

GUAACA 8 1 1.90831 0.01357

UGUAAC 18 1 2.99864 1.02235

UGUAAU 15 1 2.93977 0.80679

GUAAAA 9 1 2.03595 0.15524

UGUAAA 30 1 3.64923 1.69305

UUGUAA 18 1 3.15418 1.03092

AUUGUA 8 1 2.24007 0.05583

AUGUAA 18 1 2.99864 1.02235

AAUGUA 9 1 2.21216 0.18351

CUGUAA 18 1 2.99864 1.02235

ACUGUA 10 1 2.32747 0.30927

CCUGUA 9 1 2.16517 0.17743

GUGUAA 17 1 2.88368 0.94628

UGUGUA 9 1 2.36770 0.19562

CUGUGU 8 1 2.24007 0.05583

UAAAAA 10 1 2.19254 0.28958

**AAUAAA** 11 1 2.29790 0.40404

GUAAAU 9 1 2.21216 0.18351

GCUGCC 9 1 1.95805 0.13813

*Ulva linza*

N = 54

Base composition: A 22.3% C 22.3% G 28.6% U 26.8%

Pentanucleotide words

sequence observed expected log odds lower limit of 95% CI

GUAAU 11 3 1,65 0,22

UAACA 10 2 1,98 0,28

GUAAC 17 2 2,42 0,93

GUAAA 9 2 1,59 0,04

**UGUAA** 36 3 3,70 2,32

UUGUA 16 3 1,95 0,66

GUGUA 11 3 1,38 0,08

CUGUA 11 3 1,65 0,22

AUGUA 10 3 1,53 0,09

AUGUG 11 3 1,38 0,08

UGUGU 11 3 1,33 0,05

CAUGU 10 3 1,53 0,09

GCAUG 10 3 1,46 0,06

AACAA 8 1 1,90 0,04

CAACA 8 1 1,90 0,04

UGCAA 9 2 1,59 0,04

CAGCA 10 2 1,91 0,26

CCAGU 9 2 1,60 0,05

ACCAG 9 2 1,78 0,12

Hexanucleotide words

AUGUAA 8 1 2,81 0,07

CUGUAA 9 1 2,95 0,22

UUGUAA 13 1 3,25 0,74

UGUAAC 13 1 3,41 0,70

UGUAAU 10 1 2,92 0,39

GUAACA 9 1 2,36 0,26

**Streptophyta**

*Closterium peracerosum*

N = 136

Base composition: A 3.6% C 27.6% G 33.3% U 35.5%

Pentanucleotide words

sequence observed expected log odds lower limit of 95% CI

UGCAA 19 1 3,09 1,06

UUCAA 18 1 3,02 1,00

UUGAA 24 1 3,36 1,35

UGAAU 19 1 3,09 1,06

UAUAA 18 1 3,02 1,00

AUAAU 18 1 3,02 1,00

UAAUU 20 1 3,15 1,12

AAUUU 20 1 3,15 1,12

AAUUG 24 1 3,36 1,35

AUUGA 21 1 3,20 1,18

AAAUU 18 1 3,02 1,00

UAAAU 18 1 3,02 1,00

AAAUG 23 1 3,31 1,30

AAUGU 20 1 3,15 1,12

UAAUG 20 1 3,15 1,12

GUAAU 20 1 3,15 1,12

UGUAA 25 1 3,41 1,40

UUAUU 18 4 1,73 0,57

UUUAU 19 4 1,79 0,64

AUUUU 30 4 2,35 1,22

CAUUU 24 3 2,33 1,06

GAUUU 19 3 1,86 0,67

UGAUU 18 3 1,80 0,61

AUUUG 22 3 2,03 0,86

AUUUC 22 3 2,22 0,95

UUUCA 20 3 2,11 0,83

UAUUU 24 4 2,07 0,93

AUAUU 20 1 3,15 1,12

UAUAU 18 1 3,02 1,00

AUUGU 28 3 2,33 1,17

UUGUA 28 3 2,33 1,17

CUGUA 22 3 2,29 0,98

AUGUA 21 1 3,20 1,18

UGUAC 22 3 2,29 0,98

UGUAU 31 3 2,46 1,30

Hexanucleotide words

UUAUAU 10 1 2,37 0,31

UGUAUA 10 1 2,37 0,31

UAUAAU 13 1 2,66 0,62

AAUUGU 8 1 2,13 0,05

AUUGUA 17 1 2,96 0,94

UUGUAA 12 1 2,57 0,53

UUGUAU 10 1 2,22 0,30

UAUUGU 9 1 2,10 0,17

AAUUGA 8 1 2,13 0,05

UAAUUG 9 1 2,26 0,19

UGUAAU 9 1 2,26 0,19

AAUAAA 8 1 2,13 0,05

AAAUGC 8 1 2,13 0,05

UAAAUG 12 1 2,57 0,53

UGAAAA 9 1 2,26 0,19

UUGAAA 11 1 2,47 0,43

AUUGAA 9 1 2,26 0,19

UUGAAU 9 1 2,26 0,19

AAUGAA 8 1 2,13 0,05

AAUGCA 9 1 2,26 0,19

UGCAUU 10 1 2,47 0,32

AUUUUG 11 1 2,32 0,41

AUUUGU 12 1 2,42 0,51

CAUUUG 9 1 2,36 0,20

CAUUUU 10 1 2,41 0,32

ACAUUU 9 1 2,26 0,19

AACAUU 8 1 2,13 0,05

AAAUUU 8 1 2,13 0,05

AUUUCA 9 1 2,26 0,19

UUCAAU 11 1 2,47 0,43

UGUACA 11 1 2,47 0,43

UGUGUA 11 1 2,39 0,42

UGUAUG 10 1 2,28 0,31

CUGUAU 9 1 2,36 0,20

AUGUAC 9 1 2,26 0,19

AAUGUA 10 1 2,37 0,31

UAUGUA 9 1 2,26 0,19

UGUGCA 9 1 2,42 0,20

UGAGAA 9 1 2,26 0,19

*Coleochaete scutata*

N = 142

Base composition: A 27.2% C 18,9% G 22,6% U 31,3%

Pentanucleotide words

sequence observed expected log odds lower limit of 95% CI

UUGUU 36 14 1,12 0,45

UGUUU 42 14 1,33 0,68

GUUUU 39 14 1,23 0,57

AUUUU 40 17 1,06 0,43

UUUUU 47 20 1,13 0,54

UUUUG 45 14 1,43 0,78

UUUGG 26 10 1,06 0,29

UUUUC 34 12 1,24 0,53

UUUCC 22 7 1,24 0,36

AUGCA 17 6 1,05 0,11

UGCAA 21 6 1,29 0,38

UCAAA 23 8 1,20 0,36

CAAAU 24 8 1,26 0,41

AAAUG 23 9 1,01 0,21

AAUGC 18 6 1,11 0,18

AACUG 17 6 1,05 0,11

GCUUC 17 5 1,28 0,27

CUUUG 22 9 1,05 0,22

CCUUU 20 7 1,13 0,24

Hexanucleotide words

UUUCCA 9 2 1,61 0,02

UUUCAA 11 3 1,45 0,11

UUUUCA 11 3 1,31 0,03

UUUUCC 14 2 1,94 0,50

UUUUUC 21 4 1,89 0,75

UUUUGG 16 3 1,73 0,49

UUUUUG 20 4 1,65 0,58

UUUUUU 30 6 1,80 0,89

GUUUUU 20 4 1,65 0,58

AUUUUU 21 5 1,51 0,53

AAUUUU 15 5 1,27 0,20

AAAUUU 14 4 1,34 0,20

CAAAUU 11 2 1,59 0,17

UCAAAU 12 2 1,69 0,28

CAUUUU 13 3 1,49 0,24

AUUUUG 13 4 1,31 0,14

UUUUGU 17 4 1,46 0,38

UUUGUU 15 4 1,32 0,23

UUGUUU 22 4 1,76 0,70

UGUUUU 25 4 1,91 0,87

GUUUUG 11 3 1,31 0,04

UGUUUC 10 3 1,39 0,01

AUGUUU 13 4 1,31 0,14

CAUGUU 11 2 1,64 0,19

CAAUUG 10 2 1,68 0,14

AAUUGU 11 3 1,27 0,01

AACUGU 9 2 1,57 0,01

AAUGCA 9 2 1,71 0,06

*Klebsormidium subtile*

N = 473

Base composition: A 27.8% C 20.5% G 25.6% U 26.1%

Pentanucleotide words

sequence observed expected log odds lower limit of 95% CI

CCCCC 48 8 1,90 1,13

CCCUU 63 24 1,07 0,58

Hexanucleotide words

UUUUCC 46 14 1,25 0,64

UUUCCC 40 8 1,66 0,89

UUCCCC 32 5 1,97 1,00

CCCCCC 28 2 2,93 1,32

GCCCCC 24 3 2,28 0,99

GGCCCC 24 4 1,79 0,75

GGGCCC 26 7 1,39 0,53

CCCCCU 26 3 2,30 1,05

CCCUUU 39 8 1,63 0,86

CCUUUU 41 14 1,12 0,50

UCCCUU 25 8 1,15 0,35

*Mesostigma viride*

N = 1327

Base composition: A 26.9% C 20.6% G 23.1% U 29.4%

Pentanucleotide words

sequence observed expected log odds lower limit of 95% CI

AAAAU 236 94 1,04 0,79

AAUAA 386 94 1,68 1,44

AUAAA 371 94 1,63 1,39

UAAAA 256 94 1,14 0,89

AAUUU 349 112 1,35 1,12

UAAUU 374 112 1,45 1,22

UUAAU 359 112 1,39 1,16

UUAAA 263 103 1,08 0,84

AUUAA 322 103 1,34 1,10

AAUUA 321 103 1,34 1,10

AAAUU 309 103 1,29 1,05

UAAAU 311 103 1,29 1,06

AUAAU 250 103 1,02 0,77

AUUUU 350 123 1,26 1,04

UUUAA 291 112 1,11 0,88

AUUUA 291 112 1,11 0,88

UAUUU 306 123 1,08 0,85

Hexanucleotide words

UAAUUA 67 52 1,27 0,95

AAUUAA 208 47 1,62 1,29

AUUAAU 171 52 1,30 0,98

UUAAUU 200 56 1,39 1,08

UAAUAA 153 47 1,26 0,93

CAAUAA 97 33 1,13 0,72

AUUAAA 128 47 1,06 0,72

UUAAAU 137 52 1,05 0,72

UAAAUU 140 52 1,07 0,74

AAAUUA 124 47 1,03 0,68

AAAAAA 103 40 1,01 0,63

UAAAAA 112 43 1,01 0,65

AUAAAA 114 43 1,03 0,67

AUAAAC 89 33 1,03 0,63

**AAUAAA** 254 43 1,95 1,62

AAAUAA 114 43 1,03 0,67

UUUAAU 157 56 1,11 0,79

AAAUUU 132 52 1,00 0,67

AAUUUU 145 56 1,02 0,70
