# Supplementary material for: Gain and loss of polyadenylation signals during evolution of green algae
Source: BMC Evol Biol. 2007 Apr 18;7:65. doi: 10.1186/1471-2148-7-65 (PMC1868727; doi:10.1186/1471-2148-7-65)
Supplement: Additional file 3 — Figure S2: Distribution of various hexanucleotide words within 50 nt upstream from the CS in different chlorophyte and streptophyte algae. Chlorophyte sequence motifs are depicted on the left, streptophyte sequence motifs on the right. [file 1471-2148-7-65-S3.pdf]

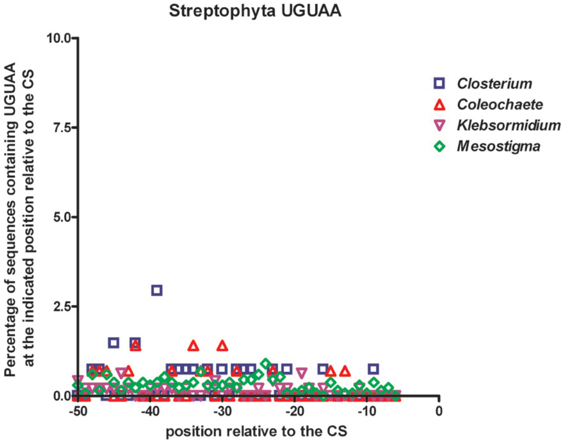

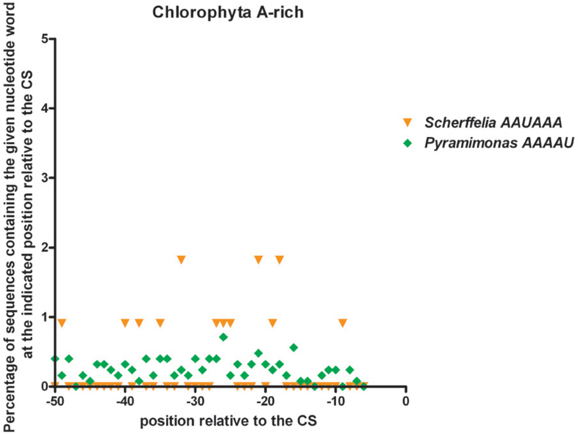

Figure S2: Distribution of various nucleotide words within 50 nt upstream from the cleavage site in chlorophyte and streptophyte algae.
