# Supplementary material for: Gain and loss of polyadenylation signals during evolution of green algae
Source: BMC Evol Biol. 2007 Apr 18;7:65. doi: 10.1186/1471-2148-7-65 (PMC1868727; doi:10.1186/1471-2148-7-65)
Supplement: Additional file 4 — Data set S1: Sequences (200 nt upstream of the CS, in fasta format) of the non-redundant Pyramimonas, Klebsormidium and Coleochaete data sets. [file 1471-2148-7-65-S4.doc]

Supplemental data set S1

Sequences (200 nt upstream of the CS, in fasta format) of the non-redundant

Pyramimonas, Klebsormidium and Coleochaete data sets.

***Pyramimonas parkeae***

>1

GCGGATTGGCATCTCTCAATCGTGCTTCGAACCACAAAATAGAAAGGGTGGTAAACATTC

TCAAATAATCGCTGATGAAAACAATCGGAATCACGCATAATCGTGTGCACACATGTTTTA

GGTCGTTGTGTTAGAACGCAAGTAGCGTTCTAGATGAGGGTTATTCCTAATCTAGAAGGC

AAGCTGTATTGTCAACGTTTAAAAAAAAAAA

>2

TTACCCGGCTAACCTGCCTTTTTGATGACCGCAGGCGATCAAGTTCATTCAGCAGGCTGT

AGGACTGCGTGGCTATGCCCAGAAGGACCCACTGATTGAGTACAAACTCGAGGGTTACAA

TCTCTTCCGCGAGATGATGGCGCAGATTCGACGCAACGTGATATACTCGGTGTACCAGTT

CACGCCGAAGCGCTTGGATGAAAAAAAAAAA

>3

TTTCCGTGTGGACACAAGTGTGTGTGCGAGTCTGATGCTGACACGCTCATGGCTGGTGAT

AAGGCGTGCCCATTGTGCCGCACGCAAGCCACCAACATCACCCGTATATCTTAGGGTGTC

AAGTAGGCTCACATCGGCTCACTCTTTGTGTGTTCATACTATTTCTAAGTAAGTGTTTTC

TAATTTTGTAGCCGTTGTTGAAAAAAAAAAA

>4

TGGCGCTCCTAAGATGGGCTGAACATTGTTACGATACACATCTTCATCTTTTCATGTTAG

AAGCAGTTCCCTCCCTCATCGTGTGGACATGATCGAATGTTAGCTTTGCATTGGAAGAGT

ATCAGTACATGATCTATACAGTTCAAGGTCCTAACGCTTTTGACTTGTATATGATCACTA

AACAAAAGTATATGATCCCTAAAAAAAAAAA

>5

CGAGGAGGAGGACGAAGAAGAGGATGAAGAAAAGGAGCTTTAAGTTATATGTATTTTTCA

GGACAATTGTGCCCTACAAGAGAGACACCTTGCAGGATAAGTAAGCAAATCATGGAACTG

GGGAAACCAATGGTGCTTTTTGGACCTGTGTGCAGACACGAACCGAATGATCATTATTTG

TGATGAAAATTACCACACCCAAAAAAAAAAA

>6

CCCAGGGCCGTAATAGCACTCTTCAAGCATGCACATGATTACGAATATGCTGCATAATAG

TCTTAGGGATCATTTGCGTTGATTTAGATTACACATCCCAAGCCCAGTACCAAAGGACTC

CGTTCAGAAACAATCCGGAACGCTTATCCGTATACACAAAACAACGATTGATTCATGTTG

CAAACGATGGTAGATTTCATAAAAAAAAAAA

>7

GTTATTTGCATGTGTTTGTCAAAGGCATATGCTGCCTGCGAAACGAGATCCCTTGCAAGA

GTGTGATCTTCGTGTGCATTGCTAGGGTCTTTCTGCATGCTTTCATGCAGTGCAAGCTAA

ACCTGATAAGTTGGTTGACATCTGGACCAACTGTTAACGATTAACAGTAAACACACAACT

GAGCAAATTTGCTCGCCCGTAAAAAAAAAAA

>8

TCCGCGAACTGGTATAATCACTCCAAGGAAATGTGCGCAAAGCTTTCCACATACGCGCTA

CATGTGTTGCTGAGTTTGGAAAGTTGCGAGATTTAGAAACTTTGACTTTTGGTTGTGTCA

TCAGAATCGGCTTGCTGGGTCGACAATAAAAAGATAAGGATACTTTATGTAACTTTTGTA

ATGTGATTAAATTCGAAGCCAAAAAAAAAAA

>9

ATCATCGTGAATATGCATCGATATACCTACAGGTAGGGGCAGCGCCCGTGGCTGGGGAGG

ATAGTTCTCCCCGAATCAATCCCCTGCATTGCGGGGGGCTCACGTTAATTATTATAATGC

ATCGATGAACGCAAAATATTTGACAAATCGTACTGCACTGTATGGTATCTGCTGGTGTCA

AAGAATTTAGCTCGAATCCCAAAAAAAAAAA

>10

CTGATTGACGTGAGCTGACAAAAAGACGTCGTTTACAGTTCCTGCGAGGTTGGAGTTTGT

TGGAGAGGATGCTTCTGATGCGACACCTTCAAACTGCAATATCGGGAAACCTGATGTGAT

ACCCAAACACTGCATAATAGGTGCTATGCATGATGACAATGTATATTTATTTGTGTACAT

CTACTTGCATAACTACTCTCAAAAAAAAAAA

>11

ATGCCACATTTTGGCGCTATCATAAGCTGGCCGACCAATAGCAGCGCACTTCATTACTTA

CATTTGTCCGGTACAATATCTAGATGTGAAAGATCGATTCACATATCAAAAGTCCTAAAA

AAAAAAAATACCCTGAAAAAAAAAAAAAAAAAAAAATCTTTGTCGCAGCATTCACAGGTA

TGGGATCGTTCTAGTTCTCTAAAAAAAAAAA

>12

GATATAGTTTCTGTGCGTTAAGGTGAGCTTAAAATGTACTCAGCGTGTAGCTTTGGCAGC

ATCAATCCCAGAAGTTCCAATTCTCCTTCAAGGCATCAGCAGGGTCAAGAGACTAGCCTC

TAGATTGAGACGGGTGCTCTTGCTTGGTAAAGATTCATTTGGTGTTGTAAATGTTGTGAT

CGTATGCTGTAATTGACAGTAAAAAAAAAAA

>13

TAAATGACAAACGAGTGGAGAAGAAGTACTGCAATATCCAGGTCACTCGCTATCCGGATC

TGACGTCAGTTTTGGCAAACAAGACTTAACATGGAATCCTAACGAGCCAAATGCATAGGT

TTGCACGTCGCTGCTCGTTAAGGTCTGATTTCGTCTACAATGTCAGCCTTGTCGTTCATA

TAATGCAATCAAAAGGATATAAAAAAAAAAA

>14

ACATAGCGTTGGCCTGGTGTGTTGGGCATATCTGGCTCCTCATCTTAGCCGCAGCAAAAT

TACACACATATTCTGTTCAGTTCATGCCTCTAAAATGACATCACGAGGAAACTGCGCTGT

AAACAAGTGCAATAAAACAGATTTAGGCGAGTGTCTTCCGCGGTGTTGTGTAGTGAATTT

CGATTAAATGGTGCACCACCAAAAAAAAAAA

>15

GGATTTTTTGGATTGGGCAGTGTTTCCGATTTTTTGGTGCCCCATATCCATTTTTTTCCC

CTTGGGGTAAAAGTCCCCCCCTGGGGGGTGGGGGTCCATTCAGATTCAGTTACCCATTTT

GGGGGGGGGGGTCCCCCCGGGCCGGGGTAAAAATGTAATGGGTGGAAAGGTGTTTACGGG

TTTTTTCCATCCCGGTAACCAAAAAAAAAAA

>16

TCCCATTTCTTGAGGGTCCAGGTGTTCTCCATCAGCTCCCGAAAGTACTCGTTGCTGAAC

GTCGTCTCCGCGCGCGTCCACGGGCCCCAGTATCCAGACCGATCCTATCGGCACCGGCCA

AGAGCGTGCGCGCCGCACAGCGCCACAATCTCCGCGTCGTTCATGCCCTGCAACACCCGA

CAACAGTAAAGCTCGCAGGCAAAAAAAAAAA

>17

AGCCGATGAGGACGTGGACGCGATCACTGCCGTCATTGAGGCAGACAAGGCTGCTGCTGA

TGCTGCCAAGCTTCCCAAGGGCCCAACACCCGCCGCTTAAGCATCTCACAAGAGAACTAG

GGAGTGAGTTTTGAAACATGAAAGGATCTTGGATGGAAGTAACAAATCGTATCAATTGCA

AGGAAATACATTTGTGCTTGAAAAAAAAAAA

>18

GATGGTCCAACGATTTGTTTGCATTTGAAGTGACAAGAAATTTAGCAATGATCGTCCCGT

CACTACGTGTGGAACCGCTATTGCAGAATCAAATGCAGAATTGACGGCATTTAGATATGC

GAATATTATGTCGGGATATGAATATTGTTCGCATAGACATAGAAGAATGTCAGCGTCTCT

TATGACGAATGATATTGTTTAAAAAAAAAAA

>19

TGGAGGCAGACGAAAAGTACACGACTCCCTAGCAGGGTATGATATTGCAGTTCTGAAAAG

AGGATAAACGGCAAGTGCAGCTCAAATTCATTGTAGTTGTAATTATCAGTGAAGGACTTC

TGAAGAGGCCTTCCTTCATGTCAACTGTCAAGCTGTTGCTTCTGGTGTGAGATTTTTCCC

CTTGTGGAATATGTGAAGCCAAAAAAAAAAA

>20

AAGAGACTCTCAAATGATCCAACTGCAGGGTGTAGGCGCGATATCGGCGATGCTAAGCAT

CTCATTGTGCACACTAAAACGCAATTGGCTAGATCTTTAAACAAGTCTCATTGCGATGTG

TAGCAGATGATGAAAAAACAAATGCCAGTATTGCGTGGTAACTGTGTTTATAATTTGCAG

TGGCTATTCAAATTAGATGCAAAAAAAAAAA

>21

TTATGGAAGTCGAGTGTGAAGTGCTCGCAAGGACGTCCGTTTGCTCATGTCAGACAAGCG

GCAGGGAATAGCCCAAACCCATTTGCAACATCAGATTACTTGCAAGCGCTGATACACAGA

TGACATGGAGCTTGATCTTGGGTTGAATCAAGCCAATTGTAAGAAATCCTTACATTTAAT

CGAGTTATCATGTTTGAATCAAAAAAAAAAA

>22

GTGAACCAGGCTCCATCAACCACACCCACAGCTGCATCAGTTGTGGAGAAGCGCGCGTTC

GTGCGAGGATTCGCCGCCAGCCGCTTGTAGGTCCATAGATGAACCTATGCGGATGTAGTT

TTCAGGATTGTCTTGCCGGGGTAGCTACATCTGTTTTCCTGTAATTTTATTATTGTGTGT

ATAACATCATTGATTCAGTGAAAAAAAAAAA

>23

TACAAGAAAGAATGAAAACGTTATTTTTTTGCAGAGTGGTGGACAGAATGGCAATGTGAT

ATCGACCAATGGTGCTGCTTGTCGGTGGTAGCACCAATGAATGACCTTTATAGCATTGTC

AGCTGTGTAGGTGAGTTTGTGCTTCTCAGGCTGATCTGTATATTTGCTTCCTTTGTTCCT

AAGTATCTCTGCATATGCGTAAAAAAAAAAA

>24

TCTCTCCTCTGAGGGATGCCCACAGGCTGCCTGTGGCCTCTCCCTGTGTATAGAGAATTT

CAAAGGACAGCCTGAATAGGAATTAACCTGTGGGCTTTCAAAGCCAAGCTCGAAGATTTT

GAACAATGGAAGGCGAAGTGAGAATAAGCCCTCCTGTTCCACCCCTAGAAATTTTCGTGC

AAGATGCATTGTGTTGTTGTAAAAAAAAAAA

>25

CTATCATTGTTGCAGCGCAGTAGATATTGGATACTTGCGCTTGTATTCAGATAGGACAGG

GGATCTCTCGCGCATAGGGTAGTTTACGTGCGATACACATTGGGTTCTCATGAAGGAGAC

CTCATTGTCTGATCCTTGAATGATTATTTGTTTTGGTACTGTTGGAGTTTTCGATTTGTA

GTCATTTGGTTATGGTCTTCAAAAAAAAAAA

>26

TTTGTTTTTTAGTTGTGCTCTTGGCACATCATAGAGCAGAAGTGATGCTGAGCTTGAAAA

AACAATGTGCTTTGGATGGTTGTCTCTCTGCTTGAGTGTCCGAGTGGAATTTCGGTGTAC

TTATGTAGCCATTCTGCAGCGTATTCTGGGAAGATATTCATGATATTATTTAATTTGTAC

ATACCAATACTGCATTAGTCAAAAAAAAAAA

>27

TTGGAACACTCAATAGAAATGAAATGCAGCATCCGCACACATGTAGGAACGAGCGGAATG

ATGGCGACATGTGAACGTTTGGTGCATGTGTACATATTGACAGTTGTTGTGCAGAAGTCC

CATATAGAGTATGACTGAAAAAAAAGAAAAATCTCGTGCAAGCTTGCATGTATTTTTCAA

TTGAACTGTGCTGATAAATCAAAAAAAAAAA

>28

TCAACCGTCATGCCAACTGCAGTCAAGGAAATGTGTACCAATGGCATCATGATATCAAGG

CGCAGACCCACTGTAACGAAGCAACTCTGCAATCACCAAGATGGCTTGCTTGCCACCAGA

CAGATGAGGCTCATCTTTAAATGCACGTCACATGAACTTATAACATGAAATTGATAAAAA

TTATCATTTATTTGCGCTGTAAAAAAAAAAA

>29

GACGCCACTCTTGCAGAGGCAATAAAACCAAAAGTGCAAGCATGCGGATTGTGTTTGGGT

TGCATATTGGATAGGGTGTCCCTTAATATTGCTGCACAGGGTGATTGTGTTGCCATGAAA

ACAGATATGCATGAACCCCTTGAGACATGTGTCATATATGTCAAGTAGTACAGGTGGGCC

CAAAAAAAAAACCTGATTGCAAAAAAAAAAA

>30

GAATAATTTTTTGTAGTTAAATTTTGCCCTATGCCCTTAGTAAGTGAAAAGTGCTGTGAT

TTTTGGATATTAAACCCCCCAAAGGGATCCCCAAATTTTTTTGGGGACCCAATGTTTTAA

ATTTTGAACCCCAAAAAAAAAAA

>31

GTACCTGCTCTCGGTGTACCGGATATATATGTTCGGAAGGTTTTATATAAGTGTCCATAC

ACTGTCATTTAGGCAAAATGGTGACAGTTTGCACTCAGCACACACCTTGTAAGTAATATT

GGCACTCAAACGAACCTGAAAAGAAAAAAAAAAAAAAAAAAAAAATACTAGTCGCAGCAT

TCACAGTTGATATTCCCATGAAAAAAAAAAA

>32

GCGAAGGGCATACGCTCCGCTGCGTTATCCAAGCTCATGATCAATGAAGGAGTTCGACCT

GAATGCATGTTGTTCTGCGATTTCTATGGCAAGCCCAATGTATAGTATGTGATTATTACG

CACACAAGTCTGTATTTGTGTGAAACGTGACAACAATTCCAGTAAAGTGTTGTTATTTAT

GTTAACCGTCAAATTGTATCAAAAAAAAAAA

>33

TCCTCGTACTGCTCCTCCTGGGTTTTGGGCTTGGCCCCAAAAATGTTGCCGAACAAGCCA

GCTTTGGGAGCAACCTCGTCCTCTTCCTCCTCCTCGTCTTCTTCTTCGTACTCTTCTTCG

TACTCTTGCTCCACGTCAACTGGGCGCGGGGCAAACAGGCCGCCAAAAAAAAAAAAAAAC

AGGGAAGCAGGGCAGCATAGAAAAAAAAAAA

>34

TAGTTTGTCAACTGTAACAGAGCGACATGGTAGGAAATGTATATATGACCTCCCGAAAAA

GACTTCTTATACCCCATGTCATGAAGAAAAAAGAAATTCCTTGTGTACACAGAACATAAA

CAGACAATTGAAGCTTTTCCGCACAATCAATATTTCGGGATGTAAGATGTGATCAAAAGA

AATGTACATTGTTTAGCCCGAAAAAAAAAAA

>35

TTGCACTACTGCTGCTCCTAAGGAGCTTGGTACACTTCGGGAAGCCGATGGTCAGGAGAA

GTTGTAGAGACTGCGTGGCCACATCGTGCTCCTTCAAGTAACACAACACAAAACGATGGA

ACTTTTACCCGCTGGCTCTTTCCGGCGTGTGTTTTGCTGCAGCGTAAAGTGTAAATGTGA

TCTCCGCCTGCTTCTATGTCAAAAAAAAAAA

>36

GGGCTCAGTTTTAGATGGCACACCCTCTTTCCTTGATTTCAAGGTCTTGGTCGTAGACAA

CACGACCTGTGTTTCAGAGCAGGCTAGGTGACTTGGATACCTGCTGCTGCGCCAGGCAGC

TGGTGTCAAGTGCTCAAAGACTTCCCCTGCCGTGTACGTTGTTTTCAAACATGTATTCAA

TTTTTAAGGTTGGGTGATGTAAAAAAAAAAA

>37

TACATGGATCAATGTGTTACCGCTATGTGGGGAAAGCTACTAGTCGGTCAATATGAGGTT

ATGAATCAATGAATTGCTAAGCAGTCGCAAAAAAAAAAAAAAAAAAATCGTAGTCGCAGC

ATTCACAGGCATATAGTCTCCATCATGATAGCCGCAGAGCATTCTGACAATCAATTTTAT

GTAGGCACACCCATGAATTCAAAAAAAAAAA

>38

TCCTTACATGGGGAATATCCCTCTTGTGGTTACCAAATTGCGCTTGAGTATGTTTGCCTG

CGGATGTATGATGAGACTTCTGAGATCCATGACATCACTCAGATTCCAAGAATCCCTTAT

GAAAACACGTTTTTATTGTGACTCTACCAAACGATGGAATTGAAGTGTATCTGCATTATG

ATCTTCACATATTACCTTCCAAAAAAAAAAA

>39

ATGTCTGACATTCATAAATGCATGAATACCAAACACAGGCAGACACGACCACACCGAGCA

GCGCATCTGCACTTGTGAACTTCTACTGGGGGTGTACCAGCGAAAGGTACACTGGATCCA

TGAACTATGCATACAAAACATTCAAATCTGCTGGTTGTGACAAAGCCTCCCGTTCAAGCT

GAAAAAAAGCAATACCAATCAAAAAAAAAAA

>40

GACACCTCCTTCTCAGTGGTTTTCTCGGTCCAGAGGGAGATGGGATAGGAAATAAAAAAA

AAAAAAAAAAAAAAATAGGTCCTGCAGCATTCACAGCTCCAGATACTCCAGTTGGTCCTC

CTTCAAATGCAGCACGATCTTGGTTCCACGCCCGATCTGCTCACTTGTGTCCTTCGCCCC

CGTAAAAAAGGAGGAGGAGGAAAAAAAAAAA

>41

TCTGTTGCTGAACATGGCATGCGTAAGCATCCAAGTGACCTCTAGGGAGGCAACTGCAAT

GTAGGCAAGCAGATGGTGAAATATGTACTAGTGTGTACTCCTTCAGAACACGCCCATGGT

GCTTGCTCATCAGATTATCAATGGAGTGGTAGTTTATCGCACAGATTATTGGTGTGATTC

TACGTAATTGTGTACAAGCCAAAAAAAAAAA

>42

TAGCTCTCAAGGTCGCTGACATTCAGCATTTGGGCATAGCTGTCATTATCGTTTGTAAAA

CCAAGTGAGCAAACAGCATGAACAATTGATTCCTACCCTCAGCCCCAAGCGCCATTGATG

GAAATTATGTAAACTGTTTTCGTTTCAAAAAAAAAAAAAAATCAGAAGGTACATTTCAGT

GTTTCATTGTTGTAACTCCTAAAAAAAAAAA

>43

ACGTGACGATCAAACCCGAGTGGACAAACTGTGAGGTTTAGCAACAGTCATTCACATGGA

TGGTGTGAGCTGAACGCTTGGTTAGGAGAAATTGAAGTAGTCCCAAGACACACTAGTCTA

ATGACTGTATTTAACATTTTGGATGAAGTGGAATATATCATTTCATATAAGGTGAAAAAA

GCATAAACGATGTAATACTGAAAAAAAAAAA

>44

GCTCGCTGGTGATTTAGGATTGGCCACGCTGACTGAAGGATCACCTTCGGTCACCTAACG

AGGATACCAAGCGCATGGACAGCCACCATGGTATCTGACTGGAAGAGACTATTCTTTGTT

GTGGAACATGATGTCTCCATGTGTGTGTTGATTTCATATGCACAGCACCGTATTCAAACG

GTACTTGCAACTCAACGTTTAAAAAAAAAAA

>45

GGGAATTTTGGGGGGATAACATTTTCCCCCCGGAACCCCTTTTTCCCCTGTTTTCCCCCC

CCTTTTTTGGGGCCCCTTTTAAATTACCCAACTTTCCCTCCCCCCCGGTGGGGGGTTTCC

CCTTTTGGTCCCCCCCCGGGGGCCCCGATTTTCCTTGGGTTTTTTTTCCCCGCCTTTCTT

TTCTGGGGGATGGGGGGGGTAAAAAAAAAAA

>46

CTGTGTTATGATTCAAAGTCGACAGTAGGATTAAAGTGATCAAGTTGCAAATGTTCTAGC

ATATTGTCACAATAGTGGTCATTATCTAATATGCAGCCTTCGGTAGTAATCGAGGATATG

ACAAAACCATCGCAATAGTAATCATTAATCTGTGACGTTTACCGATCTCCCTAAAAAAAA

AACAGTGAATAGACCGTCACAAAAAAAAAAA

>47

TCCTTTGGTGATCACCGATGCCCGCGAGTTCACAAGAAGAAACATACAGCTCATGATGTA

AGAAATTACTTTCATTTGTTGATGACAGTTGCAACCCATACATCAAAATTCAGACTGCAG

CTGTCCAGCCGTTTCAAAAACTACATTTCCATGTAAGTTCACTCATCTCGCCCCCGGCTT

ATTCCGGAGCACTGCAATCCAAAAAAAAAAA

>48

ACATACAGAGACCACCATTGAAACGGAGAATGCTGGATCTGCGCGCACCAATAGCTGCTT

GTTGTCTTCCAAATAAGATTGCCAAGGCGGCTAGCTCGTTGTTGAAAATGCTACACATGA

ACTCACTTTGTTGTTTGTGCACACGAGTTGCACTCACATACTTGCAAGGGTAATAAGTGT

ACATGCGATATCCAATTTGTAAAAAAAAAAA

>49

TGATATGATCGCCTTCAAACGTTTGAGGGAGAGCCAGTGGAGGATGGATGGACCAGGCTG

CGAACCAATCTGCCAATGCTATCTTGCTTGTGTTTTTTGTGAAAAACACAATTCTATGTA

TCGCGTTCCTCATTCAACATTAACATAGAAATTTGTGGTTTCCTTTTAACCACACACGTG

TGTATTGTGTGGTGTGCATTAAAAAAAAAAA

>50

CGCTAAATGTCCTTGCTATATCAAAGGAGATTTAGCCTATCACCAACCAATGAGCATATC

ACACTTGAAATGTCAAGAGTCCGCCGATGATCCAATATGTGTTGATCTTTAATGAAAGCA

GTGCCTTATGATAAAAAAAAAAAAAAAAAAAAAAATAAGAGTGCAGAAAACTGATGTTGT

GTTAAAAAATTTTCATGCTCAAAAAAAAAAA

>51

CATGTTCGTTGAAATTGAATGAGGTCCACTCACTCGTGTGTGGACGATATTGAACATTTT

CAAAAAAAAAAAATGTTAACGTGTGGTTTTATCTCAACCACACACATGTATGATGTATCT

GTGCATCATTTTGATGACTCTGGTACTCGATACAACCCATCGGATAATTGTGAGAACTAC

GATTAACATGATGATGATTCAAAAAAAAAAA

>52

ACCAAATGTTAGATTGTTTTTGAAATTTGAACAGACTGATCACCGAATTCACACCACGGA

CAGAGTGGCACGGACATGACATTACATATTTCGTGATTGGGACGGGTCGATTTGGAAGTG

GCAATGTCGTCTATTGATTCAAAACATGCTTATAAGATACATTGGTAAATTGTTGTCATA

ATAGATAGCGTCGGTTTCCCAAAAAAAAAAA

>53

TCGTTTCCCAGCCTTTTCGTTGGAAGCGCTTGCAGGCGCATTGACGTCAGCGTCAATCAC

TTTTCCGGGAGCCATTGACACGTTGCGCTTCGCTCCAGATATTACACGATACGTTCGATG

CGATCAGTACTAGCACTGTGCGTTGAGCGTGCACTGTCGCTGGTTCAAAAGTTGCCGTAT

ATGACGAGGCTTGTGTGAGCAAAAAAAAAAA

>54

TCGCTCGACGCGCACCTGAGCGAGGGGCTTGGGTACGGGCCGGAATGGACACCGTGTTGC

GATGACAGCCCGGAACACGGCCAGGTGCGCATGAAATTTTAGTGGGCAGCGTCATAATCA

TGGCTTCGAGTGTGGGTTCGGCAGGGGCAACCACGTGGGAGCGGGCAGAGTTTTGTTTGA

CGGCTACAGGAAGGCGCAAGAAAAAAAAAAA

>55

TGATCCACCTGCGATGAAACCGGTGAAGTCTGTAAGTTTCGCACCCTTGCCATGCATAGC

CCACCTGTATGGTGAAGGGATAAGTGCCCGTTGACTCATTCACATTGTAAGTGCAAAGTT

TTCATGAAGGGGATCCGTGTTCCTACCTTGAAACAAAAAAAAAAAATAGGTGATGTATCA

ATGTGTACAAGCAGAAGTTGAAAAAAAAAAA

>56

GTGAAATGACAGGTGATGGTGCGACAGCCTTGCTACATTGGAGAAGTATGCCTGCAAAAC

AAGTCGACCAAGCTCAGACAAGGGTGAACATGGAGTAGGTCAAGTGTGTGTGGGATCAAC

GCTGGATGTGCGATGTTCTAGGAAACCAATACAATACCCTGATAACTATGTAGAATGTAC

CTTTTTTTCCCTTGTCTTCCAAAAAAAAAAA

>57

CAACACTGTGCAATGGGCATTCGATGTTTAATCTTAAAAAAAAGATTATTTACATACACA

ACATAACACCTACTCAAAAAAAAAAAAAAAAAAATCGTAGTCGCAGCATTCACTGTACTT

TGTTCCATGTACTATGTACCAGGTGATATGTACTATGTGCTATTCAATATGTACTACACA

CTGTGTGCTGTGATCCTTGTAAAAAAAAAAA

>58

TTGTTTCTGCACTCTTGCGTATTCTGGGGTAACAAGTGTGTAGTTCAATCATCAAACATG

TTTGCTGCACGGATTGATATGAACAACCGAGCAGTTTATTCAAAGGTGTTCATGGGATGC

GGACATCATATTTGTTTTACGCTCTGCAACATTACATATAATGTATGTATATTGTCATTG

ATATTAGTCATGGAACCGCCAAAAAAAAAAA

>59

GATTTTTTGTACACAAAGTTGACACTCCCAAGCACTCGCGCTGCAGGGCTGCATCGCTGT

CTGAGGTGATATGTAGACAACGGTTGAGGATCGGACATCACGATCATGGATTTGTTTAAT

GCAGTGGACGGATATGTGCGATGTGAACGTATTGATTGGATCTGTATGTTGATGTGCATA

GACAAGCTGCATTTCGCACGAAAAAAAAAAA

>60

TGTTTTGGCACTTTCTCAGTATCTTGAGCCAGTTTTTGTGACCTCCAATGTGCAAGGAGG

AGTGTCCAGTCTCGAGTAGATAGATCAACCTAGTAATGGCACTACGAATACCCATAGGAA

GTGAGAACAATGAAAATGTATTTATTTATTTTAGTGCAAATGTGTGATAAAAAAAAAAAA

AAACATTTGATCCCATTCACAAAAAAAAAAA

>61

AAGCATAATGCAATTTCCTCCTTGGCATGAAGTTTTTAGCTGGAGAGCTCCATTGTTGCT

TGAGCTGTATTTGCAAATATTAGAATCCTAGAGCAAGGTTGTAGGCAACAAAATACAGTT

TATCCACAATCCACCGACGTCAATTCCTATCCTACACACACAACAGTAACATGCTTAGTG

TAAGAACATGCTTTACCAGTAAAAAAAAAAA

>62

ATCATATTCATCGACGCGGAAGCATGCTGGATTAGTGTGCTGTGAAAATTTGCACCCGTG

TACCGGTGTTATTTGGCCACTTATTGTAATGCCCTTGCTGAGCGGGCAATGGTGTGACCC

CTTGAGAACATGTATACATTTTGTTCCTGAGTAAACGAGACGCAGCCAAAGTGCACAGTA

CGGTCTGGCGCAGTTCTCCCAAAAAAAAAAA

>63

AATGCCAAACCTGCCTGCAAGGGAGAGTGCTGTCAGTAGGACAGGACCCGCGGCCGTACC

TTGATCAAGCATGCTGCTTCAACTCTCCAGCACTTCCTCAGACGAAACAGGACAGGTGTC

TGTAGGAAGTATGCAGGCACACACACAGAGAACAACATCCCATACTTGCAAATCTCATCG

CTTTTCCATTTCTGATTCGCAAAAAAAAAAA

>64

CACCCATTATGTAATTTTGTGTTACTTCACGCATAGATCAAGACATATTTACCTGGATTC

CTTTCTTGAACACACATACACATTTTCTCCTCAGATAATCAATTGACAAACTCTTTCATC

GATTGTGAGCCCCTGTTGAAGTCATATTTAAAAGATGTCACATCCATCTGCCACTGTAAG

GGAACTAGACGGACTTGAGCAAAAAAAAAAA

>65

TTCGTCTTCTGGCCACTTGGCAAGTGAAAAACAAATATGTGGCAAGCTAGGGGAAGCGCA

TCTTTTCATTAGGAAGTGTGCGAGGTCTTTGTGTATAGTAGTATATTATGGCGTCACTTA

AGTCGTTATCGTGACGGTTGGTGAGAAATATGATGTACTGTAAGATTCAAGGTATGATTC

AAGATCAGAAAGTCTACTGGAAAAAAAAAAA

>66

CCTAAAAATTGTGATTTCCTTTAAGTGCCTTGAGTGAGAGCAGCCAACGAAGACTCTCTT

CGTGGTCCAAACTCCGTGTTGGTGAACAGTGAAAAGTGGTTGCAGGATACCATTCTCAAA

TGATGAATATTTTTCCTTTTCCTAAAAAAAAAAAAAACAGTTATGATGGTTGTTAATGTA

ATTTACGAGGATCATTTGGGAAAAAAAAAAA

>67

AGAGTTTGGCGGACGGATATTCCTTGTACAAACTCGGGCTACAGTGGTCCCAAGACGTGG

TGGTATGTTCGTCCAGCAGAGACTCGATACAATAATACATTCCAAATCGCCATTTATGCG

CCTGGGAGCGGATAGATATTTGAGTAATTGATTAAACTTATGATGTCGTAAAAAAAAAAA

AAAAAAAAAAACTTCATGTGAAAAAAAAAAA

>68

GAATTCGCTCCTTGAGGATGATCTTAGCAGTGCTTGTGAACACCGCCGCAGGCGTTGAGT

GAATCGTATGTATCCTTCGTGGACACTCAAGTCCATGATTGCGCTAGACCCTAGCGCCAA

GCTATGCTTATGCCTGGTTTCCAACGCTTTCTGAGGGTGAACAATTGTATCGCTAATTTC

AAATGATTTTAGAACAAGCCAAAAAAAAAAA

>69

CGCGCCGGCCTGTGGTATAAATGGTCCCAGGATCAAACCTCGCATGTGTAGCTTTGCCTC

GGCGATTTGATTTATCAATTAGCCATGCTGGCCGGCGGATCAACCTCGATTAGCGAATGA

TCATACCAACCGCCTAAACAATCACCATGATTGCTGGTAGTTCAAAAGTTCTGATTGATG

ATTTTTGGTTAGTAAAGTTCAAAAAAAAAAA

>70

CAAATTACAGCAGATATGGATGGAGGGTGCTGCTCATTAGGTCTTGTGGGCTCTCTTTTG

AGTGCGTTTATGGAGAAATTTAGTCTATGAGCAGCGGGAAACAAATTATAGTCGCATGTT

AAGGACCATTTCTTTTCTGAATGTGGAAAGAAATGTACATTGATAGCTCTTGATCTCTTG

ATTTTAAGTATTTGCAAATCAAAAAAAAAAA

>71

CGCCCTTTTAGCGAGCATGTTACAACGTTGCACTTGCAGACACTCGGCTCGCAACGCGTT

GCTTGACCGGTTGCAACAGAGCCGAGCTTTAGTTATACAAGGTTTCTGCATGAGATCAGC

AAAACGAATCTCGGGATCCTTTGTGAAGATAGAAGACTTTTCTCTTGACCGAGTGTAACA

TGTGAGTAGCCGGTGAAATCAAAAAAAAAAA

>72

TAGGGCGGAGAATATTCACATACAATGAATGTTGGAACATGGGTGCGTGATTTTGGTAAA

GTGAATGCATAGCTTATATGGACATGCCATCACTCGACGCATTCTCCCGCACCGTCGTGA

TCTCCTCTACAAGTTCACAGTGTATTTGCAGATTTCCTTATTGTCCTGTTCCAATGCAAA

AAGAGCACGATTGTTGATTCAAAAAAAAAAA

>73

GCCTGGGCTTCTGCAAGAGCCTGTGCATGCGCCGCCGCCGCGCTGGACGACAGCATCTGC

GACATGAGGTACTCCGCGTGCAGCTGCTGGCTGCTCATGTCCGCGCCGTCCCGCCGGGTG

CTCTGCGCGGCGTGCGGAGGAGGAGACTCCGTCGAAGTGGTTGACGGCGCAACACTCCGA

GGCGCCTGCTGCTCATGGGCAAAAAAAAAAA

>74

ACCACAATGTCATCCCCACGATTTCTGCCAAAAGCGAGCTTGAAAATGACAAGCAGAACC

ACGAATAGAGCGGCAACAATCGCATATTGGGATCCAGGAGGAAGTTTGTCTATTTCCGTC

AGGAAATCCATGATCAACCTTCCCGGTTCTCACTGTGCTGGTGCGCCAACGCGGTTGCCC

CTCCCTACGCCGCCCGAATGAAAAAAAAAAA

>75

CCACACTTTNTANCATCATCAACCATATAACCCAANNANATCNNTCTAGTGATCCCCCCA

AAAAAAAAAA

>76

CTGTGAATGCTGCGACTACGATGAAACGATGTATTCGGTGGGTAGTCTTGTGTTACTAGC

ACACATGCAGTGATGTTGGTTAGCATTTTATAGGATTTCCTTAGGGGTTTTTGCGCCCGA

GTACTCTACGTCTGATTGAGTTCATGTTCATTGCTAGCCTCATCATTAAAAAAAAAAA

>77

AAAGCCTGCTTTTCCGTGCAATGGGTAGACATGCTTAAGGATGCTCCCTTGTGGTTTGAA

TCAGACATTCAAACCCCGAGTAGCAGTTTGCTCTGCCAACACCAAATGCAGACTGTTTTT

GAGTGTCAGTGGTGCTTCGTATCAAAATTGGCATACAGTGATGAACTGTTTTTGTTTGTA

TGTAACATTGCCCCCTGTCCAAAAAAAAAAA

>78

AAAAAAAGTTGCATCCAAAAAAACTTACATTGAGGCATCCGTCCAACATCAGACAATGAC

GTCTTCGTGACAAAGCAGGTGTATGAACATCTTCTGATGGACATATGTGGTTTGCACCTA

CAATCAGATGCGATAACACGTTAACGTATGTGGAAAAGTTATATCTAGATTCGTTGGTGA

ATCCCAAGAAACAATGTTTCAAAAAAAAAAA

>79

AGCACAAAACAGAAACGACACGAAATACAATGCACTGAAAACTTTTAATCAGGTTCATGC

TCATGTTCATTGGCAGGGGAATAATAGCTAGGTTTTAGGAAATGCGATTTTCTGACACAT

ATGCCATGTGTTGGAAATACGAATACAGACAAAAGAATGACGACCCTAGCAACCTGCATG

ATTTGCAACTTGCTGTGAATAAAAAAAAAAA

>80

TAGCTGTAGGTTGTGCCACGGTGTCAAGGGCAGTATTCGTTTTGAATACCTCACCATATG

ACTGGAAACCCATACGGACAGCCGGTGATGTTTGTGCTGGTTAGGGTTATGATGTTGATG

AGATGGTTAGCAGAACTCTAATAACGACTGTCGAATTGAAACTTGAAATGTTAAAAAAAA

AAAAAAAACCGTTGGCATACAAAAAAAAAAA

>81

GTACTAGGTAGCAATAGCTACTAGTCACTTAGCATGAGTCGATAGACAGCAAGGGAATAG

AACAGTTATCGATCATTTGAGACAATATGCCGAGTGCAGACCATACAGCCGCTAGTCATA

AAAGGCTTAGAAACAAACTAAAAAAAAAACCGTAACTTGAAGAATGCGCTCGTACACCCA

TGCGCTCATTCTGACCCACTAAAAAAAAAAA

>82

TTCCTCTTGCGGTGGTCGAACATAGGGGGCACGTACAGCACGTCCTCTTGCCAATCCCAA

AACTCCCGCTTCACGCCCACCAGACTCTCTATGCGCTTGGCCTCCGCCTCCTGCTCCACC

GTCAGTTGTGGGCCTTTGAGGTGCTCGTGCTCGATGTGCAGCTGCTTCCGGGCGTCAGCA

ATGATCTTGGACTTCTCCCGAAAAAAAAAAA

>83

AGAGTGAACACTGATTGAAAAAAATCATAGTACAGAACCAAAGGGCGGTATGAGCGCTTT

GCTTTTCTGTTGCTTTGGTTCAAGTTTGAGCACCAATCTGACTGTTGCCATGATGCTGAT

TTTGTAGGCCAAGCAGCTCCTCAAGAGAGTGAGGAGGATAGATTCCAGGCACCTGCGCGT

CCTCAAGGACGAGCTCTGCCAAAAAAAAAAA

>84

CCACTGAAAATGTACCCAGTTCCTGAATCTAGTTTACAACAGCAATTGTCTTGTACAATA

GAAGCGCACAGGTCTACCTCGCCACTCAGAGCTTTTTTTGCAGGTTGAGGAGAATATAGA

GGATTGCCCATCCATCATGTGTTGTGGGACATTTATACATTCCCGGTGTATTCCTTCATG

GTCCTTGCACATTTGTGATCAAAAAAAAAAA

>85

CTGGTAAGAGAAACTATCTGAATTACGAACTGCAGAGCTTTGTGAAGCGCCGCATCTGCA

GCTAGTCGTACATGAAGTGGTTGTCGCACATGTGGCTCTCCTGAGTGATGAGGACTTCCG

GATAATGTCCCCAAGTCCGATATTTGCGTGCAGTGATGTTCCTTGGAATACATGATACAA

AGAAAAGGACATATAAGCCTAAAAAAAAAAA

>86

CATAGCAAATTCAAGCTACAGACTTACTTGGCTTGGATCTGGAAGTGAGGGAAGAAGCCT

GACATCGCGTCGCACTATGCTGATTGAATTATCAACACATACAAGTCTCAGAAAATGAAT

TGATTGTCAAAGTATTTGACTTCACTATGTGACTGGTAAACTGCGATAGCAATTGCTTTT

AAGTGTATCCAAGCAATCTGAAAAAAAAAAA

>87

ACCTATGGATGTTTCGCAAAGTGCGTGATTGCACATGCATGACAAGCACATCGTATGCGC

AGCAATGTTGAGCATCTGATGCCGGATTGCAAATCAAATTAGAAGGGACTTGTCCCAATC

AGAGGCCCTGTCGTAAACTGTTCCATGGATACACGTGTTTGTGTGTGTCTGTGTGTGTGT

GCGCAATAATGTGGTGTTACAAAAAAAAAAA

>88

AGCAGATAGATACAGCCAAAACAGGGACGATATGCAAGCAAGAGTTCATCAACTGGTGGT

TTGCTGGTGATGTAAAAGTTAACCGTCAGTCTCCTGCATCGTTGTTCTCCTTGACTAGGG

CTCCGCAAAGACAATAACAACGTGTTTTGAAGGTAAGCAACACTGGAAATATGGCAATAA

TATAATATTTCTTGGCGGCCAAAAAAAAAAA

>89

GAATGGCAGTTGAGACTCCATTGATACCCCTGATGTTGCTCAGTGAGTGCAGCTAGACGG

CTTGGACACGTACATTATGTGTACATGTTACGTGGTGGTACACGGAGGGTTTCTCTTAAC

GCTGGATCCGCCAGCAACTTTGTTGATCCATTTAAACAATGCGCAATATTGTGAACGTTT

ATACCGAGAAGCCTGCTGCCAAAAAAAAAAA

>90

GTGCACTAGTTCTTTAGGTGAGGACAATTACGAAAGCAATTGTTCATGATACTTTTCTGG

TGCCAAGTACGGTGTGCGAAAAAAAAAAATTACAGTTTCACGTGGCTTCAACAAGCAAAG

GTTCACCCTCATCATAACCAGGGTAAACAAAGCCCTGGTTGTGATGGGGCATGGCGATGG

CTTTGGTACCATCGGCTTTTAAAAAAAAAAA

>91

TCTACAATTAGGAGTTTGAGGTCAAAGGGTTTGGTCCCTTTGTCCTGAAGAATAGGATCG

AGATGCCTAGAGGGAAAGCTGAGTGAGGGCTGAGGCTAGATTAAATGATTATTCACTTCA

AGTGATCACTTGCAATATTGCACTACACAGTGCTTAACAGGTTGAATCTGGAAATCTAGT

GAATGGAGCTTCAATATTTGAAAAAAAAAAA

>92

CCGAGTGAAAAGATACCTAAATCCTGGCCCACGAGTTCGGTGCTGTATCCCCTTTGACAC

GTGTCTTAGTAGCCATCGGAGATCGCCCGTGGTGGGGAGGGGCTCCCACATATAGAATGA

TCAATATCGCCGATCAAACGACCAGACATTCTAGCGCTCTTGTAAATTACTTTCGTCATG

CAATCGTTTCAAATGTTTTCAAAAAAAAAAA

>93

CAACAGTTGCCGCACGGCGTACGTGACGAGTTTACTGAAGAAGATCACCCATTGATTTTG

CGCTGACGTTTCTTCGTAATACACATGTGCCATTAGTATGGGTTCTATATTTGCAACGTA

CGGCCTTAGTAACCAATGTATTTAACCTGTTTTTGTGGTTAATTATCAGCATCCTCAACA

GAGGAGTAGCCGATTCCTAGAAAAAAAAAAA

>94

GTAGAATATGAGTTGTTTCAGTGAAGCTATAAGACCGGCTTCTGCTATGTACATACCCAA

GACGCCATGTACAATGTGCTGCATTGTAAACGTTACTCAGGATGGGTAACCATAAATTTC

TAGTTACTTAGGAGTGCTTTCTATGGCAGATTTAGCGTTGTTTTCGCAGTTCTATGAACA

TCATATGCACGTGTTCGAGCAAAAAAAAAAA

>95

CGGATGAGTTGTAGCACCAGTCCGAGCACTGGTGCCACCCGTTGCAGAATATTTGACAGT

GTGTGTTTATGATTGCCTGCTTATGGCATTGCAAGCATCTACAGTGTAGCGCAGGTTGGG

ATTGTTGGCTATTCTGACTAGTCATGTTGTGAAAAGCTCACGCAAACATAAAAAAAAAAA

AAAAAAAAATTTTGATTGTGAAAAAAAAAAA

>96

AACCTTAAAGAGCAGTTCGTAGCGGCGCTCTCATTTGCGCGGAAGGCAGGGATGGTGAAG

GCGGGTGACAACGTAGTGGGACTTCACAAGGTGGAGGAAGATGCTATCATGAAAATTGTT

ACTGTTGGCAACCAGTGAATCATGTGTGAAGGGACAAGTATGCGATTTTTTTATTGTAAG

TTACATTTACTTTGGCAACTAAAAAAAAAAA

>97

TGTAATGTATCAGAATGTGAAAGAATCAACTGCACTAAGGACATACCTAGGCAGGATGGC

ATATGCACCTAGAAAAACAATGTCCCAGCATAGATTATGGGACTGATGTGTGTCTTGAAT

CATTTTTGCAGTTGCTGTGAATACACTTCGACTATCAACAATTATTGATGCTGAAGGTAT

GAATATTGAAATTGGTAGCTAAAAAAAAAAA

>98

CCTTGGCGAGTGCATGCGTCAAACATGCACAATCTGGTTGCACCAAGGACCCTGTCTTCC

AACAAGACACACATCTTGGTGTATCCATTCTGTTCTTGAAATGCACTGAATGTCACGCAC

TAGATGCGGTGATCTGAATTTGAGCAGCTTTGCTTTGGACCGTGTTGCTTAACCCAATTA

ATGTAAGATTCCATTTGACTAAAAAAAAAAA

>99

TGCACTATGTTTTCCATTTAGACAAATGCCCCAGCGTTTGATCAGGGAGTGGAAAATAGA

AGACAAGACAGTGGAAAATATGTTTGTTCAGCCTTAGTGATAATGCAAGGAAATACACGT

CAACCAGTATATTTCAGCGGTGATTATGTGGTACAAAAAAAAAAAAAAAAAAATCTCAAA

TGGGTTCGCGCTCATCTCTCAAAAAAAAAAA

>100

GGGGTACTGTTTCTGTGCGTGAAGTCTGTTTCTATACAGGGGCTCCATGAATTTGGTAGC

CACCATTTATGCAACCTTGTTTCCAAGGTTGGGCTCATCTATCTATGGGCTGCTGCTATG

GACACGTGGAAGGCATATATAGGCCTATGCATATCCACCCCATACCATTTGCGTGACAAA

CGACTGGAGAAGAAGTCCGGAAAAAAAAAAA

>101

AGCCATCTCACAGTGACACACAATGGTGCTTTGGTCTGTACTTGTAGTTGTCTGGGCATA

CCAGATAAATGAGATTGTTTCGGCGTTGCACGACACGCGCTGAAATTAACAGAAATTTGC

AGAACTGTATTGATGAGGGCTGTTTGAATCATACTGGGAACATGAACTAATCATCCGAAT

ATATACAGACTCTCTTTTGCAAAAAAAAAAA

>102

CTAGTTTGTGCAGACAAATGTACTTCACATAAGATAGAGTGTTAATCAATTATGAAGGAG

GAAGAGGGGGTGTGTCTCTGTAGTAGACGTAATTCGATTTTGTGATCAAACATCATGATT

GCAAATTCCTTTCATCTCGCACTTGGATTTCAAAAAGGGTGTAACGGGCTCCATAAAGAG

CAAATTATTTACAGTATTTCAAAAAAAAAAA

>103

AAATGTGTTGACGTCTCACGTGCAGAATCATGCGGACATTGGCACACCAGATGCTAGAAG

CAACAAGACTTCTGACAAGGGTTTCATCAGCAGTCTTGTTTCATTCACCATTCCGGAGTG

CAGCCGCAGGGTGGGAGGCTGTGTGAGATCCTCAGAGATGCATCTGGAACACTTGACGCA

GCAACAAGTTGCATTTCACCAAAAAAAAAAA

>104

CCTGGATCGAGACATACACCAAAAACTTGACCCACGTGTCACAAACAACCGTGAAAGCAT

GGTACAATTCGTCAGGTTCTGGAGAACCTGACTAGTTGTACCATGGAATGTCGACAAAAG

TGATGATTCAGGTGATCCAAGTATCTGACATGCACCACCGCACTTGGTACACATAACCTA

CATGGTTATGGATGCCGACGAAAAAAAAAAA

>105

TGCTGGCCTTCGTAAGGTGTTGCCGGAGTTGGTACTGTTGATGAGCTTCCACCGCCGCCA

CCTGTCCACCAGAAGTGTTACCAGAAAATGCACCATGAAACGCATTCAAGCGAGACGTAT

CGTACGACACGTTTGATGGAAGAAGTCGCAGGTTGTCCAGGCTGGCCTTGCTTGAACCTT

GCACGCAACCCCCTTGCTCGAAAAAAAAAAA

>106

CCAAGTATGAGGCTCCCATGGAGGAAGCACATTAAATAGCTGTTATGAGTTGTGCCGGCA

GTTTCTGGAATGATTAGATGGTGTAGCTGCGTCAGGACCACCTCAAGCATGCACCAGTAT

CGTTTCACTCTTCACACCTCAACCCAGTTGCTAAAGTAAAGTTTGAGTTGTGAAAGTCAA

AATGCTGTAAGAAATTCCTTAAAAAAAAAAA

>107

CATTCATCAGAGCTTATGATACAAACAACTGGTTTTGACTTCGTGAAGTTTGTTTCAATT

GGATGTGCAAGAAATGTCAAAATGGGCTGCCAGGGACTATGAACCACTTCTCAACTAATT

TTACAACATATCATGGATTGCGCTGTTTGACCCGGAAAATTGGATTATGTGTGAAGTAAT

GGAATCTAGCTCGTAACTTCAAAAAAAAAAA

>108

AGTTCGGAATGTTGACAACGACTCAAGTCATCGCGACCGCATCGAGCCCTTCAGTGTGGA

AGGAACACAACAATCGCAATCCCGGTGTGGAGTGCGAGAACAATAATCCTATAACAAGGA

AAAGTAAGTTTCGAGTGCCAAAAAAAAAAAAAAACTCTTGTGAAGCGGGGACCATGTCTG

GTCTCTACCTTAAAATCGTCAAAAAAAAAAA

>109

AGACGATGAAGATGTATATCAGATATACAGGGAACTTGAGGATACTAGCAGTGATGATAA

TGTATTGCTAGCATGAGGAAACCATGCACCTGCACCTGTTCTTTTGCTCTAGCTGTAGAT

GACCTCCAAACAAAGAAGAACTTCAAAAACAGAATGTCTCCACTGTTATACTGATGCATC

GCACGTCGAAGCTGCTTTCCAAAAAAAAAAA

>110

ACACCTAGCCAGTAACAGCATCCAGGTGCACTTTTCTGCGACGGCCGAAACTCTTGTTTG

TGTGAGTAAAACGATAAACTAACACTGATTTCATTTAGGTGGACATGGCATTGCCGTTGA

ATTTTACGGACCAAAAATGATGGCATTGCACCAATGTTTGAAAAAACCTCATACACTGGG

AAGATTGCTCATCCTCATACAAAAAAAAAAA

>111

GCACCCATCGTCAGCTGTACTTGCATATTCAGAAACACTCAGCTTGTCTTGTAGACGTTT

CCGTCTACATGGTTTGCATGGGTGTCGTGCTTGTAGCTATCCCCAAGACTTTGCATGTTG

GCACAATCACTTTCTCGAACAATCCTACAATTATTCCAACATGATTTCGTTGTACTCCTA

AAAAAAAATGCACTCTCTTCAAAAAAAAAAA

>112

ACAAACTAATGGCAGCGAAGTACAAGTCCGCAATATGATCACACCCATGTCACAAAATTA

GAAGATGCAGGACGTTAGATACGAATGATGTATATTGGGGCGTGTCTGTTGGTATTATGC

AAATTTCACATGAATATAGGTCATTAATTTGTGGTGCTCTGTGCACTGTTTTTATCCCCC

TGAAGTCTAACGATGTATCGAAAAAAAAAAA

>113

ATGATTTTGCAGACTGCATGTGTTTCGAACAAATGGATAGGCTGTTAAGCATTTTGTGGA

CATGAAGCATATGTTCACGAATGTCCTTTGGGTTAGTGCCATGTCCCTATATATATTGAT

CATCATTGTCTGTGTAGCTGTCTGCTTACCACTTTTTCTCAGTAGAAAAGGTAATGATTA

ACGTTATTTTAATTTCTCATAAAAAAAAAAA

>114

GAAAGCCCACTTCACGCTCAAAGTTGCACCTAAAACTGCAACAATTCCATTTCTGCTTTT

ATAGCTGAAGAAGATTTAGGGTTATCCATCGATAGCAGGCATTGTTCAGTACAAAATTCC

ACATAAACATACATGTTGGCCTGATATTCTTGTTAGGCACATTTCAGCAAAAAAATTAAA

AAAATAATGATTTTAGGCATAAAAAAAAAAA

>115

CTGTGCCGCTAGTAGAGTAGGGTCTAGCCGGACTGATTTCTCTACCAAGCGGGTCATATC

TGCATTAGAGGATCTCTGTCCAGAGGCTCTCCCCTGAATATCGTGAGGTCATCTGACGCA

GTTCTTGTGTTTGGGAAAGAAAGTTTTGAAGACCTTTACATGCAAATTATCTAATTGGTT

TCTTGTTACATGACCATTCGAAAAAAAAAAA

>116

GGGGTTCAGGACTTAGGGTTTATATAGCTGCATTTTTTGTGGCTGCATCCATGTGATGCA

CTGAGCCTTGCTGAGAAAAACATGGCAAGCATCCATATGGACAGACCGTGAATATGTCCA

TTCACAAGGAACATCTAGTTGCTGGTGGCTAGTAACTCAACATGGAATCATGAAGTAGCA

CATGTCATAGGCATCGCGCCAAAAAAAAAAA

>117

TTCTAGGCGCTGCCAAACTATGTAAAAAAAAAAGTCTCGGCGAAGAGCGTGTGGTAGTTC

TTGGTATTATTACACCTGTTGACAAGTGTGTTTTGACCTGGCACACCGCTGCTAAATCTT

GGGTTACTCCCCCGGCCTCGAGGGTTAGGGTCCGTGTATGGCCCCAATTTGGCCCCGGAA

TCCAAATTTGGGGGTAATTCAAAAAAAAAAA

>118

ATGTGCAACGACCCGATCCTGACTGTTGTGCATAACATCTTCTAGAGAATGGGCATTGCT

GGCGACCTGACGACGTAGCGTACGTCGTGCAGTTACATGGGAGGCGTATGTACTGCATGA

TTCTTGCATGCGAGAGAGCGATCGATTGATAACATTCTTGGCAAGATGTTGTGATTGTAC

AGCGGGATAGATGATTTCATAAAAAAAAAAA

>119

CCACCAACATCATCCGCATATATTTGGGTGCCTTGTAGTCTTTCACGTGTACGCTTTCAC

CAATTTTCATTCCATTTTCTTTGATTTCGCATACAATGATTGTTGCTGATTATATGTGAC

CTCTAAAAGAATTAGCAATTGTTCCTCTTGACATTGATTTGTAGAGTTATATCACAATCA

TTTGCAACCTACATTCCTTGAAAAAAAAAAA

>120

TCCCAGTATGCTGTGTGCTCTGTAACATGATTAGCTTTAGTTTTGACTGTAGTATATGAA

TGTGGGTATTAGTACATGTAGCCATGCATGATTTTCGAACTGTCACAAAAGTATGTCCAG

GAACATGTATGCATGTATGTCTCAACAATCTCATGTCCTAACAATCAACAAAACCATTTG

ATGCCATTATAGTTCCGTATAAAAAAAAAAA

>121

CTCTATGCGTCAGGATCGAATGTCCCAGCATTGTCTTGGTATCCAACAATAGAGCATAGT

CACCAGGGAACAAGCGATAGATATGCTCCTTGGAGAATCGGAAGAAAACCTGCCTTCCAG

CCGAGAATGAAAGTGCTTGAGTCAGTACTCTGTTCAGCTTTTCACATGTGTGTAATCCGA

TATACCACAGATGTTGAGTTAAAAAAAAAAA

>122

CACCACATAACCCAACCATGCGCTCTGACAATGAGGTGGCGAAGTGACGACCTCCCATGA

CAGCGGCAGTGGTACACACCAGGTGACCCAAAACCCCTCCCAGCGTCACACCCACAACAT

TGGTAGATGCTGCCAGCCCAATAGTTGCAAGCTGGCTGCGGTCGCCCCATTCCGCCAGAA

AGGTAAGAGAGAACGCTTCCAAAAAAAAAAA

>123

GTTTGAGCGAAGTGGAAACTACAAGAAAGGAGGCCAAAGCTTCTCAGGTTAACCCAGCTG

TTAAGGGTGCAGAGAAAAGGAGCCAAGACTGGAAAATCAAAAACAAACTCACTCGGAGCC

CCGGGGCGACGGAGGAGGCGGTAAAACCTATTGCGAAGGTGAACCGAAACGCTCAGCTGT

CTGGGCAGGAGAAACGACATAAAAAAAAAAA

>124

ACAAGTGCTGTCCTCAGTCGATATGAAAAAAATATTCCAAAGCCCAAATCCTATCTCGCA

TGTCGAATTACATCGCACAAAACCAGCTTCGAAGGTGTGGCATTGAAAAGTATTCATGCA

ATAGCCCATGGACACAATATACGACTTAACTTTGATTTGAACAGTGTTGCTTCCTAGAAA

CTTGTAACTATTAGCAATAGAAAAAAAAAAA

>125

AATCAGTACGGAGGCAAGGGAGGCTGCCTGTAGGTCAGCGTCCCGTACAACCGTAGCAAA

TCTGTCAATTTGAACTGTCTTGAAGCAGATCTCCAGAGAGCATGGATGATACTGGTTATT

TGATTGCTGTGCAGCAGATTCCGCTTGTGTAACATGATCGTACTTTTTTAATGAAATCAT

TAAGTCGAAATAGTTTTACCAAAAAAAAAAA

>126

TTCCGCAGCAAGGTGCAAAAGATGCTCAATGACTAAGGTACCGCTTCTGTATATACTCAT

TTAGAAGAACAAACTTGATTGCTGGAATTTTGCAGATATTTGCGTTCAAGATGCTTGTTG

GATGATCAGTGAATCCAAAACACCATTGGACCATTGTTCCACAGGGATTCTCTTTGACTG

AATATACTGTCTTCTTGATCAAAAAAAAAAA

>127

GCGATGGTGACCGTTTCACCTGTCACCCCATTCGAATTCAATAAGCGTCACTAGTCCTCT

CGTCAACAGCTCTGTCGCTCGCAAATGGTCACTTCACTCAATTCCAACATTCTGACAAGA

GCACCTCGGGCATGTCTGATTGTACAAGCACATTTCAGTGGTAAAGATTCGCACGGGTAT

CGTAGTCGCAGCATTCACAGAAAAAAAAAAA

>128

ACCGGCAACGCTATGATTAGCTGGCCGACCAACAACAGTGCAACATCTGGATTATAATTT

CTGGTTCATTGTCCAGATGTGAACTCGCTATTCACATAACCGAAGTGCTAAGTACCCTAA

GTATCTAAATCTTCTAGATGCCAAAAAAAAAAAAAATAAAAAAAATACCTTGCTGTAAAA

GATGATTCTAGTACTCTGACAAAAAAAAAAA

>129

ATCGAATTTCGCATTGGCATTGACAAAGCAAAGAATAGTATGTGAGACATGTCATCGTCT

CGATGTATTTCATCACCCATTAGCGCCTTTTAGAGCGGAACGCAGTGTAGTGCACACACA

CACGCGTTGTTTAGTTTGCACACTGATTGAACAACTGAGTCCATGGGCTCTGAAGTTATA

AGTATACTAGTTAAAGTTCCAAAAAAAAAAA

>130

ACGAGGTCGTGCAGTTTCTGTTGGATGCTGGTGCGGATCCTAAATCGAAGGATCGCTTCG

GACAATCTGCATCTGACGCGGCTGCCCTAGCACTCAAGCAGCTGAAGAGCCAGTAGAATC

CCTTGGTATACTAAGTATTGGCAGTTCAAAAGGTAAACTTCCTTTCAAATATTATTGATA

TTTATTGATTAGACTACGCTAAAAAAAAAAA

>131

GGCTTAGGATCGGTGGTGGAATGGGTTGATGCCGACGAAGAACAGATGATGAATGCAAAC

TAGATGCTTATATCATCGGGTGAGCGTCCTTTGCAAAAGCGCGATTGAGACGTAAACCAC

CACATGGAAAGGTTGATATTCATTATTGAATTGATGACTGGTTGGTGGTAATTCACTGTT

AAGAATATTGTTCTTTGCATAAAAAAAAAAA

>132

TATTTACGCATAGGGTGTGAGTGCTGAAATAGAACTCACCCGATACAGAGTGTCTCCAGA

AAGAAGCGTTCAGCCATCCCGATGTTCTACTTCATCCCTACCGTGTACCGGGGGGTTTTG

AGGCCACGGATACTATCGGCCAGGGAACGATTGTATATCATTATTGGTATCACGTTAAAT

CACATTTTCATCTTGTTATTAAAAAAAAAAA

>133

GTGATAGATTATCTCAGTGGCAACATGTGTTTCCTGAGGTCATTGGATTCCATGTATACA

TGTCAATATGGATTGTGGCTTTGCATCGTGATAGGACTAGAGAAACTGCAATGTCAATAG

CCACAGGGATACACAGAAGTTGTCAAGAGTCTATCACTTTTGCTAATAGAGTTTGATAAT

GAATGATAGTGACGATTTTTAAAAAAAAAAA

>134

TCTATGGTGGCTTTGCGTTGGGACTGAAAGTTTGAAGGCAGAATGAATCTGGGATGCGTT

GTCAGATTCACGAGCAGTCAATCGGACATTTGCAGCAGTACCAAAAAGGATGGCAGATTT

GCAGGTTAGGTAGGATCCCCTCATTAGATATTTCAATTTAGTTGGATTAGTTATTGTGAA

ATTTATTATTTTGGTTGGTTAAAAAAAAAAA

>135

ACACATACTTTGTGGGAGAATTCAATTGCTCGTGTGTGGGGATCACACAGCAGCTTCATC

TTTTTCCGCTGGTTGCTGATGCCGCTATCCAAATTTGTACCCAATAGATTTGTTGAACAT

TGCATATAGCATTAGAAGGTTTAGTATATGGAGACCTTGTAACTTTATCGTTTCGGGTTC

AAGACGTTAGATTTTTGTGTAAAAAAAAAAA

>136

ACGTGTTGAAGGTCCAGGTTGGTGACAAGCGTTGGTTCAAGGGTCTTGAGCCCCCCTCTA

AGGAGGTCATCCAGTCCTTCATTGTGGCGTAAGCTAGCGGATTTACTTAGCGTCGGCTCT

GCTACTACTTAGAACGGTTGGGAAGGGCCCATATAGAAACTTGTGTACATTTAACAGCGA

TACTGCCATTTTATTCGTCTAAAAAAAAAAA

>137

GTCTACCAGGCATTCGCTTCATGGGGGATAGGGGTCGACTCTGGACATACATAGTAGTCT

TGCAATGTTCCTTAGGGTGTTGTGACAGGATGGACCGTGCATATTGTAACTTTCTATACA

CAAGAGACGGCGCAACACGTTTAGAAATATGTTTTCCAAAAAAAAAAA

>138

GAATTTCTTCAGGAATTTGTTGCCGAAGGTGTTTCGTACACAGATTTTGGCTTCGTAACC

AGCAAGATGTTTTCCTTAGGATCCAAGACATTCCAACAGCAAGACTTTGAGATGAACTAA

GCACTACATGGAATGCATGAAAGTAGCCAACTTCCCGATCAAGTATTCGAAGTGGATTTA

TACAACGTCTTGGAAACTGTAAAAAAAAAAA

>139

AACACTCGAACTCTGACCACATACACCAGGAAACACTCCTGTAGAAACTGTAAACCATGT

TTGATGCATCAAACGCAAAACAGGTGTTTCTGAAATTAAACAAGAAAAGCATAGACAGCG

TAGCCGCGTAGCCCTAGGGATATATGACCAGAGACCAGGAATATGTGCCCACAAGTTCAA

AGTGCTGGTAGTACTTGCGTAAAAAAAAAAA

>140

TTGAGTTCACACTCACTCATAAACCCCATCGCATGCGGGACGGATCGTACCATCCCCGAT

TGCGGTATCATATATAATGGGATATGACGCCTACCGACTGGGCGGGAAGCCACATCAACT

AATCACACAGATGAATTCCATAACCTCGCGATATCACTCCAAGGTACAATGCACCAAGCC

TGTCACATATCTACAGATGCAAAAAAAAAAA

>141

TCGCAAAGACCGCTAGAAGCTGCTCCCGACGACCCTGGTCCCACTTTTGATTCACGCACT

GCCAAGCATGCAGAGGCGGAAGATGATACAGATTTAGATATCGGGCCGCAGGCAGTCGTA

ACGTATCACAATGTACTAGGAACAGTGGAGGAAAGCATTTAGAATACTTTTCTCAAAATT

GTATGGTGAAAAAGTAGCCCAAAAAAAAAAA

>142

AATACAGCGAGAGCCCTGATGGTAGTGAGGGACATGGCGGCATTTCGTATATGAAGATGT

GTTATAGTGTATTTCACCATTAGCAGTGGATTCCGACGGGTTGATGTTTTGCCTCATTGG

ATTCCTCGCTATGTATGCATATTTGTTGTATGCATATATGGTTTTGAGTATTACCCTGAG

CACTGATAGAACTTGACGCGAAAAAAAAAAA

>143

TTGGCCAAGAAGGAAAAGCGTGAGAAGGAGCAGGAGTGGAAAAAAAAAAA

>144

ACGCGAATACAAGAACAAATCGGTGAGGTAACGCAGGCTGCAAAAAGCTCTCGTGGAATA

TCAAACATGCCTACTTGTAAACAGGACTTTGATCGTGTACCGTGCACCAATTGAGTCCGC

CGTGCTTGAAAGAGAGTGTGCGATTTTCACACACATACAACGGACGAACACTCGTTATCA

CCTTCAAAACGTTCATTTCCAAAAAAAAAAA

>145

AACATCCAAGCGGGGTGGTTGTGGAACAAATAGAGCGATGTCGCGGTGTTTGGTGCGCAT

AGCGCTCTGGCAGCCTCTTTTGAGCAGGTCCTGTGTAGAGATGTGGCTTTAAAAGCCAAA

GCCACTACCAACACCTGGAAAAACAGCTGTTGACGCTCCTCACCTCTCGAGATCGAAACC

TGTATTGCGCTGCTCAGTATAAAAAAAAAAA

>146

CGAAAAATCTGCGCATCACCGTTTGAAAAGACAATTCGTTTTTCTATCAAGGATCACACA

TGTACTGTCCGAGCCTGTATGCCCAACATGCCTCCCAGATCGTGCCTGATCTGCAAGTTT

TCCTGCACATTTACGTGGTGGGAGAAACGCTTGCACCGCAGATCCAAATTCTGCAGGTAG

GACAGATTAGGACTGTTCCCAAAAAAAAAAA

>147

AACTGAAGATACGGAAACCAACAGTCACATGCACTTATCGAATATACTAGTCAGGGGTGC

ACCTTGTGTGACTGGAAACTACATGATCAAGGGGTTTGTCATAGGTATTGACAAGAGGTA

TGTCAAAGGTGTGAGAACAGCAATCAGCTACTAGATAGGAGGCAGAAATATATGTATACA

TGTATAGGGACATGACACTTAAAAAAAAAAA

>148

CTTGAGCAATTTTCCCACGGCGAAGGGAGCACTTAAACACTAGATTGCTAGCTTACATAA

TATGGCGCATGGGTGTGTAGCATACATTTTCAAGTGACGGAACAGTCTTTCTGTACGCTT

CCGCCAGCGGCGGGTGTATAGTTTGCCCTCGATATATAGAATGACCCAATGGAACACTAC

TCCATAAAGTGGATGCCCTCAAAAAAAAAAA

>149

CAATGTCCGCATCTTCCAGCTTGTCATTCACCGCGAATGTAGGTTTGTGTTGGTTCTTGT

CAGCCGCTCTTTTGCTTACTATGTAAGTGGAACAGAGGTGATGGGTTGTCAACTACAGGC

ATGGCATGTATCTCACCTCATTGTAGAGCCTCGGCTGAATCGTATTTGTGATACATACAT

TACTGCACAAAGTGCAGATGAAAAAAAAAAA

>150

GAACAAGCGTGCAAAGCTGGATCCGACCGCTGCTCCCACTAGTACCGACATTGCTGTATA

GAAACACTGTTGGACGATGGCGTTTTGGTGCAAGTAGAAAGCAGAGTTACACATCTACCC

CCTACTGACTAGCTCACAAGACTTCTCAATCTATCAGAGGTTGACTGTTTACTTGATGAA

GAAAGGAATAATTTGCGATCAAAAAAAAAAA

>151

GCCATACGCATCACCCCTACGCGCTTGCGGTAAGGACATAAATGGACCATGGTTGCAGGT

GGCGCGTGAGCTGAAGCTTACTATCGAACGGTTGAATGTCGCTGGATCGCATGAATCCCG

ATTTCTTTTGCAAAGCGAGCAACTCTGGTGCTCCACCCGCAAAAGGGCTGGTTGTAAAAC

ATCACAGGGACGTTTCGTTGAAAAAAAAAAA

>152

ATGAGTAGTCCATATATCATTCACCATTCCTTGTGCCAGAGGAGACAAGCCACACATGCA

GTCCGCATGGGTACATTTGAGCACTGCTGACGCCCAACATTCCAGTGCTTTGTACAAACA

ATAGCAGATACACTCACCAACGCACAAGTATGCAGGCATGCATGCATGCAGTATGTGAAT

AAATGTGGCTGCCTAATACCAAAAAAAAAAA

>153

TGTGGTACTTCATGTGGATGCTAGTACAAGGCAGCTGAGGATCAACTGAAGAAGTAGCAT

TTTCCAGTCACTGCTAGGGAACGAAATAGTGGCAGATATTAGTCGCTTGCAGAAGAGAGT

AGACCATGTGAGAAGCTGTCAACGTTATGCACCAAGTATCCGGTGTACATGTAATTGAAG

CAAGGTACATTCGTTAGTGGAAAAAAAAAAA

>154

ACTGGAGACGGCATCATCTCCCACAGAAGTATGGCGACTGAAATGATGTAAGCTTTGATG

AATCTACTTGTATAAGAAACTCACATGGGTATCTGGAGATGTCCAGTGAAATGTGTAAAT

ATAGGGACTGTCCCTTAAAGAAACCATTCCTATGCACACTTGCAATGAGAGTGTGAATAT

AGTGCACATATTTGTTGCTGAAAAAAAAAAA

>155

CCTCCTCACCCAAGTCTTGCTCGTCGTTGAAAACAGTGACCTCAATATTTTGGTATGTTT

GAATCTCGTCGTTGCTTTGATCCAAAAGGGCAGGCTCGTACTCATCGTCCGTAGCTTCAC

CCTCGTCCATTATCGAATCGGTAGAAACGTTTCGAGGCATAGCGCACCTTGCGACATGTG

AGTCCATGTTTGTTCCTGTGAAAAAAAAAAA

>156

CATACACAATGAACACCGGGGTCAAGAACCTGCATCCCTAGGGCACACCACCGGCGCCAA

ACAAACAGAAGTATTTGACCTGTGTGGCGCAACGAACGCTTTCGAGAAGGCGGTATTCCT

TGAAATTTGTATGCACAGTGTATACTTGTCGAAGTTGCTAATTTTACCTTACTCGGTGAG

GAGTATCATAGATGATTATGAAAAAAAAAAA

>157

ACCAATGGAAAGAAATAGATTGGTGTACTGCCCACTTGCGGCAAGTGCCATTTTGCAGAG

GCCTAGTTTGAAATGTACACAGTTTTGCATGGTTGATCCTCAGGTATCCAAGCCTTTTAA

AAGTGGTGTTTGTATGTACTCAATATAGTCACATGAATGTACCTGCGTATGCTCAATGTA

ATGGAATGCTTAACTGTGCGAAAAAAAAAAA

>158

CCCATGGAAAGAAATAGATTGGTGTTTTGCCCCCTTGCGGCAAGGGCCCTTTTGCAGGGG

CCTAGTTTGAAATGTACCCAGTTTTGCATGGTTGATCCTCCGGTTTCCCACCCTTTTAAA

AGGGGGGTTTGTATGTTCTCAATATAGTCCCCTGAATGTCCCTGGGTTTGCTCAATGTAA

TGGAATGGTTAACTGTGGGGAAAAAAAAAAA

>159

AGGGTCAGCCATTTCGACGCCGAGCTCAGGGGTGCATTCCCTATGTATATACATATTTTT

GACTGCATATCCCTGCATAGCCTGCACCGCCCCGCGGTCCAACGCTAGAGCGAGTGCATG

CGGTTCATCTCGGGCGCACTGCAGAACGCGCCTCGTGCATCCCAAGAATCAGAGTCCCCC

TCTGGGTCCGCGTACCCCACAAAAAAAAAAA

>160

ATGGAATTGAAATCGCTTGCTGCATATCATATTTCAAGAGTCAAAAATACCTAATGCCAT

TGGCTTGAAGTGAAAGGACAAGATGGGAAAGCAACCCATCAATGTAGGAGAACATTTGAT

GGTTGCGTTGTAGTACAAGAATCATACGGATTGAGTATACAAATTATGTATTGTATCACC

ATTCTAAGTCTCTTTGCTCCAAAAAAAAAAA

>161

TAATATCAAACCAAAACCCATTCATATTGACGATTAGCAATTATAGAGTACATGTGTGTG

TCATGTGAATGGTTGTTTTTTGTAACATTGATTTGGTTCGTGAGAAAAAACAAAAATATA

AATAACGTTTTTTTTTTTTGCATACACGCTTTGTTTGTATAGGAAAAAAAATAAACCGAC

ATCAACAAAAATTGCACTTGAAAAAAAAAAA

>162

ACACACCAAATGCTCTTTTTCGTTCAATAGGTCCGTGAACTCTTCCTTGGACTTGAAGAA

CATCATCTCAAGATGCGACTCGAGCGTTTTCTCATTACGCACGCTACAAACGTCATTACT

TATTTTCCTCAACTTGTCCCTTCGATCTCGCATCTCTGCCATCGCGTTTTTTTTTTCTAG

GCGTCGTCCATTGCGCCGAGAAAAAAAAAAA

>163

ATCTCTTTGGTAAACTTCCCCTCAGGTCCCTGTACAGGAACTGAATAGCGCAGATCATCG

AAAGATATAACCTTGCCAGCCGTTTCCTTTGCCGATTCCACGTCCATCTTCAACTCTTGG

CACTGTCTTGAGGCCCCCGGGTCCGGCGGGTGGCTGCAAGCGGTGAACTCGAACAGGTTC

TGGAAATCCTGCTAAATATCAAAAAAAAAAA

>164

CTTCCCACCAAACTATTNTCANTTTNTNNTCCAACTTTATAATTANNACAATTCTCACNA

ATCTTNCCATACTTCNCANCATCATACAAACNANNCNAAATTCATTCATCATNAATCTTA

TCCATCAATCANCTACCCTATTATCATAATTTTTCAATACCCAATTNNCAACCTTACCTT

CCTAAACCCAACTTTCATTTAAAAAAAAAAA

>165

GCATGTGCTGGAACCGATCAGGGTCATGTGTTTTTTCGCAAAAAAAATCCCCCAGTTTCG

CACTGGTTGTAGTTTGAACGATGACCCAAAACATATTGGGAGTTCTGAAAGAGAGTTTTT

TGTTTCGGGATATTCATTGTAACAGTCTTGATTAGCCCAGTCAAAGGAGGTCCAGAAAGT

TCGTAAGATATGTGGTTCGCAAAAAAAAAAA

>166

AGATAACTAACGCTTGAAGTCGCGGCAGTTTTCACAAACCCGATGCGTTTTAGCTCCGTT

GCGGTGTTTGCGCTCGATTCTACTTCGGTGGTCATTTTCCTACCAGGAGGGTTATCGCAC

GTTGCTGTGTCTGTAGGCTGAACGCAGAGATTTGGTGAAACAAGAAAGCTGCGTCGACGA

TTCTGTTGAAACCCTCTCGGAAAAAAAAAAA

>167

ATAAATGCAGCAAAGTCAAATGACTACAAAAAGGTACTGCCAATTTATTGACCTGATACA

ACTGACTTGAGCGCATTAGTAGTTCCCTACAATTGAGCAAATGAACTCGAAACATCGTAC

TGTTTTTAACCAATGAAGCCTATACTTGATCAGCATACACATTCATGAATGTATGTCACA

GTCACCTTGTCCGAAATGGGAAAAAAAAAAA

>168

CTGGTGGAACATGGTGGCCAGGCGGCTGGTGACGTCGGCGGCGTGGTACACGTTGTGGTA

GGGGTTGTTCATGTAACCGTCCTCCACTGCAAACAGGTACGACGCCATCTTGGATCGAGA

AATCTTGAGGGAGGTCATCAGGTCCCAGTCCTCCATCACCTGAGCCCTCCAGCTCAGTTA

GCACTCAGCAGACGCTACCCAAAAAAAAAAA

>169

TGGGCAACGGATCCCCCGCCTCAGTGTCCCGGGGGTTGTATTTTTTTTTTTTTGGGGCCA

AGGCAAAGGGGGGGCCTGTAAAGGAACCAAGCCCCTTTTGGGGGGGGTTTTGCTTTCCAT

AGTTGGGGGCCCTTTTGCCCGAGGAATGTTTTTTTGTAAATAAGCAAATTTCCTTTTTTA

AAACCCGTCCTTGTTTTGCCAAAAAAAAAAA

>170

AAGGTAGGTTTTTTGGGGTTTGGAATTTTCTTCCATGTGGGGTAGGGTAAGGCTTCAGGC

AATTCTGGTCAGCCAAAAAAAAAAA

>171

TTGCACAAGGCAAGTGGTTCAGACAAGTGGTTCATGCAGGGGAAACTTAAAGGCCGGCTC

GCTGCCATGTCCCCTATATGTCTACAACTGTTTGATCGCGGTTGATAGGCACCCAATATA

GTCAATACCAGGGCATCAGCTTTCGACTGATGTAAGTACGGTTCAAATTTGATTTCAATA

ACATTTTAGCAGTAGATGTTAAAAAAAAAAA

>172

ACCCCACTCACTTCATCCAATTGTGGAATTAGTATGGGAAGCAAGTAACCAGCATAGGAG

CAGTTCATGGTTTGGCGATACCAACACATGTCTTGACAGTTGTGTAGTCAACTTGCACTC

TTCTTTTCCAGGCAAGCTGCCAGCAGGTACTTGCATAGACGTCGAACTTGCGCAAAATAT

AATTGCATCTTTTGAAGCGGAAAAAAAAAAA

>173

ATTGTCCACATGTCGAGGGGGCAAGGGCCCCGTCAACCCGGAGTCCGCTGCTGCGAGAGA

CACAAGCAAACGGCCGTAAGGGTAACGACCGCGGATGGTCACGGGACTAAAATGTGAACG

CGTGGGTGCCTTGCATAAATTCGGGGAGGCAATTCGTGCCGGCAAAACCATTGCGACAGG

TCTGGACTGCGTTCACGGAGAAAAAAAAAAA

>174

AGTGGCTCGACTACTTCATTGAGCAGGCTGAGCGTGCAAGCACGCACTCGGCCACAAACA

CTGCATTCCAGAAAGACCAGATCTCAGGACTTGACATGGCTATCTGATGTGTGTTTGTCA

ACGAGATGCTGTAGTTAGTATTCCCTTCAAGAATGTTTGATTGCGCTACATTTTAAGATT

CTTATATCACCTCGCACAGTAAAAAAAAAAA

>175

TATCTTGTGTTCTAAACCTGTACGACAAACTGACAACACACTTTGGTGGCGGTACACGCA

AGAAGAAATAAAACACGAGGCACGAGCATATAACAACATAGTGATTGTTGTTAAATGCTA

ACTATTGGGCTTAATATAATGATTCACTTTTTTCATTTTTCTATGCGTTTCTCCCGATGT

GAGAANCGCCTCTAGGATGGAAAAAAAAAAA

>176

GCTGCTGGAGCAAACTATATCAGTGTCCTGGGCTTTCGCCCGCGGGCCTCTTAAAAAGTC

TACAGCGAGACGGTAGACTGTGAACGCATACGCCTAGGGTTTGTGGCATTCCTGGGACGG

TAGACAATAATGTTAGCGTCCTTGAACAAAATGAAGTGCTAATATGTCTCTCGTTTTTCA

CCACTTTATAGCTTTGCTTCAAAAAAAAAAA

>177

GAGATAGGCCGGTCTGCGTCGCATGAAGGATAGCAGCTGAAATCCGGACAAACAGTTGGA

CCAGGGTATTCGCGTACGATAGGTGTAAATATGCCTGGTCTCAGAAGCAGGCAAAGTTCT

TGCTTCGGGATACCTCGGCTGCCTTATAGGCACATGTAATTTACTTACGATAGATATTTT

TCTTATTCCAGAACACATGCAAAAAAAAAAA

>178

CTTTGCAGCAGATACGATGGCCTGAAAGGAGCCATTGGAGGATGGATGCACCAGGCTGAG

AACGAACCTGCCGATGTTTCCTTGCTTGTATTTATTTACAATAACAATTTTATGTACCAT

GTGCGTTGAAATTGAATGAGATCCACTCACTTGTGTGTGGACGATATTGAACATTTTCAT

GTAAATTCGTGGTTTCATTCAAAAAAAAAAA

>179

TTGAACATTTTCACGTAAATTAGGGGTTTCCTTTCACCCACGCCCGTGTACATTAAAAAA

AAAAA

>180

CTCGTCGTTCGCCTCGTTCCCAAATGAGTTCAGATACATTAGCTTGCAAGCTGTCATTGC

GGTGGAAGATGTGCTTGCAAGCAAGCTGTCATGGCGGTGCAAGATGTGCTTGCAAAGCAT

AGAAGTAAATGCAATTGCTATGTTTATGTTTGTGTTTGTGTGATGTTGCGGTGTTGATGT

GACTTACGTATAGACAATGCAAAAAAAAAAA

>181

GCGATGTGGGGGCCCAACACCATTAGGGTCAAGTACCTAGTGTGAACCCATCCCGGGCGC

TTGCACAGAACGTTAACTAGCATCGCTATCGCAGCGTCTCCATGAAGGTCAAGTATATAG

CGGTGGAACTTAGACTTGATACTTTAGTGATTGTTACCTTATTAATTTTCGAAACACTTG

CCATCTTGAATACTCAACTTAAAAAAAAAAA

>182

CATGAGGCCATTACGCAAGGTGAGCAGGAGAGATCACTCGCTGTGTGGAAGATTGTGAAT

CAATGGCAGGCAACACAACTTGCACCAGTGTGAGTATTTTTTTTGGAGTTGCATGGCACC

CTAGGTAGGGAACACAATTTTTTGGACATGCGATAATCCAACATTAAATTAACAACATAC

AGTGATTTGTGGGTCAGGGGAAAAAAAAAAA

>183

ACAGGCGCTCTGGCAACACTTGTACTTGCTTGTAACGCCAAACCGTTTCAGATTTTTTAG

AGCAAATGGAAGGCCATAACAAAATGACATACATCAACAGCATTCATAGCATGTTCTTGC

ACTTTTTGCTCGAAGTTGGATGAATACAACACGTAGGTCTTTCATTTGGATGGAATATAT

GCAGGGAGTGTTTGTTCTCTAAAAAAAAAAA

>184

TGAGGTTTAAGTCTGTCTGGTAATGCTAGCTTGTTTCAGGAACAAACGACACCGTAATTT

AAATCCAAGCACTGATTTTGCAATTTGCCAAAAAAAAAAAAAAAAAAATTAGTCGCAGCA

TTCACAAGGACACTAAGCACGGAGAATGTTTTTTTAAGATTGGAGTATTATCGGACCTAC

TCAAAAGTTAGAACAACTCTAAAAAAAAAAA

>185

AGTAGATATTGGATACTTGCGCTTGTATTCAGATAGGACAGGGGATCTCTCGCGCATAGG

GTAGTCTACGTGCGATACACATTGGGTTCTCATGAAGGAGACCTCATTGTCTGATCCTTG

AATGATTATTTGTTTTGGTACTGTTGGAGTTTTTCGATTTGTAGTCATTTGGTTATGGAC

ATCAAGCTTACAATTGCGTCAAAAAAAAAAA

>186

ACATGCACCAAGAGGACGATACAACCGACTAATGTTGTGATCGCAGTCAATCAAGAATCA

TCCTTTGATTTCTACAGCACGTTGTATAAACAGCCGCTAGTCACCGAGTGTTTTTGGTAA

ACGATGGTTGGTTGTTGTCAAAGCATGATAAGACAGATATAAGAGTAGCCACATGCATGT

TACAGTATACGCTTGAAGTGAAAAAAAAAAA

>187

ATAAAGTACACTAGATGTACTATGTCTCTGTAACGAACAAAAGTACGAAAAGTTTGTGGT

GATGAGAATAGTAGCTTTTCATTCGTCACACAGGTCTCTTACTTGCACTCGAGTACATTT

CGGATCTCCCTGATAATCTGTGTATAGTTATGTGTCATTTACAGTCAAGATTTTATTTAT

TAACGGATAGAAGTCCAATTAAAAAAAAAAA

>188

CGTGTTGCGTCCGTGATTGCATGCCCCTTTTCCAGCACATGATGGTTCTCATGGTATGTG

ACTCGCGCTGAAACTGTTTCATGACCGCGGCGAGTGCACCATAATCTCAAGTATAAGCTA

TAAGCTGATTCGACTGGTGCATTTGATTTCGTAATAATGGTGGTATCTGCGACTTGAAGC

CGGCGTGTCTAGCATTTTCTAAAAAAAAAAA

>189

GCCATTTCAGCATGGAATATGCTGTTGTTGTATCGACAGGAAATCGTGTTTGCTTGCTGC

ATCGCAAGTAGACTGCAAGTTTTGCCTATTGAACATAGTTCAAGTTTTTGCCAAGTTTCA

ATGCAATGTAGATATCAGCCTGTACAGGTGAACTGACTGTTGAAGATACTAGCAAGATAA

TGGGACTTCGCGTTGCCAGTAAAAAAAAAAA

>190

ATGCGACGGGGGGGCACAAAGGAAGCGAGCCTCCAAACAAGGTCTGCGCAAACAATGCAA

CATTCAAACTACTCTACACTGTTGGTGATAGGTTTGTTGGACCATGAAATGCACATCTAG

TCATAAATGCCGCCTGTATCTACAAGCACAAGGCCCGCAAGAGTTTGGTCATTAATCAAT

GCAACTGCGAAGATTCACGCAAAAAAAAAAA

>191

GCATGCCTAGCACAAGCTTTCGTTGAGGAGCGCATCATGACGAGGAAAGACTTGAGGGTG

TCGGTAGATTTCGAATTTTGATGAAAATATAAATGGACACATCAGTCCAATAGATATAGT

GACCAGCATGTGTTTACATGCATCTGTGTTTGTACAGTGCCAGGAAATGAATTGTGGAGG

TAGAATTTAGCAATCTAGTGAAAAAAAAAAA

>192

ACCTTTGCTGCTGAGCTCATAGACATTATGACATCCGATGGCATTGAGTAGCGATGAGCA

AGGCTTAGATAGCAGCGAATTGGTTGCACATGCTGGATATGTTTTACGAACTGCACTGGT

AACCAATATGCATGTTTGGTTTATGCTGGACGACCCTTTCTAAAAAAAATATTGTACATT

GTATGTACATGCATGTATCTAAAAAAAAAAA

>193

TAGCAGCATGCTCTTTATTGGTATCATTGTGTATTGGTTTGTCATATATTGTGTGCAACG

AAGTAGTACTACGACGCATCAAATAATGCCGGTCAAGTATCGTGATTCCACGTGATTGAG

CCCTCATTTTGGTACATGAACTTGGTTTTTAGCAAGACTTTCGTATATTTGGCCAATGAC

AACTCATATCACTTGGCATTAAAAAAAAAAA

>194

TCCGAGAGGACAGTGCAAAGATCTTGACCAAACACATATACCATCGAGGACAACCCATTG

GAGTCTTGTTGGCACAGCTGAAGGTGATAGAAGTATCTATGAGGAAACAACCAAGTCAAT

CTAGCAAATCATGGTTCGGATATGCAGACTGTAAGTGTTTATTTATAGAACGTTGACAGT

TCGGTCGTATCAGGGAAGACAAAAAAAAAAA

>195

AAAGCTCTCCTCGCTTGCGGGATTGCTGTTGCGTCCGCAGTGCAATTAGGAAGTTTCTAG

TATCGGTACCTAGATTGGTCGAATCGGCTTCCTACCGAATTATCAAACGATGTAACCTGG

AGTATAGCGATCCTCATCCATGCTTCTGCTGAGAATGTGTGGCTGCATTTGCCACTAGCA

GACATCTTAGCGCTCGGTTTAAAAAAAAAAA

>196

TTGGATAGAAGCAGAATTATTATACATCTGTGCTTGTGGGTTTGTGTAGTTTGGCTGACA

AAGAGCTTGTTCGCAACTTGCATGAGCTTGTTGTAACAAATGGTCATATGCAAGGCACGT

AAAAAAAAAAAAGTAGCATATGCATGTGACACTTTGAGCAGCTAAGGATCGCAACAAAAG

GCATGATACACTGGTTTTTGAAAAAAAAAAA

>197

ATGATTTGCAGAAGAAGTTGGTGCCAGCAAGTCCCATTGAATTCGCCTGAATTGCTTGCA

ATGAAGTTCGGAATGGATCATGCATAGACAGGCTACATGGCCCCAGGAGGGCTACATGAT

TGAACAACCATTGTGTATGAATTCAGTATGCTGTCATGTGAAAAAAAAAAAAAAAAAAAT

CGTAGTCGCAGCAATCACAGAAAAAAAAAAA

>198

CCTTAATGTCGAATTATGAAGTCTACTTATCGACTAGTTCGCTTTAATGGAATGCATGAA

CCCTACAACAAGCGACAACCAGATTAAAGGAGGATTCAATACTCGAGCTTCATGACATTG

TTTCTCTCATTGCCCTTGGGACCTTGCAAATCGCATAATAAATTTGAGCTATGTTATAAG

TGCAGTAGAGCAGCCGCCCTAAAAAAAAAAA

>199

CAAGTAAAATCCTACGGCCCAAGGAGAACATATCTCGCTATCCCCATCGAGACAGTATGC

TGATCTATGGAAATCCATACGAAACACACTCTTTGCGTGTTTCATATGCAGGCGGATGCT

AGCATGTGAGCTCTGCTTGCCGGTGCATCTCCGAGGATTTGGTCACAGTTGTCAATGGTC

ACATCGTGAGAACCGCATAGAAAAAAAAAAA

>200

CGGCAATGTTTGTCACCAGGAGTCTGGTCATGATTCATCAGGCACGATTAGGTTCGAGAC

GATGTGAGATGCACGAGGACTCTCTGTACATGTGTGCACATTACAGAGCGGTAGACTAAG

ATGGTACGATGTACGCTTGTTCAAGTGACAGTACGTGGTTCTCATTGCTAAGGCTGTAAG

TTAAGCGGCTCTGCGCATCTAAAAAAAAAAA

>201

GCCTTCCCCGGTTGCTTCGTGCGCGTTGCTGGCTTTGACAACGTCAAGCAGGTCCAGTGC

GCATCATTCATTGTGCACCGCCCCCCTGGATACGAGGCCATCGCCACCAACCGTCGCTCC

GTGTAGCTGATGTACCAGTGTGGCATTGTTTTAGCTTATGGTGTGTAAGTTATTCTTGAA

TGGATGATTATTCTGTTGTCAAAAAAAAAAA

>202

TGCGCTTCACGAAAAAAAAAAAAAAAAAAAATGCGCACGTGCATAATTATAATATGAAAA

AAAACTGTGCAATATCCATCTATGAATCGACATTTTGTCTAAAAATATTCCGAACCCTAA

AAAAAAACAAAATAACAACAAATTTAACAGCCAATGATGCTCATATACCAACAACGGCTA

CCGATTGAGGCCGAATTTTCAAAAAAAAAAA

>203

AAACTACAAGAAAGGAGGCCAAAGCTTCTCAGATTAAGCCAGCTGTCAAGGGTGCAGAGA

AGAGGACCCAAGACTGGAAAATCAAAAACAAACTCACTCGGAGTCCCGGTACGTCCGGCT

GGGGCGACGGAGGAGGCGGTAAAACCTATTGCGAAGGTGAACCAAAACGCTCAGCTGTCT

GGGCAGGAGAAACGACATATAAAAAAAAAAA

>204

GAATATATGCATAGACGAGGGTGTCATGTAGGCTCGCATCTGCTCACTCTTTGTGGGCCC

TCACCGCTTCATAGTTTATTGGTTTTATCATTTCGCATCTATTGTGGAATTCTTAGTTAT

ATGACGGACTGATCAAGACAATTTGAAAACAGAGCATGCAAGTTTTGATTTGTGTTGTAA

TGTTGGAAGCATTTCCCCCTAAAAAAAAAAA

>205

GAATCAGAGACAGTGCGCACTTACCACACTCCAACCCTAGCTCTCCTGGAACCTGTAAGG

CGTTCTGTCAACACCTGTATGTAGCATAGTGAGTGCTTGCTCATCACCGCAATGCACTTT

ATCAAGACTGGGCTGATATGGCTTGAGAGCATTGGATTCCAAAAAAAATACTAGCATGTT

TATATCAATTGTTACTTTCCAAAAAAAAAAA

>206

ATAACAGCTCAACCATCTAGCATAGTGTGGTTGCTGCCATCACAGACTGTATGGCAAAAC

CTGCAATGATTAGGCTGGCGGGTTTATGTGACATTTTGTGACATGAAACGATCATCTCAA

TTACATTTGTGTATCTCATATTATGATAGCCATCGTATTAAAAAAAAAAAAAAAACCTGT

CATGCGTGGCATGACGATGCAAAAAAAAAAA

>207

TGACAGTTGTGAGTGGTGTGCCTTTTACTGTGTCATCGGCTATTTAGAACAATCAAGGAG

GCCATTTGTGGTGCATGCAATCGACAGGTTGCAGGTTTTTGACAGTGTGGTTGGAATGTG

CATTCGCAAACCATGTCGTACCAGAGACTATTGTGTATGTTCAATGAGTGTTTGATATAT

TGAGTAAGCAAAAATTAATGAAAAAAAAAAA

>208

CTGACGTAGCACAATGAAAATTCACTTCAGAATCCAATTGGGTTGATCGAGCCATAAGTT

GGATCCTGGATTTTTACTTTCGGCATGTCTGATTCTTGATCCTTGTCATGGCTGTATCTT

TTATGGTGGTACTTGAAACCGAAGATTTACTGATAGCATTCAAACCCAATGTTTATGGAA

AAGACACATGATTGTTTTTTAAAAAAAAAAA

>209

GTTGTGTACGCAGAGAGTACTTGTACATCTGCTATCGGTACGAACACATCATGTCTGTCC

CAGAATGGATGTGAGCACTGGATGCATCGCAATTCCATAGCACAACCATTCATTCCGTTG

ATTAGGGACATTTATACTGCGTCATCACTCGATGTGGATGAACGATGTGCATATGTGATA

CTTTTCGTCTTTGAAACGCTAAAAAAAAAAA

>210

AAGAAGTTGGTAAGTGTTTCTTCATGATGGAATCCCTGATGCAAATACAGGAGAGAGACT

TCATTAGTCGAGAAAGATTATGTTGACTGAGTCGCATGCTTAATTTCGCTTCTTCATTAT

GTTACCTTGAAACGTTGTGTAATTTGTCAAATGCACACGAGGCATGACCTGTAAAAAAAT

CCAAGAAAACGTGTTTCGTCAAAAAAAAAAA

>211

CGGCAATGAGCAGTACTAGCAATGAATATTGGATCGTTGTTTTTCCATCAACAGTTTCAT

GATTTTGACGCATGACGAGCTGCAGACAGGGATATACCGTGGGGAATTCGGAAGACTGTA

GCGCTCCCTTGGAGGTGGATTCAGGGTTTTATAGGGTCAAAACCCATTAAAAACGGGTAA

AGTTTCCAAACCCTTCACCCAAAAAAAAAAA

>212

TTGCCCTTTTTGGTTACTTTTTTCTTTTTTTTTGGGGGGGCCGGGCCCCGCCCCCCGAAG

GCCTTGAACGCTTTGAAGGAAATTTGTCAAGGGGGGGGGGTTTCCGAAACTGAAAATTTT

TTGGGGGGTGGAACCGCCAATGCCCTTCGGGGGGCAATTTCCGTTTTTTTTTCCGGGTTT

TTGCCCATAGTTTTGTTGTTAAAAAAAAAAA

>213

AGTAGGCTGTTGAGCTGCAGTGATCCCCGAAGGTGCCGACGATAAATGAAGTTTGCCAAG

TATCCTGCTGGCTTTGCAGCAGATTCGATGGCCTTAAAGGAGCCATTGGAGGATGGATGG

ACCAGGCTGAGAAATAAACTGCCAATGTTTTTTTGCTTGTATTTATTTACAATAACACTT

TTATGTACCATGTTCGTTGGAAAAAAAAAAA

>214

GGGTGCTAGCGGGGGGGGTGATTGTCCAGGGTTGGGGTTATTTTTTTTTTGGGGCCCCCC

GGGGGTTTGGGAACCCTTGTTCTTGGTTGTAACGCCCAACCGTTTTCGGTTTTTTTGGGC

AAATAGAAGGCCCCGGCCCTTTGGCCTACCTCCAGGTAAGTTTTTGCCTGTTTTTGCCCC

TTTTGCTTGGAGGGGGGGGGAAAAAAAAAAA

>215

CTCAAAACACCTTTTGCCCGAGACAAAAAAAAAAAAAGTTCACTGGTTTTCCTCTGTGCT

CAAAACACCAGCTATTGCTGGGAACACACAGGAGCCCGTTAATCCATGATCTCTTCGTTC

ACATTGTACTCACCACACATGTAGCTGTACACATGTAACATATGTATGCAGTAATGTTTG

CTGGGCTATCTCTGTACCTTAAAAAAAAAAA

>216

TTGGAGGGGTTTGGGGGGTTTCCTTTTTTTGGTTTTTTCCTTTTAGGGGCCCCTGTTCCA

GGTTTTTGGTTTTTCCAGCCCCCCCCTTGTGGGTTTTTGGGGGGGTTCCCCTTCGGGGGG

GAGGGGGTTGGCCCCTTTGGGGGTTAAAAAGGCCTTGGGCCTTTTTTTTTGGTTTTTTTT

GGGGTTGGGGTTTTGGCCCCAAAAAAAAAAA

>217

AGCGCCTTGTAGAACTTGTTGTACTCCTCTTCCTCAACATCACCCGCTGACCGTAGCCAA

AGCGCTTTGTTGTCATTCAAAAGCTTCCACTCCCACACGGTTTCTTTAACTGTCTTTGTC

TGGGGCTCATCCTCTGCCTCCTCATCCTCATCTTCAACATCTTCATCTTCGTCATCTTCA

CTATCTTCTTCGTCCTCCTCAAAAAAAAAAA

>218

GCCACGCAGAGTCAGCAGACCGGAGTGTTGATGACCTACATAAGCTTCGTGAAGCCGTGC

CTGAACTGTGGCAATAGTTGATTGCAGGGATTCGATGAATCGTTTAAGACATGCGCAAAC

AGTTGTTCTCTGACAATTCTTTCAGAAACAGGTGATTTCATGGCGCGCTGTTAGTTTTTA

TTTATTTGGTATCTTTCTCGAAAAAAAAAAA

>219

ACTCGCCATGAAACAAGTGTTGTCATTGTACACAGGATTGTGCACAACTGAGAGACAGTT

TAACAGTAGGGGTCACCACAACCCTTTCCATCAAGCGCCAAGCAAACCAACGTAGGACTA

TTGTTGTACTGTTGATGAAACGAGTAGTGCGGGGATGATCTCCTATAAAATTATCAATAT

CAAATTTGCTAGTTTTCCCCAAAAAAAAAAA

>220

GAGATGGAATCGTGGAAGAGCACCCGCGACATCCTACTATCCACGCGTTGCAAATACTTC

AAGCTATTTAGAGAAGTTATGCGGTCTAAGGTGCATGGTTTTCATCAAATCTGCATGAAC

GTCGTGTTCAAATAGTATCCAGCTTGGAACATTATTTAGCGAATCCCAGGATTGAATTTT

AACAGAAAATGCATTCAACCAAAAAAAAAAA

>221

AAGGGAAGGGTGAAAACACAATGGTAGGAGTAGCATCGCTGTTCCGCGTCCACACCTTCG

CATCACTTTTTGATTTTTAGGAACGAAGTATCCAGATCGCATGCCAACGAGCTAGGCAAC

TAATAGTTTTGCTTGCCTTCACACTAATACGCAAATCTGACACCCATGATGGTTGCTTAA

GATAAGAGAATTCTTGTTGTAAAAAAAAAAA

>222

ACCTCCTTTGCCACTTAGCAGTCATTTAGGGGCCTTAACTGGTGATCTGGGCTGTTTCCC

TCTTGACAATGAAGCTTATCCCCCACTGTCTCACTGGTTAGTTGAAGACATGTCTAGTAT

TCTGAGTTTGCTACGATTTGGTGCCAGTTTCCCAGTTAAGGCCCCTGCCGAAGCTGTAAA

ATTAACTTAAAAACACTTTTAAAAAAAAAAA

>223

CTATGAATGCGATACGTGCTTATCAGTCTTACTTGTAGTGTAGTTTCACAATCATGTTTT

GTTTGGGTCTAAGATCATTCATTGTGAGTGTGATTGAAGTTTCCCAGATGAGTGTATGGG

TTTTTTAGCATGCTGGAGTATTTTCATGTACACACACATGTAAGTGCACATCTATTTATA

AACAGACACACACACAGCTCAAAAAAAAAAA

>224

GATTTCCAACTCATGAATCATAGGAGAGAAACAAAAAAGCACCTGGAGGATGCAAACCAG

CTTGCGAGAGGACAGGACGAGTGTATCCCAAACAAATTATATTGAGTGGTGAAGACGGCA

CCGCAACATTGGCTCAGTACAAATTAAAAAGCATGTGAGAACAATGTCGTTGCTCGGCAT

GTGAACCAGTTGACAAACCGAAAAAAAAAAA

>225

TTTTGCAGTTGACCAACTTAAGGTAATTTTCAAAACGCCATTTGAGCCTAGAGTGCCGAG

ACAGACATTCAAATGAGTGGTGTAGACGCGGTGCACTTCGGCCAAATGAAGGTCCGTTCC

AATAAACTTTTAACATAACAGTTCCAATAAAAAATAACATAAACTAATGGGCTTAATATG

ATAATTCACTTTTTTCATTTAAAAAAAAAAA

>226

TTCCAGAGACATAGTCATATTTTCAAACATTTTAAGGTGACAAGACATCGCGAGATGTTT

CGTGCAGAAAACACTAGAGAATGGACATGCATCAATTCATTTTATGGTTAATGCCGGATC

ACGCGATTGATTTTAACTTACATTCTGTAACGGTATTGTGACATTTGATGAGAGAGTATC

TACGATGTTCGCAATAAGTCAAAAAAAAAAA

>227

ACCACCAACTTTTCTGTAGGACCAAGCGTTGCTCCACCACCCCCAGATGATCTTACCAGG

GCCACACGACGTTCAAGTCTGCCTTCTTTTTCCATGCATTCCCCATCTTGATCGTCTGTT

GCAGATGTTGTTGAGCCTAACATGCGCGCGAAAACTCTCACTGCGGAAGCCTCCCTAAGT

CTCGATATGTTAGGCTGGAGAAAAAAAAAAA

>228

CAAAAAAAAATTGGAGGATGAAGCCTCTAAAGAGGAAGAATCAAAGGACGAGCTTAGAGA

TCAATTCATGATATTGGAGTGCATAGATTTTTCTCCATAAATGGAGTTAATTAGGGGGGG

GGGATGGTAATCATTTGAGTAGGACCCAAGTTCATGAAACTGTGGCAACTAGATTGTAAA

TTGTTACAATCCAGGTCGAGAAAAAAAAAAA

>229

GTCGATGATTCATTTTCAAAATATTTGTGATGACAAGCTTGAACCCCCTGCATGTTGGTT

GATGAAAAAAAAAAA

>230

TGATATTAGTTATTTAGGGACATATCACCAAAATGAAAGCTATGCAGGCAAGTGCTTATC

GAGGGAAACCAACATGCAAATGTCGGGAACATTTTTTTCTTTCTGTCCTGCACAAACCAA

CAAAGTCAAACCTTTATTTGCACATTTTCCAACGGGCCGACCAAAAAAAAAAA

>231

ATGAGTACTCTGTTAGTATTTATATTATATAAATACTGATAAGGTCAGGGCAAGACTAGC

CGTTTAATAGATATCAAGTGGAAGGCGAGTAATCATTCCAGCTGAGATATACTGACAGAC

CGAGAAGTTAGACTTTGTGCATTTTAAAAAAAAAAA

>232

GGTTCATATGCAGTAGATTACAGGCACTTGTGTATACTCAATGGGATATGACCTCTATCG

GTTGGGCGGGAAGCCACATCAACTAATAACACAGCCGAATTCCATTACCTCGCGATATCA

CTCCAAGGAACAGTGCACCAAGCCTGCCACATATCTACAGATGCAAAACGTACATCCTGT

AAAATACGACCCAAAGGATCAAAAAAAAAAA

>233

TCATTCCACCAAAGTAAATAGTATGTCATCGCCACAAAAGATAAAGCGCGAGAAAGGGTT

TTTTCTAAAAAAAAAAA

>234

CCTTCCCAACACAGAGTTGATCTTCGTTTGTTGTTCTCAAGAATGATGGAATTGCTGCTG

CAGAAATTATTGGAAACTAGGAGTTGTTGACAATCGATCCGCAGGATAGTGATGCTGATC

ATAGAAGGCGAAATGTTTACTAGGTGAAAGCAGCATGACGTTCATCACATCAAGTGTAAT

GAAATTGACAGGCTACTGATAAAAAAAAAAA

>235

CTGCCCCTGTTCAGCATGTAGATAGGCGAAGTAGTAAGAAACCCCTTTTTTAAGTAGACA

GCATCCCTGCCCGTGAGGGTTGTCGTTTCCCGAGTTTTGCTTAAGGCCCCGGGCTAGGGA

TCCCTGGGGGGCTTTCATTTAATTGGAAAGGTAGTTTGGCCGTCGTTAGTTTTTTTAACT

TTATCAGTTGCTTTTGGGGGAAAAAAAAAAA

>236

TCGCCAGTGCGCCCGGCAGCGCTGGTAAGGTTGTACTGTTACAATCCCTAAATGTGAAAC

ACTTTCGGATCGGGTGAGAAGAACCAACTTCGTATTTGCAAGATCGCCATAAGATTCTGC

GTGAGGTAAAGACTGTGCGTTCGCTTATTGATATAGCATGCCACAATTTATTGTGTTGTT

ATGATGTATGCCAAGGCAAGAAAAAAAAAAA

>237

CTCAAAATGCTCAATGTGGTGGCAAATGCGAATGCTTTGTGCAAAGCACATCATGTAACA

GATAGGACAGACTGAGTAGACTTGTACCAGGTCTCTTTGCTTACCTGCCCTAGAAAGCCG

CTGCACTCGCATGGCTGAATAAGTTTACCATCACCAGCTCCGCTCTGATGTTACCGCCAT

TATACAACACAACTCAAATCAAAAAAAAAAA

>238

GCCAAGCGCTATCTCAAGTACCCCACCAAAAAGTACCTTTCAGGGTTTTCTATGCTCTAG

AGGTGCAGCCTCAGCCGTGGAGTGTGGGAGGGGCATCCCCTGCTTCCAACAAATTAGCTG

GTTGCGCCGTCATACGCCTGCAGCGAATTCGAACTCATTTGACTAAATATATTCCTGCTG

TAGTGTGAAGATTTTACTGTAAAAAAAAAAA

>239

CTGAGAGATGTCCTTGTTATGATTTCGTACTCATAGCTGATTGAGTGTAGGTTGTGTATG

AACTAGAATGTATTTTTCGCACATGATCGCAGTGGATTCCCATAGAATGATATCCCTTAG

GCAGCATTCATGCAAGACTATCGGGAAAAAACCGAATCAGTTCACTTATTGTCAAGAATC

TGTCGTCACATCGATGAATTAAAAAAAAAAA

>240

GGGATTGCCTTTTGTTCGCTCAGAACACTTAGGAACATCACTATCGCATTGTATTTCGAA

AACTGAGGATCAAGTTGGCTGGACCTGTAGACAAAGCTGAAACATGGACATCTGATTTTG

TTTTGTCAGGTGGGTTGTTGTTTCAATGATGTGGAACTGCGTATCTGCGTTCAATATTCC

ATACATATGTGTGAATGATCAAAAAAAAAAA

>241

CATTAGCAGTGTAATCAATATCGATTTGTCGATTCAATTGTTTGCCGAGTCTTTGCATTC

ATCTTAACTATTTTGCAAGACGGTTGCCATTGGACACGAACCACCTTCACAGGGATAGCC

ATGCTAGCCACTAGTCTAGAAATACAATGCACGGTGTCAGCGCGCTTTTTTGTTGAGCAT

GAAATTCCCTTGGTTTTAGTAAAAAAAAAAA

>242

CACCCGTCGCGAGCTTCGCAGCGCAACCTGGGGGCATGATCGGCTCCGTACGGCAGTCCT

CCGACGTTCCCCTATGGAACACCATGCTGTCAAGTTATTACAGGACAAGCTTTGCGCGAC

GCGTGCGTACTGGCGGACTGCAGGCATTGAGCTTTTGCCAATCGCCGGACCACCTGCGAA

CAGCATGCGCGTGTCGCAGCAAAAAAAAAAA

>243

CGAAACAGATGAAATGCTGTTCCATATAGCTTGACGCTCGAAGTTGATTGATAGTAGATG

CACTTACGATGGGTTGTTTTGATAAACAGCCTAGGTAGTGCCTGTCATCAGTTGCATAAT

TCCTGCCTAATATCAATATCGTCGTTGTCGGGATATGTATGGTGTTGGTTATGTATAAGC

TGGAAATTATCATGGAAGTGAAAAAAAAAAA

>244

ATTCATATCAGACATCATTGCGCTATGCGATTCTTTATAGCTAGTGTAACATACCATACA

TACGATGACCAGCCCTTCTTGAACTCGTTAAAATGTCTTTTGTTGGGAATTAAGGATGGT

TCATATTTGTGTTACCAAAAACATGGTGTGCATGGTATTTAAGCGTATGATTAACATTCA

TTCGCATACATCATCCATGTAAAAAAAAAAA

>245

GGAGATCAGGCTCAGAATCACTCTCGGCAGGTTAGCTGGGGTTTGCTGTCAGTGATGTTG

CAAAAATATTCATGCTCGTGGAACTTGAATACTAGCTAGTGGCATGGTTGACAATCAGGG

ATGCCAGTTTGAATTTTCCAACCAGGCGGTAGCCATTAACAACGCATCTGCTTTGACAGG

TGTATTGCAAATAACTTAATAAAAAAAAAAA

>246

GTGCATGCCTGCTTCGAAACGAACAGATGGAGAATGTGCGAGCTTGTTATTCGATCAATA

CTCAATCCCCAGTCTCCACGTTCAGCTGAACGTAAACGTAGTTTTGAATTGAACACCGGT

TAAAGAGGGGGTGTGATATTCTCAAGGACCCAGAGTTATTGTAATGTAGATAAGGAATGT

ATTACGAATAGCTGCTGGCCAAAAAAAAAAA

>247

GTACGTGTGTTTCCAAACACTCCTTGACGTTTCTTCTCTCATTTTCCTCTCATTGAGATT

TCCATAAAATTTTGGATCCATTTCGCATTTGTGTCTGGATAGGAAAATTCGTTAGGCTTT

CTCAAATATCGTTACCAAAATAGAATGATCCAGGACATAGACAACCAGGCAAGGTGTACA

TTAAGGCTTATTTGGTCTGTAAAAAAAAAAA

>248

TCGCGATCCGCGCTGGTGCCTTCACTGAAGCGTCGGCCGCCGATGGCGTCAATCTCGTCC

ATGAAGATGATGCAGGGCTGATGGTCGCGTGCGTAGCCAAACATCTCACGGATCAGCCGG

GCGCTCTCTCCAATGTACTTGTCCACTATCGCACTTGCTACCACCTGGCACAACAACTCA

TACACAGCGCATTCACACAGAAAAAAAAAAA

>249

CACCAAGCACCCCCAATTTTGGGAGGGGGAAGATCCCCTTTTCGGGCGTTAGGCCAGATT

TTTATGGATGGTATGTTTTTTGTGGTCCCAATTGGGGATGGTTTCTTCCAAAAAAAAAAA

>250

CTTGTACTGGTGAGCAGCTTTGCTGGATGAAGGTAGTCGGAAGGAGCTTGCACGATGGGA

AAGCTACAGATCACCACCCTTTCCCCTGTATACAGAGTGGTGTGCATCAGGCCTTGTTTG

TTTTTGCGATGAACAGTGTACTTCAATGCCAGGCAAGTGTATTCAGGATACTTTAACCAA

GCTATCAAAGGACTGATCTGAAAAAAAAAAA

>251

AAAGATGCAAAAAACAGGATCTCGCAGCTAGCAAGCTCCACCTTAGCATCAGGTTTCTCA

GGGGTATCTTCTATGTTTGCTGGAAATTGATAGACACTGTCTTAATTGTGGTTGAGCCAA

ACGTGCAACTGCTGATGGAGTCGAACAATGTTCACGTCTTGGTGCTACCCAAATTGCTTT

CAGTGAGAAGTGCTATCCTGAAAAAAAAAAA

>252

TACAACTCAGTTCACCCGCGTTCACCTATTGCAGCTGATCCAGTCGATTGTGATGTGACA

GTTGAGAGAGTTCCATGAGTTGGGATGAAGCAAGGATTCCATTATATGTTGTTGATAATC

CACGCATAGTAAGCTGAATCAAGAAGTATTATGTGATATTCAGTGTCTGCAATTAAGAAG

TATTTTTATGTGTTGGACCCAAAAAAAAAAA

>253

GCTTTGAGGGGACTTTAGCCGAGCTCAAAGACGCGCTAGTCTGCTTCACCGCGCTAGAGT

AGCAGAAGGTGAATGGGCACCATAACCTGTGAAGATGTAGAAGGTTCAGAAGTAAAGGCA

GAGACTTAAGTGATGTAAAGACTTGCGTTACGCCTTTTATAGTGGCGGCTGTATGATCAG

TACTAATCTAGAAGTTTCTGAAAAAAAAAAA

>254

CGGAACCAATAATGCATACTGTCCGCCCTTTGAATGCATGCTATTCATTACGGTGTCCAG

TACTTGCTCAGCGGCTTGGATCTCATGTGGCAAGATGGAATGTGGAAGGTCAAGAGTTTG

CAGCAATTGCTCAGTTACTGCTCCCTGAGCAATGTGTTGAGGCTCATCTTTACGACCAAG

GCTCTCCGCGCCAACCGATTAAAAAAAAAAA

>255

TGCAGCCCACGTTACGTTGCGATCGCGGCCATGATTTTATCATCGACCTTCGGTTTGTCC

ATTTTTTCGCACAGGCAACGCGATGAACACATCCACCTCCATGATCCATATGCACCAGCA

TGAAGTTGCACATCCTTGTCGGTGATTCAGAGCGTGAACCTGAAACTGACACGGGTGTCA

ACAGAACTTGCGAGTTGACGAAAAAAAAAAA

>256

GCAATAGGTATAGGCAGGGACTACACAATATCATAGTGGCTCTTAATCCGTTAGTGGTGT

TGTCCTTCAGTAAAAAAAAAAAAAAAATCTTATAGTGAATTTGTGCATTCACAGGTATTG

TGCTGCAACCACTTAATTGCACACAAATATACGCAAGCTTCGAAGTCGAGTTTAAAAGTG

ATCATTGGGTTGTAGACNAGAAAAAAAAAAA

>257

CGTGATCATAGATGCAGTGGCTCAAACTGCGTTAGAACGAAGCATTGCTAGTAAATGCCA

ATGTCCTTATGCACCGTTGGTAAAGGGTGTCGGACTTGCAGTATCGCATGACTGACTGCA

GTTCCATTTTTATGACTGGTTTCTATCACTCTGGTACATTATCTTGATGTTAATAGTTTT

TAATACATTGCATTACCGTCAAAAAAAAAAA

>258

GAAGGAATGCGTGAAACACCTGTTTGGGGAATATGGTGGTGTATGTGAAGTTGTGTTGGA

CACTTCCCTTCCTGGGGAAGCCTAGAAAGAGGACTCCTTTCTTTAGGGTTTAATGAGCTG

CTTAGGTAATTAGGATCGTTGGACAGATGGTGCTAAGAATTTGATGTAAAATTGTGGTAT

TGCATATATACTTGCCATATAAAAAAAAAAA

>259

GACAACTCATTTGTGGTTTGCGCAACACTGTCAATGCAATCAAATGCCCAAGTTGTTGTA

GTTGTGTGATATCCGCCTTATGATGATGGATGGGTGGGGACGTTGTGAATCAAAGTCTTA

TGCTGAGCGCGACTTGGACAGAATGAGAAAGTAGAGAACATGCCAAGTAAACATTTCAGA

TGTTCAAAGATACATCATGCAAAAAAAAAAA

>260

AAGTTTAGGGACAAGATCCCTCCCTTTGCGTTGGAAAGGGTGTCAATGAATCACGCGCAA

AAATCAACCCCTGCATTTTTGGGTCATGGTATTTGTGTACCCCATCAAACACAAACAACC

TTGAATCCAAATGGGGGCATATTACTATCGTTAAAAGAGTTCGGTAATTTTACCCCCCCG

CAATAATACAAAGCCGGTGGAAAAAAAAAAA

>261

ATTTTGTGTCAATTGTCCTTGAAATCTTTCTGACCTTTTCATTCATGGTGCACAATACGA

GTGCAATGGTGTACGATGCAGTGACATGGTATTCAATATATGAGACATGTGTTAGCGCAT

ATCGCATAGTAAATCTTACATAATGCACAATAGTTTTCAGCTAGGTATCCAAGACCACTT

TATTTAAAATTTGCCCTACCAAAAAAAAAAA

>262

CACCATGCCAGCGTTCAGGGCACAGGATCAGAGGCTTTAATGTTTTTCAAAGCCTCGAAC

TGCTGGGCGGGCATTGGAGGATTCGCCTCCAACTGGGCATGGTCAAGAAGCCCGTTCGGG

GGAAGTTTCTTTTCCATTTTTTGTTCGTCCGGGGCCACCAGCAGAATAGTTCCAGGCCCC

AGCGTTGTCCCCGGCGGACCAAAAAAAAAAA

>263

GACGTACGTATTGGGTGCAGTTTGAATGCTTTTATCGATATCAGTTCATTTGCACTATTT

TGAATGGTTGTGCAGTATCTAACTCAGTGCACCAACGTTCTCTAGACAGTGTCTGCGCAA

CAATCTTGAAACGAACAAAAATGGTATTGGTATTCATACTAATAACTTGGAATAAAAAAA

AAAAAAACGTTGTTAGCGTTAAAAAAAAAAA

>264

AAATTTCCACGACGCCGCATCCGCATCATTCCCTGTTTGCAGGCATTCGCACAACACGGT

GGTATGCAACTGATCAGATTTTCTCGTTGTCATTGCTGCCTGCCTGACGGCTCAAACCCA

GCGACTTAGTGCACAAGCCACCAAGAAAACTTGGCGGAGTGCTGGGGTGGCCGTTACGCC

ACCTTCTATTCGTATCTCCCAAAAAAAAAAA

>265

AAACTCTGTTCAGCCAGCAGATGTAACTATGGATGCATCTGTTGCAGATCCATAATCACT

GTCAACCGTCTTGATAGTGGAACGACGAGAATAGCCCAAGCCCTGCTTGGTAGTTGTTGT

GCAATGCACGTGTCAGCAACAGGGATCGTCTCGTTGGCTTCACTAGTTTTCTCTTATATA

TAAAGCACTCTTTGCGCATTAAAAAAAAAAA

>266

GCCCGTGAAGCTATCAACCTGAAGTGAAAATCTTGATGAACAGAATCCTTGAAAAAAAAA

AAAAAAAAAAAATCGTAGTCCCTCTCTTTCCCTTGGAAAATACTGATGATGCTTTGCTTG

GCGTCCGAAACGCTTGGCATGATGCGTGTATGGATTTTCATTCTAACACACGGACGGACA

TACCTAGTACCGTTGCTCGTAAAAAAAAAAA

>267

CGTAAGCTAGTTGTGGGTGTACCAGACAACTAGGCTTGCATTCAGAGACAATACCCAGGT

GGGGCACGAGGTCGGTGGAATCCTCCGTTGAGTCATGGTTCGAGTATTGTGCAAGCCTGG

AATGTCCAAATGGTAAAGTTGACTACGTCCACTCACAAATGTAAGGTAAGTGTATGTAGA

TGGTTGTATACCCAAGCGTTAAAAAAAAAAA

>268

AAACGACTATGTTAATAAAGGGTGTGTGATAGTGATAGATAGTGGTGGGTACTAGTCACT

CATCATGTGTCGATAGACAATGGAATAGAATAGTGATCGATCATATAAGACCATAGGTCG

ATTACTCACTATACAGCCGCCAGTATGAAATGCATAGAGACAGACAATTCCAACACTTGA

AGTATATGTTGTCACCCATGAAAAAAAAAAA

>269

TGGGCCTGGGGCCCGGCTTTGGCCCCGGAAAATGCCTAGTTTGGGGAAAGGGGTTTTCTG

TTTTGGTGGGGGGAAAAAAAAAAA

>270

AAGGAAAGTGCCGAGATTTGGCCTCGTGTGGGGTCCGCCGCAAACTCCATTGTGTAAAAC

TCCTCCCATCCCCTAGCACTTTGAGTTGTTTAGAAAAATTGTTGACCAGGGCCCGCGCCC

CCCCAAGGAGTATAGGACGTAGATGATCCTTAGAAATGATTAATACGTTGAAACTTTTTT

TGATCAATTTGTAGATTGTCAAAAAAAAAAA

>271

GCGCGCACCCGAGAGGATTTCGAAGAGGAGGCCACGGACGATGGGTATGTTAGGCTGTCT

GGCCGTATCCCCTAGCCAAGATCCAGCAATCACGGCTTGCACTGTGGAGCTAGGCAGACA

CTCACATATGCAGTCCCGCCTGTACATCATTCCTTTAAGGCTGCTGTGATCATTGTATAT

AGAATGCAGCATGTTTTACCAAAAAAAAAAA

>272

TGATGAGGTGTGCCCTGCTGGATGGAAGCCTGGAGACAAGACCATGAAGCCTGACCCCTC

AGGATTTAAGGAGTACTTCGCTGCCATTTAAGCAAGTGTAGGATATTAATATCCAACTTA

CAGTGTAAGTGTAAGATGAGTATGTAACTATATTGGAATTCATTCCTGTCCTGGGTTCCC

ATTTGGGCGTTGGTCGGGTGAAAAAAAAAAA

>273

ATGTTGCTGTCTTTCTCCACAGAGAGGGGTAGCTTGCCATTACTAGAATCGTTAGGGCAA

CGCTTCCCGGTTTCCGGTGCTTGTGATAGTTGCTGCATGCTGACACCATTTCCCTCTTGT

AGTCATCTTTAGCGTCCTTAACGATCTTGATATAATTTCATTGGACATACTTGAAGAATA

TTTCAACTATTCAAGTCAGCAAAAAAAAAAA

>274

CAAGAAGATGCGTGTTGAAGATGCTCACCCACATGACACTTTGGAAGGATCGGTAAACTC

ATCACATTTTAGCATGACACTCATGCATATGACATTAAGTGATATGGGTGTTCGTTCACA

GAGTGATGCCCACGAACACACAGCATTACAAATGCTGTATGCTGTGCAACCTTACAATGC

ACAGTTGCTATGCAATCTACAAAAAAAAAAA

>275

CGCCCCCTTTTTGTATGGGGTGGCCAGGGGGGGGGTTTTTCCCTTGTTCAGGTAGAGGAC

CCGGACCCCAAGTGGGGGGGGGGATTCCCCAAAGGGGTATTCGGGTTGGAGGATGGTTTA

AACCCTTTCCCTTTTTTTTGTAAAAACAGGGTTGCCATTTTCCCCTTGGCCCCCAAAAAA

AAAAA

>276

AAAATACAAGGTTGGGAACCTCTGTAGATATTTTGCTGGAAGCCCCCCTAAAAAGTATAG

GGCCCTTGCCTGTATGGGGGCATGAACCATTCAACAGGGCGGGGGATTTCAATTCATGGT

TCAAGTCGGTACATAAGTAGTAACAGCTCCGTCGCTGAAGCAGCTTTGATCAAAGCATTT

TTGTAATCAGCACGTTTTTGAAAAAAAAAAA

>277

GATTGAGAAAGAGGAGACCGATGAAGTAGAACAGGAATTAGGCCGTCCAAAGCGCATGTT

GTTGTACAGGGACTTAGGCTATTCCTCAAACTGATAGAAGGTATCCAGATTCAGACGCAC

CGACGAGTACATGTCAGTATTACTGCTTGGACTTTGATATCCGATCCTGCGGGGTGCATC

TAAGAATATCTATGCTGTGCAAAAAAAAAAA

>278

TTGTACAGTGGGCCATGGCACAATTCCTGGTTAGCTTACCTCTTTCCGTCAACCTCAGGT

ATGTTTTGTGCTGATAGACTAGGAACCTGCACGATAGCATGTGCGAATTGTCTTTCCAAT

GCTTGGCTATTGGTTGACAGTAAAGCCGCAACATTTAGTGTAAAGTTTGGATATTCAGAG

TATATATGGTCAGTCAAAGCAAAAAAAAAAA

>279

ACCTACAGCAACAATATCACCACCCCAAGTAGGGCTTGTACTTGTTAAAGCATTTGGTGC

TGTTAAATTAGGAGCTAAATAATGAGTTTTTTGAACCCATTGAGCAAAGACTGGTTGTAA

TTGGATAGCCCGAAAACATATCTTGAGGTCTTCCTAAAGCTGACATCGTATCATTATGAA

TGTATAAACCAAAACTATGGAAAAAAAAAAA

>280

GATTGTTTTTTTGGGTAAACAAGATCCAAAGGAGGCATCGTGTGTTGTCCCATATCACCA

CCACACACAGGCTGGCAGACTTCACATTCCATGACAGGGAAGTAGTTGGAACAGCTTGAT

GCTCTTTGATGGATCGCCAAAATTTGAGCATGCTACACTGCTGAGTGTTGTACGGTCCCT

GTAAATGGATTTTCAACACCAAAAAAAAAAA

>281

AGTTCCCCTCGATGGCCTCCTTCGGTGTGCGGAAGCCAAGGCCGACGGATTTGTACCACC

GTGATCCGTTCTTTCCGGGAGTCTTCCTGGATATGGCTTTCCCGTGGATTCCACGGATTC

CCTTCTGCTTCTGAAATGCGCGCTCGCTTTGTTCCGCCATCTCGCGCGCGAAGGAAGGTG

GTGTGAGATGCGCCGCCGTGAAAAAAAAAAA

>282

CCGTCTCCGGTATCAAGACATACTACACTTTTTGTAGATGAGTGAGGCAACTGAATTTTT

TGTCTCGCCAATCCAAACACCAATCCTAGTAGACCTTCCTTATGGGTGTGTGAAGATGTG

TGCCATTTTTGATGTAAAAAAAAAAAAAAAGGAACAGTTAATATATTCATTGTGTCTCAA

GTTTTAAGACCAGATCCTATAAAAAAAAAAA

>283

CGAGATTGTTGAACTCAGAGTTTGCAATCTTAAACTTCCTCGGTGATTGTGAAAGTTCAA

TGGAGATTCGCATGGGTCCCGTAAACTGGCAATGAGTCACACCTGTCATGCCACTTCAAT

GTAGAAAACATTTGTGGCCTATCGTGAACCTCATGTTGTGCTCCAGGTATTTCTTGGTTC

GTGCCCATCATATAATCATCAAAAAAAAAAA

>284

ACACAATTACCAAAGCAGACAAGCATCCCCTGCGGTGCACATCCATCTGCACGAAAGCCT

CAGTAAAAAATATGCTAGCGTTCAAATCAAAGCCATGCTATCAGCTATCAGATCAAAGTT

TATTCAAATCTTGAACCAATCATGTAATCATTAAAGAAGTTCCACAACCAAAAATCAAAT

CCGCTTATGAACAAAATGACAAAAAAAAAAA

>285

TGGAACTGGGGCTGGATGTTTGCGGAAACGCAGCCTCTCGTATCTTGGATGGTATGTGTT

TCTCGATGCAGTGATGATCGAAAATGGTCTCTTCGGACCAAGTCGCTTGCGGACGAACGT

ACTCGTAGCGCAATGTTTCGCGGACTATGCATGAGCGAGCAAGAGACGTGCACACGCATA

TCTCCCGTGTATGTCCTGGGAAAAAAAAAAA

>286

GATAAATGAAGTTCGCCAAGTATCCTGCTGTCTTTGCAGCAGATACGATGGCCTGAAAGG

AGCCATTGGAGGATGGATGGACCAGGCTGAGAACTAACCTGCCGATGTTTTCTTGTTTGC

ATTTATTTGCAATAACAATTATATGTACCATATTCGTTGAAATTGAATGAGGTCCACTAT

CTCGTGTGTGGACAATATTGAAAAAAAAAAA

>287

GGGGCTTTCCTTTTTTTTGGGGGGGGGGGTTTTTTGGGGGGGTTTTTTTGGGGTTTTTGG

GGTTTTTTGGGGCCCCTTTTCCCGGGGGGTTTTTTTTCCCCCAGGGCCTTTTTTTTTTGG

GGCCCCCCTCCCTTTCGGGGGGGGGGGGTTTTTTTTCCCTTGTTTGGATTTTTACCCGGG

GGGGGGGGCCCCCGGCCCCCAAAAAAAAAAA

>288

TGCGCGCTACGTATGAGCACTAAGCACACCTGCTGCACTGAGTATGACCATCTCAATAGA

AAATGTGTAAATTTACGCAAGCAATATGCAAGATTGTTGATTGCTTAGCATATCCATGGC

CGTGTGGCAGACACACTTTTTGGGTGTTCTGTGAGTAGTGAATTAGTGTTCCTTGTGCAT

GATATAGTGTCTGATTCGTCAAAAAAAAAAA

>289

ACGAGGTAGAATGTTAGCAAAAGAGATGTGGACACTGATTATATAAGTAGTCCATAGATA

GACTCAGTTGCACCATGCTCGAACATAGATGAACTCGCATACAAACGTGTCATTGGAAGA

GATTGATGGATGGTCACAGACTGAAGGGCACGTCATTGGGACGTCTATGCATACATGATT

TTTTAGGGATGATGTGTGATAAAAAAAAAAA

>290

AAGAATCATCCTGTGCTTGTTTTCCGAAAAACTTGAACCCATGAACATAATATGCAGAAA

GAAACAATATACAGAATTTGCACAGTGCAAGACACCAGTGGGACATGCCTGTAGACTTGT

GATATTGTGTATTGCACCTATTAGTGGATTCATGGACATCAGTCTCTAGTTTTAGTATTT

TACTGAAAGCCGCGTCTTGCAAAAAAAAAAA

>291

GGATACCCAAATTGAAGTGTTTGGAGGATTTTGGGCCTATTCAATGCTTCAGTCATTTCG

TTGCAATGGCGAGCATTGGAGTGTTGTTGGATTAGAACTGCTGTAGCTTGTGACATTTGT

CGGCCTTGAACATTCCCTGACACAAGATTCCAGACTGTGGTTTGTGGTAAATTTTATGCT

GCTCACTGGTCATCCACCCTAAAAAAAAAAA

>292

GCTCAAGGCACGGCACAAGGAAGTTAAACCCTGGATAAATTATTCGGGAAAACTTTCCCA

AATTCTCCACCAGTCCTACTTCATCCTGATCAGTGCAAGCGAAGCAAAGACACGACATAT

GGCGCGATTATCTTCAATCTCGAGCAGGGAACAGTGAACGACGACAGCAGTCCGTCGGTC

ACAGAATTGACGGTGCACACAAAAAAAAAAA

>293

GGTGGCTTCTATGTGCATGGTGGCCATGCAGGAGGAAACTATTTCGACGATGCCTACTTC

TTTCCTGTGTGAGCCTCTTGCTTGGACTAGCATAGAGTATAGACTACGATAGAATGAAAA

GCCTGTAGGCTAGCATGTAGCTGAGTCTATACACTTTGCCTACATTTGATTCAAGTGTAA

TTCCTCTCCTTGTAATTTTTAAAAAAAAAAA

>294

CGCTGTTGCTTTGCTGTATGTTAGTTGGGATGCCTGGGTTTTTTATGGGAGTTTGTTGAA

TGACTTGGGGCTTTCCCAGGGCCCATATACATGTGGTTGGACAGGTTGCAAAGTATTTAC

ACTTACAGTGCATGGGGTCCCCATGGGGGTATTTTTTTGGCCAAAAACTGTAAAATGTTT

TTTCCAATAAAATGCTGTTTAAAAAAAAAAA

>295

AAGACTTTTCCTCGAATTTCTGGAGATTTAAGCAAGTACATCAACTTGACAGCACGCTGG

TGGTTTACTTGAAGTTTCCAGATTTGGAATCGGGCAACGGAGACCAACTGCTGCGTGAAT

TGCCATCTGGTTTGAGGGACATAGATGATGCAGGACTGCGAAGACTTGCTGGACTTTCCT

GTGAAGGTCTTTTCGATTCCAAAAAAAAAAA

>296

TATTTGAACAGTGAGGTATGGATATAGATTCAAACTGGAGCTGTTTGCAAAAAAAAAAGA

TTGTTGTGCATGGTGCGCACAACCCCCAGGGAGGTGCCACTACCTGGTAGCGGTTGGGGA

TTGTTCGTGTATTTGGCGCGAAACACCGAGGCAGTGAGTCGCGGTAGTATCATTCGTTAT

AGCTACTTTGCCGTATCGCTAAAAAAAAAAA

>297

TTCCTTCTCTCAAAAAAAAAAAAAAAAAAAAGCTGCCACAAATAAACTACGTCCCTTTGT

GGTGGAACAATTACGAGCATGTCATAATTGGTCCTCTGTTGGGATGTTCAACCAGGCTGA

TTGAAAACAATGAAAACGATACGCATTGTTCACCCATATGGAGAAGTAAGTTCATTATAT

TGCGGAGAATTCCGAATNACAAAAAAAAAAA

>298

TGTTTTCTTTGGGGGCGATCAGTTAGACCCGGCAACACTCTTCAACTATCTGCAGCGCGC

CTTTTGCGAAAAGTCTAGAGAATATAGCCGAAGTTGGTGTTTGTATGGAATTATATCAGG

TTCTTATTTTGCAACAGATCATCTCGAGCGAGCTGGAAATGAATATATCCAGTAGCGACT

GGGGTGTGTTGCAACTGTGCAAAAAAAAAAA

>299

CATCAATGTGCCTGCCCAGGGCCCCTACAAGCCCCTGCACTACCGTTATTAGATCCAATG

GCTGTGTGGTCGGCGCATGTACTCTGCGGACCGGCCAAGACTCCTTTGGGAACATACTAT

AGATGTGAGGTCATTAACACTAGAAGCACAACTCATGTAGAGTGTCTTANAGCTTTTTAT

GAAAATGTAGGCCCTCATAGAAAAAAAAAAA

>300

TCAGGCAGACGCATTCATAAATTGTGTTTCACAAGTTTGGGTTTTAGTGCGATATGCCTG

TGTTGCCATTTAGGGGAACGCAGAGAGACCATGCTTTGGCAGGCAATTGCCATGTGGATG

TCCTTTTCATGTAGGGGGCACAGTGTAAGTATACCATTCCAAGGATTTCAATAAGAAAAC

TCCGCGTTTTGGCTTGGTCCAAAAAAAAAAA

>301

TAGTTCGCAGTTAAACGGCATTCTAGTTATTAGATTGGGAGTCGAGATAGCAACTCTTGA

TATCCACAAACACAAAACCAGTCTTGAAATCTATACAAAAGATTGCACCATCAATGCTCA

AATGGAAGGTGCTATCCAACAGGCAAAATCTGCAAGTGATCTGTAAGTTGTAATTTTCAA

CATAGTTTAGCTTTGTCTATAAAAAAAAAAA

>302

CAGGTGTCGGCCGCGGAAGATAAGTCGGACTCTGGGCTTGGAAGCGATGGAGATCAAAGG

CCTCGATTGCTGCATGACATATAGAGGTCAGTTTTGTAGGGACCACAGTAGTGCTCTCCG

CCCTTTCGGCAAGTGTAATGCACCCACTTTAAAATTCCTTGTGTTCAAAACCTTTTATGC

CACTAGATATCATGGTGATTAAAAAAAAAAA

>303

GGTTTGCGGCGTGAGCTGGCCGAGCCGGAGCAAGCTGTTCAAGCATATTAAGCAGTCCGG

GCATGCAACAATGAAAATTAGTTGATTTCTCTCCTCTAGGCTCAAGCATGTGAAAGAAGT

TTTGCATAGTATTGAAGAATAGGAACAAAATTACGGATATTTCATTGCTGTAATTACGTT

AAAGGTTTTTGCTTGCATGGAAAAAAAAAAA

>304

GCCGTTCATCTGAGCAGAATATTGGATTTTCACATTTGTGGAAAAAAAAAAAAAAAAAAT

CGTAGACCGCGAACTGGGCGGGACATGCTCGCAAGCCATCAGTTGCCAGGGTTCCGCTTG

ACTTGCAGTAGCATTGCTTCTGCCGCATGCTCATAACGATTCTAAACCTCCCGACAAGTT

ATCATGAAAGTCTCCCCCCCAAAAAAAAAAA

>305

AGTTGAATGTCTTGTGTTCCAGTTGTGAGAACCTCCATTGTTCAATGAGGGCACCACAGG

GTAGAAAGCCACAACATGTGGATGTCATCAATGATATTTTTTGTGAGCATCACCACCACA

ACAAAAATTTCCCTTGCTGTCCTCAAACGTTGCACCTATGCCATTTGTCAAGTTGTAAAT

CAGGTACCGCCGTTGCATTGAAAAAAAAAAA

>306

TCGGGGATTTTGGGGGGGCCCCCCCTTTTTGGGGGGGTTGGGGTGGTCCCCCCTTTTTTG

GCCTTCTTGCCCCCCCGGGGGGGTTTTTTTGTCCTTTTTTTTCCCCCCTTTTTTGGGGGT

TTTCGGGGCCTTTTTTTTTTGGGGGGTTTGGGGGGTTCCCTTTTTTTTTTTTTTTTTTTT

TGGGGGCCCCTCCCCCCCCCAAAAAAAAAAA

>307

GAGTGCAACCGCGAACCTTCACTTTGTTGGGAAGTACCGAGACCTGCGGAGTATGATACT

TGTCCTTTCGAATGCACTTTTTGAATTTGCCCTCCATATTCAGGATCCGCTGAGAAATCA

CAGTGCAGTGTAGCCTTTTTGGATCCATAAGGCATTTCATTTGCATGGTTTATCAGCTCA

TGACTTTTTGATACTGCCTCAAAAAAAAAAA

>308

GCAGACAAAGCCGGAGCCTGCTTCGCCTGGGCTGACGGACGAGGCCACCGAGGATCCCAC

GGGTTCTCCCGCACCCACTCGAGCCGCTTCTGGTGGTCCTCCCATCCGAACTTCCCGCCA

ATCGGCACCATGTAGTTGGCGCTCGTGTTGAGCAGCGCGGTGTGCTCCTTGTTAAACATT

TTGGATATCTGTGCTATGCCAAAAAAAAAAA

>309

CTGGCATCGCTTCCCGCCCCCGTGTGCCCAGCAGAAGAGCGTGCTTCCCTGCGCTGACTT

GTCGCACTCGTGGGGGGCGCACCTCCGCCCACCCCCGTGCGCTTTGCAGAACCTGGTGGA

TCCGCGCGAGGTCTTGAGGCAACCAGGAAACTCGCACGGCTTACCGGGCCCGTGGTCGCG

GCAGTAGATGGTGGTGCCCCAAAAAAAAAAA

>310

TTGTCTCCTTCCGGCTAGTTCGAGTGGCGTATAACTGGTATCCATAAAGATAAGGGTTGG

GGTTGAGGGTGACCCCAAGATAATGGTGAGGTTGCATTTGGCTACATGTAAGTTCAGGAT

GGTAAGTTTACTGATTGATTGGAACCAAGCATTCAATGTTTAACCTGCCTCATTTGATTG

AAATTGAGCATATGATGATTAAAAAAAAAAA

>311

AGTGTTGCAAGATCAAGCCGAAGCTCAAGAACTCCACATTTGAATAAGGAACCGATCATT

CCTCTTTAGAAGAAATCTCAATTCTATATCATGTTGACAGGTTGATGCCAATTGCATGTA

TTTCATGCTCTGTCCTTCTAAGGATAATGTCTCAGGAATACGGATCATATATAAATTGAA

AATCTTGCATACATTTGCATAAAAAAAAAAA

>312

CATTATGCAGGCTATGAAGGATCGCATGAAAGAGCTTGCCAGCAAGATTGGCTTGCCTCC

AGGAATGGGAATGTAGGCTTCCTCATCAGCTAGACGAAACAGATGAAATGCTGTTCCATA

TAGCTTGACGCTCGAAGTTGATTGATAGTAGATGCACTTACGATGGGTTGTTTTGATAAA

CAGCCTAGGTAGTGCCTGTCAAAAAAAAAAA

>313

AAATACTGAAGCGTCGAAAATCATTCCACTTTTGACAATGTGCGTGAGTGGAGACACAAT

CAGATCTTTGGCGTTATCCAGTGTTGGGCTGTACAGCTGACGTCCGTAGAATTTGATGAT

CTACGGATATTCATGTTCAATTCTGGATGCCGCTTATCAATAATTCCCATCACACTAAAC

TAAAATCATGTTTGCATTTTAAAAAAAAAAA

>314

TTGAAACGTGTACTTTTGATAGCGAGCACAATCCATAAGGGATTCCATCTTGTTGTTATA

TGCGATCATGTCAAGAGTGTATAGACTTGGAGCATGTAAAAAAAAAAAAAAAATCGCATG

GTATATAGGCTACATGGACTGTTAGCACAGTGCGGTGAGTAACCATCACGAGTCGATGTT

TATGTTTTTAGCTTCAAAGTAAAAAAAAAAA

>315

TGGCACAAGTGAAATGGACTATCCATCATGATAATCTACACGATTGTGCATCATCAATAC

AGTGTTTCAAATAGCAGTTTTGAACAGCAGAATTCCATCTACGGCAATCACGCATCAAGC

CAAAAGGACATATGGTGCTCGGACTTTGAGTAATGGACCCCGTGCATAAAACATTCTCGT

GCGCAACGGCATACTTCCATAAAAAAAAAAA

>316

GGTTACATTTTTGATATTGCTGCAGGTCATACATTTCGTAGAAAATAAGGATCAGGATTT

TGTTTCAGAGTTCTTTCAAAGGCCTGAATCCACACAGGAGCAGAAGTCAGGGATGAAAAT

GGAATTTTCACAAGTTTGGGGGTAATCAATAGCTTGCAAGTACTTTATCCTTGATTTTTA

TCCACGCCTGATTATATTCCAAAAAAAAAAA

>317

TGTTCTTTTAGCAAATATGTTTTGGACCCCCTCTTGATTTTTAGGTTATACATGGGGGTA

GGCCCGCGCAAAACTCCCCCCGCTAGGGGGGGTATCAAGGGAATTTTTTTTTCCCTTTTC

GGCCCCTCTTCAGGAAACGTTTCCCCTGGTTGAAATCAGGGCTTTGTTTTGGCAGTATGA

TCACCCCTCCGGGGGGGCCTAAAAAAAAAAA

>318

TTTTTGTTTTTGCATGCACCCTAGTCAATCTTGTTTTTCATGTGACACACCTATACGATA

TTTGCACGTGGTGTGGAGGAACGCGAATTGCCGGCGGAAGACATGTGGAACACCTGTGCG

ATGTGCTGCAGGTTCAAATTTCATTCTCCATTATGTATGTACTCGATGTCTCATCATTTT

ACAAACTGCGGTTGAACACGAAAAAAAAAAA

>319

GCTCATCCGGTAGACATGTCTCCACTAAGTTGTTGCGCATGGAGCCTTTGCGCCTTCAGG

ATCTTGGGATGCAACATACGTTACGTATACAACTAAATACGCATGTGATAACATACCAGA

CACATTTGTATGTGTATTATAGCATTGATCACCATCATGACCATGCTTTTGCTCGAATGG

ACTCAAGTAGCACACTATAGAAAAAAAAAAA

>320

AGATTGTGTAATAAATCCACAGCGCAGTTTGTCGAAGTCGGAGAAGAACTCCTTGATTCT

AATCCTGTCTTTGTACACTTTGGTTTGCACCTTTACAACAGCCTCTTCGGTGGTTGGTAC

GGTAGCTTTGTATACCATGACTCCACTCTTGGTGTGAAGGTGAAAGCACAGTTAGGGACG

TGAGGCGGGGGGGTTCTCCGAAAAAAAAAAA

>321

TCGATCCAAGTAGTATCCATTTGTGAATTATCCCCTGGGCATTTGACATAGCGTTGGCCG

GGGGTGTGGGGCATTTTGGGTTCTTCCCCTTAGCGGCGGCAAAATTCCCCCCATTTTTTG

TTCAGTTCAGGCCTTTAAAAGGCCATCCCGGGGAAATTGCGCTGTAACCAAGGGCAATAA

ACCGGATTTGGGCAGGTGTCAAAAAAAAAAA

>322

GCTGGTGGGTGCTAACATAGCCAAGAAGTGACAGTGACATTTCAGCATAATATGAATGCT

GTTGTGTCGGATTCAGGACACCTCCCCGAATGACTGGGGAACTTGCAGAAACTGCATCAA

GGCAGCTGTTCAACCAGTTGTGCTATGTTGTTATGGTAAAAAGTATGTTTTTATCAAATG

TCATCATAGCTGTGTCAGTCAAAAAAAAAAA

>323

ACTTTGATAAGCGAACAGTCATAAAACTCATAAAACTCATGATTTTTTGGCGCAAGTCAA

CTTCATGCTTCAAGATTTGCACGTGCATCATTTTGTTCAAGTTTTGGCTTGGTACCATCT

AAAAACTACATGCTCATGTCTTGAACTAGAAATTCAAACGGCCATTAAAAGGTGATATGA

GATAACCATACCCCTATAAGAAAAAAAAAAA

>324

CTGAGTTTGGATCAGCAGCTTTACTCGCTCGATGGGTGCAGTGGCAGTCTTTGCAACTGC

CCCGGACACACCGCCGGCGGCAAGATCCTTCAGGAAAGAGTTCATGGAAAACTCCTTTTT

CGCAGGAATCATGGCAGGGACGGCAGGTGCCACTGCCGAGGCAGAGAACAGGCCTTTCAC

GCCGGGAGAGGGAGTGTGTGAAAAAAAAAAA

>325

CAACCACGCAATGTCACGCGCAGAGCATTATGCTCCTGATATGCGACGCGAACTGCGCTA

ATCCAGTAAGTACGGACCTCTGGACATCATATTTGGAATATTACATTCCACTAATATTTA

GGGTAGGGGTCAGTATGTCGCTAACACTGAACGCGTACTAAGGTAACACATGTAAGCTAC

ATTAAGGATGTCTTGGGCACAAAAAAAAAAA

>326

TACATATCATCGCGGCAGCAGCTTCTGTCATGCATGCCGCGATTGCTGTCGAACTGTAAA

TATAGGATGTCAGTTTCGTGTTGACAACGAAGCGCTCCTGTGTGCTTAGAAGTAAAGCAC

CCCAACACTCAAATCCCAGCGATAGGTTTTTGCGGTTTGTTGAATTGGAATTGAAAATGT

CCAACTTGCCATTATGAGTCAAAAAAAAAAA

>327

CCACTTTCAGCAGCTTTATGGAGTGCTGTGCGACCTTTTTTATCCTGTGCATGCACATCT

GCCCCCAGCTCCACCAGCGTCTTCACCGTCTCTGTATGTCCAAATTCAGCAGCTTTATCA

AGTACTGTATTTCTGCATCGATGTATATCCCGGATATTCATGTGTGCCCCCAGTTCGACC

AGAGCTTTCACCGCCTCTGCAAAAAAAAAAA

>328

AGGAGTGAAAAACGACTCAATTCGTATGGCTTGACCATGCGCGGCCAGCGACGAACTATC

GGCGTGCGCCTCCTCCTGTGACCCCACTCGCTTATTTTCCATAGTACGCGGCGCCGGAGC

TGGCCCCGTCTCGTTGTATTCTGTGATTGGCTCGGTTCTCTTCTTCTTCTTTTTCTTGGT

AGGGGTGACCTCCCCGAGCCAAAAAAAAAAA

>329

TGCGCTGTTTGACCCGGAAAATTGGATTATGTGTGAAGTAATGGAATCTAGCTCGTAGCT

TCGTGAGCATGGTACCCAGTATATTGGTATGAAGTATGTCCATGTGTGTATGTAGGTATG

GTGATAGTCAAGACAGCTGTCAATCAGCTTAATATTGTACAATGTAGTAGATGATGGGTC

ACATCAAGCATCTGCACATTAAAAAAAAAAA

>330

CTTCCTCACAACTTATTGGATGAGCTTGTAGCGACACGTTAACGTGTTGCGTATATGCCA

CATGCATACATACCAGCATATCAGCAACGTAAACATAGTGCTCATGTTATGGTGCAAGAA

ACTTGTAGATCGCCTTTGCAGAGTGTGTGCACAGCACATTGAGAACTGAAGGTTCCATAC

AAAAAGTATTTCCACCACATAAAAAAAAAAA

>331

AACACCCGCGGCAGAAAGACCTAAAAACCAACCCGCATCCCCGTCCGGCGACATGGCCCA

AGATTTTTTGCGTGCGTTGCAAGCGTGGGCGCGGCAAGTTGAGTTGGACCTGCAACACCC

GCTCCGCCTGCACCTTCAGGCAATGAAAGGCGGCGCTTGTTATTGGCAAGCAGGTTAAGC

TCGGGGCCTTAACCCTCACCAAAAAAAAAAA

>332

TGTACATGATATTGGCCGATACACGGCAATAGTTCAAACGTAGCAAGTCGATGAAAATAG

GCTCAGACGCAGGTCCATACCGTTGATGTGAACCTGCATGATATAGTGCAAAGTAGTTTC

GGGTTCTGGTCCCTATTTTGGGAATTTACGCCAAGTAACGGTTTATCTTAATGGGCGGCT

ATCGCGGCTGTTACCTGAGTAAAAAAAAAAA

>333

CCGCGTGCTGTGTTACCTAGCAACAGTATTTTTTTTTGTGTCGCTGGCATAAAGGATAAA

TGCGTTCTGAATGTTGTGAAGAAATGACCCTAGCCCCAGCTTCATGTTGATTCAAATGAT

TTTTTGTAGGTTTGAGTCCCTTTGATGTAAATTGTAATTGGATTTATTTGATATTGGGGG

TTTAACCCCTATGGATGCTTAAAAAAAAAAA

>334

TTCAGTTGGTTTGATGCTAGTGTGTTTGGTGTTTCTCAAATACCTGCACCCCACTCCCCA

TTCGTCCCTCCCCTTCCCCCAACGACGAGCAATGCTGCTCCACCTCCATCAGCGCAAGAG

GGTCCTTATGGGTGGATGTTGTGGGTCCTCTTCATCGCCGCCTTGGTGCTGATAGGTGTC

ATTTTGCATCTCGCACGAAGAAAAAAAAAAA

>335

TGAAAGGTTCGAAGTCGATGTTAACCTTTTTTTCTTTTCCGTCATCCGTCATCACAGTGC

CAGTATACACCACCAGACCGTTGGTGGGCACTCGGTTATACAGCTTCAGCCGCTGCTGAG

CAGAGGTGATGGCTCCTAGCACAGACTGTCGGTTGACTCGATTTTTAATATTAGACGCGG

TGCCAAACTCGTCTAATATTAAAAAAAAAAA

>336

AACTGCAAAAGTTTTCGGGACTTTATGTTTGTTCTCGTGAAAAAGAGAGCAAGTGATGTT

ATCAACACCGAAGGCTTGACCACCTGATGTTCTTAAAATCCATGTCATGTGGTAGGTAGA

ATACACGTATTTAGGGAACGCTTTCAATAGTATAGCATGGGTACATTGTTAATACTGTTG

AAGAAGATGTGCAGTATTTGAAAAAAAAAAA

>337

AAATTTCTCGACATAGAGAAACTGTTGAGGTCGAGTTTCCTGGATCGTGTGGAGTTGAAT

ATCTTTCAACTCGGGCTTGATGTGTCAGTGGCTCTAGCTTCCCAAACTGTAATACTTAAG

TGAGTGCCCCCTGCATACTTGTGCGAAGTGCTTCCCTTGTCGGGAATTTTTTTGATATAT

TTTAGACAATACCTATCGTCAAAAAAAAAAA

>338

CTCCATCATGTTTCTGATATACGGCTGTCAATTATCGCCACAGCCCGGTTGCTCGGTTCT

GGTCTGCTTCGCCTAGTGGGTAGGAGTTGTTAATGCTGAGTACTAAGACACATCTGTTCA

AAGGCGAGTCACTAGGTTGAGGATGTTGGTGTAACTTGTTCTTCGTCTTGCAACAGCATC

TTAGTAACATGTCTGCCCGTAAAAAAAAAAA

>339

GGGGGGTTCCCCCCCTCCCGGGTTTTCCCCTTTCAAGTTTTTTTGGGGGTTTGGGTTTCC

AATTGGGGGGGGGCCTTGGGGGGTTTGTTTTTTTTCATTCCCCTTTTCGGGGGGGGGGGG

TGTTTTTTTTTTTGGGGGGGGGGGGGCCCCCCTTTTTTAAAATCCCCCTTTTTTTTCCTT

TTTTGGAATTTGGGGGCCCCAAAAAAAAAAA

>340

TCGTCCAAACCGTCCAAGTCCAGTACCTTCTTCTTCTTTTTCTTTTTCTTTGCACCGAAG

TCACCAAGTGACTCTTCATCCTCTGCAGGTGCGTCCGCCTCCCCATCCTCGGCGCTCACT

TCCTTCTTTTTCTTCTTTTTTTTCCCGAACATTGCCAACGGGTCGTCGAGCGATAGTTCA

CCCATGGCTCCTGCGGTCGGAAAAAAAAAAA

>341

AGTTCTCGATCCGTCGCACATAGCATGGTAATTTGTAAGTTTGCAACTAACTCGCAGTGA

GCAACTATGGCACTCCAAGAAATCCATTGGGCTAACATATTTATGTTCACAACTAACATG

AAAATTACCACAAAATGATACACTTATATCGCGTGATTTGGAATGTAACTGTTGTATGAA

TAAGCCTATAGCAGATTGATAAAAAAAAAAA

>342

GTAGAATAATTGTCAATTATATTATCGCATAGTGCTTACAGTGAAGCCGACCGTCACAAG

CCAAACGAGAAGTTGCCGTTCGCATATGCTATGCACGCGGTTAGGGTTTCGCAACGCTTT

GTGTCCGTGGGGGCGAGAGAAATCTCATTGTTCCATACAATTACATTTTGTAAAATGCAT

GTAAGGTTGTAAAAATACGCAAAAAAAAAAA

>343

CAACTTGGCACGCCAGAGAGGCGGAGCGGCGCGCGGTGTTGGATCGAGAGGCGCGCGAGC

TCCGCATAAAAAACCGCACCTTGGCGCAGCAGCAGGCCAGGGAGGCGGAAGAGAGAGATG

CAGAGGCAAAGCTCGCCGCGCAGGAGCTGGCGAGGAAAGACAAGGAGGCCAAGCAGGCTG

AGGCCCTATTGGAGAGGATTAAAAAAAAAAA

>344

CTTTCGACCAAAGGCAGAAGCACGTCTTGGTATTCCAGAAAGCAGATTGTCTCCGAGAAC

AGAGCTTAATAATCGTCGGAGATAATCGTTTCACAACATGAAGACAACGACCACCACACC

AGGTATCCACAAAGAAAGGTGCACACAAAAAAAAAACATCGTTTTCGCAGCACACAGGTG

GTCCTGCTCCAAAATTGACCAAAAAAAAAAA

>345

GTAAGCTCCTGCTGCTGGTATACCTCCGGGGGTGTGTATCCAATACACAGTGCATGACCA

CCATATACAAGATAAATTGACAGTAAATCGATAAAGAAACCTAGAAAACAACCTTACCTA

ACCCATCACCTGTCAGCAAGTGTGCCAAAACTTAGCACTGCTGTACCTTAAAGCTTAATC

ATTTCAATCTTGGAATTCATAAAAAAAAAAA

>346

AGGATTTATTCCACTGTACCTACTGAGTATGCCGTTCATGGCTATTCTGTTTTATTTCCG

GGGTCCAGCTCCCCTGAATAACCCTCTCTACCATACCAAATCCGTTGTAGAAATACAGCG

CGTATTCGAGTTCGTTGTGAACAACGTTGATATTATGCATTTTATGGAAAGTACCAGCGA

CAAAAAATCTATTTTTGTTGAAAAAAAAAAA

>347

AGAGACTCGACTCAGAGGCCATCTGCTGAAGACTTGTTCCATGAATCTAAATCGATGTAA

GATTAACTAAACGAAGATCTTATTAGCTTCAACCTTGTAAATAGAACCGCCATACTCACC

TAGCACGCAACGTGGGCTTACAGGTGTATCGACACGTTGTAGGGCTGTATATTGTAAATA

TTATACATTTGGTGTCCTGTAAAAAAAAAAA

>348

CGCTTTCAACCGCCCACCATGTTTGCAAATGGCAGGTCATCTGGCAGAAGACTCGAGTCT

TAAGCAAGCTAGAGCTCTGGACGGTGAGTTCCTTTTTGAGCACATAGACTCCCCATTTTC

CCCGACATGAGGGCTATATAACTCACATCTCAAGGAGTATGGTCCACTGTTTCGTTTAAA

GTCATGTAGATTTTATTCCCAAAAAAAAAAA

>349

GGGAAAACAAGTTCCCCCAATATCATATGGGGGGGACACCACTACTCCATGTTGGGAGGG

GTCCAAATGTAGCCGTTCCGATGTTTACGAGGGGGACTTTGTTTTCCCATGCCAAGCCCT

CATGGGGCCATTGAACAAAAAATACCCCGACTTCAGTTTTGATGGAATGCTGTAAAATGT

TTGGGATTGGATACTCTTGCAAAAAAAAAAA

>350

GTTGACCGGCCGGCGGCCGGGAATTCCCCCCTTTTTTTGAGAAAAAAAAGAAAAAAAAAA

ACAACCCTTTTTGAAAAAAAAAAA

>351

AATCTTGTCGCAGTTCCACAATTCCTGCAACATATTGGCTTCCAATCATGTACAATGCGA

ATTTAGATATCATTTGCACATCTTACAGCGAATAGAACCTATGCATATCAAAGATTGTGT

ACATTGTTGATGTATGCACTCGCACATGAAGATTGCAGTTGTAGATGTGGTGCTGTTTGA

CAGAGAAGTTGTTGCACTGGAAAAAAAAAAA

>352

GGAGAACATATCTCCCCATCCCCATCGAGACAGTATGCTGATTTATGGAAATCCATACGA

AACAAACTCTTTGCGTGTTTCATATGCAGTTGGACGCTAGCATGCACCACCGAGGATTTG

ATCACAGTCGTCATTGAGCATGTCTACTCCAGCTTCTTGGGTTGAAAATAAACAATGGTC

ACACCGTGAGAAACGCATTGAAAAAAAAAAA

>353

GGCATGTTTTGTGCAAGGTGTCTATCACAAAACTTGCGAAGGGCGGCAACAACCGGCCTT

TCAAATGTCCTTATTGCCCCAATGAATTTGTTGTACAACAGTGCAAGTAATTGCACTTTT

AGTTTGATATTTGGATCAACTTGTTAATTGCTGAGGATGAGAGGATGACATGTTTGTGAA

TGACACCTTAAATGTTAAGTAAAAAAAAAAA

>354

GTCTACCAGGCATTCGCTTCGTGGGGGATAGGGGTGGGGGGCCTTTCTGGTGTTGCCTGG

GCCTACATCTGCACCCAGATTCTGCCTTTCTACTCATAGACTCAAAAGGCAGCAGACTGT

GAACTTTTGGTTAGCTTGTAAAAGAGATTGGATATTAATCGATGTTGTCTTGGTCGTAAA

AAAAAAAA

>355

GACCATGCTGTAAGTTGGACTTGCATTCATCCAATTCACATACAGGATGCAGCACCCGTC

GTATCTCGGTTGGCCCAATGCACAGCGAAAGGCGAGAGCACCTTTTATTCGAAGTCCCGC

AACCGAACTCTGTACAAACAACCAACTGGTATGGTGGGCAGCCAGAGCGTCACCGAGAAA

ACACGGGCGAACTTGCAAGCAAAAAAAAAAA

>356

TCGCTGCATTCCCTGCTGCTTGCGTTCGAGCAGACACCAGACCGGAATCCCGGCATGAGA

AGTGTTCTTAGATTTTAATGAATGAGCACATGTATGTTGTACTAAGTAGAGAAGAAATAA

ACTTCATACATTTCTTGAGCACAAAACTTAGATAAACACCTCATTTTATCATATAATGCG

CACTGTGAGTGCACACTGTTAAAAAAAAAAA

>357

CAAGCCTGGCGTGCACTTTTCGACGACAAGATTCCGGGAGACTTAGCGGCGCAGTGCTCC

CAAACTTAACGCGGACGGACGCACGTAATTTGACTCTCGAATGTCGGTTTCGATTGATTT

GTCAGTCTTTGTTGTTGTACAGCCAGTGATACACACTTCCGAAAGATATTCCGCTTGAAT

AAAATGTACGGTTTCATGGTAAAAAAAAAAA

>358

CCCGTGCCCTTTGGGCGGGGGGTTCCCTTTGGGATAAATTTGGGGGGGGTTTTTTGTGGG

CAAGATAACCGTCCCCGTTGCTTGTGATGGGTGAACAGTTCAAAGGCCCCCGGCTTTTTC

GGTAGGGTTTTGGGTGTATACTTTTTCATTTTGTTACTATGTTTCCGTTTTTTTGTAATT

AAGCCTGCCCTGATTTTTGGAAAAAAAAAAA

>359

TGCTTGCCCAGGGCTTCGGCCTCAGCCTTCAGCTTCTCAAGCTCGCGTTCGGGAATACCA

CCGAGCTCGAACAACAAGCGACCAGTTGTGGTTGACTTACCGGAGTCGACATGGCCGCAG

ATGACGATGGAAAGGTGCTCTTTGTCTCCAGACATGTTTACCAGGTAGCGCGGGGTGGGG

GTTGTGGTGGGCGTTCACGCAAAAAAAAAAA

>360

TGGCGAACTGACAGAAGATATGAAGTACGTGCGATCATGTCAACTGTGTAGACGTGGTGC

AGTGCAAGGCTTTCAGTGGCAAGCAAGTTATGCAATTTAGCGGCATATCCGGTGTCATCC

CTTATAGTATGCATGAGGTCTATGACAGTGTAGGCCGAGGGCCGAATTGTATGCATTGTG

CTCATGTGCATTGTGCACTCAAAAAAAAAAA

>361

ACCCGAAGTTGTACACATACGAATGCATGAAGCGATAATCACGGTGCCAGAATATGATGT

ATGTCCTTAGCCAACTAGAAAATGAACATAGGTACTTGAGCTTTATCCAAAATGTGGGAA

GTTATGACAAGTGAGTCGCAGGATACATTCTGTCGTTGCCCAATGTAATTTTTAAAGCTT

GTTTGTCAGCTCCAAACCCCAAAAAAAAAAA

>362

TACTATGACACCCCCATGCAGCAATATGTAAACGTTGGAGCTGTGACAGTATTGTTGCTG

ACGAGTTGGGCTATTACTGGTTCCTCCTGATGTTGAGTATCGCAAGCGGACCTCTTTAGA

AGCTGATTCAACAGCCACGACATTTAATCTTTATTGTCTAAAGCAGATTCCGGGCATGTG

GACAACCCACTTGTCGAGCTAAAAAAAAAAA

>363

TGGCAGCCAACTCCTCAAGGGAATCAGCGAGCCTCTCCGGTCAAACGCGAGGGAGCTGGA

CCGAAACTCAAGAATGTATACAAGCCCATAAACTGGCCACCCTCCGCATCTCTTAAGATG

CATCACACAATATTGTGGAACTGTGCAAACTGCGAACAATATGTAATACACATGTAAAGC

ATCATGGATTAGTGCTTCTCAAAAAAAAAAA

>364

ATTCACAAAAAACGTCTGGACATCATGAGGTGTTGTTGCGGTGCAAGTTGAATAGCATGC

ACACAGATGCCTCTCACAGCCAAAGAGAACAGGACCGGAAGGCAGTTGAGGCAGATTTAC

AAAATAAATGTTATCGCGAGTCGTCTTCATTTGAACAAACAAAAATAATTTTTTTGAAAA

TTTCACCAACAAAATTAGCCAAAAAAAAAAA

>365

GCTGAAGATCCGGAGTGAAGGTTCTCAAGCTTCTGGTGGTGCACTAAGGGTCAAACCAGC

AGCCAAACTGTGATAACCGCTTCCGTACGGACATAGTGTTGGTTCATGTGCGTTTTATTT

ATTTGTTTTCTAAAAAAAACATCGCTTTCAATCGCATTTTCCATGGATGTGTTCAGTGGA

TGGCACCAGAGTTGTAATTTAAAAAAAAAAA

>366

AAGTTTTTTTTGGGTTTACCCTTGTTCCTTCAAGGGCCCATTTGTGACCCTTGGGCCCGG

GAAAGGATGCACCCTTTTTGAATGGGATTTTTGGGGGGGTTGGGTGGATTTGTTTTTTTT

TTTCAATTGGGTTCCCTTGGACCCCCCGCCTTTGGTGGTTTTTCCTTGGTTTGGGGGGGG

TTTAATTTGTGCCGGTCCCCAAAAAAAAAAA

>367

GTGCATGCGTGATATGTACCGGTGTGCTAAGCACAAAAGCAAATGGTATGGTTTAATACC

GAGAATGGAAGGGTCCACTTATATGAGAACCACTTGAATAGCTGCTACAAAGAGTGATTG

GACATCATCCAATCATGAGACTCATGAGATATGTGTTTATGCATAAGCTTGCACAGTGAA

AAATACAACAACAACAAGCTAAAAAAAAAAA

>368

GAAGTGAGCTGATGCGTGGTTTGCCTCGCTTGTTTCTAGGGTGATTTCAATCAGACATTG

TGTGTGTGGACATGTGATTGACTTTAAACACCGACTCCACCATGCATTCGGCATGGACAT

GAACATGTGATTGCTATCATGTGGATCGGACCGTTTTGTAGTATTGTGATTATAGTGTAA

TCATAGTATACGTTGCTATCAAAAAAAAAAA

>369

ACTTCCTCGAGACACGTACCCAAAGAGGATGCAAATTCAAATGGTCTCCAATCGCGGTGG

AGTGTCCACATGAAATTGACGCATATGCAGGGATTCTCGCTACAACACTCTTTCCTGGGG

ACGATGAGCACGGTTCACAAGACCGCAGGTGCCAAAGAAATCTTTGCGCACGCATTTCGA

TTCACACGTACAGAAATATGAAAAAAAAAAA

>370

TGCCGCTCCAAGCAGCTCCCCGACAGTTTTGCAAGGAGCAGGGCCTGATATGTAAATTCA

TCGATTGTGGCCAATATATTAGCAAGCAAGCGCGCAAAGATGTAATACGCAGAACCGTCT

TTTAAGCCTAGGTGCCCTTTTCGACATGAAAGGGTTGATATCATGCGAATGTGATCAACA

AAACCCAGTTCCTTTGGTGCAAAAAAAAAAA

>371

CCAGGATGCCTCTGCTGAGGAAGAGGGCGAGTTTGAAGATGAGGAGGAAGAGGAAATGTA

GATTGGATAGGGTCATCTAATGATATGTTAGAGAAAGCAGTGCACATCTCTCCCTCCTCC

ACTCAACCTACAGTCTCTCATGAGATGTGAACAGTTTGCGACCGCTATAGGTTGCCATTA

AAACAAATGTACCATGCACCAAAAAAAAAAA

>372

TCAACAGTGACGTGCAGTGCCACAGGCAAGCGCTATCATAAGCTGGCCATCCAATGACAA

TGCACGTAATTACTTACATTCATCCGGTACAATATCTAGATGTGAAGGATCGATTCACAT

ACAAAAAAAAAAAAAAACCTGAACTCCTAGATGCTATTTGGAGTAATTGCTATCGCACAT

TACTAAGCCTCTATAGGATCAAAAAAAAAAA

>373

GGGGGGAAAGGCCCCCTAAAGTTGGGGGGGATTTTTTCAAGGGTTTTTAACCCCCCCAAG

AAAATGGCAGTTTTTAAGCATGCCAACTGGATGTTTGGTTTTTTCCCAATGAGTTGTGGA

TTTGTTTTTGCACCCGGGGGGAATTGCAATAAACAAGTTGGCATTTTGGTTTGAAAAGCA

CCCCCTGTTTTTAGGTGCTTAAAAAAAAAAA

>374

GCTCTGGACTATGTTAGTTGGTGCTGTAGGAATGACGACCCCAATGGCTATCGCCCTGTG

CCAGTGAGATTGCCCATGCCAGGTGCTTTTGATGGTCACAGTCGGTGTAAACGGGAGATG

CAGAACACCACTGCGCAATGAGCATCACCAAGACTCATTGTCAAGTTCAACTATATTGTA

CATATGATATGTACAATCCTAAAAAAAAAAA

>375

TATGTTCATCAATTGGAAACCATTCTATTTGCAGATTTAGGCAGATGGAGATCCGCTGGC

CATTTCATCGCTTCTAGTACCCACCCAAGTACTACGCACAAGAATGGCTCTGAGATATGT

ATCACATGAGCCATGCATCCCATGAATTGACAAGTAAACATGTCTTTGCTGTATTATATA

TATATAGGGACATGACACTTAAAAAAAAAAA

>376

TGATTGTGGTTGAATTCGCAACTGCAACACAGCCATCTCCAGCAGCAAATACCGCCCTTG

CGTGCATCACTACATAACTGTTTTTTGAGTGGGTAGTCGACATCTCGATTGAAGAACCAA

GAGGGACGCTGGTTACTGCCGGGATACGACAATCCCCCCCACGATAATCAGGACCACAAA

CACACTGCCCGATCCAGTGGAAAAAAAAAAA

>377

CACTCAAAGGATACGCGCTGTAGATCAAAGGACACGCGCTGTAGAGCAAACGAACTCGTG

CATTTAGTGCGGGAACCCAAACCTAGCCCATATTATCGCATGAACTCTATCCACCAGCTT

TACATAAACTTTGCCATTTTGTTGTATTGTTGGTATTGGTGTACGGATTAAAACCCGAGC

ACCCTGCTCGAGCCTAGTTTAAAAAAAAAAA

>378

CTGGTGGAGAACTTGAGGAAGCGTCTAAGAAGTAGACTCGTAGCGTAGACCTTTACTGTC

CCGACTGTTGGTTTGGCCAGCCAGGGCGTCAAGACCGCATTCCTTTTTTAGCACCCGTTC

CCACCCTGGTTGAGTGGTAGATGAGACGTTTGATTCAAGAACTGTGTTATGAAATATATG

GCACGTATCGGTTCACGATGAAAAAAAAAAA

>379

GGGTTGTCCCCGGGGTTCTTCCCGGGGGAATTTTTCCCCCCGCCTTTGGGTTTTGGTTTT

TTCCAGGGGGAGGTGTTGGGGCCGGGGGCGTTTTTTGGGGGGCTCTGGCCGGGGTGGGGG

GGGTAATTTTTTTTTCGGGGGTTGCCGGGTTCAATTTTTTAGGTTTTTCCTTCAAAGGTT

TTGGAAAGGCCTTTTCCCCCAAAAAAAAAAA

>380

TTGAACCCTGCGTTTATAGGGAAGGGGTTGACAAACAAGCTGGCAATTGGGGCACTTTAA

TAAGGGAATGAATTGCTGGTAGCATCCAATCACTGTTTGGTAGAAATTGCTTCCATAGAA

ATGCCTCAGGGAGTTAGGGTACACCACCACCATTCTTCAAGCGTAAGAAACAAATGTAAT

GAGATCGGATTACATTCGCGAAAAAAAAAAA

>381

CTGAACGTTGTGGGTTGTTCCATATGACAGTGGACACGCCTGCCCTCTGTTTGTATTAGC

ATGGTGAAGCACTAGTCAAAAGCTTGTGCCAAAAGCATATGTATGGTAAGGGTCAACGTT

TTTACCTGGACAGTGCCCGGCTTAATTTGATGATCAGGACCTTCAACCTGTGAACTGGAT

AATAATATCATATGTTGTTTAAAAAAAAAAA

>382

GATGCACTTCAGCGCGCGCATACCCCGTGGCGACCCAAGCCCCACACCACGAGACGGCAA

TAAGTGCAACCTGGAGGCACAGCGAGAGTCCATTGTCTTGATCTATCAAACTATGAGTTG

AGCGCAAATCCTTTTGAGATATGTATACAGGATGGTTCACACGGGCAAAAGGAGCAACCG

GGTCCCACACAATCGTTTCCAAAAAAAAAAA

>383

GTGTGCAAGGTTCATTGTCGTGGCGGCCCCACGTGAGGCATGGTGGTGTGGTGCCCATTG

TGTCAAAGGTGAAAGACCCCGTGTGGGAGACAGCTTCCTCTTTGCTGATCGTATCAATAG

ATAAGTTTGCTACTTTTTTTTTTCCCAATCGTGAGCAGCCATTCCAATTGGTACATAATC

TGTATCCAGGCCGAGTTTGCAAAAAAAAAAA

>384

GCGCCAATAAACAATCTTGCCATCAATATAAGCATCTCTAGATGGTTCTGTCATCCAAAC

CAAACTGAGCTTAGCTTCCGCGACGATGACTTTGCAAGCAACATTCCTATGACGACGGTC

TGGTCGTATGAAAATGTATGGGACGTTACAATCGTTTTCGGGAATTGTATCGTAAAATCT

TGTTCACGCAAGTTTTCCCTAAAAAAAAAAA

>385

AGCTGCAGCTGCAGCACCTGTTGTTGAGGAAGAGGAAGAAGAGGAGGACATGGGCTTCGA

TTTGTTTGATTAAGGTTGAAACTGTTAGACCTCATTTGGGTTACTAACATGGTCGACAAG

TTTCCAGCGCATAGTGGCCTGACAATGAGACACTCCTTACCACTATGGTGTCTTGTTAAG

CATTAATATGCCCACGACAGAAAAAAAAAAA

>386

TATACTGGCGGCTTGTACGATCATTGCTAGTGGTCGATGATAAAAAAAAAAAAAAATGGG

CGTTTGTTTATACTATAAACAATCGCATCACCTGGCTAGTATGCTTTTCCTAGTGACTGG

TAGCTATTACTACCTAGTACTATGACACACCCCTTAATGGCAAAAGTCACTAGGTTTCGA

AATGGTGATCCTTGTTACTCAAAAAAAAAAA

>387

TGCAGATACTTGGTGCCTGAGCAATCTTTGCGACCGGATATTTCAAGTTTTTTTGTCTCC

TATCACAGAACAGTTGTGCTGTGCTCACGCAAGGAGCAAGGCACTGTGATTAGATGCATG

AACTTTCTCAGAAACCATGAAATAGCTCCTTGTTCTATTAATTTTTATTTCCTTGACCTT

GCGTGAATCACGCGCACGTTAAAAAAAAAAA

>388

GCCTGAAAGGAGCCATTGGAGGATGGATGCACCAGGCTGAGAACTAACCTGCCGATGTTT

TCTTGCTTGTATTTATTTACAATAACAATTCTATGTACCATGTTCGTTGAAATTGAATGA

GATCCACTCACTCGTGTGTGGACACTATTGAACATTTTAAAAAAAATGTGTGGTTTTCAT

TTCAACCACACACATGTATGAAAAAAAAAAA

>389

ATTCCAGCAGATCGCTACTATATCTGTTTTTATGATCTTCCTCGAGAGAATATCGGGTAC

AACGGGGCCACTTTTGCTGGATGAGGTGCAACATAAAACAGTATTCGCTCTGTGATGACA

TAATGATTCCGTCTGTCAGTAGATACGCAGACCATCGCTGAACAGTTCGTTGTATTCAAG

CTTACATTATTTTGGGGGTGAAAAAAAAAAA

>390

TGGTTCAGGTATATCAACAAAATTGTCAGAGTACTAGAAATATCCCATAGTGGCTAGTAA

TGTGTGATAGCGGTTACTCTAATTAGCATCTAGGAGTTCAAGGTACTCAAGGTATTTTGC

ATTGTGGATATGTGAATTGATCGTCCACATCTAGATATTGTACTGGATCAAAAAAAAAAA

AAAATTGCATTCCGATCTATAAAAAAAAAAA

>391

GACTAGATAGNTTCCTGGGCAGGTGGACTTTTACCGCAACTGTACATAGTTTTTTTCGAA

AGTGAACGCACGCAAATTGTGGACTAGGAAGTATGGCCTGTTGGATAATATGAGTGGGTG

ATTCAAGAAACTGCTTGCAGTTAGGGGCTTGAGACCGTGAGAAATATCATTCCAGATTTA

ATATTAAAATGTTGACTTGTAAAAAAAAAAA

>392

ATGTTACAGAGTTGTATCGTGGGCAACGTAGTAAGCTAGATTTTGCTTTTCATGTAGCGG

TCCTGACTTGACCTGAGACTGGTGTCCCTGTCCAAATGTGCATCATTGTCTGAAACATAG

TTCTGAATAAGCAGGTGTACGAACAAAATCAAAAAAAAAAAAAAAACGAGTTATAGTCGC

AGGCTCCTCTTGAAAGACTCAAAAAAAAAAA

>393

TGTATATACACATACAATACATGTATACAATATACACACATATTATATCAAAATGTATCT

GGAACAATGCACAGGGATGTGTCTTCTTGTTACAGATACATTATGATCCACAATCAGATA

TGTCGGTGTACTTTGCCATATCAGGTAGGACTTCAATTTCATCCACACATCCTTAAGCAA

ATATGAGAATTTCCCATCACAAAAAAAAAAA

>394

AGTGTTGCTCTCAGGAAGCTATAGAGTGTGCAAAGACGATTCAATGTGGACTTCTTTACG

AGTTTCAGCTAATCATACAAGCATCCATAATTCACAGTGACTTCCAGGGTTAATGGGACT

GGCAACTGCGCGAGGTGTCATTGTATTTTTTACACTTGAGGTGCCTGTGCTCAGAAATGC

CTTACTAAAGATTGTCCCCTAAAAAAAAAAA

>395

TGTTTGCAACAACACCTATCGCTTTACGTTCGCTTCTAAATTCATAAAGGGATGGGTTGA

GTAATTNTTGGAAAAAAAAAAA

>396

AAAGGAAGGGGCAGGAGTCAGTTCTGCGTTTCGCATGTGACTAAGTGCGATATCCCACAG

ACCACGGGTGTGGAAAAAAAAAAA

>397

CCGTTGTCCCCGTGTCTGCTTGCTTCAAATCATCGGTACCTTGATCATCATTTGTCAGAT

GTAGCAAGCAAGTTAGCGTTTGCCCCAATCAGTTTGCATGGATGCTATACAGCTGATAAA

CTCAGGACACATGTACTATAGCTAGTGAGAACGAGTGTCCATACACTCAATTGATTTTGT

AAATTTAATGTACTTTTGTCAAAAAAAAAAA

>398

TTGTCATCTGCACTTGGACAGCGCAACCCACGGATCTGGTTGTGGCGTGAAGAATGTTCC

CTTCGCAGGCCATGTCTCGTCAAGCGTGTTTTGAATTGGATATGAAGCCATATCTGGGAG

TTGCTATACCTTTAACTTTCAATGACATGTATAAGTATTAAACTTGATTTCAATTTTGCA

ATGAATACTCGAGTTGATCCAAAAAAAAAAA

>399

AAAGGGGGGGCCCCCCCCCGGGGTTTTTTGGGGGGGGCCCCCCCCCCGGGGGGTTTTTTG

GGGTTTTGGGCCTTTCCCCCCCCCCAAAACCCCCCCCCCCCGGGCCCCCGGGGGGGGGGG

GGGCCCCCCCCCCTTTGGGGCCCCCCCCCCCCCCCCCCCTCTTTGGGGGGGGGGGCCCCC

CTTTTTTTGGGGCCCCCCCCAAAAAAAAAAA

>400

TGATTTAGATGTGAACAATATGTCCGCAAATGAGTACATGCCTCCGAAAGATAGCGGCCA

AATATGAACAAAGCAGGTGGTTGTACAAACGTAGTGTGATTTTTCAGGACAAGCCTCATT

GTCAGTACAGCTTGTGGACTAGGGCATATGATATATTTGTGTGAAGTTGTTATGAATGAT

CATTATTGGGAGAGTGAATCAAAAAAAAAAA

>401

GGTTGGGCCGTTTTTTTTGGGTGGTGGTGCGGGTCCTAAATTGAAGGGTTGGTTTGGGCC

ATTTGCTTTTGGCGGGGGTGCCCTTGCCCTCAAGCCGGTGGAGGGCCCGTGGAATCCCCT

GGTTTTTTAAGTTTTGGCCGTTCCAAAGGTAAACTTCCTTTCCAATTTTTTTGATTTTTT

TTGGTTTGGCTTCGCTTTGGAAAAAAAAAAA

>402

TCCTGTTGACGGCAACCCCATGACATGGTTTCTCTGCTGGATGGGCCATGGATATGTAGG

CGTAGTGAACATTTAGATGGTCAACATTAGCCCTAGCACCTGTAGACTTACAGCAACGGA

ATGGAGCTGATGACGAAGAAGATGAGATGATAACCCTCTGAACGACGTAATTCTCATGCC

TTCAGATCAGCACGTATTGCAAAAAAAAAAA

>403

CCGTGCGGCTGAAAGAGCTGTTCGATTCCCAAGTACTAGCAGGTCAGGATTATTCGATTG

CAGGCTCGGGATTGGTTGTAGTCTCTGTGTGTGCGTATTCAGTCATCAAACAGGCACTAT

GGTGAATTCTGTTCAAGTAAGACTGTTTGGTCTGTAGTCAGCCAATTGTTCCTTGATTTT

ACAAAAGAGGTTGTTTGGAGAAAAAAAAAAA

>404

CCCCCCCCCCCAGAAACCCCCCTCCCCCTCCCCCCCCCATTTGGGTCCGGCCCCCAATTA

AAAACCCCCACCCCCCCCCCCCGGGGGGGGTCCCCCCCCCCTTCCCCCCCCCCCCCCCCC

CCCCCCACATCCTTTTTTTTTTATGGGGCCCCCGCGGGAAAAAAGCCTCCCCCAAAAAAA

AAAA

>405

AGCAACAAAGTCTCCAAGCTTGCATGGCTGCTCCTGCATCAGCGTAGGCCTGAGATCTGG

CTGGTTAGTAGTAGTGCCGAATAGCTAGTTTGTTTGTGAAGCCGTGGCTGCCATTGACAA

TATAGATCATCCCTAGACAGACACAAACCTAGATTCATGTCTGGATTGATTTAAATTCTC

CTCTGGTAGGTGTATTCCCCAAAAAAAAAAA

>406

TAATGGCACAGGGGACCATCCGTGCTGTGAGCAAGCACGGTGCACAAGTGATCTACACTC

GTGCAACCAACACCTAGCTTGGATGTATAGTTTAGTTACCAGAGTCTGAAGGCATACGTT

TTACATTGTGCCTGCTGTCGGCTTGAATACTGCAACATGTAATTCCATTTTATATATCGG

CTCGGATTTCGCCTCTGGACAAAAAAAAAAA

>407

TTTTTTTAGCCCCCCTGCCCCCCAAAAACCTGGGGGCTCGTTCCCCGCCCCCCTTTTTTC

CAAGGGGTTTCCCCCCGGGGGTTTTCCCTTTGGGGGGGGGGGGGGCCTTTGGGGGGGGGC

CCCCCCCCCCCCGGGGGTTCCCCCGGGGGGGGCCCCCCGGGGAACCCTTTTTTTTTTTTT

TTTTGGGATACCCCCCCGGGAAAAAAAAAAA

>408

ATGTTGAAACCCCGCGAATGTGGAATTTTCTTAGGAAACACTTAGATGTGTTTAGGTTCA

GAGAGGGCGATCTAGACAGGAAACTAAGGACGACAGATGAAATTCCGGAGGAATAGATCT

GATGAGACATGTGTGTGGATGAGAACTGTACCGTGCATGGAAATCTAAAGTTGCGTGTCT

ACTGCTTTACGTATATCAGCAAAAAAAAAAA

>409

ACAACGCCAACAAGCTCGTAGAATTGATGTTCAAAGCCTCTTCCTTATTGGCAATGTGCA

CGAGCCCACCCCTGTCCCAATGAGCCCTAATCACAGCGAGGAACCAATCCACCGTGGACT

AGGAAGGGGTGCTGGTCCTAGCTCGATTATTTTTGGATAAATCACTCTGCATGCTTTTAA

AAGTTGTGTAATGGTGGTGTAAAAAAAAAAA

>410

GTGGACAAAGAGGAAGAGGAAGAAGAGGTCGCCGTGTCTGCTTAGACCATCTTGTTTTTT

CAGCGGACCTGTTCATTTTGGAAAATGTTGTTAAGTGTTATCTGATCGGGATCTTGGCGT

CGATTGAGCAGCCAAATGAAGCTCTGACTGTGAAACCCTATTATCGTCAGATGTAATCCA

ATCGTCCTATACATGATTGCAAAAAAAAAAA

>411

CCCCCTTTTTTATAGGGATAACCTGGTTTGGTTTTCCGATGGGAACCAGGGGTTTTCTTT

TCCCTCTTTTTCTTTTGGGGGGGGATCCCAAAAAATTGTGGGGGTTTCCCCCTTTTAAAA

AGCTTCTTTTCCCGGAAAAAAAAAAA

>412

AACATCTGAGTGAATTACAACATGCACGGTCACAACATGTACATTCACAACTGATTCTTT

GGTGTAGATGACTAGCACGCCACGTACTCTTTGCGGGCAGGCATCAGGTTTTCAGCAGAA

CCTGAATCCAGCACAAGGATCAAGGAATCTCGGACATGTAAGATTATTGACGTACACTAT

AAGTGACTTATCGTTGCGGGAAAAAAAAAAA

>413

GCAGCGTGTGATTCTAGTACTCGAGTTGTCAATTTTTGGGACGAATGGCATGTTTTGGAC

ACGTTGCAACTCCAGGCCAAACTCACTACCCCTGAAAATAAAGGTAGTTAGTAGAAGTAT

GTTGGCAAAATACCAAACTTTAGTATTCCACATGTCATGTACAATGTTTCTAGTATACCC

CAGATTATATTGAATATGTCAAAAAAAAAAA

>414

CACTTCCTGACGTGGTGCCTAAAGCTGCAGGATACGACCGTAGAGAAAAACGTAGGTCAT

ATCATGAGGATTCGGATCCCCCTGGTGCATCCGACACAGCTGAAATGTCGCAAATTGTCT

AGTTTTTTTTGTACATTAATTCGTTTATAAGATACAGATGATGAAGTTATTGCGACACGC

ATCGTCGGGTTGGCTTTTGCAAAAAAAAAAA

>415

CATGTGGTACGGTATGGTGACTCGTTGTTTTCATGGAGCAACAGCGTGATCGGGCACACG

ACAAGTAACTTATGCGCAATGAAAATAAACCCATCAGCCAACTGGTGGTGGCCTTCANAA

NGTCAAGGACCCAAAGCAATATCCTCTCAGCACAATCTAAAATTAGTATGTACTAATTCA

ATATCAATAGAATTGTCTGTAAAAAAAAAAA

>416

TGAGGGGCACACTTATTGAACGTGTGTATGTACAACTAGTCCTGACAATGATTTTCAGGA

ACAGATGCATCAGTAGGATTGAGGGTAAAGCCAAAGACTCCATTTATGATAGGTTCACGG

TACTGACTTTCATAGCAGTGGATGTGTGATATGCACTAGAGTTGTATGTCTCATTTTAGT

CTCGATAATCCGAGTGCTCCAAAAAAAAAAA

>417

TGATGTGAGGCGCAGACTGTTATACACGTCGCGTGACTTGGACTGGCGTCTGGCTTCATA

CCAACGCAGTATGGCAAGTTTTATCATGGATACGAGGCTCGATAAACTCAAGTTGCAGTA

ATTAGAAATTTGCGCGAGAGCACGCGCAACAGTCAGCGGAACGCGGTCTCGCGTCAAAGC

GGAGAACACGAAGCCCGACCAAAAAAAAAAA

>418

ACGATGCACTAGTTTTATTAGTCACATACGTTTTAGGCATTCAGGAATTTGCAATGATTT

GAAAGGTGGCAACCATCTAGATATGCATAGAACAGCACAATCGGTACGTACGATACACTC

TCTGATACATGCATCAGGGATATTATGCACTTTTCTTTGTTTGTATCCATTGAATGCAGC

ATACATGGTATTGGTAGATTAAAAAAAAAAA

>419

GATTGGTCATATATATCCACACGAGTGCACGTTGCCGGCGCTGCTATGCATGCCCGGTAT

GCTGAGTGTTGCTGAATTGGAACTGCTTGTATGATTCCAATTCCAGTAAATATGGCAGAC

ACGACACTCATCAGCACTGCTCGCGCATGAGTCGGCCACATGGTACCTCAAGGATGAAGC

GACTGGTGCACGGAAATGTCAAAAAAAAAAA

>420

TCGCAGAGGGCGGAGTTCGAGCGTGCGTACCTCGAGACGTCCACCTCCGCATTTTGTTGC

TTGGAGGCCCGACCCAGGTTCATGCGCCGCAGCTTCAACCCCAGCGGTTTTGCCGCTGGG

GAGGAAGGTTCGAACGCTCGTGCGCCGCGCGAGGAGGGGCTCCCGCAAACCTGCCAGGAG

ACGTGCTCCCAAACTAATGGAAAAAAAAAAA

>421

ATTAAGAGCTATATTCGGATGCCACGAACATCTTTTCAAACTTGGAAAGTGCCTTGTGAA

AGCAAATAAGGGTGTCAGCACCGATAGCTGTTCACAAACAAGCCGTGAATGCGTACAGCC

ATTTATGTGTCAAAAGCCCGTGATGGTTGCGATCTATCAGGCTTCGAAACTCATTTTTTT

TTTTATCTCCAATTCACACCAAAAAAAAAAA

>422

CAAGGGGTTTTGGGTATCAATTTTTTCCTGATAAGCATTCATACTGCCTCGGCCTTCAAT

GTTATAGGCATAGATAAGCCCGTTTGCTTAAAGAGGTAGAAGCCCCCCCCGGGGGGGAAA

GTACATCCCTTTCAAACCAATTGCCCGTTTTGGGGGTGGGTCCCCTTCCTTAACCGTTTA

GAAGGGTAGAATCCTGTGCCAAAAAAAAAAA

>423

GCTTACCGTGATCTTTAGTAGCTGCCGCCCTTAGATATGTCAGCTTCTCAAAACAACACG

ATGTTTTGGAACTAACAATGTCTAAATATGAGATGGGAGTGCCTTCAGGTTAAAAAAAAA

AAAAAAAAAAATCTGCAAGGTCGCAGCATTCACAGAGCAGTTTCTTTGTTCGATGCGTTA

CAAGTTTTAGCTTGTTGTGCAAAAAAAAAAA

>424

TTTGTCGTCGTTGTGTGGTGTAGAGATGTTTGCGTATAAGTGGTGAGCTCGCCGGCCATG

AGGCCGGCACACTCCGCCGCCAGGTGGCGGCTCTTAGGTCAACGGTCAGAAGTTCGGGGG

GGGGGACGATTGATTGGGGTTTTTTCAGAGTCAGCTATTAGTATCTATCATTATCACATA

ATCACTCTTTGAAAGTAGACAAAAAAAAAAA

>425

GCTCGAAAAAATCAAGGCAGATCTCGCCCGTACAGAGCAACCATCATGGCACCGAGCGAT

TGCTTCAACGACGAGGTGCTGTTCGTGTTCCAGCGGCAGTGAATCAGGCCAATAGTGCAT

AGTGCATTGGGCATAGTCCTGAAATGTCTGTGGTTTGGTACACTTTCCGAGAGGGCAATT

TATTATATTAGACTGAGGTTAAAAAAAAAAA

>426

GAATGCCCTTAGCATCCATGCAATCGTAACAACTATCCATGTTTATGAACAAAAGAGGTG

GAGATGTGTCGAAACCCAATTACACAACCAAGCTGCTCAAATTGTGGTCTTACATGATGC

CAAAATGAAGTTAGCGAAAATGAAGTTAGCGAAGTCCATACATGGTATTATGATGAATGG

TGCGTATACCCTTGTCACTTAAAAAAAAAAA

>427

CACGCGCTTTCCACCAGGTGAGCATTTTCAGCAAAGTGTATCGGGACGAACCAATACAAC

ACTTTGCTGCCGAACACTTGCTCAATGTTTTTGCCTTGCTTTATGTAAACTGTTGATGTG

CGAACATGCCCTAGTGCGTTTCACTTCACAAGATACTCTTGCTAGTATTTGAGTGCGAAC

CGTAAGGTTCACCGGCGCTGAAAAAAAAAAA

>428

GCTTTGACTCTATTGTGCATAGTGCAGAGGGGGTAGTGGGCCGACTCTATTGTGCATAGT

GCAGAGGGGGTAGTGGGCCGGTCAAGCAACAAACACAACGTCTGGCGTACACAAATGTAG

CATTGGAGGACCAGTGGACGTATACGTTTGTAGATGAGCATAATCCATGTTTTGAACTAG

TTTAACTGCTAGCAACAACCAAAAAAAAAAA

>429

TTTCCTTCTAGAATAGGCTCTTGTACCGCCGTGCAGCTGCAAGTGATCTTGGAGCTACAA

GTGTAGTTGGAATTGATTTGCACCCTTCGAACTTGCATGAAGCCCGAGACCATGTCGTCT

GCTTGGCTTCTTGACAAGACGGTTGGTGTTTCTCGTGTATTTCCCACACTTCAAATGGTG

TTTAAGTGGTTGCTCGCTGTAAAAAAAAAAA

>430

CAGCGTCAGCGCGCAGAGAAACATGTCAATCAATAACAACCAGCGAATGACTAGAAGAGC

CTCCCTCGTTTTCAATACGTTTGCGTGTTTTANAGACAATGAGTGTGTTGGTATATTGTA

CCGATTTGTGTTATTGCAGTTAGCTCTAGCATCGCACATTGCTTCTGTACTGCTGTATTT

ATTGAATTTAGAGCTAGCGCAAAAAAAAAAA

>431

GGTTTGGTTGCCAGGGTTCCCCCTCCTTGGGGGGTTTGTTCCCATTTTGTTTTTGGGTGG

GGAGGAATACCTTGGCCCCCCCCAGGGGATAGCCCCTTTTTTTTCCGGGGTTCGGAGAAT

ATGGGGGATGTTTTCATTTTTTGTGCGGGGATGCCCCCAAGGTGGGGGAATGGATTTTTT

GTTTTACCCCCTCCCCCCCCAAAAAAAAAAA

>432

TGTTTTTGTGGGACATGGCCCTTGAAGTTTGAATATTTCAAAGTTTCAGGCAATGGGGGG

CTGTTAATAGGATTTTGCGGTGCTGTGGTTTAGCATGGACAGTTCATGCAACTCAAGGAC

TTTGGGGGGGGGCGTTGTAACGTGTGATGTTCCATTTGGGTAAGCATGTATTAAGTTGAC

ATACCTCATGTTTCCTTTGTAAAAAAAAAAA

>433

TCCAAACCCAATCTTTTAAACAGCAAGTAATCTCCTATTGAGAAGCAGCGAATGACAGCA

CATTCACAGGCACGTACCATTGGGGATTATGCCCTTAGAAACTACCAAACTTTGTGCTGC

TAGACAGTTCCTATGTGGCTACGGGACCAGCGTGAGATGCTCCCCATATCATATATATAA

GGGGATATGACGCCTCCCGGAAAAAAAAAAA

>434

GCCATCCCGTCGGTTCATGTGACCTCATGACAGAGAGCGCGGCATTCGAGGACTAGTGGA

AGAGCAATGCAGCAAATTGCGAAAACTATCAACCACAGCCTGAACCGTATTAAGTACTGT

TCTGGGACATCATTTGCACGCGCAGGTTAGTAACATGTTTGCGCATAAGCTAGTGGATTA

AATAACTATTCGGAGCCTTCAAAAAAAAAAA

>435

TTTGGATACGGATGTTAGCTGCTGGCCACTAGAAAGTTTTCTAACGCATACTTGTTGTTT

TTGAACGGCACAGGGAGGTTGTATAAACCCGCTGTCACTATGATGTGTAATTGGGATAGT

TGCATCCTCCCCCAAGTACGTGCTTGGTCGTGTTTGGAACGATCAACATCAACACCAGCA

TTGTAAAGTGTAGTTGGTCCAAAAAAAAAAA

>436

CGCAAATGCACCTGTTTTGCGGCTCAAGAGCGGAATCTCCGACCATGTGAGCATTTCAAG

ATTGTTTTCGGCCAGACGTGCGAAAATGCAAATCGCCGAACGAGACATTAGAACGCTTAC

ACGAGCCTAGTTGATTTTGATCGCCTTTGTACATTCATTTCACAGATATTTTCGATTTAC

GTATTTTGAGTGCACGCAGCAAAAAAAAAAA

>437

TGTGACTTTACGAGGGATCTGTGTACACAGACAGCCTGAGGCCGCATCAGTAATGACAAA

GTTCAGGCACTCCCCAGCTATCTACATAAAAACTAGACTCGGTCGAGGTGTCTGGGAATA

TGGCCTGCGAGCCTTGCTAGATGTGCTTTGATTACAATCATGCATCTGATACACCTGTAT

TGTAAATATCTCATCAGTGGAAAAAAAAAAA

>438

TGCACTCCAGAGGCGCAGCAGCAGATTTGTTTCCTGGCATGACCATTTTAGGTTTTGGGG

ACAAACTCAAGAGACATGACTTCACTTCAATGAACGCCCAACCGCTGGAATGATCTGTGT

AAATAGTGTGACATGAACAACATGTGAGGATTGTTTCAATTAGCAATTATTTGGTGTGCA

TCCCTTGAAGCACAAGTTCCAAAAAAAAAAA

>439

CACTGAAAGGGACCCATTTAGGCACTTGAGGAGCAAGTATCCTGCACCTATTGCGAGCAT

AAAACAATAATGAGAGTCTAGAGCGGACTGACGAACACCTAGTCCTTTTGTGATCCGGAT

TCTGCTGGAATGATCAAATTGCATTTATCTACGAACACAATCTAGAGTATAGAATGATGA

ATGACATGGTATTTAATTATAAAAAAAAAAA

>440

CGCTCGTAAAGTAGTTGGGTGCTCCTGCGTGGTGGTGACTGACTATGGAGAGGAGACTGA

GGGTCCCAATGTGCTCACCGAGTACCTAAAGAATCGTTAGATACTTTGTTGTACAAACAG

TCTTTACTTCTCATAGACTTTTCTCCTTCCAAAAAAGCACAAGGAAACCTTGGTATCAAA

GAGTTAAGCCTATGGTATGCAAAAAAAAAAA

>441

GGCGGATTGGACGTTTCACTCGTCAAGGTGCGTGTGGACAGTAACATGTTATAAGTTAGC

TTTTCCGCTCTCCAAAAAAAAAAA

>442

GCTGGGTTCCCGTTAAGGGGGGGGGGGTTTGGGCCCGAAGAAGAATTTTTTGGGCCCCTC

CAGAATTTTCCCAATGCCCCCGAAGCCCTGAAGGGGTTTGAAGGTTCCTTTCTTGGGGGG

GGAAGGCTTGGTTTTCCCCGAATACCATCCCTTGTTTTTTTGGGGAAACCTCCCCTTTTG

GCCGTTTTGGGAAAAAAAAAAA

>443

TTTTCCCCCCCCCTCCCCCCCCCCCCTTTTTTCCCCCCCCCCTCCCCCCCAAAATTTCCC

CCCCCCCCCCCCCTCCTCACCCCCCTATTTTCCCCCTTTTTTTCCCCCCCCCCCCCCCCC

CCAAACCCGCCCCCCCTTTTTTTTTTCCCCCCCCCTTTTCCCCCCCCCCTTTTCCCCCCC

CTCCCCCNAAAAACCCCCCCAAAAAAAAAAA

>444

GTCAAGCTCCTTTTCGCTGCCAATTTGTGACTTTCTGTGCCAACTTGGTTGAACTGATGC

TACTAGGGTTTCTGATGTTTTGATTGAACTGCTTCTGACTCTTTGGTGTTTTAAACGATC

TCCACTACCAGTCTGTATGTGCGGCGGACCGAGTCAAAGTGTTCTACCATTTGCACTACA

GCAACAATCTCAATATTAAGAAAAAAAAAAA

>445

CCGAATGTGAATTGCGTAGAAATAGCTTGGCAGCATGTTGATCGTGTGCTGGTATTTGAC

CATTTCGAGTAGATATGAAGCATTGCAAGTCAAGTCCCATCAGGGGATCATTGCAATGGC

TGAAAGCTCCCTGCTTGATTGGTGAGAGACAAGAGCTATGAATTGTCCATCACTGTTCCC

AATGATTTTAAATGTAAGTCAAAAAAAAAAA

>446

ATCAACGCAGCAGATGTCACTTGACTTTGCATCGCCTCGGCTACGGGCAATGGTTGACGC

AGTAAGGAAGCGTCTGGCGTGTCCATTTGCGTCATGTCCATTCGAGGCTGATGAAAAGAT

GATGCCGTTGACTTGTCAGGAATCCTCCGAAGTGTGGTAACAAGTATGATACCTCAACTT

CGTTACGTGAAAGCTTCTGCAAAAAAAAAAA

>447

GTTTCTTCCCTGATATTTTTGCAAGTGCCCGGCCGTCGTTGAGTTGCCAGGGGACCTTTC

GGATTTGTATGTATATATGGGAAGTTACCCTGGGAAGGGGGGGTTTAGTAGTTTAGCCTT

TGCTTAAGGGGGGGGAAGAGGGATGATTCCGCCAAAACGGGGGTAAACAAGGGGGTGGGG

CGCCGAATTAGAACGCTGTTAAAAAAAAAAA

>448

GGGACCTTGGACTATTTACCTTAGAACTTTACGACACACACACAGGGGGGTGACTGTGTG

GCGTAATATTATCTATGATCTATTGCGTAGTCATAGGTAATATTAGTATATAAAAAAAAA

AAAAATCACGATAGTAGCAGCATTCACTGTTACAAGATAATGAAGCCCATTTGTCCCCCG

TTGGGGATAGCGCGCAGGTGAAAAAAAAAAA

>449

GATTCAAAAAAAAATAGGCACTTGTAGACAACCTGACACATGTGCGTTCTGTATACAAAT

TATGCGACATATTGTACATGGGAGCGTTCGTATTGTGAGATATGTAATGAGTTCTTTTTT

TATACATTTTCAGATTTTTTCGTATGTGGAATACCGTCAAAATATCACCATTGACGGTAA

TCCACATACAAAAAAATCTGAAAAAAAAAAA

>450

AAAGAGCTTGCCGCCGCCTATGCTGCACTGATGTCATCCAAGGAGGAATCTATGAAGAAC

CTTGATGGCCAGGTTGGGAAGCTTAGCAACCAGATCGTGGAGAAGGTCATCCCCGCATAA

ACTCGGCGTAGCTGCTGTTTATAACTAGAAGTAAACATTACGTACGCATAACTTATATTT

GTAACCCTATTATTTACCCCAAAAAAAAAAA

>451

CAAAGTATTCATCCAAACATCCATAGTATGTCGGATCGAGCTTTCAGGTCATCAATAGAC

GGTCCATCCTCGCATATACCGCTTCCATTTCCAGATCATAGACTTCCTATAGGAAGACAA

ATGTACCATCACACACAACCAGCAACACATCCTCCACTATGATGTGAACTTTGTGTAATT

AATTATTTGACCATCGCATCAAAAAAAAAAA

>452

CAGATTTTTTCTATTGATTTCTTTTTTGAGTTTTTCCCCCTTCCCCCCCAAAAGGAACAA

AAAATCTTCAAAAAAAAAAA

>453

CCACCGCGCCGCCTGCGCGTCCGTCGCAATCAACCCCTTCGGCTCAATCTCTAAACACGC

AGGCACGCTTTCGCACCTCCACACCTAACAAACGCTCATTCCGCAATTTCTCTAGCACTC

AACGACGGAAGCAACTCACCAAGCAAGGTTCCCCGGTGACGTCAACAAGAGCTTCGTCAC

GCGTCGGCCAAAGATAACAGAAAAAAAAAAA

>454

ATGGCATGCACCAAATCATGGCCACGGTCAGCGATGATGTGAAATCATAACATTGACAAC

CCCGCTTTGGCGGGTGGGCTACGTAGCAGCTACATGGGAGGGCCAGGCTCTTTCAACAAT

GACGTGCGATGCCACAAGTTAGCCTTATCATAAGTTGGCCAACCAATAGTGATGCACATA

ATTGCATACATTGATCCGGTAAAAAAAAAAA

>455

GGTGGTGCATAACGACGTAATCTGAATAGGGCATATCCCAAATGGTTGCAGGTAATCATG

TGCATGACGTTTGGTGATGTGCAGAGAGCGTTAGCCAACTCCTAAATCGCCCAGACATGG

CTTGAGTAAGACAGAGGTCATACATCCTTAAGATTCTTCAGCCGTATGATAAATGTATGT

GCAAGATTTCCCTATGGGCCAAAAAAAAAAA

>456

GTTTGGCAAGGGGGTTCCAATTTTGCCTATGCTGTTTCCCTTAGGATCCATAGCCTTTCC

GTCCCCTAGAAGGGTACGGGGCCCCCCTGAAGGGGAACGGGCCCGAACCTGGTAAGTGAA

AGGTTTATCAATGAATCCGCCCCATTGTTTAAATCCTTGTTGGCTTGCCTTGTCCCATGT

TAACGCCGTCAAATCCAGGGAAAAAAAAAAA

>457

GTATTGGTCAGGCAGAAACCTCCCAACTAGGGATATAACCCAGCATCCCATTGGTTTGAT

TCCGGCGTTTTTCCCACCAAGCCTTCAGGAACGGGCTATTATGTGCTGTAAGCGTGCCGC

TTGCGAGCACCTGTTCCGCCCGAATTGTGGCATCACAACCTTCAGGGCAATATTTCAAAG

GATAAGCTACCCCCCTGTGCAAAAAAAAAAA

>458

GACCATATCGCTCGCTTGTTTCTGCAGTAGGAACCTAATGAATCGGTCCTCCACTAAGAC

CAAGTACACTGGCCCTGCTGGAGTGAACAAGACTTGCTCTGTTATTGTGTGAGGAAGGAG

GTGTAGCTTGGTCGCAAAATGTAATAATCGCCATCGTGACACATTGTGCTTTCAGATTTA

CCAAAGCTGATTTTTTGTCCAAAAAAAAAAA

>459

CTCGTGGACCGGTAACAGCTCCACCGCGTTGACGCCCAGTTCTTTTAGATGCTCAATCTT

GCTTGCGAGGCCTAGGTAGGAGCCGCTCAAGGGCCCAAGCTTGCTGGATTCGTGTGCAGC

GAAACCCGCGCACGGCCACTTCGTATACTATCAGGTCCTGGAACGGGATGTTGGGTTTCT

GATAGTTTGCGCCCCAGTCGAAAAAAAAAAA

>460

AGAAATGTGTCTAGCCATAGCACATGATGCATGCTTCTGCCATTTCAATGGTCACTGTGC

ATTTGCGCCTAGCATTATGCAGCTGTGATTCTGTCACGGACACAGCACAACAGTTCTCAA

CCCTGGATGCTCGTTACTTCAGATTTAAGTTAGCTGAAGTATACGGGTCCGAATGTTGAA

TATGCCTTCTGTATTGCTTGAAAAAAAAAAA

>461

TCGAAGACTGGAAAGTAAAGCTTGTCGGAAGCTTGGATCACGACAACTACGGCAAGTAAG

TTGTCAAGATAAGACAACTAGACCGCAATAATTGTGTCAATGGTCAGCAAATCGGTGGTA

GTAGTAGTAGTAGTAGTAGTAGTAGTAGTACATCAAGATATGTGGTAAGAACTGTTATTG

GAATGTAACAACACAACAAGAAAAAAAAAAA

>462

CATAGTGGTCACCGGCGTGAATGGAACCGGGCAACGAAAACATACCACACGTGCCGAGCT

TGTTCTCGATCGGCGGGTAATCTCCCCCCATGAGGACGAATGACGGTAGGTGTCACCTAT

TAGGTGCGATGGTTTGGACGGGGCTGCGACTCAGAGAGGCAAGCCTCGGGAGGGATGCGG

GAAAAAGCTGCTGCGGGGCCAAAAAAAAAAA

>463

TCGTGGTGCATAGGAACCAACTCTTTGGTCCAGTTGCTCCAGAGACTGTGCGGCCCAGTT

CAATATAGACCCCGTTGCACAAATATGAGTTGATATCAATTTTCCGTGGATATGATGTTC

TGCCCTCGTTTCAGGTGTATGCACTTGCAAACCGAACTCTCAAGGATCGGGTTGTCGTGG

TGTTGGAGCGGTCGGTCGCGAAAAAAAAAAA

>464

CTGACATACAACCGCAAGCGTCAGGCCAAAATTACTGGCGAACTCATTGAGCTGGTTGCT

GGAAGCTCTATGTAAACGCCGCGAGACCCAGATACGATGGAGGAGTTTTCCTCCTAGATA

AAAAGGCATTTTTAGTATAACTTACATTTTAGTATTCCATATTCGACATCGTTGTTAAGA

TTAGATTATTGTCTAAGCTTAAAAAAAAAAA

>465

CATCAGTGTTAAGCCGACCAGTGCTACATGGAGACTAACAAGAGCGTACCTTAGTTCTAT

TTTCAAGCATTTACGCCACAAAAGTTGGCGCTCGGTTTAAGTATTTATGAGATACGATCG

GAACGATGTGCATGCCTTGTATAAGATATGTATATATAGGGACATGACACTTGTATCAGA

TATAGTGATGCCTCGTCGATAAAAAAAAAAA

>466

TCGCCAGAATTGAAAATACAACACCGCGCTGTGTGGTGTTGACCTTCAGTTTCAGATAAC

AAAAATCAATAGATTTAGGTTCCGTGATGCAGTGAATCCAGAATTTGTGGAATCTGTTGC

ACATTAGGACATGGTGCTACGTGACATCCGATGTTTTGTCATACGTATTTGCGTATAAAA

AAAAACTTGTGGCGCATATCAAAAAAAAAAA

>467

CTACGCACTAGGGGTTGAAATGTGGACACTTGACTTGGTAAAAGGAAGTTTCGGACCACT

ACATGTTTTGAATACCTTTCAAGTTTGAGATACCGTAGGACGCCGGGCTTGGAGGCTTCA

ATGCACGCAAGAACCTTCTTGATATACAAAAAGCGTGTCTATCGGAACGAGAGCCGACTA

AAATTCGACTGAAACGTTCGAAAAAAAAAAA

>468

TGTTGTGGTTGCTGCAAGATCCTGCCATAGTTAGGAGATTTTTTCCCTGCAGTGCAAGGT

GACACTCAAGTTTAGAGTCACTTTCAGGACCCGTTATCAAGCGGGATACATGTGAGCCTT

GATGGGTTAGCAGATTTTCCCAGAAATATGTGACATGGACAATGCCTTGGGAGGGGGTAA

ATATCACATCGTTTTTTAAGAAAAAAAAAAA

>469

CCTTGATTGGTTTTTGGGCCGGGGGGCTTTTTCCCCCACCGTACCTAGTTTTTTTTGAAA

GGGAACCCCCCCCAATTTTGGGCTTGGAAGTTTGGCCCCTTGGGTAATTTGGGGGGGGGG

TTCCAGAAAATGCTTTCCCTTTGGGGGTTGGGGCCCTGGGAAAATTCCTTCCCGGTTTTA

TTTTTAAAAGTTGGCCTGGGAAAAAAAAAAA

>470

AAGCATGGGTGGTGTTTCGTTAGCACCCCCAATGTTTTTGAACAAATTCAAGGTCTTTTC

AGAAGCTCACTGTGTCAGGGTTCCCCCCTGCCGTTATTTTGCAACTGATGTGACGATCTG

TATTGGAATAATCGAAACCTTATAACTTGAAAGAAAGAAACAATCTTTCAATGAAGCTGC

GGCGATGAACACTTCTCTTGAAAAAAAAAAA

>471

AAACAGATCCCCTGTTGAGTTGCTTGTTCTGTTTGTTATAACGATATCTGCTCTGCATCT

GTATCGTCTTGCGTTTCGTTCTCTACTTACCAAACGCTGAGCATCATTCGTACAGTGACG

CCCTGCGAAACTCAGCCCCAGATATTGTGGACCAGAAACGCGATATGTGGTATTCAATAA

AACAATAATACGGAAATACTAAAAAAAAAAA

>472

GGCCAGTGAACACGCAAGCAGCACAGATCCCATTCCGCTATTTTCGGCTGCTTCTTCATG

GCTCGCCCTGTCAAGACATGTCGAACCTTCGTTTGTGCGTTAGTGCTCTGGAATTGTATG

GCACATTGGAGTATGAAATAGCAGACATTGAGTGCTTTGTAAGGAGTGAGTAAGTTGATT

GTGATTGGCCCTGATTTCCGAAAAAAAAAAA

>473

GTGTGCCTCCGCCTCCGCGCCGCTCGCCGACTCCCACATCAACACGGCGGTGGCAGCTGC

AGCCGCCTTGTCCTTGAGCTCCGCATCCAGCTCCTTGCTCTTGCGTTGCAGCAGCTCCGC

CTCGGACTGCGTGCACTCTGCCTGGAAGCGCGCCTCGCTCACCTGCGACACCGCGGCAAT

CTCTGCACGCGCCTTGGTGGAAAAAAAAAAA

>474

ACATGCGCTGTTGTACCCAATCAAGTACAATACCAGGACGTGGCCAGGAATTGAGTTTTA

GTCAAATGGCATCAGAGCGCTCAACAAGTTTTTTGGTATGATTCGTGCTTGTGAACTGAT

TTTATCAGATGCCCCAAAAAAAAAAAAAAAAAAAATCGTAGTCGCAGCATTCACAGAATG

AATAATGTATCATATCATATAAAAAAAAAAA

>475

GAGTAGAACAGGACCAATGGTGGAGAACAGGAGTGACGTGTAGGTGGCGATGAGAAGTTA

GGTAATACTCAGTGCATTCTGCAAAAAGCTAAGTTACTTGTCTTCTCTGTAGGTTGTGCA

AAATAATCTTGAGGCCATTTCACCAGGTACTGTGTATCCCCGATGTACACGTTCTTAATT

GGGATATAACCGCTTCGAGCAAAAAAAAAAA

>476

GACATTGGAGCAGCAGGAAATCATGTAGTTGTTTCAAAATGATCCTGAAGCAATGCTTGA

CAGATTTTGGTGAGGAAAGAATTGCAAGTTCTCTGTTGACAGCTGAATGTTTTCGGTCTG

CAATAGGCATGTCTTTTCTTTCTTACACTACGCATCCATCATATGCAACAAAATTAATGA

AGTTCTTGATGCAACGTTCGAAAAAAAAAAA

>477

ATGTCGGTGTGTCCGTCGATGGAGATAGAGTTGTCATCGTACATGCAGATCAATTTGCCC

AGACCCCAATGACCAGCCAGGGAGGCGGCCTCAGTAGAGATGCCCTCCATGTTGCAGCCG

TCGCCGAGGATGCAATACGTGTAGTGATCGACGATGGTGGCGTCGGGCTTATTGAAGAAG

GCTGCCAAGTGCTTTTCGGCAAAAAAAAAAA

>478

GTGTGTTCAATGGGCATTGGTGTGGGGTGTTGCTATTTGAAGCTTTATATGTGACGCCCT

ATGATAATTGTGTTCAATCGGGTATGTGTTTGCAACTCCCCGCGGACTATAACCGCTTAA

CCTTCAATGTGAGGGTTCGGTTTTCAAGGGTACCGTGTGTCAAATACAAAGTCCTGACGT

TAAACTAAATCGGCTTTGTTAAAAAAAAAAA

>479

CTGGCAGATTGCGCTAGCACATTGTACCAACAGTGATTCGCCTTATGCTTTCAAATTTTC

GCAAGGGGAGCCGATTCATTTATTTTATTTATTTTCCAACGAGTTTCAGTTAAACAGCAA

ATTGACTAGACTGTGTATATTGTATTTCATGCACCTGAACGCACATTTGCAGGTCAATAT

CATAAGATAGTCACTCTTTCAAAAAAAAAAA

>480

AATGCTTGCCCAGAAGAAAATTGCCAACAATGATGCTGCGATTGCGTAGGTACAGGCACT

CTAGCATAATGTGATGTGATGAGCATTTTTATGTGGCATTTCGGTTCTTTCGAATGCAGA

AATGATGGTACTTTGAAGAATTTCGTGATTGCTACACTATCCCGCCCTGTATTCAAAGTG

ATGGAATAGTTGGTCATACTAAAAAAAAAAA

>481

TCCAGCTCCCCAACCATTTCCGAATCGGTGGCTTCGACCTCGTCACTGGATCCTTTGAAG

CGTTTTGTGGATGTGCATTCTTGTTCGATGGTTTGGTTTGATTCAAAGTCTTGCACATGT

TCCTCGTTGTGGAGCTTGACGGGGGAAACATCTTCCGCGGCTTGGCCTATTGAGACCAAA

ACAGGTGCTGCAGCCCTCGGAAAAAAAAAAA

>482

ACCAACAATCCAAGTTGCGGAAACACATGCTTGAGATGCATATAACACGGTTCTTTGCAA

CTTAGCACACACACAATAACATATTGGATCAAGCTCCCTTTTCGGCATCGCAGTATTCGT

AGGTATTGCACTTTGCATCGCAGCATGCGTGCAAGTGAATGTTACAGTGTTTATTCTGAA

AGGTGATACCTATCCGTGTTAAAAAAAAAAA

>483

CAACAGCTTGCTGACGGGTTTGGCCTGATCCTTTTTCCACCCGTGCGCTGCAGTAGCCGA

GGTGATCACCGCTGCGAGCGTACCGCCTGCCCCCATCTTCAGCTTCTTGCCCCCTGTGAC

AAGCATGAGTGTGAAGCCCAACAAAAGCATACTGCGAAGCCGAGTCGCGTCTTCGCAGAC

TGGATCAATATCAAGCCTCCAAAAAAAAAAA

>484

TAATGGCACTGGGAGCCTGTCAGACTGAGCATTTAACATATTGCTGGGAATTGCGATCAG

AACTCCATGCGCAATGAACAGCATGATTATCCACAACACCTTGATGTTAGCGAGTGTGAA

ACGTTCAGGGTCCACTGTGTAGCAATGTGGATCCACTAAGAAAACATTGCAACTGCACTA

AAGTTATGTCTGGACACTTGAAAAAAAAAAA

>485

CTCCGCAATAACACCGTGTAAAAGAAGCTTTACAGGTGGACATACAAAAACCAGCAGAGG

GGTCGGCGTGTGCGTGTTCCCCCTACCAAAAGTTGTCATCGTTGCGGAGAGTCAAAACGC

TGGAGCTAAATTCGCGGGTTTTATTCACAAACAAGTTGTAGCCGTACCCAGCCGCGTGAA

GACTTGTAATAGGCAAGTCGAAAAAAAAAAA

>486

AAATGATAATTTCTTCTGGACAGACCCTCGGGTAGAGGTTGTTGTTTCCAAAGCCAAGGA

TGGTTGTGTGTAGAGCACCTGGTTACATGAAGCCTACAGCGTGAGACTGCTTAAGGTCCC

GGTCCAGGATCATCATGTTCATGGAGGGCAGTCAGCGAACTCGCTGGATTCTTTTCCAAT

ACTGCGATAGACGAATCCCGAAAAAAAAAAA

>487

TTGGCATCGTGGTTACGATCGTGATTTCACGATGAATGCACTATCACGACTATTTTGTTA

TTTGTGGCTCCAGGATGACAAGAGTGCGAGTCACCAGTTGTATAGCTTCTCATGTGCGCA

ACTTGGGATTCACCTGCTGGGACGTGGTTGCACTAAATCGTCCAAGTGACAATGATATTA

TGTCAACAATTTCAACACCCAAAAAAAAAAA

>488

TTTTGGGTTTTCGTTTACAGGATACGACATTTGTNCAAAAAAAAAAA

>489

CCCCAGCTCAAACGTAACCGCGGACCAAACTTTGATGACCCCTTCGTTGCCTGAAGTGAT

GAGCGTGCCTTTGTAGCGATCGTGAATCAGGCAAAGTACCTCACTCTGGCTGTGTGCTTC

GAACATTGAAACCAAATGCGGGCGCATTGAGTGGCGCTTTCGGACATCCCAAATACCTAC

TCAAAGGTCGAAACCTCCCGAAAAAAAAAAA

>490

TCAGATGGAGCGTGAAGCGCGGCGGCATCTCCAGCCCATAATTCCGATCCGTCCAGGCGC

AAGGCCCCACCACAGAGCCTTGGCGGAGGGAGCACTGTTGACTCAAGTCGCACTCTTGCC

CAAAGACGCACCGGTCCGTTTGCAGCGTCACCTTGCGCGCGTCCCCTACCAGGGTCAAAA

GATTATTGCGGGTGGGCCCGAAAAAAAAAAA

>491

TTTTTTTCCAATGGGGGAAAATTTCCCGGTTTTCAAAACCGGCCAAATTTTTTTTTTGGG

GATCCTTGGGCCCCCTTTTGGGCCCCGTTAATGGCCTTCCGAAGGTTTTTTTTTTTTTTC

CCGCCCCCCCCCTTTTGGGGGGGGGGCCCCCCCGGGGGTTTTTTTTTTTTTTTTTTTCCT

TCGGTTTCCCCCGTCCCCCCAAAAAAAAAAA

>492

TGGCAGCTTCCTTGTTCCTCTGTTCGTCCGTGACAAGCACGCCTTGCCCCTCCTTCTTCA

CAGAGACATCTGTATTGACCTCGGGGGCCGGCTGGGCCGTGAGCTGTTTCCAGAAATCCT

CCGCGGAGGGCGCACCTTCTGCAGAAGCAGCTCCACCTTCGTTCGCCTTTATTTTTTTGA

AGGAGGGGGAGGTCTCCAGCAAAAAAAAAAA

>493

AGAGATGGGTACGAGAGTATTGCAGTGCCTTGCCATGCCCCCCCTTTTGCAGAAGTTGTT

CAAAATGAAGACGGTGCAGGTGAAACTTGAAGTGCCCCAAACTGTCAAAGTGTAGAGCCT

GCTTCCTGAGTTCTTTGGTCCAATCAATCGCGGGATTCAAATTTTTTGGGTCAATTTGTT

GTAAGATACTGTACATCTCCAAAAAAAAAAA

>494

CAGGGACACCATTTTCAGTGGTGATATACGTGGGGGAGGATTCCCGGGAATTTGGACATT

GTTCACAACAACACCCGGGAGATCCTGGGGGTTTTTAGAGATCCAGGATGGGGCTGGCAA

AAAAAAAAA

>495

GGCCCCGCGAGAAGTGGCAAAAGAGCCGAAGGTGGATGGCCCAGTTCATAAGCAACATCC

GTTGTGTTCAGGTGTTGGAACATGTAGGGACAGTGAGTACAAGAAGCGTGTTCAGCTTCA

ATCGCTCAAGGCAGAAGATGGGACATGCCAAAGTGAACTACAAAAACCCTGAAGTGAATA

AAATTTTCCGAGTGCGACCTAAAAAAAAAAA

>496

ACCGCAACAAACAGTTTCAGGTAGCTCCAATCGTAAAATCAGGAGGGGCAGAAAAAAAAT

CGGGTAGGGGTGTGCAAAAAAAAAAA

>497

TTTCGTAGTTTTCCAGCAGGCAATTTCCATGAGACAGCTGTGAATGCCAGTAGGTTTTTA

CTGCGGTCACTGGGATCTTCGCCAGTTCATTGCGATCGGTTGATTTGCTCGAACGTTTCC

ATTGTTGAAAAATTTCGGTATGCTGGTGACTCTTCACATATTGTATAAAATCATGCCGGT

ACCATGCTCGTTCTGTTGCTAAAAAAAAAAA

>498

AAGTGCATCACTCTAGAGTGACTTGAAGTGCTGCAGTAGAACCGAACCTCTGCCACGTCG

TCCGCGCTGGTGTATCCGAGAGCTGGGCCGAGATGCCAGTGAGAGTATCCGGTGGTCGGA

GACGTGGGCAAGTGGGATCTGTCCAGTGCAAGATTGGTAGTCTCCTGCAAGAAAAATGGC

ATGGGTAGGTAACTGGACTTAAAAAAAAAAA

>499

CTTAATATGTATCTAGGCAATCTGAATACTTAGTGGTTCGTAGCATTGAGAATGTGCCAA

TAGACCTAGCAATAGAACCAATCACTCTTTAGTGTAAATCAATGACCGGGTATCGCTGAT

GACGACTGAGTATATAGTCGACGTCATCAGCGTACTCGGCACTCTTTGAATACCAAGCTA

CCGCCCCCGTGCGTGCCCGGAAAAAAAAAAA

>500

GTAAGACCCAGCATGAGATGTGTATAGAATCCATGACTGGCGTTCACACTCAACGAGCTG

TCAGCGACTTTGTGCAGATCTCATAAATGGTTTGGGTATGCAATAGATCGCAGAAGCGTG

ATGGGTAATAAGCAGTGGCCCATTTTCGTAGCAACAGAAGGATGAAATAGATGTTTTGAA

TATAACATATTGCTAGTTTTAAAAAAAAAAA

>501

GGCCCCAAACACAAGGGGGGGGGGAAAAACCCCCAAAAGGTTTCCCTTTGGTTTCCCCGA

AAAAATCCCGGGAAAAATTTTTGGGGGGGTTTTTTTCCCAAAAAAAAAAA

>502

TTTGTGGATGGAATCAATCTGTTTTCGCTATGTGGTGTCGTGGTTGCACTCACCACCAAG

TTTTTCACTGCCTCGGAATTACACGCTCACACCCAGGTGCAGCAGACTGACTGTTGATCA

TTTTAGTGGTCCGTCTGCGGATCCACCAACAAGTGTGTGAGTCCTAAATAGCGCATTAAT

TGTATAAATACCTATTGGTCAAAAAAAAAAA

>503

CAGACGACCTGCCGCCTCTTGAGGAGGATGTGGATGANGGATCTCAAATGGAAGAGGTAG

ACTGACGTGGATCGAGGTANAATAAGCAACAGATTTCTTTATATCTTGTTGAAGGTTTAA

ACATGTCAAACAAACCGTACAGATTTTGTCTCANTATGGTGTTTTGTAGATGGTTATACC

CCAATATCTTGTTCGCACATAAAAAAAAAAA

>504

AGGCCAATTGGGGGGGGTTGAAGATCAATTTTTGGGGAAAGCCCCCGGGTTTTTGATTTT

CAACGTTGTCTTTTTTTGCCAAGGCAGTTGGGGCTTTTTGGAACCAGGGTGTTTTTGTAG

TTTTCCCACCCTGGGGGCCCAAACAAAAACAATTGTTAAATCCATTGGGTCCAAATTTTT

GGGGGGAATTTTCGTGGGGCAAAAAAAAAAA

>505

CTTGGTCTGGCCACCAAATCCACTCTGCTTGCGATCGTAGTCGCAGCATTCACAGAGGCC

TTGCCAGTCTTGTACTGGGTCACCTTATGCATCTGATGCTTTTTGCATTGCTTGCCCTTG

CAGTAAGATTTCTTCGTCTTGGGAATGTTCACCATTTTGGCTGGGGGACGGCGGCGGCGG

CTGGAACGGCCGGGTGGGGCAAAAAAAAAAA

>506

CGGGTCTTCACACAGCTTGCAAAACATAGGACGGAGCAGATTGTATTCCGGATATCGAGC

GTAGATAATTAAGTATCAACTAGACTTGATTGTTAACGCAGATAATGATCAAGCTTAGGT

AGCTTGCCTTACCAATTGGTCAAGTCGAATATGGCACGGTGTGGATGTAATATGTATTGT

GTCATTCGGTCCGACTTAACAAAAAAAAAAA

>507

TGGGTGAGTTGTTGAAATCATACCAACAATCATGTCTTGTAGAATAATGCTTTTGACACC

GATGATTTCGGCATTCATTTGAACCAGTGTGGGACCAGTTGATACCTTGTACATGGCACA

ACCTTGTCGACTGCTGATGCGTGCTAAATGGTTGGGGAATATCATGAACAACCTTTTGCA

ACTCTCGATCGAATATGCCCAAAAAAAAAAA

>508

ATTTGGAGGGGGGGTGGGACCAACCCCCCTGTTAGGGGGGGGGGGTTTGGGCCCGGAGGA

GGTTTTTTTGGACCCCCTCAAATTTTTCCCCAACCCCCCGGAACTTTTGAGGGGGTTGGA

GGTTTCTTTTTTGGGGGGGGAAGGATTTTTTTTCCCGGAATCCAACCCTTGTTTTTTTGG

GGAAATTTGGGGGTTTGGGCAAAAAAAAAAA

>509

ACAAGAGGCCCCGCACGAAGGAATCGCTGTAGATCATTGAGATGAGTCCATGAGTATAGT

ACCCGTTGTTTCCATGTTCAGGATTTCTTACCTTCGTAGATATCTTCACTAGTAAAAACC

AATACGTTGCTTATATTGGACATGAATACGATGCCAGTATGCGGAAGAACAAACCACGTA

CGGTGGTTGCTGTTGCGAACAAAAAAAAAAA

>510

TTTCCAGGGACTCGTGGAGTTTACTGCAGAACTGAGAAAAACTGTCAGGTACTATGCCTG

CGCTCAACCCTCGATGTATGTCGTTGCTGTACATCCAGCATGAAACCCATACCACCCATC

CCTTCAGGCAAACATAGTAAGTCCTATTTGTGGAACAACATGAAAAATCAACTTTTTCAC

TCTCATGAAAGCAACTCCTGAAAAAAAAAAA

>511

TTAGCCAGCAACGGCTAGTACACAATTTGGACGGGTTCACTGGGACCCGTCGTGGTTGTA

GAATTTAAGCCATTTGTATGGCAGAATCCCTGCCAGTACCCAGTGTTAGCGAAGGCTCAA

CAAAATACTTCGATTCCAATTCCAACAGCATTGGTCTTTGGGTGTATTGGTTATGGTTCA

CGGAAGTAACTCGATATCTTAAAAAAAAAAA

>512

TGTTTTCCAGGATATGTCAGACTTGGCGGGTTATCCGCAAAACAAAATCATTTGCTTCAA

TCATCAACGGCTCCGTAAACACACACACACACACATTGTTTTCATTGGGTAGCGTGTGTG

TCAAAGTATCTACATTAGGTCAGTTTGCGATCGGGTTATTCGTTGTGAGAAAATACCTTC

AGCCCAAGCCTTAATCCCGGAAAAAAAAAAA

>513

TTGACTAGCGCTCACACCTGTCTACCGCTGTCAGCAATCGTCTGCAAAGGAACTTCGCAT

TTGACGACGATCCAAGCACTTGTTCTTTGCAAGTTACGTGTCAAGTCGATCGTTGGCACT

CTAGTTGTTGTGGACAACGTGCTAGAATGTCCTTGTATCAAACAAAACGCACACCTTTAT

ATATAATGGGATATGACGCCAAAAAAAAAAA

>514

GGTTGACTTTTCAGAGACACTATCAAACCACTTGGCACAGATGGATGGGAAGTGAACACG

CGACGTGAGAACAGAAGGTAGTAAAAGCAAAAAAATTGTGAAAACAAACGAGGACACGAT

ATTGTAGTTAGTGCAGGCGCGCCCGTTGTTCGTGAAGCAATCGCGTGCCTGCGAGGCGCT

GCAAGGTTGACTACTTCGACAAAAAAAAAAA

>515

CCAAACCGTGGTGGAAAATTTGTCGTGTGACGCTTGAGTGATGACTACCCCCCAAGTGTG

CGTGTCGCACATAAGGGATGAGTTCACGATTCGTGTTGACTTGCAGCCGACCATGTGCAT

TTGCTACATCTGGCACGACAGTTGTCGCAAAATGCATATATGTTCGTGATATATAGAAAT

ACCTTATGGGCATTGTTGTCAAAAAAAAAAA

>516

TAGTTAACGGTTCTTTAATTGCGTGTAGGTAAATTCGTCCTGCAAAGATTATCAACATTG

GTGCACATGCTATAGGGTATCTACATTCCTCAACGTTGATTGTATGGTGATATGATGAAT

GCAAACACGGATGCTAGATACCCTTTCAAATTANCACTGTAATGTATGATTAGTGCTCAA

ATGGCATCATTGAGCAGCATAAAAAAAAAAA

>517

GGACAAGGACCCTATTGCGTCCCACTGTGTGTTGTCAATAAATAATTCACTCATAACCCC

GGCTTCCCGCACAGGTTATTTATCAGCAACAAATCATTGTTGCATATCAATATGCACATG

ATGACATTCAAAGCGCAACAGAAAGCGCAAATCGAGTACATACCTTGGAAAATACCCACT

GCCTCAAAGAACAATTTCTGAAAAAAAAAAA

>518

TCGATCCCCTTGCCGGGGGGATTTTTGGGTTTAAAGGCTTTCGGGGCCTTTTTTTGTCAA

ATTTTCCATGGTTTTTCCCCCTGGGGTCTGGAAAAAAAAAAA

>519

CACAGGGTTTTGAGTATCAATTCATAAAGTTGAGAGAAAAGTCAGGCACACGTGTGGAAG

CCATGCCATCACATAGTTATGCTCAATGCTTTTCAACTGTGCCCGAATACTTGAATCCAA

GTTCTTTTCATGGTGTTTTCGCTAGAACCTTAATCATCCACAAGTATTCTTCAACATCAA

ATGTATTGGTTTATCCTGGGAAAAAAAAAAA

>520

TTCAAGAGTGAACAATACACTGTTCCAGCTTCCAGCATTTGCTTCAAGTTATGCTGTGCG

AAGCTTCGCCCTTGGATACTTCTGTATCCAGGGATACTTTCCTGCATGTTAATTGTATTT

GCGCGTAGATCACAATTTTATCACTTATGACACAAATATTTATTGTACATGTCGCTCATC

ACGACCATGCGGCTGGTTTCAAAAAAAAAAA

>521

CAGCTGCCTTTGATAGCCAGATGTTTATCTATTTGAAGTTGTCATTTTTTCCGAGCAACA

TGGAGGCGCATAGCTTGTTTTTATTTGCTAGGATGGAAACACCTCAAAGGCGGTTCACAG

TGTATCACCTTAGGGGTGAAAGAGATTGTACTTGCAGTACTGGTACAGAATATCATGGCT

TCAAGTGTTAGTGCAATACTAAAAAAAAAAA

>522

CCATACAAAAAAAAGGTCAAAAACATACCGATGCACATGACAACCTTCATCAGGTCAACT

TTGTGTCAGGTGAAAAAATACCAGGTGTAGGATCCAAGGTGAAGACATCTGATCATCCTT

ATCCAGTAGCCCGCTGATCTCGCAAACCCGATGTTGTATTGAAATACGCCGTGTGCTTTA

TTAAAAGCCAGATCCCGGTGAAAAAAAAAAA

>523

AATGTTTGTATAAGCTCTTGTATGCGTAACTAATAGGATGCGCGCCTGGATTATTGTAAG

ATCTCGCCATTAATCTGTGTTTTCGAAGTGGTTCCCCCGACCGCGAATGCTTGTTCCGGC

TTCTGTTTCCAAAGGGTAGACTGTGAGGTGGAACGTGTCATAAGTCCGAATTCGGGTAAT

ATTCAGTTCAAGCTTCTCGTAAAAAAAAAAA

>524

TGTTTGAAGGGTTCATGGCCACTTGGTTTTTGGCGGCATCTCCAATCAGGCGCTCAGTGT

CAGTGAAACCCACATACGAGGGGGTGGTACGGTTTCCCTGGTCGTTGGCAATGATCTCAA

CACGGTCGTGTTGCCACACACCAACGCACGAGTACGTCGTACCCAAGTCGATTCCAACGG

CAGCCATGATCGTTCGGTCGAAAAAAAAAAA

>525

GAGATTGATGTTGTTCGTTTGAAGACGCAGCACAACCTCTTGGATGATTGTACAGCCAAC

AGGTCGCTTACATGGAGGGCAACCACAGGGAGCAGAATGATGAATCCTTGTTCCCTTGGA

ATGGCGAAATCACTACAGATATGCTGAGTGTTTTCTCTTGATGTATTGAAAACTCTTGAA

ATTCACAAACAATTTTCCCCAAAAAAAAAAA

>526

GGAAATATAGGACACCAATGGAATGTTTCTCCTTGTTGTTGGCAGGGATGGCAATGTCCA

CATGCTTCAGGGGGGAGTCGGTGTCGCAGAACGCAATCGTGGGGATATTCACGTAGGCGG

ACTCACGGATGGGCTGGTGGTCAGTACGAGGGTCGGTCAGGATCAGAAGGCGAGGCTCCT

CGAAATTGTCCTGAATCTGGAAAAAAAAAAA

>527

TTTCATGATGTCGTACACTCCACTGCACTTGCCAACTTCGGCTCCAGAGTCTGATGCATC

AGGAGCAAAGCCAGCGTTTCGACATATGTGACCACTTGTGTGGCTCGAGACGGTTGTTTA

GGTAAAGCAAGACTTTTGAGGCTGACAGCATTTGTTTCATCTATGAAATGGGGTGTTCTC

TCATTTGTTTAACATTTGGTAAAAAAAAAAA

>528

TTTAGAAGGTAAATAAATACGTACCCCCCCCTTCCCCCCAAAAAAAAAAAATGAAAACCC

AGCTGGCCTTTTAATGTGAAATGAAAAAAGGAAAAGTTTGCGATTCACTACAAGAACCAC

AAAAACTGGCGGACGGGCGGCTCAGAGATGCAGGCGGTTAGGGCTCCAGGTGCATCAAAA

GCACTTTAATATTAAATCCCAAAAAAAAAAA

>529

CAAACACCCGATCGTTGGTATCAGAACCAGACGCCTCACTCCAAGCTGGTCGAAGGTATG

GTGCTAATGGAGGTTGTGTGCACGAGTGCATCCAGACTCTCAGTGGTGGCTATCTGGCGG

TGTCAACGGGGTCCGTTACTTTGTGATATGTGTAATGTACCCAGAAGCCCTGATCATCAA

CACAAAAACATTGGATCCACAAAAAAAAAAA

>530

AGTCCCGCCAAGCGTCCGCAAAATCACAAGACACCAAAAAAAGAAGAACTCAAAAGGGAA

AAAAAAAAA

>531

TCGGTTATTTTTGCAGAAGCAGCAATCGACATTTTGTGGAGCATGTTATTATGTGAGTGG

TTTGGCAGTGGTTGCCACGAAGCCAATGAGCGAATCCATGCTGCATCAGAATTGCACAAG

ATGATGTTTTGGCTGTGATTGCATAGACCTTGGTTTGTCTGAGCATGACACGTGTAATAT

TATGTTGTGTTAATTCGGGGAAAAAAAAAAA

>532

GTGCGGTTTGTCTTTCTGTCTATGTACGACATTTTTAACACCCTAGGACTACCTATGATT

GCTGCGAACAAGTACATGTAGCATGTATCCCAAGCCAGGCACATGGTTGTGCACCCATCT

GATAGTATTGGTGCTCAAAGGAAAACTTTGTATGAGAGCGGTCGCGCATTGTGTCAAAAG

TGAAGTTTATTTTGTGAATTAAAAAAAAAAA

>533

TGGATCACCTCAGTAACCAACCCCTTCACGTATCCATTACGCTCGGCGTAGTCCTCAGAG

CGATGCTGAGCGGCGCCCTTGCGATGATGGGTGTGCGACTTGAACGCAGATCCCTTACCC

TTACGCTGGGCACGGATTACGCGTCCCATCTTTAAAAGGCTTAACACACCCGCCCGCCTG

TGTTGGACGAAGGAGACTTGAAAAAAAAAAA

>534

GACAGATCCCTAGTCACGGCAGAAAGCGCCCACATGCAACTTCCAACCCAGGACCTTGGA

TGTTAGTATCCACTGTTTTTAGCTATGTCAAATGGCAGAAGCACGCATCGAATGCTGCCG

AAACATGCTTCACAAGAGACTGTTATGATAAGGTTGAAGGGCGTAAATTAATTTCAAATT

ATATATTAACCTGGAACATGAAAAAAAAAAA

>535

TGTCGTGAATCACATCCTCCAGTTTTGCCACTTTGCGCTGATACTTGGCCGCCTGGTTTT

CGAGCGCCAGCTGCTTTTCCGACTGGAGGAGCTCGCCCAACGAACGCAGCTTTGCTTCAT

AAGTCTCCCCCTGACGCGAGAGCAGGAAGTCAGAGTTGATATGACCTCCGGTCCCCTGCT

TAGGATAAAAGGCATCCCCCAAAAAAAAAAA

>536

TCAACCCCCGCCGTCTGTGAGTATGCCAACTAACAAGATGCGATTGCTGCTCGGCGTGGC

AACAACATGTCAATTGTTGCCAACATGTTGGGTGTTGAGACATATTTTAGCAGCTGCATT

CCCATGCCCGACTATCGCCTGAGCACCATGAACACATGCAGTGACTGTGTTGTAAATTGT

ATCGATCGTTCCCCCCACCCAAAAAAAAAAA

>537

CATAGTGGATTGATCGTTTGTGCGATCGGAGAGTACACAGTGGCTTCAGTCCAATAGACA

ACCAGACATTTCAATTAAGATGTATATACATACATATGTATGTATGTATACCAGAACCCT

GGTATTTCAATATGGATTGTAGGATCCTGCAGGACTCGCAAACCATACCTCGTAATGCAA

GGTGTTGATTTATCTACATCAAAAAAAAAAA

>538

CGGTGACATATGGAATTTCCTCCAGACCTTCCTCTCCCAACACAAAAGAAGAAGGCGTAG

GGTTTTACATGTTTCATGAGTGAAGCAACTGAATTTATGCTGGATTGTTCGAGCCATATT

CTGAAATAGGATATTGGTACATAAGACATTAAGTCACATGCCTACGCGTGCAGTTTATGG

CAAAAATGTGCTTGATGTGCAAAAAAAAAAA

>539

GGTGGGGGGGTTTTGGCCCCGAAGAAGATTCTTTTGAACCCCTCAAAATTTTTCCCAATC

CCCCCGAAGCTTTGAAGGGGTTTGAAGATTCATTGCTTGGGAAGGAAAGACTTGATTGTC

CCAGAATCCAATCCATTGTTTTTTTGGGAAAATTTGCTGTTTAGGCCGTCATGAAAAGCA

CTGTTTGAATGGGGGCTTGGAAAAAAAAAAA

>540

AGGTGCAAGTGGAGTAGCACATGATGCCTCCCACCTTCAGCAGCGCCAGCCCCCGCAGCG

CGATTTGGAGCTGCAGGGAGTGCAGCTTGACTCCATAGTCGGGATGCCACTGCTTCCACA

CCTCGGGCATCTTGCGCAGCGTACCATCACCGCTACAAGGCACGTCGCAAATGATTTTGC

GAACAACCACTCTCTCCCCGAAAAAAAAAAA

>541

AGGCACACCTTTGCATTGCCCATCCCAGGCTTAACATCCTTGCAGAATTTTTTCTTGTCA

GCCGAGCAAACACGCATCAAAACGGCGTTCAGCGACAGATCTCCAGCGTCCTCGACCTCC

TTGCGGAACAGCTCCTCTTGACAGTCCCATGAGAGTTGCGCACGCTTCTTGCGGAGGCAC

TCCTGAACGCGACCATCGGGAAAAAAAAAAA

>542

TCCCGAACATCTTGCATTAGAATTAAAGTGGATCGTTTAATGCCCCACTTTTTTTCAACT

GAATCTGGTCGATATCAAAATGACCGCATGAGTGTACTAGCATGTACTTCAAGTGATGAG

ATCGTCTATCTCTACGTATGTAATACTAGCGGTTGTATAGTCGATATTGGATATATTATC

CCATAGGATCGATCAGGGGTAAAAAAAAAAA

>543

GTGCACCCACGCGGCGCAAAAGGAGTGTCCGCCCATTGGAATCATCACTGGATTCCTGTA

TACATTGTTGCAAATGCGACACTTGGTGTATTTTTCACCTAGCACGTTCCCCATAGATTG

TGTTGCGCGGAACAGATCGTCGTACTGTGAGGAAGGCGTTGCCCGTCGGTTACCCTGTTT

GTGAGGTGCAACGCAAGCCCAAAAAAAAAAA

>544

TTTTTGGGGGGCTTTTTTCTTAACCCAAAAGGAATGGGGGGTTTTTTGTTTTCCCAAGGG

GAAAAAGGTTTCGGGGGGGGGGGGGGCCCAAAAAAAAAAA

>545

GTCAAGTTCACTTGAAACGTCTTTTCAGGATGGAGTCCCCCCCAATGGGTGGACATGCCC

GAGTTCTTGAACGGTACGTGGGTGTATACAACACAAATATCATTGAGAACCATGGATAAA

CAAGCCAAGCATGTCAACACAATCAGCATTCCATCCAATTGTAGGAACATGGTTAAGGGG

CAATAGCAGTGCCCTTGCCTAAAAAAAAAAA

>546

AGGGGGTTTTTCCTTCCCCCCTAAATTTTTGGTTTTTTTGGAGAAGAAAAAAGGGGCCCC

GGGGGGGGGTGGGGGCCCCTCCCTTTCCCCGGAACCCGAAAGGGGGAAGGGGATTTTTTT

TGGCCCCGGGTTTTTGGGAAAAAAAAAAA

>547

GGATAGTGTAGGATGGGCCTCGGCCCTTCGCACTAATAACATGGACACGCCTAGCCCATA

GTTAGGTGGGAGCAGCGCCCGTGGCTGGGGGGTGGCAGAGCTCCCCAGAAGCAACCTCCC

ACAGTGCAGACGGTTCCAGTTTACTATATGATAACTTTGGTATGTACTAAGCAATGATAG

GTGCTGGTCATTGGAAGAGTAAAAAAAAAAA

>548

GCGCTTCTCCTTCGCTCGCTCGTGCGTTGCCAGCTTGGCTCCCATCTCCGTCAGGATTCC

CGACGGGTCCATCATGCCCTGGAACACGGTGTCGTAAAGGAAGTTGAACCTCTTCTCTCT

CTTCTGATGCATCGCCTCAACGCGCAGCTTGGACTCCTCCACCACCTCCCGCTGCTAAGC

CTCGTGCTGCTCGATGCATCAAAAAAAAAAA

>549

CCCAGTGCCCATCAACTCTTGATGATTGTCCTCAAGCGTCGTGCAAGGCTGGGACAAAGC

TTGCTTGCTAGTAACAACAATGGCGGTCCAAGCATACATGTGATATTGTCTGTACCGTAA

AGACTTTTGATGGTACTAGATGGTAATTGATGTTGAGGACTAACAGTTTTTACTCACTTA

GCACAAGGGCGTGAGTCAATAAAAAAAAAAA

>550

TTTTGCAAAGAAGATTTGAGTCCAACAAAGTGTGATCCGACCCAGCCCCGTGAGTTTGTT

GACCCCCCGTGATAGGAAAATAGTTCAGTTGGAGTTTGGCTTAGCAATTGACTGGATGTT

CGACAACTTTCAGGGTAGTTTGTATTTCCTCCCCCGTGCCCGATTTTAACCCGATTGAAA

TTTGCATGTTAGGTTGCCCGAAAAAAAAAAA

>551

GTGCCCAGCTTGGGCCTGCAAATCAGGTTGACAAGTATCGAATGCATTTTTCAGAGCTCG

GCAATTGTGTTCTTCACGTACTATGTTTGCGAGATGATGGGTTGTAGATTGGTTTGAGTA

GACAACTATTTGAGTAGACAACTGTTTGAGTAGACAACTAGATTGGATAGATTCATCTCA

ACAATACAGAGGGAATTCTGAAAAAAAAAAA

>552

CAAACTCTTCGTGCATCTCCTCTCGAATCAGGGCCCTCATCTTATTCTGCAAGTCATTCA

TTTCGAGTGTCTGAGGACTGGCGCTCTTGCCACTCTGCGAATTGCTTGGATTGTCTCCAG

CTCCATTGCATGCTTTCTGATTTTTTAACTTGTTCTTTTTTTTCTTTGCTTGCGATGCTT

TTGCTTGTTTGCTAGCAGTGAAAAAAAAAAA

>553

TACATCACGTTGTAGGGGACATCAATGTTGAAAAAATGTTTTGAGCGAGGGACATGTGCG

TCCTTGTGGTTGACATGTGTTACATGGACATGTAGGGAATGTTTTTGGGAGATTATGTTG

GAACAACAAACTCTTGAGTTGACATCACACTTTACGTGACAGGTAATGTCGCAAAATGTT

TTAGGGCGGGGGCTTGCTGTAAAAAAAAAAA

>554

CGAAGAGTGTTCTCGGTGTGTGAAGTGCCAACACGTTCATGCACCTGTGCGTCCTTCACG

TTATCTGGATACACATGCATTCATCTTGAAATGGAGGTTTTATATCAAGACTGTAGACAC

AAGTTTGAGGAAGGTTTGAGACTAAAACAATTAATTCGTGTCAACCAACACCTAGTGCAA

GTGACGCACTGAGCATTTTGAAAAAAAAAAA

>555

TATGTGCAAACGTGAGGATAGGAGTAAATCTATACGGTATCATTATAAGGAATTGTCCAG

GCTGCGTATGTGAAAACAGAGTCGGAAGTGTGTATCTCGTGTCGTGCTGATTGCTTGTTT

GCAAAAGAAATGGTTTCATAAGCCCCTCACTGTATAGAATTAATAGATGGATAGAAATTA

AGACTTTTTAGCTTTGCTGTAAAAAAAAAAA

>556

GTGGGAGCAACCAAAGTGATAGCCAGAGCTCATGAAGAGCATGGCGTGTAGCCTGAGGGT

GCCCTAGGTTTGCGGCCCTCCCCGGCCTTTGATATTCAACCACGCGCCCAGTGGCCAAGC

ATCACGTTGAAGCAATGCGTAGTATTTTAGGAACATTTGTATAAATGTAGAGTAGAATAG

GAACCATGGTGGTGTTGCCCAAAAAAAAAAA

>557

TTTCGCGACGAGAGGATACACACACTGGTATGGATTTTCGTTGTGCCACAACAAGTTACA

CGAAACGCGTGCGAATTACGCTACTTCTAGAAACTGCGCTTGATCGTGCATTTACTGAAG

TACGTTGCGTCCGTAAACAAACTCGTTCCCCACAGCCGGGCACCGCCCACGTAGGGTGTC

CAGTTGGGTGGTGCATGTGGAAAAAAAAAAA

>558

TGGCTGTGGAGGCAGACCAAAGCCGCAGCCGTGATCACAAAGGGGGCTGCCACCCAAAGT

GGCAAGTTCCTGGTGAAAACCATGGAGGACAACGCACATGAAAACATGGTTTTTAGCGGC

AAGTCACGTGCCAGGGACACAAGAGACCTTTGAGTTGTGCGCAGCATTGAACCCTGTGTT

TATTGCAGTCAAGCCAAGCTAAAAAAAAAAA

>559

AGCAGGTGTCCCTCACCTCTCTGTATCATACCCTCGCGCTGGCAGGTTTCAGATTGTGCT

GGCTGTGACCTGCACAATATTTTGATGCGATCTCTTTTAGTGTAGTGTGCTAGGTTTGAC

TTCAGTACAATCCAAGAGTTAACTGTAAAAGTTCAAACATATATCAGTTAATACATTCAT

AAGATCCTTGCTCCCTGTTTAAAAAAAAAAA

>560

TCAAGGCGCCAGGTTGGTGAGATTCTATGATACGTGTATGAGTGGGCTTCGGCTGTGTCA

AGAATTGTTTCACCGTACATTGTACGCGGACCGAGTACCTGAAGCGGTGGACCCTCTCTC

ACACGTAAAGCAACATGGCCCTTAACAGTATCCTGAATGCCATCAACACATCCAAAAATT

TTCCATGCAAATAGGGCACCAAAAAAAAAAA

>561

GCGGATAATCAAGAGAAGAAAGTGTATGAAAACGTGGATGGTGAGGAGGACCCTGAAGAA

GCACAACGCGAAGAGCCTGAACTTCCAGATCCAGTTTTAGGACCTATGGACCCGGAGGAG

CCAGCAAAACCCAGTATATGGTCGTGGTTTGGGTAGTACACAACTACGCTTAACTGTATT

TTGTACGTGTAGGATCGTAGAAAAAAAAAAA

>562

ACGAGAAAAATGACCAGGTTTGGCCCATTTTTCAAAAGATGTAACAACAGGATTTCGGTC

AACAACGATCTTTACTTTTTTTGCCTCTCGTTCCGGTGGACTAATGGTCATTGAGAATCT

CCTCTCTTCAGACGAGACACACGAAATACTTTCCTTTCGTTTATTGTTTTGCTGCTAGTA

TATACTTTATGTATTTTTTCAAAAAAAAAAA

>563

GCGGCTCGTCCAGAGCCAGGTGACCAGACGGGGGGTCGCTCAGAGACTAATGTGGCCTAA

AAAAAAAAA

>564

TTGGGTTAGGAATAGACCGTCCTTAATCCATTTTGGGAAAAATAATTTGGACACCCCCCC

CTATTTAAGGGGGGAGGGGACTTTTTTTCCCCCCTTTAAGGTTTTATTGTAACGGGCAGG

GGTTTTTTTGGATATGGTTTTTTTCAGGGGTTGGGGCCCAAAAAAAAAAA

>565

TCGCATGCCCTAGCATATCAGTCGTGCTCACTATGTTGATGAACGCATGTTAAGCCGGCT

ATGATCAATTGATTTTGACCATAATAAGGTGAATTTCGGGGATCCGAAACCCCCCCTTGA

AAAATGCCAATTGGAGCACCCGCTATATGGGCAAGGCCAACCTTTCAAAGAGGAGGTGCA

CAGCGTTGGGGCGGCCCTGCAAAAAAAAAAA

>566

AGATGTTTGATGGTTTCGACATTGAAACGCTGATCAATATTCCCACACAAGTGGATCCAG

TCGTGTCAGTAATCTTGCCTTGTATTGGAGTTTGTCCAAATCAAATAGGATGAAAACAGT

GTTACAGCTCGATTTTACATACGGCGCTGTAGAAGCTGCTTACATTTTGCTAAATTTGAA

TTCATGTCAACAACACTTTGAAAAAAAAAAA

>567

CGAGTTTTTGGGTATGACCCAATACCAGCCTCAGGTGGTTCTTGGGATGCATCCCACAAA

TTTGACAGCTGCCAAATCCAATAGTGTTCGTGCTGCGAAACAAGGTATCTTTGATAGTTT

GGAGATGGACATTAGGTGAGACTTTTTATACATGAAATGAGCATTTAATATGTATTCCCT

CATGCGTACACTGTTCAAGTAAAAAAAAAAA

>568

CTCCTGCAATCCGAGTCCCTTGATGTGCGTATGGGTTGCAATGCGCTGTTTTTTTGTTGT

GCTCTGTACCTCCTCGATACGCATATTGATTAACACGCAACTACACGCTCAAACTTGAAC

TGCGAAATTCAGCTGCAATACGGCGTAGCCAAGTAGGAAGCCAAACACGAAATCTGGATG

TAGACTGCAAATGTGCCCAGAAAAAAAAAAA

>569

GTCAAGGTGTGTCGTTGCCAGTGGTTCAAGAGCACGCATTGGTGCATGGGCACCCACCAC

AACCCCAACCTAGAAGTGCAGCAACACCAATCCAAGTGAATCCATGTAGGTAATATATGC

TCGGTGGATGAACTGTTTTTCAACATGCATTGAACCTTGATTTTGCCGCATGGAGTGGTA

TATTACAATTTTAGATGTTGAAAAAAAAAAA

>570

TGTCGTGCATCCTACACACATTTCGGGGAAAAGGAGGGGTCATTGAGCTTGAGCCTTACG

CGCACTTTGGAAGAGCTCGTAGCACCGCGCATCTCTGCAGTAGAACACATGTCCCTTAAG

GCGTAACCTACTGACTGGAGTTGTTGATGCTGTTCAATGGTTATGTCGTAATAGTTTTTG

TTTTAAGATTTTTTTCTTGTAAAAAAAAAAA

>571

AGAAGTGTTTTTTTCAGTATGCTGTATTTTCCCAGCCATGACCATGGGCCGAGCAGCCTT

TAGGAATCCCCCTCATAGTGTTGTTTCCCGGTTTTTGTTTTGCATCGTAAGAGATTCTGT

CTCAGGCTGTTCCCGTAAATGGCAACTGGGGCATCACTAAGCGGTCATGAACTACCAAAA

TACTAGCATATGCTGAACTGAAAAAAAAAAA

>572

GCAGGTGGTGATGGAGCCACGCCTGGGTTTGAGGGTGTGGGTATTGCCCTATCTCAAATC

TCAAACAGCAAAACCCGAATAACGATGCAGAGTACTAATACCAACAAATGCGTAGTGATT

TTTCTCGAGAAGAGAAGCTCTTCTGGTTATCAAATTGCAAATAGGCTTGGTTTTAGCGTT

AAGCAAATCACGTTTAAAGCAAAAAAAAAAA

>573

ATTGATAGCTCCACTACTTCTTTTCTATCTCGGCAGCGAAAATTGTGTGCGCCAAGTGTT

TTGCTTTCAGATAGACTGTTGATCAGCGCTTGAACACTTCTTGGTTGGATGGAAGAAACA

CCATTGGTCTGAGCACATAAGTCAGGCTGAGTAAACGGTCAATGTGATTGTGGATTCAGT

GAATTGCTTTCTCTGGCATCAAAAAAAAAAA

>574

ATTTCAGGAAGACACGTTCCTGCATGTACGAGGGAATTTTGACAGCGAACAGCTAGTAGT

TTTAGCAGCATGTTGTTAGCACTATGCTTGCCCTTTACTCTCTCTGTCCGCGTAATCAAA

GCTGCCCCAGGAGAGGGAAGGAAACAGGACCCTCACTAAATGGTAAATAAACAACATCAT

TGGACGAAGTATGTCTCCCTAAAAAAAAAAA

>575

TCGTAAAACATGACAACACATGTGGAGACTTATGGACACACAGAGAAATATTCAGGTGCG

TACTTGTAGCGAAAGAATTTCCTTTTGAAAATTTGTTGCACGCTCTGATTGCACCCGACT

ATCGAGCTCTTCAATCTGCGTTGCACATGCATTTGAATCCCCAAACCAAAAAGATCCAGA

CCACGTCTTGTCTCGTCTTGAAAAAAAAAAA

>576

GTCTGCATTGATCGTCTCAACTTTCTTTTTGTTGGCTTCAAATACCATCCGCCTGTGCTC

GATCTCCTCCTCTGTGTACCTTTTACCGAACCTCTCCTGGAAGCCTTCAAAGTCGACCTC

TTCCACGTTGAGGTTGGCAAGAAAGATTGGACTTGGCTACCAGATCTGGTCTGAACCTGG

AGATTCCTGTCCCCCAAGGCAAAAAAAAAAA

>577

TGGCCGACTCGCTGCCGTATCACTGCGGTGTTGTGGTACCCGAGAACAAGGTTAAAGAGA

GCAGGAACAGGAAATACGGTTGCGCTCGCATATTGCTAAAGAAATCAGCACAACTATAAC

TAAAATAACCACGCAAATTCCATTTCAAAAGCGCTTATGATGAGTATCGCCTGCTACAAA

ATGTCGACACGTTGCGCCCGAAAAAAAAAAA

>578

CCATTCTGAATATCGGATTTATTCCTTGTAAAAAAAAAAAAAAAAATCGTAGTTGGCTCA

TTCACATTTAGGGCGGTCGAGTCGCTTGTTGGCTGCCATGCGAGTCAAACATAGATGCTT

TGAAGTCTTGGCAACAGTTGGTTTCACACAGCAATTGGATATGATTTAATGCAGCGTCTT

ACGTGATGTTTATTTTCAGTAAAAAAAAAAA

>579

AGCTTTGGGAGTCGCCACAAGTATTTGTGCAGTTTATTTATACCAAGTTTTTGCCGCTGA

GGAAGCATAGTTCGGGACGTTCAGTTGTTATCGACCTTCGGGTTAGGACACACCATAGTA

TGCAGCATAATGCATATGTGCGATGGTAGATGGTAAACTGCAACTATGCGTTTTATGAAT

ATACATATAATGCACAAGTCAAAAAAAAAAA

>580

CGGCGGCGCTATGATTAGCTGGCCGACCAATAACAGTGCAACATCTGGGTAACAATTTCT

GGTTCATTGTCTCAATGTGAACTCGCTATTCACATTTCCGCAATAAAAAGTACCCTGAGT

ATCTAAATCTCCTAGACGCTAAATGGGTAATGACTCCTGCACATACCTTGCTAAAAAAAA

AAAAAAAATAGTTTTTTGCCAAAAAAAAAAA

>581

ACTCACACAGCATACACACATTGAAGGACTTTTGAACAACATACAACTTGATCTGCATTT

GCAGACATAGCTTGTCAGGCAGCTTGAAATTGAGGTGATCTTTACGGATGCGAGGAGTTG

TTGTGTAAAGAACGATTTGTTTTCCAATTTAATTATGTATCGGATATTCTTCAATTCAAT

GATTACAGCAGCAATAACTCAAAAAAAAAAA

>582

ACTCGACCAGTTTGAGTGGGAACAAACGTAGAGCAAGGTGACCTGCTAAATCTCCATGCT

CCATGAATATTGTCGAATAGCATCTTGAAATACTGGTTGTCTGGTGGCAGCTGTTTCTTG

CGAAATTACAGTATTGTGTATCGTTTTTGATGGCGATCGCATTTTCCTACTGCAATTAAT

GATTTCGGATATCACCCCGCAAAAAAAAAAA

>583

CGCTTGCGCACCAGAGCAGCATATAGTTCGGGTCACGACCAGGCCTCGCAGGCACATTTT

GCGGTTCTGTTATCATTGTTGTTAAGGCTTGTTGCTGACCCATATGTCAGGGAAAGTACA

TTAACGCGACAATTCCTACGAATATCGCGATACCCGCCGCTTCGACCATTCTGCCGCGTG

TGACAAGAAGCACCTAAATGAAAAAAAAAAA

>584

CTACAAAGATCGCCAGCACCTCTTTCAAAAGACCACGGTCCACATGCTCGCCATCTCGCT

CGCGTTGGATGAGCGCTAAAGCGGCGTCTTTGACCGCCCGTTTGACCTCCGTATACACCA

GGGCTTGAAAGCATCGAAGGCCGACTTCATGAAGAGGGGCGAGGGAGTGCCGAGAAAAAA

AGTAACGGTCTAGATAGTTGAAAAAAAAAAA

>585

CATGAAATGACACCGAACGTCGGTGGGAACATGTGCGGAAGGAGTAGAGCGGAGTAAGGC

GGTTGGGGAACATTGTCATACATTAGCTGGGTGTGTTGATAGCGTTTATCTGTATTTCTT

TTTTATATGTATTCATTGGCGTTTAGTCATTTCTTTTTCAATCCAAGACGCTGGGATATG

GATGCTTGGTGACAACTGTGAAAAAAAAAAA

>586

GGGAGGGGTAGGCTCTTTCAACAGTGACGTGCAATGCCACAGAATATCATAAGTTGGCCA

ACCAATAGCAATGCACATAATTACTTACATTGATCCGGTACACTATCTGGATGTGAATGA

TCGATTCACATATCCAGAATGCTGAATACCCTGAGCACCCTAAACTCCTAGAATATATAG

AGTAACTGCCTGGGTACTCTAAAAAAAAAAA

>587

GTGCTAGCAACAACTTCTTCAGGAAACTGCCAATGTGCAGGTATCAACTCCTTCAGGGGA

CACGTGCCCGGTATTGCCCCATGCTATGAAGTGCATGAAAGAAGGCTCTATAAAGGATGA

CTTTTCCAGCTAGTTACACCAAGCATGTACCGAGCACAGCTGCTCTGCAGTATGAGATTA

TGAATTCAATCATTCATCACAAAAAAAAAAA

>588

ATGGGGTGTCATGATTCAGAATATCAAAAGAGCAGCGCTGTTGATGAAGAAGCATGCGTT

GGATTTAGCGCGGCACGCAGCAAGTGAAAATTGATTTGAATTTAGTGTCATAGTAGCCTA

TGCTGTGCGGATATCGCTTTAGGCTGGGTATTTCGCACATGTGCTGTTGGTATAACTTAA

ATGTGCTGTAAAGTCTTGATAAAAAAAAAAA

>589

AAATGCAAGACGCAGTTACCAGCAGGCCTTGAAGCATTTTGGTGTGACCTTGTGCTAGCT

AGCAATGCTCATTGGCACCGGAATGTGCAGAACCAGTCTTGTATTTGTTGCTTGCTTTGA

ACGCTTGCATACCAAGCTGATGGAGAAAGAGCTCCAACATTCAGCACCCCCTTGTAGGCT

ATTATTAGACACAATCCTGCAAAAAAAAAAA

>590

CCAATGCCCGTCGGCGGATTTGGAGCTGAGAGCTGGAGCGTAGAAGTTTCTGACCGCAAC

AAACGCTTCACAGTAGGTCCACTTGTAAAATCATGATGGGATGAGTAATTATTGGGTATT

TGTTTGCATGATACGAAAAAAAAAACATTCACAGACATAAAACAGACATGAATATTAGAG

TAATTTATCATGGCCTGAGCAAAAAAAAAAA

>591

GTGATTTGGCCATCGGCACCAGCAGCGAACGAACGAGCAAAGTTTCGCGTAAGAGCACTG

ACGTCGGACTGAGGAGCAACCTTAGTGTTTGACCAAGCTGTAGGCGTGCGCGTCGAAAGA

CGCGCCGCGCTACGCAGAGCAAAGGTTGCTGAGCGATGCATATCAGAGAGAGAAGGGTGG

TTTGCGCAGGACGCTCACGCAAAAAAAAAAA

>592

TTCTAGTAAATTATCGTGATTTTAGCTTAGTTGCTGTCGTTATGGCCTATCCACGTCCGC

AAAGAGTGAATGTGTTGAATTGGTGGTGCTCTTCAAATATCTGGATTGAAACAGTTCGTT

GTCACTGGTCAATCGCTGAATCTAAGTAAGACATCAAGTTGGTATCTTAACTGTCTGATG

TGGTTGATGCTGCATCGAATAAAAAAAAAAA

>593

CCGAATGGCAGCGCGAGGTAGAACTGTATGGGCGGTCGTATGACAGCAGCCCGTTATTTG

AACAACAGAAGTAGTAACAGTATTGGGAAGATGATTAAGGTTCGGGTTATGCTGCACAAA

TTGTAGTAATGCAGCTCTAGAGTATAGGCAATGTAATAAAATTTGTGTAACATTTATGAA

AGTTGAGTGGATTGTCCTTTAAAAAAAAAAA

>594

CCGGAACAGTCCGTGAAGGTCGTTCGCGTCTCTTTTTCCGACTCGTCAGCCCACGCAAAG

TCATGGCACACGCAAATAGCAAATATACCGAGTTGTAGGATCGAGCCGATTCGCCCAAGG

TTGAGCATTCTCACCGATGTTGACAAACACGAACGTAGTATCTATGCCTCCTTGTACGCC

AACAGTCTGTTGCTCCCACCAAAAAAAAAAA

>595

GCGAGATGAAATTTGGTTATAGGAGGAGGAGTACTTTCTCAAAACAGTTATGTGAGGGCA

TGCCTGAATTTGACAGCCAAGACCTTTAGTTGTGACGGTGTGTGTGCAAATTGTTTCCTC

CATCTAGTCATTAAAATTAGGAACAGTATTGTGTCCACCGATATTGATGAATAGAGCAAA

GGTGAGTGCAGTTTACGCAGAAAAAAAAAAA

>596

TGAATGATCTGTCGGCATCTTGTATAGAACACCTACAGGATCATGCATAGTTTATTTTAT

GTTGTGCCGGCAGGCTGATCAGCAGTCCATACCATTTGAAGCACTATACGCAGCGGCATG

ATTGCTTGCAAACAACAGTCAACACTATGGTTTCATGTTCGCAACAGATAAGTGATTGTT

CAGTATAATTCGAGTGCCCCAAAAAAAAAAA

>597

TTTAAAAGTTGTGTTTGCGGTTTGGGTTCGCAAACGGATTTGTATTGAACCTTGAAGTCG

ATGGTGCAATTACTGCATGCACCTGGCAAACTTTTCTTTTGTAGGTGAAACCGATCATAG

AGGACAGGGTTAGCCAGGCTGCCTTAGTGTATTGACATTTGAACTTGAGCATGTAATTGC

AAGGTTCTATATGTATAGGGAAAAAAAAAAA

>598

ACCCGCCTTCCCCAACAACGCGCGCGGGGGAGAAGTTGTCCGTGGCCTCCACCACCTCCG

CCCGAGTGTAGGCTTGTAGGTGCGCGAACTGCGACGCGCGGCTGTGGGCAGGCTCGGACG

TGGGTACACCTGGACTTGGGGCATCATGCTCCGGCTGCGTATCCACATCAGCGCCAGCTG

GCGGCTCCGACGGTACATCCAAAAAAAAAAA

>599

CTTGGTTTGTCAATTGATGAAGATGTTGCACTTGATGAGGAAGATGCAGATGACCTTCCC

CCCTTGGAGGAGGATGTCGATGAGGGCTCTCGCATGGAGGAGGTTGACTAAATGTAGGTC

TATACGGTGAGTGCATAGTCAGGGTTTTGGATATCATGTAAAATTTCCATGTTTAGTGGA

TTTTAGTGGCATGTGATATCAAAAAAAAAAA

>600

CTCTCTCAACCTTCCAGCAGATGTGGATCGCCAAGTCCGAGTACGACGAAAGTGGACCTT

CAATTGTGCACAGGAAGTGCTTCTAATCTCTGAGCATTGCGTAGTTGTAGTTGCAGTCGC

AGAGTTGTTAGCATAGCACATCCACCCACTCGTTAAGCGAATAGCCAGTAAATGCCTTTA

GAAACTTAGCCCCTCCCGACAAAAAAAAAAA

>601

GGGTAAAACCAGGCCACCTCCTCGTTTGGTTTTGCAAGGGGTAATTGCATAATTCGCTCC

CGTTTTGAATTGGAACTTTTTCAAGATGTTGTTTTGTGAAAGATCCCCCCCCCCTTTTGA

AGGGAACGGGGGGTCAATTTTGGGGGGGCCCGTTGAAGTCGCTTAGGGTATTTTTTTTAT

GATTGCCCTATTTGGTTTCCAAAAAAAAAAA

>602

TCCATGTATGCCAGCTGCGGGTTGTTGAGGAGTTGGGAGTGGTTGGCAATTCGTTTTGGC

AGATGGACCACGGTGTATTTGGACGCTTTGAGCCCTTGGGACCCGTGCCCATGGAAGACC

CCAAGGAACACCAGATTGGGATCACCCGCAAAATTGGGAATTATGGTGCCTTCATCCGGG

CACTCTTTTTGGGGCTTCCCAAAAAAAAAAA

>603

CCGCTGCTGCTTGTATCGGAACCCCCCCCGCTGCTGCTGCTATCGCTCTCGCTGCTGCTG

CTGTCGCTCCTGGCGCGCCTCCTGCGCTTGTGCTTGTGCTTGTGCTTGCGCTTTTTCGCC

CTGCGTGCTCCCGAAGCGAGTTTCCAACCGCGAGGTTTTAGCCGCGCGCGCTCCGTCGAC

ATAGGCAAGAGAGAGAGGCTAAAAAAAAAAA

>604

GAGAATACATGCACGCGCCCCAGTAGTGTCAAACTACATACGCATACATAAAAAGTTATC

AACCTGTTCGCGGCTCAGCCTGTAGCGCTTGCACCTGGCTGGTGCTGATTTGATCTTGTA

GCAAAAACCCATTTGTCAAGTGGACTTATTTTACAGAAGCCCTTTAACATTTGGTACACT

TTTGTGCGCTTCTGTGTCCCAAAAAAAAAAA

>605

ATTGGTTAGAGTATCTCATATTTGTGTTTGTAACATGGTGCCACAATTACAAACCTGAAA

ATGAAGTAGGCATGAAAATGAGGAACACTTGCCGTCTTACGCACATGTAAACGCCAACTT

AGTGCAAACATCAAAGCAACTGTTGTGCGGGTAAACCCTGTACTGCGAATAATAAGTACA

CCTCAGACTTCATTTTCTACAAAAAAAAAAA

>606

ATTTATGGGTTGTGATGTTGGTGTTGCATGCACATAGTGTGTGTTTCGTTTTCTAGTCTC

TGCATTTGTAACAAATGGTTTGTGCCTTTTCAGCAAGTACACGGCCCTTGGTAAGGTGAC

GTTACTTATGTAAGCTGGAAAACTGATACAAGACGTTGTTCTAGTTGATGTACACTCTAG

CAATGATGTCGATTGAATTCAAAAAAAAAAA

>607

CTGGGACAACATTGAGCATAAACGTCGAGTTTTGGGCTTTTTCCAGTACAGCATTCATCA

TTCCCATCAAGAACTCCCTCGCAGCAGATGCCCTCAGAAAAAAAAAAAAAAAATGCCACA

AAATGCAGCATGAACAGCGTCTTGACAATGGCACATGCTGCTCTCACAAGTGCCAAGTAC

AGAGCACCATTCACCCTCACAAAAAAAAAAA

>608

TCCACAGACCACTAGCACACACTAAATGCTTCTTCACAAGTGTACATACATGGCTTAGAC

CACTCGCCAAAAGATGCCTGCATCTGCAAGGAAAACTGTGGAGGAAGGCTGTTTGCTGCA

TCTCTATTTGAGGAAACATGCAACAATTCGCGATAGCCGCGATAGTCGGCCGCCAAGAAA

TAATTATTTCTGAGCTTCTCAAAAAAAAAAA

>609

TGCCATGTGCCGACAACAGTTTATGAGTCGTGCTCTTGTTCTGGCGATCGCAGCAAAAAG

GATTACTCAGCCGAACAACAGGGTCAGATAGGTCAAGGTGGTTCGCTGGCATGGACTCTT

TGGTCGTGACTTCCAATTATATTCGGTAAAAACAGACTTCGTTGACAGATGTGTGTACTG

GAGTATAATCCAGGTAGCCGAAAAAAAAAAA

>610

CAGGAGCGCTGCTAATTGTGCAAAGCAAAGTAGGACCATCAGCAGCGCGAGAGCTGTGTA

GGTTTAAGAATGTAAGCGGAGGCAGTTCCCATACACATCGCCCCCTTACCTCTCACTGAA

AACACACGGGCTTGTGTGTGGGATGTGATAAAAGTGCCGCATACAATAGTATTTATATGA

GATGTGTACAAATATTTTACAAAAAAAAAAA

>611

ACTTTACTCCGTAGGGACAAACAAGCGAGCACATTTGAGAAGACATTATGAATAGACGTC

TGGGTTACGACTTGAATAAGAGTGATGCCTTCGTGTACACATTCAGGGTTTGATGTGTCG

CATTGATTAACATTCACAGTTGCCACCAGAAAATTTGTTAAACTGGTCGATAAAAAAGAA

AAGTGCATTGTAGCTGTTGCAAAAAAAAAAA

>612

GACCCTTTTCACGGCTTATTCCTACTCTCATTGGGCAACGCGCAAAAACTAGAATGTTTG

CAAAATGCTGCTCTGTTTGCCTGAGATGGCCGTGGATATTATGCAGAAGATATGTTGCAT

TGTGAAACAGAGCCTAATGACATGCAACACCTACGCTTGTATTTATCACTGCTATTAATG

ATCGCGTATATACTCAAGGTAAAAAAAAAAA

>613

ATCACGAGGTCGTGCAGTTTTTTTTGGATGCTGGTGGGGATCCTAAATAAGGATCGTTTT

GGACAATTTGCTTTTGAGGGGGGTGCCCTAGCACTCAAGCAGGTGAAGAGCCAGTAGAAT

CCCTTGGTATATTAAGTATTGGCAGTTCAAAAGGTAAACTTCCTTTTCAAATATTATTGA

TATTTATTGATGGGGTTCGGAAAAAAAAAAA

>614

TCGAGCCTCTTTTGTAGTGAATTGCAGAAGAACGGGAGCTGTACACAGAATTCCACTAGT

GCTATTGTTGATATGCTATCATGACGACATGTGTCCTAAAGTAGCTGTTCCTGAGAGTTC

AGCACAGGCGCGTGTTCGAAGCTTCTGGTGTATCCTAAAGTACTCGTTGTAATTGCTTAC

AAGTACCACGTCCAGCTTTCAAAAAAAAAAA

>615

AATGCCCGTCGGCGGATTTGGAGCTGAGAGCTGGAGCGTAGAAGTTTCTGACCGCAACAA

ACGCTTCACAGTAGGTCCNCTTGTAAAATCATGATGGGATGAGTAATTATTGGGTATTNN

NNNNNATGANACNANATTTGTTAAAAAAAAAAAAAAATGGGTAGGAGAGTAATTTATCAT

GTCCTGAGCTACGGTGAGTCAAAAAAAAAAA

>616

GTTCATAGTAGCTAAAGCTGCTACCGCGCGTCGATCGACTATTCAGGGTCTTGTTGCGTC

TGCGGAAATCTTTGCAGCACCAACTATCCCGGGTGAGCCTGGATGACTACAACTTTCTAG

TCACGATGATGGGCCTGCGCCGATTTTTTTTCACCTCGCGCAACGAGGAAACATGCGAAA

GAGGAGCGCGCAACCACCTGAAAAAAAAAAA

>617

TCTGGTGGTTCAAACGTTTATGCTTGTCCCTTGGAGAGTTAGTGCAAATTAGAGTGACAT

TTCTCTCTTGCAAAAAAAAGTTATCTGCATCTCCCGGTGTAGTGCGTTGTTCGGGAGTTC

AGGTATTTAAACAAAAAAAACCTTTTTGTAACCACCAGCGAAATTGGTGATTGTCTAGGC

GATTAGTCCACTCGCTAATCAAAAAAAAAAA

>618

CGCCCTCACTGTCAGAGATCTATGATTTTGGACGTGACCAATTTATGGATAAAGTGTATA

TCTAGGGATAACAGAATTTTGTACTAGCACAATCGGTATCGGGTCAAAAAAAAAATGCTA

CCGAGCGAAAGTTGTAGATGTAAAAGTGTATCCTCCATCAATGACGTTCACTGAAATGTA

AACGACTACGTCCCCAAACCAAAAAAAAAAA

>619

TTTCTAGGGAGGTGGACTTAAAACGATTGTTGGATTTCCACAAGCTGGCTACTAGCGAGC

CGTTCGGGTGAAAAACATGTGACTCTGGACCCCCCCATGAAGATCCATCCAAGCATAATA

GGCAGTGTTGAAGTGAATGGCTTTATTTGCGCCAATAAGCAATCTTGCCATCAATGTAAG

CATCTCTAGATGGTTGTGTCAAAAAAAAAAA

>620

ACAATCCATAGTCTATTTGAGAAAACCTACCGTTTAGGCAGTCGTGACAACTGATGATGA

TTCGCTATGTCAGTTTGTCTAGACAGTCCTGATTAAAACCGCTCTGTCAGTCTGTTTAGG

CAGTCATGAAAAGCGTTGCAACAATGTGCCATTGAAATACATGCAGAATACTGATAAAGA

TCCGCTCTGTCAGCTTACTGAAAAAAAAAAA

>621

GGCATGCAGGCAAAAATGCATGTGTCAGTTGGATGCGGACACATCGACACATAAGGTTTT

CCCGATGTGTCGCGTGCAGGCCACAACGGGCGTCCACATATAGTAGGATCCACTGCAAGC

TAACATGTGAGCGCCTTTTTGAGGCCTTAAGACTGACTGATTTTTGTGTATCCTGGCTGC

CCATTATGATTCAGTTCATTAAAAAAAAAAA

>622

TCCTTTGCCATCTGCTCCCGTCGATACCAGTGTTGGGGGCAGCGTTTGCATCTCCTTCAT

GCGTACATGTTTGGTTGTTTTCCTGATGAGCATCTGGGCCGCGCTCGATATATGATATCA

TATGTGCATACCGCTATGCTAGCGTGGAACTCTTGCATTTTCACGTGCAATTAATGTATT

GAGTGCTAAGTCTCAACCTCAAAAAAAAAAA

>623

CTGTTAAATCACAACACTGACAACCCCGCTTTGGCGGGTGGACTACATAGTAGCTGCACG

GGAGGGCTAGGCTCTTTCAACAGTGACGTGCAGTGCCACAGGCAAGCGCTATCATAAGCT

GGCCATCCAATGACAATGCACGTAATTACTTACATTCATCCGGTACAATATCTAGATGTG

AAGGATCGATTCACATCTCCAAAAAAAAAAA

>624

GTAGCATTGCTTTTTGTATGCCTGGTCAAGGAGTCAGGACGTTGGAACTCGCCTAAGTTC

TCACACTCTGTTTTCGTTTAGTTTTTGCCTCGGTCGATGATTTGTCAAAGGCCATGTTGG

TTTGGGGGCCTTCATCGATGGGGGAGGGATAATGCCAAGGGTTTCATAAACCCCCATAAT

ATTGGGGGATTCATTTTTTCAAAAAAAAAAA

>625

ATTGCTGTTGATGAATCGACTTGAACAATCTGCTTTAAAAAAAAAAAAAAAAAAAAAAAT

GCTACCCACACGTTACCCATGTTATTTTAGTGCACAGCACATGATAGCCTGCTTGCCAAT

TTGATGCGCCAGATAGAGGAAAATAATTGTCAGCTCGAGACAGATGATAATGATAATGAG

AAGACATATGCACCAGGTGTAAAAAAAAAAA

>626

ATCTCGTAAGTGTGGTGTGGTGAATAGTCCATGCTGTGATAATTTTCTTACTGATGTACA

AAAGGAGATGAAACAGTTTATATCCTCGCACAAAAAAACATCTTGCTCACATGTCACCTC

TGTGCATGCTGTTTTCGCATGGGCACGCCTTGTAGTTTTATTGGTAAGCCTCAGTTGTAT

CATAGTTGGTAAGCGCCTCTAAAAAAAAAAA

>627

ACAAGTGGCCACTCAAGGCTTGATGACTGCCTGAGTCCTCAGGGCGTTTGCCGCGGCGCT

ATTGATCAGGATAGCTCCTTTGCCTGAGCGAATTTAGCGAATTTAGCAAGGTTGATTGCA

AAATTTGGAGATTTGATTCCTTGGAACAGGCATGAAGACTTGTTGTATATATAATTTTGA

TTCGTCCTGATGGTGGGCCCAAAAAAAAAAA

>628

TTTAGGCAGTCATGAAAAGCATTGCTATAATGCGGCATTGAAATACATGCAGAATACTGA

TGAGAATTCGCTCTGTCAGTTTAAATTATCCTGGTGGGAATTCGCTCTGTCAGTTTACAT

TATCCTGGAGTCCAAAAAAAAAAAAAAAAGCAAAATCGAAACAAAAACAAACACAGGCCT

GCTGTTTATGCAGCCATGAGAAAAAAAAAAA

>629

CAGCCAAGGGCAGGAGGCCCTAGGAAACGCATGAGGTCACCTTCACGAACTGAACGACAC

AGGGTTGGGTAGTGTTTATTGATTTTACCTGTGACCGTGATTGACGGAACCTTGATGCCA

AGAACTGCACATGTAGGGCCTCGTGATACGACATCTTTATTATTAGTTATGAATCTTTGG

AAGTGATATTTGTGTTGTGCAAAAAAAAAAA

>630

TATGCTTCACTGCAAATACGTAAGTCGAGTTTGAGTCATAGGTGCAAAAGCGTCAATGGC

CAGTGTTAGTGCATCAAGACACGATGATACCTATGTATGACCTGCCAGAGGGTGGCTAGT

TGTGGTCCCAGGGCTTGTGATTTTAAGAACAGATTATCTGAAAAACTGAATAAAAAAATT

CGTAGCTGAAGCATTCACAGAAAAAAAAAAA

>631

TTCTTAGAATGGGAAGTATCAATAGATCATATTTACTGAAGAAATTCATCCTTGTTGTCT

TGCACTCAGGTTCTTTTGAGTGCTGAAATGACAGAGAGGCTTGTGATTAGTGTAAACCGT

TACTTGTTAGTCGAAAATATAGTGTCTGAACAAAAAAAAAAAAAAAAAAAATCTACTGCA

TGTAGTCGCAGCATTCACAGAAAAAAAAAAA

>632

CCCTTTCAAACCAATTGCCCGCTCTGCGGTTGGCTCACATTCATTAACAGTATAGAAGGA

TAGAAAATAAAAAAAAATTTTTCTGATGTGATGAAAAAAAAAAAAAAAAATCGTAGTCGC

AGTATTCACATCATGGACAGACGTCTATGCGGACCTTTTCTCAAGTGTGAGCTTACCAAT

CTACTTTAGTATTTGAAACCAAAAAAAAAAA

>633

GTGTGCAGTGCGTGGATAGAGCCTGGGTGTATTCTTGTAGACCTTAGGATGTCCGAGACC

ATAGCATGTCATTAGGTACTGTATGCAGGCAACGACCTTATAAACACTTTTAGTTGAGGT

TTGATTAGCTTGAGATGATATTGATACGGTGGTGTTCCCCGTATGCATTGCATTTCAATG

AAAGACATGCCTATCGAGTTAAAAAAAAAAA

>634

AGCGTAAGCATTCCCAATATAGCACTCGAGTGTGTGTGCGTATCGCAAATGGATCGGTGC

ATGTCATGCGAACATCTCCTCCCCGTAACAATGACATTGTGCGTATATAAGTGCAGGTCA

ATATTCTTTGAAAAAAAAAAAAAAAAATCGTAGTAATCGCATTCACAGTGTATCATCACG

CCTTCGTTGGCCTCTTCACGAAAAAAAAAAA

>635

CGATGCAAGTAGTATGCATTTGTGAATTATGCACTGTGCATTTGACATAGCGTTGGCCTG

GTGTGTTGGGCATATCTGGCTCCTCATCTTTAGCCGCAGCAAAATTACACACATATTCTG

TTCAGTTCATGCCTCTAAAATGACATCACGAGGAATCTGCGCTGTAAACAAGTGCAATAA

AACAGATTTAGGCGAGTGTCAAAAAAAAAAA

>636

ACGGTCTTGGAACGGGGCTACCGCCGCAGCTTACAGGTGGAGCATGTAGGGTCACCAAAG

ATGCACAAATTTAGATTTGCAGAGACCTTGTAATGTTTCTATAGCATTGTACATAGACAA

ATAAAAAAAAAAAAAAAAAAAAAAAAATTGAGTATAAACTCATAATTCCATGGAGCTTCC

TAAGGCTTTGAAGTTCTCTCAAAAAAAAAAA

>637

GTGTTGGCGATTCTGAACGCAAAGAATTAAGTGCTCATGTGCATAAGAATGACTGCATGT

TTTCTCTAGCGTGTACATTGCGCAGCAGATGTGAGCATCCGGACAGAACTTAAATTCGAA

GAGTGGACGATGCCAAAAGCAATATAACTCCTGTCAAATTATTTCACTTACAGGCTGAGC

TGCACTCTGTGCATTTGTGCAAAAAAAAAAA

>638

AGATTGTTGTAGCTAGATATTGACCTATGACACTAGTAAGTGAGAAGTGCTGTGAGTTTT

GGATACTGAATCACCTCAAGGGATACACAAAGTATTCTAGGGACTAATGGTCTGAAGTTT

GGAACCCATGTACAAATGCGGGCATGTGCAAACCCTGTCCATCATTAAAAAAAAAAAAAT

CGTAGTCGCAGCATTCACAGAAAAAAAAAAA

>639

TGATCCACTGCAGTCTTCGCCAGCGACCGCCCAGACTGCCAGCATGGGAGTGTTTTTGAA

ATGATATCACCACCCGTTGGAGTGACAGCTAGTTAGCATGCCTTTGCTGCTCTGGGTATC

TTGGAAAAGTTCCGTGTGACGCTTCTTAATACGACTTCTTCAAATTGTTTAACTGCTAAG

ATTGAAAGGTTATTTTGTATAAAAAAAAAAA

>640

CCCTGGTCGTTCGCGATGATCTCCACCCGCCCATTTTTGTACACGCCGACGCAAGAGTAT

GTGGTACCGAGATCGATACCGATCACTGTACCCATAGTCTCCTCCTCCGCAAAGGAAGAA

GCGGCTAGTAGTGCGACCGCTGCGCAAAGGAAAACGTTCATGGCTACACCCATGTTGGAA

GGCGTCCGCGTGCGCCTCACAAAAAAAAAAA

>641

AGCCGAGGCTCTCAATTCCGAACCAATAGTACCAGGATGGCTTCGGAAACCATACATATA

TGAGCGCATTTACGAGTTTTCTCACATCTTGCCCTTGTTGGATTGTGGATGCCGAACACT

TGACGTTGACTTGCGCATGATGTCAATGCAAGGCACCATCATGTAGGAGAGATTTACGGT

TCTTTGTGGTGTAAGTTGTCAAAAAAAAAAA

>642

CCAAGCACCTGATACTTCAACCAAGTTGGATCTGTGGTTGTAGACGAAATGCTGAAGATG

TGTGTGCCTAGGAGAGGACATTGCTTTGGAGATCAAAGAATATTTGTGGGTGCCTAGCAA

TCTTTACTTTTGGTTGGATTGTGTTTGCAAATGCAAATATTCTTTGTGTGGAAATAAAAT

GATGAATTAGCAACTGTGACAAAAAAAAAAA

>643

TTGTGGTGTTTCTGGGCCGCACCTTTGTGGATTCACATGGTCTGGTGATGTTTGGTGTGT

TATTTAGATATGCAGGACTTCGAATTGGGATCTTGAACTGCCTTAGCCCTTCCCGCAGTG

AAGGTGACATCGCCGGGTAACACATTCACTATAAATAATCTGATGAGGTCAAAATCTGGG

TGTTGTATCCCTTGAGGCTTAAAAAAAAAAA

>644

TGATTCGCTGTTGTCCACGCAATCCAATATTGTTGAAATGTGTTTAGGTAGCATGATAGT

CCACTGCGGAGATAGGTTGATCCTGCAAGTTTCTATGCGCATGCGCCTATTGATTGCATA

GAAGACATCGTTTTGGTCACTTATGTGAGGGCAACCACGCCAACTGGATCAATATTTCAA

CAACCTAGCTGACAGCGGTGAAAAAAAAAAA

>645

ATGACAGTTGACATCACAAAATGCTGAAACATTTCGTCATGCATGTATTCTGTATATACA

TAGGGAAATGACACTTACCAAAAAAAAGGGGAGCCATGTCAACTGAAGCGTGCGTCAAGT

TCTATCAGTGCTCAGGGTAATACTCAAAACCAACGCATCAAGTAAAAACCTACGGCCCAA

GGAGAACATACCTCCCCATCAAAAAAAAAAA

>646

AAAAAAAAAAAAAAAAAACCAAGATGGCGCAGCATTCACAGTTTATGGGTCTTGGCGATA

AAGGGATGGGGTTTAGGATATTCGTGTGAGGATTTTAGGTGATGTTCTAGAGTTCAAGGG

TTTTAAAAGCTGTGGTTGAGCGTGAAGGCTTGTGGTCGTTGGGTTTACATCAATACCGTG

CTAACGGAGTTGTCGTCCGCAAAAAAAAAAA

>647

TCCCCTTGGATCATACGACATAATTTGGACCTAATGCACTGGTTTGGAAGGTTGCCCTAC

ACAGCGATGGAAGCTAGCAAGCCACTCAGCTGACATAGATAGAACTCAGTTAGCTTGCTG

CCCTTTGGGTTCCGGTCACCCCTATGTACACACGTACACGTAAAGTGAACACCAGTGAAA

ATATTACACCTAAAGGAAGTAAAAAAAAAAA

>648

GTATGTAGAATTCCATATAACCGCCTGGAGTGTTTCACAGAAAATGTCCCAATATGCAGT

ACACACATGTATGATGTTTGCCGTACATACGCACAGATGGAACACTAGAAGTAGATTACA

ATTCAACTTCATGTGCAGCACATGAAATGATTCTTCTGGATTAATCTTGTTGTAATTATT

TATTATCCTATCTCAGGTACAAAAAAAAAAA

>649

TTTAGGCAGTCATGAAAAGCATTGCAACAATGCGGCATTGAAATATATGCAGAATACTGA

TGAGAATTCGCTCTGTCAGTTTTAAATCACCAAGCATTGCTACAATGCGGCATTGAAATA

CATGCAGAATACTGATGAGAATTCGCTCTGTCAGTTTAAATTTTCCAAAAAAAAAAAGTC

GATCTGTCAGTTTATCCTGGAAAAAAAAAAA

>650

AACAATATGAGATGATTTAAGTTGGCAGTATTGCAACGCCATGCGCGCATCACTATTCCA

ACAAACATTACTTTCATATGCAACAACCAGAAAACTCTCAAAAAAAAAAAAAAAAATCTT

ATGTGCTAAAGTGTGCGCTTAAGTTACAAACATGCCTTAGTAATTATCTTGTATATATTC

GTTTTATCGTAACAAAATGCAAAAAAAAAAA

>651

ACCACGTAGCAGCATGATTCTGTGCAGACTAGCACAGCTTACAGCAGTGATTATTCGAGG

ATTAGATGGTGAGTACCAAAGTGTTTGTAGCATTATTGAGCAGTACTGGAGGTATTCATG

TGTTTGTACAATCATAGTGAAGACGATTGTACATAAACTTGTACAACTCTCCCAAATCTT

TTAAAACTGTCCATTACATGAAAAAAAAAAA

>652

GAAAGGTGCCATCTAAGTGCATCAGGACTTCAGTGTATAGAGTGTGCAAAATGGCTTCAC

TATGACAGCGTGAATTGCGTGAAGTGTGGACGCACATGGGTCAACATCATATAATGTCCA

GACAAAATCGGGTGCTTGTGACCAAGGAACATCATAATCGCTCGCGCCAAATATTTGAAT

ATTGACGTGAACGAGGCATCAAAAAAAAAAA

>653

CGTGTCTCATATTGTCAAGCTGGTGATCAGGACACGGCATCAAATGAAAGACATGAAACA

TCAGATATTCTCATGTCCGAGTGTTTTGACCGGGTTGTGCTCGGCTAGACTTGTCTTCTC

GACCGGGCAAGGCACGTCCCTTTTACTGTGCGCCCATGAAATCTCACCATCTTGATTACT

AAGTGATCGCTCCCTGGTTCAAAAAAAAAAA

>654

ACGGTGAAATTGTACCCGAGCCTTGTACACACCGCCCGTCACATTTTGGGAGCCCTTTTT

ACTCCAGGCCTAGATAATTACACTTAAAGTATAAAAAACGGGTGTATCATTTTTTAGATC

AAAAAAAAAGCGCGANTGAAATGAAGTCGTAACAAGGTAACCGTAGGGGAACCTGTGGTT

GGAATAAAGCCTTTCTATACAAAAAAAAAAA

>655

CTGGCAACCTGCTGCGGATCACTCCTGCAAAGATGGTTGGCAAGAACGTATGACCAGTCA

ACTACATCAGTTTGTCTAGGTTTTAATGCACAGGTATTGAACAGGTCTTGCTGATGTAAA

TTACCCTATTTTGTGCGCATACTTCCTAAACCTAAAAAAAAAAAAAAAGTTCTGTTGTTA

CAAGTTGAATAATGATTCGGAAAAAAAAAAA

>656

AGGGCTTTGGTGGATTCCGTTTACTAGTATGTTCATATTAAGTGTCCATGTCCAAGCTTT

TAGTATGTCCAAGCCCTCCCTTGCTGCGTTTTCGGATGGCTAGCACTCAGAGCTGAAGGG

GCTCGGCCCATTTGAAGATACAAATGACATTGGACATATTTGTTCGATTTAATATGCGTA

TGGGATTTTGCGCCATCCCCAAAAAAAAAAA

>657

GGCGAAAAATGGGGGGGGAAACAATTTCCCCCCCAAACCCATTTCCCCCCCGTTTCCCCC

CCCCTTTTTTGGGCCCCCCAAAAAAAACCCCAGATTCCCCCCCCCCCCCGGGGGGGCCCC

CCCCCAAGGGCCCCCCCCCCCCCCCCCCCCCCCCCCCAAAAATCCCTAAAAAACCCCCCC

CCCCCACCCCCCCCCCCCCCAAAAAAAAAAA

>658

AATCTGGAAGTATGCCAGCATTCATTTTCTGATTTTAGCGCACGTAGTTGTTCATACGAA

AAGGCTTACAAAAGTTTATTATAAATGCGCTTGCAAGATAGGCATCAGGAATCAGACTGT

ACACTTTAGTCAACGTAGCATTTCATTCACTGATGCAATCTTAAGAGAGATTGATCAAGT

CATATTTTAGACTGCTTTGTAAAAAAAAAAA

>659

AACATGATATCACTTGAGCCCTTTTCTGCAGAAGTCCATCTCGAAATAATGTTATGTGAA

TGTCGCTAATTTCTGCTTTTTCTTTTCCTGCCATACAATGCTTTGACAACACAGCAAAAT

GTACTCGAATAATAACTGACGAAGATTTGCCAAACGTCATTGTTGAAAGTCATTGTTTAC

TGCATTCAAACTGTTGGCAGAAAAAAAAAAA

>660

AAGCGGAAATGGGCCTTCGCAAGATATTGTTTGATATCCGCGCGCTCATGACCTGTTTTT

CAAGACATGAAAAGTGCACACTGTAAATCATGTCCATGTGTATTACATCTGATGTATGGT

GTAGATCGTCAAGAAAATACCTCGTAAATTGATGTTCTTTGAATGGACACAATCAGCGCG

GCCGCGAGGAAGCCTTCTGCAAAAAAAAAAA

>661

TGATTCGCATATCGACAAAGATGTAAGACTCAGACCTACTATGGCCGCTATTGGAGATTT

GTAAGACCCGCATGGGCTCGGGCAATCTCCTCCGCCTAGGCATATGATTTCCCTCTTTGA

CGTTGACCAGCATAACCTGGTGGACAGTGCTATGTGCACAACCAAACCGTAAAGCCGTAT

TATTTGCAGTTTTTGCGATCAAAAAAAAAAA

>662

CAAGATGTTAACAGCAGATGTCATAGCTAAGCTACGATAAGCGTGTGGAAAATGATCAAA

GGTGTGCATTGGATCGTTCAGTTTTGTCTGGCATCAATATCGAAAAAAAAAAAAAAAAAA

AAATCGTAGTCGCAGCATTCACAGTGAACCAACCGAATGTCAAATTCGATAGCTGTATAT

AACTAATAGTTTCTATGCCTAAAAAAAAAAA

>663

TAAAAAAAAAAAAAAATGAAAGATCAACCTTTCTTGAATCCAGCTTCTTCGTAACGCTCT

CAAGCCGCATCAGTGCCCTTGTCCATATTTCGACTATCCACGGAATTCCAAAGTGCTTCC

AAAAATTACTGCTATCCTAGTCACTTTTGCACATATGCTGCTATAGCAATCGGAAATAAG

AATGATTACCCCATAATCGCAAAAAAAAAAA

>664

TGTTCAGCGCCATGTGTGTGGCGCACACGTGCAAGGAAAAAGACAGCAGCATAGCTTGTA

CACTTGTTCCCACATCATCTCACATGCCTGCTATCAAATTTTTCAGCTTCCTCTGTGTCT

TCTTCTGAAAAAAAAAAGACATGCACTGGAGTTTTGATATCATGTACTCCTGAAAAATAA

AGCACTATCAAGAGTGATCGAAAAAAAAAAA

>665

TGTTTCCGTGGCACGGTGTTGGGCATAGCACACCGAGGGAATCCCTTAACATACGCTTGC

TAAAGACATACTTTACAGCCGCCATGAATCATATGGACCGCATGCATCCAACTCAGGATG

TGTCCATTGCTTGATCATAGCGCACAATGTAACATAGAATGACAATCACAGATAATTTCC

ATAGTTCTTAGAGGCAGCCCAAAAAAAAAAA

>666

CTTGTACAACAAAGCACTGCACATGGAAGACACAGGGTTGCCTTGTTCACAATACCACAC

CTAGACGTATGTCATTGGGAACTAAATAATACTTTGTGTGACGACAGTCGCACATGCTTG

ACAAATGCGTGGCACATCACCAGCGTGGCAGCATAAACATGCATAATCGTACACTGCTAG

AAATTCTCTGGAACAAGCACAAAAAAAAAAA

>667

TGAGTGAGTCGTTATTACCACACTTCTTTACGGCAATGAGTGTCGAAAACAGTTCTTTGA

TATCGATTATGGAGCTGTGGCCAGTAGTTTTGGGTTTTTTCAACACCGTCGCAAATACAA

TGGTGCCAGGGAATACGTTGATGATCCAACCCACTCTAGTTTTACTTATGAAGCTTTCAA

TGCCATGGACTACTGTTGCCAAAAAAAAAAA

>668

GAAGACCACTGCGTTCTCCGAAGACCACCTGCCCGCCTTTGGTAGAAGAGGCACGGGTCC

AGCAGCCGACCTCTCGAGGAATCGAAACATGCGGTCTCGTCCCGCGTCGATCGCGCTCAT

CTGTCCATCTTTTCACGTTCCAGCTCCCTACAGACGTGTGCATGGAACAGCGACACCCTA

CACCGCGGGTGAAAACGGTTAAAAAAAAAAA

>669

GCAGGATGACGGTAGCCAATCCGATCGAATGGACGTTCTCATGTCCACAGTTCTGAACCA

CTCAATAGACCTGCTAGATGAGGACGAAAGTTTCTAAAGAGCTTGTAGGATAGCTGACCT

GGATGTACCGTGCATTTGTGGTGATTTGATTGACGTTGTTCTCATCTTAATGAGAAAGGC

TCGGGATGTCCATTTATGTCAAAAAAAAAAA

>670

TTACCTTGTGACCAACCGACTCCTACATATAGGAAAACTCACATGCCGTGTCTGTCTACA

AGTCTCGTTGTCATGGTGCTAGTTAGCCAGAGTGTTTTGAAACAATATTGAGATAGTCCG

CCTGCAATGATACACAATATAACAGTATATATTACTGAATGGGTCAGACATGCATCAGAT

GTGAGACATTATGTTTTAGCAAAAAAAAAAA

>671

TTGGGTCCGTCATCGTTGGAGATAAGTACACGCATTGGGCGGTTCGACCCCAGTGCCATA

ATTGTACGCGTGTGAGATATGACGTGACGTTGGGCCCTTCCACGTCCAATTCTAGAATTG

CTATCGGGACCAATTCTTGGTCTCGCGACCGTGGCTCTTTGGAGACGGGGGTAAACGCGC

AGCATGCGTTACTCCAGGACAAAAAAAAAAA

>672

TAATACTCAAAACCAACGCATCAAGCAAAAACCTACGGCCCAAGGAGAACATATCTCCCC

ATCCCCATCGAGACAGTATGCTGATCTATGGAAATCCATATGAAACAAACTCTTTGCGTG

TTTCATATGCAGTACCATGACAACTATTCTCTTGCTCGTGTCCCACGAATGAAAAACCGG

CGTTGGCGATAGCGTTGTGTAAAAAAAAAAA

>673

GTCCCATACACGAACTACGTAGTCGTCGTGCCTGATGCTGGCTAGGCGTTTGCCGTCGGG

GCTCCAACTCACTCTGTCGATATCGGAGGCCAGCTCGTCACCGTGAAGATTGTCGAGCTG

AAGGGAATGCAAACCCGCTATAAGAGAACAGCATATTGATAGTGCGAGTCTCATGTCATG

GATACAGTGTCGACCGATTCAAAAAAAAAAA

>674

CTATATCAAGGGTTTGTAATACCCATGAATTGTTCCATGTCACACACATGTTCCACTCTC

TGTGATTTTGCTACAAGTATCAGCCTTTCGATTACAAATAACGATCACGAGCTCATGCTA

CTCTTACTGTAGTGTTCATGGTACAGCTATTGATGATTCATAGAAAAAGCTGTGTGAAAG

TTTCACGTCATGCATGAATGAAAAAAAAAAA

>675

GGTTGCCATAAAGATTGATAGTCTTGATGAATCAACCCGCAGCCGGACCTGTTGCTTACG

CTACAACCTGTGTGTCGTAGTACAAAGTTGTATAAGGTACTAGTCAGCAGGTCAGGAATT

TGAAACTAGCTGATTAGTTGTGAATAGAATGGACAACTGAGAACTTGAAACCTTTGATCA

TGTAGACCAGAACCAACGGCAAAAAAAAAAA

>676

CAATACTAGACTCGTTCACCTTTCGCAGAGAGAGCTCAGGATTCCGACGAAACCAAAGCA

TCTGAAAACACAAAACAATGAGTGCTCCGGTATTGCCTGCACTGCTGGCAGATGGTTCAA

AAGGGCTGGTCATCCATCCACTGGCGCTGCTGCTACCACTTGGTTTTGTGGGCAGAGCAT

CGAGGCGATTCAAATAAGAGAAAAAAAAAAA

>677

CAGACGATGATGACGACCTCAACTATCTGTGAGATTGGCTTGAAATTTTGAATACCTGCC

AAACTCACAGCCAGCGAGTGTCAGGGACAGTTCTCATGACAGAGAGTAGCGCACCTCTCT

TTCAAATGTTTGGAAATTTCGAACACGTAAATAACCATGACAGCCATAACAGCTGTGGCA

GTCTAAACAGTTTCCTGATGAAAAAAAAAAA

>678

GGCAAATAGAGCGTTGCTTAATGGGTAATCCGTTCGTGAAATCCATCAATCGCACCGGTC

ATAATGAAGCTTCCATAGTGGCAACTTGTCCAGAACGAAGACTGTATTCGGAACAAATCA

GCTCCCCATCTTCCGACGCTGATTTATTTTAGAGTGCGGTATGTATCTTGTTTAAAAGCG

TTTGATGTTCCCGGTGTTTCAAAAAAAAAAA

>679

TGGAATTTTGAGAAGTATACACCGCACCATTTAATCGTGCGGATGAGTATCAATCATGAG

AGTGTTTTCCACAAAGATGGCGACCAATTGTTTTTCAATTGGAGTTAGCACACACACAGA

CATCCTGTGGGTGCTGGTGGTTGGGAATTTTACGTGTGTCCAACGAGGTGCAAGGATACA

GTTACTTTGACACCAGTAGCAAAAAAAAAAA

>680

GACTGAATAGATTTTTGTAGTTAGATTTTGCCCTTTGCCCCTTGTAAGTGGGAAGGGCTG

TGGGTTTTGGGTTTTGAATCCCCCCCAGGGGTTCCCAAAGTTTTTTTGGGGCTCCATGGT

TTGAAGTTTGGGACCCCTGTTCCCCTGGGGGGGTGGGCAAACCCTTTCCCTCCTTTTTTG

CCAATTGATCCTGGCCTTGGAAAAAAAAAAA

>681

TAAAAGTAAACGTGTAATCCATGTATTGACAAAAGCAGATTAAAGTCATTTATGCAGCTT

GGCAGAAGGTAAGGCACACTGTGTGTATTGAGGTCCCCATTGGAGATTTCACGTCGCTTT

GTTCATTTTGGTAGCTTAATTGAGACAATGGATTTCATCTCAATATGAATTAATTTAATA

CAATCTCCGGCAAGGTTGTCAAAAAAAAAAA

>682

GCATATAGCAGATCATGATACCCAGATATCGAGTTATTGACGGGCGAACATATTCACGTT

CTTTTGAAGCGGTCAGTAACATTGAGACTGTCAAACTGAAGCTATGGGAAGCTGTTCGGG

TCAGCGCTGAGCTGGACAGGGAAAGTACTGGAAGACAACGGATTTCTTTAATTCATTGCC

AACCATCTTGTTATGAAGACAAAAAAAAAAA

>683

GCCTTGTTGCGGGATGCACAATAAACTGTGCGCATGTGTAGCTGTGCAAGAGAGCATTGC

GTATGCGAGCATCTGATAAATTCATTTGCTTACGTATTCGCAATTCAACTGTTGAATGTT

TGTAGTGGAATGAAGCCCTGAACACGATGTTGACTGGTATCATACATAATATAAAACAAA

GTTTGAACTGGGAGTGTACCAAAAAAAAAAA

>684

TTTGGACGTTTGAACACACGCGTTTTGGGCTAGCGAGGCGACGCGCGGTGCATGGCCGTT

GAAAAAAAAAGGTTTGGGACGTATATGAAGCGCGTTTCAAGTACCCAGCCGCCCGGTCCG

TACACGTTCGCTTTGCAGACATCGGCGGACGATGTTTGCAAACAACAGGGATTTTTCGAC

CCATCTCATAAGAAACTTGGAAAAAAAAAAA

>685

CTGCATGGGTTCACTTGTTGCAGGTGTCGTGGGGACTGCTTGGCTTCCTTTGCCATGCAC

GGAGGGCTTCGGCTCGCACTCCCGGTTGCCTTGCACCAGGCTCACCTTGCCGTCCTGTAC

TGTGAAGTCTGGATTCTTGAGAAGCTTGTGAGAAAACTTGTGGACTTGAATTGTGCTCTA

TTGCATTGAGTATTGAATCTAAAAAAAAAAA

>686

GAACACCTTGAACTCTTTCGTTGCTGGGGGCTTCGTACTCGGTGCTATTCTGGTTGCTGT

GATCGGAGTGTCCACCTTCGACCGCGTGAAGCGATCTTAGGCCGTGATTATAGCGCCCGT

TTGGGGATAGTTTTCATTAGTAAATAGTATGCTAATTACATGCTAATATAACGACTCTCG

TCGGCAAGTCACGAGTGTGCAAAAAAAAAAA

>687

AGAAATGTTTTCCGAAGACATGGCTTCACGGCCAGTTGGTAGGTTGGTAGGGGTTTAGGG

TTCATGTCCAAGGCAAGATACATATTTATTGTAGATTTTATTAGGTTTCCATAGGTGTCC

CCTCTGACCTGTTGAAAACACACCAGGTAAACGAACAATGGCGGTGTTGTATGTATGGGA

AGAGTGATCACTATTGTTGCAAAAAAAAAAA

>688

CAAGGAAATCGAATCCGGAGAAACAATTCTATAGTTTGAAAGGAACCTTTGAATTCGAAT

TGGAATTCAAGATGGATGCGAATCAACCCTGCAGATTTTTGATTTTCAGAACACAAACAA

AGCTTTTCAACCTTTAAGGGTTTTGGGGTGTAGGGTTTAGTGCAACATCGCATCAAATTC

TTGGTTAGGACCAAGGAAATAAAAAAAAAAA

>689

CTTGGAGATACTACATCAATCGCCAATTTCCACGAACAGGCGAGAATAAAAGTGCTCAGC

TTTTTTAAGGTGCGGTTTTGATTCAAAACCCAGAAAACAACAGGTTTGGCAAAACAAGCA

AAATGTTGTTTCAATCCTGCAAAGTATACCACCTAGCCCAGGAAAACTGGAACACTCCCC

AAGGGGGCATGTAGATTTTGAAAAAAAAAAA

>690

AGTTACTGGAGAGAAGGTTTTCAGTACATGGATGGGGGTTCGCTTCAAAGTTCCACAGTA

CATGTGTTCAATGGAAATGGGTTGGTATACCCAAGAAGGTGCATGTACTCAACATATAGA

GCACCTCTATTATTGTAATATTGGCCTGCAGATCAAACGATTGTACGTGGGTATATGCTG

ATCACAATCACATGCCATCTAAAAAAAAAAA

>691

GGTTTCTTGTCGATCCATTTGCCTTTTCCTGCCTTTGCTGTCGGAGACTTGGTTTGAGCT

CCATCATCCGGTACCATTGCATTCATGGTGGTTACCTGCGCCGGAACTCCTTTGTTCACA

GCTGTCATTATTTGTGCAGCTTGAAGTTCCTTTTTCTTGGATGCTGATTTTGCTTTTTGT

TTTGTCTCTGATACACTGACAAAAAAAAAAA

>692

AAGGCCAACTGAGGGATGGGCTCCTCCATGACGTCTACCCCAGCAAAGTTGCCGCCTGAA

GAATGTCTCGTTGAAGTCTCTGAATAAACGCTGGCCTTATCACATTGGGAGCCCATCGAT

TTGTCCACCTGCATATCCAGTGGTTCCAAAGGTCCGCCCATGGCCATCACGCCCAGAAAA

GACCCCATGCGGGAGCCCCGAAAAAAAAAAA

>693

CCCATCTTCACACCCCCCATCCACAACTCCCAAAAATACCAACAAAAACAAAAAACAACC

AACACAAAAAAAAAAA

>694

TTGAATATGTTTTTTTTGCTTGAGTTGGGTTGAATGCACGGAATGTGTTTGCACCATCAC

CATTTTCAAAAAGAGTATTTTTTACAGTTGCACCGTGGATAGCACAAAGAAGTGCAGCAC

CAAGAACACCAGCAACACCCATCATGTGGAATGGGTTTAATGTCCAGTTATGGAAAAAGT

GGAAGAAAAGAATGAAACGGAAAAAAAAAAA

>695

TTGAACCCACATCCAACAGAGCCTTTTGATTCCCAAGTATCTTTCATTATTTTACCAGAA

TCCATCCTACTTCCGAACCTTGAGCCATTTCAAATAGGAAGCATGAATATGTGAAGTTGC

AAGAAGTTCCCTGCAACAATGAAGATATAGATGTTTTCCCTGAAATACTCATTGAAAGTG

CAGCTTGTTTTTGCTCCCTGAAAAAAAAAAA

>696

GAGGAGGACCGGCTGGCGCGCCTGGCGATAGATCGGTGCAGGTCAGGAGAGCGGGAACGA

CGTCGCGTTCGCTCTCGAGATTTGCTCCGCGAACGGCTCGAGCGCTTCCCGACCCGTTTT

CGGATTACAGGTGAGCGGGAGCGACTGCGACGCCCCATCCCGCTCAGTTACCGTGGACCA

GGACAGAAGCTTCCTCCAAGAAAAAAAAAAA

>697

CCCAAGCGAAACATGATCGCAGGTATCACATCTACATAATAGAATCGCACCCAAGTTTCA

CGCGCAAGCTGGAGATCACATTCCAGGAGAAAGTGTCACGAAATATCCTGTGTTGTGGGT

GCTCACAGGAAATCGTGCACCCACAAGTCACTCCGAATACGAGTGCAAGCACGCACTTAC

AGCGCGTCGAATCGACTCTTAAAAAAAAAAA

>698

CCTCACGCACAGATTGTCAGAGTCCTAGAAATATCCCACAGAGGCTAGTAATGCGCAACA

GATTATTGCTCTACTTAGCATCTAGGAGTACAAGATACTCAGGGTACTTAGCATTGTGGA

TTTGCGAATCGATGGTTCACATCTAGATATTTACCGGATCAAAGTACTAAGCTCCATAAA

TATTGGTCGGCCATCTTNCCAAAAAAAAAAA

>699

GCGCCCTTCCGCATCCTGGTTTACTTCGTTAACCGAGCCAGGGCGGAACCCCCATCGGGG

CCTGGCTGTCGACTGGTATGCAGATTCCCCATGCTTTGAACTTTGGGCATGTGCTCTTCC

GCCACTGCTTGCTCTTTGCCATCCCTTTGACCAACTAGAGAATTTGTTCCACCCTAAAAG

GCCGTGTGCATACCANCCCGAAAAAAAAAAA

>700

ATTTGAGACCCAATACGGGATTCGTTTAGGCACACCAACAGGTGGTGACCAAACCCCAAG

ACGGAAATCCAAATGTGGCACCAACTAGGTAGGGCCGGGGATAGGGCGTTCGGCCCTTTT

GCACTCAACAACTGGTTAGGCACCAGTACCAAATAATTGCAGTAATAATAGCATAGAAAT

ACTTGTAATGTCTTGAGGATAAAAAAAAAAA

>701

ATCCCCCCCTACGGATCACGCGTGCACTTAAAAGGACGAATGACCAAAGCTGGTTTGGTT

ATAAGAATTTCAAAATGATTTTGTTGTCTCTGTGTACAAGGCGCAGATGTCAGCCCTTGT

TGCTCGTGTCCCTGGGGTGTGTGTGTCCTAAAGTTTATCGCAAACCCCTCGTGTATCATA

CATGATTCTGTGTTTTATGGAAAAAAAAAAA

>702

TAGCTCTAACATGGATACTAACTATTTCATTTGTACAACTGAAGTGAAGTGTGAATGTAC

ATTTTGTTTGTGAATAAATTCATTAGACAGATTGTACATTTGGCAACAACTTTTTCACTA

AAGTTCATGATCTTTCCGTTGGTTATGACTTTTTCAAACCATCTTTCTGCTGTTGAAAAT

ATTGTTTATGGTACCAGCATAAAAAAAAAAA

>703

CATTACCCCCCGAATTCATGGTTCTCCTCTTCTTTTCTGTTCGATACTCTTCACCGACGC

GCGAGATGTGAAACGAATCAAATTTTGATGTGTGATCTCCGACTGTGTACAAAATTTAAA

GAAATGAGCGCGTATGAGATCAAATCCGCGCAAGGGTGCTTGTAGTCTCGGGACTCGTAA

AGGTCGCAACGTGCACTCGGAAAAAAAAAAA

>704

GAGGCAAACTTCCTCTTTGGTAATGCGATCGGATACATGCCAAACCTTCGCTAGTCTTCC

AAAACGGTGCATCGTGCGGATATATTTTTGGTGGATTCACAGCGCTTCAATGAATGAAAT

TGAAAGATGAAAACAGTTGAAGTATCAGCTTCCCCGTCTCATATATTGATGTATATGACG

ATCAGACATGGTAAATCCATAAAAAAAAAAA

>705

CGATCTTGCAAACGCGCTTTGGGGCTTGCGAAGCTAGTGACCTCAGAACAGAGGTGACCG

ATATTCGAGGGAATTCTAGGTGCTGCGATGAGCATCTGAAGGCTCGCCGTTAACATTGAT

AAATGCGCAACATACTCTTTCGCATGAGAATCTCGACATGGCGAGTGGGTCAGACCAAAA

AGGGGGAGTTCATTCCCCCCAAAAAAAAAAA

>706

CGAACGATAAGGCCGCATGACATGGATTACAAATACTGCATGTGTACCCAACTGCTCAAT

CGAAGTCACCGGCCGTCGATTTAGCCGGCTCCAAAATTATTAGCAAGACCATACCAGACA

TTGTGCTTCACGTCCCTCAATGGCACCAAGTCCCAAACATCACGCCGGCATTCGCAATGT

TAAAGGACTATCTTACCCTTAAAAAAAAAAA

>707

TTTTTTTAAAAGGGGGGGGTTGTTTTCCCCCCCTTGAACCGGGGGGCCCCGTTTTTGTTT

CTTCCCGCCCCGGCCAAAATTTTGGGGGGGTTTTGGGCCCGGTTTCTTTTTTTTTTGAAA

AAGATTTTGGTTTTTTGGTTCCCCCCCTTTGGGGAAGGCCCCTTGAAGGGTTTTTTTTGG

GGTCCGTTTTTTTTTTGGTTAAAAAAAAAAA

>708

TCAAATAGATTCGCCACATTGTTTTGGAGACGTGCTGACAGAGTATGCGTATGGACAGGA

ATCAGGTATTAACGCCGATAAGGCGTGAGGAGGCCACAAATCTTGAAGCATCAGTGTTGC

TCAGTATATGTGAATGATTATTGAATGCACGTTGTTGCTTGTGTATTACTGATACAAGAA

TTATAGACCTCGGGTTCATCAAAAAAAAAAA

>709

CTTGATGGTCGACATGTGCTCCCAAGGGGCACGCGCGGTAGGGAGACGCCACACACACAA

CATCAGCAAGGGGAATCTTGTAGTACTTTGCGACTTCAAGTCGTGGATTGAGCACATCAG

ATTCAGCTGGCCAGGTTGTCATGACTTCGGGTCGAACGTTGCGCTTCAGGTAAAGGCACG

GCACGGTAGCTTTGTCGTACAAAAAAAAAAA

>710

AAGGTTACCAGTAGAAAAGGCCATCAAGTCTTGTGTGCTTGGATTCAAGAGCATAAGACT

TACGTAATGATGAATGCATTGGAGCTGCGGTTATCAAGCTGCATCGCTAGGGCTAGCCAC

CTCATTGCTAACCACTACAGTGTTCAGGATGTTGCAGAATACATAAATGTGGTTTGCAAC

AATGACTCGATTTTTCACTGAAAAAAAAAAA

>711

CAAGCGTGCAATCTGAGCCGCAGATTCCAATAGTTTGTGCAGCTCTGGATGCTTTTCAGC

CGCAGCCTAGCGTGCAATCTGGGCCGCAGAATCCAACAGTCGCGCAGGCTTGTCAGTATC

AAAATATCAAGTATAGATATATCAAAATATCCAAGAATTAAAATATCCAATTTATAAAAT

ATCAAAATATGCATCCTCTCAAAAAAAAAAA

>712

TGACACCATCCCATACCGTCGGCTTTGTTGGCTTGAAGTTCTCAGCATGAGCCTTTGCTA

TCCGTGCTCGGAGCGCCTCAATGTCACGCGCTTTGTCCTCTTCATTCTTTTGATCATGTG

AATTTTCCGCATGACTGTCTATACCCACCAGCACTCCCCTGCTGGAATTATGACTTGCAC

GGCGGGATGTCAACCGCGGCAAAAAAAAAAA

>713

ATGCTATAGATCACTACAGTAGTTAAGTGTTCACATTGTCTAGATATTAGGAACATTGCA

ATTGCAGGCTACCAGTCAACTGCGGAGTACGATCACTTTGAATAGCTGGGAGGTTAGCTT

GTGAGAAGAGCATTATGGGCTAGTGGAGAACCATTTCGAGGCAGAATATGAACACTTCGA

CCGAGATCAGATTGTATGCGAAAAAAAAAAA

>714

AGCAACTTCCCAGACACAATTTCCGCACCAAACCATCCGTTTAAGCAAGAATCAGCAGAA

GAAAACCATATGCACACCAGCGAGTCTTGTGAGGATGAGGTGGCGGAGACGAGAGATGAC

TTTGAGTTGGACCGGGATGCTGAGCTTGCACGTCAACAATTTGCGCATGCAGCCAAACAA

CTGGCTGTTCAGCAGCAACGAAAAAAAAAAA

>715

TTGGGGTTTAAAATACGGGGTTTGTTTTAAATTCGGGGGCGGGGGGGGTTTTCCCCCCCG

GGTTGGGGTGGTGGATTTCCAATTGGGCCTTTTCCGGGAAATTTTGGGGCCCCAGGTTTT

TTTTGTTCCCTTTGGGGGCCCTTGGTTCCCAGGGGGAAATTGGCCTTTGGGATTTTTTTC

AAATTTTTTCCCCCCGGCCCAAAAAAAAAAA

>716

TGTTAAGGGGGGGGGGTTTTGGCCCGGAGGAGGATTCTTCGGGCCCCTTAAAATTTTTCC

CCAATCCCCCGGAAGTTTTGAGGGGGTTTGAGGTTTCTTCGTTGGGGGGGGAAGGATTTG

TTTTTCCCGGAATCCATTCCTTGTTTTTTTGGGGAAACCCTGCTGTTTTGCCGGCCCGGA

TTTTGGTTTGGATTTCGCTCAAAAAAAAAAA

>717

AGCCTCAAGCAAAGCACGGACAACGGGTTCATGGCCTTGGGAGGCAGCATAGTGCAGCGC

CGTCTAACCATCCTTGTCAGTGTGCGACACTATCGCGCCTTCCTGCAAAGCCCGCTGCAC

CTGATCTAGTTTCCCACCGCGAGCAGCCGCGAGAAGCGCCGAAGTGGACATTCCGTTTCA

CGAGGCCCGAAATACCAACGAAAAAAAAAAA

>718

GTATGGGCACGCCTCCGAAGCTGGAGGGCGTCTTCTTGGGCTGAATGCGCGCCCCCCCTC

CGACGGACCGCCTCCAGCCTCTTTCTTGATGGCAAGCACCCCAGGCCCCTCCTTCGGACC

CTGCATCGAAACAAGCAAATACAGCCACGCAAAGCGCGCTGATCAGTTCATCGCCTTGGT

AGCTCCAGAAATCAAGCTTCAAAAAAAAAAA

>719

GCAAAATACATGAAGTACGACCTGCTCGAGATGCGTAGCCCAACTATGCTGGGGAATATA

GATTTGTTGTGTTACCTTTTTGCGCCCCTTTTACTAAATCTATGGAAAACAGGCTTAGCT

GCCACATGTGTTCGAACATGAAAGAATAATTAGCGAGACGTAAACAGTCAATGTAAAAGG

GGTGTCTCCAACAAAAGTGCAAAAAAAAAAA

>720

AAGTGTTTTTTCCGATTTAGATACCGGAGACCGTGGAAGAAATAGCAGTAATGTCGAGGC

TGCAAATGCTAGAACTGTAGCCCCCACCATAGCTATTTTTAAAAAGTTTCCAGCAGAGCC

CCCGTGCCCAACAACTGCTTTAGATTTTGCGGGCAATGCAGTGTTTATAAATAAAACATG

TAAAATAAAGGATATTCTTGAAAAAAAAAAA

>721

TTACACCTGACACATCAGTGAGATCGGATGAGTGGTAGCGCTAGCAGATATTCACACTAG

TACTTCCCACTCGATTTCATCTCTTCCACTGTGTGGCTAGACGTGCACTTCTAGTAAGAC

CGAAAAATATCAAAGCCACATGCACTTTTGATAAACAAGACGTCAAAATCTTCAAAAAAA

AATGCTGAACGTCCCGTCCCAAAAAAAAAAA

>722

CCTCCCATGTCAGTGTCCCCGATTCCTTTTTTTGCAAATCAAGAAGGGCCCTTTGGGTTG

GGAATTGACAACAGCTTTTCACTTTGTCCTCCGTTGCATGTGTTGCTGGTATAGTTTCAG

GTCCAATTTGGGTCGTGGTGTTGGGGTTTCTCAAGAGTAAGTAAAGTTTCTGTAAGCTCG

TGTTGGGTCCCCCTGTCCCTAAAAAAAAAAA

>723

CCTTTTTGCTTTGCTTTATCCCCGCCTTTGGGTTGGTAAAGAATGAGGTCAATACGTTTG

AGAAGCTCTGGTTTTTCTGTCAACACCTTCTGCACAGTTCCCGTCTTTGGTGCACACACT

CGCATGTACATCACGCTGGTGCCGGTGGCCTTGTCCATTTTGCGCATGAACCGTCGAATC

TTAGTTTGCAGCTCCGGGATAAAAAAAAAAA

>724

AACGAATTTGTTACAGATTATAAGTTTGCAACATAACTAGGGCTTAAGATTCGAGACGCA

GGTACTGTTGAGAGAGCACCCGGCGCGAATGTTCGCACATCCCAACTTGTCGCTGCTTCA

AACGACAGTTGATACTTTGTTCTCCAGGCTCTGCATTGACCCAGCAATCGTGCAAGACTG

TAAGACAAGCCGTGTGNGACAAAAAAAAAAA

>725

TGTTGTATCCATGAAGTCGGAGCTGGTCGTACTTGATCCCAATGTGTCAGCAACCACCCC

GTGGTGGTGCATTTTTTCTTTTGGATTTTCCAGGCTGTTACAGGGGAGATATGATTTCGG

CACAGAGGACTTGCTGGACTGTAAGCAAACTTCATGTTGCGGCTTATTTGACAGCCCAAA

TGGCATTTCCTGGAAAATCCAAAAAAAAAAA

>726

GATTGATGGGAGACATGCAAACAAGTCTGGAAAAGCCAGCACTTTTGGTACAGGCGTCGA

CTTTGGCGAACCGCGTCGCTCCTCCGGCTCAGGCACGTACGCGAGAGCACATATCTCAGC

ATGGTGCTCTTACTCTGACCAGAGAATTCTTCCCAACAACCAAGGCTTGAGTAACAGGGA

CGCAGGGAAGAATTTAAGCGAAAAAAAAAAA

>727

ATGATCGCCTTCAAACGTTTGAGGGAGCCAGTGGAGGATGGATGGACCAGGCTGCGAACC

AACCTGTCAATGCTATTTTATTTGTGCTTTTTGTGAAAACAATTCTATGTATCGCATTCC

TCGTTCAACATTAACATAGAAATTTGTGGTTTCCTTTTAACCACACACGTGTGAATTGTG

TGGTGTGCATTATCTTGGTGAAAAAAAAAAA

>728

AAGGCATCGAACATCTTTGGTCGATTTAATACGAGTAAATGAGTGTGCGCCATTCCCACC

CTCCCGCCGAAAGTTAAAAAAAAAAA

>729

GGCCCGACTTCTGCAGCAGGGACGCAAACTTGGTCCACGCCTCCGACTCCTCCTTCATGG

GCAGCACGAGCGAGCGCACCGCCAGCAAGCCTTGCCACACCTCCACTTTTTTTTGCACGC

CCCGGATGCGGTCGCGCCACATCTGGCGCATCAGGCCGCGCCGGTCCTCGGCATCCTGGA

CGTTGCCCACGCCCAACTGCAAAAAAAAAAA

>730

CAACGCATACAGGGCCTGACCGGAACCAAGAATAACGACTCTACTGCGTATTCAGGCAGG

ATGCGTCTTGCAATCGAACGATCGGATTTTTGAAAACCCGCTCTTGCTCCGGGAACTTGC

ACACGACACAGCAATCACTGAAACCCGCGTGAAACTGTTACACGGACGCACGTCTGGAAG

CAGATGACCGCTGGGTCCCCAAAAAAAAAAA

>731

GTCTCGCGGCGCGCGCGTTTGCTCTGATTTGTGCACCTAAGGGGTACGAAGTTGAAGAAT

TTGAAAAATTCGAGATTTCTTCGGGCCTGGCCATGGCAACGGTCTTCAACGCACACGTGC

GACGCGCGGATAGTGTACGAGGCCGCAGCACGTTTTATACATACTCTTCGGGGCGTTCAG

CATACAAAGCAGCCTAGGCTAAAAAAAAAAA

>732

AGCGGGTGTTGCAAGGCGGGTCGTCGCTTCCACCTGGCCAGCCTCGGCTAATGGGAGCCA

CCGGGTGAGAAGGCGGGGCCTACTGAAGAACTTCAGATGCGGATGTGAAATAGTCGGGCA

AGTCCGAAGGGCTGCACTAAGCACTTCCCCCCGCCCCGGCCGCGGCACCCGTCTTGCCCT

GCTCCTTCTCCTGCTCTCCGAAAAAAAAAAA

>733

GCTTCCTGGCTGAAGCGTCTCTTTTACGGTCTCTGAGTGTCCGCCCATATTGTCCGTCAG

CGAGACTGATACTGGAAATTGCACAAAAACGCACCCGCTTGTGTCCGCGTTCTTTTTCGT

GAACACGCGATTCAAATGAAGCAAAAACTAATACGAAACCCGACGTCGGTATGTGGGTAT

CAAGAACAAAGGGCCATGTGAAAAAAAAAAA

>734

TCATATTTACAAGACACCATTGTGTGTGTGCATGATGTCATTTCAAAGATTGCTGGAAGG

TGGAAAGACTTGGGGCATTGCAAAGTACAAAGGTTTGTCTGGATATTTGAGCAAGTCCAT

TTAGTGCAATCACATCAAGCCCTTTTAGCACATGACATCGGCATTCAGGCTGTGTCCCCT

CATTAGTTATTCTCACTCCCAAAAAAAAAAA

>735

CGCGCACTCAGCTAACATGGCTCCCCTCCATTCGGGTAAGGGTTGTGTTGCCACACATAT

ACAACAAAAATACAGTTAAGGTGCAAAATCTTGGACACATGCACATGCAAGGGTTGATAC

GATGGAGCTCTGCTTCTCCAAACAGGGTCGGTGAGTTTGCTGCTTGTGCTCTTGCTGTCA

AACACATCAATGGACAAACTAAAAAAAAAAA

>736

AATGACGCGTATCTCTCTGGCCAGGCACTAATCTATCAATCATTGGTTTTCATGTGCTGT

TGATGCTACTGTCAGCAATCTGGATGCAGTTGTCGTGACTGCAGCTTGGTGAAACTAAAG

TATGGACTGCTTCAATAGATTTCGTGTGTTTCACACACACCCTGCTTTGATCTCGTGCAT

GTCCATGTAGAAACATCTCTAAAAAAAAAAA

>737

CGAGGGGTTCATGAACCAACATACGGGTTAAGCGATTACGCAGCGCTTGGCGGACATATA

GAGTGATTCCCCTGCTACGCATGATGAGAACACCAAAGCGCAAATTCGTGTTTTGTGAGA

CAAGGCACGGGAACCTGTGACACGCTTGAACAACATATGGACATTGTGTGTTAGTGTGTG

ATGAATGTTCCGTTGGCCGCAAAAAAAAAAA

>738

CTGAATGGGGTGTCACAGTACAAGAAGCATCCGTAGATAGCGATGGAGTAGATATGGCCT

GATAACATTGAACAATTACATATGCGAGGAATATGCGAATAGAAAGTCGATATCTAGACA

ACACGTCGTATACAGGAACTCTGACAGAGCGTTGACATAGCTTTGTAGGATGGTCATCTT

GCATCTTAGCTCCCTCTTGTAAAAAAAAAAA

>739

ACTCAGTGTTAAGAATCACCCAAAAGACCAACGTTCGTTTCGATCTATCTAAAACTACGA

GACCAAGCTGGTATTTGATGGCAAGAAACTGATGATCCAGCAAATAAGACTTCCGCCGTG

GGACACTACCGCACACATCCCTGACGAAACCTTAGAAACTAACATTTACTGATTTTTATT

GAAACTTCTGCAAATGCCTTAAAAAAAAAAA

>740

CCCCTTTGGTCCCCCCCCCCGCGCCCCCGGTTCCCCTGGGGTTTCTGGGAAAAGGGGGCC

CCCGTTTTCCTTAAAAAAAAAAA

>741

TCGTCACACTGCAACTGCGCGCATATGTTACAATGATGTCCGCGAAAATGAAACCCTCAG

TAAGTATCATACGGGAGCTCGTACTGGGAGACAGCTATGAATTGCTACATTCCTGGACTG

TTCATGCTCCGTTCATTTCGGAGGCAACACTATTTTGTAACGAAATTATTTGGAACACCA

TAGCCACTTTACTTTGCTTCAAAAAAAAAAA

>742

AACACGGACCAGAGGCTGCCATGTGACGAAAAGTGCTCCAAAAAGACCAGCGAAAATGTT

CCCCATGATCTGAACAGTATGATTTTCTATCATCGTGTAATAGGTTTGTTGCGTATGTGT

ACCCGTCGTCTGTTTTCAGGGATGACCGCGAGGTTCACAAGCTCGTCTGGAAAGTTGCAA

GATAGAGGGAAGAAACGNTGAAAAAAAAAAA

>743

ACCCGGGACCAGGGGAGGAGGGTTTTTGTTCAGGCCGGGTTGTTCTTGTTAAGCCCGGGT

GGATACTTTTTTTTGGGGGTGTTCAACCAGGGCTTTTTTGGGTTAGTTATGTATTTTTTC

CCCCAAAAAAAAAAA

>744

TGGGGTTTAGCCGGATTGTTTTTTTTCCCAAGGGGGTCATTTTTGCATTGGGGGATTTTT

GTCCGGGGGTTTTCCCCTGAATTTGGGGGGGTCTTTTGACGCAGTTTTTGTTTTTGGGAA

AGAAAGTTTTGAGGATTTTTCCAGGCAAATTTTTTAATGGGTTTTTTGTTCCAGGCCCTT

TGGAATTTATTATTCCCCCCAAAAAAAAAAA

>745

GGGGCTCCAGGCGCCGCCGCTTCGCCCTTCCCCTTCTTCTTCTTTTTCTTTTGGATTTCG

GGTTCGGGCTCGGGTTCTGGTTCAGGCTCTGGTTCCGGTTCTGGCTCAGGCTCTGGTTCC

GGTTCTGGCTCTGGGCCCTTCCCCTTTTTCTTCTTTTTTTTCTTGCTCTCGGGCTCGGGA

ACAGGCTCGGGCTCGGGAGCAAAAAAAAAAA

>746

CTTTGGATTTTGAATTTGTTTGTCAGATAGAATCTCTCTGTATCATATGAAGGTGTACAA

AGATTGAGGATTTGTTTGCATAATCACAAATGCTCCTCCTGACAGTAGGAATCATGACTA

TCCAGAACACTTGTGATTAAAGACTATGTATGTGTTTCTCATGCCCATGTGAAAAAAATA

TGTTTGTAAGGCAAGTCACTAAAAAAAAAAA

>747

GGTTTTTCCAATAGGGACGGGCAATGCCCCATTTTGGGGGTATCATAAGGTGGCCGGCCA

ATAGCAGGGCACTTCATTACTTACATTTGTCCGGGACAATATTTAGATGGGAAAGATGGA

TTCCCATATCCCCCGTCCTAAAATACCCTGGGTACCCTGAACTCCTAGAGGGTTATTAGA

GTAATTGCTTTTGGGCCGTNAAAAAAAAAAA

>748

GGCTCCCGGGCAAAGTCATTTCGACATGCGGACGCAATGAGGCGATCTCCTGCACCTCCC

AACTCAAGTAGAGCCAGGGCAAAGTTACCACGTGCAGCCAGACGGGTCAAGTAGTATGCA

TTTGCATGATAGCCATGTTAGCCGTGATAACCATGGTTTTACAAATATTATATTTCTTTT

ATGAATTCACATTCATTTTGAAAAAAAAAAA

>749

GGCCTATGAGGAAGCTGCAAGTCACTTACAGTACCCATTGTATGCAAAAGGATTGTGCAA

CAGATTCATGTAAAAGAGCGTCGGGCTTTTCTCTGTGGGAGGTGGTTGGCTCGGCTGATG

ATCCTTCATTGGTCGTACTGGCCGGTGAATGGCCGTTCCAAGAGCTTAAACAAAACACCC

ATCGTTATTGGGATGGGTACAAAAAAAAAAA

>750

GCATGTACCATGCACTTTGCAACATTCGCCCCTGTTCTTTATGCTGCTCTCTTTTTCCAG

CCCGTTTGTGCTATGAGCTTCGTGTTCGTGTCCGCTGTGATAATCCGTGAACGCAGCGCT

AAGTTTTTCTCCCCTTTTAATATCTCCCTGGTTCTCACCTGAAGGGTGCGACAGCAAGCA

TGTGGGGAAAGGGGGATATTAAAAAAAAAAA

>751

AGTCACCCACACAAATCATAACACAAATCGCTTGCGCATGCACCCAAAATCAAATTTCAT

GTCAGTTCACAATAACGGCCTTGTTGCTGAACCCCCAGGGCAACTAGTATTTCTATGTGA

TTGGGTGCAGGTGATTGGAACATTCACTTAATACTCGCAGATTACTCGCCAACTAGCGCG

GACATTGATGTATCATCGGCAAAAAAAAAAA

>752

TTGCCGTGTTGCCTGCCGTATCTCTAGGTATGCCTAGTCTACTTGTACGCGAAGGCGCTT

CGACAGATTTCACGGTCGAAATTAAGATTAGCTCATCGCGCGAGTCATCAACTGTACACC

AACACCTCGGGTTAGCCGTTTTACTGGCGAGACGTATGTTTAGAGTATTCATTAATACGA

TATTTCCTGTCATTACGTGCAAAAAAAAAAA

>753

GCTGGACCAATGCCAACCCTTGTTTTGTTGCCTTTGCGCAGGCAGTCCGCGATGTTGCCC

GATTCCGGCCTGGTGTAGATTGGAATATCAGTTGAGCCCCTATTGATACTCCTGATTTGG

CTCAGTCAGGTCAGCTAGACGGCTTGGACACGTACATTATGTGTACATGTTACGGGGGGG

TACCCGGAGGGTTTTTTTTTAAAAAAAAAAA

>754

GTTTCTTGTGTGGTGGAAGCAAGAATCATCTTCTTTACTGTCCTTTACATCATACAGCCC

ACGGCCAGCACCCCGCATCGTACGGTGACTGCGGCTATTGTGGATATCTCCTACTGTGAT

GGATTACAGATGCAAGAGATGGTGCGACTGAACGCCGAGCGTGTCCAGGGTACGCAACAA

GCTTACTTCCGGGCAGCAGCAAAAAAAAAAA

>755

GGATTGTTTCAATAGTCGAGTACCGTGACGGTGGAATCCCTGTTCATATGCGAGGAAGAT

CGGGTGATGTCGCAGATTAATAGTTAGCATTGTGATGGCATCGTCACATCCACAAGTGTT

ACGTGGGGTTACGCAATAGTTAATGACCACGATTATGATACACTGCTTAGTATGGTTGAT

GTCAAAGGGCTGTGGCTTTCAAAAAAAAAAA

>756

GACCCCACAGACCTGCGTGTTTGGTGCGCCTATTCCAGTGCAGAAAGTAGAAGATCCCAC

TGACGAGCAGGTGCAAGCTCTGCATGCGAAGTATTGTCTTGCTCTCCAAGCATTGTATCA

GGCTCATAAGATTGAGGCGGGCTATTCAGAAAAGGATAATCTGATTATAAAGTCCTCATA

GGTATTAGCATGCCCGTGGCAAAAAAAAAAA

>757

CAAGTATCATGGACTGCTTCATCATGTGCAAGGGACATGATACCCTGTGTTCATTGAGAC

AAATCATGTAGCACTCCCTCCACTTATCCGAGCCTTCCATATCCTCAGTTATCACATGAT

ACAACATCTGAAACAAACACTCATTTCCATTATTACGGTGCTTTGGGTTTCACAGCATTG

GGTTTGCAACAGAAATCAAGAAAAAAAAAAA

>758

TGACCAATGCCAACCCTTGTTTTGTTGCCTTTGCACAGGCAGTTCGCGATGTTGCCCGAC

TCCTGCCTGGTGTAGATTGGAATGGCATTTGAGCCTCAATTGGTACCCCCGATGTTGCTC

AGTGAGTGGAGGTAGACTGCTTGGACCTGTGCCCTGTGTAAAGTTTAATGTGTACATATT

GTGCGGGGAAGCCAAAATGGAAAAAAAAAAA

>759

TCTGCGAGTGCGATAACGTCACAGATTATATTGTGGTTTCTTTTTGCGGTTGGTGATAAC

AGCACGAGCGTGACGACCAAAGGAGACAAAAATCGATCTAAACGTGAATCACACTGAAAT

TGTTTTGCGCTTAGCTCGTGTGTAAAAGTCGCGAGAATGTCCAGGCGCAAGGAAGCACAA

GCTTAAATCTAAATACGGTTAAAAAAAAAAA

>760

TGATGGAGCAGTGTGTTGGTGCCTGGTATACCTGCTGCTTCAGTCGTGTGTCGTGATAGC

ATTAGACAGCAATAGCCACTGGTCACCTAGCGTTAGGATATCAGCCAACCAACATCGATG

GTGTACATTCATGCAAGATCGTTTTTGAGATCGAGTACAGCTCATTCAGCAGACGGCGTA

AAGTAAATCTATTCCTCGCTAAAAAAAAAAA

>761

CCCCCGGGGGTGGGGGGGGGCCCTGTCCCATGTTCCCTCTTTTTTTTGGCCCGGTCGTTT

TCGGGCGGGGGTTTTTTTGATTTTTTTTCCCCTGTTTTTCCCAGGGGGGTTTTTGGGGGG

GAAATTTTCCCCTCCCATGAATTTTGGACCCCGGGGGGGGGTTTTTGGTAAAGGGAATGC

CTTGCTTTTTTGGCCCTGTTAAAAAAAAAAA

>762

CGTATCCAAATATGTCGTAGTACTAGGTGGTAATAGCTACCAGTCACTAGAAGAAGACTA

TTAGCCAAGTGATACGATTGTGAATAGAATGAACAATCGGCCATCAGGACTAGAAGGGAA

CAACTACCAATAGCACTGATCGTACATGCCGCCAGTATAAAAGGCGCAGCGTAACATTCG

TAATCACTTGAGCCTCTGCCAAAAAAAAAAA

>763

AACACCTGCCATCTTTTTCAGCATCCTTCCAAAGCTTGCTGAAGGCGTTACTGCAAGAGG

ATTCAGTTTTTCGCCCTTCGGCCAAACAGATGTTAGAGCATTCGGTCATTTGTCAAAGAT

AAGACTGATGTTGTACCATTAACACGAGACACACCAAGTTTGCTTGGTATGAAGTATACT

GCGTGAGCAAGTCACACTGCAAAAAAAAAAA

>764

TTAGCATGAAGTGGCTTCAGCTACAACGTGCCCCAAGTCGCGAAGCATGGCGCACAAATG

TGGTATTTCGGTTTAGCCACGCTCACACGAGCTTATACACGCTTCATTTGTTGTGATTAA

CTGTATATGACAGATCAGAACTGCCACATGCCCGTGTCAGTGTACATTTAACGTGAGGAA

TGATAGGTCCAATCCAGCCTAAAAAAAAAAA

>765

TGTAGGTTTCAAAATTTGCACTCTCAAGTTTGGCGTCCTCAGCCTGTCTTTGCTTGGCCT

CCAGTCCGATGACGGTTGTTTGAATACGTGCCCGCATTTTCCTGTGTTTCTTGCGCATCT

TTTGGTGGGTCTTGGCCAGACGCTTGATGAGATCTTGGATGTCCCGAACCATTTAGAGAG

TGGCCTTGTTTCGTCCCACCAAAAAAAAAAA

>766

GGCCACTTCATACATTGTTGACATTTTTTCATTTTTGAACTCCCAATGGGGGGATTGAGT

GACTAATTAAGCTCCTCATAGCGCACATAAAGAAGGGAAAGTGATGTTCCATAGGTGACA

TACGAAAGGTGTACTGCATAGTTTTTTCCTATGTACAATAAATACTGTAAATTATGTTTA

TTCTGAAGAGATTTTAGCGGAAAAAAAAAAA

>767

TGAGCCCATGCAGCTCAATCGGCAATCCATGGGGAATGAAAACTACGCAAGGTCTGGACA

TCGAAGGCTCAAGCTGCGCTTGTTATGAACTAAAACGCGCATCGCAAATATATCATGAAC

TAGAATTGCCAAAGCGCACACTTGAGAAACAACCATTGAGTTCTTCGATACCAGTCACCA

GTTTGACGGGACGATTTCATAAAAAAAAAAA

>768

GACATGTGCTGCGGATTATGATTCCCTTGTCCCATTCCGTGAGGTTTGAGAACCCCCCCC

CCATCAAGAGAAGGCAACTTAGTCCCAGATACGGGAGATGTTCTGTGGAAATATTATCCA

ATGTTTGCATGGGGGGAATTATAGAATACTCCATCCAATATTGTACAACCAGGTTGATTA

ATGCGGTCTCAAAAAAGCGCAAAAAAAAAAA

>769

CTGTGGGTTGTGCAAGGGTTTCCCCTAAGCCTGAAAATGCTTTTTGGGCAGGTATACAGA

GGGCTTTGCTGGTACGGGGAAGTTTTTTTTTTGCGGGACATTTTTTGAACAATCAATAGG

GGACAAGTTCCTTTTTTGTCAAAAGTCGCAATGTATTTTGTTTTGGGCCCCCAAGGGGAA

AAAAAAAAA

>770

TTTGGGCCCCCCGGGGGGGGAACCCTTTTGGGGGGGTTCCAATAAAGGGGGGGGGGCCCA

AGGGGGGGGGTTTTCCAATTTTTTTTTGGGGGTCCCCGGGGGGGGAAAATGCCGGGGGGG

GCTTTTGTTTTTTTTCCCCTTTTTTTGGGGGCCCTTTTTGGGTCCCCTTTTTTTTTTTTT

GGGTTTTTCCCCCCCCCCCCAAAAAAAAAAA

>771

GTGGAGGATGGATGGACCAGGCTGCGAACCAACCTGTCAATGCTATTTTGTTTGTGCTTT

TTGTGAAAACAATTTTATGTATCGCATTCCTTGACATTGAATGAGGTCCATGTACTTGTA

TATGGACCTCGTTCAACATTAACATAGAAATTTGTGGTTTCCTTTTAACCACACACGAGA

TGTATTGTGTGGTGTGCCTTAAAAAAAAAAA

>772

AACCAGAGGGAGAGGCGGAGGGAGAGCTTGTGGGTAAGCGTGTGGGCGAAGCTGTGGGGA

AATCGGTGGGGAGGTCAATAGGGGATAAAGTGGGGAAGGAGGAGGTGGAGCTTGGGGGTT

GGGACTCGTCATTTTCAAATGGAATTTCTGTTTCTGTTTCTGTTTCTGTTTCCGCGTCGG

AATGGAAGTACTCGGAGCGGAAAAAAAAAAA

>773

AAGAGAGGGGCCCTTTTTGTTGGCCAAGCTCCATTTTTGGGACCATGTAGACCCTGCGGC

TTCCTTGTTCCCCAAGCAATTTGGGGGCAATTTCCCCCATCCCCCCGCAATTTTAAGTTT

TTTGGATTCCCAAACCATTATTGGGGAACCCTGGCCAAAAAAAAAAA

>774

GCATCCAGGATGGTGGGTAGTGAATGCCAAACTGTCATGTTGGCAATTTTCGTTGTGTTC

AGAGAGGTGTTCAGATTCATGCGAAAATAATGAGTATGTCGGATGTGTATGTAATGGTCC

AAACTGACAAAATATAGCATTAGTTGGCAGCAAACAACGTAGTGTGTAACGATGAGTATT

TCATAAAGTACCTGTACAATAAAAAAAAAAA

>775

TTTTGGATACTCTGCAAATGATGTCGTGCCCTGAAAATATTAGCCAAAATTCACGACACT

TCATACAGGCGAAACGTATGTACCTCTGTGGAAATCAACCAAGGAACAAGCTCACACAAA

CATACAGTTGTATATTCACACGTGTGTTTTAAGGAAAGCAGGGATTCAATTTTCTTAATC

AAATATGCATGTCACCATTCAAAAAAAAAAA

>776

AACTTGACGTGCGATTCTTATATCGGTGCACAGGGTTATACTCAATAACCGTGTATCATG

TCAAAAGACATGTTGCTTGATCCAAGGAGGACGTAGATCTACATGGGTGGCAAGCCTTTG

CCAACCCAAAAATCGCGTATGCGACGCGTTGGGTCATTTACCATATCATCACTCCCAAGC

GTCGACTCGCCTTTTGCCGCAAAAAAAAAAA

>777

GATATTCGAGATCCAAGAGACACGAGCAACGTAAACTATTTACATGGCATTCAAAACAGA

AACTGTCAACTCACCTGCTTTTATCACTAACGGTTCGCAAGGACACACCCAAAAACACAG

GGCGACCGCCCATCAATTTGTTCCCAACATATCGCGGGTCGGAGCAACCCTGTCAAGCAC

AGGGATTTGAAACCTGCCCCAAAAAAAAAAA

>778

AAGACATGAGAGGGTCTGCCAACACAGCTCCCATAGCTGGAAGGGCAAAAGTCACCACCT

GCGGGACACAGCAAAGAATAAACCAAACATGCCAATGACCCTATAAAGTGATGAGACCTC

AGATGAGACCATATGCGATTCAGATGATGCACAGCTCAGCCATGCATGTGATCAGCATGC

AATAGGGCAATGCGAAAGTGAAAAAAAAAAA

>779

TTTCGTGTGGGCCCAAGTGTGTGTGGGGGTTTGATGGTGACCCCCTCCTGGGTGGGGGTA

AGGGGGGCCCCTTTTGCCGCCCGCAAGCCCCCAACATCCCCCGTTTTTTTTTGGGGGTCA

AGTAGGGTCCCCTCGGGCCCCTCTTTGGGGGTTCCTACTATTTTTAAGTAAGGGTTTTTT

AATTTTGTTGCCGGTGTTGGAAAAAAAAAAA

>780

CCCCTTGGATGAGAATGTGCCATGGTATGCGTCCTATATGTACCTTATCTGATGGTGATT

CACAGACTCGTGCGATGCATGTAGACCAATANTGAGGATGTGATTGGTAGTTGTTGGTTC

GTGGTTTGCTGTCAACGTACACATTTCATGAAAATAACTTGGCTTGTTTTAACAGGGTGA

ATGGACGGTGACAAACAACTAAAAAAAAAAA

>781

TTTGACCCAAACCCGAAGTTCGCCTCAGGAAACCAGTGATTTTCGTTGTTTGTGGGGTTT

TTTTGCGCCGTTGCACTTACATGACGGAGCTTGCCCATATTTGTGTGCAGCAGGGGACAT

GTTTCGGGAAACAACAGTGCCAATGATTTTGAACATAACAGCGTCCCGGAAATTCCAGAT

TTCCCTCAATCCTTTAAGAGAAAAAAAAAAA

>782

TAGTAGTCACTCGACATGAGGACATGGGTCAATGTGATAGTACAAGGTAGTAATAGCTGC

CAGTCACTAGGAGGAGATAACCGGCCAAGTGATAGGATTGTGAAAGGAATGAACAATCGG

TCATCAAGACTATAATGGAACATTTAACAACAGCACCGATCGTACAAGCTGCCAGTATGC

AAAGAGAGTAGCGTGATTTTAAAAAAAAAAA

>783

GGAACACCAGGCAAATGCAATCGAAGGAAGCTCACAGGGTGGAAACTGAGAAAGCCTTTA

CACATGGAACTAGGTTTGGTTCGTCCAAGCGTTAGATAATCACCAATAAATGATTTCTCA

GTCGACTAGCGCGTTTAAGAATTCAAGAAGCACTTTGTTTGTTTCATGAGACAACATTAA

TTTGTTATATGATATATATCAAAAAAAAAAA

>784

TACGGAACATTCTTCGCGCTCAGGTGCCTGTCCGTGGTCAGACTGTAGTTGTGGCTCTGC

TTGTAAATGTGGTAGCGGTTGCCACTGTGGCTCAGTCCGTAAATAGAGGAAACACGCCAC

ATACTTGATACTCTTTGTTATCAGTACATCTAGTTTAGTTCGTTTTCAATTTCGTAAAAG

AAGTACATAATCTTCCCATCAAAAAAAAAAA

>785

CCCGCGCGGTTGAAGACCGCTGCAATTTCTGCTTGTGGATCACAGCAATATGCGTGCAGG

ATACAATTCGCGGATTCATCAGACGTCGCGGGTAACAGTCTAGGTTGTGGCTATTGGTGA

TCAAGGTGGAAATCAATTTCCGACAATGAATCTACAAACAAGAACGCCACTGACGCGTTT

CTCGATACACACTGCGCAGTAAAAAAAAAAA

>786

AAAATTCGCATGCATGCCAGCACTGGAACCAGCCCTGGAATCTTCTGGAGATTGCGTAAG

CGTGAAGCTCGTTTAAATAATATGCGATATATATTTGGTCGATTGTGCACTGTAAATGGT

TTTGTCTGCGCTGGGTTCGCGTATGACTGTTACAGAATGAAACTGATAATGTTGAAAAAA

ACAATTCAAGACCGAAACTCAAAAAAAAAAA

>787

AGCGGGCGGTTGCGTTCCTCGATCTCGCGCTCCCATCGCATCGCATTGTATTGCTCAGGC

GTCATCGAGCCGCCCGCTCCGGGGATTCCCCCTGCACCCAGGCGCGCGGCGATGGCGGCG

GGGGAGGGCGTCTCCATGCCCGGCTGCGCCATGGGGGTCATGCCAGGGGTGAAGGAAAAT

AGTGGCACCACCCATCCTGGAAAAAAAAAAA

>788

CCTGGTGGTTCACATCCAGTTCAAACGACGTACCCCAAGACCAGAGTCAAGAACCTTTTT

CCATGATTTTTGGACTAGGTCCTTCATTTCAGGGACATCCATTGTCAACCTTGAAGGGCA

ATTGGTTGAGTGCGCAGCAAGGTGCCTATTGCTCCGATAACGCGCTGCCGTGCTAATGTC

TCGGGAAAGTTTGGTTCCCGAAAAAAAAAAA

>789

ACGAACCATTCGTTTGGCTGTCACACACGGATAAGCGTATATTCATTTGCTTCCGCAAGG

TAGCACCCACACCCCACAAAACTTTTGTTCGCTATGCTAAAATATATCAACGGCTTGTGG

CCGAAGTTGTCCAGCGGACGAATCGCTCTGACAACTAACGTCCCTAAAACCTGCGTCGCG

TAAGATAAAAGAAATTTACTAAAAAAAAAAA

>790

CCCCCTTAGGGGTCCCCCGCGGGGGCCGGGAACTCCTGAAGGGTTTTGGGGACCCGGGGG

GCCACATTTTGGGGGGGGGGGGGGGCAAAAACAAAACTTTTTTTCAGCCCCCCCCCCCGG

GGGGTTTTTTCAAAAAAAAAAA

>791

TGGTTTGTGCGGGGCGTGAAAGGATTGGTCGCGGGATACCTCGTCCAACATGTCTGCATC

TACAGCCGAATCCTCACACCACACTACATCAGAACCCGTGGATCTTAAGTACCGTACGAA

ACCTTCATCAACGGAACCCCATTTATTTGGCATGGCGGTCGTCCGAGAAGCGTATCAAGT

AAGGTGCGCGGTTAGCTACTAAAAAAAAAAA

>792

GTTTATTTGCAGCGAGCATTCCACCACCAATAGTCTCTCATTGGGATCATCCCACACCGA

CCACCACCAGTCACTAAGCCTCCGATAAGATGCATGCGCTGTTTCGTGAGTAGCGTCCCC

GCAGAAAATCGTTGAAATTGTCGGAAATAGAGATATCGTTGTATAAGAATGTTAAAACAT

GCCTACATACATGATGCTTTAAAAAAAAAAA

>793

ACCTCTAACAATTTGGCAGGAAGCCGTGCCAAGCGATCCGAGTACAGAAAATAGGAGCAT

CACTAGATTAGCCAAGGATGCTTGATCGTGGATCTTAGGTTCTTAGGAAATGTATTCCGT

ATCCTTTGAGGACGACAATTTTCGTGCATACATGTGATGCGACAGTATATTGTAAAGGAA

TTCCTCACCTGTGAGCGAGTAAAAAAAAAAA

>794

ATCCCCATCGAGACAGTATGCTGATCTGTGGAAATCCATACGAAACAAACTCTTTGCGTG

TTTCATATGCAGTCGGACGCTAGCATGTCAGCTCTGCTTGCTGGTGCACCACCGAGGATT

TGATCACAGTTGTCATCGAGCATGTCTACTCCAGCATCTTGGGTTGAAAAAAAACAATGG

TCGCATCGTGAGAAACGCATAAAAAAAAAAA

>795

TTCTTCATGCATTCCTCAAAAAATGTATGCTTGTCTGTCGAAGTGAGATGCTTAGAGCGC

AGATGCGAAGGCGATCCCCGCATCCTGGTTTAGCACCGCTAACAGATTCACATCCAAATA

TAGCGTAGCTAATCATCAAGACACTCTGCTTGATCGGTAACAAGCAGAAAAAGTGTTTTC

AAGAAAATCTCTTCGGTCGCAAAAAAAAAAA

>796

CCAAGACTGGATCCGCCTCGTTCACCTTCTGAGATTTCGCGGATACAGCCTCGTCTTCCG

CAAACTGACGGCTCATGCGACCCGACGTCGCGTTCGGCGTGGCATCCCCAAGCACGAAGG

GCTTCAAGGACTGTCTCGAATCCCTCGCCTCAAGATCCGCGCGCATCGCGTCGGGGAGTG

CGCTTTCCTCCCCCGCCCCGAAAAAAAAAAA

>797

TGGGCGGGTTGCTGCCCCCCCTTTTTTTCCTTCTTCTTGCTCGGGTTGGGGGCGGGGTTT

CCCTTTTTCGCCTTGTCTGGGGCCCCCCCGGTATGGAAAAAAAAAAA

>798

TGCCAGCACGACACTAGCTTGCATGCTTGTGAGGATGTTTCATGGACCTTTATGTGGAAT

TTCCAGGGGCTTAGAAATTCGTTGGTGCTTGCTAGCCGCCATTCCCCACAAACGAGTACT

GCTGTGTTGCATATAACCTCGACCTCCGGGTCCCAAGCTCGTGTACATTATTTGTTTGGA

TGAAAAATGTGCTTGCTGTCAAAAAAAAAAA

>799

TGACTCAAGGTTGATTGAGGATCATCTAGGCGAACGTCCACAATCATTTTACGGGGAGTA

TTTCGATATATTTTTGCGAATTGCTAGGCAGCTAGACGAGTTAGCAAACAAAGACTGAAT

GAAAGTTGCTTGATGATGTTGAGCTCAGAACACTACTATGAGCTCCATGGTGAGAGAATC

AGGACTTGCACCCTGGGGGGAAAAAAAAAAA

>800

GTGCTCTTTCGGCAAGAAAAGAAGAAACGTGCCAAGGTTCGCGCTGCCAAAATCGCTGGA

GGAGAGGAGGTGTTTGATGATGACGACGAGGACTGGGAGTAGACCAGTAGTCAAGATTTT

GCTACAGCAACTAAATGGTTTGCCCCATACTGCTAGAGATCTCGGACAGCACGGGAGTTT

AAACAACATAAATTGTTTTCAAAAAAAAAAA

>801

CGGCAATGACCAGCCGGAGGGTATGATGAGACCTGAAGATGGTGGGCCCGTATTGGAGAC

CAAGAGTCCGCATTCTTGGCATTTTTACCAAGATATTTTGATCCCATGATTTGCCAATTG

CCAATTAATTACATATTCCTTGATTTTTCAATTGCCAATTAGTGACATATTGCAATCAGT

GTTACTTATCCTTGTATTGGAAAAAAAAAAA

>802

ACCCCAGCACATGTGTGTTGTTTTGGGATGTCACTTAACAGCAAGCTTTTTTCAGCAACA

CTTTAGTGATGAGCTATACTGTAGCTAGTGACGGTTGAAACTGTTTAGCATAGCCCCTTT

GCTGTCACATCAAAATTTATTTGGCAAAGTGACAGTTCAATTGTTTGAATGACTCGTGCA

GTGGAAAAGCTTGTGTTTCCAAAAAAAAAAA

>803

CCCTCAGCCATGCTTGTGGCTGTTCTGTGGAAAATGATTTGTAGTGTGTGGTAGCATGAC

TGTTTTAGTACAACACTGAGCACAGCTGTTGGGGACCCCAAGCTCAAGCATTGCGTTTTT

CACTGTACAGGCATGCATGTAGAACCCAGTACTGTTCATATATAGTCTAACATTTTGCTA

CTTGAATAGCCTCCGCGACTAAAAAAAAAAA

>804

CAGGACATCACGTTTTTCCCACATACTTTTGCAGATTTGTTGCGGCAATGGTTTGAAATT

ACGAGCACGGACTAAGTACAATACCACTAGTGGCCACTAGGCATGTGTTTCATTGGCAGT

GGTAGTGTAACAGTTCAGTGACTGTGCACCGGATATGAAGTGATTACTTTGTTTCAAAAT

ACATTTAAAGCCATGGCCTTAAAAAAAAAAA

>805

CGGCTGAACATATGTCGCCTTGCGCGAGGCACTACGGTGTAGCACAGTGGATTTAGCTGT

ACTGCGAGAAATGGCGCGAGAAAAACCAGCTCCGCGCAAAGCGGGGACCGTGATCTTCAT

GCTAGTCATGGTGTGGTGGTTTCCCGCAGGGTGGTTGCGGAAGGAATGTTGGGTGTGCAC

AAAGCCCCCTGATTTTTTCCAAAAAAAAAAA

>806

CCCTGATAAGGTGGGCGGGTTTTGGCCCAGAAGAAGATTCTTAGGGCCCCCTCAAGAATT

TCCCCAATCCCCCTGAACCCTTGAAGGAGTTTGAAGATTCTTTGTTTGGGGGGGAAAGAT

TTGTTTGTCACAGAATCCAATCATTGGTTTTTTTGAGAAACCCTCCCGTTTGGGCGGTCG

TGCCAATTGTTTGGTTTCGGAAAAAAAAAAA

>807

CGTCCAGGTCCACCAGGGTGATGCACCCAACCCCAGTTCGTGCCAGCGCCTCCGCCGTCC

ACGAGCCCACCCCACCCACCCCCACCACACACACGTGCGCGGCGGAGAGTGCGCGCATCC

CCGTCGCCGCGTACAGGCGCTGTACGCCTTCGAACCTCCACCGGTCCGCTGGAGCCGGGT

CCTAAATTCCCTCCAAGCCGAAAAAAAAAAA

>808

CTGTTCCCTACAGTTTTCTTTCATTTCCGACACAAGTTCAGCAGCCCTGATGGTGTTCGT

GTCCACGTACTACGAGTACCTAGCTGCGGTTGGTGAACTACGAGAATATAGCATTAACAA

AGTCATGCATCCCGTTTGATTGTATTGATCACTCGTCGTCATTTTCTGAATGATGAAATG

CATAATGAAATTTGTTGTCCAAAAAAAAAAA

>809

ATTGTCCCCATGTCGAGGGGGCAAGGGCCCCGTCAACCCGGAGTCCGCTGCTGCGAGAGA

CACAAGCAAACGGCCGTAAGGGTAACGACCGCGGATGGTCCCAGGACTAAAATGTGAACG

CGTGGGTGCCTTGCATAAATTCGGGGAGGCAATTTGTGCCGGCAAAACCATTGCGACAGG

TTTGAACTGTGTTCCCGGGGAAAAAAAAAAA

>810

TGGGGGCTCCATGTGCCAAGTTGGGCCTTTAAAGGGGGGGGGTGGGGGGCATATCCAAGT

GGTCTTTGTTTTTCTTCCGGGATAGGGAACCCCTTGGGGTTTTGGGATGTTGGGGCCCCC

CGTTCTTTGGGGGCCCAAGGGGGCCCCAAGGGGGGGGTTTTTTTTTTCACCAAAACCTTT

TTTAGGAGGTCCCCCTTCCCAAAAAAAAAAA

>811

TTTGGACCTTCCCAAATACAAAGCCTTTTTTTGGGGGTTTCCTGTTTTTGTCCCCGGTTA

GAATTCGGAGGGGTTGCCCGGGCCAAAAAAAAAAA

>812

GATCAACACAAAACCAGCTACAAATCCTTAGTCTGTTGACCATTGATAGCCACGTCTCTC

TGTTGCTTATTGACTTTAGTTTTCAGGTGTTTCAAGTAGTATAGGTCGTAAAGTGTGGTA

CAGATGAAGGAGTTGGATGTATCTGCTCACATGCCGTGATAATATTAAATATGAGGGGTC

GAGTGGGGAATGGCACCTGCAAAAAAAAAAA

>813

ACCTTCCCCAATCCCCTTTGGGGGTTTTTGGTGTGGGGTCTGGGGGGTGAAATTTTTTGT

CCCCCACAAACGTTTCCCCGTTGGTCCCCTTTTAAAATCCGGGGGGGGGGGGTAATTTTT

GGGTTTTTTTTTTCATATTAAAACCTTTTTTCCCGTGAACCCCTCTCGGGTGGGGGGGTA

ATTTTTCTTCTCCGGGGCCCAAAAAAAAAAA

>814

TGCGAGCGTTGGCATGTAGGCGAGCTGCACGTTGAGGTCCCAGTGCAGGTCTGCCCATGA

CGTGGGAGTGAAGAGCCACGGCCCCGTGTTGTCCAGAACAGGACCATTGGGTCGCATCGC

GCTGCCCAGCTTGTACATCTGGATGTAGTAGAAGCCTTCAAGCCACGTCTCCGGAAAGGA

CACCAGTGATCCGTCTTGTGAAAAAAAAAAA

>815

TTGAAGGTGAACGTTTTGAGAAGGTAGGATGATTCATGTGACAGATTTGCTTTGGAGAGG

ACCACGTGCTTGATCCACTCACTGGCTGTGGTAGATATAAGTTCGTATGGCTATTATCAA

ATATTTTACGGGCCAGGCATCACAGACATGTATACGTATAGACAAAAGTTGCATTTCGTA

AATTTCAAGCGCATAATAGCAAAAAAAAAAA

>816

TATGATCGATGAGCCGAGGTCGCTGCACATGCGCTACAATCGCAAGTATGTGGAGGGTAT

CTCGGCGGATGCTGCACTCTGTAGTGCCATAGGTAAAGTGAAGCGAATCTAGCCGTATTG

TAATTTTGTGAAAACGCAACAAGATCTGGATACTATGAAGTTTGATGACTTTGAAGCTAA

CATCCTGAGGCACTACTTTGAAAAAAAAAAA

>817

TTTACTTTTCCCCACAGCGACGCGCTGGATGCGATGGTTTTTGCTAAGGTGGTTAATGGT

ACGGTTTGAGAACAAACTCCCTGATCATACACATTCATACGAGGGTTGATAGCTAAACCG

GCTGCCCGCCACTGAGTTGACAACACAGAGCGATGCTGCTGTCAGCCCGCAAGCAAACAG

CGAAACATCTCCCTCACCTGAAAAAAAAAAA

>818

TTGGCGCCCAAAGGGGGCTCTCCTCCGCGGCTATCCTCTTCATCAAAAACACTGCTTAGC

ACTTCCCCATCCCAAAAAAAAAAA

>819

GGGCCCCCCCCCTGGGGGGGGGGGGTTTTGGCCCTTTTTCTTTTAAGGGGGGGGTTTTTT

TTCCCCCCCCCCCCGTTTGGGGGGGGGGGGGCCTTTTTTTTGGGGTTTTGGGGCCGGGTT

GGATTTTTTTTTTGGGGCCATCCCTTGGGTTTTTTGGGGGAATTGGGTTTCCCTGGGGGG

GGTTTAAAATCCCCCCCCCCAAAAAAAAAAA

>820

TGAATCAACCAGCAGATGGACCTGTTGCCCACGCTATAACCTGTGTCATAGTACAAAGTT

GTAAAAGGTTATAGTCAACAGGCAAGGAATTTATAACTAGCTGATCATTTGTGATTAGAA

TGGACAACTGAGTACTTGTGTGGATCATGTAGACCAGAACAAATGACATGATCACCAGTG

TGAAAGGCATATACTTTTCCAAAAAAAAAAA

>821

AGGGGACATCAATGTTGAAAAATGTTTTGAGCGAGGGACATGTGCGTCCTTGTGGTTGAC

ATGTGTTACATGGCCATGTAGGGAATGTTTTGGGAGATTATGTTGGAACAGCAAACTCTC

GAGTTGACATCACACTCTACGGGACAGGTAATGTCGCAAAATGTTTTAGGGCGGGGGATT

GCAGTACTCTCGGCGTCTCCAAAAAAAAAAA

>822

CCCAGTGGAGGATGGATGGATCAGGCCGCGAACCAACCTGCCGATGTCTTGTTGCTTGTG

TTTTGTGTGACAACAATGCTTTGTACCATGTTCATTGAAATTGAATGAGGTCCACGTACC

TGCGTGTGGACAGTATTGAACATTTACATGGAAATTGGTGGTTTTGTTTGAACCATACAC

TTGTATTACGTGTTATGTGCAAAAAAAAAAA

>823

ACGAAAATGACGATGTACACATTCGAGGAGAGGACCACGATTTGATCAGAAGGCTCAAGT

GGCTGGAAATCGTGAGGGACTCGACTAAGGTCTGCAAGGATGAGGCCAGGAGGTGCATGA

TGGGGCTCGGGGAGTTGATATCCACCACTGCCTCTGCCATTAGCAGGAGCACCTACAGCA

ATTGTTCTCGCATAAATGGTAAAAAAAAAAA

>824

CCCCGCCCCCCCGGGGGGCCTTCCCCCCCCCCCGCGGGGCGGGCTGGGGGCCATGTTTTC

CCCCCTTTTGGGGGCCCCCGGGTTTTTTTTTTTGGCCCCCCCCGGCCCTTTGGTTTTTTC

CCCCCCCCCAAAAAAAAAAA

>825

CCCCCTTGCTTGTGAGGGGGGAACAGTTCAAAGGCCCCGGGCTTTCTCGGTAGGTTTTGG

GGGGCATAATTTTTCATCTTGTTACTATGTTTCAGTTTTTCTGTAAAAAAAAAAA

>826

GGAGGAGATATCAAAATGCGCATGTGCTCCCAGTCGGGGTCACCTTTGGGAGTTGGGCGG

CCCCAAGTGTAAAAAAAAAAA

>827

ACAGCCCAAGGATCCACAACACTGCCCCGACGAATAACGTAGCGAGTCCCGCGGAGATTA

TCGCTCCCCAGGAGGCAGGCTCGATATCCGAGTCCGAGTCTGACATCTTGGAAGAAAAAA

TCCGTCACAGGTTGCTTTCGCGGTTGTACACGCCTGACTTGCAGCACAACAGCGAAGAAC

CGGCCGTGTTAGCAGGAGTCAAAAAAAAAAA

>828

TGGAAAAGTTCGATTCAGTCCTTCACTTACTTTGGATGACTGTTTACCCATAAGGAAGGG

TTCACGTCTCGGGACATGCCCTCTACATGACGAACGCCCTGACCAAATACAGCAGAATTT

GGTAGCCTCTTCAACTCCCTATCAAAACCAGTGGACCAATCGAGCATGTTGATAACTGAA

GATGTTCCTGTAGACCCTCCAAAAAAAAAAA

>829

GTACAGGCACCCATGGTCAGCGGTACTGGCATATTCAGAACCATTCAGCTGGTTTGGTGG

AGGTTTCCGTTTCCAGGGTTGGCAGGGGGGTGGGGCTGGTAGCTATCCCCAGGACTTGGC

AGGTGGGCCCAATCACTTTTTGGACCAATCTTCCAATTATTCCACCAGGATTTGGTGGTA

TTCCTAGCATTGGTGCATTCAAAAAAAAAAA

>830

GGGCTCGGCCCATTTGAAGATACAAATGNCATTGGACATATTTGTTNGATTTAATATGCG

TATGCGATTNTGCGCCATNNCCAAAAAAAAAAAAAAAGTTTTTCAGGGGGCTGTATCATT

TGGTGCGGTGAGAAGGTTTAACAGACAACCTTTGTAAAGCTAGATTTACAACTTGAATTT

TATTTTTTCTTACGCATGTCAAAAAAAAAAA

>831

AAACCGCAAGGGTAAGGTACACGCGACTCCGTTCCTGGTCTTTCCTTTCCCCGAACCTTT

TCGCAGCTACGTTGATACCACCATATAATGATGAAACATAAATTTGATTCCAATTCGCGT

TTGGATCCAACTGCCACGATTATCGGGAGGGCACCGCCCGCCCGTACACTGTACACTGCC

ACTGTAGATAGTAGGAGGCGAAAAAAAAAAA

>832

CTGAACAACGTTGAATCAAGGGGAGCGCACGCTGGATACACCACGGTGACCAGGAAGCTG

AGACCGCCGAACACAGCGGACAACATCTCGAAACGGAAATTCTGATGCTGTCTGAGGATT

TGCAGCTCGACCCTTCCGGCTCACCGTTGCAGCCAAGAGGAAAGTTGTCGCGTTGCAGGT

CTCGCTCGTCGCCTGCCCTGAAAAAAAAAAA

>833

AAGTTTGCAGGCGGCCGTACGCACTCATGACCGTGTCCGGGCGGTTTGCCATGGGCGTGG

GCTCTCGAGCTGCGGGAGAAGTGGAGCGCTGCAGTTTTGCAACAAAGTGTTCGTGCAGGC

GAACGCGGCCTTTGCGCCTGTCCGCGGGCTTCGGGCCCCTGCAGGTTCGCTTGATTGAAT

GCCCCTCAGCAGAGGCATCGAAAAAAAAAAA

>834

GCCTCTCTTGACCTTGAAACTGCTTGAGCTTGTTCCAATAAACATAGGACGTGACCCTTA

CCGGCTGGGCGGGGGGGCACATCAAGTGATCACACGTTGAATTCCATAACTTCGCGATAT

CACTCCAAGGTACAACGCACCAACCCTCCCACATATTTTCAGATGCAAAACGTAGATCCT

GTAAAATACGACCCAAAGTTAAAAAAAAAAA

>835

GGGCCCGTCCCAGATGCAAATGCCATTGGCCATGGTCGTTTGATTTAGTATGAGTATGCG

TTTGTGCGTCTTCCCCAAAAAAAAAAA

>836

CAAGGATGTCTTAACGCGAGAGCAGGCCGGCCGAGATTCGGGCCGCGTTGCACGTGCTGC

CGCCCCCCATGCCGGCCGCGGCCACTCCATTCCCCTTTTGCGGCGGGTTCCTTTTTTTGG

GATTTTTTCGTTTTAGGTGCTCTTTCCTAGCTACCGACGTAAACAACTGGAATTACGTCT

TACAGCGGGAAAGAGCNCCTAAAAAAAAAAA

>837

GTCCGCCACTGTCAGTTTTGCTGACCTAGAAAAGGAATTCGGGGAAGGTCATTTGGGGGA

GTAGATGAAGTTGCAATTAATGAAGCTTTGTGGTGATCAGCTTGTATTTTTGTGTGTTTA

GTCACCATATTCTCGGGACTACCAGGCCGTGCGCGATGATGTTTGAATCGTATAAACATC

CCCTTTGAATGCTGGTTGTGAAAAAAAAAAA

>838

AACTGCTTGAGAAATAAGCGTATGTCTGAATTCAGAAAAATAAATCGGAAACATTGGGAC

TTTTTGCGAAGCACAATCCCGCCATCAAGAAAACAATGAAGTACTTACGATAACAAACAT

GCGCAGCCACCCCGAAATGCGGACATCCCCTATGAAATCATATGCCAGGGTGCGCTTCGA

GTAAAACAACAATCTCAACTAAAAAAAAAAA

>839

CGCAGTTACTTCAACTGTATGTGATGCAGAAGTGGACTGGCTCGCATGAGCTATGCTGAG

ACGATGCTCGAATTCTCTTAATCGATCCGTTTCCATAAGTTCCTTTGCGCTTGCCACATG

CATGGTTGCGTACGGTTGACGCAGCGAGGGAGCAGGTAGCAACGTGATGCCGCAAGGCTG

GTCCGAAGCCGTAAACGAGCAAAAAAAAAAA

>840

CCGCTGCTTGACCAATGCCAACCCTTGTCTTGTTGCCTTTGCGCAGGCAGTCCGCGATGT

TGCTCGACTCCGGCCTGGTGTAGATTGGAATGTCAGTTGAGCCCCTATTGATACTCCTGA

TTTGGCTCAGTCAGGTCAGCTAGACGGCTTGGACACGTACATTATGTGTACATGCTACGT

GGTGGTACACGGAGGGTTCCAAAAAAAAAAA

>841

CCTAGGGAGCGCGACCGGCGAGCGAGAGGAAACGGCGTATGTACAGGGCGATTTCGCCGA

CGCATTTCACGATCTACGTCATCCCAAGTTGGGGTCGATATTATTCGTCGGATACATTTG

AAGGAATCAGAAAAAATGAGAGCGCCACCAATTTGTTCGCCATGCAACGGCTGTCCAACT

CACGCGTGAGGTGAGTAGGCAAAAAAAAAAA

>842

GTTGAATCCATTTATGATATACCTTCCCCAGAGATCTGGGGAAGCAACAACAGCATAACG

ATCATGGTTGGGAAACAGTTCATAGCACAATGTGTTCCACTGGTTTTCGGATGTGCACCG

GCCACTTAGGGCATTGGACGTCCAAGTCCAGGTCCAGGTTTACGTACTGAATGAAAACCA

TGTTTTTGGCACTTTGACCCAAAAAAAAAAA

>843

GAGGATGGTGAGCAGGAACAAAAGGAAACATGATTCGACCCAAGAGGTGAACATAAAAAA

CACCACTCTGCTTCCCTACATTTGAATCGGGCCCAAGGTCTTCAAAATGACGCTATGATG

CTGCTTAGTTGAATATTACCATCCGCTGTTTTGTATGTTTGAGACGGTTGCTATCGTGAC

AAAAACATGTCTCGTTGCTCAAAAAAAAAAA

>844

CCAATGGGGCATGGAAATCCATGCGGATTACGGTTTGGAATTCGTTTTGTCGGCTTACGG

ATTGGATTTCGCTCAAAAAAAAAAA

>845

TATACTCATTAGGCGACAATAGCGAGGTAGAAGTCTCATCTAGGTTCAGGCGAGCAGGCT

CTGGGAGGAAGTGCTAGAACTAGTGCAACTGCATTGACATGGGTGTCGGGGCACCAACCA

AGCTCTCTATTTATGGAAACAGTGCCATATCGTAACGCATCGAGCAGCATGCATGGATAC

AATTTTACTTCGTTATCGTCAAAAAAAAAAA

>846

TTAGAATATGTGACAACAATCTCCAAATTAGGTGTTCGCACAGGGATCTCTTCACATCGC

TTCCAATTTTGACTTATGAAATGACTTGCGTTGTAGATGTTTCTCAAAAAAAAAAAAAGT

ACACTCTAGTGCAAGCCTTTCCTGTGGAGTATGCTTGCATCTTGATATTCAGAATGTATG

TAGCACTTATCCTCAGCATGAAAAAAAAAAA

>847

TTTTTTGGGGGGGGGGGTCCTTTCCGATTTTTCGAACCTTCCCGGTTGTCAAGGGGTGTT

TTGCCGACCCTCGAAATGAAAAGGGGGGGGGAATGGGGAACGTTTGGCTTTTCCCCCCGT

TTTTTGTTGGAAGGGGCCCTTGACGTTTGTTCCCCAACGCCCAGGGGGTTGCCAACGGTC

CCATTCGAAACCGGGGTTGGAAAAAAAAAAA

>848

TTTCCCATTACTTTTCACCCGGTCCCAAAAAGCTGTGGTATTGCGGGGGGGCCATTAGTA

GTGGAGGTTTTCTGGGGGGGTTGGGAATTTTAAGGGCCCCATCCCTTCTCCCGGAGGATT

TTTGGGTTTCAGGGGTCCTAAGTTTTGGGACAAAAACATGTTGTCCAATTTTTTGGGGGT

TCAAAAAAAAAAA

>849

GTTGTTCGGTTAGGATAGAGCACTGCCTTTAACCTACGAACGCCTTTGCCCCGCAAGCCG

ATTGCAACAAAGGTCACGGCGTGCACGTTTCGGAAAGAGCGATGCGACTAGAAACCGGAA

CCGATCGGGCGTCAGGGGTCGGGTCATTCCGGTACCAGTTGATGATCATGAGAGCTGGTA

AACGAGACACCGTTTCCCTCAAAAAAAAAAA

>850

AGATGATCCCTCCCACCATGAAGAAGGGTTGCGAAGCGTTTCTCGACCGCTACGGGGAAG

GCATTGAAGAAGTGATGTACAAAGGCGGGAAGGAGTCAGAGAGCAAGGTGTGCGAGAAAG

CTTGTGAGGGGGTGAACTGGAGCAAGGAGGACAAGCCCACGGGCGGACCACCCCCTGGAG

CGGCGCCACCCAAGAAGGACAAAAAAAAAAA

>851

GTGACTCCATATGATGTGTGTAATCCACATCGTAGAGCTGAGTATCAGATTCGTCTCCAT

GGTATCTAACAACACGGGTGTGTATGTATAAATCTACACATAGAAATTTTTTGTTGGTAT

CAATGATTTTCGGAATATTCACAACCTTGCATGCATGATCAAAACTGTGAGGGAGCATTG

AATACTAGTTCATGCGCCTCAAAAAAAAAAA

>852

AAGGCGGCCAATGATTTCAAGTCCCAATTGCAATATTTGTTGGTCAAGATAGAAACAAAG

TTGTCTCATCCATCTGCTGAACGCTGCAATTTGTTCTAGAGAGATATCTCCATACCAAAT

GGATAGGTCAACATATTGCACCAGTGACAATCCAAGATTTCCTGTAAAATCTATGAATGT

TCTCAAAAATTTGTACATCCAAAAAAAAAAA

>853

AAAACAAAAATGATTTAAATTGGCACGAAATTAATTGGTTTGGGCAAGGAAATGTAGGGA

AGAGGTGGAGCATGAACGTGCGTCGGTGCTGCCATTTCACATGATGCCCGCATGTGGATT

GTAGCAAGGTTCAATTCAAACACAGTTTAGCATTGGAGAATTTTGATAAATTTGGAGTGT

TTTGCACAAGCATTTCCGCCAAAAAAAAAAA

>854

TCTGCTGCAAAAAGGTGTTCCGTCGAAAGCATGAATCCGAGACCCACGAAGAAACTCCCT

CCCAGTTCAACCAGGCAGTTGCCAGCCTGACGGTTTGTCACACAACAAAAAGAAGGTCAA

AGGCTGCTTCGTTGCATTCTCCAGAAGCTCTGTTCGGGCGTCGATTGCCAAGCTCTCGTT

GTCTCGAGCGTCGTCATCCCAAAAAAAAAAA

>855

GAATGCATCTAAGCACAACCCACATACAACCAGCCTGCAGAACCCCTGGCGGTCAGCGTA

TGATTGAATCGAGAATGCAGTCGCGATTGGGCGCTATTGCAGACCCATACAGAAAGGAAA

TCGTATGCAAACACATGTATCAGATTCTTCAAATCTGTCCAGTGCATACAGTGAGAGCCC

AGACAATAGGGGAAATAGGGAAAAAAAAAAA

>856

GGGGGGTTCCATTGGGGGGGGGGGGAAGTTTTAGGGGCCACCCTTTTTAAAGGTGGTTTT

CCACCTGGAATTTTTTTTTTTCTTCCCCAAAAAAAAAAA

>857

CTATGATAAAAAACGAAGATTTTCACCGAACGTTATGATCCATCTGCAAGTACTACTCAC

CTATCACTATTCTTATCTTTCTCCCTTTCGTCTGTACATCAACGACAACGCAGCCTTGAA

GAGAAGGCTTAAGCATGCATGCGATGTCTAGTAGCAACCAGCAATTGTGATTCGGCAAGC

GCTATTTCTGAGTGGAAATGAAAAAAAAAAA

>858

AGGATTACGGATTAGAATTCGCTTTGTTAGTTTGTTTAGCCAGTCTGGAGGGGAATTCGT

TCTGCCAGTCTGTTTGGGCAAAAAAAAAAA

>859

TCCATCGAGCTAATCAGGACATATATGGATCGGAACTGATGTGCAATTGGTAGCAGAAAG

ACAGCTTTGAGGAGTTGTCCTGTGCAAGAGATGCGTAGTGTATATGGAGCTCCTATTGAG

TGCAACAAGTGGTGGCTGGGTGCTTANATGAAATGGTCATGGTTTGATATTGACAAAATG

CCATCGAAATTTCCGCTTTTAAAAAAAAAAA

>860

CTAGCGCTTTGGTTTGAATTTTTTGAAGGGGGCCGGAAACTGTTTTTCCCCCTGTTTGGC

CTACCCCTCCCAGGGGGGTTTTTTGCTTCCGGCCAATTAAATATTTTTGCCTCGCTCCCA

AATTTTGGGGGGGGGGAAAAAAAAAAA

>861

CTGTGATACCGATTGGGCTTACACTTCGTAAGCGCGTTCCTGTGGGCGTACTGATAGGCC

CGTTGTCAAGCGGCGTCAGGAAGGGGCCGAGGGTATTGCAGTCGTGCAGCAAGACCTGCC

CATGCGCTGTGCCTGTGATGTGGGTCTAGGGTTCATGATGTAGGGTTGCATGTGTTTATG

CGCTTAGGAATTGTTTTGTCAAAAAAAAAAA

>862

TCCACCAATCACGTCCAGTGGGGAGTTTCGGAACTTTTCCACAAACGCCTCCGCAAGCTC

CTTCTCCACTCCGACTTGCACCTGCAAACAGACGGGCTCAAAAAGCATGCACTAGTATGC

TGTTGCTCTATGCAATACCACTCGCAATTGGTCCGCAGGAGCACGTGTAAAAAAACCTCA

AGTCCAGACAATATGCCGCGAAAAAAAAAAA

>863

CCGATGAAATGGTGATGCTACTGTCGCCCTTTACTGAATGTCAAGGCACGGGTGGCTGCA

CGCCCAGCATGGATTTACGAGTGACATGCATGGATAAGCTGGCCCGCCAATAACAATGCA

CCGTAAGGATTACATTATCTCGTTGCTCGACTAGATGTGAACTCTGTATTCACATATCCA

CAATGCTAGTTCTCTTGAGTAAAAAAAAAAA

>864

GGCTCGTTTTGGTGTGTTCGGAGCGCCAACATCCCATCACGGATCCATTCCATGATTCAC

GACTGGCAGTTCTCCCAGCTTCCGTTGACAAGGGGCTTATTGCAAATGATGGTGTTTTGC

GAACTCATGGCCTTGACGCCGTCATAAGTTAATAGTAAGATGACCCAGAATCTGTGTAAT

TTTAATACTCGAGCTGCTGCAAAAAAAAAAA

>865

CCAAAGGGAAGGATTCATTTCCCCAATTAAAGGTTGAATATTTTTGGTGCCATGCGGGAA

ATTTGGGGGAAACTGTGCAACGATTTTTTTTTTTCTTGATGCAGATGGCCCCTGCTTTGG

AACAAGGGTGTTGAAAGTGACACCCCCCAATGTACTGATTTAGTTTTTGTTTAAGAAAAG

TTGGCCAGTAACACCGCGCCAAAAAAAAAAA

>866

TAACGTTGACGTGTTACTTAATCATTCTTGCTTCCCCAAGGACAATGTCAAAATGCAATG

ATGATACGATCAAAAACCTTATATCAACAGGTCATTTCAAAAAAATCACTGGCATGGGCA

CTAAAGCGCACCACAAGGGGATCCTCAAAAAGACCAAGCTGCCTTGGCATGCTGTAGCAA

ATCTTGAAGGTATTCTTCGTAAAAAAAAAAA

>867

TGGTATCACGATCGCGCACACGTACTTGAGCCACAAGGTACCATTGGAACGCAGCAAATG

CAGAGCCCAAGATAACCGCAAATACGATGATGGCTGAAGCAGCACCTTCAGTAAGGAAGG

TCTCAACAGCCATGGTTGGAGATTTGCAATGAGGTAGGCGGCGGGCCGTACACCGCGTTT

GAAGGTCCTCGCGGCCCGGGAAAAAAAAAAA

>868

TAGCATCAAGTTCATAATCAACACACACTGTTCATGGTGTGCTTGCTGTGGGTGCGATGC

AGATACTTACTCCCTTTCGTACGCGTGTGAAAGTTGTTGGCTATTGTTCAAGCGCAACAG

CGCTTAGCCCCATCGAGAGCGGTTTCCTCATATAGGATCATAGCCATGGCCACAAACAGC

TACTGTTGAAAAAATACATGAAAAAAAAAAA

>869

GACAAATCGTGCCCGTGTTGATCAGTTCATTGTAGAATCGTTACACGATCTTGGAGCTGG

GTACGAAAATATCGAGCACGATTTTTTCGACATATTTTTGATGTTCATGCCTATACAGAG

GGATTGTGCCAAATTGTTGTTGGGACTGAGCGTCTCCCAAATGGGTGGTAGTTGACAAGA

TTATTTTAAAGGCGATCAGCAAAAAAAAAAA

>870

TAAAATCGAAATGCTTTTAGAATCTCAAGAGTCTTTAGAGTGATTGACCTTTACAAATTA

TTAGACACCTGCAGGGCATGCAAATATCCGCATGGGACGGGTTTTGTATGAAGCCGGAGC

CTGTCAAGGCAGGGAGCAGGCGCAGACTTTATTGGCAATATGTTCCCAGTTGTTTTGCAT

TTATATCGAGATGTTGCTGCAAAAAAAAAAA

>871

ATGCAGGTGAGAGAAACCAATCGTGGCGCTGTAGTCATGAGTGTCGACAGGACGCGAAGG

CTCAACCTCGATATGTGTTTCAAGTCTTGTGTGAATTCGTCGCAAGGGTAAGGTTGCGCA

AACAACTGGCTTGGCACACTTAACGCGCGTGCTGTATCTGTACGACCCAGCAGCTTGCTT

GGTCCGTTGCGCCACTACACAAAAAAAAAAA

>872

GGAACTCGTTACCCGGGTTGGATCCGGGCAGGGGGGGATTTCCCTCCCCCTTGGATTTTT

CAATTGCCCCCCCGGGTTGGGGGGTGGCCCTCGGCTAGGGGGGGTTTTTCCCACCGGGTT

AAACAATCCCCAGGAATTTGGGGGGTTCAAACGTTTTTGTTTTTCCCTGGGGGGGTTTGG

GCAAATTGGGGGGCCTTTTCAAAAAAAAAAA

>873

CAAGGAGGCAAATCTCCATTGGAATAATCTGCTGATCTGGGCACATCCATATGAAACAAA

CACAATGTGTGATTCATATGCTGCCGTGTGCCGGCGCACAAGCTCTGCTCCTTGATGCAC

GTCCATGGATTTGTTCACATGTGTTAAAAAGTCTGCTTGTAGGATCTTAGCTTTCGGAAC

AACCTCCAAAAACACAAATCAAAAAAAAAAA

>874

GTTTTTGCGGGGCACCCCTCATTCCTGGAAGATTTGGGTTTTCCTGGGGCCCCGGAAGGG

GTTGTTTTGACTTTTGAAACAGATAGCCTTTGTCCCGTTTTTTTTGTGGAGGGTCCAGGG

TTTCCCTCCCCCCCGGAAAGGGGTGCCCATTAAAAGGGCGGGTTGGCGGATCCTTTGGGA

CAATTTCCAGGTGGTTTGGCAAAAAAAAAAA

>875

TACTGAGAACACGCTCTTCGTCGAGGACGCATCTCCGGACATGAAGTTGAGTGGACGCTG

TAACGTCGACCAGATTGCGGTGCACAACCGTGCTCTCTGCGAGGAGGACATCGCGGCCCA

GAACGATTGGGGCAACTACTTTCGAAACATATTCGTGAAAAAGTCGATGGCAGACAATGC

GCTTTAGTTGCTTACATTTGAAAAAAAAAAA

>876

TCAGCTCTCTCCTCTATGTCTTCCTCCTCCACAAAGGGCTGATATTTTGAGAGTCCATGG

TCTGCCGCCTGCAGGCGCAGCTGCTCCTCTAGAATGCTCATCACCCGCAAGTGCGGGAAT

CTTGTTTTCAGGAGGTGCGTGCGATCGTTGCCACTGAATCCGCCATATAGAACACGCGCG

GGAAGGGTTCGTATGATCGCAAAAAAAAAAA

>877

TGTAATTCCATTAGATACCCTGTCATAGGTCAGCACTGTTCCAACACCCCTTGCACATGC

CAATTGAACGATTGCACTGCACTGCAAGACGTTGGTAGAGTGGGGCATACTAGCCACACG

CGCCACGCGCTCTTGGTATACAAGTGATGAGAACGCCGGGACAGTGCCTATTCGTGTCAC

CCTGGTGCTAATTCGTGTTGAAAAAAAAAAA

>878

GGTCATGCCCCTTGGTGTTCCAAAATCATGCGTGCCACTTTCCCTACCGGGACTTTGGCA

GCAGATCCTGTGAGGCGGACCATACAGGATCCATGCTTTGAATTTGCACGAGAGTGGTAT

ACCATTTCCGATTCACCTTAGGGTGACATTGTGAGATGGCGGATTTCATATACGGGATAT

GTTTATGTAGCCACCCGTCCAAAAAAAAAAA

>879

TGATCCAAGGAGAACGGATCTCGCCATCCTTATCAAGACATTCTGCTGATCCGCGTCAAT

CCATATGCGCGTGTTTCATATGCAGATAGTTGCTGGCACATGAGGTCTGCTGGTTGGTGC

TGAGGATTTGTTCACAGGTGTCATCAAGCATGTTTACGCCAGCCTCTTGGAACAAATGGT

GGGAAAACTTTAACCAACTCAAAAAAAAAAA

>880

GGAGATAGAGATGTGCGGTGTGGTGAGCGTCCGTTGTTGACGTTTTGTCGTGTCGACACT

CGGTAGTTTGTTGACGACCCCAATGAGCGACTCTTCTTTAAGTGACTGCCACCTGCGATG

TCGTCTTTAAGCCCGTTGATCCACAGGCCACGATGTACCGGAAGGCCAAGAAAGTTTACC

CACTGGATCCCGCGTCCTGCAAAAAAAAAAA

>881

CGCTCGCTGCGCGCATCGGCCGCGTCCCCGAGCTCCGCCACCTGGCTCGAGGCTGTCCAG

CCTCCACCTCAAGCTTCCTCCGGCCCAGCCTCGAGCCAGGATGCTAGCTCGTCCTCTGCT

CATGGCGCAGGGTGCGTACCCGTAATCTAGCGTGCTTTTTGCAGCAGGGAACATTTCATG

TTTGACAATGGCGTTCTCGTAAAAAAAAAAA

>882

GGGCCCCTTGGGGGTTTGGGGTTTTTTAAAAACCCCCGCGGTAATTTTTTTCGGGCCCTT

ATTCCCTTCCGCCTTCCCCGTTTTTGGGGCCCCGGTTTTTTTTTAAATTTTTTCCCCCTT

TTTTGTTTTTTTGGGGGGGGGGGGGGGGGCCCCCCTTTTTTGGAAGCCCCCTTAATTTTT

GGGGGGTTTTTTTCCCCCCCAAAAAAAAAAA

>883

TGTCACCACAGCGTCGGGATGTCATGAAGGATACGAGGGAATTGTCGCACCTGAATTCCT

TGCAGCCGTATGGCATGGTACAAAAATACACAACAGTATCCCAGTGCATAGTGTCCACCC

ATCAGGACAATTCTGTTTTTATGCTGCCTTACTTTTTGGGGGTGTTTTACCAGTAAAAAA

GGATGGAGTTGGTTTTTTCCAAAAAAAAAAA

>884

TCCTTGGTGATGTATATGCTGTGATAACAGGCTCCAAAAATGTTCACCTTGCTAGGAGAA

GCATGTAAGTGAGAAGTTGTAATTACTTGCAGTTTGGGCCTTGTTTTCCCAATCGAGACT

GTGGTCTCACGACAGCTGAGGGTGTTTATGGTGTAACTTGGATACTTGGTGTCTAAATGT

CTTAGCACATCGTTTCAATCAAAAAAAAAAA

>885

GTGTCATAGTACTAAGTAGTAACAGCTACTAGTCACTAGGTGAAGATAATCAGCCAAGTG

ATTCGATTGTGAATAGATAGCACAATCATACATCAAGGCTAGAATGGAACATTTATCACT

AGCACTGATTGTACAAGCTGCCAGTATAAAAGGCGTAGCGTAATATTTGTTATCACTTGA

GCCTCTAAAAAAATGCTTGTAAAAAAAAAAA

>886

CAAAATTGCCCCCCGTTGCCTTTGCCTTGAAATTCAAGCCAGCGATCGGGTCCGTAATAC

CTTTGCCCTGAGTGCATAATTTATTCCCCCTGTTGCAGTACGCTTGGAAGAGTTCAAAAA

AACTTGGGGTTACGATTGCCTAAATCTATATACGCAGTTTGCTTCGGTGGGGTTGTGCCC

GCAGCAACCTAACGTGTTGGAAAAAAAAAAA

>887

GCACTGCAACACATGCCTCGCGCTCAGCGAACACGAGCGGAAACCAAAATCATTGGTGTC

TGCTTGAACTTGCAGTGTTCAACGAAAACGCCGTAGGCACTTGGGCAAAAGTGCTACGCC

ACAGGTTGAGGTCAACCCAATCTAGTCAATCTAGTCAATCTAGTTCAGTTCGCCGAGCAG

CGCGGAGATGCGTACCGGGGAAAAAAAAAAA

>888

GTCGAATACCGGAGATGCAATTGAGAAACAAGACACAGAAGTGTGAGGAGAGACTTCCTC

GGATTGTAGGTTGGGGCACTGCTCTCATCACAGTGATCATGGCACCTGTCATAAGGTGAT

GTTAATCATGCCCTGAGATCAGGAAGCTAAGACAACATCGAAAACAACCACAATCAGTTA

CATGACGAAAGAAAGAAATTAAAAAAAAAAA

>889

CAGTGTCATAATTAAGTGTGGAGTGCGTGATGTAGCAGCAGCTGTGCAAGGTGGGAACCA

GCATGCCATTCATCAGCTGCGATTGTTACTAAGAGGAACATAAGATGTTGCATCAAATTA

TCAAAATAGTATCGCTGTCTAGTCTGTGCAAAGTGCAACAGTAGAGCATATCATAATTGT

CATAAAGATTAGCCCAGAATAAAAAAAAAAA

>890

CTGAAGAGTTGCTGACTCTCCCATGTAAACGTATCCCTTGGCAAGCAAGTGGATTGTAGA

GCATGTTTTAGTTTGTATATAGGATGACGATTCGCCACATTCTTAAATCGAGCAACTACA

GGCTCGGCCATACCAGATAACTCTGTTGTTAGAACTTATGGCTCGAAACTCCTATATTCG

TAGATTCTTTCCTTTGTGTTAAAAAAAAAAA

>891

ATTGCATACTTGCTCCCAAATGATAGTGGGGACAAGGACCCCGTTGCGTCCCACTGTATG

TTGTCAATAAACAATTGACTCAAAAACCCCGGCTTCCTACACAGGTTATTTATCAGCAAT

ACATGATTGTTGCATATTAATACGCACATGCTGACATTCAAAAGCTTAACAGAAAGCATA

CTGCAAGCACATACCTGGGGAAAAAAAAAAA

>892

GGGGTCAAGTAACGGAGGTTTATTTTAAGACCGTCAGACTGGCCCGGGTCAATGTGGGGG

GGGGGCAAATTATAGACATGGGTATTTTCGATGGAATAGGTAGCCCATATGAAACCCCGA

CTCCGGTGGTACTTTTGCACACACAGCAGGGCTTGACTGCAGAAATAGCCCGTTATTTCC

CGGGGGGGTTTGGTTTTTGGAAAAAAAAAAA

>893

CAAGAGAGTGAAGCCGGTTAAGCGAACCTCACTTCCTCCCGCATACCGGCGAGAGCACAT

AGAGATGTACGGGCTCATGAAATGACCATGTGTTTGTGTGATTTGGGCGTCAAATACAGC

CTAGTTAGTACCTTGTTGAAGAAATGGTCGTGATTTAATTATGCAATGACCAATTGATTG

GACAAACGCCTTGAAGCCTTAAAAAAAAAAA

>894

AACTGAACATACATTTGCTAGGAAAGGTCTAGAAGATGGGGAACCACGTGGTCTCAACTG

TTCTGCTAGTCCTTGATCCTGTCTTGTTCGTATTTGGGGACAGCCAGTGGTAGTGGTGTA

TTTTGCACCTGTTTGGATGCCAAATAGTAGATCTTATGAACAATTCAAGGTTCTCAACAA

ATGTGCTTACCGTTGTTGCCAAAAAAAAAAA

>895

GAATAGATCCTCGCATTTTGGAGCAATCCCCGCGGGATGCAAGCTGGGCAGCATCTCTAT

AGACACTTGAGCCGTGTCTCCAGATCGGGTCTCTGCCATGATCTGCAGCGTCTCTGCAGC

ACGACGAGCGCCGCTGGATACCAACACCTGTTACAGAACAAGCAACAGCTGCAATTCTAG

GTTCAGAGAGTCTGTACTGGAAAAAAAAAAA

>896

AACAGCTACTGCTCGCTAGGAGAATAGAACGCACCAAGCGATCAGTTGCCAGCATACTGG

ACAACCAGGTACACAGCTTACGACAACTACGCGTGTTGTACCGACACCGGGCATGCAGTC

GCCAGTACACAACTGATCATATCATCACGCCGCCGTCACCCGCCCGAGATTCCGCTTCGT

CCAACCCCGAACTCATTTTGAAAAAAAAAAA

>897

GCGTAAGTAAGAGCTAGGCGGAAATGAAGGGGGAGCTCAGCGGAAATTGCGGGTTGGATA

TGGTCATCAAGCTGACATCTTTAGCAATAGATGATGTATGTCTGATTGGGCTCTTGGTAC

TGCGCATAGCTTTTTATGGGATTTTCGGCCGATGATAGCACCATGTCTGCAATCAACGTT

TTGTAATGGTTGTGTCCTGGAAAAAAAAAAA

>898

ACGAGACAGCTTATCAAGTATAACTGCTCAGCAAAAAAAAAAA

>899

GTCAAATCAATATCAGATTCCTTTGGCTCGGCGTCATCCCCATTGTCAGAGATTCCTTGT

GGTGCGCCTACAACCGCTGCATTTTGCCCCATGAATCCATCACAAACACTCCTGGCCTCC

CACTGCGGTGGCTTGCTTGGCCATTTCGATAGCTCGCTCTGTCTCGTCATCCCCTTTTTT

TCGCTTCGCATTTTTTTGCGAAAAAAAAAAA

>900

TAGGGGTGACAAGGGGCTGAAGGGGCATGTCTTGCACTGAATTTCTTGAGTGAGGGGGAG

GTAAGTTTTGAAAACTTGTCATGATGTGCCGCTTCTACCCGCGCTTGCCGCCCATTGATT

CGTCTTAGAATTATGTACGATGAAAAAAATTCACATGAATTCCAAAAAAAACCCCTATGT

GTATGTGCTACCACATTCCGAAAAAAAAAAA

>901

GGTGTCTGCTTAAAGAACAAGTACATGAACCACATTCGCAGTTCTGTGAAGTTGCCATGG

CTCAATATATGGTATCATGTATAATCACGCAGGACAAGGTCAACATGATCACTTGTACTT

TGTAGATTCTGAGATGACATGCATGCATGCTGAATTTTTCATGGTTTATTGTAAAAGGAT

ACCCATCTTATGTTCTGAGTAAAAAAAAAAA

>902

TGAGCACTTCATAATCTACTTAACCTTTTATTTGGACCCGCCGAATATTCATTTCAAACG

GAAACAGCAAGTCCAGCAAAATCTGCCTGATTAGAGAGATGCACGCATGTTTTAGATCAC

TCGTCAGCTCAATCTTTGGACGACACTTGGCTCGTGGGGATCTCACAAGATTTGGTGTTT

GTTGTGTTAATTGCAAACCTAAAAAAAAAAA

>903

ACATGAATACAAAAAACACGCAACCATAAGAGCCAGTTAGCTTTGATAGTCGCCTTTCAG

TGCTGGCTGTCCGAAAGCAAGCACCGGCGTGTGTGAGCTGTATGATAGAGAAAATATGGA

TTATGGCACGGCATGTGCAGGTTAAAGGTTGCTTCACGGCCACGTGACAACCAGTGTGTT

TACAACGACTTAATCCCCCCAAAAAAAAAAA

>904

CTCATGCATGAACCACGCTTTGCGCGCGCTCAAGCACACGTGCAAAAACTTTAAAACTCA

CTCTGTACATACACCGATGACAAAGCAGCCCTAAGACATGACGAGCACGTACATCATTGG

CTAATTTTCCGCGTGAGCGAGCTATGGGAGTCATGAAAACACAATCCCAGCGAACAGGTC

ATCTTCGAAAATGTTCTCGCAAAAAAAAAAA

>905

CAGGTTCATGCGTCTGTTTTTTTTCTTGTGACGATTACGGATATCGTTGGCCTTCTGCCG

TGCAGCAGTGGGTGCAGATTTGCGTGCGTCCGTCATCTCGTATTTTTTTTTTCAACGGAT

GGTTGTTCAACAACTTCTGTTTTATCGGTTGGTTCAACATACCCAATACCACGTTCTCTC

ATTTTTGATCCCCTTCCATTAAAAAAAAAAA

>906

TTAAAAAAGTGGGGTGAAGCGCGGTATTTTGTGTTTGCGCGCCTGCTAAGGCCATATGGG

GTTTTTCAATGCCTAGTACCCAACCCCCTGGTTTTTCAATTGAAAGAATAGAATTTGCAC

GCCCATACAGACACCGTTGGGTTTTTCAAGAATGGTATCCCCGTGTTAAATGATTACAGA

CAGGGTCCCTTTTGGGTTGGAAAAAAAAAAA

>907

GCGGCGGGCCCAAAGGAGGGCACTAGTATGGTGTAGGGAGCTTGGGATCATGGGGCTCGG

GCGGGCCCCTTCTTAAAAAAAAAAA

>908

TCCAACTCGCTCGGCTCAAGTGACTTGAATTCGTGCCCATATGGCCCAGAAAGTGACGTG

AATGAGCACCACCGGGTCGCGTATGCCGCGCTAGATGCTATACAGGATAGAAAGATTAGC

GAACGGATCAGTGTGACGTGTTGACAAGTTACAGGACCAGGTGTTCCGTAACCGTTTCGA

TATTAGTTTTGCAACACTGCAAAAAAAAAAA

>909

AAATGCGCAAGGAGGAATTGCGCGAAGCCATTGAATATTGGGACACGATGGTTCTATCGA

AACGAAATGACATTGATCCTTCAAATGACACCTGAAGAACCGTGTTGATGTTGATGTCTT

CAGTGGATCCAATTGGTGGCATTGCCGCATCGTGCAGTGATAGACATAATTTGGTGTGGT

CTTTATATTTTCATATAGACAAAAAAAAAAA

>910

GAGGAAGTGGCGTCTGAGTCCCACAGAAAAGCCTTCATGGCCCAAGTGTTCATAGGTTCA

AGTTCCTTCACAAGTGCCCCCCCGACCTTTGTAGCAGCGTCTCGGGAGCGCCTTTGATCC

GGTGACCGATAGAAACCGTCGTTAGTGAATGCCGTCTCATCACCTTCGGGCATTGTACAG

GCTTCACTCAGATTCCATCCAAAAAAAAAAA

>911

CCTGTTCGACGTCAACGACGAATTCCGCAAGTTCTATGGGTCTTGGGAAGATGCATACTG

TGGGGTAGCTATGCCATCGTAGGACAGTTCACAAATGAAACTCGCCTTTGTTGTGGTGAT

TTAATGTANGACAATGCTCACAATGTTCATAACTGTAAATTTCGTGCTGGTATGACATTG

TAATGTAAGAAGGCATTGGGAAAAAAAAAAA

>912

TGTATTTAATCATCAAGTAATGAAAATATACGGCACCTGATACGCAGAGTATGCGCTGCC

GAACACGGTCGCACACGCTTCGGCGCACGTATATACTATGGGAGAAGTGGAGAAAAGAGC

ACCATCCTTGACTAGAAACGACCCAACATGAAGGAAATTTGTAGCCGCCAGTTTGCGCCG

TCCTAACAAGACACCATCCGAAAAAAAAAAA

>913

CTACTTGCCTGCAGTGGTTGAGACATTGTGCGATTCTCAAAATTGGAGTAAACATCTTCA

ATGATATGCACATACTAGATTGGAAATTCAACGAGACGAAACATGTAGTCCATTGCACGA

TCGTGTAACGTCAGCCGCAGAATATGAAATGTTAATATGATTTTAACATTGCTTTGTAAC

TTCAGCATATGGAAACAATGAAAAAAAAAAA

>914

AGTACAGGGACGTGATGTACTGGGCCCCCGTGGACTCGTGCTGAAGCATCGGGAAGTTGG

ACCAACAGGCCAGGTCTCGTTCCAAGCCTGGATAGGATAGGATGCCCCAGATGCACGCTT

TTTTCACCGGTAGAGGGACGCCAAATCGCTTGTCACAAGGAAAATACGGGGGGAGCCCCA

TCGCGCGTTCAAAGTCCCCCAAAAAAAAAAA

>915

CAGGCTTCGTGATGCATTTGCATAAGCCTAATGGTCCTGTGTTTCATTGTGCACAAGATC

TTCCCGGATAGAAACGTTGTGTCCTNAGCCGCAGCGTATGGAAAACCCATTATCTGGCTC

GGCCCTACCTCTGTGGGAATGTTTATCGTATGGTTGAGTGCCTTTGAACTCAGTGTGGGA

AAATGTCGCTTGATCAAGCCAAAAAAAAAAA

>916

ATGACGCGTGAAACCTGGAGTGCAGGATATTTGTATGTCCGAGTTGTCCCGATTCACGCG

ACTAGTATGCCAAAATGAAGGAAACCAGGGATTCGAAGAAGCAGATTTTTCCACTTGTAA

TTCAAAATCACCAGGTGTACAGGTTCTGACTCACCTTGAACATCGATCTGTGGAGAATAT

TGGAATATTTAGCTGCGATTAAAAAAAAAAA

>917

ACGGCTACAAGCAAAATCCCACAAATGTCGGAGCTCACAGGCCAACAAGCGTCGTAAGAT

TTGCGCAGCCCAGACGTTGCAGGGCAACTCTGTAACACTGAATTATCTAGTGATGGGTCT

CATGAGTAGTGAAAAACGACAAAGGTTTGAACTTTGCAAGTTACTTACTAGTACTTTTGA

ACCAATACTACCAAGTCGGCAAAAAAAAAAA

>918

TCCGACAAGAAAGGTTCCCGGAGAGACAAGGGGAAAGGAGATTGGTAGGATTGCACAAAC

GTAGCCGAATTGTCTTTGCACGGATTTCTTGTAACACTCAAGTAGTCTGCTGGAAAAACT

ATCTACATGTATACTTTCTCCTGTGATATTAAAGGAATTGCAGCCTATTTGATAAAAAGC

AGGCTCCATTCATCCCGGTCAAAAAAAAAAA

>919

GATTCTTGCCGATAAGAAGTGCATTCCCTGTTTGACATGTTCGGTTGTTGAGATGATGTA

GATATTCCTGACCTGTAGTTGGTAGTAGTAGGTGTGTAATTGCTTTAGCAGCAGCAATGT

AGAGGTTTGCAGCCAAATATCTGTGCAGGATATTTGTTTCAGAATTGAAAATGGATTGCA

AATATGTATTTGCAAGGAAGAAAAAAAAAAA

>920

GGTCACTGCTTTCCGGCATCCATGGGAGGACGACCACCCAGATGGTGTAGTACACCCACG

CTACAATCGAAATCACTAGAAGCGCGGAACCAAGTGCCGCGTCGGACGTCGTCATTTTAT

GTTGGGTTACACTTGAACCAGAATCGTGGGGGCGATGCCCATATACGTGGTTGCACCGGG

AAGTTGGCTCCACGGTAGCCAAAAAAAAAAA

>921

ATTGGATGCCACCAATCGCAGGCGTACAATTTGAGGACTGCATGATTTGGTTTGCAGCCG

GTGGTCTAGCTAGGATGATATGATTTCTTTACAAGTATTGAAAGCAAACGAGGAATTTAG

GGTATTGAGTGGGCAATGGACTTATGGAGTCATGTTGTTGTTCGATGAAATTGCCATTGC

TAAGCATTTGCTATCATTCTAAAAAAAAAAA

>922

AGATTCGGCGACTTGAAGAACGAGACGAGAAAAAATCTTCGCATGCTCCTATTTAGTCAC

TTTCTGAATGATGCATATTTGATATAGATTTTCCCGCATGTTAGAAGTGTCGGTAAGTTC

CGGTATCCCTAGGAATTTGACAGAGAGGAGCCTTTTTCTTGTCAGGAATCCATCCAATAG

CAAATATATGTTGATCGCATAAAAAAAAAAA

>923

GGTGTAGATACAAGTGCTACGGGTGATCTTAGCAAAAATCCGGACCGTAGATCTCTATGA

CAGTTATCCACAGTGTGATGCATTGGCGATAACAGGTTGGCTACGAGGGCAAGAACGGAA

TCAATTTTGCCTTGTTGTGGCTTGGTGAAGCAACTTTTTGCATGTATTCGTGATTTTCAA

AACCGATTCAACCACTGGCCAAAAAAAAAAA

>924

TTGCCTCACTTGTTAAAACAACTTGACACATAAAAAATTCATGCACTTCGACATCTAAAG

CATTTGACAAGCACTTTCTGAACAGTGTGCCGTATACATTGTTGAAACGTTCACTGATGT

TAAACAATTTATGATATATAACACAGTGGACATGCCAAACGCTTGCAGGCTCGTGGGCCG

GTAGCAGCCTCAAATTTGGGAAAAAAAAAAA

>925

GCGTGGGGGGGAACCCCACAAGTCCCAGGGGCGGGGGGAGCGAAAAAAAAAAA

>926

GCACGTTCGGTTGATGAGTTTCTCCGACTGCAATACACCACAACGCAAGCTGAATACTAG

GCAAGCTGAACCCGGAGACTTCTCGCCACGCTTGGACATTGATAGTCCCAACCATTGATA

ATCATCACCGTCTATTGTTGCTAAGCACTACACCTGGAGATAACTACGCATGCATGTTTT

ATTTTAAACCATGGCATACCAAAAAAAAAAA

>927

CAAGAACAAAACAACTTGGTTGACGTTTCTGATGGCTTCTAACTCACATGGACATGCATC

GAGATTGCGTAGGTTACAAGATAGTCGCGTCTACACTTACTTTTGTTCGACACACGTCGA

AGTATGCGCATATCATGGAACCTTGACTGGCATCTTGATATCAATTGAAATTTTCGCGAC

TGCTCTGCAGCATTGTGGACAAAAAAAAAAA

>928

GCGTCGTAGGAGGTCCCTGCGTCCGCTAGAAGGGCTTGGTCCTCGACTGTCATCTCATTG

GTCTCATCAGAAGACTGCACAGAGCAGGTTCGCGAAACGTGATGCTCCAAACAGCAACCT

GAGCTCGGAGCAACTGAATCAGCATGCATCAGCGCAGCGCGTTGGGCATCCACCACAATT

TCAGAAATCACGCCCTCGGGAAAAAAAAAAA

>929

ATTTGGAAGGGTCCGACGTGGTAGCTGTTGAGTCCAGAGTTTTCCCCAAGGTGCGTGCGT

TCTTTTGAACCCATCACCAGCACCTGACAAAAACCAATCCCAGCCCAGGCACATAAAAAA

TGGAAAGAAACCGACTATTCTCATTTTTTTTTTTTTTTTTTTTTTTTCGGGCCAAATTGG

AGAATCCCATGGTGGCTTTCAAAAAAAAAAA

>930

TTACCCTGGTTGCCAAAGGAATTGCGCAATTGGAATCCACTGCTAATTGTGCATGGAAGT

GTAAATTGTACCCGGTTGAGTTTTTAGACAGAAGGAAGAAGTTCAGAAACTCACGCCCCC

CAACGTATGGGACTTGACCGTTAGGAATAACGGAATGTTCCATAGGGGTTCCCCCATTTT

GTTATACTCTGTCTCCCCCTAAAAAAAAAAA

>931

AGAGTGGAGATAGACGTAGGATGAATTGTACGGCTCCTGGGGACAGCTATGTGAGAATTG

TGAAAATGAGGCTGCTAGGTTCCTTTCTGCAGTGATTTGATTTGAATCACAGCATGAAGA

GGAAGCTTCAAAAAACGAAATGCACATCATGAAAATTAATTGTAATTGTATTTGGATTTC

AATGATGTCATTTGTTCCTGAAAAAAAAAAA

>932

ACAAATGTCACCTTCACTGGTATTATGGATATGGAAGCAACAATCACAGTGCCGCTTGTT

CCCCCTTTTGAGAACCATGTAGTTTCAGTTTGGACACCATTTTGTGTTTGGGCTGTGACA

TTGGGCACAGCTTTGACAGCATTGGCAGGTGCGTCAAGATGAAATTGTGAAATTTCTTGC

CAATATAACACCTTGTTACCAAAAAAAAAAA

>933

TCAGCGATAATCTTAGGCACAGTCAAACTGTGTATGCAAGAGCATATGTGTTCACTGTAA

TTCAGGAACGTTAACCCGTTCATCAAAAAAATAAACGATTCCGCACTTGACACGAGTAGC

CTGAAATCGGGGACGGATCGCTGCACGAGCTGCAGTGGCGTGCATAGTGACGACGGGCAG

AACCATGGTGCAGGGTGAAGAAAAAAAAAAA

>934

GCCACGGATTGCGCGAAGCAGCACACCGTCTCCAAACACAGGCACAACTCCGGGCAGCTC

TGCTCCTGCATGCGGCCAGAGCACGGGCAATGCCCCGCGCAGCAGATGTATCTGGCGCTC

CAGGCAGCCATAAAACAAACTCAACCCATGTCACAATCCCAGGACGTACCGAAAAATACC

GCAGAGAAAAAAAAGCAGCGAAAAAAAAAAA

>935

CGCGGACTTTCAAGCAGCTATAGAAAGCTCACACTCCAGCCGTATGCGGTGGAACTCGTG

CTATTTTCGGAATCCAATGATCGTCTTCTGCTTCACCACAACTTCTGGTTGATAACAGCT

CTTGAGTGTGATTTCGAATCAGACAGTGCGGAGTGCTGAGAAGCGTGGAAGAAACGGGGT

TTGTTCGGATCGGTCAACCCAAAAAAAAAAA

>936

ATCCCCATCGAGACAGTATGCTGATCTATGGAAATCCATACGAAACAAACTCTTCGCGTG

TTTCATATGCAGTCGGACGCTAGCATGTGAGCTCTGCTTGCTGGTGCACCACCGAGGATT

TGATCACAGTTGTCATTGAGCATGTCTACTCCAGCATCTTGGGTTGAAAATAAACAATGG

TCACATCGTGAGAAACGCATAAAAAAAAAAA

>937

GGCGCTCAGATAGAGTGCGATTGAGCCACGAATCCAATGCCGATAAGATTTTGGTCATAT

GCAAAACTCTCTTGAGCTTCTTTCTGTTTCCTTTCATTGAAGTGAGGGTGCTAAATTGGT

CCCGCAACCTTGCGATGCAGCCTTCTTTGCTCACCTGGCATATTGCAGTGCTGTGTCGTT

ACGATTCAACACTTAATCATAAAAAAAAAAA

>938

TGTAGAATCCTCCTTCAGATGACGCTGCAAGTAGTCTTGCCGCGTTTTCTCCGAGATGAA

AGTACGATGTTGTTGCCCACAAACCTATCAAGGCTAGGAAAACCACCGAACAGGCGCGAG

AGGAAATTAGCGTGCGATTTTGGCTGCGTGAGCGAGAACCTATTGGCAGTAGCGGCAGGT

GCCGACGTAAAGGTCGTTCCAAAAAAAAAAA

>939

CGCGTATACAACTGGGATGTGAAGGGCAGACGTGATGCAAAGGCGAACCAGACAGAAGAC

GTCTGAGATAAAGTGACCCCGATCCTGGCCATTTCAACGATAATGATGCCAGATGAACTT

GAACCTCATAACGCAGACATTGTTTCATGGGAAGATGCTTACAGCCACGGGATTTCATAT

GCAACATAGACATGGTTGTCAAAAAAAAAAA

>940

AAAACTGACCAGAAATTCAAATGTCATTGATTTCACGTGTCCACTTGAATGCATTGAACA

TTGGGCTCTTTGTCCTCACAGCGACAAACCTGCTTGCTTTTGAGGTGACGGAGGTGCTGT

AGATTCGATTGTGATAGGATGCGCGTATGTGCAAATGACACAATTTCAACGGCTGTACAT

CAGAGGATTAGCTAAACTTCAAAAAAAAAAA

>941

GCACCTCGTCTCGTAGCTTCCCCGCTCCAGGGGTCAGGCCCACCATGCCCACGGCGCGCC

CGCCTTTGGAGGAGGCCTGGTCGTGGTTGCGGACGTCGTTGTGGGCACGTTTTTTTTGTT

TCGCGGCGGCGTGCATCAGCCCCCAAGCGTAGGCTCGCCCGGAGCGGGTCACAGCAAGGC

TGTGCAGCGATCCGCNCGCGAAAAAAAAAAA

>942

AGGGTCATGTGGAAGACAAATATGGGACTCACTGGGTTTATGTATGTTTTGTTGATTAGC

ATGTCAGCAATTGATCAACAGGCAATTCATACTGTTTTTTTGAAGGGCCCCCAGTCATGG

TAGGTTTGAAGCATGCCACTTGCATTCAGTAGATCAAGGATCACTGTTGGTTTCCATTAA

TCCCACTCATATTTCCGGTCAAAAAAAAAAA

>943

GGTTCCATTCAGATGGCTCTCCCTTCGTTCACAAGCTAGGCAACATCCACAAACATGAAT

TTCATTGATGTTTATAGAGCTTAGATTTGAATCAGTATTGCACGTGTTTTTCGGTAGATC

ACCCGAACAGAGAATCATTTGCAATGTACAAGAATACTATATTTGTATCTACGAATGAAA

ACCAAAATGTGATTGGCTACAAAAAAAAAAA

>944

TAAAGCCAATGACCATCTTTTTCATGAGTTTCCTGAATTTTGCAATATAGACGTTCCACA

TTGGCAGTTGAAGCAATACCGCAGCTATCCAGTAGAGTCAGATCAAGATCGCCGTCCACA

ATTCGCCCGTTTTTTTCGTGTTGCGTCACCGGCTGTCCAGGGGTGGTGTACGGGCTAGGA

TGGCCCGTCAGCCCCCTGGTAAAAAAAAAAA

>945

CTGAAATGATGAGACAGGAGAAAACAATATAGGCATATGCACAGCAAATCAATAACCATA

AATAGTGTTGAAGCGCCTTATGTCCTCAATCTATTCAACAACAGGGGATCTCAAGGAATA

ACAGCATGAACAAGTAACAGGATTCAGACACATTGCCCATTTCAACATCTTGCAAAAACA

ACCTGGCAACAACCTTGTAGAAAAAAAAAAA

>946

CCATATCCGAGAATCGTGCCGAAGATTTGGAGACCGGTGGGGCCCAGTATGATAACGAAG

GCCAAGAAACGGCGGGTGTCCACCTGGCCCAGTCATAGGAATGAAATCGTAGAGGTGGTC

AGATGAAAGGAAATTAAGGTTTCCCGTTGGACAATGCTCCAAGTATTCAAGCTTTAATTT

TTGATAAAGTCGCACGTGTCAAAAAAAAAAA

>947

TCCAAGTTAGGTGACAAGTGCGACGAGGTTCGTATCGTTGCTTTAGCGTAACAGAACACG

GTACCGTCACAGTCGCTTTTGGCTTATTTGGACCATGCATCACATCACCCACCGCTGTAG

CGAAAGGCGTGTCTTTTGAAAGGTGCTTCACTCTATCTTGTGTATCTCCCTCTTTGATGT

GTCAGCTTCTCGCAACACCCAAAAAAAAAAA

>948

AGACGCACGCGGTTGGGGGTCAGCACTCCCTGCCTCATGGGGAAACCCTGCTTGTCGCAG

CCGCCGGTGATCTTGAAGACGTACCCCTTGAACTCATCACCGAGAGACTCGCCAGGAATC

TCCGCAGCAAGACGACGATCGTAAAAGTTTCGGAGTTTGCTCTCATCGTCAATCTCCAGT

TTTTTTCTGGCAACCCGTCTAAAAAAAAAAA

>949

TAAAAATTNCCCAACCCACCACACCCCTCCCTCCACAGCCTTCACCCATTATGTAATTTT

GTGTTACTTCAGGCATAGATCAAGACATATTAAAAAAAAAAA

>950

CAGAAGATGTAATTATGGCTTTTTACACTGAATGCACAACATGTACAAGCGGTTTCTTGT

TTCAAGCATTCCTCATGGATATTCGTAGGCAAATATTATTGTCCTCTGCACAATGATGAA

GGTAAAACAGATCATTCACCCACTAGGGAAGACTCAAGGCATTGCTGTGTGTTTGAGTGA

GAAAACCTAGTAGCACTTCTAAAAAAAAAAA

>951

TTTAATTTCCCCCGTCAGGGGTTGGGTGCCTTTTCCGGGGATTGGGGGGAAGGGTTGGGG

GGAATTTTTTTTTCCCCTTTTTCTGGTTTTTTTGGGGGGGGGACCAGAAGGTTTTTCCTT

GGGATTCTTGGGGGTGTTGGCCAGGTTTGGGCCAGGCCTTTCCCAGGCCTTGTTGTTTTC

CGGAGGAGGTGGGTCCCCCCAAAAAAAAAAA

>952

AGGCACCCCTGAGTCCATTGGTGATGTCAAAGACAGGGAAGGTTCTCGCAGAGATGGTAA

ATCTTGCTACAGCCAATCAAGATCATGCAGGTTCGGTCGCATATTGGAGAAATTCATCCA

AAGGAAATCCTGAAAACAATCAAAAATCATCTACCGAAAAACAACAACATACTGAAAAAA

ATAAACAATATGTTGGTTTGAAAAAAAAAAA

>953

AGTATGCGTGTGGCGGGCAACTTAGCGTCGTCATGGTGGGCGGGGCAAATTTGCATCCTT

ATTTTGGCCGGTGCAAAAAAAAAAA

>954

CCCACTACAGATAGTCGCAATAACCTCGATAGCTGGATATCGGTAAACATGTTTATCCTG

AGGCGGGTCTCCTGACAAAAGGAGTGAACTGACTTTCTATGACACGCTAGCTGTATTGCC

AACCTTGAAATGGGATTGATTTTACGCTTCACAATCTCGCAAAGATTTGTGCTAGTTTGT

AATCCTAGATCATCCGGTTCAAAAAAAAAAA

>955

TCCGCCTGCTTCAATATGCACACACTCACCGCACTCGTTTCGTACCATCAAGCTGCCTGG

GAATGGGGCAGCAGTAGTGCCTTGATCACTATAGCTTGCAGGTTATTGCGATGCTGAGTG

GTGCTAGATCAAGGGGTCTGCTGTACTAGAAGTGCATGTCAAATCTGAGTTTTGTGACAT

TTATAACATATTATACCTGCAAAAAAAAAAA

>956

GACGGTTAGCGGTCAAGTGTTCTCTTCGCGTGTATTGTCTCCGATGCACGACGCCCGGGC

TGCTTCTCAAAAAAAAAAA

>957

GTATAGATCCTGTAAGATGCGTCCCTAAGGATCTCCTGCCAAAATCCCCGCGGGGACACT

GTCATGCAAACCCGAGCAACCACCTTAATCACATACTTATGTGGCAAAATGATCGCACCA

AGACCGGGCTCAAAACGCTCTTGCTATTGGTACACCTCTGGAGTCTTGTATCCAAGACAC

AGTGCATGATCACCGTATACAAAAAAAAAAA

>958

ACTGGTGCTGCATCTTCTCACCGGCGCAGGATCTCGCAGGAGTCGGATATATCAGATGTG

GAGGAGATACAGTAGTTGCGAACCTAGCAGTTGTTTTTGTTATTCCGCTATACAGAAGGC

TACGTCACACCTGGCTAGCAGGATGAAAAACCATGCAATAGATGGATGTACTGTTCAATT

TATCGTGGTTCCCCTCGTTTAAAAAAAAAAA

>959

TTGTTGACAGAAGTTATGGGTTGCGGGTTCAGGTTGTTAGCTAGTGGTCAACACCAACGC

ATCCAGTATATTTTGGATGAGCTTAACAAGCAAGCACCAGTTTGTGGTCTTTAGGCTACT

TTAAAGACCTGCATGTTTTATCAAAATGCGTACCCCGTTGAATGTGAAGATTTGTCAAAT

CGCTGGTGTTTTGTTGCTGTAAAAAAAAAAA

>960

TCAACATTGAAGCATAGTTAGAGAGAGAATCATGGGTGAGCAGGGTGTGTAGGCTACCGA

GCTGTCCTTGCTTTAACCATGGAGGCAAGGATTGGGCGTACTTAAGCACTCAGGATGTAC

TGATGTATGCATGATGGTGCTATGCACTCGATGTATGTATGTAGTTTATATGGTTTTTAA

GATACATGAGATGCGCCGGTAAAAAAAAAAA

>961

CATGGGCATACACAGACAATCGGCCAATGTCTCATACAATTGGAGGTGGCGATCTGAAGC

TTGAGGGGGGAACCAGTCAAAAGCTCGCACTTCCCGTCGATAGAGCGACCGAAGCCTTCG

GTATAGATACAATGGATTCATGTTAGCGAACAGATATGTATTCGCTGACTTTAGTAAATC

AGTTCAGGACTATCTCAGTGAAAAAAAAAAA

>962

AAGAAGGGGGCCCCAGATTCCGACTAGAATGGGGGGGGTCCTGAATGCCCGGCCTGGGTT

GGCCTACGTTTTTTGTTTCCCGGGTTTGTGGGAAAGCTTTTTTTCTTGCCGGGCAAACCG

GTTTGGTTTTTTTATACTTCCCTAGAGGGCCAAAAGGGGGTAATTGGGTAATTGGGAATA

CCGATATTTGTTGGGCTCGGAAAAAAAAAAA

>963

CGAGAAGTTGCTTTCTCCACAACAAGTCGCGTCAACAGTCGAAAGAGTCGTTACCAGAAT

ACATACGGCGGCCTTCTTTCAGAAAACTATAGATATGTATATGTTGCGCAAGGTCACGTG

CTGTTTGCAGGGGGGTTCAAGACATCGACAGGGCCGAGGCTGACACGATACCGGTCACGC

CACAGGGCAGCGAGTGTACGAAAAAAAAAAA

>964

AACCAGACATGCAACCTGGTTCCAGTCACACCCCGAACAATAAGCGACAATGGGGAGCAG

TACTGTTGCTGACCGAGCCTCCTAGTATTGGTGCTACTTATAGTGTATTGTCATATCCGT

CCAGCAAGATGGACCCACCCACCCCACCATACTGGCCCGGTGGTCTTTGTAGAAATGCTG

TTGCCAATTATTTCAAGACCAAAAAAAAAAA

>965

AGCAGATGATCTATTAGCATGTGAAGCAAACCCACTGTTCACCGGGAATGGTTCCAGTGA

CTTTTTGGCTCGCATTCCGTTGCTGCAAGTGTTGAGCGATGATAGAGCAGGATTGCAGGT

GAATAGGTTACCGAGTGTCTTATAGGTAGACCAGCGATCAAACATGTGGTCTGTGGCCTG

TACAACGCAGCATTCGCATCAAAAAAAAAAA

>966

AGAGAAAGAGGCTGAAATTGCGCAAAAGGACGTAGATGCGTGGGTGCAAGCCTTAAAGGA

TGCAAAGAAACACTCGACTTGGGAAGGGAAACAAGATGAGTCGGTATAGATGTCACATGT

TAAAGTCCTCCATCTGGTGTATATCTCAATGTAAAACTGAAACATTTGGGTAGAATCAAT

GTACTATACAACATCACCTCAAAAAAAAAAA

>967

CGAATGGTGGTCATGCACACGTGGTGCATCCACCTCTCGCAGACAAGATTGCCTCGCGAC

AATTGTATGGATTTGTTGAGAAGTGGTGAATTAGAGCATCCACATCCACAGGGTGGTATT

CACTTGTAGTAAGGAGGAAAAGTCCAATCCAGCCTAATATGCTGGCGGTTAAATCGATCA

TTGTGCCTCATGTTTGATCTAAAAAAAAAAA

>968

CCTCATCAGGACAGTCAGCTGAGCTGTGTAAATCCGTATAAAAATAGACTCTTCGTGTAT

TGCATATGTGGTCTGTTGCTGGCCCGTAAGCTCTGCTTTTTTTTGCACTTCCGAGGATTT

GCTCACAGTTGTCATCAAGCATGTCTGCTCCAGCATCTTGGATTGAAAAATGAAAATTGA

CATTTTGATGAACGCATATCAAAAAAAAAAA

>969

CCTTTGGGCAAGCGCTAAGGACAAGCACTCAAAGAGATTGTATTAGTAGGACGCCCTGTA

AGAATGCTTGCTCATTTTTGGTAAGCATACCGTCATCTTCGCAACGCGGTTGAAACATGG

CCTTGATGGCTACGATATCTGAGTCGGTACAATATGTTTCTGTAACACACATTTGTATTG

TGACTCGAGTATGTCAATGTAAAAAAAAAAA

>970

CTGGCTCTCTTGGTATGGTGATCGCCGTGAAAATCACAGAAGGGCTGTAGGAGTTTGGGC

ATGGTTAGAATCATTCATTTCATGATCCGAGGATGTTGAGTGTTANGCTATACAGTTCGA

GGATTGTGNCAATGTTTCTGTACGGACTTGCTCGTTTTCCATGTTTCGTCTGTGCCTTTC

TATATTGATACTTTAACTGGAAAAAAAAAAA

>971

TTTATGGAAAGGGTATGTTTTGGAGCCCAACTGGGGTGGACCCGGGGGGAGCTACTTTGG

ATTTGGAGGGGGGCAAAAAAAAAAA

>972

CCTTCACGGCCCATCATACGCCCGCCCTCACGACCCATCATACGCCCGCCCTCACGATCC

ATCATGCCTTCACCTTCGCGGCCCATCGTGCCGGACTGCTGGGGAGCCGCCTCCTCGACC

TCCACAACAACCGGCATGGACATCCCAAAGCGTACGGGAATGGAGCGCATCATGGCCATG

GGCGAGAAGCTCACGTCTGCAAAAAAAAAAA

>973

AGCCATGCAAGCTGTTTAGTTCATCATAGTCTGAGAGCAAAGCCTGACTGTCATCAGCTT

AGCAGGAAGTTATACTGGGCCATGTGGGTCTTGTTTGGAAGTTTCTCTGCCGGGCATATA

TGCAACGTTGGTGAGAGATTTTGCACCGAGGTTATTTGACTCTTCCTTGATTGTTGGTAA

TCAGATTCAACATCTTGTTGAAAAAAAAAAA

>974

GACCTTTAGATGCTGTGTAGTTTCAAACATGTCATCGTCAACGACACAGCTGCAGTCGCC

ATTGCCTGTACAAGAACAGAATATAGGTGAATGCGCGGCCATCACGGATTGAAGAGTGAC

GTGCTTTGCTACCGGCAACGCTATGATTAGCTGGCCGACCAACAACAGTGCAACATTTGG

ATTATAATTTCTGGTTCATTAAAAAAAAAAA

>975

CCTCCGTGTGTGCCAGCCCGTTCCCTATTGGGGAAAGGTCCTTTGGAAGATAAGACATTC

CAACCAAAAGGGTCCCCTTAGTTTTGGCGTCAATGGTAAATGGTTCCATAGATACAAACC

CCCCGGGGCCCTTGCACCCAGGCCCTTGTAGGTACTTGCATAAGTTTTTGGTTTTTTCAA

AAAATAGCAAGTCCCTGTGGAAAAAAAAAAA

>976

GGAATCCCGGTTGAATCGCTAGATACGTGGTACATATGTATCATGTCCCTTTACGATTGC

CCCAAGGTGTGTCACGAATTAGAGGGGACATGTCGAAACGACTGAGGGATCGTGTGTACG

AAAAAACGACAGTTGACTATGGAATGGTGTACGTCTCTAGAATGTAAATTGAGCTCACCA

AATTAAGCTTGCATTTGCCTAAAAAAAAAAA

>977

GCGAACCATCCATGACACTGGTGTTCCATGCACAGACATGGACAGGCACCAGGACAGAAT

GCAATCGCACTGTAGCGATTTGTGAAGGCTTCACGGGCACCAGACGCTTAATGTACACAT

TTGAAAGCATATCTTATGCTTATCTCAAACACAGCTATCTTCACAAGCATGGTGTATGAA

ATGTTGTCATTATCATCGCGAAAAAAAAAAA

>978

TTCTTCAATGACATTTGCTTAATCTTTTGCACAGCCTTGCCCAGCCAGGAGGCTCGCCTT

TTGGGTGGCCTCAATGCGCTAGCTTCCAGGATTGTAGAGTGCGCCTATGTGGAAAGAACA

CCACCCACGCATGAGTGTGATTACTGGAATGCACATATTTGCGCAACCTAGAATTAAAAT

TGTATACCAGACTAGCATGTAAAAAAAAAAA

>979

TGGTCAGTCATGCATTTCTCAGAGTAGATTTGCGCCACCACTTTCAGGATCTGTCCGCGC

GGCTTGATGCGCCCCACGCGGTCCGACTGCATCAGGTGCCCAAACCCGCCCACGTTTTTG

CCGGCAGCCGCAGCCTTCTTCTTCTTCTTCTTTTTTTTTTGTTTTTTTGTTGGTTTCCTT

GTCCTCCGCAGGAACCCACCAAAAAAAAAAA

>980

CCTGGGCATGCCTCAGATGTTCTGTAGTGGCCAGATGAAGATTCCGAAACTCAATGATGG

CTGCTGCTGTATGACCGGCGAGCCAACCCTACATACACCGCACGCAGCAGCAATCAAATT

AGTGTGTGGGCAGACTTCGCTGCGACTATCGTTGGTTCACACAGGAAGAACCACATCAGG

CTTCATGGTCTAAAGAATAGAAAAAAAAAAA

>981

GATTTGAAATGAGTACTGTTGACAAAGTTGAAAAACACACCCCTAGTGACATCATAATTT

CTAGGTAGTCTCTGAGTGTATTCCTGCACCTATACATTCAAACATACAATCTCACCTGAA

TATGAGGGTTCTAGCTGTGATAATCAGGAAACAACAGGGAAAAACCTAAAAATCAACTTT

GCGACAAAAAAATACACATGAAAAAAAAAAA

>982

CGGACTTTGCTAGCCTCGTGTAGGTGCATGGGTTCACGATTGAACTTGGTCCATTGCATA

ATACAAACAGCTTTTCCAGGCTTGGGAAGTCCAAGGTTATCGCTGGAATTGCATAAGAAC

TCCTGCAGTGTGCCAACACACAGAACTCGATGCATTCAGAATTTTCAATTTTATTCTCCC

TATGCCTTGCGCACTCAAGCAAAAAAAAAAA

>983

TGTATTCAAGGTATGTAGTCTTGTGGATTTGTGAACCGATCCCTCACGTCTAGATATTGC

ACTGGAAAAAATGTCCGTATTGAAGTGCAATGTTGTTGGTCGACCAGCTCATGACAGCGC

CGCCTTGTGGCATTGCACATTACTGACATATATTTAGAGTGACATGTGATAATAGCAATT

AGTACCAGATGTCATTGTCGAAAAAAAAAAA

>984

TGTTCCCAAGTCGTATTATCCCAACCGACATTCCATCAGTCCGCGGGATTAGGGATGGAC

ACGAACCATGGACAGAAAGGCAGAACGCATCTCGCGATACCGGAAGGCGTCCGTCAATCT

GAGCGTAGCGAATGATGCAATCCAGGAGGCCAAGGCAGCTTCCTCTCCGTCGGATGCGGG

CGCGGGACGGGATGCCATCCAAAAAAAAAAA

>985

CGTGGTAATGACCGATGCTGGCTCTGAGGGTAATTTAACAATCGAGTGCACTATGTTTTC

CATTTAGACAAATGCCCCAGCGTTTGATCAGGGAGTGGAAAATAGAAGACAAGACAGTGG

AAAATATGTTTGTTCAGCCTTAGTGATAATGCAAGGAAATACACGTCAACCAGTATATTT

CAGCGGTGATTATGTGGTACAAAAAAAAAAA

>986

ACATAAGATAACTTCTGGAGATTTTTTTTGCCACACACCCATGGGTCAAACGAGACTGCG

AAGTTGCCACGGCAGTCACATATTCTCAATGACCAAACACAACTCCCTTGTTGCTTTCAA

TTACATGGATGTTTAATAGATTCACATTTGACCTTTGGCGTACACACAGATTTGCTTGAT

GTAAGCAGGAAATAACCAAGAAAAAAAAAAA

>987

AGCGCCTTGCGTGCGCACCATACGCTGATGGACCTCACCAGAGCGTCTTGCATTCGCACA

TGTCGGGGATTGTTTGCGAAAACAATACGATGATTAAAACCTTAGGAAGACTACGAAGCC

ATGTTTGAAAACGGTTAGTTCCGGATCAAGCACAAACGAACGTCAATGTGAGTTTTGGAG

TCACTCAATCGGAATTTTTTAAAAAAAAAAA

>988

TCATGTGTGCAACTGTTGCCACCTTGTTCTACATAACCAGTACCTGAATAGAGCAATGCT

GTAGTGCGCGAGTCAGCAGGACTATCGTGAGTTGTCATAGATGTAAACTCAAAGGAGACA

GTCCCTCTAGCAAGTGTTATGAACGAGGATACAGTTCATGTTGAGAGTATACCCGACATT

ATTATCCCTAGGGGAATGCTAAAAAAAAAAA

>989

ACAGTCATCTTTCGTGCTTCCCGATCGAGCTGAGCAATGAACGCATGCAAGCCTTGTTTT

GTCAACTTAGACAAAAGATTGTTCGCAGACAAAGCCAGTAACCGTATACAAAATTCCGCT

GCCTGACACGTTCCGCGCTACTCAAGCTCGCTGAGGAGGTTCGCCCCAGTCGCATTGACA

AGGCAGCAAATGGAAAGCGGAAAAAAAAAAA

>990

CAAAACACACAGTCCAAGGAGAACGGATCTCTTCATCCCCATGAAGACAGCGTACTGATC

TGTGTAAATTCATACGAAACAAACTCTTTGCATGTTGCGTATGCAGTCGGTTGCTGGCGC

GTGAGTTCTGCTTGCTGGTGCACCTCAGAGGATTTGGTCGCAGTTGTCATTGAGCATGTC

TGCCCCAGCATCTTGGACGGAAAAAAAAAAA

>991

GTTCAAGTGCTGGTAGTACTTGCGTAAGCATGTGTACTGTGTGCATTGTCACTGCGTTTG

AAGCTTGTAATTGCCCTGGAAAAAAAAAAA

>992

TTGGGGCGTGCTTCCCTGATATCATTTAAGAACTGGACTTCATGCGAAAACATGAACAGG

AACAGGAACATGATCACATACTCATACAGTCGAGCTCTGTACTCCTCGACGGATTCTTTT

CTTGTGGCAAGGTTGATGGAGTTCTCGCAGCAACAGACATCGAAAAGGCAAGCGCACGAC

AATAACCAAGAAAAATAGCGAAAAAAAAAAA

>993

GGTGTGCTCTGCGATGGCCGCCGCTGTTGGCCAGTAGAACTGTTGGTTGTGTGCAATGAG

TGAAGCGAGCAGTTGTTGGGTGCAGCTGGTCACATGGATGAGATGGATGGTATGACCAGC

ATGGCGCACAACCTAGAAAGCGACAATCGCATTGCTGTAATATTAAGTGTGATGTTTTCA

TGACAAACTAGAACTCTCTGAAAAAAAAAAA

>994

TCCGGTATGAATCAACAACACATTCAGTTCATCTGTAAGTGTGTCCGATGTGGTTATGAT

CAATTGCAGAAGCATCAAAAACAGACTATGTGTTCAATAACAAGAAACAATCCTAAGTTG

TATCGACACTGTTGACTAGATTCGTGTATGATCAATGACAAATGCCGTTGTAAGCTTGTC

TTAATCAGGGCGTGGTGCGGAAAAAAAAAAA

>995

TCACCAATCGTGTCTTAGCAAAGCGTTGAGCGAATGTATACAGACATAGCCGCAATAGCC

GCGATAGCCGGCCGCCAACAAATGTTATGCTTGTATTATGCTAACATATGTAACACAAGC

AGTTCAAGATGATGTGGCTGCATTTTAAGGTGTAGCACCTTGACTGTACAGTTCGAAATT

AATGAGGCCTGGTGTTTCGCAAAAAAAAAAA

>996

ACTACCGTTCCTAGCCCATACGCATCTGGTTGTGGACACATGCAGCTGTCAAGAATATAT

GCAAGCGCTCAAGGAAGCGTGTATGTTGGAGAGGGAAGACCGTGGAAGCCTTGCATGAAA

AGAATTAGGGACTTCATCGCCGCGTTTAAGCGGGGTATAATGTGTATGCGCGTGTATTTG

TAAGAAATCATGGTGTCCTCAAAAAAAAAAA

>997

ATTTTCCCAAGTCTCGAATTTGACAAAGGAGAACTTGATACCAGCAAAGTCATGAACACG

AAAGGGAAAACATTTACGAGCAACCTCACGAACGAGCGAGACATTTCAAGAAGCAGGCCT

GCTCTGTATGCTACCGGGAAGAGTCGTTATTGAGCGACGCGTGTGTTCTATTTCGGTCGT

TGCGCTGGGGAGGGTGTGTCAAAAAAAAAAA

>998

CAAGGAAATGGTATACAGAGCCAACCTCTTGCTTTCCCAGTGGATGATAGCTAGATTGCG

GGTGCAATACCCCGATGCCTTGATTTTTCCAGCTCCACAAGATATTCTTGAATGCCAGTA

GCAGCAGAATAGTTTTTGAGGGTTGATATTCTTGGATCCTATATTCTGATGTGTGAAATG

TGAATATCTTTAGTTTGGGCAAAAAAAAAAA

>999

CAAAGACGCGTTATTTTGTTTCACCGCGCTAGAGTAGCGGAAGGTGAATGGCCCCCATAC

CCTGTGAAGATGTAGAAGTTTCAGAGGTAAAGGCAGAGACTTAAGTGATGTAAAGACTTA

CGTTACCCCTTTTCCAGGGGGGGCGGTATGATCAGTATTCATTTAAGAGTTTTGGATTTA

GTCCTGATATCTGTTTCCGCAAAAAAAAAAA

>1000

TAATCGGCATGGTTCCTGTGCTGTTTTTGAGCAGGTATGGGGGTTCTGCTGAGAAGCCAT

TTCCATCCTTTCAAGGTCTCCGTAAGTTTGCTTAGAGGCCTGACATCAAGCTTCACTTAT

TGTAATCTCTAGACGTGTTCGACTGCTGCACCTGATGTGACCAGAGCTAAACTCCTACCT

ATATGTAAAATATACTCACGAAAAAAAAAAA

>1001

ATGTAGACGATTTGGAGTCACTGTATGCACAAAAAAAAAAA

>1002

GAGACGGCCCGCTCACTTGGTAGGTCCCTCCCGCCCCGGTCAGGCGCACGGTTTTGCGAA

GTGTCGTTGAGGAAGGGCTTTGCGTCCATCCAACCTTCCATCGGTCATCCGCACCAGTTC

CAGACACACTAGCCTTTACCTTGGGGTCGTTTACCAGCAGGTTGCTATGTGCGCGTAGGT

TCGACCAGCCTGGCGCCTGGAAAAAAAAAAA

>1003

GTGCAAAAGCAGAGGCTTCCAAGGTGGTTCCCATTCTCGTCTTGGCTTCCACGATCTTCT

TCAAGATTTGCCGAAACTTTATTGTCAAAGCATCCGCCTGATGGTCAACAAAGCAGAGAA

TTGAAGCAGTGAGGAAAACAAACAGACTTACCAAACAAGCCTTTATCCATGGCTGACCGG

TGTATTTATGCATGTTGATGAAAAAAAAAAA

>1004

GATGCTCCGAGGTGTTTTAGATACCATCAAACCAAATATGCCTTGCTTCTCAGTGTACAT

AGTTTGAGCAGTTATCTGCAGACAAATTACCCGGGAGTTTTGGCATGAGATCAACTTGCT

GTTGAGTCCAGCAGTACCCGGACACCACTGACACCATTAGTAATCTTTAGATGTTTTCCA

GTTGCTTATTTTTGACTTTCAAAAAAAAAAA

>1005

GAACAGAGAAAGCACCCATCGATGGTGCATTGCGCCCGATCGTATAGCATGTGAGTGTAT

GCACAGGGATACTCGCTTTGTAGCAAACTCTTCTCAGATAAAGGGCCCACGTGTAGTCTT

CAAGGAGTTCTGAGTGTGAATTGAACAGAGTGACCATTCCTACAGTGCGCTAAATGATTT

CACATATTCATATGCTCATGAAAAAAAAAAA

>1006

TTTTTAGGTCATTCCATCAAGCCCTGGCATGGGAGGCTTCTGCGTCGGCCAACGACTTGA

GGAAGCGCCACTCTGGCTGGCAAGTCACCTTCCATTAGCGAGCAACCGTGCCTTAAGAAC

AACCAGCACGTCGAGTCCCTCGAATCACCATCCGACAGTTGGATCGATCTCTAAGCGAGA

ACAGTAAGACGATGTCGCACAAAAAAAAAAA

>1007

CTCCAACATCGTGGATATAACGATGCTAATTTTGAACCCCCAGTCATCATGATGATCGGG

TAGGCGATTGGTGTGATCACCGGCCAGTTTCAAAAAGGATTGGATGTTTTTGGTGGCAAT

CGACTTCTTTCCAAATCCAAGTATGTGCATATTTGTGGTGATCGGGGTATGATAAATCAG

AATAGCCCCATGTTTGGTGTAAAAAAAAAAA

>1008

TTTCAGGTGCCAAGAGGAATCTACGGTGTGTATTGGTAGACTCAGGCCTGTTGAAGGCTA

TTGGAATCGAGAACATCTTTGTGGAACTAGACGAGGCTGTGATCTCTGCTGAACATAGGG

TGATGGAATACCTAGAAAGCAACCCATTGGCTCTACGTGACAATGTAATCATTTGAATGT

AACCATGTTCTCAGTTGGTCAAAAAAAAAAA

>1009

CAAGCGAAATACAATTAGTGTGACTCTCCAAACCTCTTCACAGAAACAGCTGACAAAGAC

CCTTGTTCTCTTGAGATGGTTTCACACTTCATAGGAGTTGTAATATATACTTAAAGCGCG

ACCGTAAGCTCGCTTCTTGCCATCTCAAAACTTGCTGCACCTGTTAGGAAATGCACAAAT

GGATTTTCTAGGTTTCCGGTAAAAAAAAAAA

>1010

ACAACAGATGCTGAGTCAACCACCAGCTGTTTGTTTGCGGGGACCCCGATGAGTGATTTT

TTGTGCCTCTTCGCGACTCAAATGTGGAGGTCATGATATCTGAAGCGTTCACAAATAACG

TTTGTGATCAGATATAGAAGATTTCCCTGGAAGGGTGGTAACATGAATATAATATTTCTT

GGCCGGCTATCGCGGCTATCAAAAAAAAAAA

>1011

TTCAAATGCCTCTGCAGTCTTGTCAGGCTGTCTGAAGTACCCAGTGGACACTGCAGCGCC

GCGGATCAGCACCTGGATCGATCACCCCCCAGTTACATGACTCCAACCTAATACAGAGGT

TGTAGTGCGAAGATGGATCAGATGTGGGAAACATAGTGGGTTCTTGCGCTCAACATGATG

TTGGAAACATGATAAAGCTCAAAAAAAAAAA

>1012

GGTAACTGCTGATGGGTACTTCTGAGTACCGCCTTGAAGGGAATCAATTGCAACACCCCG

TGGACTAGCCGAACCACACATCAATATTTTTTCTTTTTATCTTGTGATTTGATGCTCGAC

TATTTTGCTACTGCAAGTTCATGATTATTCGAGCAGCGGCAATCGCAGTGGTAATAATTG

GGGGCATCAAATCACAAGATAAAAAAAAAAA

>1013

CAGCGAGGCTCGCTTTTGTTGCGCTTTACTGGTTTCTTGGGGTGCAACGCTTGCTTTTTT

GCAAATGCTGACTTGTTCACGTTTTATACTTCGAACACTAGCAAGTTTGGCCACTGATTC

CGCGGCTATGTCAGCCATTTTCCAATCAATAGAACACAATCGGGGTGTTCTCCTTCATTA

ATGTAGTAATCATCTCGGTTAAAAAAAAAAA

>1014

CCCGAGCCATAGCAGACGTATGCATATTGGGTTCACTCATGTTGGGGCCATATGCCTTTG

GGAACACAATGCTTTCAAGGGATTTCCTCGCATGCATGTGAGTGCCGGGTTGTTTGCATA

GTGCAACTTTCCTCGCTTTTTTTGCACATTGTTCATGCAACAATCATTTGGTCAGGTAAA

GTAGGTACGCTTTGGGGGGGAAAAAAAAAAA

>1015

TCAATTGACCCTACCATCATGCACAGGGTATTGGTTTACATTCACCCAGGAATGACAAGT

CGCCATCATTTATTCCACAGCCTTAGGAGTGAGTTGGGACAACACAAGTGTAATGATGAC

AACTATGGCATTCACACCGACCAACATGACTCACGTGATCGTCAGAATGCAAGTCATTAA

AGTAAATGTAAAATCACTGGAAAAAAAAAAA

>1016

GCAGTCAAACTGATTCTCAGTGGCACCCAAGTTCTCAATGGCAGATATCTTGTTCCCGCG

AAGATCAAGCTGGTATTCCTTCACAGCATTCATCTCCTGCGGTGAACTGAGGATCAATTC

TGCAGTTAGACGGCCCGCGAGTCGCGGAAGCCCGAACACCGCCTGCGCCATGGCGCTCGG

GGGCTAGAAGGCTGCCACGCAAAAAAAAAAA

>1017

GCGCAGGATTGAGATTGCGTAAGAAGCCTGCCCTGTGCTGCCGTGTTTGCAAGCTGGAGG

TAGCAGGTGTAGCTGTAGAGAGTGTACCATAGAAGTGCACTGTCAACAAACTGTATAGGT

GATTATAAAAGGATGTCATGCATCTGACCCCGTAGTACATGTAAGATATTTGTAAATTCA

GAATCGAAGCAAGTTGACTTAAAAAAAAAAA

>1018

CATAGCAAATTCAAGCTACAGACTTACTTGGCTTGGATCTGGAAGTGAGGGAAGAAGCCT

GACATCGCGTCGCACTATGCTGATTGAATTATCAACACATACAAGTCTCAGAAAATGAAT

TGATTGTCAAAGTATTTGACTTCACTATGTGACTGGTAAACTGCGATAGCAATTGCCTTT

AAGTGTATCCAAGCAATCTGAAAAAAAAAAA

>1019

TTCAAGAGCCGCGCACGGTTGGTTTTTTAATACTGCTGTTGCGTTAACAGCCTGCGCGTC

TTTGCGATTGATGCTTTCGGATATTTCGGCCGCAACTGTGTATAGACAAATGGACCTCGC

GCGGTGACTTGATATGGCTGAGAGTTGCCAGAGTAGCGTTGTAGGTCAGGTTCGCGGGTC

GCTGAAGGTCGGTTACAGTTAAAAAAAAAAA

>1020

AATGAGGAGCAGGGTCGCGAAAAGATCTTCCAGGGTCTGACTCCTAACCCTGAGGATCCG

GATGATGATGAGTACTCATTTGTGCGAAAATAGATTTGAACCAAGGGATAGCTGTTACGG

TCATGACGATTCTTGCTGTCTAGCATTTGAATTCCTATACGTGGTTGTGGGAGTAATTTC

AAATATTGTGAGATGATCCCAAAAAAAAAAA

>1021

ACTCGACGATTGGTGTCAATCTCCCGAACCACAGACAACACAGGATCAAAACTCCTACAC

GGAGCGTCGGCCCCGTTTCCTAGGAGTTCAGGTCCCATACTGGGTCAACCTAGCTTTGCA

GCGCAGATTGCAGATGTGAAGTATGCGTAATGTAAGATAAGCTCAGCTCTGCAGAGTCAG

CATATATAGAGAAGTCTAGGAAAAAAAAAAA

>1022

TCAACAGGTCAGCCTCATAACCTACTTGCAAGATCTTGGGTATGTGGTGACAAAAGATAC

ACATGATACATACGCAGTCAGGGAATAATGACTACAGGTTAGGATTTTAGTTCGAGCATC

AACATTATGTTCAAGGGTCAAGTGCTTTGTGTTGCATCTATGGATCAATGTACTCAATGG

TTTGGCATTTTGGGACNNTGAAAAAAAAAAA

>1023

TTGATTGGTAGGATTCGTCACCCACCAAGTACAAGCATATAAGGACAAAATATAGACATC

CTGTGCATGTTCTCAAAAATACAAGGCGCTGCACAACCAAATTTGGCACTAGTTACCAAA

AATCCAGCAGCAAAACTATAAAAGATCCTAGTGTTCAACGCGTCCACTAGAGACCACTTG

TAAATGTCAATGTCCATCGCAAAAAAAAAAA

>1024

GCTCAACACCTGCTCTGGCTTGAGGGTCACTTCTATTTAGTGAGTGCCCACACACTGCCC

AAACAACAGCCACGCCATCTGGTTGACTGGCAGTTTTCATTTTTTTTGGGGGGGGTCTCG

GCACTGTCGTTTTGGCGACACGAAGACAATCTCTCACAACAAGGTCAGATGCACTGCAGA

TCGCTAGCTTGTAGAGACCGAAAAAAAAAAA

>1025

TGAGTTGAGTGCTTCACCAGCTTTTCCAGCATTGAAAGCTGTTGTGTGCTCTGTTTTGTT

GAGAAGGGGGGGCAAAGTGTTCTCCCATTTAGATGGCTGTACTATGGACCAGTGCGTGTT

TTACTCACCCGAATGTACATGTAAGCCGATTTTGATGACTGTTCCTGAGTCGAAGTAAAT

GATAGATTCCATTTGTGTTGAAAAAAAAAAA

>1026

ATTCTAGCTCCGTGAATGGTAGTGTGCTAGATAGATTTAAAGAAGACAGGGCAGTGTTGC

AAATGGCTGGTCACGCAGGACACAGTGAAGGCGTTCGATAGCTGTCTTGTGCATTGGGTA

GGATTTAGATTTGCTTTTACCTACAATGAGACGTGCAGGCGGGCGTAAATGGTTTAGTGT

TTGACCGCATCGCTCAGCTGAAAAAAAAAAA

>1027

TGAGAAAAACCACTGATCAGAGGCTCATATCTCAGGTGGAAGTCCCACATACCAGAGAGG

GAATCATTGTGTTCAGAAATATGATGAGTATGATCAACAATCACAAGACCATAACCTTGT

AGCTAAAACCGTACGGTTAGCTTTGCATACTTCACAAGTGAGCATGTGCACCTTTTCTCA

CATTTCGATTCACAATTCACAAAAAAAAAAA

>1028

TGGGTAGGCTCGAGCCCCGTGGCCAATATGGTTCTTCATATGATGCACGGCCATACTGAC

GGTCAAATGTTTTTCCTCCACCAGCACTTTGCTTATATGGATCCAATTCACAGGGGTTCT

GTTGCATGATGGCCGTCCATACAAATACTGGAATAACAGAATAATATCGCACAATTTGGA

AATTAAATAACTCAACACTTAAAAAAAAAAA

>1029

CTGCAACGAGGTATCGACTCTCTGGTGGCAGATTCGTGGGCGAATCGTTGCTAGAATCCA

CAAGTGTAGTTTGGAAATCAGTAGCTGTGGCGAGCACTGCTACTACTATCAGGGTGAGCC

TGGGAATGACGAGCCCACGTGCTGCCGAGTTCAGGCTGAAATCTTTGCTGAATGTCCCTC

GCCGGTTCATATCGTCCCTCAAAAAAAAAAA

>1030

TGCCCCTCAGCTAATTGCATAGGACAAAGAAGATCCATCACATGCCAAGATGTTGGTGTG

GCTGTTTGCTAAAAGAAAAAACGTCAATTATTTGAAGACGTCGCTCAGCACAATGTCGGG

GTATTGCCAATTAGCTCTGCTCTGCAACCCATCACATCACGAAATTGCAAATTCTTGCAA

GGCAATTAAATCGCAATTACAAAAAAAAAAA

>1031

TGCAGCACCACAGGTTCAGCAATGTATAGCCCGAATGATGTTGTGTGCGTGCCCAGAAAT

GACATACAATTGTACGTGCTACTTCTTGGAATCAGATGGCGATACCTCCAACACATTCTG

ACATTTGCATTTGATAAAAGACAAAACATTCGTGTTTTGCTGAGTTGTTTTGAATTGAAG

AAGATAATTCATAATTTAGTAAAAAAAAAAA

>1032

GTTGTTTTATGTGACCATTATTTTCAATCCAAGATGCCAGGGCAGACATGCTCAATATCA

AGTGTGAATAAATCCTTGGGGGTGCACCAACAAGAAGAGCTCACATGCAGGCAACCGACT

GCATATGCAACAAGCAAAGAGTTTGTTTCATATGGAGTGACACAGATCAGTAGAATGTCC

TGATGGAGATACGTTCTCGCAAAAAAAAAAA

>1033

GCTCGAGGCGCTCCAATTTACGGAGGTCACACACCCCTTCCAGCTTGACGATTTCATTGA

AGCTCGCCACCAGCACGCGGAGGTTAACCAGCGGCGCCAGGCACTCCAGCTTGCGCAGCC

CGTTGCCGTGCAGGTTCAGGTACGTCACGTTGATCAGTGTGGCCACCGAGGTTTGCTTGA

GAATCTGCTCCTCCCGACCTAAAAAAAAAAA

>1034

AGACTTTTGAGGTGACGCTGACAAACTTGGTAGAAATTTGAAGATCCTCGAGCGATCCTT

GAATGACTGAAAACCATCGCAGTAGAAATATACAGATACTTTCCCCAAGCATTGTTGGAG

CCCCATTTGGGTTTGCAGCATGTTGCATCAAACAGGAAAGAACGCTAGTTGACCGTCCCG

ATATCATTGTCCCCATGCTCAAAAAAAAAAA

>1035

TTCCCTTTTGGGGAAGGGGGGAACATCCTTTCACCCTCATTTCATGCCCCCTTGTTGAAC

AGGTCCATTATTTTTTTGGGGGGGGCCAATTAGGAAAGCAATTGTTCAGGATATTTTTTG

GGGCCCAAAAAAAAAAA

>1036

ATGAGACCTGATCAGGAGGGAACTCCTTGGGAATTCTATTGTTCTTCTCCGTGCACCCAC

AAGTTTTGAGGCCTCGGATCGGGATCTAGCAATAAGTTTCCTTGCATTTCGTGCTGGCTC

TGTGTAAACAAGCGTCTTGTCCGATTAATAGTGCTTGAAATCAGAACCACACTGCATGTT

TATTTGCAGTTTATCTATTCAAAAAAAAAAA

>1037

CGCAAGCGGTAATGCCGGCAGCCGTTGTCTTATGCAGCGGTTGCAACCCGAGAAGCGCGT

AGCTTGAATAGTTTCCACACTCGCCAGGCTCTGTGAGATGGGGCGCTTCTTCCGCCACTT

GGACAGTGCTGTCATCGCCTCCACTACGTCCGGCGTGATTCCCGCCTTGGCCCGCTTTCC

TTCTTTCGGTGCTTCCTGGCAAAAAAAAAAA

>1038

GGCCGGCCTCGTTGTTCCGTAATTTTGTTGCCCGGTGAAAATTTTTGATGCCCCCGGGCC

TTTTGCGTTGGCTGGATCAACGGGGGTCCCGGGTTGTTTTTGTTTCAAATGTTTGCAGGG

TTTCGGGGCCTTTTTGGCAAGTTTGGGGGGGTTGAAAAGTTGCTTCCCCCCCTTGTTGGG

TTCCTTTTTTCGGTTCCGGGAAAAAAAAAAA

>1039

AGAGCTTTTGTGGTCGGGTTGGCATATGGGTCTCTTCGGGGAATGGACGGAGAGCCAGGA

GCCTTCAGCGTGTCAGCGTGACCCCCACCTCCCATAACTCGACGTATGTGCGGTTGTGAT

TGGTATTGGCAGCAAAGAATCGCTTTGATCTCAAAAGATCTACCACCTCTTGCGCATGCA

CAGTGTGCGGGTTGGCCCTGAAAAAAAAAAA

>1040

AAGGTATCAAAACAAGAGCTTGGACGAGATTGGTGAATTTTGTATCCAAGTCACTTATCA

AGGTGTATCGAAGTTCGTTCGTGATTTGAGGTGTGGTGCAATTGCGTGCCTGCCACGTGG

CAGATAGCCATGATAGCCACTATAGCCGTAATAACTTTTACGTACTATATATATACCATT

AATGCAAAGCGTTCTTCAGTAAAAAAAAAAA

>1041

AAATGGCTAGGCAAGCCATACGAGTATTAGTTAGGGACAGCGTAGGACGAGCCTCAGTGC

TCTGTGCCATTAGCATAGATACGCCCCGCCTAGAAGTAGGAAGGAGCAGCGCCCGTGGCT

GGAGGGGCAAAGTCCCCTTCCATCCAATCGCGTGCAGTGCAGACGGTTCAACTTTATTAG

TATTATGTAGGAAGAATTTCAAAAAAAAAAA

>1042

GCAGGCTTGTCCCTTGCTTTTGCAGAGCGCTCCTAGAAAGATGCTCTTTGCACGAAGAGA

AACGCGCTCCTGTCGTACCTAGAAACACCTTTTGCAAACGTCATGCCTTTTCTACCGCTT

ACTCACATCAACGCGTGCATTTTGATCCAAAATGCATCACCGCTCCACTAAAACCATAGA

TGCAGCGTGCCGAAACACGCAAAAAAAAAAA

>1043

TGCCCGTGAGTACTTCCCTTACATAAACAAGGTTGACTCTTAAACAAGTTGCCCTATGAG

AGCTTGCAGCATACGTCAGACACGAAGCATTCTATTCGCGTCCGCCGGGAATTGTATACA

GTAATGAGATGCTTAGTACCATCCCATGTACTTGATGTTTAAACATCTGAGTAATCTGGG

AGCAGAATTAAGTGTTGTGTAAAAAAAAAAA

>1044

GTCTTTAGGCTACTTTAAAGACCTGCATGTGTTTATCAAAATGCGTACCCCGTTGAATGT

GAAGATTTTGTCAAATCGCTGGTGTTTTGTTGCTGTAGATGAAAGTAAAATTATGATGTA

CTAGTACGTGCATCGCTTCAGAGCTGATTTTAGTGGGCTCCTTTGATCCAGTCAAAGTGA

ATGGATTAAAGTGGTTATTCAAAAAAAAAAA

>1045

GCGAGCAGAATACGGCAGAGAAATCGATCTGGCAGGGAAAATTATCCGTGGAGGGAACTC

GATCTGGCAGTCCAAAAAAAAAAA

>1046

TGTGATTCGTGTTTTTTGGGGATGGCGGGGCATGCCCGTGTAGAGTCGTGCTCACGTGCA

GCTTCATGTGATCCCGCTGGTTTGAAATTGATGGCAGCACACGTATCAGGCACATTAGAA

ACGTTCGCTACTTTGCTTTAGAACTGTTGTATGGCAGTATATGGGTTATGAACAGCCTTA

GCAACAACCAGTACTTGTTCAAAAAAAAAAA

>1047

GACAGGTCCCGAACTTGAAGCTTGGGCATGGCAGGAGGAGCCATAACAAGTGCGGCCAAA

AATGACAGCGTCCGGTGTATCGCGCGCGCCATGCCGCTGCTGGTGTGGAGTACTCCCACA

AACAGAGTGCAAAAAACAAAAAGCAGAGGGCGTGTTCCCACACTCACGACACGCTCACCG

AAAGCATGAGGCCCGTGGCGAAAAAAAAAAA

>1048

AACGTTTTCACAAAATCAATTTCTACAGCTTCACAATGTCACTCAAACAAAAAGTATGCT

AGGTGCCTACTGGGTGATGTCAAGCAATACAAATATGAATACAGAAAACCACTAGAACTG

CGTGCCGCACCATATCAATTTTGACCCACCAGGTATGATGGTGGTTGTAATAAGGCGATT

GGTATGACCACTTGCTAATCAAAAAAAAAAA

>1049

CTATTCACTATTTTCCTTGTTTCACTTTCAAGCATCCTCATGGCCTCACGCCGGACAGGG

TCATGCTCCAATACGTAAAACGTTTCATTGTAGTTGCTTTTGAAACAGTTTATGAGACTT

AGGCTACCGTTTGGATTGAATTGTGAATTGGGACGATGTTGGCGCATTTGGTGAGATGAA

TATATTGCAGTTCTTAGCGTAAAAAAAAAAA

>1050

CAGCTTCCATTAGACTGCGCACATCACCTTTAGAGGCATCCCCTTCCACATCTTGGAAAA

GCTTGCCTAATCGCCTCAATCCTTTTGCGCGCTTGATGCTCTGATCTTTCTCAACTGAAT

GGTCAAACAACACCTGTGCATGCAACGGGAACACGAGCAGCCACACAATTCATGATCAGA

GAAAAATGATTTAATATCGTAAAAAAAAAAA

>1051

TTCTGCCAGCATCTTCTCCTCAAACAGATGGAACAGGGGGGAAGTACTGGCAGACTTTGG

CACCACCACCAAAGCATTTTCAGAAGATCTCTTTTTCCACCAACCTATGGCTGCCAGAGA

TCCGACAACCGCAGAGCACCCAAGAACCATATTATCTTTATTTTCTTTCTCCGCGACCTT

CAGGGAGGCTGGTCTGTGAGAAAAAAAAAAA

>1052

GGAAAGAAACGCCTACAACAGGTTTTGATGTTGGTATGTACCGTGCTTCTAGTTCTTTTG

GCTGGAGGCATGACTCTGGTATTCGCCAACGACTTTGTAGCTGCGTTCATTACAAAGGCG

ATTGGGTAGACATTCTGCACTGACCTTCTTTCACGCACACTGTGTGTATGAAATACGAAC

GTGCATTCCATATCGTTGCTAAAAAAAAAAA

>1053

TCCGACGGTGTGCAAGAGTTGAGCGCTTGACACTCGATAGGCAAGAGTGCTCAGTCTTGC

AAAAACTCGGCTTTTCAGTACCAACCCAATGTTGACTGGTAATAAGGAGGTACTCGCGGG

AGGCACAAGACAAAGCAAAACGGGTCTGTGGCCGGTATGTGTGTGGGTTTCAGGGTGGCA

CTCAGGGAACCTCTTAGCGTAAAAAAAAAAA

>1054

AGATCGAGACTGATCTGTGGTAATAGCAATCGGCCCCTAGATCCAGATATGCAGCATTAC

AGTATAGAATACGAATAGACAGTCACTAGATCAGTTATGGACAAATGATCTAGACAGCCG

CCACTATATAAGGCTGGACCGGACCTATCTTACCCACTCTAAGTGTATACTGTAACAGCC

CATGTGTCTATTCTGATATTAAAAAAAAAAA

>1055

CCATAGTTTAGAGATCAGGAGTGCTGTGTAATGTCACGCTATTATCTGGCAACCCGCTAG

AATTTTTCATACATCAAAATTAATGCATCGCTCCTCTGAAATTATGTCTGGACCACCAAT

TTTAGTGCAGAACGCAGCAGGGGCGACGCCGTTGTTAAGTTTCGATTAAAGGTCTATGAA

CAAAGGGTCACGTTCATTTCAAAAAAAAAAA

>1056

AGCTCGCCATCCCAACCAGGACAGTATACTGATCCATATGAGACAAACTCTTTGTGTGTT

TCACATGCAGTCGGTTGTGGGCGCGTGAGCTCTGCTCGTTGGTGCACCTCCGAGGATTTG

TTCACAGTCGTCATTGAGCATGTCTGCCCCAGCATCTCGGATTTGAAAACTATGGTCACA

CTTTGAGGAACGCACATCAGAAAAAAAAAAA

>1057

CCACCAAGAAGGTAACCATTGTGGACTCCGGCGAACTCTCTCTGTGAATTGACAATACAG

ATCACACACGGGCCATAGGATTCAAGGTTATGATACGTACAGTACTGTTGATATGATACA

TTTGCAGGAGAAACACCTGATTATTCCTGTGGTAATGGTATTCTGGTGGGTCTCCTATGG

GTGTTCCACTACATCTGGTGAAAAAAAAAAA

>1058

CACTTGACCGATTCTGTGCACCCAATTCTACTTGAAGAAGGAAAGAACAGCCGGAAAAAT

ATTTGCCAACATTAATTACAATGCCGCCGAAATACCGACATTCAAGTAGAGGACACTGGA

ACACATACGAAACAGTTCAAGGGTCCTTGGGAAGGACTTCATATTATTTTATATAGGGCT

TGTAGACCCACTGAGATTTCAAAAAAAAAAA

>1059

AAGGCAAAGGATTGCAGCCAGTGGCATACTGCTCGAGGAAGTTAATCCCAGCAGAAACTC

GATATGCTGTGCATGAACAGGAATTGTTAGCGATACTGTTCTGTTGTTTGAAATGGCGAC

ATTATTTGGTAGGGAGCAGATTCTGCGTGAAGACAGATCATGCCCCATTAAAGTATTTGT

ATACTCAACCAAATCTGAGCAAAAAAAAAAA

>1060

TTTGCTAAGCATCCAGAGCATCATGGCCGGTTAGGGTCTCTTGTTCAATGAAGGAGGTTT

GGGGCCCTCGGGGAAAAAAAAAAA

>1061

CAACTGAAGATGGTCGCTCCTACACACACTATGCTGAAGCGCACCGAGCAGCTGGAAGAA

GTGTTTGACCGCGCAGCACAAGCATTTAGTTTGTAGTTGTGCTAGGCTGGTTTCTGGGAA

CGTTGATGCATTTTTGCACTCCAGGATGACCCAAGTGAAAATATATGACTTTATTTTGGG

GCTGAGCTTCCTCTTGTGTGAAAAAAAAAAA

>1062

TAGCGGCTTCAGTCTATCTTATGGCTCAATGTCGGATTCTAGGACAGAAAAGCTCGCTGG

GAAACAAGCCGCTGTTGGTTGAAGTGCAAACAGCAGTCGCACGTCGTTGCACCCACCAGC

CTGGGATCTTGACCTTTGTCACAATATATATACATACATTCAGCATGGCGTATCATTTTA

TGGGTTGTTATTACTGACACAAAAAAAAAAA

>1063

TTAGTGACTGCGGTTGATATTCACTACGCCAAGTCAGCTGGACAGCGTCGTTTCCTGGGT

AGTGTGAATGAAAAAAAATCAAAATCTGGATCATCGTTTATGCATTGCAGACATTTAGGG

AGGTGGGTAGTCATGGCACACAACCAGGCCACATACTTTGGTTCTGTCGCAGCATCATGT

TTGGATGAAACCCCTGCATGAAAAAAAAAAA

>1064

AAGGCGGGAGTCACAGGAATTAGGAACATGTTAGCCTTAAGGCACCTGTGTGTGGGTCTA

ACGCCTCTTTGTGCAAAGTGAAAAAAAAAAA

>1065

ATGTCGCCCAAGCTTGAAGGGACGTTGCCCGGTTTCCCCCTTTTCGGTGAACCCCTTCCC

TTTGAACCCCCTTTTGGGAACTCCTCCTTGGAAGGGGGGGGGGCCCCCTGATTCCCGGGC

GGTGTGGATTTTTTTGAAGAAAGTGAAACTACCCCGGGTTTTTTTTTCTGTACGTTTTGT

TTCGCTTTTTTTCGGGCGGTAAAAAAAAAAA

>1066

ACTGACGCAGTCAATACTGTACTGCGGGTTGACCAGATCATCATGGCAAAGCAAGCGGGC

GGGCCCAAGGCAGACCGAGGCCCTGGCAATGATGAAGATTAGATTCTGGGTGAGATGATC

CATAGCTATGGTCCAACAAGCAAGAACAAATTGCAGATAGTTTTATATTTTACGTCGACC

CTGATGTGTTCATGGAATGCAAAAAAAAAAA

>1067

ACATCAAAATGACCCAGGATTGAGGATATTTCGCAGCAATCTTTACAACCGGCTGCGTAG

TTGCAAACCACCGGGAATACAATTAATTGTTTTCTGGCAGTATCAAAGCACTTCATAGTT

ATATCAGTTGTTTCTAGTCACAAGTCGTAGATATAAACAAGTACTATGACTAATGAATCA

GAATAGCCACATGCATGTTCAAAAAAAAAAA

>1068

GCGTACTTGGAACTGACGTCGATGTTTCAAGCCAGCACTACACCTCTTGGAACGCAACAC

ATACCCGATGTGTTGTTTCGCTTGAAACGGTGTGGTAATTTTGCTCATCGCGCAAACAAA

ACACATTCGCGCAGGTTGTTGCAGTTGTTCCGCCCAAACTGCCAAGATTCAACCTGAAGT

CCACACGTCACCTGTGCTTGAAAAAAAAAAA

>1069

TACAAGGCTCAGTTGGTATTGAATGATCATACTTACCAAACACAATTTGCATTCGTTTAA

TAGCCCTCAATCAAATGTGGGCCTTGTTCCATCTATATCTAACCTATATTCAAGCTAGAG

CAAAAGTACTCTGTTGTGGTCATCAACACAGACCAATTCAATGTAGACCCCACCGTGGAA

CATTGAAAATATGCAATGGGAAAAAAAAAAA

>1070

CAGCTGTCGTGGCCAGAGTCCCGGTTTGGCCTGCCAACCGTTTTCGGCTGGCTATTCGCG

CAATCATCTTTGGGCATACTTGGGTCAACCACGCTTCCTGTCAGTATAGTTGCCTCATCT

GTCGTCGCCACCTCCCTACTTTGTTGAAACATTCATCTAGAACATGATCATAGTTTGATG

ATTATTGCCACACTTTTTGGAAAAAAAAAAA

>1071

AACTTATTCAGTCTTTAACAGGGTCTTATCTGTTGTATCATTGGGGCACGTATGGTGGCT

TCCATCCATCAAAAAAAAAAA

>1072

CCAGTCGGTATTCATTTGAGACCCAATACGGGAGTTGTTTGATAGGCACACCAACAGGTG

TGACCCAACCCCAAGACGGAATTCCAAATGTGACACCAACTAGGTAGGGCCGGGGATAGG

GCGTTCGGCCCTTTGCACTCAACAACTGGTTAGGCACCAGTACCAAATAATTGCAGTAAT

AATAGCATAGAATCTCGCTTAAAAAAAAAAA

>1073

AAACCCAGGGGTCCTTCGGAGGTTTTTTTTTGGGGTAATCCAGATTTTCCCCCGGGTCCC

GGTTTGGCCGGTGTTCCAAAAAAAAAAA

>1074

ACGCACGCAAAGTATTCAGTGGTAGTATGCAACAGTCTGTCAAATGTATTACCATGCCGG

AGAGGAGATGCACGTGCATACAGCAAAACGTGCACCCCTGTTAGGCTGCGGAGACAACAA

GCCCGTTGCGTATACCCCCTTGGATCATGTAGACCAGAGCAAATGACATAATCACCAGTG

TGAAAGGCATAGAAATTTCCAAAAAAAAAAA

>1075

AGCTCAATTGTTCCATGTGGCAGTGCGGGACTAACATGAACACAACGGACGAGGATGTGT

TGACGTTGATAGTGTATGAGAACACACAACATAATCCATATACCATATAGGAACGGAATC

CCATATGTGACCAGCCTGTGCGTTCAACTGGTTTGCATGGTTGTATGAGCAAAGGTGTAC

AGGCATGAACAGCACGAGCCAAAAAAAAAAA

>1076

CCTGCTCTGGTTTCAGCCTGCATTTGTGTTGCATGCAGCACCCATGTCCACCACTTCAAG

GCACAAGAAATCATTGGTGACAGAGACATAGTCGGTCTGTGGAGGCCTACTAAGTCGTGA

CAATGGTACAGAGATTATATTTCCATTATAAGCTTGTGGACTCTAGATGCTGATGTGGTA

AGACTGTCCTTGATTCACTGAAAAAAAAAAA

>1077

ATACAGAAGCCTCGCCAACCTGAAATCGCCAAATATGCATGCATTAGACCATGAATTTTG

CAGAACGTTGAGTGCATAGCCAATAGCTTGTTGAGAGATACTGGCATTGGTGACACCAGA

AAGCATTCTGCCCTCTGGGTGAGTGGCTTCTATAAACACATGAACTTTGTCATATTTTCA

CTGTTTACAACTATTTCGCCAAAAAAAAAAA

>1078

GGACGGCAACACATTTAGGATGATTGCAGAGACAGTTTTAGGTACTTGAGGGTGGAAACA

ACTGATACCACAACTGCATTCATTTCCTGCTTGGAAGTCAATAGCTAAAAAGTTCATATT

GGTAGCACAGAAGTTCGCCTTCAAATATTGTTGGACATTCGGCCCTAGTTAATCAGTATC

CTTGTAGCCTGTCAGAAGGGAAAAAAAAAAA

>1079

GACGATATTGGTGACAGAGTGGACACCACATGCACAAGACAGACACTTGAGTGTCTAGTT

AATTCTTGTGAATTATCAAAGCAATATCATATGTCAGATGGTTGGACAAATGTCTAATGA

GCATGATGCCAGGAACATCTGAATGCACTGCTCCCATCACAAGATAAGTGATGTGATGTT

TAAAGCTCATTTTGTGGATCAAAAAAAAAAA

>1080

AAGGGGGACACCATAATCCATGGGGATCATTTATGCATTTTTTATGCAGGGGCCCTTGGA

TTTTTTCCCTTAGAATTTTAGGCCCCCCCCCCAGGGGGGGGACTGTGGGGGGTAATTTTT

TTTATGATTTATTGGGTGTTCATAGGTAATATTAGTATATTTGGTAATCCCCCCCCGATA

GTCCAATAGAGTTTTGTTCCAAAAAAAAAAA

>1081

TCGATTGAGCGTCAGCGATGAACACGCTGTAGATTGCTAGCAGACTGTACGGTACAGAGT

TCATTGTACCGTTCACACATATGTATGCACAAAATCACCAACTTGATTCGTATGGTCAGC

TTGACGGTGACTTGTGTGTTCGATTCTTGACATTTGCTTTGATGTGGCCTTATTGAATGT

ANGATCATGACCCTTACCCCAAAAAAAAAAA

>1082

CTCATCACATTGTAGCGGTCAATTAAACAATAAGTATGCATCATATGTTCTTTTGGAAAC

ATAACATAGCACCACGTCACAGGGGTTAGGCACATTTGGCAGTGGGTCAGTGAGGTAAAG

CTTGTTGACTTGAATACAATAATACGTCAATGAACCTAGCAGAATGACGCTGTTCAAATA

TAGATGACACCTCCCATGTCAAAAAAAAAAA

>1083

ATCATCTCTTGAGTACTTGAATGATGGAGATTTAACCTAGATACATATTGCTGTGACACC

TTATATGATCGCTTGCTTCTTACTGGCAAGGAAGAAATTCAAACTGTAACTTTGAACGAG

GCAGCTTGATTCAAATGCCATCCCTTTGGGAAGCTCCAATAAGGTGAAGCGTTCACTTGA

TTCAGGTTGAAAACATACTGAAAAAAAAAAA

>1084

GCGTCAGCGCGCAGAGAAACATGTCAATCAATAACAACCAGCGAATGACTAGAAGAGCCT

CCCTCGTTTTCAATACGTTTGCGTGTTTTAGAGACAAATGAGTTTTGGTATATTGTACCG

ATTTGTGTTATTGCAGTTAGCTCTAGCATCGCACATTGCTTCTGTACTGCTGTATTTATT

GAATTTAGAGCTAGCGCCTCAAAAAAAAAAA

>1085

GCGTGCACCTGAAGATTGGAACGGTTTACGCGAAGAGGAGCACGGGTACAAGCAGAAAGC

GACGTACTGCTCCGGACCGCCCGGGGACCAGCGTTGCGACGACCGACTGTACGCACGACC

GCAGGGGCGACTGAGCGCATGGTCACTTTGGAAGCCGCCATGGTCTGCATCCTGTTCCTG

ACAGACACATTGATATGGATAAAAAAAAAAA

>1086

AGATGCATGATGTAAGAGCATGCGTGACAGAAACCTGTGGTTGTTCTACAAATGTCATAG

GATACGGGTGTTCGAAGCAAATCACTTATAATTGAATGGGAGCTGATGGGTGTTGTTGAG

TGGATTGTGGATTCTGTGTGCCCAGATTTGCCCGCTAACTACTCATCAGAGAACAGACGT

TTGAGCGAAGTGGAAACTACAAAAAAAAAAA

>1087

TGGCGATTTAAGACAGGAGCAAACAAGGATTAGCGAATACAAGGGAATAACCACATTGCC

CAGTTGAAAGCAGTGTGATTAACAGCAATACGCCATGCTTGATCATATTGGGATGGTTTT

TCAATAGGTGAACATCGCCAATCAATAGCAAGACGCTGCCGGGTGGAGAATCGTCGGCAC

GTTCACAAACGAAGGCTTTCAAAAAAAAAAA

>1088

TGATAGCACCCTTGTGTGACAAACAACTGGAGATGAAGTATTGCAATACCAAGGTCATTT

CCTTTTCCGGACACGTCGTCAGTTTTGGCGAACAAGACGAAATATGGGATTCTAACAAGC

CAAATGCACAGGTTTGCACGTCGCTGGTGGTTGAGATCTGATTTCATCTACGATGTCAGC

CTTGTCGTTAGAACACAACCAAAAAAAAAAA

>1089

GTCATCCATGAGGAACCACCTAATCATGGTGAATGTTGAATGTCAGAAAATAGTGGAAGT

TCCGAGTGAGAAAAAAATTTCCTTGAAGAGTCTTCTGAAACAATTCAAACACGGGTTCTT

GAACCCTCCTAGAATTCAAACAACAAATGTTATTTGCCTGGGAGTGCGAGAACTTGACAG

CAGCTGGAGCATCAGAAAGTAAAAAAAAAAA

>1090

GTGGCTGATCAAACAATCGTCGAGTTTGAGTGACAATGTTTGAAGACGGGTCTTGTAGCG

CATGTACGAAATTCACGTCATGAATCCAGTCGTAGCAAACCTGCATTGGCATTCAGATAT

AGCAAGGAATGCATAGAGACACCGTATGCATGGATTAATAGACAGTAGCTTTCAGAAATG

TATGATACATGAAGCGCGCCAAAAAAAAAAA

>1091

CTATGTCAAGCTGTGAGGTATGGAATTCCATGCACGCTGGAATGTTGAGTTCATGCATTG

TCCATGAACCTGAAACATAGATTTACGTAAGCACATTTGAGCTTTCGAAATGTAGCAAAG

TCATCTGATGGTCAGCTGCAAAAAACACCACTAAGAGTAGAAAAATGTAAATGTATTTTT

GTTGTATGCCTACAGTACTCAAAAAAAAAAA

>1092

GTGATCACTGAAGGTGCAGACGATAAATGAAGTTCGCCAAGTATCCTGCTGGCTTTGCGG

CAGATACGATGGCCTGAAAGGAGCCATTGGAGGATGGATGGACCAGGCTGAGAACTAACC

TGCCGATGTTTTCTTGCTTGTATTTATTTACAATAACAATTCTATGTACCATGTTCGTTG

AAATTGAATGAGGTCCACTCAAAAAAAAAAA

>1093

GGGTAAACCCCGGCCCCCCTTTTTTTTGGTTTTCCGGGGGGTTTTTCCTTTTTTTTCCCC

CTTTTTTGTTGGGGGTTTTTTCCGGGGTTTTTTTTTGGGGAGCCCCCCCCCCCCTTTTGG

GGGGGGGGGGGGGTTTTTTTTGGGGGGCCCCCGGGGGGGGGTTTTTTTTTTTTTTTTTTT

GGGGCCCCCTTTGGGGTTTTAAAAAAAAAAA

>1094

GTTGCAGCTGGAGCAAGCATTTTGCGTTTTGTCGCGTTCCACTTCGAGTTCAGCTTCCTA

AGGGAATCGAGCAGTGGGTCAAAGGAACCTTGGGGAAGGTTAAAATAGTATGACTATGCT

ATGAGTATACTAGCATGGATATTTGGTGGTGCATCATCTGAACACAATATTTGCGAATTG

AAATCATAACCGATCGCTGTAAAAAAAAAAA

>1095

TAAGCATCTGATGCGATCTCACCCGAATGGCTGGCTGCAGCGCTTCACCTACTGGTAGAG

ACACATTCTGATTGCGACGCACACATATAAACAAAAAGTATACGAAAGGCACATGAGGGC

CTGTGAATTTGTTCTTGAAGCCCATTTACACTTGAACTTCTATTATGATTATGTGTGTAG

TTTCCATTGTCTGGAAGTGCAAAAAAAAAAA

>1096

ACAAGGAACAAGGAACAAAGAACGAGGAACAAGGAACAAGGAACAAGGAACCACGAACAA

CGACAAACCACAAAGACAAACCACGAACCACGAACGACGACGAACGACGACGAACGACGA

AGAACGAACGACGACGAAGAACGAAGACGAAGAACAAACAACGAACGACGACGAAGAACA

AACAACGAACGACGACGAAGAAAAAAAAAAA

>1097

CAACCGAGCCTTGGCTAGAATCAGCACGCACTCCCATGCTGGTTTTCCGCATCTGGAGGG

GGCGCTTGAGTTGAGCGGCAGGAACGCGGCCAAAACCCTGTTTGTTCCCTGCAGTCGCAT

TGTATCCGAGTCTCGATAGGGACAGTCCAAATTTGCAATCTTTAACCGCGAAACTGTTTT

GTGAGGTTTTTGATCTTGATAAAAAAAAAAA

>1098

TACCACATTCGTGCAGACGTTGGCAAAGCCACCAATGAAATCATGCCACCTGTGGCAAGT

CCAAGACCCCAGATCGGGTTCTATTGTAGGTATATGCTGCTGGCTAGCCTCCAGGGACAT

TTGTTAGGAACACACAGTGCATGTTCACGTACTAACGCCGTCAGTCGGTGAGAAGACAAA

TCCTCAAACCTCCCGCCATCAAAAAAAAAAA

>1099

CCCGGCTTGTAAGGGACTTCAAGAACTTCACTTCACAACGCACAGGCTCAGCCACTCTGC

GCAAGACGGTGGCGGTGCCAGTGTGACGTCGACACGGGCTCCATAACATGTGATGAACGC

TGTATGATAGGTCAGAACAGCCGCATGCGTGTGCTTGTGTACACTTTAAGTGAGGAATGA

TAGGTCCAGTCCATCCTTGTAAAAAAAAAAA

>1100

CGTAGCTTGCTTTGTCCGGCGTACAGCGCAGCGACTCGAAGGCACTGTAGCTGTGGCAAC

GTCGCCGGGCGCTCCGAGACGACGGTCCACACCGGCATACGCACAAAGTGCACCAGCTAG

TTGCGACACCCGACAGTCTTCATGTGTGTTGCTAGTCCCATGCGTACAGCCACTCGAGCC

GTGCCTGCGCAGAAACTGTGAAAAAAAAAAA

>1101

CGTCAACCGGACTTTGTACAAACCTGTGAATCGGGATGACGCACCTTCCTACGGTGCCAC

CAGCGCAACAACCACTGGGGGGTTTCGGTACTGGGTGACCAGAAGCTATAGGCTAGATTT

AGACAAAAACACCTGTGCACGTAATGTGCAGCATGTGAATGTTTTGTTGTAAACCATGGA

CTCTGCCCAACGTGTGCGATAAAAAAAAAAA

>1102

ACGAAAATCAAATGTTGCACCTATACATACAAGTTGTCAGTGCACGAGATGAAAGGAACA

TCAATCCTATATTTACAAGAAACACTGGCTGCACTGGCCTAGACCCTGATACAAGCGAAT

CACTCATTGAACAATAAACGAGTTGTTAAACAAGTGCATGGCACATACACTCTCTCGTTG

ACGTAAAAGAAAGTAAGAGGAAAAAAAAAAA

>1103

ATCATGCTCTGAGCATATCACATGCCTCAACTTGAGAACACACACCTGGAACTTTCGTTG

AGGCGTTATTCCCGATTTCAGAAGCTCGTCAAGTGCAATGAACTTTGACGTGCGCTTAGA

TTTGGACGTGTGAATGAAATGTACTCTTCGGCTTTAGTTTCTCGTAATCTGTCAACATAA

ATTTCTCTCATAATACATGCAAAAAAAAAAA

>1104

ATCTGACAACTATGTCTGCGTGTTTAAGAGGTGCTCTTGTTTTCCGCTGGATCCAGCCTT

TCTTCCTACATCTCAAGTGGTTCAAGTGGAGTGTGTTTGCGTTATTGTAATCGCCTCGCT

AAATTGTGTGTGGAGGGTGCGCTTGTTTCAGGACACCGTGATGTATTCGCTGATAGACAA

GGAACCTAAAGTGATACTGCAAAAAAAAAAA

>1105

ACATCCCCTGTGGGGCAACCTCCCCATCGTTGTTTTGGGTTTTGCAGGCATCAGTGCACT

GCAGAGGTGATGAAAACCAATTGTGCTTTTGTTGGTCCTTCCCGTATGTAGATGCTTTAA

AGGACAGTTGCTTACTAGGAAGCATTTTTTGGAAGTATTTCCGTACCAGCCGCGTTGTGG

AAAATAGATGCTGGGGGTGGAAAAAAAAAAA

>1106

TTCTGTTGCTTCAAAAGCTCAAGACCATGTCGGGTCACCTATATGGTAGGGGGTCGCCTC

AATTCCCAGAATTGCGAGAGTTCAAGCCCTGGCGTGCGTAGGTCCTGCCTCGGCCTTTCA

AGATATCACAAGGCCATGCTGGCTGGCGGGTCATGTTCGATTAGCGAGCGATCATACCAA

TCGCCCAATCCATCACCACGAAAAAAAAAAA

>1107

GCATCCACGTGCAAGGCCCATGCGTGCCTGATCAATTGACTGACTTAAACTCGAATCGTT

TGATGGCGGGGTTCGCTGCTTTGGCATCAGTGCCTGACCCATTGCAATGGGACCCATCCC

ATTGGTATTTGCATTTATGCTTGCTAGGGGTGATATCATGTGCCTAACACCGCTGCTATT

GCGACCCTTTTTCTCACTGTAAAAAAAAAAA

>1108

GCACAATTCCTGGTTAGCTTACCTCTTTCCGTCAACCTCAGGTATGTTTTGTGCTGATAG

ACTAGGAACCTGCACGATAGCATGTGCGAATTGTCTTTCCAATGCTTGGCTATTGGTTGA

CAGTAAAGCCGCAACATTTAGTGTAAAGTTTGGATATTCAGAGTATATATGGTCAGTCAA

AGCATGAAGAAATTTGTCGTAAAAAAAAAAA

>1109

ACTGTGTCTTTAACACGGATTGTGCATTTGAGTGCATAGTGTCCAGTTTGCGAGGACAAG

TTGGATAGGAAGAAATTCGATGTGAAGAGAGTCAATCAAGTCTGATGGAAAATGATTGCT

GTGAACGTGAGATCAGTGTGGACATTTGTCAGATGTGAAGGTAAAACATGTAATTTTGTA

TGTAACATTCATTGACACTGAAAAAAAAAAA

>1110

AGAACGGCTGGAGTATGAGAGCAAGAATTTGCGTGCACGGCTTGCGGAGTACCGATAGCT

GTTTCACGACTGCAGCAGGCGGGGCTGGGGTCAAGGATCACAGACTGCTAGATTTTTTTG

ACATGTTTTTTGCAGAAGAAGCCTTGGTTGGTGTTTGTACATATTGAGCACATTGAGTTG

TAAATGAGCATCGTGTAAGCAAAAAAAAAAA

>1111

CCAGGAGGCCAACAAGACAGGCCACCCCTGGCTATGGTGGGACTACGTGTCTCGCTTCAG

CACCTCCTGCAAGATGTCCGCCACGCGCTTTAACAAGGACTGCGCCGAGCCCATCATCGC

GCAGATCGGTGTGTCCTCCCTCCTCCCCTGGGTGGACTCGAGCCTGGGAAGACGACGAAC

GATCAGATCGTCTGTTCGTGAAAAAAAAAAA

>1112

TCCGTAGAGCATCGCGATGCCTTTGCAGAATAAATTGAAACACACGAAAAACAAGTCTTT

GGCGTTGGCTGAATCACATAACTGAATGATGACCTTGTGTGGTAAATCTTGTAAAAAATT

AGTTCCGAAACGCGTGTTTCCATCTTCAAAGACATGATCTCTTTTAAAAATATATAGTAA

TGGATTTTCCGACACTATACAAAAAAAAAAA

>1113

TCGAAGTCATCAAATTTCGTCACTTGAAACACGTGATTCTTGTCAAGGCGGTAGCCTTGT

GTATGCTCCAATGCAGCGTCCGCCATCGTTTCATCCATGAAATCCACAAATGCATAACCC

TTTGTCTTCTGTGACTCCTTATCCATGGGCAGATATAATCCACCCTCTCTAATTTCCCCA

ATCTGGCTTAAGACTTTGTGAAAAAAAAAAA

>1114

ACGGGCCGTCAGTTGTGCATGGACATGCAGTGATCAGCAGATGCTTATGACAGGAAATCA

CGCCTCTTTGGATTTCAACGCGTCACGCAGATTCTGAGGCTTTCCTTTTCAAGTCATGGA

ATTTTGTATGCAACTCGGTTGGTATGACTTTACACACATAGGGTATGTTTAATGGGGTAC

GATTCCTACCACAACAGACTAAAAAAAAAAA

>1115

TTGTGTCGTGAAGTTGTGCTGGATCAATGTGCGGGTTGTACTTGGTACGAAACTGCGACA

ACTGCGCTGAGGCCTTTTCCGCCGCTTCTTGTTTTCGACGACGTTTCTCTGCCCATTCAG

AGAGGTCCCCAGACATCGTCAAGCTGAGTTTCCGGTTTCCTGCGCAGTCAAGGTCAAACG

GGAGAAGGGCGCTCGAAGAGAAAAAAAAAAA

>1116

GGTCGACGCTGAAAATTATTGTATGACAGTGAGTGTCTACTGTCGTTTATAGACGCAACT

GCAAATGTGCGAACACGTACCTCACATGAGGATACCACGTCAATTATGAAAGTGTACATG

GGAATTGACGAGCTGTACTTGATGAGTATGTAAGAGTCCAGACCTTTGTAGCTCTGCAAT

GATTGAGTATACTCTTTTCCAAAAAAAAAAA

>1117

GAGATTTCTCCTGAGATGTTCATTTGCGTGTCGAATTTTGATGAGGGCTCGTTGATGTTG

GGGCAACCGAGATCAGCACATGCTTATACTTTCGTAAGGTTTGGTAAAGGTGTTGTAGTT

AATACCTTGATAATCTTGGAGCCCAAGCTTTTGAAGACAACTTTTAAGTAAAAAGAATTT

TAAACTAACGGTTCCAATTCAAAAAAAAAAA

>1118

TGTGGTTGCTCACATTCTTGTCAGTCTGGCCACCGGTTGCATATTCCACAGGGAATGAGC

TACTGAAGTCTGTCCTTGTATGAATAGATAACTCTTAGAGGTGAACTAGATAACTCAATA

GAGGTGATATACTAGGGTAACCTTTAGTGTTCCACAGAAGCTTGTAAACTTACTGTATTT

TTACAACTGTAACCTATTTCAAAAAAAAAAA

>1119

ACAAATCCATGAAAACACGCCAACGCTGGTGAGCAAATCACCAGGCCAACCGCTCCACCG

TGTCCGCCATCGCACAAGCCGCTTGATGTAAGGGCTCAGGGTGTTGGGTCTAGGGTAAAA

TTAAATCCCATCAACCAGACAACGTATTAACTCTCCTCCGCGTCACCTCAGCTGTTCGTC

GCCCGCCATCTACCGCCGCGAAAAAAAAAAA

>1120

CCAAAGTGCGTGAAACGTGCGCATTGTATCAACGTATTGAGCACATGAGGGACGCCTTGC

ATGGTATATGCGCATAGCCGAATGCATTGGTCGTTTGTTGTGAATGCAGTCACATGACTT

ATGATGATATGGGATATAAGTGGGCTTACATGCAAAGGCATAGGCTCAAGTGATCATTCA

TGTTATGCTACGCCCTGTATAAAAAAAAAAA

>1121

AGAAGTAGAGAACAGTATCATCATCTGTGCGCGGAAATGATGGTTTGGTTGGATGATTTT

CATTGATACACGAGCTGCAATCCCACTACGAAATTTGAAGCGAAGCTGGGACGATGCTCA

TGTTATTGATATTTGTTGCTAGACATCAGTAGTCAAATGCAAAGAACAATTGTTGGCGTA

AGTTGAAAGCATTAGACCCTAAAAAAAAAAA

>1122

CTTAGGAAGCTGCGACATCGATTTGTTTTTACATCTTTTTTGTGTACTCCACTCTTAGCA

GCTTGGCCAGCCCACACAATGTGTCTGATTCGTGGGACAGGAACTCGGTGAGCAGTGCAC

TTGAACAGCACATGTTCGGAACCGTTGTGGGCTGGCCAAGTTGCTAAGAGTGGAGTCCCC

AAAAAAAAAAA

>1123

TGGCAATACACAAGAGCGTTACGCAAGGGGTGGACCCGAATCAGCACTTCACTGTACGGC

ATGATAAACTGGCGCGCGAGCGCGTGATGCGCATAGCTCATAGTAATGGACCGGAAGGTC

GGACATCATACTTGCTTCCTTAGTTCACATGTTGGCACGAGTATAAAGTGTTCACGTAAT

TTTGTCAAATAGACTTGAGGAAAAAAAAAAA

>1124

TTGGAAAATTCTCCCGAGAGTCTTTCCCAGATGTCGTCATGCGGATTTGCGTGGATTGAC

CCTTTAGAATCTGAGCGATGACTATGCACATGACAGATTATATACACCTTTAGGATTTTG

GGTAATGTGACAAAGTTTTTGGGCGCATGAGTGCGAGTATCCGTGCCCATGCATTTGATA

CGGACGACAATGTCCCGCGCAAAAAAAAAAA

>1125

CGACTGTTGCAACGGAATGCAAAATCGACCATTCCGATTATTTGCAATCAGACATGTCCT

GGAGCATAGCATTATTTCGTGTAAACACAATGCAGACTTCGGTCGGGTGACGGAGCCCAA

CGCTAAACATGAGAACGCTAGAAGGTGAACTGTGATTCTAGAAGGATAACTATTCCACGA

ACACAGGATCTCTCTCAAGTAAAAAAAAAAA

>1126

ACCCTCAACCTCAACAAGGAGAGCGCATCGTTCACGGTGTCCGCAATTCAAAGCGGGGAG

GGAATTCGTCAGACCATGCATGGTCATTATCTGCTCCCCCGCGTTCTCATTCAAGTGGAC

CTTTGTCGGTTGCTGTAACAGGGCGCATTCATGTATGATTATAATTGTTGTGTGAAACGG

GCTGCCTAGTCTGTGTCTGCAAAAAAAAAAA

>1127

AAATCAACTATTTGGTTGTTTCATCTGCAGTCGGCACGTGAGCTCTGCTTGTTGGTGCAC

CTCCGAGGATTTTGTCACAATTGTCATTCAACATGTCTGCCCCAGTATCTTGGATTGATA

AAAGAAATGGTCTCATCAAGAGAAACGCACATAAAAATCATGAGAACACATTAGACTGTC

AACTCCTTCAGCTTATACATAAAAAAAAAAA

>1128

CACCAACTGGTGACCCTTATTGGGGCAAGGGCCCGCAGCGACTAACATAAAGCAACGAAC

GGTTCAGGGCGAGGCGGAATCTTGACCTCATTGGCCATGTTCCCGTCTCATTTCGTGCTG

AGGGTATCTTTTTCTTGTTTTTGTATTCGCAAGGGGGGATCTGTGACCAATCAACGTCGC

GTTTCCCCCCTTGCGATTCCAAAAAAAAAAA

>1129

CTTTCCCCCCGAAATCAGGGTTTTCCTTTTTTTTTTTGTTGGATACTTTTCCCCGCCGCC

CCAGATGTGAAACGAATCAAATTTTGATGGGGGATTTCCGCCTGGGTCCAAAATTTAAAG

AAATGGGGGGGTTTGGGATCAAATCCCCCCAAGGGGGCTTTTTGTTTCGGGGCTTGGGGG

GCTCGCAACGTGCCCTTGGGAAAAAAAAAAA

>1130

AGAGGCGAAGCGTGAGCAGGAGGAGCTCGAACGCAAGAAAAGCGATGAAGAGCGTAAAGC

ACTAGAGGAGGAAGAAGCATCGGATTCCGATGCTGAAGATGATGAATCATCTGAGACTGA

AGGCGAGGATCACGACGAACTGTAAGACGAAATGATCACAGACAAAGCTTCATTGTAAAC

AACTTAGACGGACTTACTTGAAAAAAAAAAA

>1131

CTTGGAGAGCAGCTAGTAACCGAAGAACCCAAGGAGGAACCTAAGGAAGAGCCCAAGACG

GCGGAGAGTGAGATTAAACTTTCTCCTGAGGGATGAAAAAGAGGTTGGCAGCCAAGGCTG

CAAAGAGCAAGTCGCGCCCCTAAAAGCGCAGCCGCCAATGCAGCGAAGGAACTAGCGGCT

AAGGAGGCAAAAGCACGAGCAAAAAAAAAAA

>1132

GGTGCTACGTTTGTGGGAGGACTATCAGCACGACCAGGATCTCTCAAGCTTCAGTGAGTG

TATCGATTGAATGCAAGACTCTGATGCCTTGTATTTGACTCCAGAATACGTATTAAACCT

GCGCCTGATCACGCGTTTCCAGTTTTTGGTAGCTGACAACCCGTCCCCGCCATTCAGCTG

AACACGAACTGCGCCCTCCGAAAAAAAAAAA

>1133

AGAGGTCGTGGCTCGTGCACGCCGTAAGTCCATGGTGGTCGGCATGTTTTATAGGATTGA

AACAAACCAACAAACCGTGAATCTTTTGAGTACATTTTATCGATCTATCGAAATGTTGGC

TCCATATGAAATGACGAGGAAGTACGCGACGGTTACAAACGCGATTGGTATGACCGGCAA

AGGATGTGCCTAGTCTGCTCAAAAAAAAAAA

>1134

TCGTGGGGAGAAAACAAGCATTCTGCCAGCACAAACCAACAAAACAAACGTGTTGAACAT

CATCCACAGTGTTCAAGCTCACAAAGGGTGACTCTTAAATTGCGTCTAAACACCAGCAAC

TACGCTTAACTACTACCATCACGAGATTACCAACGTCTTAGGCTACGATAAGCTTGCATA

TCTCACAGAAACTGTCAATGAAAAAAAAAAA

>1135

GTAAACGCATTTTCATGCCCAAAATGACCCGACCTTTCTGGAACTGGCAGGTTGTTGTTG

ATATTTAGCTCCTGAAGCACTTCGCGAGCCCGGCTTGGACAATGAAGACCGACTCGTACT

CCCCATCGCGGCAGGTAACACTAGTAGAACATGTTTTGTAATAAAAAATATTGTAATGTT

GAAATTTAGACTGTTGGTTCAAAAAAAAAAA

>1136

GGGTCCGCCAGGTTCTTCATCTTGAAGAGCACCCAGTCCCAGCCCAGCGCCGTCTGCGTC

GGGTGCAGACTGTGCTTCACCTTGCTCCAGCGCTTGCGCACGATGGTGTCCTTCTCCTCC

GGAGCCACCTTGGCCGCAATCTTTTCTTTCTTCTTCTTTTCCTTCTTTCCCTCTTCAGGA

CTCGACTCTTCGTTTTCATTAAAAAAAAAAA

>1137

GGCGGATTGTTCCGGGGATGTGGACTTGAAACAATTGTTGGATTTCCACAAGCGCGCTAC

GAGCTGGCTGTTAGGGTTCTTGATGTGTAAATTCGCCCCCCCCGGGCATATAAAACCCAT

CCAAGCATGTTCAGGCGGTGTTTGAGTGAATGGCTTCACTCGCACCAATAAACAATCTTG

CCAGCAATGCAAGCATCTCTAAAAAAAAAAA

>1138

TCGGACAGATCCCCACTGAATACTGCCACTCCACTTTTTGAACAGTGTAGGAATTCAGCT

GATGAGCTCCCCCCCATCATGGAGTCGGAAAAAACCTCCCACTGCGAAAGTTCTTCCACC

GCTTTAAAGGTGAATAGAGGCCTTTCCTCGGGGGGGCTGACTTCCACCACTGCATGCAGT

CACCAAAACCACACTTACTGAAAAAAAAAAA

>1139

CCACGCATTTTTTTTTTTTTAATCTTGTCTAAGGTTTTGGCTAGTTTTCTATCTTATCTT

CGAAACCAGCTCCGCTTTCGTGCTTCGTACAACGAACCTCCTTATTGCCTGTGCTGGTTT

CTAAGAAAATCTAGAAAAATNGCAAAAACTTTGGAAAAAAAAAAA

>1140

TGTTTTGGTGTTTCACAAATTCTTTCGCAAAAAAATACAACGTACAAGATAAAAAATTAT

GAATACTCTCACTCGTCCGAAAATAATCGATGTTTCCGGACAGCACAAATGCTCGCCTCG

CAGCACGTTGTGCTCAAGCTTGTTTCGATCGGCTGTTCACAATCACGAAGTCACGGGCTG

GGCCGAATAGCGGTTCTTGGAAAAAAAAAAA

>1141

GCATGGGCATGTTGATAACAGACCCAGGAAATAGTCAGGATTGTTGTTTCATAACCGTGT

GAATTGAAGTCTGGTTTGTCCATCAAACACGGCGTATGATCATAGCGTGCGGCAAGTTGG

ACCTTTATGATGTACATTTTGTGAACCGGTGAAAATGGACACGCCGATCAATTTTAACGT

ATCTGTTCCACCAGTTGGTTAAAAAAAAAAA

>1142

TGCCAGAGGACTTGTGTGATTCTATATATGTTGTGCGTACTTGTGGTTTTCCTAAAAGTG

CGCGCTGCCCACGTCGAAGAAAACAACAATCACTTATGGACCAACGAATAAGAAGTCAAT

TTCCAAGTTTATGATAGTGGGGACTTGTTCGCGCGAGCACGCAACGTGGATATGTTCGTA

GTGCACGTATATGTAGGTTGAAAAAAAAAAA

>1143

GAGTCGTGCAGATGAAATGTGTTGAACATACACAGGTAGTTGACACGAGTGTGGCTGTCT

ATCATGCGTCTGCATGTGTGCTTACGTTTAACAGGTAAGATTTGAAGATATCACATACAC

TTCATATGTATCGGGACATGACGCTTATATTAAGGTCGGGGAGCCATATGATCCTTGAAT

AAAGGTCGGTAAGCCAAGTCAAAAAAAAAAA

>1144

CCAAACTTCGCCGTGCGGCTCCCGCAGTTGACCATAAGACGTATTTTCGTTCCATCTCCT

GGTTTCCGAAGCCTGTCGTTGCAAGGGAAAGCCCGCGAACCTTCGTGCGCGCCCACCCCG

CCCAACTGCGAGGCACAGAAAGCCCTTGGCCTGCGACATCTATTAATTTTCCCGTGCTTG

TGCCTCGTGAATTCGTGCACAAAAAAAAAAA

>1145

GACCGGATAGATATGCCGGAAGAGTATTAGTTAGGGATAGCGTAGGTCGAGCCTCGGTGC

TCTGTGCTTATAGCATAGATACGCCCCGCCTAGAATTAGGAAGGAGCAGCGCCCGTGGCT

GGAGGGGCAAAGTCCCCTTCGAACCAATCGCCTGCAGTGCAGACGGTTCAATTTTATCAT

ATTATACCCCTTACACTCCCAAAAAAAAAAA

>1146

CCCCATCGAGACAGTATACTGATCTGTGGAAATCCATGTCACACAAACTCTTTGCTTGTT

TCATATGTAGTCGGTCGCCGGCACGTAAGCTCTGTTTGTTGGTGCACCTTCGAGGATTTG

GTCACAGTTTTCATTGAGCATGTCTGCCCCAGCATTTTGGATTGAAAACAAATATTGGTC

ACGTAACGAGAAACGCACAGAAAAAAAAAAA

>1147

CCAACCCCCCCCCCCCCCCCCCCCCCCCCCCCCCCCACCCCCCCCCCCCCCCCCCCCCCC

CCCCCCCCCCCCCCCCCCCCCCCCCCCCCCCCCCCCCCCCCCCCCCCCCCCCAAAACCCC

CCCCCAAACCCCCCCCCCCCCCCCCCCCCAAACCCCCCCCCCCCCCCAACCCCCCCCAAC

AAAAAAAAAAACAAAAACCCAAAAAAAAAAA

>1148

TTTGACAAACATGTCAACAATAATACGCCATTACGCATACGAATGTGTTCACAAATTCGA

ACATTTCCCCTCATGTTGTGAACAATTGTTGGTCTCAGTCCAATCGATGGCGCAAGTATA

AATTGACAGCTAAGCTATCTGTGCACACCCGATAGACACATTCATGCGCGCACACTGTGA

ACATCTCATGCTTTCTTTTCAAAAAAAAAAA

>1149

CAGATGAGTATTTGGACGGTGCCTCACTTGCCGGTGAACAGGGAACTTCTATGCCTGACA

GGGCATCCTCANGGTCGGAGGCCTCCAAAGCTGCTTACTCTTGGTAGGTTTGAGTTCAAC

GTCGCCATCCATATGTACGGGCAAGTATAGTTAAAAATGTAATGTATCACAATGATTAAT

GAAATGTTGTATTGAAACGGAAAAAAAAAAA

>1150

CATTTTCCCCCCGGAAACAGTTTTGCCCCTGTTTTCCCCAAGCTTTTTTGGGGCCCCTTT

AGAATACTCAAGTTTTGCTTCCACCGCGTTGGGGGTTTTCCCTTTTGGTGGCCCCCCCGG

GGGCCGGGATTTCCTTTGTGTTTTTTTCCCCGGCTTTCCTTTCTTGGGGGTTGGGGGGCT

TCCCCGGAAGATGTTTCCTTAAAAAAAAAAA

>1151

GCTGCGAAAGATGCGCGCTGCGGGCACAGACTAGAAAAAAGCCAAGAAGAAATAGAGTCT

GGGCGTTTACGAAGAACTCCAACACCTCGCCACGAACCACTCGTAGATTTTTGCTAACCA

GCTTGGGTTCTATGACAATGCATCGCTTATGCCGTGACCAAGAAAGAACTTTTGGTGTAT

TATTTCCATTTTCTGAAAGTAAAAAAAAAAA

>1152

CTTATTTGCGGGAGGGGGCCCCCCCCGGGTCCCCCTCTTTTTTTTTCCCGCCCAAATTGG

GGGGTTTTCAATAAAAAAAAAAA

>1153

GAACTTCAACCAGTCTCGTCCTCCAATAGAAAGCAAAGACAACCGCCACTCACAGGACAG

AAACGCGTCGAACTCTGCAGGCATCGTACACTTTGTGAGTCCGTCAGAAAGCCTTTGATA

GGATGAGCTTTTGGTGTGGTATACCAATTCTTCAACGTCGACCAGCCAGCCACGGGCTGT

TGCTGATTCGGTCTCTGAGCAAAAAAAAAAA

>1154

GTGTTTTTGATTTAAATATTGATTTATTAGTTCATTTATAATGACTTAAAAGGGATACTT

TTTTAACAAAATTTGAAAAGCCCCCCAAAAAAAAAAA

>1155

AAGAAACAGCGCTTCACAACACTGCAATGTTTGTGGAATGTACTTTGGGTTCAGTGAATG

AACTGAGATTCATGAAGATCCTTCTCTTTTGGCCCATATGATGGCCTCAGGCAATGATAG

TGCGTGTCATCACTGTTTGATTGCTGGACTTCTTCTGTGATATTGTTACCGAGCACATTC

GAGAAAGCTTCATAATTTCCAAAAAAAAAAA

>1156

TCGAACCATCCATGAACCCCGAGGTTCCAGTGGGATAGCTATGAACGTGGTCAGATGGCT

GTTGATTCGCAAAAAGTCTGCCGACTGTGATAAACCTGACTTCAGCGGAATTTCGCAGGG

GGAGTATCCTAGAGACCGAGGAGTTCCGTCAAGTTCGTCCATCAGGCTCTGCAGAAGAAA

GGGAGTCGACATGCTGCACGAAAAAAAAAAA

>1157

AGACTGAATCGGATTTGAGTGAAGCGTAGTTTGGCATTTCGTGAGACCCCATATCGATGT

ATGTGTCAAGCATAGAACGGAACAGGCTGTTTTTAGAGAAGGGGAATGAGCCAGGGATGG

AATCCCATATTTTTGTTGTTTGGAAGTCAAAACTTTCCCCCATGTGTAAGTAATGAGGGG

AATTTTGAAGAACTTGCGCTAAAAAAAAAAA

>1158

GCAACCAGGCCAAGGGGGGGGGGGACCCAACCAGTACGGATTAGATTGTTAAGTTGTACT

GGAAACTTGGGGGGGGAGTGGGACTTTCCGGGGAAATTTTGTTGGGACATGGTATTTTTT

CCGCCAACTTCGCCCAAAAAAAAAAA

>1159

ATCCGTACGATTGATATGTGAGCTGAGCATAGATACAGTATATACAATGTTCCACAATCT

AGCAGCGTCTTGCGTATTTTATGTTGATGGCGTTGTTCTGAGAATGTAGCAATGCGTGTA

TGAAGTGGAGCCATGTACGAAAGTTGAATTTGCACTTGCTAATTCAAAGGTCTGCTTTCC

GCAGCAGTAAATTGCCATGTAAAAAAAAAAA

>1160

GCTCCTGGAGGGTGATGTAGCCCGAACGGTCCTTGTCAAATCCCAAGAACAGCTCTTTCA

ACCCTTGAATCTCGGAATCCGTCATGTTGGCCGCAATCGCCTGGAGTGCAGCTTTCTTGA

ACTTGGTCATTTGGGAGTAGCTCTTGAGCCGCTTCATCACTGTGTCGTCAATGGGGTCAT

CGGACGCTACCCCCCCTTCCAAAAAAAAAAA

>1161

CATCGCAAGGGAATGGGATGAATCAGATTCGGCAGCGAACAGGGGCGCACCTTGTCGTGG

TAGTGGTACGGAGTGCGCCCGTTCCAGTCCTTGAAAAAACCGTAAGCGGTGACGTCACGG

CACAGGTGGGAAGCCAGCAGAATCGCGTACAGCCCCGAGGTGGGATTCACCTGCGGCAAT

CAGCTCCCTCGTCCCTTATGAAAAAAAAAAA

>1162

GCGCAGCTCCGCAGCCTCCGCCTCCGCCGCTGCCAAGCGCGCACCACCTCGTCCACGCTC

TCTCCAGACTTGCCGTCCCCGCCCGAACTGGCGCGCACCCTCAGCGTCTTGGCTCGATGG

AGGCCAGGCCTGGAGGAGCTCCTGCGAGTCGCACCAGCGTCACACCAGACGCGAGAAACG

CATCAGTTTCGGTAAAAATGAAAAAAAAAAA

>1163

CCTGCCAGATGATGAACCTTGTACATGTACTAGAATCGAACAGAAGGATGAAACAATCAG

AAACCATCTACAATTGTATTGAATGTCGACATAGCCTCCTTTTGGACGATGATGGTCCTT

GGATATCTTGTTGATGAATTGTCTACTTTTGAATTAATTGCTTGGATCATGCTAGCAGTT

AATTCATTGTTTATGTTCTTAAAAAAAAAAA

>1164

TTTGTTGGCCTTTTGTTTTGAACCCCTCCCGGATCCTGCCTAGTTTTTTTTTTGTTGGGC

CGGCCGGGTGATCAGCCGTCCCTTCCCTTTGAAGCCCTTTTCGCAGGGGCCTGGTTGGTT

GCAAACAACCGTTAACCCTTTGGTTTCATGTTTGCAACCGATAAGGGGTTGTTCCGTTTA

ATTTGGGGGCCCCCATTTGGAAAAAAAAAAA

>1165

ACCCCGGGTGGGGTGCCCCCCAAACAGGTGGGGTTGCAACCAGCTTGTTGAGAAAACGCC

CTGCCCAAATTTGGTACTGACCCCATCGTGGGCCTTGAAATGTCATAGAACTAAGGGGCA

AAAACTTTTTGTGGCTTGGGGAAGAGAGTCAGCCAATTGATTAGATTGTCAATGGAATGA

ACCTAAAGACCTCGGGGCGGAAAAAAAAAAA

>1166

GTTTACGTTTTCGATCATCCCGCCCTTTTTTTTTGAGCCCATAAGATCACTGTGTTTGGA

ATTTTTTTCCCCGAGTTTTGACTTGATACTTCGGAAGTTAGTCCACCAATCGTTAAGTCG

ATGTTTGCTTGCGGACGCCCCCCTGGATTAAAGTTTTTGGACTAACGTAAATGGGGATTG

TTTAGTTGATTGGTGTGCTCAAAAAAAAAAA

>1167

TTGTTGCTAAAGTATCACAACCGGGAGTCGTGATGGTATCATATCCTGGCCGCTGATGCG

CCAAGGGCATCTTGAGCGATTGAGATCTCGTGTAGCTCGATAAAATATGTACCACTTCAG

CTTGACTATGTATTCTGCGAACGCAAATGTGTCAGTGAGTGTACATTCCTGCAAAAGTGT

TTAGCCAAGGTATTTTTGTCAAAAAAAAAAA

>1168

AGGTAATGGCCAGCATTCCCAAACAGGCCTCCATGATGGCCACCTGCAGTTGCCGGTGAA

ACCATCCCGAATACCATCAAGACCATTACCAGAGAGCCCTTTGTCTGAATTCGCACCATA

GTGTTGCCACCTGTTGGACGCTGATTGACTGCCTTGGCAAGAGGGTTGTGGTCTGGGGAA

GCGCACGTTCACCTTCCCGCAAAAAAAAAAA

>1169

TAGTATTTTGCCAATGTTATCAAACAATTATCGCGTATTGTAGATACAATGCCCTTAAAG

TGCACATATATGACTATAGACATGTGTTGCACTTATGGTATGCAACCATAATATTGTGCA

TAAGTATGAACTAGGTCTGATATTCATGGTAGGCACATTTTGGCATGTGTTGTGAATCTG

TTACTGAAGTGATATAGCCTAAAAAAAAAAA

>1170

CGGCTTCGAACACACTGTAGAAATTCTTTGAGCCTCCAAATGCCGTCAGCACGTACGAGT

TTGTGACATTGCAAAATACACCGATGTCATTGCTGTTTTCAAACTGAAGACGGACCGCCA

TGTTGGAGGAGCAGACAAGATTTCACTAGCAACACGAATACCGGCGTTACTGGCGGCGAT

CTCGGTTTGGTTGATACTGCAAAAAAAAAAA

>1171

AAGAGGGTTATTAATTCGGGCCCGGGGGGGGCCAACCCACCAGCCAAGTTGTTGGGGGGG

CCGCCAGACATTTTTGGTTTCTTCACCCATTTTTTGGGGGGGGGGTGGCCAGATTATTCG

CCCCATGATGTGTTTTTTCTTTTGGGGAATATCCGGAAACTGGGTTTTTTTTTGTGGATT

ATTGTAATGTTTGGGGGACCAAAAAAAAAAA

>1172

ATTGTTTAAGCGATTGGTATGATCACTCACTCGTCGAAGACGACCCGCCCACCAGCATGA

CGATTTGATGAATCAATGGTCGAGGCAATACCTACCCAGGACATTTGGACTGACATGTAG

TACTTTCGTCGACACAAGTGACGTGTCCTTGAACAGTTGCATTCTTCATTTCTCAAGTTT

ACTACTTTCACACATGCAGCAAAAAAAAAAA

>1173

ATGCCTATTGTACCCTATCACAAACAAGTGTGACAGGGTGTAGCCGGTGCACATTGTTGA

AGTAAAGCTCTTTCACGCAGGTGGCATGTTAGTAAGACTTTGCCGCCAATATTGCGCATA

GATGGAATGTGGTTATGTAAGACGAATCGCATGTATAACATACACGAAAGCATGGTAGCT

TCTGAGGAAACCCATGATCTAAAAAAAAAAA

>1174

AAATAGGCCTCAGGGTGCAAGATACGACTGATATATAGAGTTGTAAATGACAAACAATGC

TGTTACCGTTTGGCTGGATTGCCAAAGAACTATCTTGTGGTTCTATGTCCGACGTGCATT

TGATCAACGCGAGTATTCATATGGACTTGCACTAATAAGTTCTTATACAATGTATAATGC

ATTCTGAACAACTTGGTTGTAAAAAAAAAAA

>1175

CGCTGTAGGAGGGACGAGGGCGGGCAGGGCCAGAGATGATTTTTTTTTTGCCCCACCCCC

GGGGGGGCCGAAAAAAAAAAA

>1176

ACAGCTAAGTAGAAATCCCTCTCCAGGTAAGAGTGCACAACTACTCCGTGGTTCAATTAG

AATTGCCAGAACTACTGCATATGATTCGCGACCACATATTTAGTTTCCTCTATTGTATTT

CTCGACGAACCCACAATGTATCAAATCGTACCATATATGCTGTGAAATATATGATCCGCC

GGATATCATGGTGAATGTTTAAAAAAAAAAA

>1177

GTCGTACATAATCAAAGCACGTGCTTTCAAGTGATGCACGTTATTTCATGTGATGCACGT

TATTTTAAGGAATGGTTTCAGAATCTATGGAAAGGGGTCAAGTGTGTGCATGGACGACAT

TGAGGACGATTGTAGAACACTTTCTGGTTAGATTGCGAAAGCAGAAAGCTTACTTTTTAT

TTCAACCACATCTGCCTCTCAAAAAAAAAAA

>1178

AAAAGCCATTTTTTGTTAACCAGAAGGCAGAAGGAGGACGTCAAGTTTTCATGGCCCTTA

TGGGCGGGGGTGGAAGCGTTTTCCAATGAAAATTCCAAAGGGGGGCAAAGAGGCAAATTT

TAGCAAATCCTAAAAAATTTCCTAAGTGGGGGTTGGTTTTTGCAACTCGGGCCCCTGAAC

CGGGAATTGGGGGTTTTTGTAAAAAAAAAAA

>1179

TGGGGCCCCCCCTTTTTTGGGGGGGGGGGGGGGTCCCCCCCCTTTTTTTTTTCTTGGCCC

CCCCGGGGGGTCCCTTCCCCCCTTTTTTCCCCCCTTTTTTTGGCCGTCCCTGGGGCCTTT

TTTTTTGGGGGGGGGGGGGGGGGTCCCTTTTTTTTTTTTTTTTTTTTTTGGGCCCCCCCC

CCCCTTTTTTTTTTTTCCCCAAAAAAAAAAA

>1180

GTCCGAAATGCAAGCTCAAAGGCAGCAAAACGGGGCCCTTCGTTTCACATTTCATCCAGC

TATGCACCTATCATGCGCAGTTTTGTGAATCGTGGCGAATGACCGGGACTGCCGTCAATA

ACGAACTGGATATGACAGTGAAGTGTACAATATTGGTCTGTAAGTTAATGGGACTCTTCA

TCATGAGCCCGCTCGATCATAAAAAAAAAAA

>1181

ACCAATGTAGGATACAAGTAATAGGAAGAAACAAGGATATCGATGCGACATCTGCGATTC

CGCTGCGCTGTATTCGGGCCTCGTGCTGCTCGTTCACTGGCACACTTCATTGCTTGCGGC

GCTTGCATGTGTTGTTGATACAGCGCGGACCCCAAGCACAGGTGATTGGAACGATAACTC

GTTTATCGAAGACGCCCCGCAAAAAAAAAAA

>1182

TGTATGGGGCCTGGTGGATCAAGAGAGCCCAACTCAAGAGAAGGATTTGGCACTTCAATT

GATGGTATACATGAATCGGTGCTTTTCACGTATAAAAATTAGGCTTCTTTACACAACACC

AAAGACAGAACCATTCGAGCGCACGGCGCAGATTAGGACGATTGCAACAGGGAAACGTGT

TGGCTGGAAGGAAACTTGTCAAAAAAAAAAA

>1183

TTGCCAGGACCTCTGTCAGTAATTGATGTTAGAAATATCTATTACAACGCGTAGATTCGC

ACGAGGAACAGTGTAGGGAACCATAGAATAGCTACTTAGGGATGACTCCTAGCCATCTGA

GCCTAGACTGCCATAGTTACTTGGATGCTCGTTTTGTTCTAGTTTATTATTTTTTGTGAA

TTGTGTAGCTGACATTTTTGAAAAAAAAAAA

>1184

TGCCTTTCTGCATCTCTCTGCAGTGGTCTGCTGATGGCTCAACCCTGTACACTGGGTACA

CTGATGGTCTCATCCGCGTGTGGACCGTGGGTCGTGCATTGTAAGGAAAATGTTGCCAGG

AATTTTGTAGCAGCATAATTTTGATGCTAGCGCTGTTGTTAATTGACAATTTGTGATGGG

ACATAATATACTGAGTCTGCAAAAAAAAAAA

>1185

TCTTGAAACGACGGCAATCCCGTCCAAACCTTTGGCAGGTTCGAGCTCAAGATCCTTCAC

AAACGATCCTGGTGTGTTCTTCACAGTTTGTGTAGATGGATTTTTGAATAAGCTTGAAAT

AAGTGCCTTGTTGCGGGAGTTGTTAGGTGCGTCGGCAATACCGATGACGGTGTGGCGAGT

GCTTCCAAAGGGAGGTTCCGAAAAAAAAAAA

>1186

CGAATGTTCTGTTGGGATACTGCACAGGGAGTGTGCCCGATCTGTAGGAAGGAAGTGGCT

ACCTCTGAAGGTCCCGGTGTTGAGTCAATAACTTGAGCATGTTTCTATAAGCACGCACAT

GTTTTAGGGAAAAACATGTATTGTCAAAAGGAGACAACAAACACCCGTCCCTACCGACAA

ATTAACACAAACACACCCCCAAAAAAAAAAA

>1187

GCTCGGTGGTGGACGCCTGGAAATCCGCAAACTCCTCACTCACCGCCAGGCTGCCGTAGT

CCACCTGCTGGGACTTGCCTGAGCAGTACATCTGAATGATCACCTTCAGCTTGTTCATGA

TGCTCTCCGCCACAACCACCGACGGGCGCTCCTTGCCGTTCCACACCGACTTCATATTCA

GGATGCTTCCCGCCTCGTCCAAAAAAAAAAA

>1188

TGAGCACTCATACTGTTCTATTTGTACGGTGATATGTTCTTTCCAGAATGTCCTTTACAA

CCAAGGGAGAGGTGTTTCTGCAAAGGATTTTAGTGATATGATGTTTGAATCCATGCTGCC

TCCGTGTTGGCTAGGGTGCATAGTCCGTGTGAAGATATGAACTGACGCGTCATGAAAGCT

TGTACAATTGATTTTGGGTGAAAAAAAAAAA

>1189

CACGTTTGTGAGTTTTTTACATCGCGCTAAAATTAGAGCTCCAGCAACCTAGACCAACCG

TGAGAGCGATTTGATAATGTTGACGGGGATCAGATATGCCATGCAAATGAAAATCCTTTG

CGGGCATTGCTTTCTTTTGCGACATCTCAGGTTGCACCCATAGCATTGGTCGTATTTAAG

TGTATTTCTTGATTGGTGGCAAAAAAAAAAA

>1190

GAAATGGTAAGCAGTGATACTGACTTCATACCCACCAGCTCCAATCTCAGGAAAACTGCT

ATACAAATTGAATGTGGCTGAATTATGGTACTGTGCGGCACACAGGTCGGGATATTGAAT

ATGGTGCATCGGGGTATTCGTTTTCGATTTTCAATTGAAATTTCGTTGCTATAACCGCTC

TGTATATAGGTTATTGCACCAAAAAAAAAAA

>1191

AACTTTTCGTTTTGTTCGAAAAGCTTATTTCATAGCTCAAACAGTGGCAGTCATGTTGGC

CGGTTGTTTAAGCACATGCAAAAGAAACTAGCACGCTCTGTGAACTGGAATAGTCTTCGC

ACCACAGAAGAGTCTCTGTTAGCGATGAATCAATTTAACACTTGTATTTCCAATTCACTC

ATTAACCCATTGCTTTCTGTAAAAAAAAAAA

>1192

TTGTAGAATTGGAAAATCATGAGATTGTGTGACATACCAATGAAACGGCTCGTAGACTGT

CCAGTGTTATTTTGTGGCGATGTTTGGCACTTTGCGACATGTATGTTGCCATGAAGCGAG

TCGTCAAACATCATCAGACGGTACAGCGTAGCATTCTAAATCATGTCATATGTGAGCCAG

CATTATACGCACATTATTCGAAAAAAAAAAA

>1193

GTAATAGCAACTTGTACTATAACCAGATATGCAGCATTACAATATAGGATTAGAATGGAC

AGTTTCTAGATCAAAAAGAAAGAAGATGAACTAGAGGGCAGCCACTATATAAGGCAAGAC

TGGATAGATCATTCCTCAATTCAAGTGTATATTGTGACAAGCATGTATTTATCTTGATAT

CGATCTTATCATGGTTATGTAAAAAAAAAAA

>1194

ACATGGGGATTTGGAATACCTGAATGGAACACCCGCATGTGCAATATCTGAAACCACAGA

TTACTTTAACTAGATGTGAATACTAGGATGGCCACAATTCTCAATGGTGTGTATTGTGTA

TTGCTGTGATCAAGGCGTTTGTGATGTGACAAAGGGCAACTCATGTTGTGATTTAGTATA

AAAGACCATTCGCGAACCATAAAAAAAAAAA

>1195

TTGAAACGAAATAGTACATGCACGCTACAACATGACCAATAAATAACATCATGAAGACCA

ATTTTACCACCACAAGCAACTGTGTGATGTGGAATAATTCGTCCTGATATCGCTGCAACA

GCCTGTTGAATTTCATCAGACCGATAAGCTTCACAAGGCGAATCATGCGCAGAATCTTAA

AAGCTTTAAAGTACTGCCCCAAAAAAAAAAA

>1196

CTATTTTACAGCTAGGACGCTTCATTACTAGTGATCTTGGTCTTGTGAGTAGAGTTCGCC

AAGTCTTGTGTGCCATTGAACTGGCATATGTTTCAGAAATGGACAAGACGCGCCTTGGGC

TATGCCATCCGCTTTTGCTTCCACAAACATGATGACTTGACATAGCGTTTGATAATTGCA

AGAAACCTCCCCAGCCTCGGAAAAAAAAAAA

>1197

TCCACGCTGTCCTCCTTGAACTCCTCACCCGCCTCCTCAGCTTTCTTCTTTGCTTCCTCA

ATGGCTTTGGCAGTTGCCTCAGCGTCCGGCACCTGCTTGGGTTCCTGCTTCTCACTGTAC

ACCTTGATGGGGAATGAGATGAACTCTGAGTACGTCTTCACCAGCTGGCACAGGCGCGAG

GCAACTGCCAAAAGCCATTGAAAAAAAAAAA

>1198

GTGGTGTTCTCCCTCAAATAGATACGTATCATGTATGGTGTACTTTCTGCTATATTGAAG

TCCCGCGAGTTTAAGGCTGTGCTGAAGACACATTCCCGTTCGCGTGATGGACGCGCGGTA

TACTTCAAGGTATTTGAAAAGCTTGGAACCAATTTCTATGGTAAAGCTGCGTGACTACAT

GAATTGACTTATCGCAACCTAAAAAAAAAAA

>1199

ATGTCGCTCAAGCTTGAAGTGACGTTGACAGGTTTCGCCATTCTCGATGAACACCTTCAC

TCTGAACACACTATTGGAAACTCCTCCTTGGAAGTGGAGTGGGCACACTGATACCAGGAC

GATGTAGATAGTCTTGAAGAAAGTGAAACTAACCTGTGTATTATTATCTGTACGTTTTGT

ATCGCTTATTATCGTGCTGTAAAAAAAAAAA

>1200

GAGACGCAAATGTTTGAGCGTCCCGATCATTGTAATCGGTGCTTGTTAGAGAGAGTTTTC

TGAGTACGTCGTGGGTGTTCATATCACGAAATTTGTGTGAAGTCAGCGCATATGGATGCA

AATGGGCAACGGGAAAATCCTGGTGCCTTTTGTCCCCGTCATTCGTGCTCATCGTTTAAA

GCTCAATCTGACACAAGGCCAAAAAAAAAAA

>1201

TGGGAAAATAATGCAATAACTGTATCCTCTGTATCTTGATGCGATGCACAGAGCTTAGTA

TTAACCCAATAAGTTCAGAGGTGCACAGTTCTAAACCCAATAAGTTCAGAGGTGCACAGT

TCTACGGCCAATGTGGATTAGTCTCATCCTGAAAGACGATTTGGTTGTGCATCATGTGTA

TTAAGGTACTCTCTCCCCCCAAAAAAAAAAA

>1202

GTGTGCGCTTTGCAGGGGCTTCACCCTCAACGTCTTCTTCTGGCTTACGCTTGCCAAGTG

AAAAGTCAGTGATCTCGGAAACACCATCAACCGTCGTGTTCAGATTATCGGCTTCTGGCG

CGATCTCGGAGATGGTTTCAATAGGGGCGTTTGAAGAGTCGTCTGCCATGGGAAAGCGTG

TTAGTGCGAAGCAGGTGCGCAAAAAAAAAAA

>1203

GACCTATCATTCTTACTTCAAGAAGTACTGTAAGAGGCACGTAGCTCTTCTGATATCCAC

CTTGTCATACGTTTCATAGTACCCCATCCTCCTCCAAAAGATGTTGAGCTCGAGACTCGA

TCCCCTCACAGACATTGCTTGTTCTTTTGGCTTGATGTTGTAAGATATGTGGTATTGAAG

TAGACACGGCTTACGGCACCAAAAAAAAAAA

>1204

AGTGATGAATGCAAAATACGCCTCACCAAGTCCCAGGGATACGTAGGCGTATCGGTATCG

CATTTAAGCTTGTCCACGGCAAGATCACTCTTTGAACGTGTCTTGCTAAGACTGTTCATG

AAACGGCCCTTAAGTACACGTGGTGGTCGGATCATGCAACATCCGTGTAAGCGCTTAAAG

ATGGGTCAGGATCGTGCTGCAAAAAAAAAAA

>1205

TTTCAGTTCAACACGAAGCATCGTGTTTGTTCAATCACAACGGAATCCAAGGCAAATTTG

TTCAGATATGGGTGGCTTCTTTCCCATGGCAAACTTGTGGGCACGTGCCTAGAATTGAGT

AGATTAGATATGTATGCTAATACGGGTTGAATGTATGACATTCGTAAATGTGGTGCTAGT

GGACAATATATGTTGCCTGTAAAAAAAAAAA

>1206

CTGGGCATTCGCAAGACAACATGTGCCATCTAGGTTTGAACGCACACCTGGTGCTCATTT

AATATCAAGGATTGACTTCACCTGTGTGTATGTTATATTGCACTCATCCCTGTAAGTGAT

CCTGCCCAACAACGCAAATCGTGACAACCAGAGTAAATAGTGGCTTTTACTCTAGAAAAT

GTGTTCGTCCAGGCTTTATCAAAAAAAAAAA

>1207

CAAGTCTTCGGCGAAGCAGGTCCCATCACGTGTGAGGATAGAATGACATGTCAATCATTT

CTATGCCATTTGAAATCCCCGTGGTTCATCTTACCATGCTTGGATTTTAATAATGATCAC

ATGCGCTGTTTGTACATTCAAACTACTACGTGAAGACAGCGATACATTGTAAAAATAAAG

TAGCAATCTTAGTTCTTGATAAAAAAAAAAA

>1208

CCAGTTAGCTGGAATTACAGTGCCCGGTGGTATGGAGAATTGAAGATTGATGCTCTTGAG

CTTCCAACCTGAGAGCCGTGTGTAGGTCGAACCCTCGAACCATGACATCTTGGATGCAAT

TGTTGTAGAATATCCACAATGTAGAGCATAAAATTTACAAATATTTTATGTTAAGAAAGA

AATACTCGCAACATTCAAGTAAAAAAAAAAA

>1209

GAGGGGGGGCCCCCCCCCTTTATTTTTTTTTTGGGCCCCCCCCCCCCCCCCCCCCGGGGG

CCCCCCCCCCCGGGCCCCCCCCCCAAATTTTTTGGGATTTTTCCCCCCCCAGTTTTTTTT

TTTTTTTCCCCCCCCCCCCCCCCCCCCCCTTTTTTTGGAGTTAATTTTTTTTTTTTTCCC

TTTTCCCCCCCCCCTTTTTTAAAAAAAAAAA

>1210

GATGGACGGACCAGGCTGAGAACTAACCTGCCGATGTTTTCTTGCTTGTATGTATTTACA

ATAACAATTCTATGTATCATGTTCGTTGAAATTGAATGAGGTCCACTCACTCGTGTGTGG

ACGATATTCAACATTTTCATGTAAATGTGTGGTTTCATTTCAACCACACACATGCATGAC

GAAGGGTGTGCATCATCTTGAAAAAAAAAAA

>1211

CATGAAACGTGGATGCTAGCACCTGGCCCTGGGAACAGGTGAACTCATCAATAGCCGAGT

AGACGACTTGGAACCAAGTTTCGCATAGAGCGTGTGATCACGCGCAGAGCTATCCAATAG

ATCAACATAAGTGTGCCTAGAAGTACTTCTATTACCATGCTGTACAAATCTGTAAATGTC

AACATCATTAGCCATAATGTAAAAAAAAAAA

>1212

GAATGAACTGAGGTAAAAGCAATTAGTAGTAGAACCAGATATGTTGTATTGCAGTATAGG

ATACGAATAGATGGTCAGTAGATCATGTATGGACAAGATGATCTAGACAGCCGCCACTAT

ATAAGGCTGGATTGGACCTTTTCACTCCTCACGCTGTGTGTGTACAGTAACATGTTCTAT

GCTAGCATTACTGCAATCATAAAAAAAAAAA

>1213

AGGGCCTGGCCCTGGTTTCTGGCCGTTCCCCCCTGGTGGGGGGTAAAATGGGTTTTTTGG

TTCCCGGGGGGCCGTTTTTTGGGGGGGTTCCCCCCCTTTGTTTTTGGGAATGGTTTTTTT

TTTTGGCGGGGGGGGGTTTTTTTTCCCGGTTTAGGGCCCCTGGGCCTTTCCCCGGGGGGG

GGGTTTTTTTTTTGTTTTGGAAAAAAAAAAA

>1214

CGGTAGTTATTGTATGTTCTATAAACTGCAACATTCAATGGAAGACCTTGACGAGAACGT

TTAACCAAGGAAGCACATGATGCTTCGTAGAGTGGACACGTTAGTTATGCGGGATACTAG

AGCATCTCACAAGAGACTTTCACTAGCTCTACGTCCAAGATCATGTCAAAGATTGGGTCA

GCAACTATTTCAAGTGTCGCAAAAAAAAAAA

>1215

GTGCATGGCTCCAGCATGAGCTATGAGCAAATCTTCTTGCAAATAGGTAGATCCGGCTTC

TGCAAAGACAAGCGTTGGGCATTGAAGCCATGCGATGAGCTGGCCTGCATGCTGCCTGGA

ATCACCTTCGATCTGTAGAAATTTATCATTTGTTGCAGACCGTATAACAAGTGTGAAAAG

TACGTCTTTCCGTTGCATTGAAAAAAAAAAA

>1216

ATTGTACTTTCGGGCACTTGTTGGACGTTTTTGCAAAGTTACGTTACATACCGGGGGGAC

CCGAGGTTTGCCTTTGGTTTAGTGCCGTGGGGGTTGGTGTGATAAACTGTAAGTGGTGGT

TTTCAGAAAGACCCCATGGGTGTGATAGCATACTTCAAGTGTTGGAATGGAATGTTTTTA

CCCCTTTTACTGGGGGCTGTAAAAAAAAAAA

>1217

TGTCTCAAGGTCTGGATCTCGGAGACGTCGCTCGAGAGCCGCCAAGCTTGGGTTAGGTGG

TGGTGATGACCACAGTGTGAGGAGCTAACCACTCATACAATGGCATTGAGGTAGATGCTA

GCAATGTGTATGGCACACAGACATTTAAAACAACATTTTCGTAACGATATTGCCAATAGA

TAATTTCAAAACACACTTCTAAAAAAAAAAA

>1218

GAAAAAGGCGTTCAAGACTTGGCGAGATTTAGGTCGCCCTGGCTTGCGGGGCATTCCATC

AATGTTGCGGGTGAGAAGATAGCCTAGTTGCAGAATGGGAAAAGAACAGTGAGCTATTTA

TCTTTGTCATACATTAATATAGTGCTGACGCATACATGTTTTCAAGTTATTTTGTGCATG

GTGATGAATAAGCAATCTCCAAAAAAAAAAA

>1219

TCACCCATGATGTTGGTAGGTCCATACTGGATCCGATGAACATAGAGGTTTTCTCTTAGG

GATGTCACATGTCAAATGTCAAATCAACGGTGGTGGTATGAAGACCCAAGTCGCATAGCA

GCCTCCAGACCAGGAAAGGCACTTTTGCATTTTTGTAAATACACTTGTAAATTAAACAGT

TTCAGAACCTGTCTGGCTTTAAAAAAAAAAA

>1220

AATGTTTGGGAAGCGTCCAGATTACACATCGCTTCATTGTATCCATTGCATCATAACGGT

AGTATCACAACATGTGGCAGTGATAGTTTGTCCATAGAGACTTGTTTGTTGAGCTCATTT

ATTTGTATGGTTGCAAGGCGCTGTGGGGGGATTTGACCCGCACAGACAAACCTTTTTGTA

CAGATCTGTGTCGTTGGCTTAAAAAAAAAAA

>1221

TCGCCAATCACCAACAGAGTCAGCTTTCATGCCGGCACTCCGGATGATTCACCGTACAGG

GAATGTTACCGCATCAGCCTGTCTCAGCGTCTCTGCGCTGCGCGTTCAAGAGAACAGTGA

CCCAACCAGGCAGTAGGGCAAGGTGATTTCGGTTCTTGGCAGAGCTCAGACGCACACCTC

ACAAGTGAGAATTTTGAGATAAAAAAAAAAA

>1222

ATAGGCATCCGATAAGTTTGAAATCAGAGAACAGAGACGATGTGGTGGGGTGATCCACCT

GCGATGAAACCGGTGAAGTCTGTAAGTTTCGCACCCTTGCCATGCATAGCCCACCTGTAT

GGTGAAGGGATAAGTGCCCGTTGACTCATTCACATTGTAAGTGCAAAGTTTTCATGAAGG

GGATCCGTGTTCCTACCTTGAAAAAAAAAAA

>1223

GGGCCTCCCCCCTCGGCCCCCCTTTTTGGGGGGGCCCCCCCCCCCCCCGCCGGGGGGCGG

GGGCTGGGGGGCCCCCCCCCGGGGGTTTTTCCCCCCCTTTATTTTCCCCCCTTTTTTTTG

TTTTTTTTTCCCAAAGCCCCCTTTCCCCCCCAAAAAAAAAAA

>1224

CTCATCGACAGATCTGGACCGACGGATTTCGCCTTCGTCAGCGCGATCGCTATCTGAACC

CATGGCGTCACGAATGTGTTATTGCAGTGTTTTTGCAATGGGTTGGTATGCGAGAAGGCC

GGTGAAAGGCGTTAGCGACACGTGCGGGCGGACCCTTGCTGGAAGTGTTCCACGAGTTTG

GCGCCAGCGACTTACAGCACAAAAAAAAAAA

>1225

CAACCTAAAGTTTGAAAAGCCAACGCGGAGCGCATGCCTCAGTTGGGACGGTCCTTCGTT

GCGCTTAGGGTTGTTGTTTTGACTCCCCAGCGACGGGCCCCCGCGGGGGAGTTGTTGAGA

AGCCGGGGTACTGTGGTTGCTGCTGCATGCCTTTGAAGCGAAGAGCTCCCGCGAAATAGC

ATCAACGTTTGAACGCGATGAAAAAAAAAAA

>1226

TGTACTTTTTAGGATTCATCTGCATGATGCCACTCTGCTGATGCTGTACACCGACTTGGA

GCATAATGCACTGTGATTACCGTGCTAGTCACGATCAAGGATTCTTGTATAGACTGACAA

GCAACATCCGGCACCTTGATTAAGAGGCAACATATTTCGTCAAAAGTTACCTCATAATTG

TGAGGCAACCTTGCTTTTCCAAAAAAAAAAA

>1227

AGGCCAAAGGCCAAAGATAGTCCCAACGATTCTCAATCCTATCCTCCAAGCAATTCAAAC

ATATGCGTTCAAAAAACACGGCGATTTCGTCCATCGGTCCTTCCGATTTCTTTAACGGCA

CGCACGCGAGTAATGTCAAAAGGTTGGCTGTTTGAGCCGCTTTCCTGCAGCTGCTTGATA

CATTGCACAATCTCGTATCCAAAAAAAAAAA

>1228

GCTCGGAGTTAAACAAGACATATTCGAGCCAAACGAGCGAGACCCGTTTGTCCTGGACAA

GGTTTAAGTTATACTAGTGTGCCGGACAGCTTGCACAGTTATAAGTATTGTTGGATACGG

ACGTACTTGTGACATTGAAAGTTAGGTGACAACTTACAAAGTGTTAGTGGACGTGGCTCC

TGTTGGCGGATTTTGTTCCNAAAAAAAAAAA

>1229

CTGTCCCCCAGACAGATGCTGTACCGGCCAACTGGTACACGAGGTGGCATCTACCCATGC

GACAGCTCGAACTCGGGCTTTCCCATGAAACAAGCCGACGCCCTACCAACTAGGCCAACC

GGCATGGTCTTTTATATTGAACCACCCAATGACATGGTGACTGTTTTCGGCACTGGGTAT

GATAACTCGCTCGCTAATCGAAAAAAAAAAA

>1230

GTTACCACCATCTGCCAGCATTGGAAACCAGTTTGCGCACCTATATCAGTAGGACATAGC

AATGCAATGACCGGAGCGCCTCGTTGTTTGAACAAATACGGAAGACATATCCTGCACACC

TGTTTTATTTTAGCCTAGACTCATCTCCGCACCTGTGACAGAACAAGGAAATGCGATTAT

GAATGCCTTGGGGCTCCGTGAAAAAAAAAAA

>1231

ATTTGCCTAATTGATAATGGCAGCACCGTGCGGCTGAAAGAGCTGTTCGATTCCCAAGTA

CTAGCAGGTCAGGATTATTCGATTGCAGGCTCGGGATTGGTTGTAGTCTCTGTGTGTGCG

TATTCAGTCATCAAACAGGCACTATGGTGAATTCTGTTCAAGTAAGACTGTTTGGTCTGT

AGTCAGCCAATTGTTCCTTGAAAAAAAAAAA

>1232

TTGGAGCCAGTTTTGGTTGGGTAGTTATTTGCCAGGGTTTGTTAAAGGCATTTGGTGCCT

GGGGAACGGGGTTCCTTGCAAGGGGGGGATTTTTGGGGGCCTTGGTTGGGTTTTTTTGCC

TGGTTTCCTGCCGGGCCAGGTTAACCTGGTAAGGTGGGTGGCCTTTGGGCCCACTGTTAA

GGGTTTACCGTTAACCCCCCAAAAAAAAAAA

>1233

CAGAAGACGGACGTAAATGGCTGCCGAGCTGGGCAAACACGAAATGCCCCCAAGTCTGGA

CCCGAATAGGAAACAAGTCCATTGTCCTTGGCTCGGAAATCCCTGATCCCTGCAAGCTTG

GTGGCCTTCTCCAATCGTCCGTCCAGGCCATAGGTCCATCTGAACCAACCTTCACAATTA

TCTCAGGTACAAATCAATCGAAAAAAAAAAA

>1234

TCGGACACAATCACCATTGATCCGTTGAAAACTAGGGAAGATCATCTTCCTCAAATGCAA

AGCAGAGACACATGGGTGACTGCGACATGCGTCACGACACTGGCAGCCTGAGCGCATTAA

AGATGAAACTTGGGGCAAGACACAACGAAAGCCATCCAACAGGAAGCTCTGATATAACAT

CCTAGATGTTGCGAAAGCATAAAAAAAAAAA

>1235

TTTTGGGGGTTTTTTTTTTTTTCCCCTTTTGCCCCCCCCAACCCCCCGGGGGGGCCCCCC

ATTTTTTTTTTTTTTTTTTTTAACCCCCCCTCCCCCCCCCCCCTTTTTTTTTTGGGGGGG

GGGGGCCCCCCTTTTTTTTGGAACCCCCCCCTCCCCGGGGGGGTTTTTTTTCCCCCCCCC

CAATTTTTTCCCCGGGGCCCAAAAAAAAAAA

>1236

GGACCACGTATGTCCCTGTTCTTTTGATTTGCTATACATTTGCTCGCCAAGCTACGGTAG

CTGATGTTTTTACGTCACGGGCATGTCACGAGGTCACCAAGCGCACATTTTTTCGCAAGA

TCCATGAGTTGCAACAATACAAGTCAATGTGACGATTGCAAGCCTGCTACACGTATTTCG

TATTTATTGCTACACCGACTAAAAAAAAAAA

>1237

ACATAATGCCTGCCCTAGTACGATCCGCCAATTTTCCTTCGCGTTCATGTGGAGGGAGAA

AAGCAAAGTATTGCTCTGCTTGCCCATTCATTTCTTTGAGGTACGGAGCTGATGGAGACA

CCGTGATTCCCCGGTCTTGGCAATACTGCCAAGGCACGAGGCCTCTATGAGAGGGCCCGA

CATATCGGTGGTGACAACCGAAAAAAAAAAA

>1238

TATCCGAGAATCGTGCCGAAGATTTGGAGACCGGTGGGGCACAGTATGATAACGAAGGAC

AAGAAACGGCGGGTGTCCACCTGGACCAGTCATAGGAATGAAATCGTAGAGGTGGTCAGA

TGAAAGGAAATTAAGGTTTACCGTTGGGCAATGCTACAAGTATTCAAGCTTTAATTTTAA

ATAAAGTCGCACGTGTCTTCAAAAAAAAAAA

>1239

CCCAGTTCATTCAACAGACGTTTAAGAGCAGCAATTTGGCACCCAGACAACCTCCCAACT

CTGAGCTGCAAACGGCATAAGCCTGGCTGGCAGATCAAGCGCATGCAGTGCACACAGTCG

TAAAGGGTATGCACATGGTCATTATCATCCAACTCTGCATACTCAACTAGGCGAAGAGCG

TGAGTAGCGACAAGAGAAATAAAAAAAAAAA

>1240

ATTGGCATCAGAACATGTCGGTCCACATTTCCAACAAGTGTGAAAGTACTACACAGTACT

AGCTTCAAGTCACAACGTACCAGAACTCTATTGCTTTAGCTTTGTGTCTGATTGAGTGTG

GTAGTAAGCGACAACATTTTACTCCAAGCCAATCATAGTGAATATCTTGTGATGTTGTAA

AGTACGTTTAGACACTACTCAAAAAAAAAAA

>1241

GGGAGATAGAGATGTGCGGTGTGGTGAGCGTCCGTTGTTGACGTTTTGTCGTGTCGACAC

TCGGTAGTTTGTTGACGGCCCCAATGAGCGACTCTTCTTTAAGTGACTGCCACCTGCGAT

GTCGTCTTTAAGCCCGTTGATCCACAGGCCACGATGTACCGGAAGGCCAAAAAAGTTTAC

CCACTGGATCCCGCGTCCTGAAAAAAAAAAA

>1242

TATTTTTCCGGGATGATTCCGGGTTTTGGGGATCCCCTTTTTCCAAAACCCTTTTTTCTT

TTGGCCGGTGGCCTCCCCAAAAAAAAAAA

>1243

TTTGGTTCCCGGAGGAAATTTTTGGATGCCCCGGGGCTTATTGCGTGGACGGGTTCAACG

GGGGTCCGGGTTTTTTTTCGTTACAAATTTTTGCGGGGTTCGGAGCCTTTTTGGACAATT

TTGGGGGGATGGAAAAGTGGTTTCCTTCCATTGTGGGTTTCATTTTTTGGGTTCAAGAAT

TAGGTGGGGGGCCCAAAAAAAAAAA

>1244

AAAGACCCTGCGTGGTATTGACCCGGAGTACAACTAGTTTGCATGTAGACATCTATGGCT

GCGATCATATGCACCCACAATGAAAAGTTTGGACATGAATCCCATGAAAAAATGCCGTAT

CCATTCAAGGATATGGAACCATGTACAACATGTAAAATCAGTTAGCTCAGCTGAAAGCTT

GTATACATTTTAAGCTTGTCAAAAAAAAAAA

>1245

CACTGTTTTTGTTCTCCAGATGCATAGAGAGGTTTGCCGGAAACAGCAGCTTGAATGAAA

TCAACAGTATTGAAAATACATCACGCCATTCAATTACCCAGTCGATCGTTCCTTGAAAGC

GACGCCTTGCTGAGTTGCTGAGGGCTGAGCATGTGAGTTTTGACCGTGCACATGCTTGAA

GTGTAAAGTTCTCTCATTTCAAAAAAAAAAA

>1246

GGGACAAGATGCGTGCTGCGGCGATTCAAGAAATAGAGATGGACACCCAACGTGCACAAT

CTCAATTTGGCAACGGGATGTTTAGAGCCTGCGTGTCGTAAGTGCATGGCACATGAATAT

AATGAAAACTTGCAATGACAGGGTACTCTCCTCATTATCTGAGGCATAGCACATTATATG

CTCGTGATAACTTTACACTTAAAAAAAAAAA

>1247

GGGCTTGGTGAAAGACCAAGGGGCCCCTCTTTCCCCTTAGGGGGTTTTGTTAGCCATTAC

TCTCCCCCGGCCAAAAAAAAAAA

>1248

ATAGCCGCGATAGCCGACAATCAAGAAATATGATAGCTGTGCCACAATAGACATGTTACT

CGTCAGGTCAACAATACATGCTACGATATTCTTTCTTCGGCACTCTTGAAGGCGCTTTGC

GGATGATCCGCGAGGAAATATTGGACAAATTGCCTCTGACTGATCTATTTTGTCAAAAAG

TTTGTAAATAACTTATACATAAAAAAAAAAA

>1249

GGATCTTCTTTCTGTCTGCGTGCTTTTTGCATCCAGACCAGACTGAAATGTGGACTTTCC

TTGTCCAACCCCTCGGTGCGTGTTGAACAACAATGACGCTCTTGACTGGTGGATTAAATG

TTGCATGTTTAGTCGTGTATAGGAGGGCGAGGAGCCAGGACTTAATGGTGCGCCGGTCGA

ACGTGTGCATGTGTGATCATAAAAAAAAAAA

>1250

AGTGCAATCTCACTGGTTTTCTGGATTATCAGGCTCTTGGTATTGCACATTGAACAATAC

TATTCCCATCAACCACTACAAGAACTCAGAGCTAAGCAGCTAACCCGCCAGATTATTGTG

TTCGCACAATGAAAACTCCTCGGGCATACCTTGCCCCATGGTTTGACATAGATTAAAGCA

TATCACTCATAGCTAGAACGAAAAAAAAAAA

>1251

CTGAGCGTAATGACGTCCATCATCCAGTACGCCGCACCCTCTTCCCAACAGACAGCTATA

AACAAAAAGCTTGATGTTGACAGTGCCCTTATATGCCTGATGCTGCAGCGATTATTTTGC

AATATTGGAAAATGACTGTTGTAGATCTCTGAATCAAGGAATTGATATCTGGATGAGTTT

GTTGTAAGTCGTGCATATGCAAAAAAAAAAA

>1252

AACTCAGGCTAGTTGTCAGAGTACTAGAAAGATCCCATGGAGGCTAGTGATGTGCGATGG

CAATTACTCTAGTTAGCATCTGGGAGTTTAGGGTACTTATGGTTTTTTCGCGATGTGGAT

GTGTGAATCCTTCATATCCAGATGTTGTACCAGATAAATGTAAGTAATGAAGTTCATTGC

CATTGGTCGGCACGGCCTGTAAAAAAAAAAA

>1253

TGGCTTGCCTCCCGGAAAGGGAAAGTAGGGTTCCTCTTCAGCTAGACGAAACAGATGAAA

TGTTGTTCCATATAGCTTGACGCTTGAAGTTGATTGATAGTAGATCCCCTTCCGATGGGT

TGTTTTGATAAACACCCTAGGTAGGGCCCGTCATCATTTGCATAATTCCTGCCTAATATC

AAAATTGTGGTTTTCGGGGTAAAAAAAAAAA

>1254

CATCTACTACATCAAACAAACCATGACAAAGATCAAGATATGTAATTATTGCAAGAACGC

ACCACGCAGCCGAGTGCATCGAGCATGGCCCAACGAGCGTGGATGAGCTCAGTTTCGCGA

TATGCTCGGAAGGTTTTGAGGATCTGCAGAAAGGCCTGGGACATTTCATGGGCAAAAAGC

AGTGTTGGGATATCTACCAGAAAAAAAAAAA

>1255

ATCAGTTGTGAATAGAATGGACAACTGACAACTTGAAATCTTTGATCATATAGACCAGAA

CAAACGGCACAATCACTAGTGCGAAAGGCATAGAAGTAACCATTTGCGGTACTTGAGTCG

CTGCCTTCATCCCACTGTTTGCAATTTGTAAGCATGTACTTTTCGGAAAGGTTTGAGCAC

GTAGCAAAAGCACGTAGGTGAAAAAAAAAAA

>1256

GCGAGAACTCGTTGAACATAATTGTGTGGGCTGGCTGGACCACGTCGTGCATCTTGCAGA

TGTTGACATGGATTTTGCATAGGAGGGGGGTTTCACCATTGGGAGGGGGGATGATGTGTC

CACTGGAGAACACACTCTGTGAGCTGTCGCCTGTCATAGCATTTTTGCACCAGTGGACCG

GAAAAAGTAAGCTAGCATTGAAAAAAAAAAA

>1257

TGATTCAAATTCGTGTAGACACATACAACAGACACAAGCGTTTCCATGGCACTATACCAA

ACATTTAGTGATTAATCTGAGGTGGATCATTATAGACTGATGTTTGCAAAAGATGCCCTG

CACCCGTCAATGTCGGGCACATAAACGTTTGTTTCATGTTTATTTCACAATACGGCATCA

AAGAAGGAATACTTTGTTGTAAAAAAAAAAA

>1258

ATCTCCGGAATGTCGAATGAAATGGGGGAATCGGGCGAGGTCAGGGCCAATGGCAACATC

CTACAAATACGGCAACAACGACATAGCACCAGGTGTCATATGCGTACTCATGAATTATGC

AGAAGAGACAGGCCAGTCTAAGCCTCAAGAACGAAATATCGAGATTCCCGACAGAAGTGT

TATAAATAATGTGAATAATCAAAAAAAAAAA

>1259

TGATTCGGAGTTCGAAGAACATACGGGGAAGAGAGATATGCGGAGACGTTGTGTGTCATA

CTCCCTCTTCAGATCAAGCTCCCTGTTACATTCTTGAAATGTTTCAGGTGCGTGACTCCG

CGCCGAGTCGACCGACGAATGCAGCTTTTGGGGTTGGAGAACGACAGAGCTTTTCCCACC

CCTATTAGTCTCAATCTCTTAAAAAAAAAAA

>1260

TTTTTTGGGGCCTTTTTTGGGTTTTTTTTCCTGGTTTTCTGCCGGGCCAGGTTAACCCGG

AAAAGTGGGTGGCCTTTGGGCCCCCTTTTTAGGGTTTACCGTTAACCCCCCAAGGGGGCA

TTTTTTTTCAAAAAAAAAAA

***Klebsormidium subtile***

>1

TCCGGCAATCATTCTCATTCAAGCCTTCTGAGTTCGTCAAAGCAGAGCGGCAGCGTTTGA

GCTAGAGAAGTCATTTAGGACATCTTGCTACGAAGATTAGGGTTCGTCTTAATTTGCGGC

GGTTTTGGTTCAAGTCGCCGTTCAAATTTGGTAAGACGTCCTGGCTCGTAGAGCCTATAC

TAATATTACATGGTCCAGGTAAAAAAAAAAA

>2

CTGGCTGACCCCGCCGTCAACAATGCCTGGGCTTATGCCACCCAGTTCGCCCCTGGACAG

TAAAGATCTTGGTAGTGTCCTGTGCATAAAGACCATTAACTGTGTATGAACAAAATAAAC

GAATTTTGAAAAAAAAACAGGAGACATGTGTTGTCCCGCCATTAAATCAGTTTTCCTGAC

GACAGAAAAGATGTGGGGATAAAAAAAAAAA

>3

GGCGGCCGCCCCGGGCGGTGCAAGAGGAAGAACGCCAAGGCTGCCTCCAAGAAGGCGGGC

GGCGACGACGAAGAGGGCGAGGAGGAGGACGAGGAGTAGACTCAGCAAATGCCTCACCTC

TAGTTCTGTTCTTAGCCGGGGGAGTGTCAAAGACAGAGGGCACTGGTTTGTCGCGTTTCG

ATTGGATCTGTGCCGTTTGCAAAAAAAAAAA

>4

CGCTCTTAGAAAGTGCTCAAGTGCATTTCATCATGGCAGTGTGGGTTAGAGATGATATTT

TAGGACAGATTACCAAACCTGAAGTCACCCCGGCCCTTGCCAAAATGGCCAATTAAAGAA

ATGCTGAATTGAAATACATCAGCAATTTGATATCGCACTAGTCATCATCTTGTAATCGTA

ATTGAAAGAAAACAATCGATAAAAAAAAAAA

>5

TTTGTACAGAAGTAAAACTGTTTTTAGATTGCATCTTAGCAGGCCTTGGCTTTGGCTCTC

TGTAGGCAAAACTCTGGATCATTCTGGTAATGGTTGTTTTGAATTTGTAACCTAGCTCTT

CTGGTTTCATCAGGTAGAGCAACTAAGCATCCCTTGCCCTTCGGACAGTCTGAAAATAGT

TGCTAAGTTGCATTGTTGTCAAAAAAAAAAA

>6

GCCCCCCCGGGGGGGGGCCAAATTTTTTTTTTTGGGGGTTTTTTTGGGGTTTTTGGGGTT

CCTTTGGGGGGTTTCGGGGCCCCCCCCCGTGGGCCTTTGGGGGGGGGGGGATTTTTTTTT

TTTTTTCCCCCCCCCGGGGGGGGTTTTTGGCCCCCGGGGGGGGGCCCCAGGGCCCCCCCC

CTTTTTTTTTTTTTTTTCCCAAAAAAAAAAA

>7

GGGGCCCTCGGGGAAATAGACTTGGGGGGGGGGGCCGTTTGGATAACCCCTTAGACCCAG

AAGGGAGCCAGTTGTTGGGTTGATAAGGAATTTTTTTTGGCGGTCCGAATTTGGACTTGG

GTAAATCCGATTTGGGCGGGCGGGATTGAAAGGAAGCTTTTTGAGTTGAAACGTGGTTAT

TGGGGAAGGGGATTTTGAAGAAAAAAAAAAA

>8

TACCATAGCAGGGTTTGTCGGAAGAAGGGATAGAACTTCGAGAGATTTCGAGAGATGGCA

CCTGAAAGAGGCAGGTTTCGGTGATGGCTTCTCGGAAACTGTACGCACTGGTTGCTCAAT

AGGGACTGCAGAAAGCGGCACTGTACTCTTCAGAGATCAGCAGCTGTGTTGTCTGCATTC

TTCTGAAACTGAGTGGGGATAAAAAAAAAAA

>9

TCCCCTTGGAGTGCGTTTGTGTGGGCTTTCCTTGGGGGAGTTGGGCATGCCTGTTTGACT

TTTTGCCGGGGACTTGCCTCCCGGGGGGGGGTTTTGAAGGGTTTAAACCCGTCCCTAAGT

TTTGAAACCCAACGGTAACATGTTCCCTCGGTTTTTCCAGCTAGGGGGTGATCATATGTA

AGGGCTTTTTTTGGAATTTTAAAAAAAAAAA

>10

TTCAAAATCGTCAGATGAGCCGCTGTTTGCGGATGTACCATACATCATGTACGCAAGTTT

GACAAAAGAACCAGCCGCTCTTTGTAGAAGTACGGCCGTCACATTCCATAATCGTTGCAG

TCGTTTGCAGTAACCCCTTTCGCTGTTCAATCCGGTCGGACTAGCGTGATCACACGAGTT

AAGAGGAATTCAGCCGGTTCAAAAAAAAAAA

>11

GGGGCCCCCCCGGGGGGTCCAGGGGGAAGACCCCCAGGGTTCCTTCCAAAAGGGGGGGGG

GGGCCGACGAGGGGGGGGGGGGGGGGGCGGGGGTTTGGTTTCCCCAAATCCCTCCCCTTT

TTTTTTTTTTTTGCCGGGGGGGGTTTCAAGGCCGGGGGCCCCGGTTTTGTCGGTTTTGGT

TGGGTTTTGTCCCTTTTCCCAAAAAAAAAAA

>12

TGAAAAGGGGGCTTTAAAAGCCCCGGGGGGCTTGAAAGACCAAGGAGGATTTTGGGGCGG

GAACGAGGGGGTTGGGACGCGTCCTTGTTGTTTTTTCGGGGGGGGGGGAATTGGGACCCG

GTTTTTGTGGGGTGGTTCCCTTGGGGGGAATATTTTTGGATCCATTGCCCGGTTTGTTCC

GGGCCAATCCATGGGGGTTCAAAAAAAAAAA

>13

CCAGGTAACAAGGTTTCCCCTGAACAGAGGGGTTCTTGGTTTCGGGGGTAGGTATGGTAC

TTTGTTTGGAAAGCCGGTTTGATGTTTGCAGCCTGGCAAATGCCCTTGAAGGGGACCTTT

GAAGGCCGTTGTTTTGTGAAATTTTGGACCTCCCATTTTTTTAAACGAAGTTTTGGAAGG

TTTTTTTTTGTTGCCTGGGGAAAAAAAAAAA

>14

GGATTGAAAGCATGGGCGGTTTAGGGGAAGAGGTCGTGGCTAATGGGTATGAGAGTGAGG

GGGAGGCCCGGGGGGACGGGATTGTAGAGATGCAGCCTTACAGGTGACAGTGAGCTTGAA

CGGAGAGGATTGCCTTTCATAGACGATTTTGCTTGGAACAATATAACGTGTATATCTGTC

ATACTTACTTTTTTACCAACAAAAAAAAAAA

>15

CGACAATGAGTACTTAAGATAGATAGTCCTGTTGGGCAGCCGCGTTGAAACAGTTGAGGT

CATCTTACTCGGAAATGAATTCTGGACTATGGAAGTTCTAGCTAGACAGCTGGTGGACTT

CATCGGATTGAGGTTACCATCGGATCAGTGACATTGGTGACCACTTGTAGCCTCTTATCG

CAATGGGACTACAATGATTTAAAAAAAAAAA

>16

AAGTTTTGATGGGCGGGTCGTCCCATGTCGCGATTTTTGCCCGTCGTGGGGTTTAGCTGG

GGTTTTGCCCGGTAAATGGTGAAAGCCCCCTTTTGTTACCCATGTGAAAAGTTTCTGTGC

CCGTTTGTTTTTGTCTTCCCGTAAAAAATTCCATTTTTTGAACCCATTGTGGAATGGGAT

TGTTTGAATTGTTCGGTGTTAAAAAAAAAAA

>17

ATCTGGGTACAAGAATCCAGCTGGAGTGTTATAGTAGTTGAATCGAAGATAGACGTTCAA

ATGCATACTTCGTTAGTAGAACTTCTTGAAGCCCCATGAAACTCTGAAGAGGGCCGAACC

AGCAGAAGAACATGATTAACACCTTGTATGTTGCTTGCTAGCTTGCCATTAATGACTGGC

ATTGTTAACCAGTTGCTGCCAAAAAAAAAAA

>18

GTGAAGGACGAGATCGTCCTGGAGGGGAACGACATCGAGCTCGTGTCACGGTCCTGCGCG

CTCATCAACCAGAAATGCCACGTGAAGAGGAAGGATATCAGAAAGTTCTTGGACGGGATC

TACGTGAGCGAGAAGGGTATCATTGAGGTCGCCGAATCGTAGCCAGGCAGTTGCAAGGAC

GTGAAACTTTCTCAGAAGCCAAAAAAAAAAA

>19

ACAACATTCAGAAGGAGTCGACCCTTCACTTGGTGCTCAGGTTGAGGGGTGGTTAGTAAT

GATTAGAAGGATAGTCGTTGATGTGGGAGTGGCCATCTTGTAAATGAAACACTGTACATT

GTGTAATGTCCGAATTATTGCTGCAATAACTTGGTCCCGCAATTCTGTTGTTCTCGCGCA

ATTAAAAAAAAAAA

>20

TTTCCCAAACTTTACGCTCCTTCTTGGGGGGTTGTCCGTTGCCTCCCGTTTGTGGTTATT

CAAGTTGGGCGGTTGCCTTTGCCTTTGGACGCCCCCTTAAAGTTAAGTTGGGTTCTTTTT

TTGGTCAAGATTTTGTTGTTGATCAGCGCAGCGCTGATTCCAAGGGTTTTGTGAAGGGCC

TCCATGGGGGGTGTTGTTGGAAAAAAAAAAA

>21

GTTGCACGTACGTGTTCATGGATCGTCAGCAACAACGTTGCTGTCGTCCGCGGGCTGTAT

AGGGTGGTGCAACGCTTGGGAGGAGGCCTGTCTCGTCTGTCGTCTTCTCGACAGGCGAAA

AGACGCCTTCGTCCACCTTGGTAGCTTTGCGTCCGAAGAGAGATACAGATGTAGACAAAT

CCTGTAATGTTTTCCCCCATAAAAAAAAAAA

>22

TGTTGTAAGCAGTTCGAACGAGTTGGACTGAAAGCAGCTAGCAGCCAACTTTGGAATTGT

GGGCTGTATTTATGCGAAACAATCTTTCAGTGTTTACGGATGAGGGATTGGTAGTCAATC

CTTAGAAAGTAGTATTCTTTGGCAAAGCACGCAAACACTCGTGTATCATAGTAATCGTTC

TGGAATGAGGAATTGTTTAGAAAAAAAAAAA

>23

TCGAGTAGGATAGGAGGCGTGTTAGGAGGTGGGTTGCCCCGGCAAAGTCGGGACAAGAAA

CAATAGACGCCAATGTCAAGCGATGGTGGATTTCAGTAGTATCCACTCAAGTAGGTCTCT

AGGTTATGATAGGTGAATGAAACTGATTCGCTGTACTGTTGTAGACGCTGACGACTATGC

TGGTGCTCTTGTTTATGTTCAAAAAAAAAAA

>24

TAGGTGGTAGTTGGCATATGCGTGGTCTAGCTTTCAGACAACGTACTCCAGCATACTGCA

ATAATAAAACCATACACTACAAAAAAAAAAAAAAACCAGATAGTTGTTGTGTAGAACTCG

GCCCCAACGTCCCATATACACAATCAAGCCCCATGCTTCGGTACGGGCTTTGTCAATGAA

AGATATGTATGCTTGACATGAAAAAAAAAAA

>25

CCAGCTTCCTCCATGCTGGGGATATCTGGAATGGTGGACCCAGGCATGCTCATTGAAATA

GAAGCAGATGCTGTGATATCAGACTAGTTTTAAGATTTTAGGACAAGTCGCCCGGCCCTG

AAGCGCAGCTACATGCCGAACTAGCTGCCAGGCTGCGTCGTTGCACGCAGATTTATGAAA

CGTGGAACCTGGTGACTCTTAAAAAAAAAAA

>26

AGCACCACCCAGTTCCACGACAGCAAGATCAAGTTCCCCCTCACCTACCGCTTGATCCGC

CCCTCAAGCAGGAAGCTCAAGACGACGTACAAGGCAAAGAGGCCGACCACATTCATTTAA

GCTGGTTGGACAGACCATGTTCGGTGACTGTAAAGGGTTGCTTGTCTTGTGTAATTCCCG

GAAGCCATCAAATGCGCTGTAAAAAAAAAAA

>27

GGGTCCACCCAGAGAAACGGAAGGTCCAGCCTGTTGCAAGCGGCAGCTTATTCTGGATCA

GTTGAAGAGGCGCAGGCACGTGATGGATTACCTGTAACGTAAGCCGCTCGCTGTTGCATA

ACAAGCTGTCAGTTGGAACCATATGCGACGCATTATATAAGAGACTTTTAATGCGGACTG

GCGTCCAATTCAAATTGAAGAAAAAAAAAAA

>28

CGTTCAAGCTGTCGGCGCCCCTCGGCGGGCTCAAGAAGAAGCGGACCCACTACATCGAGG

GTGGGGATGCCGGGAACCGGGAACTCAAGATCAACAACTTGATCAGGAGAATGAACTAGG

GCGGATGGACGGTGCTAGCGGGCTTTGCGTTCTCGCGTCGGGGACGCGGGGCTTGAAAGA

AGATGGTTGTGTCTCCCTTCAAAAAAAAAAA

>29

AGTAGCTCTTTCCAAGTACTGTTGGGTTTGTCCAAGCGGGATTGTAGATGTAAGCTGCTG

ATGTGGCCAGATCTACCCTGCTGGATTCGACTTCTGGTCAATGCCTCGACTTGCAATTAA

CGACAACTTCTGCTAGAAAAAAAAAAAAGTGTTGGGACAAACTTACAACCGATTTTCGTC

GAATGCCCCAAAAAAAGTCCAAAAAAAAAAA

>30

GTTGGAGGTAAGGTTCTTTACGGTAGCGTTCAAAAAAAAAAAAAAAAAAAATCGTAGTCG

CAGCATTCACAGCCCTATCCCCATGAAGCGAATGGCTGGTGTACGATTACATTGAACGGA

TGAAGGTCCAAATATCGTCGAACTTAATACCCAAGCTTTGTGATCAGCTGACTTGAGGTT

GATTCTAGATATCATGCATTAAAAAAAAAAA

>31

GCGGCCAGAGCACTTGTTGGAGTCAGCGGAAAGTCAGAAAAGGCTTCAGATGTTTTCAGC

GTGCCAACTGCAGTAAGATGAGTGCATCAAGGGTCTGCAATCAGCAGCCCAATGAAGTAA

AGCACGCGATAAGGCAGTCAGCTAAAGACAGACTGCAATTGGACAGTCATATGCTCGCTC

AGTCAGAGTTTTGCGATAGCAAAAAAAAAAA

>32

GAGCGTTGTGTCGATATGCATCCGTCCTCATGTGAGAAGATCCAATAGCTAGTACTTAGG

GGCAGTAAGAATGAAAGCATCCTTTTCACAGTAGTCTCTACTTGCCCATCTTGTTGCAAT

AGAGTTACGTTGGACGATGACGAAGGACAATGGTCTTCCTCAAAAGGTACCTTGCAGATC

TGGCAACCACCAGTTTTGGCAAAAAAAAAAA

>33

GGCACCAAGCCGTGGGTCAGCCTGCCCAAGGGCAAGGGAATCAAGCTGTCCATCTTCGAG

GAGCCCAGGAAGGCGCGGGCGGGCGCTGCCAAGGTTGCGGCATAAGTGACGGTTCAGCAG

AGAAGATTGGCATGGGATGAGGGACAGTTGTAAATGATTCGAAAAGTCTGTTTGCAACCC

CGTGCCCTGGAGCATGTTGCAAAAAAAAAAA

>34

AGAGCCATGATGAAGCGAATGCCTGGTAGACCTTCAAAGACAAAATACATCTATCAGGAT

ACAGATTAGTGAATTGGTTTTCTAGAAGAATGTAATTTGATCCTTGTTTGCATATTAGAG

TAATGCTTGGGATTTGTATCGAAGGTCAATGCCGACCTTGAAAAAACATTTATAACAGCA

AAAAGAATAAAAAATTATATAAAAAAAAAAA

>35

ATCAGGCTGCTCATCTAGTGACACGGTCTCAAACCCAATTGGAGAAACTGAAACGGAAAA

AAAAAAA

>36

CAATCTTAGTAATAAGCATACGAAATCAGAACAGAGTGATTGGTGCGAAAACAACGCATA

ATTGACCTACTTCTATGATAAAGATGGATTGTAGGTTTGAACATATAACAAAGGTCGTGC

TTAAAGTGGTTTTGACAGACGTGTAACAAGCCTGATGTACATTTTGACAACCTCTATCAG

CAGCTTCGGCAATCTCCTTGAAAAAAAAAAA

>37

GACCCCCCCACTAGGCTCCGCTCGCTTTCAGCAAGAGACGTCTTGGAGCCCCCTCTGTCG

CTCGGGGGAGGGGGGGCAACAAAAAAAAAAA

>38

TGGCGTGGGTTTGAAAGGACTCGATTGAATTTTTAGGGAGGGTTTTGTTTTCTTTTTTCC

AAGGGCCGCTTTGTTTTTCAAGGGATTTTCCCCCGGGGGTTGGGATGGCAATTTTGGGGG

ATCCCGGCCAATTTTGCTTGAATCAAGTTGACCCTTGTGCAGTTTTTTCGGGGGGGTATT

TTTTTTCCCTGAACTGCTTTAAAAAAAAAAA

>39

CTCCTATACCCCACAACCGATCCCGGCCTTGAAAAGCTAGTTGTGATTCCGGTTCGGAGC

AGAGAGTCTAAAGGCGCTTCTCACAATGCATCAGACCTGCTCTCAAGCCTACTGCACTTC

GGGTTTGCTGAGAGTCTGAAACTTCACTTGTATTCTCGGACTGCTACATCTGTACTGTGC

TCTTGGGGGGCTTGGTCGTTAAAAAAAAAAA

>40

GTCTACCAGGCATTCGCTTCATGGAGGTAGGGGAATCTTTGTATATTGCGTTGCTCAGGT

GACAACGTCATCCTCTGTTCTGTACGTAAAAAAAAAAA

>41

ATGGCAAGAAGCGAGGTGATGATACATACCCTCCGCGAACTGCCAGACAGAATAGCAGCC

GCGGTAAATTCGCAGTTGGCAGGACCGTTAGAGGCGATCGCTACAGTTTTTAGAGAGAGG

CGGTTGTAAATACATTGTATATAAGATCAATGTTGGAACACAGGACATATTGGAGCCGAC

ATGCCTGTGACAGTTATCCCAAAAAAAAAAA

>42

CTGACGGATGAAGCATATAATCGTGGGAGTGCAGACAACATAACTTGTGTAGTAGTCCGG

TTCTTGCATTGATTGCTTCTTGAAGTTGCGAGCAAAGCAAGCCTCTTGGTCCTGTATACT

TGTAGAATGAACAAACATAGTAAACTGAACGACCCCAGCTCAAAGACGGTCTCACTAAGC

GAAGCCAACATTGCATTCTTAAAAAAAAAAA

>43

TGGGTAAAAACCCTTGAAAGATCGAAGGGTTTCCGTTTTTTTGCTCGCGGGTGGGGGGGC

TTTTGGGGGCTTTAAAATATGTCTTCGTGTGGGGTTTTGGTAAAGCCTTTGGTGGGGTTT

CGGGCCCCTTGGGGCCCCGTCCTGCCGTTTTTGTTTGTTGGCCCGGGCCTAAGAAAGGTC

AATTCCCGCCCTGGGGTCCGAAAAAAAAAAA

>44

GAGAGTGCCGGTGTCACAGTCAGTAGGTCGCCCGCAAAGCTCGGTACCACCATGCTGCAA

CTGTTCAAAGATCGTGGGTTGCTCAAAGAGTAGGCGGATGGATAGCTTCCACTGATTTCC

TGCGTTGGAGCCCCTGTAGCACGTACGTTTGTACATGTTTTTAGCGCATCGAATCTTTCA

ACCCTTATATTGCCCATTTGAAAAAAAAAAA

>45

CAGAAGTGACCGAGGGCTACTCAAACCTCGGCACCAAGATCGTTGAGGATGACGAGTAGG

TTTAAGCTTTTGGGTTTGACGAGAGGGTTGGGGAAGGTGCAGAACCAGGAAGCGGAGACT

AAGTGCTAGGCTCTGCTTGCAGTGGGCTTCCTACTATCTGGACACAAGCACTCTTTCTCC

GCATCCTCATACCACTTTTCAAAAAAAAAAA

>46

TACATGGCGCAGATGCACCCTCTAGTTGTCCCTATCCAAAAAGCAGAAGGAGAATGAATA

TGGTATAAGGAACATTCGCTACAAGTTGACAGCTCTTTGTACATTTAAGCAGAATCGGTA

TGCAATCAAAACGTTTCCACAACATTTATGAAAAGGGGCCTTTGAATTGAAGCAATCAGT

TTTTACGTAACTTCTGCAAGAAAAAAAAAAA

>47

ACCGTACTAGCAGTCCCAGTCCACGACGAGAATGGAGGTACAGGAGTTGGTGCTCCGAGT

TCCTAGGTTAATCAAGTCGGTAAGGAGTTGCTCAGACAGTCAGGTCGTCGACGCAGCTTG

GATCAAGAACTGCGGCATTTGTCATGGGGATTTACTGTAGCCTGACTGCCCTTAACTACT

TTCCTCTTTTGGAAACTCTGAAAAAAAAAAA

>48

CGCAGCCAGGAGGACGAGTAGACACAAGCACCCATGTACAATTGTGATCTTAGCTCTCTA

CCTCGGGAGGGCCGCGTTAAAACCACGTTGCGTTATTTTCAAAAAAAAAAAAAAAAAAAT

ACCATGACTCTTGCAGTCTTACATATAGATCTTATATGATTCCTTGTCCTTTGGATGTGC

TGTGATTGTAGCATTTCTGTAAAAAAAAAAA

>49

AAGATTGGCAGGATTGAGGACGTCACTCCCATCCCCACCGACAGCACAAGAAGAAAGGGT

GGACGTCGTGGACGTCGTTTGTAGTCAGCCTACGCCACTGCCTCAGGGCAACCGTGGGCA

GAAGCTCGGAAGGACCGCTTTGGGGTTGTACCTTCAGCTTCTGGCTATCATCGTCCCTTT

TCAACATCAGTTTTTGAACTAAAAAAAAAAA

>50

GATGTTGCCTTGGCAGTGTCAAAAATGAAGGAATTGCCTGGGTAGACAGACGTCACGAGC

AGCTACGAGAATTTTTTGAGGAGAGCGAGAAAAATTGAGAGAGTTAAAAGGATGGGGAAG

CTCCGAAGCAGATATAACTCGGACGCCCAATAGTGGGAGCCTCTCTGTTCCGACCTGATG

CACATGGGTGTCCATGAACGAAAAAAAAAAA

>51

CCGGAAGGAAGCACCGGGGCCTGCACGGAAAGGGCCACAACTACACCAAGGTCCGCCCGT

CAGTTCGCGCCACATGGAAGACGAACAACAGCCTGTCTCTGCGGCGCTACCGATAGAGTG

GTTAGGCTGGTCGGTTCAGGACTTGACATCATGGTTTGCGTTTTCATGTCCGTTTGGGGC

GCCAGAGCATCTCTCACCACAAAAAAAAAAA

>52

AGCACATTTGTTGCTTGGCAGCAGATGAGCAACCGTGGGCAATAAAATGTGGTTAACTCT

TGGCAAGGGTTCAAGCTTGTCCGGCACCAACGAATAAATTGACTGTTTACGCTTGTCCAA

TCATGTGGACTTAATATCGATCAGAGACTGGCAAACCTGCCTGAAAGGCGTGAGCCAACA

ATTGGGCAGCGGTTTTAATCAAAAAAAAAAA

>53

TGAAGATCAAGTATGCCTTCGTCAACTGCTGATTGAGCTATTGAAGGTTGGAGAGGGCGA

CCTGGGATCTGTTGCAAGATCCGCTCATCCGGCTCTGCCAGTGTATACTTATAAGTCGCC

CCTTGCAGTGACTGTTTGAGAGCTAACCTGGACCCGGCATCATTGAGTCTCAGTATATCA

AGCCACTTAAAAAAAAACCCAAAAAAAAAAA

>54

GTTTGATAAGGCTTTTGTTTTACTCGTTGTATGAGTAACAGCCAGGTTGCGTGTCCATGA

GTTGTCGGAGTCGTTGGGTCATAGACTGAAAGAGTACAGTGCTCCATTCTTTCTCAGCGA

TTGGCAGTTGCAAGCAACATAAACCTTGCACCTCATCTATTGTTCATATGTTGCTGTCTC

CATAAGCAACGTTGCGTGTGAAAAAAAAAAA

>55

TTGTACTGAATCTGCACCGTCGATATTGAGTTAGAGTCGACATCGAGGACTCTATATTAT

CGAGAGTCTAGTGGATAGTACTAGAACCTTCTAATGTAGAAGAATCTAGAAGCATTGAGG

CTCGGGCAGTGTCCCAGAGCAACGTAAGAACTTCAAGAGACAATTCTGATGGCAGAACAA

GAACAACTTGTAGAGATGCTAAAAAAAAAAA

>56

TCGCATTGAGCCGTACAGGTGCCGTAATTATAGTTCGATAGACTTGTACCGGTCGCAGGT

GTTAGAAGCCCTAGAACCGGACCTCGCCCAGTCCCGTATATTTCGGCCGCCGGTAGGAGT

GACGTCATCGTACATTCACGCGCTTGGCGCTGTTCCGAAAACTGTGGATTCCGTACGGGT

TATTCACGATTTAAGCCGTCAAAAAAAAAAA

>57

GATTTAGAGTTTATTGATAGTGTCTGCTTTCTTTTGAGTACTGTATTTGTAACGTGAGTC

GTCACAAAAGGTCACGTGTCAAGCAACTTCTTTTGACAGATCTAGCGCCCGGCCGCTTTG

TAGGCAGCTAGGGCGTCTAAGCGATGTTTTGATCTACTAAGTTGACCTCTCATTGAAGAC

TAAGTTCATGTAGACAGTGCAAAAAAAAAAA

>58

TGTCCCCCAGCTTCCTCCATGCTGGGGATATCTGGAATGGTGGACCCAGGCATGCTCATT

GAAATAGAAGCAGATGCTGTGATATCAGACTAGTTTTAAGATTTTAGGACAAGTCGCCCG

GCCCTGAAGCGCAGCTACATGCCGAACTAGCTGCCAGGCTGCGTCGTTGCACGCAGATTT

ATGAAACGTGGAACCTGGTGAAAAAAAAAAA

>59

GTGATATGAAGAGATTTAAAGCTAGTGGACGGCACTGTGCAATATCACAAATGTAGACAG

GCTGGACAGCCGGAGACGTGGCTGCGCTCAGCTTTAGTTGAAGACGTCGGCGTAGTGACC

TTGTGGATATGCGCCGCGCGCTTCAACTTAGGGCCGCGGGTGAAGATTGAGCTGCCGCCC

AAGATGCGCCATGTGCACGGAAAAAAAAAAA

>60

GGGTTTTTGCATAGGCAGCATTGCACACGTTTCGTGGAGAGGTGGCTATAACGAGTTGCT

GTACCATATTGATAATATAGGACGCGAATACGGATAGCAAATGGAAGTTGTGCTTCAAAG

CAAAGAGCATATAGAGCACGAGTTGATTGTTGATTGTTGTAATTAATTGTTCAACACTAG

ATGCTAGATTGACGTCTTTTAAAAAAAAAAA

>61

TGTCCACTTACTTATGTGTTTCTGTCAAGGTTCGATTCTGGACGAGATGGAACACCGTCG

CATGAACGAGCTCTTCTTGGCTGTACAGTACTCCAGTGAGCTAATTATACATATCATTCA

GCCATAGAGATGAAACAATTAGTTAAAAGGTCGCCGCTTCACTTTGTACATCATTGTGAG

TATGAGCTTGCAGTGTATTGAAAAAAAAAAA

>62

GCCAAGCATGTTATCTGTGTAGCAGCTGTGGATCGAGTTGCTTGTGGGTATAGTGGTTCG

CCACTACAGCGTAGCATCTAGACTATGCATCGTTAGTACACACATTCTTGGTTTTTTAAT

GTTATACAACAGTACTTCTTGTCAAGTGACAGTCCTATACGACAGCTCCGGGCCCAGGCA

ATTTACAGATCGGAATCCTTAAAAAAAAAAA

>63

CGGGGAAAAAAATGGGTTTTGGCCCCCCGGGGGGGGGGGGGGTTTTTTCGGGGGGGGGGG

GAATTTTTTTTTACCCCCCCGTTTTTCCCCCCGGGGGGGGTTTTTTTTTTTTCCCCTTGG

CCCAGTTTTTTTGGGGGGGGTTTTTTTTTAGAAGGGGGTTTTTTTTCCCCCCCGGGGGGG

GGGCCCCCCCCCTTTTGTTTAAAAAAAAAAA

>64

GAGTTGTGCCCGAAAAAACACCTTAATAACAATAACGCCACATTTTGTCATCAACCTCAA

TTTTGGTCCTCAGCTTGGAAGTTTGTTAGTGCAATGGTTAAGGTCCTCGGCTTGGAAGCT

TTGTTTGTGACACCTTAGAATTGGGTAAGGACAAAAGGTTACTCTGATTTGTCATTGGTT

GAATATGAACTAATACACACAAAAAAAAAAA

>65

GCAGCGGCAGCGCTTCAAAGAATTGTGCCTGAAGTCGTTACCTTTGCATCTGGGAAGAGA

TTGCACGAGTTGTGACCTGGGGTCTTATTCTTGTTTAGTCATTAGAGACGCTTTGATTTA

TAACAGATTGGTGCGATTAAGTCAAGACGCGATAAATGCTCGTGTTTGTGGCCGGGTTTA

ATGGGCTGATGGCTTGGGGCAAAAAAAAAAA

>66

GAACTAGTCAGACGTGCCTGTTCTGTAAGTCGCAGGGTTAGCTAACGTCAATAGGATAGA

CCAGAGGACGCCAATCAAAGCAATGGGTACATACTCCAGCTGTACAATCGCGCTAGAGTA

GATCCATTCAGAGATTCAAGACGTCCGGTGATTTTGTAAACATTGAGTCCGCAAACTTCT

TTCAAGGGTGTGTGAAGGGGAAAAAAAAAAA

>67

TGGCGCAGTTGCCACCGGCGGGGGTGGTGGCGCTGCTGCAGGCGGTGCCGCTGAGGAGAA

GAAGGAAGAGAAGAAGGAGGAGAAGGAAGAGGAGGAAGAGGATGAGGACATGGGCTTCTC

CCTTTTCGACTAGGCCTGTTCGGAAGAGCCAGAAAGCTTCAAAAGTTGTACATGTACCTC

ACTGGAAAGTGCTTCCACTCAAAAAAAAAAA

>68

CTCCCAGTACAAATAAGTCAGCAACGTTTCGTAAGGGAAGAATCATACGGATGGGATTTG

CGCCACTCTGAAACTGAAAGTCGTTTGTACAATACCCAGTTGGCAAAACTTCTGACAAGT

TGCAATGAAAGAATACGAGATTAGAAAGAACATTCGCACGCTGATCTGTACATGAACGAT

ATAAATCTGCATCACATCTCAAAAAAAAAAA

>69

TCATGGCCAACCACTTCGACCGCCACTACTGCGGCAAGTGCGGGTTGACCTATGTGTACC

AGAAGAAGGGCGAGGAGGCAAGCTAAATTCGCTGAAAGTTGTTAGATTTTCCCATACGAA

GTCAGCTTGAAAGGCTGACGTTGTACTGGAAAGCTATCCGCTTCAGCGCATAGCCAACAA

GCATGTTTCTGCATGGTTATAAAAAAAAAAA

>70

AGTTGCCTTACAGGGAGCTCCCCCAATACATTTGTAAGTAAGAGTCAACCGGTGCTTCCA

TTAATGGCTGTGCCGAGGGTTGTGCTATCGCGACAGAGGTAGGAATCGAGGGTACTAGCC

TTCTTGCAAATGCACTGGTCGGAGTCATGTTCTGCCATATGCTTTGAATATCACTCTATC

AAAAATCTGGTGTTTGGATTAAAAAAAAAAA

>71

AAACATGGTGCAGATCGAGGCGCCGGCCTTGTGACACCTCACACGGCTTGAGATGCTCGT

GTTGGGTACTTCTTGCACAACTGATAGCTTCCTACTTGTACGATCCATACTCGAGTTGCA

AAAGCAGACTCGTTGAAGAAACCGCAAGATTCTGATCCTTGTACAGTAAACTTCGATTTG

ATTGGATTTTCCTGACTAATAAAAAAAAAAA

>72

TTGACGACTGATAAGGGGCGGGCGGGTAGGAGTGTCGACCAGTGTTGTACAGAGTCAAGA

GCGCACGAGTATGAATGTCGATGAACGTCTGCTTGCGCATAGTCCGGTAATCTAGTCACT

GAACACTGCCAACGTTGAGCAGACCAGTTGTGTTTGATCATTGGACACACAAGAGTATCA

AGGTGATTTTCTCAAAAGCTAAAAAAAAAAA

>73

GGGGTTGTTTTCCCGGCCTTTGCTTTCTGGGGGGGGGGGGGTTTTTTTTTTTTTTCCCGG

GGTTTTGGGGGGTGTTGGGCCCCCTTTTTTGGGGTTTTTTTCCCTGGTACCCCCCTTTCG

GGGGGGGGGTTGCCGGCCCCCTTTTTTGGGGGGGCCCCGGGGGGGGTTTTTGGTCCCCTG

GAAAGGGGTTGCCCCCGGGGAAAAAAAAAAA

>74

CCCGCTCCAGCCCTCCAAGCCTGGCCCCCGCGGCAGGCTCTAGATCAACAGCCACAAGAC

AATAACTTGAAGATTTTCACCCACTGCCCGGTCGCATATGATTCTTCGAAGTGTGGCATA

CATTGTACCCAAGTGTCGACAAGTAAATAGTACATTGGTTACTATCGCAGCATTCACAGT

TGCTTCTATTCAAAGCCATCAAAAAAAAAAA

>75

CAGACAAATTGTCTTTTGGCACCTAGCTATCCTCCGCCCGCCGCGCCCGAGAATATCTGT

GCGTAGCATTGACAATCAGATGTACTTGAGTACGGCATTGGGCTTAGCCACAGTCTCTTA

CTTAGCCAATTATACTCAGAGAAAACAATTAAACAATTAACACTTCGTAAAAGAATGTGG

CTTAGCCCGCACTTACCTCCAAAAAAAAAAA

>76

CCTCCAGAACTTGGCTGACCACCTCGACAACCCCACCGTCAACAACGCCTGGGCATATGC

CACCGCCTTCACCCCTGGCCAGTAGACTTATGTCTACACAATTTTTCGAATTGTAGTATA

CTGCCCCACCGAACAAGCGCAAGCTAATTAAATAACAAGATTCATTGACCTTGCTTTTGG

TAATAAAAGCGGATCCGGATAAAAAAAAAAA

>77

GTTGCCCCGTTACCGACGGGTTGACAATTTCCACATTGTGGAGAAGATCTAGGTCGATCT

GCGTTGATAGTGCAATCACAGCAAGTCTTTTTGGACTTCTATCGTAGATTGCATATGCAG

GAGTAGTACGGGCGAGCGACTGACATTGCTATCGTTGTTACTCTAAGGTCCATTGCAAAT

GAAGCGAATGCCTGGTAGACAAAAAAAAAAA

>78

TAGATAAACACGACTCTTTGAGCACGGAGTTGACCAGTTCTGTTTCTCGCTCTAGCACGG

GTCATACAGCCAGGTCGTTTGGCCCCCCCTAGATGCGAGAGATTAGCACAGCAAAGGATC

CGCTGTTGAAGCTCGGCACATTTTGACGAGTTGCTTAGGCTGCTGTGAGAATCCAACCTG

TCAATAGAATCCTTTTACTGAAAAAAAAAAA

>79

GAGGGACTCCCAGAGCTTGGAGACTATTTTTCAGAGCAGTCGTCTTGCCGTGTGGTAAGG

TAGACAATACAACGAGTCGTGTTCTGCATGTGTGATATAGTACAAGCTATACAAAAAGAT

TGAGACAGTTTGGCTGCGCAGCAAGACAAATAATTGACATTGCTTTTGGTGTAAAGCTGA

GATGCAACCTTTGAGTTGATAAAAAAAAAAA

>80

TACCCGGGCACGCCATCCGCTTAAAGAGCTACTGGTTCCTTTTGGCCAAAGTTGTTAAAG

ACTCGTCTTTTTGCATGAGCCCCAACGCGTGCGCTCGTAATCGGCGCCATTGGCAGGGCT

GCTGTTTTTGAAACTTGTGTGCAGCTTGTGCTTGTCCGAGTACGGAATTGGATCACAAAA

AAAAAAATTGGCATGTGTGCAAAAAAAAAAA

>81

TTTGAAGAGCTTAGGGATATTAAGCAAAGGAAATGTTAGGTGCCATACTATGGATGAGGC

TGACCTTTTACAAACACAACACGTTACGTTCTGGACCACAGAAACATGTAGCCACATTGT

CTCTAGGATCGTGTAGACCTAGCTTGTAGACCTCTTTGTCAGCTCCCCTTCAGACACACG

TCCAGCCATTTTCTCCCATCAAAAAAAAAAA

>82

AGTAGTTGTAGGTTATATTGGATCTTGATAGACAAGACGTGTCGGGTCAGTTACTATTGA

TAGGTCGAGCAGGAAGAGACAGCTGTTGCAAAGAATAGTACAGACTTGGTTGCACAGATT

TACGCTCAGTCCTTGAGGATGTCAGAGAAAGAATTTTTGTTGGACCATATCATTCGACGA

GTATGGTCAGTTAAAACGTTAAAAAAAAAAA

>83

TTAACCTGACTAGACCACTATCGAAGTGGTTGCTTGCGTGTTGACTTTGTGTAGAGTCGT

TGCTATTGACGAAGTGTAGATAGCCTAAACTTTTGTGTGACGCAAGTACATGTCAAAAGT

TGAATTACGAAGAGAAGGTCGGACTCAAAGGTGTCCTGTTTCTTTGTCAGAAGTGGTGAT

GTCAGCTAGTTGCATTTGCTAAAAAAAAAAA

>84

AGTTGTTCTAAGTTGATCGTTGTACACACACTGCTTTGCGACCTGATTTTTTGGAGAGCT

CATAGTTGTAGGGGGCATTGTGAGAGCGAGGATCTGACGATCAAGCAATGTTGTACTTAT

ATGGATGATCTCGTAGAACACTCCACGTGTCAACTTCAATTTAGGCCCTTAAAAAAAAAA

AAGTCGTAGTCGCAGCATTCAAAAAAAAAAA

>85

GTGCGGCTTATGATGCGATAGTCTTGTGAAAAGATGCCCATCAATTGCTTATCAATCAAT

GCTGTGTTTGCTTAATTAGTGTATCTTTTAGATGTCCGGTCTAGAACATCAGCTGTAGAC

TGAGTCAGAGTATAGGATTGACGTTCGGCAATCTCTTGTAACGTATCTCTTCCAGTTTGC

CAAAGATAGATCTCGAAGTCAAAAAAAAAAA

>86

AGAAAAGGCGCGAGCCAAGCTGGAACGAAAACAGTCAGCAGAGAAGGCAGCTGGGGAGGG

GTTGTTCGGTCGAATAAGCAAGATGTTCTGGTAGTGGGAGGAGCACACGATAGTCATTGT

CATCTTTTTTAGCATCTTTCATAAGCACAGTCGATAAAGCACGCACGCTTGACTCTCGTG

ATCTGGTCATTTTTTCAATTAAAAAAAAAAA

>87

ATAGAGAGAGTGAACAAGACGTTGCTAGATCACGGGCCAGCACACTTGTTGCTTGGCAGC

AGATAACTAACCCTTGGCAATAAAATGTGGAAAACTTTGGCAAGGCTTCAAGCTTGTCCG

GCAGTAATAAATAAATTGACAGTTAACACTCGTTCAAAAAAGCAGACAGAAGATTGATGA

AAAGCTGGCAAGTTTAACCTAAAAAAAAAAA

>88

TGGGCAGGTCGTCACATGTCGCGATTTTTGACAGTCGTAGGGTCTAGCTAGGGTTCTGAC

AGGTAAATGGTGAAAGACACATCTTGTAAACAATGTGAAAAGTTACTGTGACCGTTAGTC

TTTGACTAACCGTAAAAAATACAATTTCTTGAAACAATTGTGGAATGAGATTGTTCGAAT

TGATCGATGTTAACCTGTTCAAAAAAAAAAA

>89

CCTTTTTTTAGATGCTTCATGACATATACATAGATTGACCTGAAGTAGGCAGTTGTGGGA

CTCAGGGGTTTTGTGAGATACCCCCCGTGATAAAGGCTTTGAATACAGCCCTAACCTAGG

GTAGTCCTGAAGCAATGAAGTCCCCCGCCCCCTTGATTCGACGCAATGGATTCAATTTTT

TTGAAGCCTTAAGAACTGGGAAAAAAAAAAA

>90

CGCACCCACTCAAATGCACATGTAGGCCTGAGGTGCAGTATAATGTCAAGCTCGATGTCG

AAGTGACTAATTGAGTGGGCCTGCATTGCCGGTTGGCCGTGCACTAAGTGCTCAGAGCAC

AAGCTTTCCAGTTGGACAGTTCCGTCCGGTCAGTGCCTGCCCACATTGCTTGCTAAGCAC

CGCTGCAACCTGTCCGCCTCAAAAAAAAAAA

>91

GGGGAGAGCCTCCACGTGAGAAGACAACCCGCAGGACCTAAGCCCGGTTTCTTGGTGGAG

AAGGCTGACGTTGAGACCGTCACTCCTATCCCCTACGACGTCGTCAACGATCTCAAGGGA

GGATACTAGAGAGTTTGGACAAGTTTGCGGAGACTTCAACGTTGCTTTGTCCTTACAATT

TCGACCCCATCTTTCTCGGCAAAAAAAAAAA

>92

TGGCCCCATTGGAAAGGAGTGCGCAGACTTGTGGCCCAGGATTGCAAGTGCAGCAAACGC

AATTGTATGAGCTTGAGACAAGAGGTCAGATTCATGTCTACAGAGACGTCTGACGCGCTT

TGTTAGTTGGTTTGTGCAGTTGGTACTTGTTGCAATGCGAAGAATTTTCATCCACTTGTT

AATAGAGGAAAAAGCCATTCAAAAAAAAAAA

>93

TAGTTTAGCAACTGCTTTATCGGCACTTCTCGTGCGTCGCCAGCATAAGTTGGCAGCTTG

TTTTGACTGTTGAAGCTATGCTGTCACCTTTCCTGGCGGCCGGCGATTATCCGACAGGCA

AAACTGAACCCTGGGTTTCCCGGTAGCTAGCAGATAACGTCCTGCATGAAGGACTCAAGG

TAATGACCAGATTGCCGGGTAAAAAAAAAAA

>94

TAAAAAAAAAAAAAAAAAAATCGTAGTCGCAGCATTCACAGCCCTATCCTGCATAGGCCT

CAGGTTGATATTCAATTCACAATCGGCGGTCAATCAGTCATACGGGAAAAAGGACGCTGT

ACAAGGCTGATCCTCATTGGGCGGCACACTAGTGCGAGTCGATTGCAGAGGGCCGGAACG

GGATCCACGTGCTCAATATCAAAAAAAAAAA

>95

CTGAAAGGACTCGATAGAATATTTAGAGAAGGTATTGTACTCATATTTACAAGAGCAGCT

TTGATTTTCAAGAGATATTCCGCAGGTGATTGAGATGGCAATTTTGAGAGATCACGACCA

AATCTGCTTGAATCAAGTTGAACCTTGTGCAGTATTCTCAGGGCGCTATATTCTATCCAT

GAACTGCTTTATATTTCTCGAAAAAAAAAAA

>96

AGGAGCGATTCAATGATTGAAACAATGACTGTACGGTTTGTGTATATAAATTGTTTGACA

CCGGACTCGATGAGATGGGTATAGCTAAGAAAACTTGAAAGACTGAGAGAGAGTGCGCAT

GGGCCGATCAGCTTCGTACTGTCAAAAAAAAAAAAAAAAAAATGGCCCGTTATGGAGGGC

CAACTTTATGTCAAACCTGGAAAAAAAAAAA

>97

CGTCAGTCGCATCACGTTAGTGGTAATACAAGTTGGACGGTCGCATTAGCCTCTGAACGC

CCACTTAAAGTTAAGTTGGATACTTTTTTGGTCAAGATCTTGAAAAAAAAAAAAGCAGCG

CTGATCCAAAGCGTATAGTGAAGGGCCTCAATGGAGGATGTCGTTGATATCTGGTTCCAA

TGTTGGATTGTTAAACCGTTAAAAAAAAAAA

>98

CGGGATTTCCTTGGGGTTTGTTTCCCGGCCTTTCGCTTCATACTGGGCTGGCGGATTCAC

CGAACCCCGGACCCCTGGGGGGTTATGCGTTGGAATTTAGATGGGGGGTGGGGTAGCTGG

TTGGAGAGGTCAAAAAGTCGTTTTTAAATGCTTTCGCCCCGCCTCAATTAAGAACGGTGG

GTTTTCCGCTGCTGTTTTTCAAAAAAAAAAA

>99

TGTTTCGTATGAGATCCGGCAGGGAACTCTTGATTGCAGGTTGTTGGTAATCTCGATTGG

TCTCACCACAAACTAGTGGTCAGGCAATCAGACAATGTCGCCGCTTGTAACGGTGTAGAG

TCTCTCAGTCTTAAAGTGGATGGTGTTTACAATCCTTTACTGAAAACTCAGCTTGTTGCT

GCAAATTTAAAATTCAGATCAAAAAAAAAAA

>100

GGGCTTTCCCTTTTGGTCGCCCGCCCGGCGGCCGCGATTCCCTTTGTGTTTGTCTACCAG

GCTTTCGCTTCATGGGGATAGGGTCAGGAAAAAAAAAAA

>101

GCCCAGTATTGACCAGACCAGTATAAACCAGACCAGTACTTACCACTATTGACTTGCAGA

CCAGACTCACAATGGCAGTTCAATAGCTACTGAACCATATAATATGGCAAAAAAAAAAAC

CACAGCAGGCACAGCAGTGTCATCATTGAACTGCAACGCCTCAACGTGGATTCATTTTCA

ATACGGGAAGCGTATCAAGCAAAAAAAAAAA

>102

AAGTCGTCAGTCCCAGTCAGGTCTGAGTGACTCGAGGGTCTTACAGTTTGAACTTGTGGA

AGTTTCCACGGGTGCCTAGCCCATACAACTTGAGACGTTGGTCACAGGGAAAGGAGAGCA

CTGAAAGGCTTTATATTGAGATCAGAGACTAGTAGCGGAGTTCCCTTCTGCGGTCAGAGT

TGGTCCCGCTGCTGACATCTAAAAAAAAAAA

>103

AAAGGACTCGATAGAATATTTAGAGAAGGTATTGTACTCATATTTACAAGAGCAGCTTTG

ATTTTCAAGAGATATTCCGCAGGTGATTGAGATGGCAATTTTGAGAGATCACGACCAAAT

TTGCTTGAATCAAGTTGAACCTTGTGCAGTATTTTCAGGGCGCAAAAAAAAAAAAAAAAA

AAAAATCGTGTCGCAGCATTAAAAAAAAAAA

>104

CTGGCAGACCCTGGCCACAACACCATCTTCGCGCAATTGTAATTTTCGACCCAATAAGCT

CGGATTGTCATGATTGGCATTGGTCGCCGCGTCTGCGAGCTGGGTTTGGTCACAAGCTAC

TGCAGTTTCTGATTGCTTACCTGTACATGTTCATACGGTCCCAATACAGAGGCATGTGTT

GCTATAAAAGACTCGTCACCAAAAAAAAAAA

>105

AAGTGGATACCATCGGTGGTTGAAAGTCGTGCAGGTCTGCTAGTCAGCTGCCGCACAGTT

TCAGGTAGATCTCATGCAGTTAACATGTATGGTTTAGTGAGGAACAAACAAAAACGTCCG

TCCATATCATTGTAAATATTGGGTTTGTACAAAAAAAAAAAAAAAATCGTAGCCGCAGCA

TTCACAGCCCTATCCCCAATAAAAAAAAAAA

>106

ATATCTCATGGTAGCATAGCGAAGAGCTCGCCGGAAGCGAGCACTAGTATCATGCTACTA

ATGCGTCCCGGATCCGGGTTGGGCTACCAGGCTCTTGAAGGTTTGTTGACAAAGGACCCA

ACCGTTAAGAGATGGGCCACTCTTTGCCATAAAAAAAAAAAAAAAAAAAAAAAAAAAAAT

CGTAGTCGCAGCATTCACAGAAAAAAAAAAA

>107

CCTGGTGGCCGATCCGCGGCGGTGCGAGCCCAAGAAGTTTGGTGGGCGGGGTGCCAGAGC

CCGTTTCCAGAAGTCTTACCGATAAGCGAACAGAACGAATATCTTGCGAGGATTGTCCAA

CGGATTTCCAGCCCGGATTTGCAACGGTGTGCGCATGGATTGTTAAAAAAAAAAAAAAAA

GGAATTGCTGATGGTTTGGTAAAAAAAAAAA

>108

CAGCTGCGGTTGAAGGCCACCAAGAACGCCGCCAAGGCGCTGGAGCCCATGGAGAAGGTG

CTGGCCCCTCTCTCGTACAAGGCCGCTGCATAAGGACGCGCGATTGGACAACCTTCTTAA

AATCGTTATGGGGTCTTAAAAAAAAAAAAAAAAAAAAAAAAAAAAATCGTAGTCGCAGCA

TTCACAGCCCTATCCCCAAGAAAAAAAAAAA

>109

CAAGAAGCCCCGGGCGGACAAGAAGAAGCGTGCTCTGACCACGTCGTAGATCCGGACTCA

TTTCGTCTGCTTTCAACAGGTTCTTGCCGATTCCACCGAGCTTGTTGTTCCAACTCTCTT

GCGGTAACACGAATGCATATGTGCGCATCGATACGGCCTGGCTGGTAATATTTTCTGAAT

TCGATCTGCACGTCATTGGTAAAAAAAAAAA

>110

CCTCATAAGATTTACAGAGCTATTCAAGAGACGAGTTGGCCAAGTTGATTCTCTGCGAGC

CAGAAAGCATCTTTACTAGCCGTAGATATGCAGGAGGCCTGTACCGCCAAGATGGATCAT

CGACTGAAATTCCTGTGTTGAGCTTAAGAGCGAGCCTTGCAGATCCCGGTCTGATCTGCA

AGCTTCGTGAACCTGGTTCCAAAAAAAAAAA

>111

AAGGTTTCGCAGGGGGTCGTGATTTGGTGGGGATAGTTGTTTGCAGGGGTTGTGAGTTAG

GATATGCCACTGGATTGATAGCGCTTTCCGTCTAAGGCAAAGCAGACTACGTGTCAGGCT

GTCGTGGGACTCCGATTCGGGATTGTTCTCTTTACATGACCTGCTGGTGCTCACCATCAT

TAACCAGATGCACATCGTCTAAAAAAAAAAA

>112

CAGGTTGTGACAGGCTAAGCTAGGTACTCTTTGTATAATAAGCACGGTGCTAGCATAACT

AGCCCTTGTCGATTGAACAACAACCTGCCGAAAGCCCCCTTGAAAGAAATGTGTGTGGGC

AAGGCAATTAAGCTGTGTAATTACCGTAGCGACAAACGTCTCTAGGGTTGGATTCATGAA

ACAAAAGGGATTTTATAAGCAAAAAAAAAAA

>113

AGTCTGCAAGGAATAGTATGTGGGCTGCTTTTCTGGCAAGCAACTCAGTCAAGTTGCTGC

TGAGAAAAGGGCCAATCTAGTGGCGGCGCCAGCTTAGATGATGCCACTTATTACAAAACG

TTTTAAGACTGGAGCTTGTGTACATGTATAGAGACTGGTGCTTCATACAATCAATTTGTG

GATACCTGCAAGTGAACATTAAAAAAAAAAA

>114

GCCAGGCCTGCAAGCACGTAACCCAACATTCACTGAAGAGGTGCAAGCACTTCGAAATCG

GTGGTGACAAGAAGGGTAAGGGTGCCCAGCTTTTCTAGGTGCATGTCTCAAGTACGTTCT

TGCTAACTAGACGTGGGCGAAGAATTCCTAAGGAAACTTGTACTTGTATATGCGCGTTTA

AGAATCTGAATATCTCCTTTAAAAAAAAAAA

>115

ATCCCAGAATGGAGAGGAAAGAGGTCACCTGAATTTGGTCCGGGTATGAGCACCCGTCGT

CATGCTGGTCTAGCTCTAGATAGCTGTGGCCGAGCCTCCAAACTTCGAGTCGATCCTTGA

GGCGATCATTTGATTCAGCTTCTTCCGACGATCGCAGCTTTGTACTTTCGGTGAGTGCTT

GATTGATTTTGTGTACCTACAAAAAAAAAAA

>116

TGGGTGCAATGGGGGTTGGGGGTTGGGGCCGTTGCCCCCGTTTTGCCCGCCTTCCGTTGT

CAATGTTTCCCCGGAGCCCGATTCGCTAGGTTGGCCCCAAGGGGTCAAGAAACCCCAACC

CCCGCCGTTCGTCAAAGGGGGTTTGTTGGGCCAGGTTTCCGTCCCTTTTTCAAAGTATTC

GGCCCCGTCCTGATTTTTGGAAAAAAAAAAA

>117

TCCCCCTGGGGGCGGGTTTTCGGAAAAAAAATTGGGGGAACCGGAAAGGGGACCCCCCCC

CCCAAAAATCCTAAGGGGGGGAAAAACCGGGCCGGGGCCCCCGGGCAAAAACCTGGGGCT

TTTTGGAAAAAACCTGGGCCCGCTGGGTTTGACCCCCCCCAACCCTGGGGGGGACAAACC

AAAACCCCCCCCCCCTGGCCAAAAAAAAAAA

>118

GCGGGCATTCGCTTCATGGATAGGTGAAGTTTAGGTCTGTATCACGTCATGAGAGCTCAT

CTAGAAATTGCATGTGCATGGGAAACAAGACCAGAGGGTTGTTCTTCAAGGATAATCAAA

TAGCCATTTGTCAGGCGGGCACCATTCAGGCCAAAAAAAAAAA

>119

GAGGCATATTCCATATGGAAAGTGAGATGTGAGTTTGTGTTTTTGTTAATGAGGCCAATA

CAGAGGAATCAGCCATCAAGTCTTTGATATAACAGCTAGGTAAGACTCGAATTTATGCGT

TAGGTATATCGTCTCGCCTACTGGGATTGAAGTTCCGGGTCCTCAGTCGGGCCAAGTTAT

TCTTGATGCAACGACAATATAAAAAAAAAAA

>120

TTATCGGATTCTTTGACCCAGGGAGCACCCTCTGGGGTTGGCAAAAAAAAAAA

>121

TTCCCGGCCTTTGGTTTTTTCTTTATAAATACAAAATATAAATAAAAAAAGACACATCAA

AATAATAAACAAAAAAAAAAA

>122

TCTTTGGTGGAAGCACGTTCTTTGTACATGCTGATGTGTTGGTGTGGTAAGAGAGTGAAA

TTCAGTTGCATTGCCAAAGGACTTGGCCAGCTGTGGAAGGTGATTTGTAGAGCTAGAAGA

ACAGCGTTGAGCAGATTCGATCAGTGGCCAATATTGTACTCGGAGCTGGTAAAACGATCT

GGCAGGATCGATTAACGTCTAAAAAAAAAAA

>123

TGCTTTATCCTGGAGCAAAGCTCCGACTGCCTCCCTGGGTGGCTGGGGCAGCCATGGCCT

TCTCTTCTGTCTCGGTCGTTTGCTCCAGCCTGCTCCTCAGAAGGTACCGTAGGCCCAGAC

TGATTCAACTCAGGGAGCTGGCAATTGAATAGTACGACAGCAGATCTCGCGATTTCGTAG

ATGGGTTAGCAAGAACCTTTAAAAAAAAAAA

>124

GGACCTGGCCCCCCCGGGGTGGTTTTACCCCCTTGGGGGGGGGGGGCCCTTTTGTGGCCC

GGCCCGGTTTCTTTTTAGGAAATGGGTTGGGGCCGCCCCGGAAGGTTTTTGGGGGGTTTG

GACCTTAAAGGCCCGGGGGGGGCCCCTTTTTAAAATTTGTAAGGCCCGGTGGGTCCGGGG

CCCGCCCTTTTCTTTTTTTCAAAAAAAAAAA

>125

GCATCGCGGAGGTGACCGGCAGAGAGCGAAGCCTGGATCAGGTTGACCACGTCTGCGTTG

CAAGTGGGATGACGCGTCTCTGTACGGTGCCTGCCCTACCACAGCAGGGGTGCGATGATC

CAACAGGATGTCAACACGTCGTTATGAAGCTGGTTTATGTGTTGGCTCCATAGAAAAAAA

AAAA

>126

GCAAATCAGTGGACTTGATGTATGAGGGTATATTTGCATAGTGGATTTGGAAGCCTCCCC

TTCAACAACTTAGTTGAGAAGTTGCACCAGGACAACTCGGTTCGTGTACTTTTGGGGTGT

TGCTCGCTACAAAGGCCTGGGATGCAATAAGGTTAAATACGGGTATGGGCCAAATTTTGA

ATTCCCCAAGACTTTTTGTCAAAAAAAAAAA

>127

CCGGAGTTCCATCCCGTGGGCACATTCTCGTTCGAGTGTGCGTGGCTCGGCCCGCTGTAT

TGCCCCTTATATTTGACTTGCCCCGTAGTGGCCAGCCCCGTATAAACCAGACCCGTACTT

TCCCCTATTGACTTGCAGGCCGGGCTCCCAATGGCAGTTCCATAGGTACTGAACCATATA

TTTTGGCCACTGGAAAAAAAAAAA

>128

CCACCGACCGGTCATGTGGTAGTAAGTGGTACCCCGAAAAATGGCCCGGATTCCGGTAGA

ATTCGGGGATCTCAAATTTTGTTCCTCGGTAGTAAGTATGTACCACGGTACAGACGGTAC

TATGGCCACGAACCAGCGAATAACAAACCCGAAAATTCACCCATAATGAAGGCCCAATGC

CGGGGATTGTGCACTCGGCGAAAAAAAAAAA

>129

GGTGTACTTCAACTACTTGAACAACTTCAACCCGGAGTTGTCTGTAGGTAGCACTGTCTT

GATCGTCCTTTTGCTCAGCCTCGCAGCAGCGGCTGTAGAGGCTCTGCCCTTTCAAGACGT

TGACAACATAACGGTGCCGCTTGCTGTAATTATTCTAGGCAACTGGCTCTTGCCGTTTGG

AACCGTCAATTGTGAATACTAAAAAAAAAAA

>130

GCAGTTCCTCCGTAAGCAGCCGTGCCCTAAAAAGTGAGCCATGTTTTGGTCCTCCCCGGC

AAAAAAAAAAA

>131

CAGTTCGGCCTTCCGAATGTCAACTTGACACAGACACGAAGACGCAGACGCCAATATAGG

CAGCTTTGCGGTACTGGTTCGGAAGTCGTAAGTACTGCTCAAGCTAGCAGGCTGGATGTA

AAGTACGTAGATTGCTCACTGACTGTCCCAACATCGATCTGTCGATGGAATAGTATATTC

TTATTAAGTGGTTTAATTATAAAAAAAAAAA

>132

CATTCGCTTCATGGACTTGCCCTTTTGGTGCTATTTCTGTGTGAGAATCTGTTTGGATGC

ACGCCATGCTGTTCGAGACTGGTTGTGCGTCTGTCCTTCACAAAAGGATGGAGATCATCT

GAAACAAGAGAAAACGCCACTAACCCCGTCTTTCCTCTTAAAAAAAAAAA

>133

CTATTTCCACAAATCAGACTTTGTGGAAGCTGCAGTGGATCTCACAGTTTGTGCATACTT

GTTTACTTTGTTGCTAAAGGGACACACCGTGGGAGGACAGCATTGAGTAAAGTGGGACTG

ATGTGTTGTGGTGAAGGGAACTGGTCCGGTAGTCTGAGGAGGAAGAAGTTTCTAATGTGA

CATCCTTTAGTTTCGGAGTCAAAAAAAAAAA

>134

GTCTACCAGGCATTCGCTTCATGGGGGATAGGGTGGCTTTGGTTATTGTGTTGTTATGTG

GCATTTATATTCGATATTGTGGATCGCGTTGTGGCATCGCGTTGCATGAACATGTTCATC

AGACATGTTCGCATTTTAATGAAAGCCGTGAGCGAGGATTCTCAATTTGACCTCAAAAAA

AAAAA

>135

TTCGATACGGTTGTTATGGCTGAGATGTTCCAGAGTTGAAGCAGCACTGAATCAATGACC

CTACTGGTGTTTCCATTCAAGAAATTGAGCCTGGGTGCCTGCTAGTCCTTGACCCTGGCA

TTTGATGAGGCCCAGCTTTTTGTTCTGTTCTTTTGTTCATATCTTCAGCAATGTCAGCAT

GAAAACATGGATCGAGACATAAAAAAAAAAA

>136

GAAGGTCTCCGGAGTTTCATTGCTGGCTTTGTTTAAGGAGAAGAAGGAAAAGCCAAGATC

ATGAGGTTCTGTACAACTAGTATGGTATTTTCTCGTTTAGTTTTTGACTGAGATGCGAGG

TGGTGGTAAAGAAATCTGACTTGATGCATACCTTGGGTAATACTTACACCTTTGGTAATC

AAGCTGTGATTTGTTTCTTGAAAAAAAAAAA

>137

TTGGTGTTACTAGGTTTGTATGGCGTACCTCTATTTTGTACCTTGTTGGGGAAGTTTACA

ACATGTTTTTCCCGAGCAAGTGACTGGTGATAAAACATTGGAGTTGTACAGTGTGTGCAT

TTGCATGTAGTGCGGAGAGGCTTCACAAAAGATGTCAGCAGTTTTGTTGTGAGAGAGAAA

ATGAGTCGGCAATTCGTATGAAAAAAAAAAA

>138

AGGACTTCCCTTCCCAAATAGATGCCGGGGAGCTTGGGGTCCCTGAAGAGTAGTTTTGGA

AGATTTTGCCCTTTTTTATTCCCCCTTTTGCCCGTTTCCATAACCGTTTCCGGCTGCGTT

TTTTTCCAACCCAGGCCCCTGAGGCCCTGTTTGCCCCTTTAATTTAAGCCCCTTTTGGCC

CCCTCCAGTTTTTTTCCCGTAAAAAAAAAAA

>139

TTGCAGAGGAGTTTCACAAGTTGACCCTACTAATGCGAGAGTACTCGGGTTTTATGGTGG

ACAGATACAGCGAAAGAAAGGAAAAGCTTGAAAAAGGCCGTTTGTTATCGCAAGCAGAGA

AGGAAGCAAGTACAAACTCCGTGAAGGAAGTTGTCTAATCGTTCGCATCTGTCTCAAAAT

GCAGTGATAGCCTGAAGTATAAAAAAAAAAA

>140

ATAAGTTACCGAAAAGGTTTGCAGATTGAAAAGCGTGTTACTAATGGTGTCATTGACCAG

ATAATACAACGATCGAGCATTGCTCTTAGGTTTGCTCTTAGGTTACGTCCTTGACTGAAC

TTAGATATGTATTGGATATGGTGCATTAAGGTGCACGATTCGTGATCGATTAGACCTAGG

CTCCCGTGTGGTGACAGCTTAAAAAAAAAAA

>141

GGACAGGACGTTTCACGAATCAAAGCACAGTCAGCAAGCAAATATGTTATGTGCACGAGT

TCCATAGTGGGGCCGGGCTAGCACGACTTACACATGAGGCGTCAGCTTTGGAAACCCTTT

CGTAATCGAAGTGAGAAGCTAGGCACTGATTTGGTATCAGGACTACGGTTCGGTTGTCAA

TTTTGCGGCACATCGATGATAAAAAAAAAAA

>142

GACGTAGATTCAGCTTCAGGCAGGGGCTGGCTGTTAGGGTGACTTGGCAGAGAGTGTAAA

TATAGAACAGGAGAGGTATTGTCACTGGGCGGATGCTTTTGGGCATTGTTTTCCCCTCCC

CCATCTCGCTGTCTGACAACTAGTTATCTCGGTTACTTCCAAGTTTTGACGTGGGTCATG

ATCAAGAAAACCTCTTGTCGAAAAAAAAAAA

>143

AAACCCCCCAATTTCCGGGCCGGATTAGTGGATGTTCCAGGGGAGTTTCCCAAGTTGCCC

CTATTAATGGGGGGGTACTCGGGTTTTAGGGGGGCCAGATCCAGGGAAAGAAAGGAAAAG

CTTGAAAAAGCCCGTTTTTTTTCCCAACCGGAGAGGGAAGCAAGTCCAAACTCCGGGAGG

GAAGTTGTTTATTCTTTCCCAAAAAAAAAAA

>144

AGGTGGGGACACTTAGATTGGGGGTTTCTGTGTGTATATAGGTGCTTAATGTCATGCATG

TTTTAGGGATGTGGAGTGCTTGTGCTGGACCCTAGAGATGTTAGAATATGGTTCGTGTCG

TGCTTGCTTTTTCCCACTGCAGTTTGGATCCAGAGACAGATACCAAATTGCAGTTCAGTT

CCCAACATGCATAAGGGTGTAAAAAAAAAAA

>145

GTGTTGAAATGATAGCTTGCTAGTAGTCGGATCACAAAATTGCTGGCGCTTAGCTCAAGC

GAGAAAGATGCATAAATCACAGGACCAGATGAGCAGCTCGGCCCGGGTTATTAACCGAGA

AACTATCAAGGGAGTGCAAGTGCGCAGAACCGAGAGAGAAGCTGTACATACGTAGGAGTC

AGTCAATTAAGTCGGTTGGCAAAAAAAAAAA

>146

GGTCAGAACAGTTGAGGTCCTTCATTGTCATGCAGCGGCGCGTCATGTGGTCAAGATCAG

GAGATGTGACCTAGTGACTTGATACTCATGGAGGCTTTCAGATAGCTTATCTTGAGGTGT

AGATTTTGTGCCTTGCCACGTTGTACAGTTATGTCGCGTTTGATGCACTTGAGTTAGTTC

ACGCTTATTGCGTTTCCATGAAAAAAAAAAA

>147

CAGGCATTCGCTTCATTCTTTCTCGGGCCCGTTTAGGCTTGGGGGTATAATAGTAGGGAG

TCGTGGGCAGGACAGTCGGCGACTCAGATGTGGTTGGAATAAGTACACACGCTCTGGCAT

GTAAAGCGCAGACGCGAACATTGCTTTCATATATAGACAGACACTTACAAAAAAAAAAA

>148

TATTTAGAGAAGGTTTTGTTTTCATATTTTCAAGAGCAGCTTTGATTTTCAAGAGATTTT

TCCCCGGGGATTGAGATGGCAATTTTGAGAGATCCCGGCCAAATTTGCTTGAATCAAGTT

GAACCTTGTGCAGTATTTTCAGGGCGGTATATTTTATCCCTGAAATGCTTTATATTTTTT

CCTCGATCGTTTTGGGCGGGAAAAAAAAAAA

>149

GTCACCAGGCATTCGCTTCCATCAAGGAGAGGGGACTACCAGGCATTCGCTTCATGGGGG

ATTGGGAGGCAGAACCAAAAAAAAAAA

>150

AAGAAGAGAAAGGAAGAGAAGCGAGCTGAGGTAGAGAAGAAGTTTCAAGAGATAGAGGCC

AGCCGAGCATCATAATGATGTTTCAGGTGTGGTAGGTCCCTCTAGGCTGGTTATGAAAGT

AGTCTGGCCGCTGTACACAAGGTGACGTGCTCAGTCATGCTGTACAATTTCGTACCGTCT

GCGCGGCCACTGTAACATCGAAAAAAAAAAA

>151

GGGGGAATTTTGGTTCAGAGGAGTGGGGAATAAGTGGGTTGGGCACCACGTAAGTCAAGT

TCCGTTTCCGGGTCGGGCCATTATTAGCCCGTCGTTCCGTGGAAAGTTTCCGGCCCCGTC

AAAATTGTTAAAAGATGGGTTGGGAACCCTTTTCAACCAATTGGACCCTTCCAGGGTTTT

AAGGGGGTGCTTAACCTTCCAAAAAAAAAAA

>152

TATTGAGGCCCCTAGAAGAGGAGCGATTGCAGGGCACCTAGATGTGAAGCGATTGGTCAG

TAGGTTCTATGCTTCATCGAGTTACAAGTCAACTTTGAGTCTGTATACTAAAAACATTGA

GATCTCCTGACGAGTCCAGCAATCAGCACTTGACGGCCATGCCCAGCAACAAAGTGTTTG

TCAAAAGTACTGTCTTGCACAAAAAAAAAAA

>153

ACGTAGCTGGCCCATGCATTGTCTTGGAATAAGAGTTGACCCCCGGGGTCGGGAACTAAC

CTTCTTAAGGCACCTTGGTCGAGAAACTGTACCCGAAGACGATCGGTACCAGATTACAGT

AAAAGTTGGTTGCTGTCGAGTAATTGGTCGTTGCCTTTAAGACCAGACCCTATAACGGTT

CTGATTAAACCTCAGACATGAAAAAAAAAAA

>154

TTGAATGTCGAAGCTCACTAGCTGGCGTATATTGAATGATGTGTATACTTTGATTCGATG

CATACTGCTAAAGTGAGAACTTTGTTCTGGAAGCTTTTTAAGTGTAGTAGGAACTTACGT

TGACATTGAACGCTTATATTTGATTGACTTTGTTTGGTGCACTGAAGTGCAGGGCGGCGG

CCCCGCATACAATCCTATCCAAAAAAAAAAA

>155

ATGAGTCCAACAGGAAAGCTACGTGCAGTACAACAGACAGTGGGAGGGAGGTTTTGTCCG

GTTGAGTTCTGCCGGTAGTTTGAGGATTATTCACGGATACCGGAGGCTTTTCGAAGCTAT

AACCCGGCGGTTCGCGGATATCCGGTACCAGTCCGTACTCGTATGGACCTAATCCGGTAT

TATTATTCATTTTTCGTAAGAAAAAAAAAAA

>156

CCCCCTTGGGATTTTTCACGTCTCCCTTCCCCCGCTGGGGGGCTTTTCCCCTTGGGGGCC

CGCCCCGCGGTCCCCGCCCCCCTTGGGGGTTTTTTCCCCGCCTTTCCTTCCTGGGGGGTG

GGGGGACCCTTTTTTCCAAAAAAAAAAA

>157

CCCCGGAATTTTTTTTTTCCGGGGGTGCCAGGTTTGGGGTTAGGGCCCGGCTTTTTACCT

TGCTTTTAGGGCCGTTCGGGGGGTTGGGGGGTTTTTTCCTTTGGGAGGGGGCCCCGGGGG

TTGGCCGGCCCCCTTTTGGGTTTTTTGGGGGACTGTTTTTTCCCCCTTAGGGGGGTTGGG

CCTTTTGGGGGGGGGTGGGGAAAAAAAAAAA

>158

TTTCGGGGGAAACCGGGGGGGGGGGTTTCGGCCCCGCCGGGGGGGGTTTGGGGGGGGGGG

TTAAGTTTCCCCGGGCCCGGGGTTTTTTTGGGGGGGGGTTTGTCCCATTTTTTTTTGGCG

GGGGGGGGGGGGGGGGGGGGGGGGGGGGGGAACCCCCAACCGTTTTTTTTTTTTTTTTGG

GTGGGGGGGGGGGCCCCCTGAAAAAAAAAAA

>159

CTGGTTGTTGGCAAAGTGATCAAGAATGTGGGGGGTTTTTTGCCATTGTTCCGTTTGACA

TTTTTGATTTTTTGCGTTGTTCTGTCAAAATTCGGGTTGTTCTTTGTCCAACTTTCTTGA

AGGTTTGCTAACGGGGTTGGGGTTGCGCCCCCCCTCAAATGCCCATGTTGGCCTGGGGGG

CCGTTTAATGTCCAGCTTGGAAAAAAAAAAA

>160

TGACTTTAAATAGCTCGAGCAGACCCTTTTAGAGGTGGAACAAAGGAACAATGAGGACGG

ACAGTTTATCTTTTACCGTATAATCAGAAAGCTAGGTCTAGAAGACATTACTAACAAACA

AAATTGAGACTGAGTTGCTTGGGAACCGCTGAGGACTAGACTAAATAGATCGCGGCTTAC

TTATACAAGATTGATAGTTGAAAAAAAAAAA

>161

AAATTTTGGGGAAGAGTTTTCCGGCCCGAGTTTGTGTTCGAACAGCTTCGGTTCCCCTTG

ACCTTGTCCCGTTCATACCTAGCTCCCCCTGAAGCATAGCGAGCCGGGTCAAGCCGTCCC

CGGAACGGTGTTTGGGGAACCCCCGATTTTTGGGTTTTTTTTTTGGGGGGGATTTAATAA

CTTGAACGGGTTTCCCATGGAAAAAAAAAAA

>162

CGGACCTCCCAGAAGACGGGGCGCTCGAGTCCAACTTTTTTTTTGACATCTTGTTAAGTT

AAGCGAGGGGGAACAAGGGGGTGGTTAGCTGCCAATCCTGACGGTCGCCCGCGAAAGAAA

TAGGTTTGACGTGCGAGGCAGCTGTTTCGGACGTTGCTCCTGCGCCTGCTATTTAATCCC

GGCCTAAAGGGGAAAGTGGGAAAAAAAAAAA

>163

TCCCAAGTAGCGCCTCCAACATGTGCCCACCATATAAGTACACATAGCCTGCCTAGTTTC

TGGTAAACAACAGAAGACAGTGGGAAAGTTTTGATGACATTGCAGGACCATCGTAGTAGC

CTGTGAGCAGCGTGTTGGCTTCACTAAGGTTGGCAATTGGGTCTTCCATAGTTTAGCCAA

CACGAACCGCTCTTGAAAGGAAAAAAAAAAA

>164

GGCAGGAAGCTGGCGGTGCAGGACAAGAGGGCGAAGTTGGGCGACTTTGACAGGTTCAAG

GTCATGGTGGCCAGGGTCAAGAGGAGTGCTGTCCTGAAGCGAGAGCTAGCCAAGCTGAGG

AAAACAAAGTAATATGGACATGGGCACCTGTAACATTGCCAAGTACGGCTCTGACTTTTA

TCTCGTGGTTCCTCCCATGCAAAAAAAAAAA

>165

CCTGCCTAGTTTTTGGTAAACACCAGAAGCCAGTGGGAAAGTTTTGATCATTGCAGGCCC

ATCGTAGTAGCCTGTGAGCAGCGTGTTGGCTTCACTAAGGTTGGCAATTGGGTCTTCCAT

AGTTTAGCCACCACGACCCGTTCTTGAAAGGATAAGTGTATCATGTGATCTTAAGTTGGT

GCCGTTCTGGTGCGCCGCCCAAAAAAAAAAA

>166

GTAAACTTGCAGGAGAGTGGGCGGGGTTAGTTGACGTAATTTAGTCAGAGTGTACTTATA

CCGTGCCTTCAGTAGATGTGTAGTCAGTATGTTGTATAGGACAGTCTTCTTATGTGAGAA

CTAATGTTGTTGAGTATCTAGGACTAAGTAGCTGAGAAGGAACGAAAGAGGGGCCTTCGT

ATGCTTGTGTTATAGCCCGTAAAAAAAAAAA

>167

CTGATTGATTCGAGTTGCCTTTTGAGTATCCCAGCTTCACATACAACACTCCCTACAATC

GAGTGTCGACACTGAGACAAATATGTACATAGAAGTAGTCGAGAACGATGCACCAAGCGT

TTGAGGCATAATGTGCAGGTGCAGACAATCATCAAGGACGATCATAAATCATAACTTCAA

ATTTAAAGACGTTTACAGGTAAAAAAAAAAA

>168

GTTATTGCATCGGAAGGAGATTAGCAGCACAAGTATTAGCACAACTGCGGCCGCCTTCAG

CGATTTTTTCACACTCTTGTTTTGGAACGGTTACGATCCGACTCGGCTCGGCCGACAGCT

TGGCAGAGATGGTCGAATGGGGTAGTGGGTTCTTCCGACACAACGCACTGCATGGCAGTT

GGAAAGCACGTGTCTGTCACAAAAAAAAAAA

>169

GGGTTTTACCTAAGTTAAATTTTTTTTTTTTTTTTCCCGGGGGGTTTTGGGGGGTTTTTC

CCTTTTTTTGGCCAAAAGGTTTTTTTTTTTTTTCGGGGGCGGGGCTTTTTGGAAGGGGGT

TGTTTTTTTTTGGGTCCCCCTTTGGAATTGGGGGGGTTTTTTTTTTGCCCGGGCGGGTTT

TTTTTTCCCCTGGGCTTGGGAAAAAAAAAAA

>170

TGTTGTCCACATAGCTTGTGCCAAAAGCGTGCGCAGAAGCTGGTGGTGATCAAGGAAGTG

GAACCCCGAGACGGATCACACGGCCAAAATTTACCATTGGTCGGAAAGTCTTTCAGCACG

TTTAGGCAAGCAAGCTCGAGGGCATCTGTATCTTGCTTCTGCACCCTCTGTATATACGAA

GGATTTTGCCTTGACCCCCCAAAAAAAAAAA

>171

CCGCCCAATTTTTCCCATGCCCAGTGGGCCAGAAACCTGGCAAAGGGAAGTATTTTTTGG

GGGCCCCAATGGAAGTTATGCAGCTCTTAAACGGTTTAGCCAATTTTGGCCAACAACCCG

GCCCACTGGAGTTTTGTAACGGAAAAAGTTTGCTCCCTTTCATAAGGTAGGACTGGCCTT

TCCAAAAAAAAAAA

>172

GAGAACCCGAAAGTAGTCAACATCTTCGATTCAGGATAGGTAAGGGCGTCATCAGTGTAC

AGAAAGAAAAGGTGCAGGCGATATTTTCAGATGAGTTGGCCTAGACTCGTATGTAGGAGC

TTCTCTGTAGTGAAAAGGTCACTGCCATAAGGCGCTTCCTAGATGAGGGACTGCTATAGA

TCCCCAAGAATGTTGCGAAGAAAAAAAAAAA

>173

CGTCTGTCCCTGTTGAGTTAGGGAGACTCGTTTGGTAGAGGCCAAAGTTTTTTTTGCCAA

GCATAAATAGAAGTTTCCTGTGTTTTGCTTGATCAATTGTTTTGGTGGGCTGTTGGCAGT

CTTGGCTTTCTTACAAAGAGTAGATCAAAGACACTTGTATAGATAGGCTGATATATAATT

CCATTCCGGAGCTTCAATTTAAAAAAAAAAA

>174

GCTCCCGTAGGTGGGGCAGGGTTGGGAATGTTAGGTGCGTCTAGTTGCTCAGCGGGAGGG

TTATGGACGGGCTGGGTAGCCAAGCCGGCGGAGCAGTGTACAGATACCGAATAGACAATG

TGTACATCTTATATACTTGAAACATTGCAGATTTGAGTCTTTTGTGTCACTTTGACGTAT

GTAACGTAAGCTTATAGCTCAAAAAAAAAAA

>175

GTCTACCAGGCATTCGCTTCATGAGTCATAGGGACATGCGGCAAAGGTAGGCTTCAGTAA

CGTTTGTCCTTATACTCCCTTAGTGAGTTGTTCTGCCTGGTCGGAAACCAAAAAAAAAAA

>176

TTTGGAACCGCGTTCTAGGAATCATTATGTTCAGCTTTGAGGTTTGGAGATACGAAGCCC

CATAAAAGTGGCATAAGCTGTGTACATACGACAAAGGCCTATGTGAGAGTAGATCTTTTG

TATGGCTTTCTCGTTGATAAACTATACTGTACTGTTCTCAGTGGATACACTTGATGTGAT

GACAGGTCCAAGTTTTCTCGAAAAAAAAAAA

>177

CAGGCAGGCAGTTGATCAGATCCGACACAGGTCCGGACCGCGAGTTGTTGTTTACAGTTC

CCGTGTCGAAGTGCACCTACGTTGAGAGAAGGGGGACCCAGTCCGTCCCCCGCAGCTAGG

GCAAGTCTGTACAGTCGATTTTCCGAGGCATTGAAACACTCCCAACGACAATAATGGAAG

TTTTAAGCACGTCTCCTGGTAAAAAAAAAAA

>178

GTTTGTGGTAGTTGAAGGAGGTCACCACTCAGGCGTCGTCCTATTGCATAGGGATAGGTT

GTTTGTTGTAGTCTTTAGTTCAGACGACCCAAGAAAAGGGGTAGTCAAATTGCATAGGGA

TAGGTTGCAAAATATATAACCGAATGTTGTAGAGACTGCCATGAGCCTTTACTAAGAAAT

CAGAAATCCTTTTCGATTCGAAAAAAAAAAA

>179

CGGGCTTCCGGGGGGTTCATGAGCGCGGGGTTTTTCAGGGAGAGGTTCTTTCGTCCCTTC

CTTGTGGGGGGCCAGAAGTTTGTGAAGAAGGTGTTCAAGTTCCGGAATTCAAAGGAAAGG

GGGGCTCCCAAGGCCTCCCCATAATTTTTTTCCGGATTTGGGGGATTTTTTTCCCCTTAT

TTGTTTCCCGGTCCTCCGGCAAAAAAAAAAA

>180

GAGCATGGGAAGACACCAAGGACAGGGCTCGTGATGCCACGAGATAGAAAAATTAGGATT

TAATTAGCCTGTGAATAATCAATTTTGAGTCAATTTTGCAAGACAGTCGAGTCAGTGTCT

TGAACACATACAGAAACTGTTCGATCATTTTTGAACATGTGGTTATGAAAGTGACCTGAA

CAAGCACGTGATTGTACATCAAAAAAAAAAA

>181

AAACACATGTATTTGGCAATTGTATTTGTTTCGAACCTGTCATCAGGTAGCTTAACAAGT

AAAAATATCAGAAATCGTCATTTGACAAGGCAGTAGGACGAATAGATATGTAAAGGTTTT

TGAAAATATCCTGTTCAATAGTAATGCATTGAATAACCGACTTTTTTGCCATTTCAAGTA

GTCATACAATGGTCCAATGGAAAAAAAAAAA

>182

CCCCCCCCTTTTTTCCCCCTTTTTTTTTCCCGGGGGCGGGGGGGGTCTCCGTTTCCCCCG

CGGGTTTGGGGGCCCCCCCCCCCCTGGGGGGTTTTTTTTCCCCCCCCGGGGGGGTTTTTT

TTTGGGGGCCCCCCCCCACGGGGGCTCCCCCCAACCCCCCCTTTTTTTTTCTTTTTTTTT

TTGGTTTTGGGGGGCCCAAAAAAAAAAA

>183

GGAGCTGCGTACCCTGTATAAAGGAGTGAGAAGTGAGCGGCTTGTGTTGGCAAGGAGGGG

TTATAGCGCGTGGCATAAAGAGCTAGGCAGCTAGTACGAAACCAGGTCGTTATGTACAGC

ATCTTTCCCTCATGGGAGAGCTACGGCTTTACTCAGTGCTGACAGCTGGTGGCCCAATTT

TTATAGTTGACCGTCCCTTGAAAAAAAAAAA

>184

TGTCCACGGATCAGAAGAATGCTCTATATAAGGTCAGCCTGTACAGTCCTAAGTAATCTT

TTCTAGCCGCATAAACTGTAGACTACGGATGTGAGGCACGTTAAGTAACCATAACCGTCC

GCGCAGCAGTGAGGTTAGTAGCTCGTCAGCTACATCGAGGAATGAACTAACTGCAGACTG

CAACTGTTTAGTCTAGCTTGAAAAAAAAAAA

>185

AGGGTCGAGCGGAACCGTCAAGGTCACGAAGAAGAAAGAGTAGCGCTTCTGGGAAGCAGG

CACTCAAGAAAGCACTCGTGCGGGGCAATTGTGTGTGTCTATTCTGTCCTAGGAACTTTC

GAGGTTAGATGGTCCCTCAGCGGCTTGTAGGGGAGAATCGCGACTGTGCTTTTGATTGAA

TCCGAGAATCGTTTCACAAGAAAAAAAAAAA

>186

TTTGGGGGTTTCCGGGCCGCCCAGGGGGAAATCAAGGGTGCTGTGGAGAAAATTTTCGCC

CTCCCACCCAGGAAGGTCACCCCCCTGATCCGGCCCGATGGGGGGAAGAAGGGGTTCGTC

CGGGTTTCCCCCGGTTTCGGTGCTTTTGGCCTTGCAAACCAGGTTGGGGTCCTTTAATTT

GCCGGGCCTGGGTTTGTTGGAAAAAAAAAAA

>187

GGGGCCCCCCTTTTTTTTTTTTTTTTTGGGGGGGGGGGGGGCCCCCCCCCCTTTTTTTTT

TTTCAGGGGGGCCCGGGGGGGGGTCAATTTTTTCCCCCCCCCCCCCCCCCTTTGGTTTCC

CCCCCCCCCCCTTCCTTTTTTTTTTCCCTTTTTTGGGGGGAATAAGGGGCCAATTTGGGG

GGTTTTTTCCCCTTCAATTCAAAAAAAAAAA

>188

GTCTACCAGGCATTCGCTTCATTGGGGATAGGGCACTAAGGCCTAGGCGCAGCACACATG

CTCTGTTGTGGACACCCGTGCTGTACAGTATTTGACGCCCAAAAAAAAAAA

>189

GGGCATCCCCAAGAGGGTGATTGACTTGCCCAGCCCATCGGAGGTTGTCAAGCAGATTAC

CTTTTTCACCATTGAGCCCGGTGTCGAGGTGGAGGTTACCCTTGCTGATGTTGCATAGAG

GAACCGGAACCTATTGATATCATCCTTTTGGGGACATGGGGCATAGTTCCCCCTGTGGGA

ATGGTCGACCTAGCTTTTTTAAAAAAAAAAA

>190

GTACTGTAACTCGATTGTACATACCTCGAAGAAACTGCAAGCAATTGCAAAGCAGAAATG

GGACGTCTCTCTCCTTGTACAGCTATAGTAGCTTTGTGTAATGGAGCAGATAACCAAGTG

GAATGCAAGACCATTCGGCTACGGCATATACCTGGTGAACCAGTGAAATTGCATCAGCGT

GCAAGAATTGAGAGGCTACTAAAAAAAAAAA

>191

GACAAAATGTACAACTGAAAGCAAACAAGTGCCAAAGGAGCCGATCATATAGACGTGTAA

TGCGGATGGCAATGGGTAGCTTCAGCTAGCCGATAATTGATAGTCCTAGCGAAAGCTGTT

GTTCGTGTAGTCCCAGGCCTGTCATGACGTCCTTGTACATAGCTCTCTGGGACACCTTAA

TGCAGTAATAGTAGTTTACGAAAAAAAAAAA

>192

TAATTAACCGTCTATACATGATCAGTGTTCCTTTCGATAATTCTCGGTGCCAATCAGGAT

CTGGGGTCAAGGCCTGAATACTTTCACTGCAGGGCAAGCAAGGTGATCGATCGGACTTGG

ACACCAGATTTGCGAGAAAGTCTAGCTTTAGCGATCACATGATGCTGGAACTTAAGCTTC

GATCTGATATCCGTTCAATTAAAAAAAAAAA

>193

GGACCGGGTTTCCTTGCCTTTAAATAGCTGGGGCAGCCCCTTTTGGGGGGGGACCAAGGG

ACCAATGGGGCCGGCCAGTTTTTTTTTTCCCGTTTATTCAGAAAGTTGGTTTTGGAGGCC

TTTTTTACCAACCAAAATTGGGATTGATTTGTTTGGGACCCGCTGGGGATTGGATTAAAT

AGATCGGGGTTTTTTTTTCCAAAAAAAAAAA

>194

AACAATCGACCAAGGCAAGTGATTCAGCCCAATATGGGAATACCAACTAGAATTGAATCG

GGTCTCTCCTAGAAGTTAGAAAAGAGGAGATACGGACTCAGCATTGGGCGGTCCGAGTCT

CTTCCCGTATATGGGCCACATTGTGACGGTGCTAATTACGAACCAAGTATAAGAAGTCGG

GCACCGACCTGGCATGAGTGAAAAAAAAAAA

>195

TGTTGACAAGGAGCTCTAGAAGTGTTTGGTTACTCGACTGCGAGGACATGTGCGTGGTGT

TCGGCTGATTGGTGCTACTTTCCATTCTAGATGGATAAAAATGCACAATAAGAACTCGTG

AAGAGCTTGATTGGATTTCGGGCCGAGGATCTAGCATATTAACATTGACAACATCATTAA

ATATATACGTGGTGCCGTTCAAAAAAAAAAA

>196

GGAGTAAACGGATTTTGCATTGTCAGGCAGTTGCATTCCAGATGGTGTTCTCACTCCTCA

ACGTCGCGCTTTTGCCAGGTCGAGCAAGATCGAATGAAATCATGTCTCTGCTCGACAATT

AATCAATGCTCTTGATATGTGATTTGAGGAACAATCCGCAGGTTCCAGAAGCATCCTTTG

GAGACCAGAATCGAAAGATCAAAAAAAAAAA

>197

AATATGTAGGTTACTGGTGCTGCTGGCTAATCGATTGTTGGTCTGTGGTTTCAGTAACTT

ACAGTGTTTGAGATAGTACAGACCTTGAGGAGGGGGTTTGTGGAATAGTTCAACAGTTTG

TGGCAGTTGAGGCTACTGGACAGTCGCCTGCACTTGCGCGACAGATGTCATGTATTGCAT

TGAATTGCGGTCTGACCCCTAAAAAAAAAAA

>198

AAGCCCTGAGTCGATCTTCCTTCTCGGTCATCAGCAAAGCCGAGATCAGAGTCCAATGGT

CCCTTTTTCAAAAAAAAAAA

>199

CAAAACCGATCGGTTGGCTAACGTCTATGAAAAACATGGCAGTATACAGCTTTCGTATCA

GTTGGACAGACGAAGAATGATAGTGCAGGGAGAGACGTACTATTAGCCCATGATCTCTGA

CTGTGTCCTGTATAACGAAGGTTCTGCTCGCAGATAAAGACCCGTACGCGACTGCAAATC

ACGTGATTGTTCAAAACCTTAAAAAAAAAAA

>200

AGAACAATGGCCGGCGCGGGGGGGCCGTCGGTGTGTCCAAGAAGCGGTAGAGACGGTAAC

CCTTGGTTTTTCCCGGCAGCGTTGGGGGGTATGGGGAAAATGTTGCCTCCCTTTTTTAGG

GCCGAAGGGGGTGGGCAAAACCTATGGGCCAAAGGGTGCTTTCCTTATGGAACGTTGCAA

GGGTTTTTTGTTTGGGGGGGAAAAAAAAAAA

>201

CAACTCATTGAACATTACGGTAGTAGCCGATCCTCAGGTCCGTTGAATCCGTGGTAAGTT

TCCTGTGACGTTCCAAATCCCCCCAGCCAGAGTGGGAGTTGGAAAATGGTACCAGAAACG

ACAGTGTCTATTGGAGGCCATCTTTCCTCTGTTCGCGGACTCACATAAGTCAAGGGCGGG

GTTAATGTGGTGTTATCAGCAAAAAAAAAAA

>202

AAAAGTTTCTCCATTTCCAACAGGGATTTACTTGGTTGGCACAGTGACATCAGCCTGGTT

GAAAGATAAGAAGAGACCAACATGTCAGATCTGCTTCTATAAGTGGTGTCGATAAACACT

TCAGGCGGATTACATCATAGTATGTAAACTCAAGTTTCTGATCACAGGGACTTTCTTGTT

GTCGAATCTGTTTCTTGATCAAAAAAAAAAA

>203

GAGTTTGGGGTCGTGGGGTGTTTTCTTTTTTTCCAAGTGGATTATTGTTGTTGGATTTCT

TATTAAGCATTTGGAGGTTCTTTTAAGGGATATGTTGGAAGGGGTGGTGTTGTTTTTGCC

TTGGTGGCCCCAAAAGGGTTAAGCCCAATGGGGTTTTTTTTTGGGGCATGCAATTTTTGG

GAAATGTGAAATTTGTGGGCAAAAAAAAAAA

>204

AACGCTTACCAGGGATCTGGGAAAGAAGCGGCCATAGAGCAAGCACAGGCAGTATTTGAG

GCGGCTTTGCTGTAAAAAGTAAACGGTCACGTGCAATCAGCTTGCTATAATCTAGTAGAA

CCCGAGTGGAGGTTTCTGAACATCGCAAGTAGGTATTGAAGCCACTTGATGTACAGCAGA

AATCAAAAGTTTTAACCCTCAAAAAAAAAAA

>205

AGCAGATAACTAACCCTGGGCAATAAAATGTGGAAAACTTTGGCAAGGCTTCAAGCTTGT

CCGGCAGTAATAAATAAATTGCCAGTTACCATTCGTTCAACCATGCAGCCAGAAGATTGA

TGAAAAGCTGGCAAGTTTAACCTAGAAGGCTTGATCCAACCTTGGGGCAGGGGTTTCAAC

ATTTTCGGAACCCTTTCCGCAAAAAAAAAAA

>206

CGTCATGGTGTGAAAGCGTGGACTGAGCTGTGTAAAGTCAAATGAACAAGTAATTATGGA

TAGAGATGATAGCCCGATGATAGTGTCGTCGCTAGCTGATTGGCAAGACTTGATCGACTG

AACTCTGCTAGTTGCCTGGTTGGAACATCTTCATGTAAAAATACCCATCCTATGTAAGGA

TATGGTTTTGGAAAACTTTTAAAAAAAAAAA

>207

GACTCGGTTCTAGGCCCTAAGCTTTAAGCATCAAAGCTCAAAAAAAAAAA

>208

GTCAGGACCTTCCTCGATATTCGTTGGTGGGACGTAAAGCTTTGGTAGGATGTAAGATTC

AAGCTGTGCCTGAGCCTTTTCGACCGACCAAGGGCCTAAAATTTAGGTATATCCAGTATG

ACATTCGAAGGCTTTCTTGAATCAGTGTGCACTTCAAAGGTGGTTCGTTTGTATCCCAGC

CTCTCTCTCTTTTCACTGGCAAAAAAAAAAA

>209

GGTAAAGAGCGATGTCTGGGCGGATGGTCTGTAATAAGGGCACGATAGCTTGGTCTGCCC

GTGGTCATTGCTGCGAGGGGGACAATTGATATCCATTCTTGACAACTTTCAAAACAGCAT

ATCTGACTGGATGGCATAGGAGTCCTGAAAGGACGTCCAAGGTAGTTGTGCAATATGTAG

TTTATGGAGCGTGACCATTGAAAAAAAAAAA

>210

CCAGTTCGGCCCCCTGAACCTGGCTGAGGACCCTGACACCTTCGCTGAGCTCAAGGTCAA

GGAGATTAAGAACGGAAGACTCGCCATGTTCTCCATGTTCGGCTTATGCCACCCAGTTCG

CCCCTGGACAGTAAAGATCTTGGTAGTGTCCTGTGCATAAAGACCATTAACTGTGTATGA

ACAAAAATAAACGAATTTTGAAAAAAAAAAA

>211

CATTTGTATCAAATACTCCACATCAATTGTAGGCTAGTGCACCGCTAGGGGGCGTTCGAT

TATCGCTAGGTCGGGCCAGGAATTTGGGAAGGTGGACTCCTTAGGTCGAACCGAATAGTT

TTGCAGCTTCTGAAACCTGTACTCCTAAGCAGGCAGCCGATCATGGTGTGTGCTCTTCAG

AAAGCTGAGCAATTGGCCAGAAAAAAAAAAA

>212

AGTTTCGCGCGTTTTGGGGCAAGGTCCCGGGGGCCCCCGGCGGGAGCGGTTTTGTGCGCG

CCCATTTCCGGAAGAACCTGCCCCCAAATTTTTTGGGTGCCCCAGTAGGGGGGATGTTTT

CCCCCAGCGGATTTTGTTTCCGGGGGGGGGTAAACCATTATGTCCAATTGCCCTCGTGGA

CCCTTGGGCAATTCGCCTTCAAAAAAAAAAA

>213

GACAGCCCCCTTAATAAATTTTGGGCGAAGAAAAAGGCTGAGTTTAGCCAACAGTGAGGG

TGACAGGCGCAGGCAGTGAAGGACTTTTGGAATAGGGTCACTTTTTTTTTGGGTTCCTGA

AGTTCTCAAATTACCTGGGCCCGGGGGGAAACACGCAGTATGGAATGTATTGTAAAGCTT

GGAGGGTGGGATATCCCGCCAAAAAAAAAAA

>214

GGTGCCCCCCCTTTGTTTGTGGCCCGCTTTCGGATCCTGTTGTTTTTGTGGGCTCGGCTG

TCTTTTTTCAAAGTTAATCCCTGTTTCCAATTTGAAAATAGTTGGGCAATGGGGGGTTTC

TTTTTTAGGGGGGCCCCCTTTGCCCCTTGGGGGGTTTAACTTGCCCGATGGGGATAAGCC

TGTTTCCTGTCAACTGGGGGAAAAAAAAAAA

>215

ACGGCGGGGCAAATGGGATCGAGCTGGTTAGTCAGAAAAGAGACCCACGAGGGGGTTTAA

ATAGAGTAGGGGGTCCTTTCATTTCCCTGGAGCAAGGGCAAGCGTGCGTTTGTTATTTTT

CCCGGTTATTCAATTTTTCCTTTGCAATAACAAAACGGGTCCAATATGGAAAGGGATTTA

GGTTGCCCATTTTTTCCTTGAAAAAAAAAAA

>216

GGAAAGTTTTGTCCCCGTTTTTCCCCCCCTTTTTTGGGGGCCCCTTTAAATTTTCAAGAT

TTTCTCTCCCCGGGGGGGGGGGTTTTCCCCTTTGGGGGCCCCTGGGGGGGGGGGGGATTT

TTTGTGGGTTTTTTTCCGGGCTTTTTTTCTTTTGAAATTGTGGGGGGATTTTTTTTTTTT

TTTCCCCTTTAAAAAAAAAAA

>217

AGCTCCCCCGAGGCCGACCAGCGGGTTCTTCAAATGTACGCAGCTACGCAATAGGCGATG

GGACTGTGCCGTCTGAGCGACGCGCGGTTTGAAGCGACGCGTACAGGGCACACTGGTGCG

TCCTGGCACCGGAGTAATTTCGTAAAGCAAACGTTGTATATAAGTCAGGATTTCGTACCA

TACGGCATGCGCATGCTGTGAAAAAAAAAAA

>218

CCAGCTTGGGGGTTGTTGGCCTTTGCGGGGTTTTGCTTTCCGGCAACGTTTTCCCGCCTT

CTGCCATTATTAAACCCTTCCCTTCCCCCTTTCCATTCTTCCCGATTGTTGTTGTGTGAA

TTTGGCCCCCACGTTCCCTTTTCCCCATCCAGCCCCCTGCCTCGGTTCGGGGTTTTTCCA

TGAAAGATTTTTTTGCTTGGAAAAAAAAAAA

>219

ACTCGCACATCGATTGTCTACCTGGCATTCGCTTCATGGGATAGGGTGGTGCAACGCTTG

GGAGGAGGCCTGTATCGTCTGTCGTCTTCTCGACAGGCGAAAAGACGCCTTCGTCCACCT

TGGTAGCTTTGCGTCCGAAGAGAGATACAGATGTAGACAAATCCTGTAATGTTTTCCCCC

ATAAATGTCAATGATAACGTAAAAAAAAAAA

>220

GTAAGCTGTAAGTTGCAAAGCTGGCGTCGTTGATTAGACAAAGGTAGATGTTGTATCATT

GTGCAACCTTGTTTGTGGATGAAATTATGTGATTTTTTGCGTCTATTCGTGTCTTCTAGA

ACGGGAACTAGGAAAGAAGTTTGTGTTCTAAACAGCTGGAACATGTGATCGTAAATTACA

TATGTCAGGATATACCGCATAAAAAAAAAAA

>221

AACAGGTTGTTCGAGAAGGATTGATGAGCATCCTTCTTTCCTGACCTTACAAATTTTGCC

CGTTTGTTTTTGAGCTAAGCTTTGCTGCTCTAAAAAAAAAAA

>222

GAACCCTTGGTGGCTATGCGTTGGAATTTAGATTGGAGGTTGGGTAGCTGGTTTGAGAGG

TCATAAAGTCGTTTTTAAATGCTTTTGTCCAGACTCAACTAAGAACGGTCGGTTTTACGC

TGCTGTATTTCAAAAAAATTCATGGTGGCCAGGGCCAGTGCTCCAAGGCACCAATTGAAT

GCGGCCGAATTTTGCACGTTAAAAAAAAAAA

>223

GCGATGTGGTCATTGTGGGGCAGTCCAGGCCTTTTTCCAAGACGGTTCGTTTCAACGTCC

TCAAGGTGATTCCTATGGGCACTTCGGATGGGGGTCCAGGAAAGAAAGTATTCCAAGCCC

AATAATCCATGTAAAGGATAAGTGAGCTACATTTGATGCAGCTTGTATTTGGTTCATCCC

TTTCAGTATTGCACCGCCCCAAAAAAAAAAA

>224

CTATATGAGATATACAGATAACTAGTGACTTCGGGTGTAAGATATGTTAGATGATTATAG

AAAGTTTGTTTGCTAATAGCTAGCTAGCTTTGAATCAGATCATTCAATGTTTTGGAACTT

ATAACGGAGTGAATTTGGCAGAACTTTCAGATTATACCGTCATGACCTATAGAAGATTTA

ATTAAAATTCATTGCCGGGGAAAAAAAAAAA

>225

CAAGCTTGGAAAAGGGGGCCTCATCGGGGTACTTTGGGTCCAAAGTCCTTCAGATATTGT

GGCCCGTTGGATCAAACCAAATTTTGCAGATTTTCAGGGCAGCGAGGTGAACCTTTATTG

ACGGCAAGAAGACTTGGTTGGAAAATATCCGTGTGCCTTGTAAAGAAGAGGGGGGTAAGT

TGCCTTGGGTTTGTCCCTGGAAAAAAAAAAA

>226

CCCCCCCCAGGGGGGGGGGGGGGGGGGGGGGGGCCCCCCCCCCAAGCCGTGGGCCCCGCA

ACCACGGGGGGGGGGGGGGGGGCCCGCACCCAAAAAAAAAAA

>227

CTTTGAGAACCATTATAACAAGGCACCCTTTTTTAAACCAACCCCCCTAAAAAAGGGAAA

AACCCCCAAAAAAGGGGGCCCCCCAAAAAAAAAAA

>228

AGACTGGCCTTAAGGGGATGAACGTTGCCCGCTTTCTATAGTGGAGTTTGCCAGAGGTAA

CCCCCGGGGTTTTGCAAGCCAAACTTTTTAACAAAGAGTCCTTTTTAGCATAGAGGCACT

TTTTCCAAGGAGCCTTTACTTTTTGAAAACTTGGGAAGCTTTTCGGGGATCCACCCTTTA

TAGGATCGGAGTTCCCTTTTAAAAAAAAAAA

>229

AAGAAGCATAGCAGCACTGCAGGACCTTCGGCGGTTCGATTTGTAGATAAGAAGTTGCTA

TTTACATTTGACATTCTTTGACCTCGTGTATGGGGAGGCAGGCTAGCTCAGGACACAGAT

GTATTTTCAAGAGTCCTGGGCTTGTGGCACGAACGCTCGCTAGGCAACCAACTGATTGAA

GCAGATGCTTCTTTACACCCAAAAAAAAAAA

>230

AATAACTTGCTGTTTTTTGAAAGTCGCAGTTTGAACGTTAGTGATGGGGCCAGTATTTTT

TGGGCAGAGCCATGATGAAGCAAGACCTTGGGACAGCTTTAAAGACAAAATACATTTTTC

AGGATACAGATTAGTGAATTGGTTTTTTAGAAGAAAGTAATTTGATCCTTGTTTGCATAT

TAGAGTAATGCTTGGGGTTTAAAAAAAAAAA

>231

GTGAAGATGCAGGCTAGAGCAACGGAGGAGCACGGAGCCGCTGAGGCTCGGTTGCGAGTG

GTGGAGGAAGCTGGCGGCGGCGCAGGCGGAGCTGGAGAGGGTGCGGAAAGAGAGTCTGGA

TGTGCGGGAAGCAGTCCAGGCGTCGGCGGGAAGCCTGGAGGCCAAGGTGGCAGAGCTGCA

ACAGGAGCTGCGAGAGGCTCAAAAAAAAAAA

>232

GAAGTTCATTGGAAGCTGTACATCCCCAAGACAACATAGCACCTCTTATTCAAGGATCCT

CATAGGAGTACATAAAGCAGAAGGCAGGTTTGTAAGTTGAGCATTGAACACGGGGCACTA

GCGTTTTTCTGTACGTACAAACAACCACACCAACATGTGTACCGCCAGAAATTCACTAAA

AGGATATGTTTTGTGATCCCAAAAAAAAAAA

>233

AGGGGGGGATTCCCGGGGCAAAGTTTTTTTAAAGGCTTTAGGGGGGGTTCGGGGGGTTTT

TTGGGGGCCAAAACCCCTTTCCTTGATCCCGTGGCCGAGAGGGATGTTCCCCGGGCTTTT

TCCCGGGGGCAAGGGGGGTAGGGTAATTTGGGGTTAGCATAGGGGGGGATTTGGGTTTCA

ATTTAAAACCCTTTTTTCGGAAAAAAAAAAA

>234

GGGCTTTTCTCCTTTTTTTTTAAAACCCCCCGGGCCCTGGGGGGGGGGGGGGGTTTTTTC

AACCCCCCCCGTTTTTTTTTTTTGGGGACCCCCCCCGGGCCCCCCCGGGGGGTTTTTGTT

TCTTTTTTTGGGCCCCGGGGGGGTTTTCCCTTTTTTTTTTCCCCGGGTTTTTTGGGGGGT

TGGCCGGGCCCCCCCCCCTCAAAAAAAAAAA

>235

AAAAAAGAGCCCGGTCAACAATTTTGGGCCCCCCGGGAAGTTGGAACTTTTGGCGGCAAA

TTTTAGATTGATGAAGACTTTGTAGAGGTCCCCCGTTAAAGGGAAAATGACCGTTTTTTC

GGGCAATAGACAGCATGTGCAGGGGGGTTTGCCTTTACAAGGAAAGGTTTTGTGCAGGGC

CCCGTACATGGGTTGGTTGGAAAAAAAAAAA

>236

CCGAGACATCTAACTCCTAACCCAAGCTTTGCAAGCGCAATAAGATGTGCCATAATATTC

TGGAGGTAGGAGCCACGTAGCTGAGACAAAACTCTTGCTGATTTCCAAGCTATTGTGCCG

CCATTGAACAGCCGTAAGCTTCCGCTATTTTGCTGAGCTTATACCCGGCCACTAATTAAA

TACGCTAAGTTGTTTGTTTTAAAAAAAAAAA

>237

TGGGGATCCATTTTTTTTAAGAAAGAAAAGCACCCCCCCGGGGTCCCTTTTTTTTGGCCC

CCCTCCTCAGCCAATTTTGGGGTTCCTTTTTGGGGTGGGGTGGTGTGGATTCTTTTTTTG

GGGGGGGAGGCCCTGGGTTAGGTTTCGGGGTCCGGCCGTTGTTTTTTGGGGGGAGGGGGG

TTGGAAATTTCTTTGGGGCCAAAAAAAAAAA

>238

AGATAAAAGTGTGGTCAAACCGCGGGACCATTTCCCCGAATGACATATTCGATATGCAAG

CACGCGCATAGCAAGTACAAATTCAGTTCGCAGTTAGAGCAGCATTTTGACGTTAGGAAA

TCGGGGGGTATCCCCCTGAACCAACTAACCCGATGCATTCCGACCCGAAGTTTTGTGCAG

CATGTAACGATTTTTGCCATAAAAAAAAAAA

>239

GGCGCTGGCCGCCAGCATAGTCATGGTGCTGGCGCACGCGTCATTCCGGACGCCGAACTT

AAAAGCCCGCCTCAACTCCTTTAGAGAAGAATTCCGAAAAGTCTGGAGCGAGTACAGTGA

GGCCTGATCACAAACACACACACCGTATGTCCTCAACTACCATGGCACATGTACTTTAAC

CTGTATGTAAGTCAGTGTCCAAAAAAAAAAA

>240

TTTCGCTGAGCCAGTACATCGTCGAGTAGGAACAAACCAGATTCTGATTCTAATCCATGA

CTTTTCTAATTAGGTCGGGAGCTGCAAAAACTATGTTGAAGTTGGCATTGAAGCTCGTCA

ATAATCTCGCGTTGTCAGTTTGGCTAAACTTTATTTCTAACTGTTGCACTGACCTAAGCA

CATGATCGCTGAATCACTCGAAAAAAAAAAA

>241

GCTGGGATAATATGGGCGGGGGGATATTCCCCCTTTGGGTTTAGTTTATCCAAATAGGAC

CATTTTGGGAGGGATATAGGACAAAAATTGGGGTTGAGCTTAGCGGGCTTGGAGTGTGGA

CCATTGACCCCCTATAGGGGACAGTTTAACCCAGACGGGATTCCCGGGCACTGCCCAAGA

ATTGCAAAGCCTTTTCAACCAAAAAAAAAAA

>242

CGCCCGTGGGGGGCGCTCCCACATAGTTGACCAGCAGACGGCCGCGAAAACACAAGAGAG

AGGCAACCAAGCAATCGCCTCCGGGGGTGGGGGGAAAAAAAAAAA

>243

CAAAGTTTCCCTCCCACCCCTGGGGGGCTTTCCCAAATGGTTGCCCCCCAGGGGGCCCGG

AAATCCCTAGGGATTTTTTTCCCGCCCTTTCCTTCCTGGGGGGAAGGGCTCCTTTCCGCT

CAAAAAAAAAAA

>244

CTCTGGGTTCGGCAGAAAGGGGCACGCTGTGGGAGATATTCCCGGAGTCCGGTTCAAGGT

GGTGAAGGTGGCAGGCGTGTCACTTCTGGCCCTGTTCAAGGAGAAGAAGGAGAAGCCTCG

GTCATAGAGCATCTCGTCTCTCATGGTCTCGGCCGACACGCAACTTTGTACACTTACCTA

AAGTTCACCCACGTTTCCCGAAAAAAAAAAA

>245

GTCCGGTTTAGGGTTTTTTGAGGGGGTTTTTAAAACCTTGGGTTTTTTCCTGTGGCCCGG

CGGGAAGGGGTTTTTTTTTTTTTTCCAGGAGCGGGGGGTTTTTAACCGGCCTTTTTTTTG

GGGGCGGGGGGGAATTTGGGTTTTCCCTTTAAAAAAAAAAA

>246

GTTTTCGTTTTGGTTTTACTGGTAGGCCCTTGCAGGCAGTGAGGGATCATCGTGTAGGTC

GGCTTCTGCTAGCACAGGGACTAGCAACAGGTCCTGCTCCGCTTGAGCAAAGCTAGGATA

GTTCCACATGCTGAACACTGAAGTACAATATGCGTGATTGTACTTCAACAGAGGTGATAT

GATTACTGGCAGGTGACTTCAAAAAAAAAAA

>247

AACCCGGAAGACGGATTGGGGTTCAAGACAGGGTCCGTCTGCCTTTGGCTGGGCTTGTGG

ATCAATCCCTTTTAACAATTGTCGATTGCGGCTACCATTCTTCCAATGGGGCAGCTTAGC

TCTTCTCCTTGACAGCGGACTGCCAATTGTGACTTCACCACCTCTACACGTATTTGGGTG

CCTGTTCCAGGCTACTTTTCAAAAAAAAAAA

>248

AGTAGCCACAAGCAGAAAGTCGTTGAGTTAAGGGGCCTCATGTCAATCCGGTGATCAACA

TCTGAATGCGTGCCAACCAATTTTAGGTCACCCGGGACTGCACTTGTCTCAATAAGGCAC

AGTTGGCAAGAAAGGGAAGAATGTGTTTTGACAATCCGCACATCTGCAGTTGACCATTTT

TGAAGCATTTTGGTTCACTGAAAAAAAAAAA

>249

AGGTTTTTGTGAGATATGTTTATGACCAGGACTTGTTTCCCCAAGGATTAGCCGATTTGT

GTTGGGCTTTCGAAAATAAGGAACTTCAAACAGATTTCAATGGGAATTAAGGCTTTGGTC

CATTCAACGGGGGGAATTGACAACTTAATGTTGTCCTTGTTCTATAAACCCCCGGTTTTG

CCCGGATACATGCCCCTTTTAAAAAAAAAAA

>250

TTTCCAAGTTTGGGTTTTTTGTTCAGGCCCTTGTCACTGGCAAGGGCCCCCTTGCCAACT

TGAAAGGCCCCCTGGGGGGCACCGCCGTCAACAAAGCCTGGGGTTATGCCCCCCAGTTTG

CCCCTGGGCAGTAAAAATTTTGGTAGGGTCCCGGGCAAAAAGGCCCTTAACTGGGTTTGG

ACAAAATAAACGAATTTTGGAAAAAAAAAAA

>251

CAAGTCTCTTTGGGTAGTCTTCCGAGCATTCACTTCCTGGGGGTTATCGGGGGGTTTCGC

ACAAAAAAAAAAA

>252

GGGGGCGGGCTCTGGATAGGGGTTGTTAGACTGGCCTTCGCTTCTTGGGGGTAGGGGGGG

GGGGGCATGTTTTTTTGGTGGTTTTTTGGCCAGGGGAAAAGAGGCCTTGTTCCCCCTTGG

TAGCTTTGCGTCCGAAGGGGGATCCAGATGTGGGCAAATCCTGTAATGTTTTCCCCCCTC

CATGTCAATGTTTTCGTGAAAAAAAAAAA

>253

CGTTTTGCCCTTTTCCCAACCCGAGTAGCATGGGGCCCGGGAAATCCCGTGTGATTTTGG

GGGGGCCCCCTTGTAAGGGTAAATATTCCTGGAGGGCCGATAGCGAACTTGCAAATGGGG

GGGGGGGGGGTTTTTGAAAAATTGGTTGGGTATGCCCTAAAGAATGTTGTCCCTTGAGTT

GTGTGCACCTTGGGGGGTTTAAAAAAAAAAA

>254

AGAAGCAGGTTAATAACCTGTGTGAAAATCGTCAGACCCCGGATCAGACCTCAAAGCAGG

TCTTTACGACCTGTAGAAATATCAGACCTCGGATCAGACTGTAAACCAGCTGGTTTTCCA

AGTGCAAATTGAAACTTTGCATGTGACACTTCACTATATCCGAAATTGTAATTTTGCATG

TGACAAACGTCACGGTATTTAAAAAAAAAAA

>255

CCCAGCAAAGGATTCTCTGTTGAAACTTGGCCCATTTTGTCGAGTTAGTTAGGCTGCTGG

GAGAATCCAACCCGTCAAAAGAATCCTTTTACTGAATTTTTCTCGCGGAAAAAAAAAAA

>256

TTGTCAAGTGCTGGTAAAGGGTATATGTAGTTATATTTAAAGGGCTTTGGGGATTGCAAG

AGGTAAAGGACTTGCCAGTTTTGGCCCTATACAAGTAAAACGACGGGGTAGAAGACCTGG

TAGTACAGCTCCCCTTAACCTTTTTGCAACAGATGGGAACAGGGCCAAATCAGCAAGTTG

TTGATTGCTTGCTTTCCGGGAAAAAAAAAAA

>257

TTTGGGGGGGGCGGGGGGGTTTTACCCTTTTGGTTTTCCCAGGGGGTTGGGGGAGGGCCC

CCCCCCCCAACCGGGGGGTAAAGGTTTGGTTTTTCTCCCCCGGGGGTTCCTTTTTTTTGG

GCCCCCCCCCCTTTTTTTCCCCCCCCCCACCCCCTTTTTTTTCCCTTTCCCCCCGGCCAC

TTTTTTTTTTTTTTGGGGGGAAAAAAAAAAA

>258

AGTTGTTGCTGTGTCGTAGGTCCTTGGCATCCTCGATACCCCTGGATTGATGGCCTTGTG

GCAGGGGATAATTACCTGAAGTTCCTGAAGCTTTGAACTTTTGTTAAAATTCAAGGATGG

ACCTGTCCTTTCATTCCCAGGGGTTAGAATTTTTAAGGGTTGGGGGATTTCGGATCCATT

GGGGGGAAAGTTTTTTTTTTAAAAAAAAAAA

>259

AGGTCGTTTCGTGGCCACATGTTGAGAAAAGGTAGACGGTTGCTAGCCTGCTATCAAAGC

TGCGGTATAAGTTACACACGTACGTTGAATAATGAGGGCAAGCTAGCTAAATATTTGAAA

GTCTTCAGGATCATTGCATGGGAGCTCTTGCCTTGGCCCCCTCTCGAAAAAGTTGCAATT

AAATCTGCAGCGGTGTTTTTAAAAAAAAAAA

>260

TTGGGGGGGGGGGGGGGGGTTTTTTCCCCCCTTTAAAGTTTTTTTTTTGAAGGGGGGGGG

TGGGGGGGTTTTTGGGACCCCCCCCCCTTTTGGGAAAATGGGCCCCCCCCCCCCCCCGTG

TTTTTCCCCCCGGGGGGGGGGGTTTTTTTTTCCCCCCTGGGGGCCCTTTGGGGGGGGCCC

CCGTTGGTTTCCCCCCCCCCAAAAAAAAAAA

>261

CAGTATTTCGTTTAGCGGGCCCCAAGTTCTTGTAGACCGTTTGTTCTAGTGAGCATCGAT

TGTGCATAGTAGAACTCGCAGTGGTAGATCGAGTAGTAAGAAAGGTAGGTTGTACATACG

GATATGTTCACTAGTAGCAGTGCCCCGGGGGCAGAACTGTCGGCATAGACCTTATTGAGA

GGAACATGGACCTTTGACTTAAAAAAAAAAA

>262

CCCCTCCCTGGGGGGGGAACCAGGGCCGGGGGTTCTTTTTTGCGGGGGGGGGGTTGTTTT

GTCCCCCGGCTCCCCTGGGGTCCGTAAGTCGGGTGGTTTGTCCTTTTCCCGGGGGGGTTG

GGTACCCCGGGGGCGGGGGCCCTTCTTTGTTGGGTTTTTTGGGATTTTGGGGTTTTTTGT

TTGGGTTGTTGGGGGTTGTTAAAAAAAAAAA

>263

GGCCTTTCCTTTCTGGGGGGGGGGGGGGCCCCGGGTGGGCCCCGTTAAAGGGGTCGGAAG

GTTTTTCCCCCCCAAAAAAAAAAA

>264

GTGGAAAGTTGGTGTTCCTTTTTGGAGGTTTGCTTGCCCTTACCCCGGGAAACCGCCGGT

TTTTTTTTCCGCCCCGTTTTTTAGCCTTCTGGTTGGAAGGGTGCCGGCCGAAATTGGACA

AAGGGGAGGTGGGTTTTCCCTTGGGGTTCCCCCCGGTTGGGCCCGTCCCGGTTTTGTTTC

GCCAAAATGAAAGGCCGGGGAAAAAAAAAAA

>265

CTATTTAGGTTTCCCCATCCATTTCTCTATGTGGGCACGGGGGTTTTCCGAGCTTTCCCT

TTTGGGGGGCTGGGGGGGCCCCCGAATTCAAAAAAAAAAA

>266

GGGGGGGGGGGTTTTTGGGGGGCCGGGGGGTTCCGGGGGGGTTTCCCTTCGAAAGGGGGC

CCGGGGGTATTTTTTGGGGCCCAAGGGGTTTTGGGGGCTTTGGGGGGGGGGGTTTTTGGG

GGGGGGCCTTTTCCCGGGGCAAATCCCGGGGGGGGCCGGAATTTTTTGGGAAGGGGGTTT

TTTTTTCCCCCGGGGCCCCCAAAAAAAAAAA

>267

GGTGAAAAACCCCCCCCCATTGGCCCCCAAAAAAAAGAAAAAACCCCCCTTTTCCCCCCC

GGGGTTGGGCTGAAAGAAAAACACCGGAGGGATCCCCTTCTTCCCCAGGGGAGGGTTCCC

CCTCTTCACGGGGGGCAAGAACATGGCCCCCCCAACTCCCCCTGGGAGCCTGGTTAGGGG

AACCTTGATTCCCCTCCCTGAAAAAAAAAAA

>268

TGTTTGCCAGGAATTAAGGACTTGGAAGTCATTAGGCGGTGTCAAATACTTGGTGCTAAA

TCTGATGAGCTAGCCAGCGGATCATCAAGTTGAATGCAACGGCCTTGAGTCCTGAGTCGG

AATCCAATCAGGACAGGACGTCTACCTGGACGTGACAGGAAGATATGCAAATGTGATCAA

TCCCAGTGTTTGTACCTGTTAAAAAAAAAAA

>269

GTCTACCAGGCATTCGCTTCATGGGATAGGGTCAAGTAAGTCTGTAAACTAACGTACGCT

GAAGTCGTCAACATTACTGTAGTTTCTTTTAAAAAAAAAAA

>270

TGGCCCCGCCGGGGGGGGAATGCTGTCCCGTTGTGGCCATTTTCGTTTTAAAGCTAACGT

TGCCAACCATTCCTTGTTAGAATTTTTTGGAAAGGCCACCCCCCGGGCCCCTTTTTTTTT

TTTGTACCAAGAGCCCCGTTTGGTTCCCCTTTCCCGGCCCGTAGTTTTTTTTGTGGGCAT

TTTTTTTGGGGGGGTTTTTTAAAAAAAAAAA

>271

GGTTTGGGGGGTTTCGTTTTTTTCGAAGGTTGAAGGGGGGGCTTGGGAGATTTCCCCGGG

TTTCAAGTTTTTTTTTTTCCAGCAAAGGGGTGGGGCCGGAGCCTGGGGTAATGGGGGGGG

GTTTCTTCCCCAGTGGAAGTTTGGCCACTTTCCCGTTTTTTGGTTTTTTTTTGGGGTAAG

GCTTTCCCAGGTTCCCCGGGAAAAAAAAAAA

>272

AGTGCCCTGATCTAGTTCTGCGGCGAATGGCGGGTTCGTAGTAAGTCAGACGTAGTGATC

AAACCCGCAAAATAGCGGTTGCTCGTCGAGAAAGTACTGCGTCTTGGTTGCGAATTGCTT

GCGGGCACCGCAGTCGATTAACGGGCTCATGCCCACAGCTTGTACAGCTTTCATTTAATA

AGAAGGCCTCGTGTGGTATTAAAAAAAAAAA

>273

GTAGTGCTCCCTGCTCCTGGGATGTCATATGTTCCCTTGAACAAAAAAGGTTTACGCCCC

CCCCTTCAACCCTGTTTTTGTGCAAAGCTTCCAAGGGAGAGGTCCCAATTCAGAGATCTT

TGGAAACCCCAGGGAACATGGGAGTCGCCTCCGTACGAACTATTATTTGGATGACTTTTG

AAAAGGGCCTCAGTTCATTCAAAAAAAAAAA

>274

CCCCCCTGTCTTGCCCCCTCAGGCTTTCGCGTGGCACCCTGGCTTCGGTTTGAGGGGGGT

TGGGGCCCACACAAAAAAAAAAA

>275

GTTTCCCAGGCATTCGCTTCATGGGATAGGGCCCTTTGAGTTGATAATTCCTTCTTCCAT

TTTCAAAAAAAAAAA

>276

GTCTACCAGGCATTCGCTTCATGGATAGGGGTCACATTCAGAAGAACTTAGGCGAGGCAG

GTGGGACGCTTTAAAAAAAAAAA

>277

CCCGCCTGTACTGCGGTCCTCTGCCCGGCATTCTCTTCATCGTTGATTGCGTTGATTCGC

TGTACTGTTGTAGACGCTGCCGACTATGCTGGTGCTCTTGTAAAAAAAAAAA

>278

CTTTAGCTGACGGTAAAGCGGGCTCGGACATCCAAATCTTGTTGGAACGCGGTTCATAAT

GATCCCTTGCAGCGGGTAAGACGCGTTTCGAAAACTGCACTGTTGTACTTTAAGAGCAAC

GTTAGCAACGTTTATACTGGCATGCAGCATATAGAAAAAGTAGAATCTGCGACACGCTGT

TAAACTTCGTTGTCAATCTCAAAAAAAAAAA

>279

ACCCGTGTAAAAACGATTTTGGCAGCCGGTTTAAAGTGCCAACCTGTTCCGGATTGCCAA

AGCAAAAACATAAGAAGGATTCAAGGAGACTGATTTGACATGAATATTGCTGCTGTGTAG

GATGTACCCAAGTTTGTCATCATTGTACTTTGGCAGGGCTCAAGTGTACTGGCAGGCTGT

CAATTTTAGATGGTCTGTGGAAAAAAAAAAA

>280

CAGCACGTTAAGGTAGTGACGCCTTCTGTACCATGCGATTCTGTGTAGTGGCAGTGGAGG

ATTTGAAAGGAACCATATTTGCATGCGTCTATGTACCCTGATCTGGAGGCAAGGCTTCAG

AGAGGCAGCAGGACTAAGTTTGTCTTTGTGTATTTCAATGTTTGTCGAGGCAACATAATT

CTGGAGTGGTTCTGCACCGCAAAAAAAAAAA

>281

CCAAAAATAAATTTTTCAAGGGGGAAAAAATTGGGAAAAAAATAAAGTAAACAGCACGGG

GTTTCGAACGGGGTCAAAAAAAAAAA

>282

TTTAACAGTTTAAAGTTCAGGGGGCCCAGGGCAAAAAGGAGTGTTTTCCGAAAGGGAAAG

TTACCCAAGGGGGGAAAAAAAAAAAAAAAGGGACGGGGCCCCCGGAAACATCCCAAAGAG

GGGTTTGGATTTTTTTTTGGGGGTCCCCCCCAAAAAAAAAAA

>283

CCCCCCCCCCCAAAAGGAACCCCCCCCCCCCCGAATCCCCCAAAGGGCCCCCAAAAAAAA

AACCGGGGACCCCAAAAGGCCCCAAAGGCCCCGGGGGGAAAAAAAGGGGGGGCACCCAAA

AACACCCCCCCCCCCCGGGGAAAAAAAGGGGGACCCCCCCTAAAACAAAACCCCCCCCCC

CCCAGAAAAGAAAGGGGGGGAAAAAAAAAAA

>284

GGGGATGGTGGTCCCCTGGCGGATTACACCTTTCGGAGGGGGTGGCCCCTTCCCTTGGTG

CTCGGGTTGGGGGGGGGTTAGTAATGTTTAGAAGGATAGTCGTTGATGGGGGGGGGCCCT

TTTTGTAAAGGAACCCCTGTCCATTGGGTAATTTCGGAATTATTGCGGCAATAACTGGGT

CCCGCAATTTTGTTGTTTTCAAAAAAAAAAA

>285

TATGCGGTACGCTCGCCCCCCCTCCGGCCGCCCCTCCGGGGCGGTGGCCGCCCCCCCTTA

TCGTCCTTGGGGGGTGGGGGGGGGTTTTCCCCCCCCCTGTTTAGGTTTCCCCGGTCCCAA

AAAAAAAAA

>286

TTTTTTGGTTTTGGGGGGGGGCCCCCCCCCGGGGGTTTTTTTTTTCCGGGGGGGGGGGTT

TTTTTTTTTTTTTTTTTTTTCGGGCCCCCCCCGGGGGGGGGGGTTCCTTTTCCGGGGGGG

GGGGGGCCCCTTTTTTTTCCCCGTTTTTGGGGGGCCCCCCCCCCCCCTTTTTTTTTTTTT

TTTTTTTCTTTTTTTTTTTTAAAAAAAAAAA

>287

GGGGTGAAAACCAATGGGGGGGGGGAATCCGGTCAAGATTTCCTCCCCCAAAGAAATTCA

GGTAACAAAAAAAAAAA

>288

GCGAATTAAGCTTTATTGAATATGAGATCCTGAGCAGTCGACGTCAGGCTTGGTAAACAT

GCCGCAGTACTGTTGGAAATTTCGAGTTGCATCGGACTGCCATAGTGCAAGACGTTATCC

CAATAAATACCAGTGACCTTATTGTAGTCACCCGCTATCAAGGTGATTGGACAGCAATCT

GGAAATTGGAGTTGACTAGTAAAAAAAAAAA

>289

AATACCAACCGGGGGCAGGGAAATCAATAGGGAATATATACCAGGCATTAAAATAAAAAG

GGAAAAAAAAAAA

>290

ATCGAGGCGGCAAAGGAATCAAAAGCACTGCAGAAAGCAGCCTGATTGCGGCTTACCTAT

AGGACAGTACAGGTGCCCAAAAGCATGATCAGTATTTCTATATACTAATTAATCAAGCCA

CACAAATTAGGTGTACATCACACGTACATCATCTGGTGACGGGGTTTGTTTTGTCTATTT

AACAAATTGATTGGAGCGTGAAAAAAAAAAA

>291

GCCTCCCCAGAAGATGAGATCAGAGGGCAGGCCAACCCCTTGATTGTCCTGAGGGGTTTG

TTTGTGTACAATTGTGTAAGCACGCTCGCATTTTGAGGCCGCCAGATGGTGGCCCCTGCC

CTTTTGACCATGTCAACCTGGCATAATCCTGGGTTTTTGCCCTGCTAGGTGGAAAAAAAA

AAA

>292

ATCATCTCTCAATCTCTTGAGGGTCATCTCGGAACAAGAGGGGGTTCCAATTGAGGAGCT

AAATGCTGGCCGCGTTGTAGACTGGTTCGTCAAAGATAAAGAGAAGCGGAAAGAAGATGT

GGATTCAGCGGTCCTGAAGTGGCCGTCCATGCCTGATGACGATTTCCAGGAATCTATTTG

GTAGTATTTTTTTTGATCGGAAAAAAAAAAA

>293

TTCCCCAGACTGTTTGTACCATATCAACACTGTCTTAACCTGCAAGTTTAAGAGTTGCTG

GATCAGGCGCAGACTCCTATACGTGCACGAGCAAATATTGCAGCTGATGGGACACTTAGT

AAGTAGGTAGGTACAGGAGCTACGGTAGGTCTCGAGTCCCCCGCGCTTAGCTTTGCATCA

AAGTCGTAACATTGCACGGTAAAAAAAAAAA

>294

GTTTACCAGGCATTCGCTTCATTGATAGTCTGACTGCAAGGTAGGGGACCCGAAGAGGTT

GGGATCAGAGATTGTGAAAGGCTGCACCATTCCTTGCGTATGGAACAACATAGGTACTCG

CGGCACAGAGCTGTGACTTGGAAGGAAGCCACGGGCCCAAACGATAAACAATAAGCAACG

TTTTTTGTTGTCCTTTTAAAAAAAAAAA

>295

CGGTACCTTTGGAGTTGTCTGGGCCCTGTACTACACCTTCAGCAAAGGCCTTCCGGAGGG

CGATGACGACTCTGGTCTTTCCTTGTAGGGTTAGGCCGAGTGTACAATTTGTGTAATTTG

ACCTTAAGAGACTGTCACGATTTGAGCATTGAAGAACCGAAGTTTGTCATATAACGCGCA

CACCAATGCCCCTTCACGCGAAAAAAAAAAA

>296

TTTTACCCGCCCTTCGCTTCATGTAAAGATGCGTATTGGCCCCTAGGTTATTAGGCCCCC

CCCTAAGCTAGGGGTCATTGCTCTTCCAAAGTACGCTTCCAGACTGCCAGTAGCTGCGTG

GTAGAGGATTGATCCTCCTCCATACCAATATTGATTGGGGAAATATGTTTGATCGATTTT

CCCCTTAAAAAAAAAAA

>297

AAGTCATTTCACATCAAAAGGGGACGCATCAGGGGATTCTAGATGACTCGTAAAATTGAT

CTCAAACAAACCTAGTTAATTGGATGTACAAATCATGATGGCATGCCAAGGACATTCTAG

ACTACTGAATCTGGCCGGTCTCAATACAGGTCTGTTGGGTGTACAAATTATGATGGTCGG

CATGACAGTGGCTAGTGCGCAAAAAAAAAAA

>298

AGAATATTTGGCTCGAGCCGCAGAGACGAGTGGCCACTAAGTAGCTTAGACCTATTCTTT

TTTGCCTTAAGAGCATTTGGCAAAGTATTATATTCGTCCATCTTAAAGGATTTTGGCGTA

AGCAGTTCCAGTTGGGCGGGCATCCTGACATTTGCCTTTGTATTGTTCAAAGACTTGTTA

AACATTACTTGAGTCTCATCAAAAAAAAAAA

>299

TCCCTGTTTGCACTTGTACGCATTGAGGTTGCTTGTAAAATCGGACTCACCTTTGAAATC

GGTCGTACTATGATCCAATCTCCTTCACATACGAGTGCAGCTTAGTAGGAGTAACAAAGC

CATGGAGACTTGTCTCGACATATCATATCTTGAACGATTGTGTTTTGCTCCATTCTAGGA

GTTGAAACGCCACTCAGGCCAAAAAAAAAAA

>300

CACGCCCGAGTCCAAACTAGAGACTTTCGGGGGGGTGTCCAAGAAGCTCCCCGGGAAGGA

GGTCACGTTGGAGTACCCCCCGGAGCAGCAGCAACCCACCGTTGCTTAGGTTGTTGTTCG

TCTTTGCGGGAACAGTTTTCAACCGGGGTTCTTCCCGGGCGCTGGAGTCAATCGGGAATG

GAGGCCGGTTTTTTTCCCCCAAAAAAAAAAA

>301

TTTTTGGGGCCCCCCAATCACTGGTGTTGCGGGAAAAACAAACAGAGAGGAGACAAAAAA

AAAAA

>302

TTGTAACAAAAGACCGGACGGATAGTGGTTTTGAGTAATAAGCATTGCTAGTTTCAATGT

ATAGATTGCGCATCAATGCCGTCAGCCTCTGCTCGTTACTGGGGGAGTCCTGGGCACCAG

GCCAGATAGTCCCCATTTTAATTTAGGCACTTACAAAACCGCCAGCAAGCGGCTTCAAAT

TCCGCAGGCGCTTTGAGGCCAAAAAAAAAAA

>303

TGGACAGATATTTGATACTTGAGACCTAGCACTATTTGGGAGCTGAGTTGGTAACAGCAA

ATGGAAGAAAGATGCCGAGTTGATGCTCTGGGGGCTGGCAAGGGGACTTTGTGACGCTTG

CTTAATGCAGTCGCTAAGGTTGAATCCAGCTAGTCTACTTATTAAACGAAATTGCAATCT

ATCTTTTGGTTTTTGCGGACAAAAAAAAAAA

>304

AGAAGGAGGAGGTGTCGCAGGAGAAGATTGATGCGCAGAAGAAGGTGGACGAGGCCCTTC

TGCCCGCCATTGAGAAGGTCGCTGACCTGAAGAGCTACCTAGGCGCGCGCTTCTCACTAA

CGTCTGGTGTCAAGCCCCACGAGCTGGTCTTCTAAGCGGAACCTTTGGAGCGTCTCGAAC

GTAACCAGAGTTTGTCTCTCAAAAAAAAAAA

>305

AGGGCCCTTCAGTTTGTGCCCTCGAAGCAGATGAACACGTGATTGCTTAAAAGCCCGTAG

GTTGGACGTCATACTAAAAAGTACGACGTACCTAGGATGCTGAGACATAACTGCCCCAAT

CTCGCAGAGAAGCGGTTTCATGCAAAAATTGGTGCGTAAGCACGGACTCATTTATGAACT

CAATAAGACAGTTTGCGATTAAAAAAAAAAA

>306

TTGACCCAAAAGCTTTTTTTTGACGGGTCGCTGGGGAATTTTGGAACCATCCGGGGGATT

ACCAATCCCTGCCGGGAAAACCCAAAAAAAAAAA

>307

GCACAGCGTAATTTGTGGTGTAGAACTGCCACAGACGGAACTTTGGGCGGAAGAGAGCTG

GGAAGAGGTCATTGGGCCACGATCCCAAGTAGCATTTCTTTGGAGCAGACCATCTTTGAC

TGGACAGAATCCTACTCAACATAGTAGGGCCGCCTAAGGCCCTCGCGAAACTTCTGTCCT

ACAAGAGACTGCTTGGTTTTAAAAAAAAAAA

>308

AACAGATCTTCACAAGTCGCAATTTCCGGTTGCTAAACGGGAGAGCACTTGGGGGGCCAT

GTTGGCAGTCGTCAGATGATTCCCCAAAAGAGGTCTTCTTGTCTGAAACCGAGGAGTGTC

ATATGCTAGGACCCATTGATTCGATTGTTTAGTACGTGTTAAGGCTGAAGGATCGACTAG

CTCATACAAATTGTGCAAATAAAAAAAAAAA

>309

CTAGGTCCCGGCGCTTTTTCCCCTTAACAACGTCAATGGTTTTGCAAGGTTTGCCTGCTT

TTTGATCCCAGATAGGAAGCCCCCGTTTCCTTGGGGAGTTGAGCTTTTTGATGATGAGCT

GTACTCCTGGTAGGTTTTGCGCTTCAACTTGTTTGCCCCGTCCTACTGTTTTTAATTTTT

CCCCGGGCCGGGGTTTTTGGAAAAAAAAAAA

>310

CAGCTGGTCGATAGTCCTTATTAATAAGATTGATTTTAGTGCTTCTTCAATCGACGATCG

ATTGCTTTCGAAGAGTCGGATCCTCCTAGTGTGTATGTAGTTGACGCAAGCAGCATTTGA

ATCGACCTGCGTTCAGAAGTCAATTGAGATTTAGACCGTATTTGTACCATATTGGACTCC

ATGATATTGTTGCGCAACTCAAAAAAAAAAA

>311

GAAGGGCCAGTTGAGATTGATATTGAGATCGATTAACGCAGCACTCGAGCGGACCGTTAG

AGTGCCTATGTTCATAGACATTTGATCCCATCGTCAGATCTCAATGACTTGTACATACTG

TGCTAGATGCATATGCCATAGGAAGTAGCACACAGAAGCAATGAGTGCAATGTCTCGTCT

TTGAAATTTGTCATCGTTCGAAAAAAAAAAA

>312

TCGCTCCATGGGGGAGGGAGGGGGAAATTTTTCGTTGGAACTATTAAAGCTGGGAATTAA

TCAGCCCTGTTTTTCCAAAAAAAAAAA

>313

AGGGGAAAGGATTCTTATATGGAGGGTTTTATTCATGCTAGTGGTGCTTGTTTTGAAACA

TTTGTTCTTCTGAATGAGGAGTTTGAAAAGTTTGAAAGCCTTGTTACTTCTACGGATGTC

ATGTATGTTGCTAGGCGGGTGAGTCTAAGAAGAAACCAAGATAACACAAAGTTGCAATCA

GATTCGTTCGTTCGACATTCAAAAAAAAAAA

>314

GTTTACTCCATCGCGTGGAGCCCCGACGGGAGCAAGGTGGCAACGGCGTCAGACGACAAA

ACTGCAAGGATCTGTTCAGTTAGGGGTCGTGCAGATTAGGTCACGCAGAAGCAGGGGGTT

CCTCCAGGGTCTCACTCTGTTGTACATAAAACTTACAGTAGGCAGAAGGATGCATACCAA

GTTTGTCCATGCTATCTTTTAAAAAAAAAAA

>315

TGGGAAAGGGGCTTAATTTTTCCTCTTTGGGGGATCCGGGGGTAAGGGATCCCCCCTTTT

TGTTTTCGGGCCAATGGGGGTTTCGGAACCGGAATGTTGTTTCGGCTTCGCCAGGGGTGG

TTCGGCTTTTTTTTTTTGGGCTTGGGCTTGCCCCCCAAAAAAAAAAA

>316

CGGCGGTGGAGCATGGCTGCTGAACAGATTGGAAGAGCAGAACTGCTTTATCGAGTACAT

AAGCGGTTGAGTCCGTCATCATTGCTGCCATTTTCATCGAGCAACTGGGTGCAGTACAGC

AGTGTCAAAGTCGGTGCAGACGGACAAATTCAGCAATCAGGCGGGTGAAAAAGTCCGCAA

TTGTCGGTCGACTTCGCTTTAAAAAAAAAAA

>317

GAGACTTTTGCAGCTGTAGGCTGACGTGTACTCATAGCAGATCGAACGTTGCAGCCATTA

CGTTCCCAATCGCAGGAACGGTCTTAAACAGTAGTTGTCAAATTCAGCATTACAGAAGCT

GACAAGTTGTGTCGCCAAATAGTCTTCGTGAGACGCTTGCCCGTAAGCAGAGAAGTCGCA

AGATAAAGCGAATTCCGATTAAAAAAAAAAA

>318

TTACTTAAAGCTCAGCTGGTCGAACAGATGATAAAGTAAGGTCAGGGCGGAAAGGATTGA

AAGCATCCTTAAGCTTTTGGACATAGAAAACTCAGGAGGCGCGCTCGCACGCAATTAAGT

GGGAGGACAGACGGATCTCGCAGCTTCTTGAAACTAACTGGACTAAAGACACATGTTATA

TATATAACGCCTTTTGTTTGAAAAAAAAAAA

>319

GGTTGAAGCTTTGCAGAGGACAGTACCTGACACAATTTGACCAGAGTCAATCGGTCAGCA

GACTCTTGAACCTATGTGTTTTAATGAGCCGATGAGCTGCTCATCAGTCTCGCAAACTTC

AGATGTAATCCTGCACCTTTCTTTACATTGAGCCAGTTGAAGGACCTCAGGCTGAGGTTG

CTGAGGCACTTGTTCCCCTGAAAAAAAAAAA

>320

GGAAGCTTAGTGTGCAGGGTGATTGGTCAGCTTGGCTTATTTTAGGAAACCAATTGTTAG

AGCGACGTGGGTCAATATCTGTTTAATTAGCAGCGCCACATCCGGGCATGTCCAGACAAT

TTTTGGCGCACTGCACAAACCTCTCCTTAGGTACTCTTGGGCGCTGGAGTTGCTCGCAGA

TCAGTCCTGACCTTGCATTCAAAAAAAAAAA

>321

TAGCCTTAATTCAGTAATTGCTGGATTTGTTTAACGTCAAAATAAAGTAGGACAGGGGGA

CTCCCGTTGTTGCTGTAGTGACTGCTATAGGCACGGTGATCAGAAGCGTTTGAGCAGCGA

GGATGTTCGGGAGTCAATGACCGAAGCGCGGACCGCGCAATATGCACCGCGTTTTAATTT

GAATTTAAGTCGGAAGCTGGAAAAAAAAAAA

>322

GGGGGCCTGGGGAGGGGGGTGCCGGGGGGGGGGAGGGGGTTTTTGGGGGGGGGGGGTTTG

GTGGGGGGCCCGGGGGGGGGGTGGGGCCCCCAGGGCTTGCCTTCCCAAATTGAGGGGGGG

GGGGTTGGGGGGCCCGGGGGGGGGTTTTGGGGGGTTTTGCCCTTTTTTTTTCCCCCCGGG

GGCGGTTTCCCTTCCCGGTTAAAAAAAAAAA

>323

AGCGGAAAAGGGACGACATGCTGCCGCAATTCACCTCCAAGATACCGTCTTCATATCTGA

GTAGTAAAGAAAGAGTTTTGTGAATCACAGCGGAATGTCTCGATGTAAGGTTTCATTTTG

AGTCCTTGAAAGAAACATCTGGACACATTGTACAAAAGTAGATGTTATGGTTGTGTATAC

AGGATGTTCATGTCGAGCTTAAAAAAAAAAA

>324

AGCCGAAATGGAGACTGGGTAATTTGCTGCAGAGTAGGCTAGGGATGAGGGAAGGAGGCG

ACAGGATGATTGGATTGCTCGAACAAGAGTTTGTCGGGCTCGATGCGGCGTTGAGACACT

CTAGAATTGCAGTAACCTAGGTGTCTGATGCCGTGAAAGGCTCTTGGAAAATTCTTTGTC

TGCTTTTAGATGAGGTGACGAAAAAAAAAAA

>325

GATGCCCACGTGGCCGCAGATCCACGGTGCCCGTCGTTAGGAGTTATCATAGATTCAATT

ATAAGCCAGCGTGCTTACAGAAACACGACAGGTCTGCGTACGAGGTGTAGCAAGCACAGG

CCCAAGGCGGCTCTGACCCTGCAGGTTTATATCACCACGTAATGACTTGATTGTCCCAAA

TATCAAGTTTTTTGGTTTCGAAAAAAAAAAA

>326

TACCCCATCTGTCTTCAGCGACAGGCTCAGGATAAACGGGAGCCTCGCGAGAGCGGCGCT

GAGGGAGTTGGAAGAGCAGGGTCACATCCGCCCCATCAGCAAGCACGCCACCCAGTTCAT

TTACACACGGGCAACCAACACCTAAGAACAAGTTCGGAAGACCTGTGTACAAATATTGTT

TCTCCAAAGCTTCTTGCTTCAAAAAAAAAAA

>327

ATAATGGTTTTTTAGGGTTGCCAAGAAGCAGAAAGGGTGGGTGGAATCCATGTTTCTTTG

TGGGGGTTCATCAATTGTACATAAATTAAGACAAGCTAGCTTCCCCTCCGTGGGCAGGGA

CACTTTTGGGCAGTTGTTTGATTTTCAGGCAGTGCCAAGATTTGATATGCAATCCAGTTT

AATTAATTTCAGTAACCCGGAAAAAAAAAAA

>328

TTTGATGAACACGTGGTTCAATCAGGTTGCGAGCTTGAAGAGGCGGTCTACTAATGATTG

GATTAATGTGCCACTGCCGAAGCGACGTCTGAATCTGCTCTTGGAAGGGGTCTGCTAATT

GCGGTGATTTGACTCTATACTTTAGTATCATATGTCATTTTGAGCTTGTACAAACGGAAC

GAGTTGTACTATAGCTTAACAAAAAAAAAAA

>329

CCCCCCGTGAATTGCGGAAAGAAGAAGGGTGGGCCTCCCCCCCCCCTGGGGCCCCAGAAG

AAGATCAAGTTGTTTTTTTTCCGGGAAGGCGCAAAGCCTCCTTGGGGGGTCGTTCCACCC

CCCCGTTTTGTAATTTCCTGCTTCCGTTGGCCGGGTCAATGGGAAGTTCCTTTTTTTTTT

TTCGGTCCTTTTTTTCGGGGAAAAAAAAAAA

>330

CCGGTTTGCCCCTCGTTTCCCCCCCCTTTTTTGGGGCCCCTTTGGAATTCCCCACATTTG

CCTTCCTCCCGTTGGGGGTTTTTCCCTCTGTTCGCCCCCCCGGCCGCCCCGAATTCCCTT

TGTATTTTTTTCCCGCCCTTCTTTTTCTGGGGGGTTGGGGGGAAAAAAAAAAA

>331

GAAAATCAATTCGTTCGCTTTTGTGTGCTTCGGCAACAAAGGAGCGGCCGGTAAACTGAG

TGAATGTGAACTAGCAAGTTTTGCTTGAAAGATTTTGAGAGGTGACATCGAAAATGGATA

CTTGGGTCTCCCCATTGGCATGCGAACATGTGTACTATTCTGTTTCAGCCTTGATTTTAT

TTTGGTTTTGAAATGCCCCTAAAAAAAAAAA

>332

AGCGCCATCTCACGTCTTGGTAAAAAGGTTGCAAATGAGGGAGGAAAGCTTTGACCGGGC

TGTTCAAGCTTATGCCTGAAATCTGGACTGACAGTAGGTCTGGAGCGGAAGGTCGCACTC

TTCAATTGCACGTCATATCTGTGTGTTGAGACTAGACAGAGTGTTTCAAACTTGCTTAAA

CGATGCACCATGAGACAAAGAAAAAAAAAAA

>333

ACCAAAAGGCACACTCAACAATACACACGCTCGCACTACGGTAGCTTTTGCCATCCAGGA

AACCACCATATTCAGGCACCTCTTGAAAGGTCTGCACGTTCATTGTTCTCCTTATTGTTG

CAGGCGCTACCTTTGCTCAGACTTGATCTCACGTCCGATGTTTTCTGTGCAAGAAAGTAT

GTATGCCACAAGTTCTTCTTAAAAAAAAAAA

>334

GAAGGCCAAGACCTTCGGCAGGGTCAAGTCGTCCTAGACGTTTGGGCATGGCTTGGGCGG

AGGGCAGGCAGGTTCCTCAGCTGGCTTGGGCCGCTGCGAGGCGAAGCCGCTTCGGCGCTT

TCCTGGCATGCCCTCTGCTGCTCTGCAGACAATCCATGATGGGGGGCCGTACTTTTATGA

ATGGGGATGGTCTTCTGGTGAAAAAAAAAAA

>335

GAGGGTCAGGCAGGAGAGGAGCAGGGACTGTCAGGGCAGCAAAGCCAGCCACAGGGCCTC

AGCTGGCTTGGGCCGCTGCGAGGCGAAGCCGCTTCGGCGCTTTCCTGGCATGCCCTCTGC

TGCTCTGCAGACAATCCATGATGGGGGGCCGTACTTTTATGAATGGGGATGGTCTTCTGG

TGCATGCTTCTCGCTAGCACAAAAAAAAAAA

>336

TAAAATGGGGGAAACTTTGGCAAGGGTTCAAGGTTGTCCGGCCGTAATAAATAAATTGGC

CGGTAACCCTTGTTCAAACCTGCCGCCCGGAGGTTGGTGGAAAGCGGGCCAGTTTTACCT

GGGGGGGTTGGTCCCCCCTTTGGGCGGGGGGTTCAACCTTTTTCGGACCCTTTTCGCAAG

CCCTTTCCCCGTTTTCCCGGAAAAAAAAAAA

>337

ATGTCCATCCAAAGAGAAAAGCAATTTAAAAAGTTGACCCCGATAGCATCCCCTTTTGAC

GTCCGGGACTGAGTAAGGGGCCCAGGGCAGCAGGATCAGGCGTTTGGCGGGTTTTTTTGA

CTTGGAAGACAAGTTGTTTGGATGGAAGATGTCGTCACTTTGAGAAGTTGATTTTTTGGA

ATTCTCAAGATTGTCACCCCAAAAAAAAAAA

>338

CGGTGTCAACGCCAAGGTGTTGGGATCCCACGGGATTCTTGCTAACCGGGCCGCTGGCCA

GGCTTTCCTCTCCGATGACATCACTGATATCAAGGTCCCCGCATAAGGACAGACTACCTG

TGCAGTGCAGCTGACCGCCTGGACTTCGTGACACAAACGATGTTCGGGAGTTGTGGATTG

AGTTTGCATGGTCCACGCTTAAAAAAAAAAA

>339

CAGTCAAGCGCTCATTGGAGATAATCGAGCAAATGGGGCTTAGCAAGAAGCGGAAAACGT

AGTCGACCATGTTCTTTCCTGTGCTGTTCGCTTTGGCTTGTGGTAGTGGCAGTGTGTGGC

TTTGAGGGGCCTTCAAGTAGCGAGTGAACATGGGCCTTGGAACTCCAGCTCAAGCAATAA

AGGAGAGAGTGCTTGCCTTCAAAAAAAAAAA

>340

CAAGCCTACATTGATACGGGCAAGCCAGCCTCGAATGCAGACCAGCAAGTAGGTCGGTGA

CATGACATTTGAGTTTTTCGGGCAAGCTGGGGCTTCATCTTCCCTGTCAGGATGACTGCG

GCGCTTGTGCTATTGGTCCGGGCACCAGATACCAATGGTTGTGTATATAGAATTCAACAT

AAGCAGCTACGGTTCTTGAGAAAAAAAAAAA

>341

ACCTAAAAGATTACCCAATCGAGAGATATGTGCGAGACTTGCGGGTGCACACAATTCTGG

AGGGTACGAACGAGATCATGAGGGTAATAATCTCAAGACAGCTTATGCAACATTGAAATT

CTGTACAGTGCGTTTCGATCGCGCTCTGCTTTGGTCTAGGTACCTACAGTGAGAGCCGTA

GGTTGTAAAATAGATTGGCCAAAAAAAAAAA

>342

GGAAGATGGTTTCCATTCTTCGGGGGGGGGCCCAAATAGGGGGGGGGGGCTCCTTTCCGG

CCAAGTTTGACAAATTTGGTCCCCCGGGTGGGGTTTTTTTTGGGTTTTGGGTAAAGTGTG

GGGCCGGTTTCCACCCCCTTGCTTCCAAGGGGTTCAGGGGTTCTTTTTTTTTTGGGGGCT

GGAAAGGGGGGATTCCCCCCAAAAAAAAAAA

>343

CGGTACAACATCGGTGTGGGAGAGCTCGAGGCCTGGACTGCCAGACTCCTCCCCTCAAGA

CAGTTTGGCTACATCGTCCTGACCACCTCTGCTGGCATCATGGATCACGACGAAGCCAGG

AGAAAGAATATGGGAGGAAAGGTGCTGGGCTTCTTCTACTGATCTGTACTCTCGAACTGT

TCAATATGTCACTTTTGCACAAAAAAAAAAA

>344

AACGCCAAGAAAGAAAAAGAACTTGCAGAAAAGCAGGAGAAGGGTTTTAAAGAGATCTAC

GAGGGGTATGAGATAGTCCGGCGAACAAATCATTCCCAAGGGGTTGAGCAACTAAAGAGA

GTGCTGTCGAGGAGATAACAAGCTTACTGGACACAATGATTGCTCGCCTTACATTTTCCA

ATGATGGCGTTCGGCCACGCAAAAAAAAAAA

>345

TTGAATATGTAGTCCCTGTAAAGTAGTTCACCCGGTATATCCTTAGTGTGTCTCCCCATA

GTGCCTTCACAAGTTGGGCCCGTTTAATCCATTTTGAGATTGAACTTAAGGCTGTCAAGT

CGGTTGTTAGAGCCCATGGTCAAGTGGAGATCTTGTTGCAAGTTGACCCCCTTCGTTTCT

GACTGGGTCGATTGTTGGGCAAAAAAAAAAA

>346

ATCTGTTAGACGAGTTCGTTGACTGGTTGAAGGGGACAGGGCACGTGCAAGCGACTACGA

CATAGGACAGGAGTACGGCAACTCCTGGGCTTGGGTACATGCGTTTCTGGACATAGCGCT

TCGCCTCTGAGAATGTTGAATGTTCCTAGATCTGCGCGGATCTAGGATGGTAAGAGTATG

CTTTAAAGTCTAGAAGCTCTAAAAAAAAAAA

>347

GTAATGACAGACACAAGGATTGGGTTGCCGGCCTGCAAGCAATTCCAAACAGTAGAAAAG

AGATTCTTGCGGATTCTATTGACTTTTGCATTGACAAATTTGAATAAATTGCGTGGAGTG

ACGTGCTAGCTGAAGTATGCAGTGACAATTCTCGCAAGCCTGGCCCCAATTTCAGTATTA

AAGCAAGTAAGCTCGATAATAAAAAAAAAAA

>348

TGGGAAGCTGAAGGTGGTTAAGGTAGACGTGGAGGCAAACCCCAAGACCACTGAGAAGTA

TAAAGTATATGGCCTCCCAACCGTCCTGATTTTTGAGTCCGGAAAGCTGTTAACTCACAA

TGAGGGGGCGATCACCAAAGCTAAGTTGGACAAACTCCTGAAGGAGAAACTTCCTGTGCT

TGTATAAGATATCCACAACCAAAAAAAAAAA

>349

GACTCGATCTTCGTGGCCGGTGGCATTCATGCGGCAGGTTTAGGAGTCGAAGCTTTTGGA

GAAAAACCAGAGCCTTCGAGACTCATTGCGTTGATTAGAGAAAAAGGTGCACGAACACCA

AAGTTTTGTCTGCCCTTGTTCAATTGGTGAACAAGGATGCATCCGGCCATTGATAGGTAA

AAGACGTGTTGCTAACTTGCAAAAAAAAAAA

>350

GTCTACCAGGCATTCGCTTCATCCACTGCCGGTTGAGCGCAAAATTTATTCGCAATACTT

TTGAAGTAAAAAAAAAAA

>351

CCAGGTCAACAACGCCTGGGCCTACGCCACCGCCTTCACCCCCGGCCAGTAATCTGTCGA

TGCTAGCCTACGGAAGTTCTGTGTCTGCGTCAAAACTGTACAGAGTCGAGTTAAGACCGT

CCGAGACTGATTCATTGATTTTTAGCAACACATGTTGCCACCCTTGCTTTTGTTGGTGAA

ATAAAGTGGATCGGATCGCTAAAAAAAAAAA

>352

GTATGTTTTGTATGAGCATGGATCCGTTTCGACTGTTCCTTACACAGATTGAGGACCTGT

GACTGCGAAGGTACTACTTGAGATAACTTGAATAAGTTTGAGATAGCAACAGCTTCCTTA

AAGAAGGGAGTTTGTTGAGATAGTTTGATCGAGTTTTTCCATTTGTTGAACGCTTTGAAT

CTTGTAATGGTGAATTTAAGAAAAAAAAAAA

>353

ATCCTCAGCCTGGCGTCTGGAGAGAATCCATGTAAGTTGCTAGGCAGCTATTGGTCTTCA

AGAAAACTTCAAAGCTGTAGCAATCGAGAGTATGGAGAATGAATTGATCGTTCCAGACAA

GCCTAGCTAGTAATAATGTGTTCCAAACGTTCAATCTGGTCTCAGGTTTCCGAAGCCGCC

ATTAGGCGTCTGTATTTACCAAAAAAAAAAA

>354

TGGGGGCCCCCGTCCCCCCCTTTGGGTTATTGGTTGGGGGGTTTGTTGCCCCCCGCTTTT

CCAGGGCGGCCCCTGAAATTCGCCTGGCGTTTGTTCCCCCCCTTTTTTTTTGGTGGGGTT

TAGGGCCCGTTTTTTTTTTTTATTGGCCGTCCCGTCCATTGGCCGTCCTTTTGGCCGCCC

CGGGTCCCGGTTTTGGTTGGAAAAAAAAAAA

>355

CTAAACGATGCCAAGATGGCAAACTGCGAGAAAAAGTTGGCAGCGACTGATCCGGAGCTT

TTGAATGTCAATCCATTCGAGAAGACATTAGCAGGCAGAGCACGTCAGCAGCAGGCTTTG

TTGCAGTGGAACTCTTAAGATTGATAAGGCACATTGACACTCTCAGCTTTCCTATCATGC

ATAACTTGCACCTTGTATCTAAAAAAAAAAA

>356

CGTGACGACATTGACTGAGGCGGCCGGAGGTGCACCCATGTTCACGGTCGACCTCAGAGG

TAAATCAGGCACAAGCGCAAGTGCACAGGTAGAGACTCAGGATTTTGAGGAGGACGGCGA

GGACGAATCGAGCAATAAAGATGAGTGGCAACTTCTGTAACATACTCGAAATCCTGTGCC

ACCACTGTGGTCTCGATAAGAAAAAAAAAAA

>357

GGCATTGGATACGTTGGGGCAGATCCTGCTGATGGAGTGCTGGAAAACTGCTAGCTCATA

GATGTGTATAGCATGCGACTTCAAGTGCAGGACCGACTACTGCCCAAGATTGTGGTCAGG

GCTGTTCGATGGGGAGTTTTGAATCATGGGAATCAGTCTGGATCCTCGTAACCGCCTTGA

CGAATGTCATACTGAAGGATAAAAAAAAAAA

>358

TCGACATGAGACGTTTTTCTCGGTTCTTGAACTCGTTTGGGGAGGGGGTCATTGCTTGGG

AAGGGGATTCTTTTGGCGTTGTTGAGGCTTGAGTCACCTGCGGGCTGGCGGTCTCGTCAA

AGACAGTAGAGCAATAATGTGGTTCGCCGAAGGGTGTACAATATAAGTCGACCTCAGATT

GAGGGAGGTCTTTTTTGGTGAAAAAAAAAAA

>359

CTGGGGAAAGTCAACGGCCGGGGTGGGACTTGGCTAGCGTGTTCGCGGGGTTGGGAAAAG

GCCAGCCGGGGGGTAAATACTTTGTCGTTCAACGGGTAGGGCAACGGTTGGTTTGTCCCT

AGATTTTCGCTTGGAACTGTTTCAAATGCCAGCAATGAAACTCGTTTTTTTGAAAAAGTC

CGCCAAGGATCGGGTGTTGGAAAAAAAAAAA

>360

TTGCTGGGGGGGGGGGCCGCCTTTTTGGGGGGGAAGTCCCTTTTCAAGTAGCCATAGCCT

AGAAAGGTTTTTTGCCCCCGGTTCTTGAACAGAGGGGGGTTTGCCCCATCTTTTTTTTTG

TGGGGGGGGGGTTGGTTTTTTGATTTTTTTTTGGGGGGGGGGGGGGTAATACATTTTTCG

AATTGGGAAGGGGTGCCCGGAAAAAAAAAAA

>361

TGTACTTTAGCTCAGGAATCGGTGTACACCATACAATAGCTCGATCAGATACGTATTCTA

GTATATGCGCATATTCAGCACAAGTACTATAGCCTGCCATGTCGGACGTCGATCGACTAG

CGTAGCCTCGTGGCTTGGACTACCTTCACATGGCGCTGCGGCCCTTACATATCAGCCAGA

CTTAAAAAAAAGCTTGCACGAAAAAAAAAAA

>362

AACCTGGAATTTTTTAGGGGGGTCAGGTAGGCCCACTGAAGTCAGCGGGCAAGCAGTGTT

CCTAACTTTTACTAACAGGACAAGTCACGGCTACGTTTGTAGCCGTTTCTCTAAAGGACT

GGCATGGAAGCCCAAATTTCAATACAGGGTAGCTATAAATGCTCGTTCGACATTCAATGA

GCCTAACAACTTTAACCGGTAAAAAAAAAAA

>363

GAAAAGCTTTTTTGTTGCTATGAATTGATCATGTTTGTTCCTTGTCTACGTATGCCACGG

CACTCACGTTCATTCAAGCTTATAAGCAGGTATTGATTCGTAGAAGTACCAAGCAACCAA

GGCGAGTGGCATTGTCAGTTCCAGATGGGACGCTTTTGGCCGCGCATTGTCGAACCATAG

TCCAATGTTTTGGCTCACACAAAAAAAAAAA

>364

CAATTGGTCCCTGATATCAAGAGCCGAAGCAGTGTTTTGCAAGCTTGATCCCTTTTCCTT

TACAAACCAGGACGGTCAGTCCTATGAAAGTCTTCTGATAGTGCTGCTGAGGTCTACTGC

CGTAGCTTGGCCTGGAGCCGCAAGCTGAGTATGGTTTCTCCATCTACTGCTGGGAATGAG

CATTCAACAAACTTGCATTCAAAAAAAAAAA

>365

AGCGAGTATACTACACGGTAGGAGGACATTCCTCCCTACAGACAAAGACAAAAAGTTGAT

ACGGTATACTGTAAGGTCGTCGAGAGTTGTTCGAATGGGTTTTGTTGAGCCAGAAGCCTC

GAAAGTCAAATGTTGCTTATGGAGAGTCGATTTACAGACGAAACTATATACCAGCAATAT

AGATGAACCTACTCCGTCCTAAAAAAAAAAA

>366

ACATGCTGCTTCGTTGTTAGTAATTGCTCGGTAACTAGGATAGAAACACTTTTTTCAGAA

ACGCTGAAATTGCTTGTTTTTTGCGCCATGGCCCGGCCCAGGGGCCTGAGCGTGAGTAAA

ATAGTGACCATTGAACGGAATCCCAAACTGTACGAGGAGCTTTGAGATTTTATATATACC

AGAAAAGGCCTTTTTTGATTAAAAAAAAAAA

>367

ATGGGTCACTTTACGTGTGCGCAGCTGATAGGCTGAGTGAATCTTAAGGTACTAGTGTAT

TGTAGCCTCAGCACATGGCACTCAGAGCCTTGTGTTAACAACGTATTGTGACTCAGTGAG

CACTGAGCAAGACGCAGCAACCAGCAATTAAGATTCAAGTTTGATGCATCAAAATCTTAA

ACAATCTTTATTTTCTAGTTAAAAAAAAAAA

>368

TGGGGTACTTTGGGTCCAAAGTCCTTCCGATTTTGTGGCCCGTCGGGTCAAACCCAGTGT

TGCCGATTTTCCGGGCCGCGGGGTGAACCGTTTTTGGCGGCAAGAAGACTTGGTTGGAAA

GTTTCCGTGTGCCTTGTAAAGAAGGGCCGGATAAGTTGCCTTGGGTTTGTCCCTGAAGAA

TTGGCCTTGCCTTGCCCTGGAAAAAAAAAAA

>369

GGCATGCCGGCCTGTTGGAGCATCTTATTGCGGTGCCTCGCAAGAGGCCCGGGCCCACGT

AAGCTTTGGACTGCGGACAGGCTCGGTACAAGGCTAGCCTGCCTTTATATGGCTCGATTG

TGTACATACATGCAGAGGCCAAAATATCTTGGAACATTGATACTCGGACGTTATGTTTAA

TCAAGCCGCCGATCCTATGCAAAAAAAAAAA

>370

GCGCTGCTCAAAATGAATGAGGGGGCCTGCTCATCCACTAAGAGACTCGATATTGTATCC

TACATAATTTGCAGTTTGTTGTGCATCCATGCAGACCGGATTCCGTTTTGCAACATTATA

GGCCTTTTGATACTGGTCAAAACATACTGTGAACGTTTCTGCCAAGCGTAGGCGGCCTGC

TGGCCCACCCCGGTGACCAGAAAAAAAAAAA

>371

TGATTTGTAGGGGGTTTTTGCTGGCTCCCCAAGGGGCTGTGGGGAGCCAAGTTTGAAGTC

CGGGGTAAAGGATAACTTGCCTGACTTGTGCCCTTGGGGTTGGAAACTTCATACCTTGTC

CCCGTACTTGTTTTTTAGTTTTGTTCCCCTTGGGCCTTTTCCCTGAACATTCCGTTGCAA

TGGCCCGGGTTTTTCCTTGGAAAAAAAAAAA

>372

CCGGAAAGTTCCTGATGAAGAACCCAATGGCTTAGGCCAAGATGGATGTTTGGTCTAGTC

TGGTCTACTTCGTTTACAACCGAGTTGGAATTGCTATCGAACCAGAACATCTCTGGGCTA

TACTGGATTCAAACACTGCCCGTGGACACCTGCACGCTGATCTTCTCGCCCTGAGATTTG

TAAACAAACCTCGTCACTTCAAAAAAAAAAA

>373

AAGAGAGCTTGGGAGCCAGCCGACGAGATTGGATGAGTGACAGGGCGCCGAGCACCATTA

GGGGAGGCACCCCAGACCAGGTAGTTTCAGAGGAGCACCAAGAAAGGCTACCTTCGGAGC

TGGATAGGGTCCAGGTGAGAGAGGGATACCATTTTGGGAGTCCCTTGTTGCGCTGTAATA

GCATGTCCATGTTGGGTTGCAAAAAAAAAAA

>374

CCAAATCAAGATTATCGTCGGACATTCAGACGTTGTTAAGCTTAGCGGACTGCTTTAACA

CGTGGCCAAGTTTTTTTTCGGTACTCGCCAGCCTGCTTCTGGGTAAATACTAGGTTCGTG

TCTATTATTGGCGGCGGCAGGCACATCAACCTGTTTCCGGATGCAACTCAATCCCGCAAG

GCAAGCCTCTGAAATCCATCAAAAAAAAAAA

>375

GCTACTCAAGTGCAGCACTTGAAATACGCATGACGTTTGTACACACCATCTTGTTCTGGT

AGTGTTAAGCGACAGTATCTATTGTTAACTGACAGTCCCGTCAATTGACAGTCCTTTCGA

CAGCTCCGGGTCCAGGTTATGCTTGATTCTGAAGCTACGTCTCACCAAAGACTGTGTATC

AATCATGGTTGAACCACGCTAAAAAAAAAAA

>376

TCAGCTCTGAGAGAAACTGAATTCCTTGAATTAGCTATTAAAAACAGTCTTGAGCATACA

GACAGTGTTGTTGGAAGCTAAGACATATTTGAATCGATTCAGTATTGAAAGACAGACGTC

TATCAATCTGCTTATTCATTGGTGAACAATTTGGTCAATTGACGACAACCGTAAGTAGAA

ACGCACAATGTTGACACCTTAAAAAAAAAAA

>377

CTTATCGGTTGTATTTATACAAGCCTGGCCAAATCATTGAATTGTATCTGTAGATAGGGA

AACAGATAAGTACCGCACGGAATTATCAATTAGGCAGGACAGACGTCAAGAACATGATTG

GCTTTAACTGGCCACATTATGACTTCTTGACCGTCATATACAATATCCCTGTTCGCAAGC

GCAAACCTGATTTTGCTGGCAAAAAAAAAAA

>378

GGGCCATTTTTTTGGGCGGGGTCTTTTTTTTTGGGCGCCGTTTTTCCAAGGGGGAAGGGG

CGGCCCCTCCAAGGGGTGTGCCGGATGGGGCTTTGGGTTTTCCTTTTTTTTTTTTTGGGT

GGGGAGCCTCCAAGAAGGTTGGGCAAGGGGTTTTGCCCCAAGGGGCGCCCCCCCCCGCTG

GAAATGGGGGGGGTTTTTCCAAAAAAAAAAA

>379

CAGTGTAAATATGTGGGGTAAGGGTTATGTAACAACATGTCGTACCGAAGCTTGCAACAG

CATATGGTAGACAGGTTTCTGTATTGCTCGGTTATCAGGGTTAGCTAGGGAGAAACAGTA

GGATGTAAAGTACATGTGTACAAATTGTAACTCACCAGACTTCTTACGGCAATCTTAATT

CAATACTTCAGCCTTGCGGCAAAAAAAAAAA

>380

GGCAGGCCGGTAAATCATTGGGCTTGGTAGGCCATAAGGTTACTCAATTGTTTTTTGGTT

TTTTTCGGTTTCGGGTCTTCTTTCGGGGTTTTTGTGTTGGGTTAAGTCAGGGGTTTGTTT

TTTGGGTGGGTCCTTGGTTTTTTTTCGGTTCCCCTCCCCGGGGTTGGTTTGTTTAAGTTG

GGGGGTCCTTCCGGTCCTTCAAAAAAAAAAA

>381

CACCCTTCTAGTCAGAGTGGCGGCAGCGCTTTCGAAAGGGCACCTTTGACTTCGGCTGAT

GTTGAGGGAGTTCCGGCCAGGAAACCGGCTTCTTAAGTGTCATTGACACAACCCCTGGCA

TCCTTGCAGTTGCCGGGCGAGCTTGCTTTTGATTCGTTCGAATGTAATTGCAGTGAGAAG

GTGGCGTTCTATATACATTTAAAAAAAAAAA

>382

GTACGCCCAGATCACGAACGACCCAGAGAATGATGGCTGCATTAATGGGGTCTTGTTAGT

ATAAACAGTGTACATATGTCGTTCTGCTCAACCAACGTTCTGTCAAGACTAATCTCGGGA

GTCAAGCCGACACAAAACGAGGCAATGCGCGCGAGGCCTGCAACAATCGATGTTTCCTTC

AACAAGCAATTGCATATGCTAAAAAAAAAAA

>383

GCCCAGGCCCCAAGATCGAGGGGGTTGATTAGATACGCTGTTAGACGCAGACTGTAGCGT

TTGGGTAGAGTCGTGAGTATGGACTGATTGCAAAACGGAATAAGCGCGCCCAGTGACAGC

TTTCGCCCTGTACGGATAATGCCTCGTGGTATTTTTGCAATCCCCAATCCCAAGGTGGGG

TGACCACTTTTTCGCTTTTTAAAAAAAAAAA

>384

CCTGTCCCTGCGTCTCCACGGTTGGCTTTAAAAGACCCCCTATAGGCCGGATTGGGCCCC

TCCTCCCAGCCCCCCCCCCCCCAGGGCCGCCCCCCGAATTCCCCCCCCCCCCTGGCCTTT

GCTTCCTGGGGGGTTGGGCCCGAAAACAATTTTTTTTAACCAAAAAAAAAAA

>385

GAGGTGGATATCGGCTTGGATATATTTGTTGTGGTTTCGCCACGTATGTACGTTCAGGTT

TAGTTGTATAAGCAATCCCTGAAGTACGTCATGCAGCAGTACCGGTCTTCGAGACATGTG

CGAAACGATTGAGTTCAGTTTGAGCTAGGAACCGTGTCGCAAGTCTGTGTAGAGGAAGTT

ATATAGCGCTTAGAACTCCCAAAAAAAAAAA

>386

AGACAACCTGCTCGCCTCAACTTCAGCAAAGAACAATTTGGGGGGGTTTTCTAGGCCTTA

TTTTGCCCCTTTTGCTGTAGAGGGTATGCTGGGGACCAACTTTAGCATCGCAGAGCAAGC

GCCGATACGAATTTGAAAAGGGTTAAGTCATCTTTTTAAGAAGCTTCGCCGCCAGTTCGA

AATTCCAAATTCCCAGCCGGAAAAAAAAAAA

>387

TTCCTGAGGGGATTTTACGTTGTTTGTATGAGTAAATCCTAGGATGATCTGGGAGTTTGA

TGTATAATAGCTTGGCACGCAAATTATCCGCGAATCTTCCAAGTTTTGAAGGAGTTGCAA

CAAAGGTCAAGTTGCCAACAGCAAAGGATCGATCATAACCAATCTTCTGGCACATATTCA

GGCTATCAGTGCACGTTGTGAAAAAAAAAAA

>388

TGATAGCAAAAGCGGGTTAGATTTGGGTGTCCTGGGGGGGTTTAAAAACCATCCCGTCAA

AATAGGTGTTAAGGGGTAGATCTTTGCAAATAAAGTTTTAGGGAGTTCCCTCGCTTAGGA

GTCGGAAAAACCAAGTCCGGGGGGGGTGGTCCAAGCTCCAATGGTGTTTTTTTCTGTACC

GGGGGGATGGGCCGGGCCTCAAAAAAAAAAA

>389

CGAATTGAAGCACTCAGGCAGTTTACATATCAACATGATCAACACAAGGGTTAATAATAT

ACACGCCTAGCTAGTACTTTGGAGTTTGAGTGCCGAAGCTAAGTTGGTTCTACGGGTGGG

NCGGCCACCGCACTGAACTGTAGTATAACAAAAACAATGGCTGCCACCGGTCAGAAGCTT

GACTGAAAGCATTGATCTTTAAAAAAAAAAA

>390

ACTACCTTGGCGAGTTCTCCATCTCATACAAGCCTGTGAAGCACGGGCGGCCCGGTATTG

GTGCCACCCACTCCTCCCGTTTCATTCCTCTCAAGTAGGCCCTTCAAAGGATAGGCCAGA

TGTGGAGGACTTTGCAACTGGTGGCAGTCAGCAGTCAGAACTGGCCATGATTCCTATGTG

CTTTTACTTTCCCTCAAATCAAAAAAAAAAA

>391

AAGGTCAAGATCCTCAAGTCGCCCAAGTTCGACCTGGGCAAGTTCATGGAGGTTCACGGA

GACTACTCGGAGGACCCGGGGGGGAAGATCGCCCGCCCTTTCGGGGACGAGGCTGCTGCG

GAGGGGGAGGCCGCCGACCCTCAAGAAGTCGTCGGTGCCTAAGTGCAACCAACCGCTGTT

TGTAAAAAGTTTTGGCCCTCAAAAAAAAAAA

>392

GAGTTTCGCAATCGCTGGGCGAGGAGCAAAAGATCGTAATAGCGCACTTGAGACGCTGGA

AAAAGGACGCAAAGTTCTTTCGGAAGGCGAGCAAGTACTTTGACGTCCGGCTCGTCTATT

CGCACCCTCCGGTTGGCGAAGCAAGGAAAGGAGTGGAGGTGTTTGAGATGGTTGCAAAAA

GATAACCACAGCTTCAATTGAAAAAAAAAAA

>393

GTCCTGTGAGCTAAAATAGTATTTTGGTCTGAAGTTGATCAAAGCCGGGGGGCTCTCATA

CGGTGTCCTTCGAGTGATCTGTGTAGTCATTCTCTGAGGATTGTGACTCATGTATGCTTT

CAATGGCGGTGCTGGGCTTTCTGCCTGCTTTGAACGACTGGAAGGGCCTCGGTCGATGCC

GCAGTCCAGCGTGCGTTTTTAAAAAAAAAAA

>394

ATGTGGAGATATCGGTAATGGAGCAATGGCAGAACCGTCAAAGATCGTCCAAGTGACGCG

ATCGGTGACGTGTGCTTGCGTCATTTCCTCCTTAGCAGGTCTCGATGTCGCAGCCCGTAA

GCTTGCAGAATTCACGTGACCATACTACGGATATCAAGTTTCCGACGCCTATGAGATGGG

CTTATAATGAACCTATTCCCAAAAAAAAAAA

>395

ATGACAAAGTACAACATTGATCTAGCTCTCCTCCTGTAGATCTTAGAGGCGTCATTTCCA

TTCTTTAAGCTTCCGACATATCTCGAAGCACTCTTGATTTTTGGAGACTCGGCCAGGGTT

TAGCTAGATTGCACTTGACCGGATGGTTGTCAACTCAATCATTGGACTCACAATCAAAAG

CGGCGCTTATCCAAGTTACTAAAAAAAAAAA

>396

ATATCAAATGCTTTAGCTTTAGTTTAAACTTCCTACATAGGGACAAACGGGTTGCTTATT

TTTGGGCCGTTGCCACTTGCTCTGTGGCACGCCAACATTTTGCAGCACTTTGAATAGCTG

TAGCTATACGTACAGACAGCGTATGTGAAAATCGAACAATCAAATCGATTGTTTGACAAA

AGAGTACGTTTTTGAACCACAAAAAAAAAAA

>397

GACCAGGCTTTGAAACTGCGGAGAAGTTTCTTGAATTCCATGCCCTTAGGTTGACGTTGT

ACTGGAGGCCAGCACCCTTTTGCAGCTGCATGCTAGACAGTTACTTGAGAGGTTTTTCAA

AGCTTGCACAAAAGTTTAATCAAGACTCCCATGCAAAAGCTAGCTGCGATTGGTACTAAC

CGTCATTCCAATTGCTGGGCAAAAAAAAAAA

>398

AGCCAAACGACGATATAAACCACAGTGACATCCTCTGTTCCGCGTGGGGGAGTGCCGTTC

GCGCATCTTTGCTTGTTTTCTGACAGACTGTAGTGTTGTAAGTATCAGGCAATGCGGGCC

TGTTAGAACATCCTAATGTCATATAGTACACTGAGTTAGTTTGGAGATTGCAGAGATCAC

TTTCTGTGATCGCGCACTAGAAAAAAAAAAA

>399

TGCGGAAGTACCCGTGTTGACGGGACCGAATCCTAAGATGGAACGGGCAAAGAAGGACGG

GAAGAAGGGGAAGGGAAGGAGGTAAACGTGTTGGGAGACATCGACGGTTGGTGCAGAAGT

TCTTTTGTCGCAAAGGACTTGCGCCGGAGCTACTTTTTGTTCTCTTGGGACTGGATGAAA

CAAAGTGTACATCTCAATTGAAAAAAAAAAA

>400

GCATTGAACATCGGTCTTACGCGTTGATATCGACACATTGCACCTGTACATCACATTCAC

GCAAGCCAACAATCATAAGGAGTCATGAAAGCACTCAGTGTTAAGGGCAGTGACCACTGA

TGTGTGGAGAAAGATTGTGACCTGGTCCCGGACAAAGTCCTGGTTGTCTCATCTCTTGCN

TAGCGTAAAGTTGAACAGTGAAAAAAAAAAA

>401

ATAAGGTTCTGCTTTTCCTTAAGCCTTTCCTAAGCCCGTACAGAATGCTACTTACGAGGG

TAAGTATACTTGAATTAGCAACTCACCTTGGACACGTGTTCAAACCTAGGCCTACTAACA

TGTTGAACATAAAATCTTTGATTGTATAACACTCGAAACAGCACTAGCTATGACTTAGCT

GTCAGCTGGAGGTTATGAACAAAAAAAAAAA

>402

AGTGTTGGGGAAGAGTTGTCAGGCGCGAGTTTGTGTACGAACAGCTTCGGTTCACATTGA

CCTCGTCCAGTTCATACCTAGCTCCACCTGAAGCATAGCGAGCCGGCTCAAGCAGTCCAC

GGAACGGTGTTTGAGGAACAGCAGATCTCTGGGGTATCTTTCTGGGGATGATCTAATAAC

TTGAAACGGGTTTCAATGTCAAAAAAAAAAA

>403

GGGCCTGAGTCAGTAGCTTGTTTCGTCGGAGTTTCACCGTAGTCTTGCACGTTTCCGTTG

TAGGTACTTCGCCTTTTCTTAGATACGGCTCTTGATGGCTTGGCGGCCAGAAACAAAGAC

TAGTGTCTTCCAACAAGACTTGGGGGCGAGTCAATGGTTGTTTCCGGGAACTGCATGGAA

CCGCTAGTCGGCTGCCTCTGAAAAAAAAAAA

>404

CAGATACAGGCTGGTCCGGAACGGCTTGGAGACCAAGATCGAAAAGTCAAGAAAGCAGAT

CAAGGAGCGCAAGAACAGGTCACGGAAGGTCCGCGGTGTGAAGAAGAACAAGGTGGCCGG

AGACTCCAAGAAGAAGTAGATGGGACACAACACCAATCTGTCTCGTTGAAACGTCTTCAA

TCCATCAATCACCAACCTCCAAAAAAAAAAA

>405

GACCAGGCCCGGTTGCGGACGACTAAATATTTTTGCGGCGTGCCTCTTGTTTTGAGGTCA

CTTGGGTTGAGTGGACTTGTTTTGGTGCTAGGGGCGGCTCGTTAATGTGGCCGACCACGG

TCCGACTCACCGCACAGGTCTTTAGCTCCCATTATACTCGTACATTCTTGTCTGTAATCG

CATGTGAGTCCATTGGCAGCAAAAAAAAAAA

>406

GTTTACCAGGCATTCGCTTCATGGCCTATATTGGACTTCGTAAATAGCGACGTTGATTTG

GGCTGTTTGATCTTTCAAACTCTTTTTGATTGGTCGGTTCGGATCTAAAAAAAAAAA

>407

TTTAGTTTTCAAGCCCGGGACATAGCTTTTTGGGTTGGGGCAGTAATTTTAACCCGCAGG

GATCGTTCCCCTTAGTCGGTGGTTTGGGGCCCCCTGGGTCAAATAATTTGCAAGATGTTG

GGCGGATTCTTCCAGCGACTGAAGTTGTCATTTCAGACGGACAAATGTTCCATGGATAGT

CAAGATGGTTTGCCCCCTTTAAAAAAAAAAA

>408

CTTTGCTTGAAAAGTAGGAACCGGCTCCAGTAAAGAGCCGGTTTCCCTTACTTTTCCCCA

TAGGGTTTTTTTTTACTCTTGTACAAGGCGCGATAACTTGGACTGCGTACGAGCAGCTGG

CATGAAATTGGACAGCCGAACGTCGCTTCTTTTTACGTCAGTTGAAGCCAATATTTTGAC

TTGCTAATTCGGTCGGTTTGAAAAAAAAAAA

>409

GACCCGAAGGACGCTGATACTCTTCAGTCGAGGAGTCTTAAGGGGTCCGAAATGAGAAAG

AATTTGCGGATTCCAGGGCGTAGAGCAGATATCTGGTGTGGGACTTTTGGTAAGGCATAC

TGGGGAACTTGCTTACCAGAGGAACTCAGGCGCCACACTAGACGAGGCTGCTAGATATTA

TCAAAAGGCAGATTCTTCACAAAAAAAAAAA

>410

TTTGTGGACCCTCTGCCACCTCGCCCTGACCAAGGCGGTAACGTAGTAGCTTTTAGTGAT

TCCAGTCCCCATGCATTAAAATTGCAGTTTGCAATCGCAATGATACCATCTGCTATGAGA

GTACTTCGCAATTTCAGTGACAGCTCCACATGCGAACTTGATTAGTACTACCGCCACTGT

GTACTGTCTTGGTCACTGTCAAAAAAAAAAA

>411

TTTTTTGGGGGCCCTTTTGGATTTTTCCATTTTTCCTTCCCCCGGGGGGGGGGTTTTTCT

TTTTTTTGGCCCCCCGGGGGGCGGGGTTTTTTTTTGGGTTTTTTTCCCCCCCTTTTTTTT

TTGGGGGGGGGGGGTTTTTTTTTTTTCCGGGGGGTTTTTTTAATTTCCCCCCCGGGGGAA

ATTTGGGTTTCCTTTTCCCCAAAAAAAAAAA

>412

GTCTACCAGGCATTCGCTTCATGGGGGATAGGGTCAACAAGATTTTTTGACGGTTTTTGG

GCTGGCATGTCCAATATGATGATAGTAGTAGCTGATTCTGATCAAATGAAATCCGAGCAA

GCAGACGCTTTTGACGCTTCAAAAAAAAAAA

>413

GCTTCAGCCCGGTGGAAAAGTGAGGCCGAGCTCGCAATGCTTCAAAACTGCAGAATTGAA

TGTTCTTTTCCGGATTTTAGGGCGATATGGGTATGACCATGAATTAGTTAATGGTCTTAA

GTTTTTGACATGAGATTCTCGAGTAAGTTCCCGCTGTATGCCAGTAAGGAAGATTGACAG

CCTAAGAAGCAGTCTTTTTTAAAAAAAAAAA

>414

CGCTACCTTGAAGACTCCGAGGAAAAGCCGAGGGAAGAGATCGCGAACTGGCAGGGGTAG

CTTGGAAGGAACGGTAAAGATAGCCAAGGACAGTGGTCTGTTTGAAGGTAGACTAGATCA

AGTTGCTTAGTGCAGCGAGTGACGGTCGAACGATGAGACTGTGAAAACCATAAACGTTTG

TAATTTCAATTTGGATTGTGAAAAAAAAAAA

>415

TTAGCACGTAGCGCAGAGTGTGAACTTGACAGTGTACAGGAACATAACCTATTCAGGGAG

ATTGCAAATCTTGATGTGCATTTGAATAATGGATGGCTTGAGTAGTGAGACAGGTAATTA

GACTTCAACAGTTAGTGGTATACTGATTACATTCTAATGAACATCATGTGTTACAGATAA

ATTGAAATTCTTTACCTGGCAAAAAAAAAAA

>416

GTTGTTTGAGCATCTTCTACAGATGTTCTAGGTCTTGTCTAGTCGCGACACCGTTTTAGT

TTAAAAGTGCTCCGGATTAGTTTTCGGCCATCGGGCTGCCATAAAGCTGAAGGGGCAGGG

GGCTTGCTTTAGGTTGTGTGGGCTTGCTTAAGGTGGCACAGGTGCTCTTAATTGTCTTAA

AAGTCATCACTTGTGTTGCCAAAAAAAAAAA

>417

CGGATCCGCAGATTTTGTGCGCAAGACAGTCCTCACAGCATTGGCCTCGGCTTGTAGTGG

ACTGCTCCGTCCATCAAAAAACAGGTCGCAGGTTGGTCGAGGTGTGTATGATGAGTAATA

GCAAGCCGTGGCAAGCTGCTGCTGCTACTACCGACCTTTTGAACGTTAGTCACTGCGTCT

TTAACAACGCAATTGCCTTCAAAAAAAAAAA

>418

CCGTGAGTCTGTGCGGCATTTCGGCATCGCTCCCGGCCAGCCCCACAGCCACACCAAGCC

GTTCGCCCGTGCCAAGGGCAGGAAGTTCGAGAAGGCTCGTGGCCGGAGAAACTCCCGTGG

TTTCAAGGCTTAAGTTTGTGCAACTTGGACGCTGCAGACAAAGTGACCGATTCAAAAGGG

CGTCGGGGCTCTGTGATGTTAAAAAAAAAAA

>419

AAGCGGAACCTGTGGCCTATAAGCGCTCAAGAAAGGTCTAGCGACTGGAGACAGGACTCT

GACCGACAATCATAGTGAACATTGGGCCATGGGGGAGTCGCTTTCGAGATTTCGTATTGT

CACTAAGTAGTTGCAAAATCACCCCTGTCATGTACAGAGAAAGTCAAACCGTCCAGGCAA

GATTCTAAACGAATCAAATGAAAAAAAAAAA

>420

GCGGCGAACGCGCTTAAGTAGAGATCAGATTTTACCATACTGAAATAGACTCAATATTGG

TGAGTGCAACATAATATCGGTTTGGAGGTTCTTGCCGGTAGGTTTTTCAGCCACGCGGGC

GTGTGATCAAGTGCGGAAGAGCAGACCACATATGCTAGGATTGTTAGGATCATTCAGCCT

AACAGATTCAAGTTCTACTTAAAAAAAAAAA

>421

ATGAGTGTGGAATAAGTAGGCTTGGCAACACGTAAGTCAAGCTCCGCTTACAGGTCCGAG

CATTACTAGACCGTCGTTCCGTCGAAAGTTTACCGACACGTCAAAACTGATAAAAGATAC

GATAGGAAACACTCTCAAACAATTAGAGCCTTCCAAGGTTATAAGCGGCTGCATAAACAT

CCGATCACTTTTGTCAACTCAAAAAAAAAAA

>422

GGAATGGGCCCTGCGACCTTGAAGCGCCTTTTGGAGGGTCCGATTTAGAGTTAATAATAT

CGTAGTACACGTCCACAAGCACTAGGTCGGGTCCTGCGACAGCTGACAGCTGTCAATCGC

CCTTTTTCAAAAACGCCTAAGTTCAGCCAGTCCTGGGGCGCCATAGGCTTCAAGGAACAC

AGCTGTTCACCTTTCGATTGAAAAAAAAAAA

>423

GCTTTGTAAGATACATATGATACTTGCAGACATGTGAAAGTTGCGATGTAAGATATATGC

CACTTGTGAATAACGATAAAGCTGCCGCAAGCTGCTGCCCTACCGGTACTTCGGACTTTG

ATTCTAATACGTAAAGACAAAGCCTCAGAAGTGTGACAAGTGTATCCAATTGATATCAAA

GCTACTTGCTTGTTAGTAGCAAAAAAAAAAA

>424

GTCTGCTTTATGTGCATTATCATTTGGTCACCCCATACTGCACTGTCATTCTCTCATACT

ATAAATCCGATGGCAATCTAGATATGACTAGCAGCTGTAGCAATCTACAGCTAGGTCAAC

AAACTATTCTAGGTCGGGTCAGAGCTCAACTTGACAGTTGTGGCCAAGGACATTAATAAG

ATAAGAGTTTCTTTCAAACCAAAAAAAAAAA

>425

GTCTACCAGGCATTCGCTTCATCAGCCTACTCAACAAGCTCGTTTACAGTCCTCTTCAAA

CAACGTTCAATCAATTTTCGCACCTATTTTGGCCTCAAGTCTGGGCTCTTTTAGGCCCGG

GTTGGCTTTACAATTCAGGCAGCAAAAAAAAAAA

>426

GAGTCTAGGTTGTAGTTTAGTCGATGCATACGGAGGGGTGTATTTCAAGTGGAGCACAGA

GCGATTGCATGCAAGTCTTGCCCTCGATCGGTGTGCTATTCAAAGAATTGTGCTTCTGCG

CGTGTGTTTGCAACTGTGATAAACGCAACTCCATCCTTTAAGGCGGGTACATCGGGCCTA

TAGGTGTACGTACCAAATGCAAAAAAAAAAA

>427

ACCCTCCGAGGGCTCAGCCTAGGATTGCTCGATGAAAGCATTGAGGATCATTCGATGACG

AGAAGACAAATTAATCGTTAGTACAGTGACGTACTGTAAATATTGCAGGTCTTTTTTGNC

ATTAGAGCTATCTAGGGCGGGCTCGCGAGATCATTCAGAAGGACATCCTTCGATAAAGCT

AGCTGAAATTATATACCGACAAAAAAAAAAA

>428

CCCCTCGACTAGAATAGCCGACCACGCTAACGTTCATCGTTTCAGCTATGAGTCTTTTTT

CCAGAGCGGTTAATAGACGATTGAAGAGGCATGTTGCGGCATGCGTCAGCATTGTACACG

CATGGGAACGTTGCTTTACTCTAGATTCAGATCCTTTGCGATCGGACTTCTTTAAGTTCT

GATATGTTATTGCAGAAATCAAAAAAAAAAA

>429

CAAGTGCACGCGTGCAGCATATTGTTGCAGAGAGTGTCAAAGAGAGGATTGGAAACAGCC

CAAGCTGGTTTGTGTGGTAGGAGCCGCTGTAGCAATTTCCTTAAGGGAGTTTGCTGAGGA

CTTGGAGGAATTTGAACGAGCGTACGCTGCAAAGACGGTATTTGAATAAGGTTTTGCTTT

ATATAGCATCAAGTGGTTTGAAAAAAAAAAA

>430

GGGACGCTTAGAACTAGCTTGAGAATTTCTACAGAGTTTAGGTCGACGTTAGAAGCGGAA

ATATGCGCGCATTAATCTGACCTGTTGTGGACGTTATTTTTCATTACAATCCGGTTGTCA

GCGTGAGTCATACAGCCAGGGCCATTTCAAGGACCGTACGTCAGATGCCTTTTGTATATA

AGGAAGTAGTGCTGACATCCAAAAAAAAAAA

>431

ACGCGTAGGGAGAAGCGTTGTATGTAACCTTGTAAAGTAATTGAACTCGTGCAAACAGGA

TGATGATTGTGGTGCCGCAACTGGCATCAGAAAAATTGAAAAGGAGCTGAACAGAGATTG

TAGAGTTTTTAACGCGTTCTTCAAAAAGCGGAGAGGAGAGTACGTATTTTCTCATGTAGC

AAACACGTACATTACATTTCAAAAAAAAAAA

>432

TCAAGTTGCCTTTTAGGCTTGCTTGTGCAAGATTCTTCCTAGTACACCGTCAAAGCTTTT

GCAAACAACGAATTGCACCTTTGACGCTTGGGCCGCCTAGAGTCATGATGTGTTTGAACA

TGAAAGAAGCAAAAGCTCTCAAAGGCTGTAGTTGAGTCACTTATTCCGTTTCATTATATA

ATCTAATAATGCACACGCGCAAAAAAAAAAA

>433

GCCTCATCGTGGTACTTTGGGTCCAAAGTCCTTCAGATATCGTGGCCTGTCGGATCAAAC

CAAGTGTTGCAGATTTGCAGGGCAGCGAGCTGAACCGTCATAGACGACAAGAAGACTTGG

TCGGAAAGTATCCGTGTGCATTGTAAAGAAGAGCAGACTAAGTTGCCTTGGCTTAGTCAC

TGAAAAAAAGGCCTTACCTTAAAAAAAAAAA

>434

AGTACAGCTGCCAGTTAAATTGGGCTAGTGGGCTGCCCAAACCAATCAGGACAACACCCA

CACCCTGGCAAGTTTCAAGTTTTGCTCATGATGAGGCCTGAGCTTAGTTGTCCACTGTCC

AGTCATGTGTTGAAAGCTGCCATCCTAGACAATACCGACTAATTCTCTCGCTCATTTTCA

TGGGTCTTTCCGTGACATAGAAAAAAAAAAA

>435

GGTAGAGTATTAGGTTAGGTTTTGACGATCAGAAATACTCTGGACAATACCACGTAACAA

GGTTCCACATGAACAGAGCGATTCTTGATATCGGTGCTAGCTATGCTACTTCGTTTGGAA

AGCCGGTTTGATGTTTGCAGCCTGGCAAATGCCATTGAAGCGAACATTTGAAGGCCGTAG

TCTTGTGAAATTTCGAACCTAAAAAAAAAAA

>436

TCCATAGCTAGGGTTCGATAAGAGGGAGTAATAGATTTAAGCTGAGACTTTGAACTTAGC

CACGATATTAGAATTTGATACATCACACAAGTTGCGTAGCCATTAGCATCACTGGCAGTC

GTTGATAAGTTTGTTAAAATGGGTCCATTTTGTCAAAGATTTAGAATACCTTTCATTAAG

CAAAATGGTTTTCCGCCCGTAAAAAAAAAAA

>437

GGGGGGGGGGGGGTTTTTTTTTCCCCCCCCCCCGGGGGGGGGGGGTTTAGGGGGCCCCCC

CCCCCCCCCCCCGGGGGGGGGGGGGGGGCCCCCCCCCCGGGCCCCCCCCCGGGGGGGGGC

CCCCGGGGGGGGGGGGGGGGTTTTTTTCCCCGCCCCTTTGGGCCCCCCCCCCCCCCGTTT

TTTTCCCTTTTTCCCCCCCCAAAAAAAAAAA

>438

GTTGGCTTTTTGTGAGGGCGTCCAGCTAAGTTGTTTCGTGGAGTGGAGCAAAAAGGATCA

GCAGATTGTCAGCAGCTTACGGGACTTCTGAAAAAGCTGCAGAGCGGTAGGAGGTGTCCG

GTTCACCGGTTGACATTCAAGTTGCAGATCATCACGTTTTGAGATACATTGACTTTGTCA

TGGGTCAATTATATGACTCGAAAAAAAAAAA

>439

TACTGAGTGGGGCTGTCAAGAACCGTATACGACGAAGTCAAGAATGGTTGGGCGACCGCT

GATTGCGCATATAGCATCTAACATGTACAGAACAAAGTGTAAGTATGATGTCCACATGAT

CCTGACGAAATAAAGCAGACGTTTCTGCTCCTCAGTTGGAGATATAAACGGCCCGATCGT

TACGAATATTCTATTTTCGGAAAAAAAAAAA

>440

TAGCACAAAAGAGGAGGACTCGTACAACAGACTTAGCTTGTCCTGGAGCATAGACATAGA

CAAACGAATTTGATACGCATTGGGCTGGTCAGAGAAAGAGTGTGGGCATGAATGTCTGAA

TTTCTCTGAAGTANGTATACGACTGCAGACTTGAGTGTGCATGAGCAGTGATTTTCGTAA

CGATGCCGTTTGATTTCACTAAAAAAAAAAA

>441

GGGTCTGAGGCCAGAGAGCGCTTTGTATAGGCGCCTGGGGGCTCTGGATGAGGGGGAACA

GAGTCAGGCGAGGAACAGTTTGTAGGGAGCATGGTCCAGGAGCGGTTTAGACGACCTCTT

TGGACGGCAGGTAGAGGGAAGGATGACAAAGACGCTCCAACTCTGGGACCATGCAAACAC

AGTTAAGCAAGGTTCGCATCAAAAAAAAAAA

>442

CCGGGAGTTACGACCAGGCAGATTTTCGGTTTCGGTCAACGGTTTTCAAATGTAGGTAAG

TCTTGTACGAATTGTTTTCGAGTTGAGTCGGTAGGCGCATTCCGCGTACTCACCAGCGCG

CACCCTTCCTTTTTGAAGACTTTTGGCTACAGGCCGGCATGTACCAGTACGCGTGATTGC

CTTCATACAATTTAATCTGCAAAAAAAAAAA

>443

GGGGGCTTTTTTTCGGGGGGGGGGGTGGGGGTTTTTTCTCCCCAAACCCCCAGGGAAAAG

GGCCCGGGGGGCTTTTTTTTTTAAGCCCCCAAAATTTGCCCTTTGGGGGGGGTTTTCCCG

GGGGCCTCCCTTTTTTTAAAAAAAAAAA

>444

GTCTACCAGGCATTCGCTTCATGGGGGATAGGGGCCGTGTGGCCGGATAGCTGGAGTACG

TCCAGACAGAGCCTGGCGCTTTGGAAAAAAAAAAA

>445

CAAGTCACAAGGAGTCACAGTGAAGTTCGAGATAATTGATAAGAGGTTGGGAATGAAGTT

AGTCACGCTTTGCGTGTGTGGGCAACAGTTCTAACTTAGTACGAGACATTTTGAAAACCA

GCTTAAAGTTTCCTGTACCTGCTGACAGTATTGGACTTGAAGAACTCGTCCACATGTTAA

AACAACCGGACGTTGGCGTCAAAAAAAAAAA

>446

CCCGCCAACCAGCGGGGGGATGTTTTTCCCCGGGGGGTTTCCACCTTTCGGGGGGGGTGG

CCCCTTCCCTGGGTGTTCGGGTGGGGGGGGGGTTTTTAATGTTTGGAGGGTTTTTCGTTG

ATGGGGGGGGGCCCTTTTTGTAAAGGAACCCCTGTCCTTTGTGTAATTTCGGATTTTTTG

GGCCAATAATTGGGTCCCGCAAAAAAAAAAA

>447

CAGCTAGCCATTTCAGATCAGGCAAATGTCGAGTTGTTTATAAGTTCCTTGAAGGATTAG

AAGTGTGTTTCTCGGATTGATTAGAAAGCAGGATTGATTAGAAAGCAACTTTACTTCAGT

CTAGTTCCGCAGTCCTGGTCAAAACAGCAAGTTCGAACCAGCAAGTAGCTTAATTGCGGA

ATATGACACCATGTACCATGAAAAAAAAAAA

>448

GCCAGCCCTTGTAAAAAGCTTCGCCTCCGTGTCCAAAACGGTCGGAAGACTTTTGCAGCT

TGAGTTACAAGCCTGATATGCGTGCGGAAGTAATATAATGGTACCCAGCCATCTTTGTAA

AAGTCGCTCGAGTTGAACGGCAGTTGATGCAAAAGCAAAAAGGCTTCAATCCAGTAGTGG

CAGCCGCCCCATCGGGCTTTAAAAAAAAAAA

>449

AAAAGGAGGCCGGCGGTGGAGATATTGCGTGGAACTTTGGCAAGTTCTTGGTCGACAAGG

ACGGGAAAGTTGTCAAGAAATTCGACCCCAGGACGAATCCGTTGGCCATAGAGCAGGAGA

TTGAGAAGATTTTGTAGGACATCAAAAGGAGGACGACCTGGTCATCTGATCGTATCATAA

AGAGCCAGTATGTGCACCATAAAAAAAAAAA

>450

TGCCGTGAGGGGAACCGTCTGGGCTGCCGACTGCTTGGAGCGTTGTGTGGTTTGTTGGCC

GCAGAATCCAGAGGTGTACTATAAGTGCGATACTATGCCACAGGATATAGACTCGCGCAG

GCTTGAGTTTATCGATCGCTGATAAGGATTGATGTGGTTGACTGCAATCTGGACTTTTTA

TTTGGAGGATGCGTTGCCAGAAAAAAAAAAA

>451

TGCAATTGGACAGTCATATGCTCGCTCAGTCAGAGTTTTGCGATAGCATAGCATGGGTTT

AAGCATGCATGCCCGACATGAACTTGCATGCATGTATGAACACATGCAGCTTGTAACCCA

TTGTGCAAAGCTAAGTGGACAGTCGGCCGGGACTCGACCAATTTTCTCCTGGTCTCGTCA

TCGGTTACGGATCGACTGCGAAAAAAAAAAA

>452

CGTTCGTCATTTTGGAATAAGACATTAGGCATTTGTCGTCTTGTTGTGTACAGCGGACAA

AGGCGCGCGCCTTAGCGCTCTTGGCAGGACTCAGAGTTGTAGCAGTGCATACTGGACTGG

TGGAGAAGGTTATGTCGGACATGCTTCTTATGCGATATTGAAATTGCACTGAGCAAACAT

CCAGTGCTGTCGATGTTGAGAAAAAAAAAAA

>453

ACAATTGAAAAGGGATCAGGATGGTGCACAGGAGGAGATGAAGGGGAGGCATTCTCCTGC

ACTTGGCGGTTTTTGGGGTTAGGACACCAAACCACAGAATTAAGTCCGCTAGACAGCGGA

AGATGTTCAGGGGGGAAACTTGGAATTGGTTGCACGTCTCTGTTGAGAGAGTTTCTGGCA

GTGCATGCAATTTGTCTTCTAAAAAAAAAAA

>454

GCGGGACTCGGGGGGGGCCCCGCGGTACCTGGGGGGGCAGATCCCGCCCCGCCCCTTCGG

GATGGTTTGAACGGGGCGGGCTGACGAATCGGAAGAAAGGCCCGCCCCAAACTTATAGAG

GCCTTCATGTCAGTTGTAACTTTCATGATGTCTTCTGGACTCGGGGTTGGAACCCGTGAT

CAACTGGGGTTGAATTTTTTAAAAAAAAAAA

>455

GTACGAGGAAAGTGGCCCGAGCATCTGTCGTTCGAACCGAGTGTTCAAGGGCCTCACTTG

AACAGCGTTGTCTTTACCGTATGGTAGCTTGTGACCATATGGCGATCAATTGCTCCACAG

AATAGTGGATAAGTTTTAATGATCCGCTGTCACGGTGGCACTCAGTCAGTGACGCATCGT

AACTTACACCGTTTGCATTTAAAAAAAAAAA

>456

GGAATGGTGGACCCAGCCATGTTCATTGAAATAGAAGCAGATGCTGTGATTTCAGACTAG

TTTTAAGATTTTAGGACAAGTCGCCCGGCCCTGAAGCGCAGTTCCATGCCGAACTAGCTG

TCAGGTTGGGTCGTTCCACGCAGATTTATGAAACGTGGAACCTGGTGATTTTTAAGCCCA

TTTATTCCGCGTTCCATGCCAAAAAAAAAAA

>457

TTTTTTTTAAGGGGCTGGGTTCCCCCCCAAAACCCCCCCTTTGGGGGGTTTTTGGGGCAA

ATTCATCCCTTTTGGGGGCCAATTTTTCCTTTTTCCTTGGGAAAACCCCCCCCCCTTCCG

GTTTTGGGGTTCCCATTCCCCCTTTGGAGGGTTGGGTCCCTTTTTTTGGACCCAACCCCG

GGGTTTTTTGGGGGGGCCCCAAAAAAAAAAA

>458

ATGATAGGACCGTCCGCGTTGCCGTCTAGGAAACATCCGTCGGACCATCTTCCAGTCTGC

TGCGAGCTTGCTTGGCCAAGATGAGATGCTAGAAGTGGCGACAATGATTCGGACCGTTTG

CATTAAGTAAAGCGAGGAGGGAGTTGAGCCGAGTGCTGGCACACCCTAGTCAAATAACCA

GGAAGGTGATTTGGCGAATGAAAAAAAAAAA

>459

GGCTTTGTCATCAATCCACCGGACGAGAAGTACTTCAAGTCCGGCCGCCGAGGCGTCGCG

GTTGAGGAAGAGGGGTCGAGCGGTGATACTTCGAGCGATGCTGAGCACGCGAGGTTCCAG

CCCGAGCCATTTAGCGGGGCCGGACCTCTGGAAGGAGAGACGGGATCAACCGGTTTGCGT

TCGAGGGGGTTTTAGTAGCCAAAAAAAAAAA

>460

AGAATCATAGGAGATCAGTATCGAGAAGCTTATGTTCCCTTTTCAGAAGTTTATGCCTAG

CTTAGCATTTGACGGTTTGAGAACGAGTTGCATCACGTCGCGCGCATCAGCGTTTAGTAG

AGTACCTCAAAATACCTTCTACGAGATAATAGTGAGAGCTATGAACTTTCTTACACTCTT

GAATTGAAAAAACTAGTCCNAAAAAAAAAAA

>461

CAGCATGCACCTAAGTAATTTTGTAGTAATCGTCTGGAGCCAATTACGAGGAGCGTTGTT

GCAAAATGTTGAGCATCGATCCAATGACGTCTGCGTTGTCCAAGAAAATGTGAGTGTTTT

TGAAGCTAGAGCATGTTTTCAAGTAGAGAAGACCATCTGAGGTGACAATCTTCATTGGAA

ACAAATCTCCGCTTGGTTTGAAAAAAAAAAA

>462

AACCTGGAAACCATGTCGAAGCAACGGCATCACATTCTTTCACAATACATTGTTGGGCCA

GATTTGTCCATCCAAGCAAATCAAGTTGGGATCAGAAAGAAGGAAGGGGAGACAGAGTTT

CTTCAGAGGTTTTATCTCACCAACTTATGAGGAACATTTCAAGACTCTGTTCAACTCTAG

CTTGCATGTGCTATGTTAGTAAAAAAAAAAA

>463

GTCACCAGGCATTCGCTTCATGGGGGATAGGGAGTTTCAGGTAGATCTCATGCAGTTAAC

ATGTATGGTTTAGTGAGGAACAAACAAAAACGTCCGTCCATATCATTGTAAATATTGGGT

TTGTACAGTCCGGTTGCGTTTCTCCTTGCAAAAAAAAAAA

>464

AATCGGCAAGGCACTATGTTGTAAGAAGGGTGGACGAGATTGTAGGGTTGATATAGTGGA

CGAGTAAAAAGTAATTAATCGTTGTCGATGGTAGAGTATTAGGTTAGGTTTTGACGATCA

GAAATACTCTGGACAATACCACGTAACAAGGTTCCACATGAACAGAGCGATTCTTGATAT

CGGTGCTAGCTATGCTACTTAAAAAAAAAAA

>465

AGAGCTTTACGGACGTCCTGATGGTGCTGAAAAGTGCGAGGGCTGCGTCAAACAATTCAT

AAAGAATGATACAGGATGTAATTAGAGATTCTCAGTCAAGGGCTCCCTTCCAATTTGACG

CGCAAGTGCCAAAAAGACATTATTAGCATGGTGACAGGAACGAAAACACAATTGAAGTTC

AACAATGGGCAAAGTTTCACAAAAAAAAAAA

>466

TTTTTGGCCCGGCAAATTTTTTTGGGAAAGAACCCCGGCTTTGGGTTAAGTGAAGCCAAC

GTCCCCCCCTGGGTCAATTTGTCCCCCTTTTAAAAAAAAAAA

>467

CTGTTGAGGCAGGGTGTTTTGGGTATCAAGGTCAAGATCATGTTCGACTGGGATCTTAAG

GGCAAGCAAGGGCCGTCCAAGCCGCTGCCGGATGTGGTCCCTGTCCATAAGCCGAAGGAG

GAGGAGGAGTTTGCGTCAAACCCTTTCATTGGGAAGGAGGTTGACGCTGGCATGGTTGCG

TAAATAGTGGCCCTTGAGCCAAAAAAAAAAA

>468

GACCTATTCCTTAGGCCGTGCGGCTAGACCATCGCACATTTGCAGTGCCCATGCCAGGTG

GACATTTGACAATAAAACTATTGCAGATTCATGGCTGCAAAAGATGCTCAGTTGAGTAAT

CATTTTCAGAAACAATCGTATACTGTACCGACCGTTTTATACACGCTACCCAATTTTTTT

TTTTAATGATGTATTGGGGTAAAAAAAAAAA

>469

CTTTCTGGACTCCAATATCGTTGTTGTATCTGTAGCAAGCGAACGACTAGACCTGCACGA

ATGATTTATTATGGCTTGTAAGATCGATATGAATCCAGGATTTGTGGAAGGTTCTGGACG

GCTTTCATAATAATCCTGCATACAGTAGTGGGTTCCTAGCATGTAGGTCCAGCTAAACTT

AAAAAGGGGTCGTCTCGGCCAAAAAAAAAAA

>470

TTGGCCTTGCCCCTGCCCCTGGGGCGGGGGGGGTCGTTGGCCCTTTTTAAGGGCCATCCC

CGCCCTTTTCGGTTTGGGGGCCCGTCCCTTCTTTTTGAAAGGGGTCCCCCCCCCCTTGGG

AGGGCCCCAACCGGGGGGGGGGGGTGTTCCCCGGGCCCCTCCCTTTGCCGGGGGTAAAGG

GGTTTCCCCGGGCCCCGGGGAAAAAAAAAAA

>471

CCCCGGGAACCAGTTTTGCCCAGGTTTCCCCCAAGTTTTTTGGGGGCCCTTTTGGATTTC

TCAAGTTTTGCTTCCAACGGGTGGGGGGTTTTCCCATTTGGTGGCCCGGCGGGGGGCCGG

GAATTCATTGGGGTTTTTTTCCCGGGCTTTGGTTTCATGGGGTGGGCAGTGGAAAGCTTT

AAAGGGGGGGGAGGTTTTTCAAAAAAAAAAA

>472

ATGTGTGCACAAAACAGTAGGTTAATCACAGATTGAGGTTCCGCAACAAGAAAGTAACGT

AAATATGGAAGTGAAGGAACCTTCGCTCATGTGATTGATACATAGGGTCTCGGAAACTTC

CGTTAATAACTTTTCTAAATAGATATATCAACTACAGATCTATACGAAACCTTTGCTATA

CTAACGCTTCTTGTTTGTAGAAAAAAAAAAA

>473

CATCAGGTGAAAAAACCTTGGTATAGTTTGATAACCAATGCTCCCAAGCTGAAATTCAGT

TCCAAACGCTCCAAATCTTGAGCTTTCTTTCCATGACTTCCTCCATAGAATGTAGGATAG

ACAAAGGAATGTAGTTCGGACTCGCGGTAGATAAGTGGAACTTGGTTCCGTGCAAACTGA

TGCCCGGCACCTCGTTTCTTAAAAAAAAAAA

***Coleochaete scutata***

>1

AACTATGTATGAGATTTAACCAAGCAAACATTATAACAAGCTAACACTACTTGATAAATC

CCAGCTTATATCTTTGAGTGATTTGGTGAACTCTACCAACAGGACGCTAGTTTGAACTAC

ACTGGTATCTAGGAGATACAGCAGCCACTGCATCTCTTACAAGAAACTCTTCGGCCTGTG

TATCTCGCTCAGTAGTCGCCAAAAAAAAAAA

>2

GGAGGGCAACGCACGAAACTAACTCTCTTGCACTGTTGCAAACACTTGCCCAAATATCTC

ATTAGGTCGCCAGACCTAGATGCCAATCAGCGGAAACCTCTCACTCTCATGTCACTGTTG

CCATTGCAGGGTCCTGACTGATGCATGCTAGCAACTGCGGAATGTTTTGTCCTGCTCTTT

GATTATCGTATGTCTATCGCAAAAAAAAAAA

>3

GTGCGAGCAACTCCAATATGTGTACATTATGTTGAGGACCAAACCATTGGATTACTTGTC

GCTTGTCACCTCAATGGCTTGCGATAATTGCAATCAACTAGAGTTCACCTGACTATTGGG

CCCATGCGGAAACACTTCCTCCAGCTTTGACCGGGATTGTTTTTTCATATGATCAGAAGT

AAACCTTGTAAACGTATGTCAAAAAAAAAAA

>4

TTTCAGCAAACTAATTTGCTGGGAAATGCAGACAGGTGTGAGAAGCTTGTATAGATTTTT

TGGGTTGGTGCAGTAAAGAATGTGCCCAGTTTCTTTCTCTGATCCTTTCTTGTGAATAGA

TATTGTTTTGTAGACATATAATCCCCTCATGAACAAGCCAATTTCAAAACAGCCCCAGTT

TTATACTTGGTCAAATTTTGAAAAAAAAAAA

>5

CCCAGCCTCCCCCGGCTGAACCTTTTTGCCCACTGACCAGTTTTATAAAAGGTATGTTAC

TCGGTTGTACATTTCAGTGCTGTTAAGCTGAGGTACTATATAGTGCAAGGATAAACTCAT

AAAGGCTGTGGCAATGCCCATCCACTCGAAATCAAATGTTGTCCTAAAAAAAAAAAAAAT

ATCTCATTTTTTTTGGAAGCAAAAAAAAAAA

>6

CCAAAGCCCCATTTTAGGACTGTCATATGTCATAAAATGTCAGTGTGAATTTGAAGCCTC

CAGTCACGCTACCAAGTGAACCAGCGCATATGTATAAGTCCTTGCATTCGACGGGAAAAA

AGGATGGCCCCATTGCCAACTAAGCTAAAACAGTGTCCCAAACCACCAACAGAATGTGCT

TTGAACACAATGAGGGTGAGAAAAAAAAAAA

>7

TTGTATTCTTCAGGCTGCTTCACTTCAGATTGGACTCTCTGGAAACGCTTATCAGATGAC

TTTGACTTGGGAAGGGAATGTAGTTCTCAGTATTTTGTTATCGCCCCGCAATATGACCTC

TGTTTTTTTGAGGTTTCACATCTTGACATGTTGACGCTGAGTCTTTGATTCTGTTGCTCA

AGAGAAAAAGGTTTAAGCCCAAAAAAAAAAA

>8

ACATATGATCGTGTGTACAGCTTGATATTGGCACTCTAGGATTTTTAGAAGTTGGGGAAT

TGAAAGGCAGTACTTCCTGGCAAGCAGCGCTGGTTTAGTGTGCTCTTGTGTTTGTAGATT

TGTCTTTTTGTGCATATGTAGAGATTGTAACTGTTTCATAAATTATGATGTGTCCCACCA

AGATTTTGTCATTTGAGTGTAAAAAAAAAAA

>9

TGGTTTGTTCAACCCGAAAGGAAGGTTCAGCCTGCTTGTTGTTATGTGCTTTTTTTCAAA

ACAGTTGGCAGGGTTTGTTTGTTCTGTTAACCTCCCTTGGTGGCCTTTCAATTGTTGGGG

ATCATCCTCGCCCCCTAAGGTTTTTCAAAGCCCTTTGTAAAGTTTGTTCCCTGTTTTCAA

TGCAAGGGTTGTTTCCCGTTAAAAAAAAAAA

>10

ATTGAGAAGCATATTGTTTTAAAACTTGTATATGCCAGTCTTAATTAATCCAAAAAAAAA

CAAGAGTAGAAGCCAACTTCCACAACCATGCTGTATGGGGCCGGGGTAGTCGAACTAAGC

ACAGTACAGTAAAAAAAGGAAGGAATGTGAGATTATCATTTTTATCAGCACATGCCTATA

CGACCTTAAGAAGGAACATTAAAAAAAAAAA

>11

GAGCATGCTAATGCCTTGTTATGTTGGACTAGGGAGCTTACTCGCATTAATCTATGGTAA

TGTGGGCTTCAAGACAAGTACTTTTATATCCATGATAATGTGGGTTTCAAGACAAGGACT

TTTATATCCGCTCACATACTTTGGGCAATTGTGATTGTATGAAATATTCTTTGATGAATG

CAAAAGTAATGAAGTGTACTAAAAAAAAAAA

>12

GAAGGTCTGTAGAGGGAAGGTTTTTTTTTACAAACAGCTTCTTGTTTTGTGGATTTTGAA

TTTGAGAGAGTGGTTATCACAAAGCATACAAACAGCTTTCGTTTTGCGGATTTTGAGTTT

GGGAATGTGAAAAGATGACATTCAAGGCTGAGAAATTAAATGGACAAGGTTGAAGTTGAA

ATGAGTGAAGTTGCTGGGTTAAAAAAAAAAA

>13

CTGCGAAGGTTGTAGACGGAGTGTTTGGATTAAAGACCACCCTTTGGTTGTCTACCCCTG

GTTGTCCTTGGTTGTCATGTAAAGAAGTTACTGGCAATAGTTTGGATTGTGCATGTTATG

TGTTGCTAACTCTAGGTGGGATAGTAACATTTGATGTAAAGAAGTTACTTGCAATAGTTG

GAATAGGAACATTTGATGTGAAAAAAAAAAA

>14

TACCTGAAAGCAAAGAAAATCTGGAAGAGTGGTGGGAGATTGCGGCTCCAGAGATATGCT

CATCTTTGTCAAATCATTTCTCCCTTCCAGCAACGGTGGGCAAACAAGAGCACAAGATGA

AAGGCAGGAGTAGTGGAACTATTGTGCTATGAGCAAGGTGATGTGGGTTCTTCTTTTGCT

GGAATACATCTTGTTTTATTAAAAAAAAAAA

>15

AATATGTGGATTGTGGGTCTTCAACATGTATGGTGGTCAATTTTTCTTATGTGCATCCTC

ATCATGTATGTGTACTTCAAGTGAAACTTGCTTTGTTCATGGCTCGTCTTTGAGACTATT

CTCATTTGACATGGGGGAACGATTGCGACCTGCGGAGTCCATCTTTTGGCTTGTTAAGGC

GAAAAAATGGGTGTGACTTCAAAAAAAAAAA

>16

GCCAAAGTAGAGCAAAGGAACAGTACAGTCTACGTGATTTCGTTTTTGCAGTCACAAGTT

ATGCCAAGCTATTGGCATCAGGGTTTTTGATGTTTCAGTTGTCCACTTTTAATCTGTACA

TGACGCTGAATCTCCCCATTGGATGCGCTTGCTCCCCGTGCAGCTGAGGCAGATCTGCCT

TAGTAGTGCTCACAAGTTTCAAAAAAAAAAA

>17

CAGGTTAGGGAGTGTCTCTGTAGACTGGATGCATCTTCAGGGTCCAAAGGGGTGCAAGTA

TAAGATGCAGTATTTGTAATTTTGGTTTAGGATAAGCAGGAACACAGCGAAGTGTTTCTC

ACTCGTTGCTAAGTGGATGGACGTCGTAAGGCTTATCAACTGCCTTCTCTTTGGTCAAAT

GTGAAGTTTACTGACAAATGAAAAAAAAAAA

>18

GTAAATATTGACTTTTGAAGAGGAGTGTAATAGATGTTGGTACTGTCTTTGTAGATAACG

CTGGGCAGCAGGTTGGACTGGGATTTGCTTCCAAAATGCTTCAGCACTGATGGATGCAAC

AAGTTTTTTGAATTTTTGGCAAACTGACTTCTCATTGTGGTGTTCACCCCCAATTGAAAG

TGACTGGATTTTTTTTTGGGAAAAAAAAAAA

>19

AAAACAGGAATAAGAATGAAATAAAAAGGAGTAATGAATCAGCAAATGTATTTCTTTACC

AGAAAAAATGTTTGAAAAGGGAAAAGCAGCTACATTTACACCAACCTTTCCTTTCCAAAA

CTAGAAATGAATCAGTAAACATTCAAATCGTTATGTAAAAACATAATGACGTGGTAATAG

TCGCGTGATCAATCTCTTCCAAAAAAAAAAA

>20

ATGGGGCCACAATTTCACGAGGATCTTGTTAAGCCATTCCACTCTTTCATACTCTGGGAA

TCGGATCCACGCTGGCACACTTTTTGGGCTGAAAATGGTTTTCAATGATCCGTAGTCTGG

AACCGCTGTTATAGCTGTGTGTCGGGCCTGCAAACAAAAAATATATCACATTAGTTAGAG

CACTACTTGCAAGCGGACGCAAAAAAAAAAA

>21

TAGAATGCTGTTAGTGTAGTCAAATGTATCTGGCTGGAAGTGTGCTTTGCTAGAGTAGGA

CATTGGTAAATTGTTCCATTTTGTAGCCTCTGGTAGATGGCTGTTTTAAAATAACAGCTG

TAAATTGTACAAAATGTTGTTGAATCTTAGTATACATAAATGTACACTCTTTTTGACTCT

TTAATATAGCCCACTAAGAGAAAAAAAAAAA

>22

AAATAGACAGCAAGCACTCTGTCACGCAAGTAACACCGCTTCTTGTCTTACCACTAGCAC

TGGCAAGCTGTGCAGGTTTAATTTTCCACCCATCAAATCAAGCCTGGCAAGGGACATAAC

GGGGAAATCATCACCATGATGCAATATCCTGCACTCCGGAAGGCCAGTACTTGCTACATG

CTGTTAAATGCATGTTCATTAAAAAAAAAAA

>23

TGTGTTAATGATAAGGCTGAGCAGCTGATTAATTTCAGCCTGGAAAGCAAAGGTTTCCAC

ATCTCCCTCCGGAGCCATGCTCACTTCTGGGACTTCGGTAATGGTGGCCATGAATTAAGT

CGCTTCAGAAAGCAGGTGGAAGCAACAAGATAAGCGATGGTGAGCGCGTACAGTGAGGAA

TTTTAAGGCTGTATCAATCCAAAAAAAAAAA

>24

CCACACTCAGGATCACCCCCGATCCCTACGGAATACACTAGCAGTCTCAAGCAATGCGTA

ACGGTCAAATGCCTTCCCTCATGCAAGAAAGGACTTGGGCACGACATGCCCTACATGTAG

CCCAAAGATATTAGAATTGATTTCCCTTGGTGGACCTCTGGCAACATCATTTAAGATGAA

GTTGTTTTGTGTTCTCTCTCAAAAAAAAAAA

>25

TTGCAGCACAGGGCTAGATACGACTTCTTGGTCCATTTTTGTACGCCTGAGGGTCGCTTA

TAGAAAAGTGCATCCCATCTGAGCTGACTGTTGTGTTGCTGACATCAGCCATTTGTAGTG

ATGGTCGTTGTACATGGAACTTTGCACTTTGTCCACAAAGACACTGGAATGCCATCTGTT

GTTCAAGCGATATTACCTTTAAAAAAAAAAA

>26

TGATTTTTCCAAAGGCCTGTAGATAGAAATTTCTATCTTGGTTCTGTTTGCCTATGCACT

TGTATAGTGGTTTGGAAGCTGCCTGGGTTATTGTACAGTGTGGTGTTTAATGGTGCAATC

TGTTTGTGTATTTTTGTGCAAAAAAACTGTTTTTATGCACATATGTTCTACCTGTGTCTT

GAATGTGATTCTGCTCTCACAAAAAAAAAAA

>27

TGAATCTGGCACATCTTCTGAAGGTTTGGGCAGTGCAAAGAATAACTTGGCCGCATAGAC

CAATGGTTGCAAAGATGGAACTGGGAAAGACCTGACATCAAACTGAACACTTACACTTCA

TTCAATACACTGTCATACATGTACATTCTTGATGAGCACAAGCAATTGCTCATGGGACCA

CAAATGGCATTCGATTTTTCAAAAAAAAAAA

>28

TGGCAAATTGAGCTCTGAAATTGTCTGGGAAGGATATCTTGAAGGCCAGATATTTCTAGG

GTTTCAGAGGTTTCATTAAGGAAGACGACTGTACAACCCTGTAAGACTTCCTTGTTGAGC

TATAGAATCTGTTACAATGCCACGTGAATCCTTTCAATCACCTCATGTTAATGTGTGTTT

TTTTCGCCATCAGCACATTCAAAAAAAAAAA

>29

TCGTATTCGGGGGCCTTGGGAGCTTCAGTGATGATAAACTGATGAGATGCTGTCCTACGC

TGCACATGTAAAGGGAGATTGTTGTGCTGTAAATAGATTTTTGAGGAAAGTAAAATTTGA

TCTGTTGGGATAGATTGTAAGGAAGAAGTATGTTTCTATGGATGGAACCTGTCTTCTTAA

TCAAATCTCTCTCTCTCTCTAAAAAAAAAAA

>30

TGGATTAGCTGTGAAAGTGCATTCGGTGTATTCTTGTAGAGAGTTGATGGCATCAGAAGT

TTTTGGGCTTGTTGTTCTATACTCTGAAGCTGTTGAACAAAGGTGCAACAGGTGGTTTCC

AACTGCAATTCTTCATCAACATTTTTTTGTATGAGCAGTCTTGCTTAATACCTGATCCAT

CAAAGTTTTGTTTGTGAGACAAAAAAAAAAA

>31

TGAGTTGATGCACGTATCANATCTCAAGTCCTAGTCCATAGCTTTGTGCTTTCTGTGCCA

CATAGTTCCTAAAGATGGGCCAACCCCTTTAAATGTATAGAATGTCAATGTGGCACTTAT

TAATGGGGGGATGGTTTGGCTGAAGGATTGGAGAGACATGACATGTTTCGCCTGTTCTAG

CANGCATTCGTGATTGTGTCAAAAAAAAAAA

>32

TAGATGCTATTCGGCAAACATTTTTGGTTAACGCACGGATGTGCTGCTGTTGGCATATGC

ATGGCAGGCAATGCATACGATGATTCAATCCCTCTCTCTCTCTCTCTCTCTCTCTCTCTC

TCTCTCTGTCTCTCTCTCTNANATGACCTCAAACCTCANGAGCTAAAATNAAGTCNAAAT

TTCGTGCAGGGTCTCACATTCAAAAAAAAAA

>33

GAAAGGGCCCCATTGCCTGCCTCAATGACCACCTTGCTGACCCCACTGCCAACAATGCAT

GGGCCTATGCCACCACCTTCACCCCTGAGCATAGATACTCATGCTTGTATTCGCATTTGT

TGACCAGAACAACCTGACATGCAGCTAATGATCTTGTATTATATATACCTTCAGTAATAA

AGACCAAGTGTAAACTCTTCAAAAAAAAAAA

>34

TGAGTATACCAGTCATATTTTACAAGTAACTGTGTACACGAACTGGCTANGGCGCCATGA

GACCTTCTCCCCGTGCATGGTGGCGACTAAATGATTTTTGCTATGATCTAACCGTATTGA

TCGAAAGAGGGAGTGACGCTTGAGAAGGTGTTTTTTGACTTTCAGTTGTTTTGTAACTAT

GCCCTTGAAAGGTGATTGCTAAAAAAAAAAA

>35

TGGGCTCCCACCAAGGAACTGCTTCCCCCTGCTATTTATTGGTAAAAATTTGCGTGCTTA

GATCCCCTTTTTGGTCAATGTGATGCCCCCTTAAGTTGCAACTCGCATGTGCTCCTTCAA

ATGTTTTCGGGAAAGGCCTCGGGAATTTCCGATATTTTTTTCTTGTGTTATTGGGGGGAG

TAAAGGCTAGTTTATTCCTCAAAAAAAAAAA

>36

TGGTGTTAAGTAAATGGGCTTTGCCATTTATATTGCTCTTTGCCATTTTCACATGTAACC

TCACTTGAGGGCATGTAGGGTGCAGTTTTGATGCTATTTGCATATCTGCAGAAGAATTGC

TGAGGTGTTTTAGCATGCTTTGTACAAAGGAATATGAACATTGGAGGAACTTTGAATATC

AATTGTACAGTTCTGAGGGTAAAAAAAAAAA

>37

TGGTTTTGGCAGCTCCCCCGACCTCGCTCCAACCGTCCAGATGGCCTTCCAGTACACGGT

GCTTTTGCCAGCCGAGGACAACCTTTCCCCTCAGACCCCTCCGAAGGCATTTAATAAGCC

CGTGATTTTTTGATTTGTGACTCGTTGTAAAGGTTTTCCTCCCCCATTTAACGCTTGCAA

TTGCATTTTGTATAAGTGTTAAAAAAAAAAA

>38

TCGCTTTTCGTAAATCCCAAGGAATACCTGACCCTCTTAACATAACCCCACTAAACCCCG

CACCTGACAATGTCTTCCAAAAAAAAAAA

>39

TCGCTGCAAGCTTATTCGACACACATCTTTTGCGGCCTGATGGCTTTCTCAACAGGATCT

GGAAAAATTGTAACCCGTGCTTCGCGGCCCTAATCTGGTGTTCCCCCCTAAATGCGGTTC

GAAGAGATATCCGCGGGTTGCGGTAACGAACTGTGGAAATGCAGACCGATGCAACGATTA

AGTTAATCTCGCACAGTTCTAAAAAAAAAAA

>40

TCTGAAAGCTGAGCTGGTTCAAGTCCACCCCTGTCTCAGCTCATTGTAAAGTATGTGCCA

TGTGAACATAAATTACAAATAGTCATCCATTGAAATGATTGGGTGACTGAAAGATAATCT

GGAAGGTTCAGAGTATTGCGCTGTCCTGGTTTAAAACAGGAAGAATGCTGTTGTAATGGA

AGGCAGCGAGATTTGATTTTAAAAAAAAAAA

>41

TTTACAAAGCAGTTCCCATTTATGCATGGGGTTTTCCGTATCATGCTTTCAATTAGGGAC

GGGGGGAGCCTTTGAGTATAGCTGAGCCTTTTAAGTGTTTGAATAAGGGGGGGGGAATTG

TAAGAAAGATTTAGTTGTCCCCCCAAGGAAAGTAGTTTTGGGCCTTTTGGAAAAGAGAAC

AATGCATTTTTTTTTTTTGTAAAAAAAAAAA

>42

GTCCATATGTAATATTTGACTCCCAATGAGGGCAGCAGGGAACAAGCCTGCCCAGATGGT

GGAAACAGTTTTTGTTTTTTTTGTTGACACTTCCTCCCATTTTTACGCAAAACATAGTTT

TTACCAATTGTTTGTACATTTGTGGAAGCCCTTTGATATTACAAAGCTTCCCAGAAATAA

GCTGTGCTTGACCCCCCACCAAAAAAAAAAA

>43

GACACCACGCAGCTCAGGTCCACTGTTGCTGCTCAACGACATACACATCCACACCCCAGC

TCAACGTCCACAATGGCAAACGGGAGTGCTGCTGCTGCTGCTGCTTCGGAGTTGGAGGGG

GAGGAGGGACTAGGAGGGTTTGACAATGATTGTCAGGCAGCAAGAGGAATGCGGACAGCT

ATGTTTCCATTGCAACAACGAAAAAAAAAAA

>44

TCAACAAGGATACATGAACGTCCCGTACACTGCTACCGCAACTACCTACACTGATCAAGG

CGATATCGTAGTCATTAAAATGAATGACACATTTAGTGGGATGGCATCTTACAATGTCAC

TACTATCTATCACAAACCTCAACCCATTGACTAGACCTCGAGTGTTTGGATTGTATAGGA

CAACATCGCTTGCTTATTTTAAAAAAAAAAA

>45

GCAAAGTGCAAGTAGTTAGAGCAGTCTGTTTGCTGTCTCTTTGCAACTCTGTTTGTACTC

TGTATGTATTCAGTGCCCCAGCAACATGCTGTTTGGACTCTGTTTGTCTCCTGGTGCCAT

CGGCAGAAGGGACAGGCTGATGTTTAAACTTGTAAGCCCGAGTGCCAAGGCCTTGTACAG

CTAAATTGTGAGTTTGAAGTAAAAAAAAAAA

>46

ATGAAACAAGGATGCTCCTTGTCCGCTGTGTTCCATGCTGGGACTTGCTTGGGTTGCAAA

CTCTGAGGTCTGTACATAAGTGATAAAAGGCACCATCGAGGTGCCCACATTGTAATGATA

TTTTTGATCGGGATTCTGGAACAATGAAGCAGCAGTTCACTGCAAGAGCTGTCTGGACAT

CGCCAGTAAAGCAACTGATTAAAAAAAAAAA

>47

AAGGGGGGTTTTTATGTTCCCTCCCAATTCCAGTTCCAGTTGGGATTGTTCAATTCAGCT

TCCCTGGGGGGGGCAAAAAAAAAAA

>48

GCTCCTTCATGCAAACTAGAAAATTTGGTTGCCCTCTTGGTTCATGCTCCTTTATGCCAA

TCAGAAAATTTGGTTTTTTTTTTGCATCAGGGTTTCCTATACAAATCAGAAAATTTGATT

GCTTTCTTGGGTCATGCCTTCCAATGCAAATCAGAACACCTGACTTCTTTCCTTTCAATG

CAAATCAAAAATTTTGGTGGAAAAAAAAAAA

>49

AAACCATCGGTAGACGGGCCTTATTAGAGATTAGGGGTACGGAGAGACGCTTGTGTAGGT

TTCTGCATCACCCTGAGAAGGATGTTGTTGGTCATTTCTGATTTAGAGTGTTTAAGGTTA

GAGTGGTTAACAAGAAGGTTGCCCAGCTCCGAGGGAATGGTGCCTCTCAGCTCATTAAAA

TGTTTTTTATCATTTTTTCCAAAAAAAAAAA

>50

AGGCTCCCCTTTTTGTTTTTCCCCCTGTCCCTATTTTTTTGGGGAACGATAAAAAGTGAA

GGCCGTGTTTTTTTTTTCTTCCAAGCTAGAGATGCTTTTGGTAAAATACCCCTTGGGGGG

GGGGCCTCCCCTTTTTGATTTTTTTCAAAAAGGGGGGGGGCAAACAAATTTGAATTGGAA

TTTAAGGCATTTCCCCCTTTAAAAAAAAAAA

>51

AGGGTTGGGCGGGAGATACTGTACAAATATTGCAGTTTGCAGCAGCAAAGGAGGTCAGTA

GACCTAACTTCTTAACCATATGTTTCCAGTTGTGAGTTACCTTGTACTCAGGTCAACAAC

TTTTGCGAGTGTTGGCAATGAAGCCAGCAGTTTGAACTTTTGGTCCCAACAGATCGTCTA

TTGGAATACATTTTTGTTGCAAAAAAAAAAA

>52

ACAACCGAATGTAGGAAGTCAACGGAAGCCACTCCGAATCCAGGGTATGCCCAGTCTTGG

TACCATAGGAGCAGCTGGAGGGTTTGTAAAAATTGGGATTGTTGAGTGAGTTGTATTTGC

TAGGGCACCAATAGTTGTTGGGCATCACCCTGAACCCTTCGATTGACAGCAGTGTTTCGT

TGTGCCATTACTTACTGAGTAAAAAAAAAAA

>53

TTGCCTTCACCAAACGGCAAGTTGGGAAGTGTGATACATGTAATGTAGTCGTCACGTCCA

TGTCGAGTGGGCAGCGACATGTTAGCTACTACGGATTGCGAGTGCCTTGGGATTAGAAGT

TACATACCGCAATGGTGAATCCTACAGTTAAAAGATTGTATGATTGATCTTGGATCTTTA

CAATCAAAGATTTTAATGAGAAAAAAAAAAA

>54

GCCGACTTGTGGCCTAGGATTGCAAGCGCTGCCAATGCTATTGTTTGAACTGACAGTGGA

ATCCAGTGAGTTCGATATTTGCAATTTTCTCCTCAAACCCACCAGCCAGACTCCCATTAC

ATTTCAGTGATGTGACTCGACAAATCCTGTGCAATCATATCATATAACCATGGTTGTTGA

AATATTACCCTTTTTTTGTTAAAAAAAAAAA

>55

CTAATGTGACATCCTTTAGTTTCGGAGTCTCTGTCTAGCCAGTGCTGAGGAAGTTGGTTT

GGAGTATATTTCCCCAGATATCCTGTGGATTTTGTGGCCTCTCTTTTCTCTCATTAAGCT

GGAGGGTTTTTGATGAAATGATATATTAAACCTCCACTGTTACTCTTGACCATTAAGTTT

TTCTGCTGTGAGAGCACACCAAAAAAAAAAA

>56

GGTTTTCTAGGAAGTTTTGCTGGCATCAGACTTTTGGTTCATCATAATTGAATTGCCAAC

TTTGGGATTGAAAGTTTCCTTGCTGTTGAAGTGCAGTACAAATTGTTGATACTAAGAACC

AATTGGGGTTTCAATGACTTGCTGTACCGCCTGCAAGCTTTGTCATGTATCTCTTGTGAT

GGTACAATGCACTGATGTTGAAAAAAAAAAA

>57

TAGGCTTAATTGGTGTCACAATGTGACTATTAAATGCTCTTTTTGTTTGAGCAACACCAT

GTGCTTGCTGTAAAACGCTGTTAAAGAAGGTAGGCATATTTGATCGTAGTTACTTGGCTG

CAGGTTGCTGAAAGCTTCCCTTTTTCCTTNCTTTNTTCTAGCGCTCGTAACCATCATTTT

TAAAGGAATGCAATTGTTTTAAAAAAAAAAA

>58

GAAGGGCAAGGGCAAGTTCACAGAGGGAGAGGTCCAAGCTATGCAGGATGTCGATTAAAT

TTTTCGATTACTGCAAGCATAGCGATGGCAACATGACTAATTTTTGTGATTGTACATCAT

GTCTGTTTTTAAGTAGAATGATATTGAATTCAATGATGGTTTCACTTGGTTGTTCATGGG

AAGTCCCTTTGCTCTTTTTTAAAAAAAAAAA

>59

TTTCCATGTTTGGGTTTTTTGTTCAGGCCATTGTCACTGGCAAGGGCCCCATTGCCAACT

TGAACGACCACCTGGCTGACCCCGCCGTCAACAATGCCTGGGGTTATGCCACCCAGTTCG

CCCCTGGACAGTAAAGATCTTGGTAGTGTCCTGTGCATAAAGACCATTAACTGTGTATGA

ACAAAATAAACGAATTTTGGAAAAAAAAAAA

>60

CTAAGGAAGCAGTCAATCGGTCATTATGTGTCCCTCTCTGAACAAGTTTTCAGGTATCAG

AGTTTGTCATGGTCTGACTTTAAAGATGTGTTGATCGAGCTAAGTAATTGAGCAACGTAG

GGTATTGTCTTGAGGAGTACTCTGGCCCCCTTTGTTAGGGAGAATCCGAGCTGACTAGAT

GAAAGTGTCCAGGAGCTTCTAAAAAAAAAAA

>61

GAGCATGTTGTACTTCATACATTCGTCGCCCTTAGGCTTCACTTTCTGGTAATCATGTAG

AAGGGGCACGCTGTGGAGTTGGCTCAACCTGAACTTGCAGCAGTATTGTTCTGTAGAGCG

TCGGTACTTGGTCGCTTATAGGGTTTTGGACTCCAATCGTGGTGACACATAACTTAGTAT

TATGACACTTGTATCATGGCAAAAAAAAAAA

>62

GTTCATGGATCGTCAGCAACAACGTTGCTGTCGTCCGCGGGCTGTATAGGGTGGTGCAAC

GCTTGGGAGGAGGCCTGCCTCGTCTGTCGTCTTCTCGACAGGCGAAAAGACGCCTTCGTC

CACCTTGGTAGCTTTGCGTCCGAAGAGAGATACAGATGTAGACAAATCCTGTAATGTTTT

CCCCCATAAATGTCAATGATAAAAAAAAAAA

>63

GGACTCGATAGAATATTTAGAGAAGGTATTGTACTCATATTTACAAGAGCAGCTTTGATT

TTCAAGAGATATTCCGCAGGTGATTGAGATGGCAATTTTGAGAGATCACGACCAAATCTG

CTTGAATCAAGTTGAACCTTGTGCAGTATTCTCAGGGCGCTATATTCTATCCATGAACTG

CTTTATATTTTTTCCTCGATAAAAAAAAAAA

>64

ACGAAATCAGAACAGAGTGATTGGTGCGAAAACAACGCATAATTGACCTACTTCTATGAT

AAAGATGGATTGTAGGTTTGAACATATAACAAAGGTCGTGCTTAAAGTGGTTTTGACAGA

CGTGTAACAAGCCTGATGTACATTTTGACAACCTCTATCAGCAGCTTCGGCAATCTCCTC

GGATTGCACTTTTCCATCATAAAAAAAAAAA

>65

CAATAGGTTCACAAGTGGCAGGGAATGCTCCCTCTCCACGGGGCTCACTTTTCCTATCTC

AAATGTGTACACTGAATGCTTCCTAGGTAATTAGTTGGTGGACCATCAGTGTTGGTAACT

GGCATGCACACAGGAGTATACACTTTAAGTGCATAACTGTTGCTTCCGCGCGGGGTTGCA

GGTAATTCAACGGTTGGCCCAAAAAAAAAAA

>66

GCCTGTATGGTTGACACCGCCCTCCTGTGGAAGCGTCCTTGAGAGATAAAAAGGAGTACA

GTGGTACTATTTAAATCGAGGTGGGAAAATGTCTCCTTGTCGCGTCCTGGAACTAAGCAT

AACATGATCGTATCTCAATTGGACGCCAAGAGGGGCTGAAGTAGTACTGTCAATTCACAA

GAAACCGACGATTGACAACGAAAAAAAAAAA

>67

CCACATTGCTTAAGGAGTTTCTGTTGCATCGCTTTGTCAGTACTAGCGCCCCTTCTGCGG

GTTGCGATAAACATTTGAATACGGTTGTACTTTTAGCTGGTGCTTTTCATGGTGCTTTTC

AGGGTGCTTTTCAAAGATAGGTAACGACTTCGATACCTCATTTGACACACGTGACGACTT

TGATACCTCATTGACCCATCAAAAAAAAAAA

>68

GACGCGAAGCTAGCCCGATGGGACTGGACTGGTTGTATTATGGATATGGGACGAGTACGT

GGTATATTCCGCGGTTGTGTACCCGTTACTTAAAAAAGAAGAAATATAGGCGGTTGAAAA

ACTGCAGCTTAAAAGACTTTTGGGAACTTAAATTACAACTGTAACAACTGTAAGTGACCT

CTTTATATATGTCGATCTTGAAAAAAAAAAA

>69

AAGAGGCTGTGCGTCAGCATGCCCACATCTTCCGTCTGCCACAAGTAGAGTGCCTCCTTG

GGGTTGGAGGGGCTTGGGACTTCTGTAGTCACATTAGTCTCAAAAGTGTACATTGGACTC

ATTGAGTTGAGGGAACCTGCCTTAATCATTCGGACTCGCATGCGTGTATTTAAGTATAGG

AATGAGCAATTGTTCTATTTAAAAAAAAAAA

>70

GCCGTGAGGACCGCCTCGCCCAGGGGGTCCAGGATACCGCGGCAGAGGCGGAGCCACAGC

CTGCGGACACCCCGGCTGAGGTTCCGGAGAAGAAGGAGAAGAAAGCCAAGAAGGAGAAGA

AGTAGGCACGAAAGACTCTTCGCATGAACTTCGCACTTGGCCTTGTACATTATCCCTCTT

GGATCGTTTGGGCGATCTCCAAAAAAAAAAA

>71

ATGCGGAAACTACTGTAACAATCTGTACATATAAGTGACTCCCTCTGATAACTGCGCTTA

ACGAGCACTAGGCTATACTGCCAGGGGAGGCGATATCGCGCCAAGCTGACTAGCTGTAAG

ATATTGCCAGGATACTGCTCGGGACATCGCTTCACTTACTTGTACCGTGTAAGATGCAAC

GCTCGATCGTTTCAGCACTTAAAAAAAAAAA

>72

TGCCAGATGTTTGGAAAGTCATGTAACAAATTTGTGAGGATCTACATCTTCTCAAAAGTG

GCGCCTTGTGTACAGACGGGGAGACTTGTGATCTACGATCTTCTACAGTAGCATCATGTA

TAAAGCCAAGACATTGTGAACTCAATCGGTACTTTGTTGTTGTGAGTGAAGGGTGGACTA

TAGAAGCTGTGCTTTTTGGTAAAAAAAAAAA

>73

TTTTGAGGGTGAGAGGTGGGGTGTAATTGACATATTTGTATATAATTCATTTTGCGCTTG

AAAAGAAGTAATTGTAACAGATTCATTTGATTTGTGCTTCAGCAGACAGCACCTGATATG

GTAAGAAGATATAGGGATAAGAGAAGACATGGTTTTTTTGAGCCAAAACCTGAAGACAGT

TGGAAATATATATTGATTTGAAAAAAAAAAA

>74

CTTGCTGACCCCACCGCCAACAACGCCTGGGCCTATGCCACTAACTTTGCCCCTGGACAA

TAGATTTTCAAATCCCTTTGTTGATGCTGCCCCTTTTGTTAGTTTTCCGGGCTAGCCCCC

GTCCGCTGTGTAGTCCGTAGTTTGGGAAGACATGGGAAGCTCCTGGGTTTACATATTTTG

AATAAAAGTGGGCTTCCTGGAAAAAAAAAAA

>75

TTTCGTGCCATACCTGAGAACGTTCAGCTGTAAATACTGCGCAAGGCCCTTTGAAAACTA

TTCGCGTTGATGGTCAGGATAGTTGTTTTGAAACCGATGGACGGTGTTTGTACGAATAGT

TGGAAGCATGACATTTATGTACTTTTTGGCAGTTCTTTCTAAGACCTGATGTATGAATAG

TTGGAGATGTGACATTTGGGAAAAAAAAAAA

>76

TTGCCAATCAAAAACCTGACTTCTTTGAAGGCCTTCTGGTTGAAATGCAATCTTGGGGTA

AACGTGTAAACCCAGAATACATTCAATTGTTTCTGGTTCGGGCCGCTGGTATCGTGCGCT

GTCATGTCGGAGCCTGAATGGCCTCACTTGGATGCACCTCTGCCATGCCCAATTTACACC

CCCTGTCAGCTTTGGTTCTCAAAAAAAAAAA

>77

CAAGTTTGTACGAAACCGCTAGGAGTCATATCCCCTGCATGCATTGGAATTTTGAATTGA

CAATGCCCATGCAATTTGATGTTTGGAAGTCGGTTTTGTATTTTTGAAACACTTTTGGGT

CAGGACACTCTCATTTATTAGGTAATATACGTTTGACTCAATTGACATATTCGATTAAGT

ACAAAACGTCAAAGCCCTTCAAAAAAAAAAA

>78

GGGTGGTTTTTTGTCCATGGGGGGGTTTGTCCCCTAGTTTCTTTGTTTGGGCAAAATCAC

CGGAAATTTCCCCCAGGTGGAATTTTTGAAAGGTGGGGGGGTTCAAGTCCCCCCCTGTTT

CAGCTCTTTGTAAAGTTTGGGCCCTGGGACCCTAAATTTCAAATTTTCTTCCCTTGAAAT

GATTGGGGGGCTGGAAGTTTAAAAAAAAAAA

>79

TAGCATGCCTCCCCTGATTGCAGGTTGATGTATCACAAACTTGACCTCCATTTGAACCCT

AAATTAGGAACCTTTTTTTTTTTTTTTTAACTTAAGCTGACTACACGACATTATCATTAT

CGTTCCCCCCCTTTTCAGGGTTTGAACTCCTTTGTCATGCCTATTATATAATGAATATAT

ATTTTGGATTTGTTGGGTGGAAAAAAAAAAA

>80

CAGTACATGAGAGGTTGATTATCTCTTTTGCCATGTTGATTATGTTGCCTTATACCAATT

GTCTTGCAACATTGCAGTTGAAAGGTTATGTATCAGTTAAAATTTTGCATGTAGTGCAAT

TCAGTGAATTTGAACAATCTTGTTGGATCATAAACAAAGAGGATTGTTTTCATGAAAATA

TGGGATTGTGGACTTCTTGTAAAAAAAAAAA

>81

GTTTTTCTCTTTTGCAAATGTTTTAGCGGGAGTGTGGGGAAGTGATTTTGTGAACTTGTA

CTATATGGTCTTGGCCTGATGTTGTAGCGGGGGTCCAAGGGTTGTTGTGCTTGTTGAATG

AGTTTTTGCTTTTCTTGCTCGCTCTCATTGCAATGACCAAAACTAGGAGGTCTTGGCTTA

ATGTTTGTTCCCTATGGGTGAAAAAAAAAAA

>82

TGACGTTAGGAATCAACGTAGCTCATGCAATGTGTTTGTTCAAGCAAAAAGCATGGATGT

TATGATGATGTGTGACGAACTGATAAGAAACTCTTGGGAATTGAGGTGATGTGCATTGCA

CGTCCAAAGAACGGTGCCCTACATTCTTTCCAAAGCTGACATGAGAATGGAGACTTTCCG

CTCTCAAATGTTGATGCCTCAAAAAAAAAAA

>83

ATTTATGCTGTCTGGTTTGCTGCTAAATAGAACATAATGGAAGATAAATAGAACATGATG

AGAAAGCAGAAGTGAGAGATGTTAATGGACTCTTTGGCAGTGGAACAATTTTTGTACGTA

GTTTGTGACTAACTGGTAAGATGCTGCCAGTACAATTGCACACAGATGGCTTGATACTAT

TTATGTACACTTTTTTCAGGAAAAAAAAAAA

>84

GTCTTCCTTGTTCTTCTAATCCAGCATGATAATAAGGAGTTATTTGAATTTCACACAACA

GCGGGATTTCCATAATATTAGTATAATTACATTTCGCCAGAAGATCAGGACGAATTACGT

TTTGATAATGAAATAGTAATCTAGGTGCGAACGAAGTGAGCACAAATTTTTTCATTTTGT

TTTTAAAATTATTAGTAACTAAAAAAAAAAA

>85

TACTCTGCTTTCTCAAGGTGACAAATTTTGAGAAAGCAGAGTACTTGCAACGTTGATGGT

TCTGAAAAAAAAAAA

>86

TTTCCTTTTTTTTTTTCCCCCCCATTGTCCTGCGGGCCAAGTAAAGCTCCCCAACTTTGG

GGTTTTTTTCGCCCCTTCAGTGTTGTTGATGGGGTATGTGTCCTGAAGTACCCCTCTTTG

GATACCAGAATTTTTTTGGGGGGGATTCCCTCAGCCAGCTTGGATAGGGGGAAAATCAGA

AATAAATTTTTTTGTTTCCTAAAAAAAAAAA

>87

TCTTGTCCATATGAGCTACTAGCCAAATTCGTTGTTTTGCGTCAAATTGGTTGGGTCAGT

CCAGCTGTCAATACGTTTCAACATGGAAGGAGTAATTTTTGCCTAATTTTCACCATTTCA

AGGTGTCTACGAGGTGAACTGTTGAATGGCATGACCATGTTTCCAAACGGTTTTTCCTAA

TTGTTTACCAACCACTTGATAAAAAAAAAAA

>88

GATAGTTTAAATTTGGTTTTGGTAAAATAAGAAGGGAGGTTTTCCTTTTGACCTTTTGCT

GCTGTTGCTTGGGGGAAGGGATTCTTGGGGCAAGGGTTACCGATGGCCTTGGCAAATGCA

TAGTTCAATTTCCCGCTTTTTGGGGGGGTTTTCAAAGGTTTTTTCTTGCCACCTTCCTTT

TAGAAAATGGGGGGTTTTGGAAAAAAAAAAA

>89

CTAGTAGCACCTCCAGCGACCATAAATCTGCCAGTACTTGTCAATGTGGACGTTAACTCC

AGCGACCATAAATCTGCCAGTATTTGTCAATGTGGACGCTAACTCCAGCGACCATAAATC

TGCCAGTATTTGTCAATGTGGACGCTGACCTCGACTGACAAGTAATGCTGTAATAAGAAC

TGACTGATCTCTGTTTATGTAAAAAAAAAAA

>90

CTGATCTCAAGGGTGGAAATCTGAGAATCCTGTGCTTCTCGGTCTCAAAGCATTTGCGAT

GATTATTTCGAAGATGGATCTTTGTTTCTGAATTGTTGAGTTTGGATTGTTCGCAACCTT

CATCGCAAATATATAAAAAGGATTATTCAATATGGACATTCGATTCGATTATACATAAAG

GTTTATATTGTTTTTGGTGGAAAAAAAAAAA

>91

TGAGGATAGGGAGGTAGCTTTTTTGGCTGGCAAAAAAAAAAA

>92

TAAGTGTCTCCTTAGAGCAAAATTCAGTGCAGAGATTAGGGTTTCTGCTCTCTCTCTCTC

ACTGCAGGGAACTGTCGAGTTGTCTTACTAATCACCAAGGCCAGGGCTACGAAAGTAACA

ACTGTGTCATTGGGAGCTCAAGTGGAGCGCTTCATTTTGCTGGAAATGTTGCCAAAAAAA

AATGTATGTGTACAATTCACAAAAAAAAAAA

>93

GCATTTTTTCAGGGGATTTTTTAGCAAGGTTTTTTTTTTGTAGACAGTTTCCAATAAAAG

GCAAAAGAAGTTTTTTTTTTAAGAACTCTTTTGGAGTTTTTTAGTAAAAAGGAAAAAAAC

CCTTTAAGGGGTTTTTTTTTGCAAATTTCCACGGGGAGGGGGGAAGGGTTTTAAGGGAAA

AACAGGGGGGGGTTTTGTTGAAAAAAAAAAA

>94

TACGTCTTAAGGAAATGGAAGAGGAGAGGATGCGTATAAAACGTTGTGTTGATGTGAATA

GGAGTCGAAACCATTTCAGAGTGTCCCTGTGGTCCAATGTCCATTGTTTATTAGGATGTG

GCACACTTTGGATTGCATGATATTGATGCTTAGGTTTGCGAGACTTTGGCGAGTAAACAC

AGCCACACTCTTGGTTTTTTAAAAAAAAAAA

>95

AGACATTCTCAGGTTTTAACAGATTCTCTGCTTTCTTCTTTTTTTCAGAAAGGGACGTTG

ATACATTCTCTCGATGTTGGTTTCATACATTCTCTGGTTGTAGCTCTGGTTTCTGTTTTT

TAACAGATTCTCTGGTTTCTGTTTTTCAGAAAGGGACGCTGATACATTTTTTAACAGATT

ATCTGGTTTCTATTTTTCAGAAAAAAAAAAA

>96

GAGCCCATGTGAGCTGAAGTAGAGCTTGACCTTCTTGTAAAAGTTCAACACGTGTATGGG

TTGTGTGCAAGTCAACCTGTGCATATGAGAGAAGTCGAATTGTTTGTGTTCATCCATGGA

TAATGTTCAAGGCTATCACATAAGCCATCTGAAGCTGGGCAACTGCCAATAGCATTCTTG

CTGGCAGTCATTTTCTTGTCAAAAAAAAAAA

>97

CGAGATACTGTTCCAAACCTTGTAAGCATTCTACAACAAGGAGAAAATCGGAGAGTACAA

AGGTACTTCTCATACACATATTATCCTTTTACATCAATCCACCTCTGCTTCATTTGTTCT

TTTCTCTCCAAGCAGTTGATCATGCAAAATTAAACGACTTCTACTGTAATTCTGCTGTAC

AGTTGGTCTCATTGACGTTCAAAAAAAAAAA

>98

GTGCTTGCCTTTCATTATGTAGTTGAATATGGGGTTAAAGAACTGGGGGGATCAGTGTCC

TGTCGTAGGTCAGAAAAGTGCAAGTTTTTTTTTTGCCTTGTAGGGAGGTATGCATTTAAC

CATTTAGGCCAAGAATTTTCCTGAGTTTGTTGCCCCGATTGGGATCAATTTTTCCTGCAA

AAAGCAGGTTTGACTCCCGTAAAAAAAAAAA

>99

TTTCCCCCGGGAAACAGTTTTGCCCTTGTTTCCCCCAAGCTTTTTGGGGGCCCCTTTGGA

ATACCCAAGTTTTCCTTCCACCGCGTGGGGGGTTTTCCCATATGGTGGCCCGCCGGGGGG

CCGGGAATTCATTAGGGTTTTTTTCCCGGCCTTTCGTTTCTGGGGGGTTGGGGCAATTAA

TAAAGGGCGGAGGCTTCTTCAAAAAAAAAAA

>100

CCTTGTCAACCTTACACTCTTATCACACGTATCTGGTGCTTCCATATCTGGTGGTGCTGT

CAACAGCTTAAACACCTATGGATAAGACAAGTGTGTGCCAAGACAACTGTACTCTTTCTT

GGGACAAGATTCTCTTTTATAAGCTGCAAGATGGTGACATGTAAGCAGTCTTGGAATATG

CATGTTGTGAATTAGCAACTAAAAAAAAAAA

>101

CAAACATTGTGCAAATTTGTGGATATGTGAAGGAGGGATTGCAAATAGGGTAACGGATTG

AATTAAGACTGTTCATACGCTTGAGGATACAATTGGTGATACTGATGATAATGCACACTA

ATTTGGTTTCTGTTCATTACCAATACTCTGTCGGCCATTCAAGGCTGGATTGTTTATCCC

AAATTATGTGCCTCATTGGCAAAAAAAAAAA

>102

GGGAATTTGTTTTTTGAAGACTTGGTGAAAGGTTTTGGCCGGACTTACAGACGGGTTTTT

CAAGTTTTGGGGGGGGGGCCTTTGGACTTTTGAACTGGTTTTTATTTTGATGTGGGCCCT

TTTAAGGATCGCTGGGGAACTGGTTTCCCCCCTGGATAATGTTCCCCCCCCCGTAAACCA

TAAAATGCCATGTTTGTCCGAAAAAAAAAAA

>103

TCTTTTTATGGCTGCCCTTGTATGTCGTTGCTTGGTGCGGACTTTAAAGTGCAGGGAAAT

GTCATCATGTGGGCTCATGGGGGGGGGATATGCTTGTGATGATCTCCGTAAGTGTATTGT

AGCTTTTCCTACAGTTGCCAAGTGCCAACAATCCGGGGATCATGTTCAATTTAGTTGTGT

TTGCCCATGAAGCCCATGTCAAAAAAAAAAA

>104

AGGACTTTTGGTTATTCTCCTACATAGGTAGAGCCTCCTTGACCCTCTGATCGGGTCGCT

ACGTTGTGTGATGAAAACCATTCCCGAGTCACAGAGTACTTGTACCTGTATCGTTGTTCA

AAGTCTCATGGATATTGTGCTACTTTTCTAGCACATGTTGTAAACTTTTCCTTGTAATTG

AGTGATGAATGTAACGCTTCAAAAAAAAAAA

>105

TTTAGGGGATAGAAGGATGATACATTGCCAAGGTCAAATGAGCTACTCACCCTTGGTTAG

CTTCAGCGGTTGCTAGTGTGGTAGGTGTGTATGATGGATGAACATGTCCAAGAATGGAGC

CTAAAGTGATGCAGACTTGAGGCTCAAGACATGTAGATAATTTTTTGACCAAATAAAATG

TGCAGATGTTTCCTGCTTTCAAAAAAAAAAA

>106

TCAAAAAATCTGATTACTCTCTTGCTTCATGCTCTACTATGCAAATCAAAAAACCCTATT

GCTCTCTTGCGTCATGCTTTCCAATGCAAATCAGAACACCTCACCACTATCTTACTTCAG

GCTCTACTGTCCAAATCAAAGAATATGGTTGCTCCCTTGCTTCATGCGCTTCTATGCAAA

TCAAAAAATCTGATTACTCTAAAAAAAAAAA

>107
[truncated: 7,774 more chars]
